# A clinical chemical atlas of xenobiotic toxicity for the Sprague–Dawley rat

**DOI:** 10.1007/s00204-025-04008-0

**Published:** 2025-05-06

**Authors:** Janonna Kadyrov, Samuele Sala, Lucy Grigoroff, Novia Minaee, Reika Masuda, Samantha Lodge, Timothy M. Ebbels, Michael D. Reily, Donald Robertson, Lois Lehman-McKeeman, John Shockcor, Bruce D. Car, Glenn H. Cantor, John C. Lindon, Jeremy K. Nicholson, Elaine Holmes, Julien Wist

**Affiliations:** 1 Australian National Phenome Centre, Health Futures Institute, Murdoch University, Perth, WA Australia; 2 Centre for Computational and Systems Medicine, Health Futures Institute, Murdoch University, Perth, WA Australia; 3 Department of Metabolism, Digestion and Reproduction, Faculty of Medicine, Hammersmith Campus, Imperial College London, London, W12 0NN UK; 4 Formerly Pfizer Global R&D, Ann Arbor, MI USA; 5 Formerly Bristol-Myers-Squibb Company, Princeton, NJ USA; 6 Formerly Drug Metabolism and Pharmacokinetics Section, Dupont Pharmaceuticals Company, Stine-Haskell Research Center, Newark, Delaware USA; 7 Department of Metabolism, Digestion and Reproduction, Faculty of Medicine, Imperial College London, London, UK; 8 Institute of Global Health Innovation, Faculty of Medicine, Imperial College London, London, SW7 2AZ UK; 9 Chemistry Department, Universidad del Valle, 76001 Cali, Colombia

**Keywords:** Clinical chemistry, Toxicity, COMET project, Biofluid, Rats, Sprague–Dawley

## Abstract

The Consortium for Metabonomic Toxicology (COMET) studies was designed to model metabolic responses to organ- and mechanism-specific toxins to predict acute drug toxicity in rats. A range of clinical chemical parameters were measured in 7-day toxicology studies for 86 toxins eliciting a range of organ- and mechanism-specific effects. Additionally, 21 surgical or physiological stressors were evaluated to identify physiological or metabolic responses that might confound the interpretation of observed toxicity profiles. From these studies on a total of 3473 rats measured at six pharmaceutical companies, we provide a set of 12 serum and 5 urine physical and clinical chemistry parameters. Samples were collected at 24 h, 48 h and 168 h post-dose for each animal and are presented as a downloadable database file. We also summarise the main observations based on the group response at the level of the individual toxin. We demonstrate that correlations between parameters, such as serum bilirubin and aspartate aminotransferase (AST), provide a more nuanced profile of organ-specific toxicity than consideration of individual parameters alone. In addition, we highlight the variability in the measured parameters across the dataset attributable to inter-laboratory differences, and the heterogeneity of metabolic responses to particular compounds or differences in temporal patterns of response. This clinical chemistry atlas of toxicity serves as a valuable reference tool for evaluating the potential toxicity of novel drug candidates.

**Supplementary Information:**

The online version contains supplementary material available at 10.1007/s00204-025-04008-0.

## Introduction

Drug safety issues are a major cause (ca 35–40%) of attrition of drug candidates (Tosca et al. 2021), with hepatotoxicity, cardiotoxicity and nephrotoxicity accounting for the majority of withdrawals from the market (Wilke et al. 2007). As part of the development of a new drug, a rigorous, multi-stage pipeline for safety testing is implemented involving a series of in vitro (cytotoxicity, genotoxicity, drug–drug interactions) and in vivo (acute, sub-acute, chronic, carcinogenicity) evaluations to ensure that new drug candidates are safe for human use (Walker et al. 2020; Stark and Steger-Hartmann 2015). Several tools have been adopted within the pharmaceutical industry for screening drug candidates with a view to minimising attrition and hence cost by early elimination of products associated with toxicity or adverse reactions. In silico screenings have contributed increasingly to reducing drug induced toxicity over the last few years (Amorim et al. 2024; Tran et al. 2023). The most common computational approaches are those based on quantitative structure–activity relationships which are rooted in a range of different machine learning approaches (Tosca et al. 2021). Such methods can be trained or validated on data from in vitro or in vivo studies and used as early screening tools.

Prior to clinical (human) testing, in vivo toxicity testing involves preclinical (non-human) evaluation, with one of the most common paradigms consisting of a 7-day acute toxicity study in rodents (typically mouse or rat). The rat has been a popular choice for acute toxicity evaluation based on the size, ease of handling, short breeding cycle and similarity to humans in response to multiple types of compounds (Smith et al. 2019). In addition, the historical use of rodent models in toxicity testing has resulted in the accumulation of vast amounts of data on clinical and physiological parameters. Although the pharmaceutical industry has increasingly come to rely on postgenomic technologies in the evaluation of drug safety, the 7-day toxicity study with conventional clinical chemistry tests and histological assessment remains a key feature of the safety evaluation for new drug candidates.

In 1999, six pharmaceutical companies (namely, Bristol Myers Squibb, Eli Lilly and Co., Hoffman–La Roche, NovoNordisk, Pfizer Incorporated, and The Pharmacia Corporation) and an academic partner (Imperial College London) came together to explore the formation of a consortium with the aim of generating a comprehensive toxicological database of NMR spectra anchored with conventional clinical chemistry and histopathology measurements to construct an expert system for prediction of organ- or mechanism-specific toxicity (Lindon et al. 2003). NMR-based metabolic phenotyping is an approach that can identify novel biomarkers of liver and kidney toxicity before any clinical effects occur, which would be invaluable in identifying hepatotoxicity- and nephrotoxicity-inducing drugs early on and preventing such drugs progressing through clinical trials and to market (Nicholson et al. 1989; Sanins et al. 1992; Nicholson et al. 2002; Olesti et al. 2021; Shockcor and Holmes 2002; Robertson 2005; Araújo et al. 2021; Robertson et al. 2011). Over a 3-year period starting in 2001, the consortium sought to study 86 acute toxins and 21 induced physiological conditions that confound the interpretation of toxicity data in the Sprague–Dawley rat, building databases and predictive models to better understand and mitigate drug toxicity. The resulting databases and models were used internally by several of the pharmaceutical company partners with the aim of improving the early identification of therapeutic candidates with adverse effects. Multiple publications reporting new understanding of toxic mechanisms (Lindon et al. 2005, 2003; Ebbels et al. 2007; Cantor et al. 2013; Bohus et al. 2008) and physiological responses associated with toxic side effects, for example weight loss (Veselkov et al. 2010) or liver regeneration (Bollard et al. 2010), were generated.

Although the main aim of the consortium was to generate information-dense metabolic profiles of each of the toxins using proton nuclear magnetic resonance spectroscopy (^1^H NMR) (Lindon et al. 2005), modelling the changes in the metabolic profiles required information from conventional toxicological endpoints such as histopathological assessment and clinical chemistry assays. Since this is one of the largest collections of rodent toxicology studies ever conducted under identical protocols across multiple laboratories, we are now making these data publicly available to provide reference ranges for the various data modalities. Here, we report the clinical chemistry medians and ranges for serum calcium, sodium, potassium, phosphate, glucose, albumin, total protein, total bilirubin, urea nitrogen, creatinine, alanine aminotransferase (ALT) and aspartate aminotransferase (AST), and for urinary volume, pH, osmolality, glucose and protein for the 107 treatments and for matched control animals across multiple time points. We provide a downloadable SQL database containing the curated clinical chemistry parameters at the individual animal level. Thus, this paper provides a clinical chemical atlas of liver, kidney, testicular and pancreatic toxicity based on standard laboratory parameters.

## Methods

### Experimental design

**Fig. 1:**
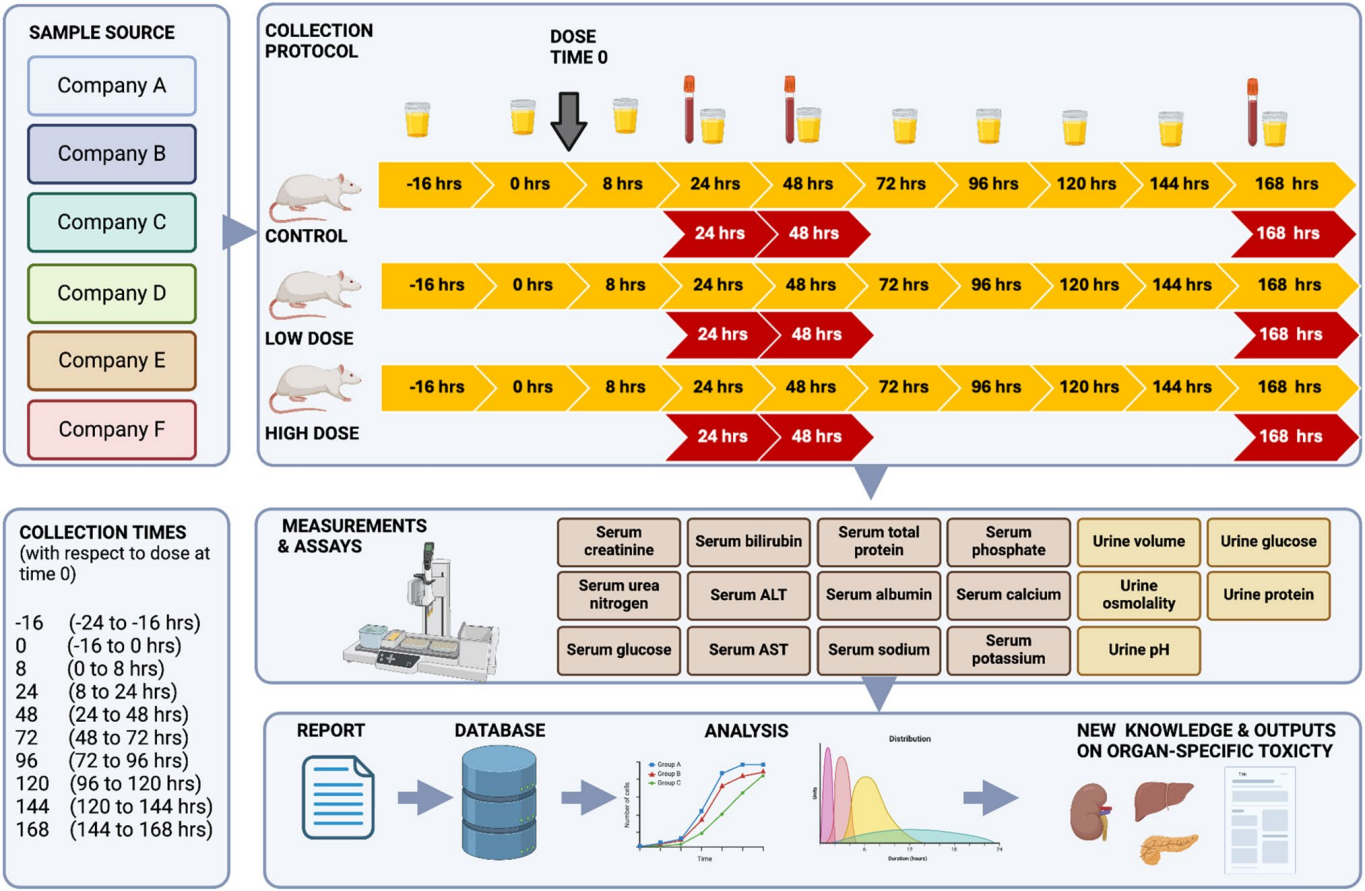
Schematic of the experimental design for the Consortium for Metabonomic Toxicology - COMET project

The study design for the 7-day toxicology experiments used in the COMET project is standard in most pharmaceutical companies and has been reported elsewhere but is described here briefly and summarised in a schematic diagram (Fig. 1). All animal studies were conducted in accordance with the current guidelines for animal welfare (Guide for the Care and Use of Laboratory Animals, 1996) and the procedures used were reviewed and approved by the Institutional Animal Care and Use Committee in each company. Studies were carried out within the laboratories of each of the companies (Bristol Myers Squibb, Eli Lilly and Co., Hoffman–La Roche, NovoNordisk, Pfizer Incorporated, and The Pharmacia Corporation), who have been randomly assigned a letter from A to F for the purpose of reporting the data. A total of 86 toxins that induce toxic effects to the liver, kidney, pancreas and/or testes were selected and administered in an appropriate dose vehicle, predominantly saline or corn oil, either orally or via an intraperitoneal injection as defined in Table 1. For each compound studied, 30 rats (8- to 10-week-old male Sprague–Dawley from Charles River Laboratories) were allocated randomly into a control group, a low-dose group and a high-dose group with dosing levels being based on the literature or earlier dose-range finding studies to generate a threshold and a clear toxic response, respectively (Lindon et al. 2005). In addition, 21 physiological stressors, summarised in Table 1, were studied to ascertain the metabolic effects of physiological processes that may compound those of the drugs and toxins administered. These treatments included partial hepatectomy, unilateral nephrectomy, chronic food or water restriction and administration of compounds that, for example, decrease renal tubular reabsorption (e.g. probenecid) (Silverman et al. 2008) or act as a carbonic anhydrase inhibitor (e.g. acetazolamide) (Molon-Noblot et al. 1992; Owen et al. 1993). The total number of studies carried out within the COMET project and the total number of studies for each target toxicity are presented in Supplementary Figure S1. Animals were acclimatised in standard cages (n = 5 animals per cage) for 2 weeks prior to the start of the experiment and subsequently transferred to individual metabolism cages that were under regulated temperature and humidity conditions (21 ± 2 °C and 55 ± 10% respectively) in a fluorescently lit cycle of 12 “daily” hours (06:00 to 18:00) and 12 “nightly” hours. All rats were allowed unlimited amounts of food (Purina Chow 5002) and given free access to water across the entire study, except in studies designed to measure the impact of food or water restrictions. The full protocol is provided in the Supplementary Material.

**Table 1:** List of toxin names with corresponding dose routes, vehicles, dosages and target organ of toxicity

Toxin	Dose route	Dose vehicle	Low dose	High dose	Affected organ
^E^ 1,1-Dichloroethylene	P.O	Corn oil	37.5 mg/kg	150 mg/kg	Liver
^E^ 1,2,3,4,5,6-Hexachlorocyclohexane	I.P	Corn oil	24 mg/kg	120 mg/kg	Liver
^B^ 1-Fluoropentane	I.P	0.9% saline	2 mg/kg	20 mg/kg	Liver
^B^ 2,4,6-Trihydroxyacetophenone (THA)	I.P	2% DMSO in corn oil	40 mg/kg	400 mg/kg	Liver
^B^ 4-Amino-2,6-dichlorophenol (ADCP)	I.P	2% DMSO in corn oil	7 mg/kg	70 mg/kg	Liver
^C^ Aflatoxin	P.O	1% carboxymethyl cellulose	0.2 mg/kg	2 mg/kg	Liver
^C^ Allyl alcohol	P.O	0.9% saline	12 mg/kg	120 mg/kg	Liver
^C^ Allyl formate	I.P	0.9% saline	25 mg/kg	50 mg/kg	Liver
^B^ Azathioprine	P.O	10% acacia + 0.1% antifoam	30 mg/kg	300 mg/kg	Liver
^B^ Bromobenzene	P.O	Corn oil	150 mg/kg	1500 mg/kg	Liver
^C^ Butylated hydroxytoluene	P.O	Corn oil	100 mg/kg	1000 mg/kg	Liver
^D^ Carbon tetrachloride	P.O	Corn oil	640 mg/kg	3200 mg/kg	Liver
^C^ Chlorpromazine	I.P	0.9% saline	30 mg/kg	60 mg/kg	Liver
^B^ Clofibrate	P.O	0.25% methyl cellulose	100 mg/kg	1000 mg/kg	Liver
^B^ Cyproterone acetate	I.P	Corn oil	40 mg/kg	400 mg/kg	Liver
^A^ D-Galactosamine	I.P	0.9% saline	50 mg/kg	500 mg/kg	Liver
^B^ Diethylhexylphthalate (DEHP)	P.O	Distilled water	2000 mg/kg	20,000 mg/kg	Liver
^C^ Dimethylformamide (DMF)	I.P	0.9% saline	250 mg/kg	1000 mg/kg	Liver
^C^ Dimethylnitrosamine (DMN)	P.O	Sterile water	1.5 mg/kg	15 mg/kg	Liver
^A^ Gadolinium chloride	I.V	0.9% saline	1 mg/kg	10 mg/kg	Liver
^A, B, C, D, E, F^ Hydrazine	P.O	0.9% saline	30 mg/kg	90 mg/kg	Liver
^E^ Indomethacin	P.O	0.2% carboxymethyl cellulose	5 mg/kg	25 mg/kg	Liver
^E^ Ketoconazole	P.O	0.9% saline	50 mg/kg	350 mg/kg	Liver
^C^ Lead acetate	I.P	Sterile water	11.4 mg/kg	114 mg/kg	Liver
^A^ Lipopolysaccharide (LPS)	I.V	0.9% saline	0.5 mg/kg	5 mg/kg	Liver
^B^ Methapyrilene	P.O	Distilled water	20 mg/kg	200 mg/kg	Liver
^E^ Methylene dianiline	P.O	Corn oil	13 mg/kg	50 mg/kg	Liver
^C^ Monocrotaline	P.O	Sterile water	80 mg/kg	160 mg/kg	Liver
^C^ N-methylformamide (NMF)	I.P	0.9% saline	400 mg/kg	1000 mg/kg	Liver
^D^ Phalloidin (chronic)	I.P	0.9% saline	0.25 mg/kg	0.75 mg/kg	Liver
^E^ Phenyl diisothiocyanate	I.P	Corn oil	2.25 mg/kg	11 mg/kg	Liver
^E^ Phenyl isothiocyanate	I.P	Corn oil	20 mg/kg	100 mg/kg	Liver
^B^ Retinyl palmitate	P.O	10% acacia + 0.1% antifoam	50 mg/kg	500 mg/kg	Liver
^B^ Sodium valproate	P.O	Distilled water	200 mg/kg	2000 mg/kg	Liver
^C^ α-Naphthylisothiocyanate (ANIT)	P.O	Corn oil	12.5 mg/kg	125 mg/kg	Liver
^D^ 2-Bromophenol	I.P	Corn oil	100 mg/kg	200 mg/kg	Kidney
^E^ 3,5-Dichloroaniline hydrochloride	I.P	0.9% saline	30 mg/kg	120 mg/kg	Kidney
^E^ Atractyloside	I.P	0.9% saline	10 mg/kg	100 mg/kg	Kidney
^D^ Bromoethylamine hydrobromide	I.P	0.9% saline	15 mg/kg	150 mg/kg	Kidney
^D^ Cephaloridine	I.P	0.9% saline	200 mg/kg	900 mg/kg	Kidney
^B^ Chlorethanamine	I.P	0.9% saline	100 mg/kg	1000 mg/kg	Kidney
^A^ Cisplatin	I.P	0.9% saline	1 mg/kg	5 mg/kg	Kidney
^A^ D-Limonene (chronic)	P.O	Corn oil	15 mg/kg	150 mg/kg	Kidney
^E^ Dichlorophenyl succinimide	P.O	Corn oil	43 mg/kg	170 mg/kg	Kidney
^D^ Ethylene glycol	P.O	0.9% saline	0.5 mL/kg	3.0 mL/kg	Kidney
^A^ Folic acid	I.P	150 mmol/L sodium bicarbonate solution	35 mg/kg	350 mg/kg	Kidney
^A^ Gentamicin	P.O	0.9% saline	50 mg/kg	150 mg/kg	Kidney
^B^ Maleic acid	P.O	Distilled water	40 mg/kg	400 mg/kg	Kidney
^A^ N-phenylanthranilic acid (chronic)	P.O	0.5% aqueous methyl cellulose	65 mg/kg	650 mg/kg	Kidney
^D^ Para-aminophenol	I.P	0.9% saline	40 mg/kg	150 mg/kg	Kidney
^A^ Puromycin	I.P	0.9% saline	5 mg/kg	150 mg/kg	Kidney
^B^ Vancomycin hydrochloride	I.V	0.9% saline	20 mg/kg	200 mg/kg	Kidney
^E^ Acetaminophen	P.O	0.2% carboxymethyl cellulose	400 mg/kg	1600 mg/kg	Liver & Kidney
^B^ Aurothiomalate	I.P	0.9% saline	10 mg/kg	100 mg/kg	Liver & Kidney
^C^ Chloroform	P.O	Corn oil	50 mg/kg	500 mg/kg	Liver & Kidney
^D^ Cyclosporin	P.O	Corn oil	70 mg/kg	700 mg/kg	Liver & Kidney
^D^ Dichlorobenzene	P.O	Corn oil	75 mg/kg	400 mg/kg	Liver & Kidney
^C^ Ethionine	P.O	Methyl cellulose	200 mg/kg	800 mg/kg	Liver & Kidney
^B^ Hexachlorobutadiene (HCBD)	P.O	Corn oil	20 mg/kg	200 mg/kg	Liver & Kidney
^B^ Mercuric chloride	I.P	0.9% saline	0.5 mg/kg	2 mg/kg	Liver & Kidney
^E^ Microcystin-LR	I.P	0.9% saline	0.02 mg/kg	0.08 mg/kg	Liver & Kidney
^E^ Rotenone	P.O	Corn oil	10 mg/kg	100 mg/kg	Liver & Kidney
^E^ S-(1,2-dichlorovinyl)-cysteine (DCVC)	I.P	0.9% saline	6 mg/kg	60 mg/kg	Liver & Kidney
^D^ Thioacetamide	I.P	0.9% saline	20 mg/kg	100 mg/kg	Liver & Kidney
^E^ 1-Cyano-2-hydroxy-3-butene	I.P	0.9% saline	15	150	Pancreas
^C^ Caerulein	I.P	0.05 M sodium hydroxide in 0.9% saline	0.05 mg/kg	0.2 mg/kg	Pancreas
^E^ L-arginine	I.P	0.9% saline	1000 mg/kg	4000 mg/kg	Pancreas
^B^ Streptozotocin	I.P	10 mM citrate buffer	25 mg/kg	60 mg/kg	Pancreas
^D^ 1,3-Dinitrobenzene	P.O	Corn oil	10 mg/kg	30 mg/kg	Testicular
^C^ Cadmium chloride	P.O	Sterile water	7.5 mg/kg	75 mg/kg	Testicular
^D^ Cadmium chloride	I.P	0.9% saline	0.75 mg/kg	1.5 mg/kg	Testicular
^D^ Carbendazim	P.O	Corn oil	50 mg/kg	400 mg/kg	Testicular
^D^ Di-n-pentyl-phthalate	P.O	Corn oil	1100 mg/kg	2200 mg/kg	Testicular
^D^ Ethane dimethane sulphonate (EDS)	I.P	DMSO:water (1:3)	30 mg/kg	100 mg/kg	Testicular
^D^ Methoxyacetic acid	P.O	0.9% saline	150 mg/kg	650 mg/kg	Testicular
^B^ Adriamycin	I.V	0.9% saline	2 mg/kg	10 mg/kg	Multiple organ
^C^ Amphotericin B	I.P	Sterile water	5 mg/kg	50 mg/kg	Multiple organ
^C^ Azaserine	I.P	0.9% saline	8 mg/kg	80 mg/kg	Multiple organ
^A^ Dexamethasone	I.P	10% DMSO in corn oil	2 mg/kg	100 mg/kg	Multiple organ
^E^ Mitomycin-C	I.P	0.9% saline	1	4	Multiple organ
^C^ 1,1-Dichloroethylene and maleic acid	P.O	Corn oil/sterile water	40 mg 1,1-dichloroethylene/kg in corn oil	200 mg maleic acid/kg in sterile water	Physiological stressor
^C^ 2,4-Dinitrophenol	P.O	Water	1.5 mg/kg	15 mg/kg	Physiological stressor
^B^ 4-Pentenoic acid	P.O	Corn oil	50 mg/kg	350 mg/kg	Physiological stressor
^D^ Acetazolamide	P.O	0.5% methyl cellulose	60 mg/kg	600 mg/kg	Physiological stressor
^C^ Acivicin	I.P	0.9% saline	1 mg/kg	10 mg/kg	Physiological stressor
^E^ Ammonium chloride	P.O	0.9% saline	0	0.02	Physiological stressor
^D^ Carboplatin	I.V	0.9% saline	30 mg/kg	60 mg/kg	Physiological stressor
^A^ Choline and choline/methionine deficiency (chronic)	Diet modification		Choline devoid (CD) diet	Choline + methionine devoid (CMD) diet	Physiological stressor
^B^ Food restriction (chronic)	Diet modification		75% of the regular food intake	0% of the regular food intake for 24 h	Physiological stressor
^D^ Furosemide	P.O	0.9% saline	10 mg/kg	50 mg/kg	Physiological stressor
^B^ Insulin	S.C	0.9% saline	10 IU/kg	750 IU/kg	Physiological stressor
^E^ Methotrexate	P.O	0.2% carboxymethyl cellulose	40 mg/kg	400 mg/kg	Physiological stressor
^A^ Partial hepatectomy	Surgical study		Sham surgery (SS)	Partial hepatectomy (PH)	Physiological stressor
^A^ Phenobarbital (chronic)	I.P	0.9% saline	10 mg/kg	100 mg/kg	Physiological stressor
^A^ Pregnenolone 16 alpha carbonitrile (chronic)	P.O	Carboxymethyl cellulose	10 mg/kg	100 mg/kg	Physiological stressor
^A^ Probenecid	I.P	0.9% saline	20 mg/kg	200 mg/kg	Physiological stressor
^C^ Rosiglitazone	P.O	1% methyl cellulose	100 mg/kg	500 mg/kg	Physiological stressor
^C^ Rosiglitazone (chronic)	P.O	1% methyl cellulose	10 mg/kg	100 mg/kg	Physiological stressor
^E^ Sodium bicarbonate	P.O	Drinking water	0.1 M	0.35 M	Physiological stressor
^A^ Unilateral nephrectomy	Surgical study		Sham surgery (SS)	Unilateral nephrectomy (UN)	Physiological stressor
^B^ Water deprivation (chronic)	Diet modification		Deprived for 24 h	Deprived for 48 h	Physiological stressor
^E^ Acetaminophen (chronic)	P.O	0.2% carboxymethyl cellulose	200 mg/kg	800 mg/kg	No Effect
^C^ Buthionine sulphoxime	I.P	0.9% saline	222 mg/kg	889 mg/kg	No Effect
^C^ Ferrous sulphate	P.O	Distilled water	250 mg/kg	1250 mg/kg	No Effect
^B^ Ifosfamide	P.O	10% acacia + 0.1% antifoam	10 mg/kg	100 mg/kg	No Effect
^B^ Lithocholic acid	P.O	10% acacia + 0.1% antifoam	10 mg/kg	100 mg/kg	No Effect
^E^ Paraquat	I.P	0.9% saline	5 mg/kg	22.5 mg/kg	No Effect
^D^ Potassium dichromate	I.P	0.9% saline	10 mg/kg	20 mg/kg	No Effect
^C^ Trichlorethylene	I.P	Corn oil	200 mg/kg	1000 mg/kg	No Effect

_A-F: indicates pharmaceutical company and sample origin_

### Sample collection

Urine samples were collected across an 8-day period including both day and night time points. All animals were placed in metabolism cages 48 h prior to the first urine collection to allow the animals to acclimatise. Urine samples were collected at 16 h pre-dose covering the continuous collection over the 8 h period from −24 h to −16, then 0 (−16–0 h pre-dose), 8 (0–8 h post-dose), 24 (8–24 h post-dose), 48 (24–48 h post-dose), 72 (48–72 h post-dose), 96 (72–96 h post-dose), 120 (96–120 h post-dose), 144 (120–144 h post-dose) and 168 (144–168 h post-dose) as described in Fig. 1. Serum samples were collected from all rats via tail vein puncture 24 h after dosing. Half the rats from each group were euthanised at 48 h post-dose and the other half at 168 h post-dose to assess both the acute and chronic effects of each toxin. Serum and tissue samples were collected upon euthanisation, and histopathological examination was conducted. Serum samples were split into two aliquots, one for in-house clinical chemistry analysis and one for proton nuclear magnetic resonance (^1^H NMR) spectroscopic analysis (performed at Imperial College London). The urine samples were collected into containers with 1 mL of 1% w/v sodium azide as a bactericide and maintained at 0 ± 2 °C. On collection, the urine volume was recorded and approximately one-third of each urine sample was transferred to a tube for urinalysis and submitted to clinical pathology. The remainder of the sample was centrifuged for 10 min at ~ 1200 g and subsequently stored at a temperature of –40 °C until NMR analysis. Urinary volume was recorded, and osmolality was measured using a freezing point depression osmometer and all clinical assays were performed using standard in-house protocols.

### Data curation and statistical analyses

Improbable values in the clinical chemistry data, for example negative concentration values, were identified through basic univariate statistic and removed. Parameters with more than 40% data missing were also removed. A random forest (RF) imputation approach, using the missForest (R package v1.5), was applied to all missing clinical values (number and percentage of missing values for each parameter is provided in Supplementary Table S1) in the entire dataset (control, low-dose and high-dose samples). The performance of the imputation method was validated through comparing the distributions of the imputed dataset to the original dataset using half violin boxplots (Figure S2-S4) with accompanying normalised root mean square error (NRMSE) values for each parameter. For the purpose of illustration, the distributions for the control, low-dose and high-dose samples are shown in Figures S2, S3 and S4. The distribution prior and post-imputation is shown in green and purple respectively for each clinical parameter. The normalised root mean square error (NRMSE) values generated using the missForest algorithm are also reported along with the percentage and number of missing values within each dose group. The distributions for both the original and imputed datasets are symmetrical across all parameters and dose groups, validating that the imputation process did not alter the overall distribution of the data.

Univariate statistics for each parameter were summarised for all control samples (*n* = 13,200). The median, minimum, maximum, mean and standard deviation values are provided in Supplementary Table S2. Additionally, similar values were computed for each individual toxin and for every parameter at 24 h, 48 h and 168 h post-dose vehicle and post-high-dose administration and are provided in the Supplementary Material (Supplementary Table S3-S19 post-dose vehicle; Supplementary Table S20-S36 post-high dose). To visualise the impact of the toxins on the clinical chemistry parameters, scatter plots were generated from all toxins using samples from 24 h post-dose. Correlation matrices, based on the Spearman’s correlation coefficient, were generated for the control dataset and for subsets of the high-dose administered data using samples from all time points and stratified by toxicity type: liver; kidney; liver and kidney; pancreas and testes, to visualise the correlation between clinical chemistry parameters and to identify organ-specific toxicity profiles.

## Results

### An atlas in SQL format

The summary serum clinical chemistry values (median, min, max, mean, standard deviation) for control Sprague–Dawley rats for the COMET project at 24 h, 48 h and 168 h post-injection with dosing vehicle are listed in Supplementary Tables S3-S19, stratified by main organ or tissue of toxicity and identified by the source of sample origin (pharmaceutical company). Summary statistics for serum parameters are listed by assay: calcium (Supplementary Table S3), sodium (Supplementary Table S4), potassium (Supplementary Table S5), phosphate (Supplementary Table S6), glucose (Supplementary Table S7), albumin (Supplementary Table S8), total protein (Supplementary Table S9), total bilirubin (Supplementary Table S10), urea nitrogen (Supplementary Table S11), creatinine (Supplementary Table S12), ALT (Supplementary Table S13) and AST (Supplementary Table S14). Urine clinical chemistry parameters for matched time points to the serum samples are provided in Supplementary Tables S15-S19. Urine clinical chemistry summary statistics are provided for urinary volume (Supplementary Table S15), pH (Supplementary Table S16), osmolality (Supplementary Table S17), glucose (Supplementary Table S18) and protein (Supplementary Table S19). The corresponding summary clinical chemistry values for rats post-high-dose administration are listed in Supplementary Tables S20-S36. The raw values, batch corrected values and batch corrected with imputed values for each sample are provided as open-source sqlite^1^ database file allowing readers to readily explore the impact of dose vehicle, route and toxin on the panel of standard clinical assays, as well as to explore inter-animal variability. With 26,546 entries for 3545 animals, this is the largest body of clinical chemistry data collected under a common protocol published for acute rodent toxicity studies and provides a benchmark for control value ranges in Sprague–Dawley rats.

### Assessment of toxicological effect

Although the model compounds were selected based on literature reports of toxic effect, not all toxins, sometimes even at high dose, were found to induce reproducible histopathological consequences as indicated for animals euthanised at 48 h and 168 h. Treatments that failed to elicit a toxic response, as defined by histopathology are listed at the base of Table 1. For these compounds, none of the clinical chemistry measurements were significantly out of the control range. It should be noted that all studies were performed according to prevailing best laboratory practices in the professional pharmaceutical laboratories and so the observed variation for some compounds reflects intrinsic biological variation rather than procedural.

**Fig. 2:**
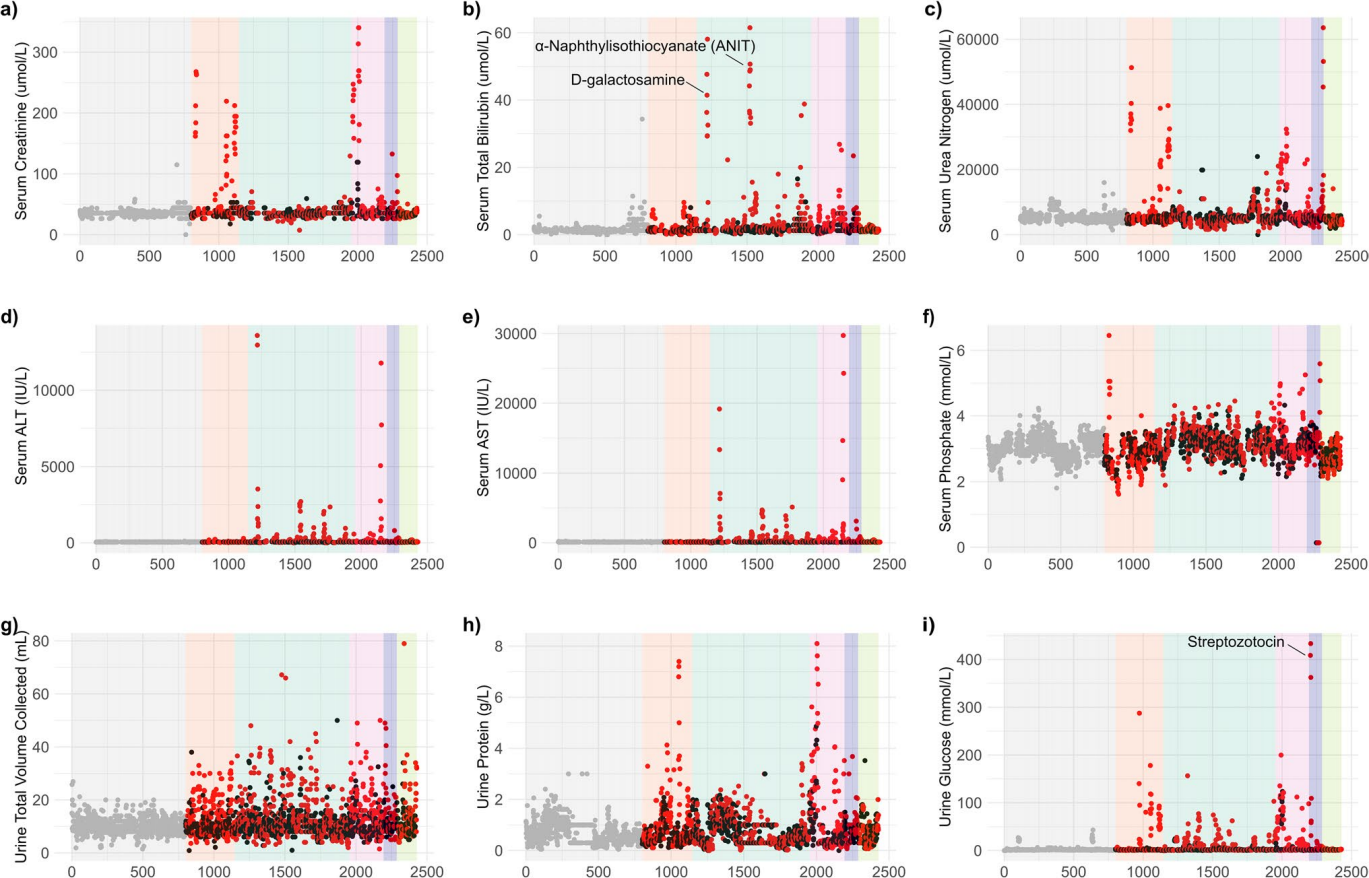
Scatter plots of clinical chemistry parameters using samples taken 24 h post-dose. Control, low-dose and high-dose groups are represented as grey, black and red points, respectively. Background colour corresponds to the target organ of toxicity: control (grey), kidney (orange), liver (dark green), liver and kidney (pink), pancreas (purple) and testes (light green). Abbreviations: aspartate aminotransferase (AST), alanine aminotransferase (ALT)

For certain parameters, the baseline was noisy with a high degree of variation in measurement values apparent in the control groups as well as the dosed, making it difficult to conclusively attribute small changes in mean or median values to toxic effect. The relative impact of the model toxins and clinical chemistry values at 24 h post-dose can be visualised in Fig. 2. Scatter plots for the parameters demonstrated good reproducibility. Both the low- and high-dose groups for each toxin were denoted by black and red data points, respectively, and contrasted with the control samples depicted as grey data points. The data points were ordered consecutively by study, with control samples placed in a separate category on the left-hand side of the scatter plot for ease of visualisation. The treated samples were ordered by primary organ of toxicity, indicated by the shaded background: control (grey), kidney (orange), liver (dark green), liver and kidney (pink), pancreas (purple) and testes (light green). As expected, this stratification of the data by organ of toxicity shows ‘spikes’ in the overall distribution of data points for a given parameter. In addition, toxins that targeted the same organ showed expected similarities in their clinical chemical profiles, although not all toxins targeting the same organ demonstrated equivalent profiles due to differences in the extent of the pathological lesions and differences in the time of response of individuals. For example, two distinct spikes in the total serum bilirubin plot (Fig. 2b) are associated with the high dose for d-galactosamine and α-naphthylisothiocyanate (ANIT), both being potent liver toxins. Similarly for urinary glucose (Fig. 2i), the ‘spike’ is in the pancreatic band and associated with streptozotocin. Also, there is a large dynamic range of response in some of the parameters, for example aspartate aminotransferase (AST).

### Correlation between parameters enhances the predictivity of the toxicity type

**Fig. 3:**
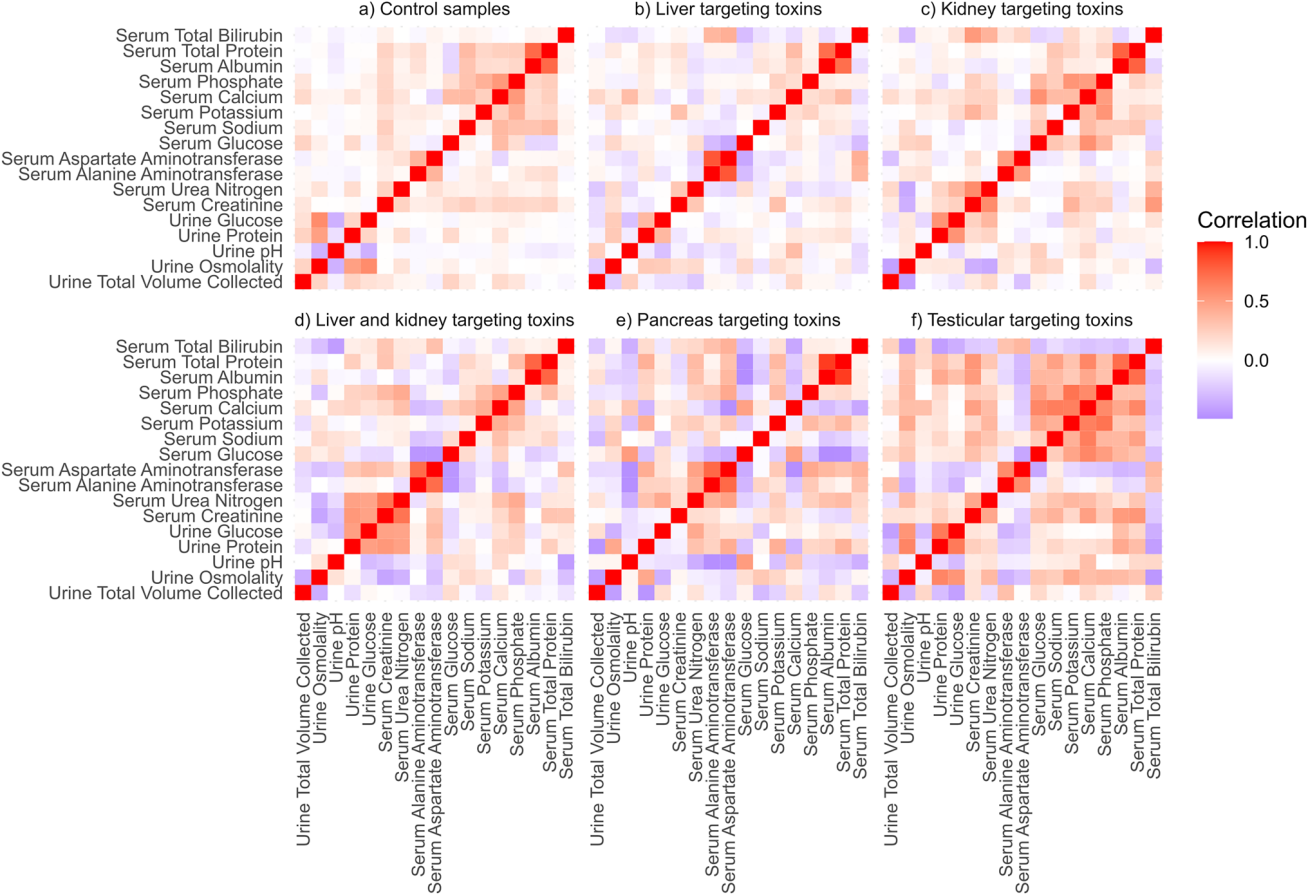
Spearman’s correlations between clinical chemistry parameters using all time points for control samples (**a**), and high-dose samples that were administered. Liver-targeting toxin (**b**), kidney-targeting toxin (**c**), liver and kidney-targeting toxin (**d**), pancreas-targeting toxin (**e**) and testicular-targeting toxin (**f**)

To obtain a more generalised overview of response across the different toxicity types, correlation matrices (Fig. 3) between the clinical chemistry parameters using samples from all time points were constructed. For control animals (Fig. 3a), a strong inter-parameter correlation was evident between total serum protein and serum albumin (*r* = 0.74), while weaker correlations were observed between urine glucose and osmolality (*r* = 0.59), serum calcium and phosphate (*r* = 0.52) and urine protein and osmolality (*r* = 0.49). Urine glucose and osmolality were weakly anti-correlated with urine pH in the control dataset (*r* = −0.34 urine glucose to pH; *r* = −0.33 osmolality to pH). In the case of liver toxicity (Fig. 3b), AST, ALT and bilirubin were all correlated with each other (*r* = 0.82 AST to ALT; *r* = 0.41 AST to bilirubin, *r* = 0.38 ALT to bilirubin) and negatively correlated with serum glucose (*r* = −0.38 AST to glucose; *r* = −0.29 ALT to glucose; *r* = −0.2 bilirubin to glucose). The correlation between total serum protein and serum albumin observed for the control dataset was retained, but not the anti-correlation between urine pH and osmolality. Instead, a weak anti-correlation was noted between urine glucose and pH (*r* = −0.27). The correlation matrix for renal toxicity (Fig. 3c) showed a different inherent pattern from control or from hepatotoxicity. Here, the strongest correlations were between total serum protein and serum albumin (r = 0.77) and between urea nitrogen and serum creatinine (*r* = 0.59). As observed for hepatotoxins, an inverse association was apparent between urine volume and osmolality (*r* = −0.40). As expected, the correlations for toxins targeting the liver and kidney (Fig. 3d) showed characteristics of both the individual correlation matrices for liver and kidney toxins. For pancreatic toxins (Fig. 3e), the strongest correlation was between total serum protein and albumin (*r* = 0.91), and AST and ALT (*r* = 0.75). For testicular toxicity (Fig. 3f), the defining features were direct correlations between serum ions calcium, potassium and phosphate (*r* = 0.70 potassium to phosphate, *r* = 0.64 potassium to calcium; *r* = 0.60 calcium to phosphate), and correlations between urine glucose and urine protein (*r* = 0.67), and serum calcium and serum sodium (*r* = 0.65). As for hepatotoxins, a strong association between total serum protein and albumin (*r* = 0.73) was observed.

## Discussion

We present a summary of clinical chemistry data for one of the largest academic acute toxicology projects yet commissioned as a reference database for Sprague-Dawley rats. We summarise the statistics for control Sprague–Dawley rats (*n* = 3473) as well as 35 liver toxins, 17 kidney toxins, 12 toxins targeting both liver and kidney, 4 pancreatic toxins, 6 testicular toxins and 5 toxins targeting multiple organs. In addition, we present data for 21 surgical and physiological stressors to allow for the characterisation of specific metabolic responses that may be part of, or may confound the predominant toxicological response. All studies were carefully planned so that all treatments were under the most similar conditions possible, including food sources and all sampling regimes. We also provide data for eight treatments that elicited no demonstrable effect, as ascertained by histopathology and clinical chemistry. All raw and curated data are provided as an SQL database (before and after batch correction and imputation) to facilitate researchers to reuse and integrate the data into their own models and databases.

Based on data acquired at 24 h post-dose, we show organ-specific patterns in clinical chemistry parameters using both univariate and correlation analyses. As expected, there is a range of magnitudes in the response to toxins, even within a set of organ-specific toxins. For example, elevated serum concentrations of AST and ALT, together with bilirubin, are classically associated with liver toxicity, but the range associated with each liver toxin is different such that some toxins are associated with values that reflect between a 0.5- and 28-fold increase in serum bilirubin. For example, α-naphthylisothiocyanate and D-galactosamine demonstrate a response that is an order of magnitude greater than other liver toxins (Fig. 2b). The differences in magnitude relate to the extent of damage, the time of collection and the sub-tissue and/or mechanism of pathology. Some parameters show contrasting behaviour in response to toxins targeting a particular organ. For example, whereas most liver toxins elicit an increase in liver enzymes AST and ALT, the steatotic liver toxin hydrazine is a transaminase inhibitor, and in this case causes a decrease in the concentration of these enzymes compared to the mean values for control animals as previously noted (Garrod 2005).

One of the disadvantages of relying on clinical chemistry parameters as measures of toxic response, is the fact that many serum and/or urinary clinical markers are transitory following a single dose alone of a toxic compound. This is particularly true for enzymes such as AST and ALT, whereby it is feasible that a peak in enzyme release from the cells into the bloodstream may be missed as enzymes tend to be present in short bursts (Nicholson et al. 2002; Ramaiah 2007). As toxicity profiles were generated for 24, 48 and 168 h (serum samples), and only the 24 h post dose samples were modelled here for illustrative purposes, it is almost certain that the exposure window was not optimal for most compounds.

The power of considering multivariate relationships between clinical parameters, thereby capturing interactions between parameters, is illustrated by the correlation plots in Fig. 3 and could be further extended to multivariate modelling. For example, an increase in urine glucose is a key feature of both kidney and pancreatic toxicity, but only for pancreatic toxicity is this accompanied by elevations in serum glucose and reflects the osmotic ‘drag’ created by the high concentration of glucose in the renal tubules which causes diffusion of water into the tubules to maintain osmotic balance. Organs are not typically homogeneous in structure and multiple studies have described variation in clinical chemistry parameters according to the site of toxicity within an organ. For example, necrosis of the renal papilla is more likely to induce vast increases in urine volume and decreases in osmolality compared to a toxin that targets the renal cortex, whereas a toxin that induces necrosis in the renal proximal convoluted tubule will manifest as an inability to reabsorb glucose, electrolytes and other compounds resulting in an increase of these species in urine (Radi 2019). Similarly, the mechanism of action of a toxin can result in differences in the clinical chemistry signature. An obvious example would be a hepatotoxin such as hydrazine that inhibits transaminases compared to a cholestatic hepatotoxin such as galactosamine that causes release of transaminases into the blood. Those data provided here are derived from toxins targeting a range of sites (e.g. glomerulus, cortex and papilla within the kidney) and a range of mechanisms (e.g. cholestasis, steatosis, necrosis) allowing readers to select appropriate benchmarks as comparators for their own studies. Indeed, the characterisation of a particular compound as a “liver toxin” or “kidney toxin” is overly simplistic as highlighted in this compendium of data.

Finally, the provision of a large body of clinical chemistry data for control Sprague–Dawley rats shows stratification according to pharmaceutical company and study. These observed inter-laboratory differences are most likely a product of minor differences in laboratory environment and although the study protocol extended to details such as batch of chow diet, the source of the animals and their exposure to environmental differences prior to the study may account for some of these differences (Ericsson and Franklin 2021; Robosky et al. 2005). The ranges we report here largely match the values reported in the literature. In the case of serum sodium, calcium and albumin (Supplementary Table 2), the values reported substantially overlap with those reported by Petterino and Argentino-Storino (2006). However, for some parameters, there are significant differences between those reported in the literature for Sprague–Dawley rats. Serum urea nitrogen (umol/L) and total bilirubin (umol/L) are two such examples where we find a range of 1430–16000 with a median, mean and standard deviation of 5000, 5237 and 1143, respectively, for urea nitrogen and a range of 0–34.34 with a median, mean and standard deviation of 1.2, 1.42 and 1.1, respectively, for total bilirubin. As we provide the raw measurement values in addition to batch-corrected and imputed data, it is possible for us to estimate what percentage of ‘spurious’ or improbable values would be expected in a large study and to provide a baseline for improving power calculations. 

## Conclusion

We provide an atlas of toxic effects of a wide range of drugs and model toxins with different sites and mechanisms of toxicity. To our knowledge, this is the largest collated set of clinical chemistry parameters published for rodent toxicity models and should help provide a resource for benchmarking acute toxicity studies in rat models. Tables with basic statistics (median, range, mean and standard deviation) for controls and high-dosed samples are available in the Supplementary Information. The raw values, values after outlier removal, values after batch correction and values after imputation are provided as sqlite tables (See Data Availability).

## Supplementary Information

Below is the link to the electronic supplementary material.

Supplementary file1 (PDF 6711 KB)

## Data Availability

The datasets generated during and/or analysed during the current study are available in the zenodo.org repository, 10.5281/zenodo.14963725.

## Footnotes

1 Sqlite databases can be shared as a regular file, i.e., without the requirement to install and configure a database server. It is an industry grade open-source format that allows to perform queries in SQL language and that will work on any operating system.
