# Supplementary material for: A clinical chemical atlas of xenobiotic toxicity for the Sprague–Dawley rat
Source: Arch Toxicol. 2025 May 6;99(6):2669–81. doi: 10.1007/s00204-025-04008-0 (PMC12185569; doi:10.1007/s00204-025-04008-0)
Supplement: Supplementary file 1 — Supplementary file1 (PDF 6711 KB) [file 204_2025_4008_MOESM1_ESM.pdf]

A Clinical Chemical Atlas of Xenobiotic Toxicity for the Sprague-Dawley Rat

Janonna Kadyrov[1,2], Samuel Sala[1,2], Lucy Grigoroff[1,2], Novia Minaee[1,2], Reika Masuda[1,2], Samatha Lodge[1,2], Timothy M. Ebbels[3], Michael D. Reily[4], Donald Robertson[4], Lois Lehman-McKeeman[5], John Shockcor, Bruce D. Car[5], Glenn H. Cantor[6], John C. Lindon[7], Jeremy K. Nicholson[1,2,8]*, Elaine Holmes[1,2,7]*, Julien Wist[1,2,8,9]*

[1] *Australian National Phenome Centre, Health Futures Institute, Murdoch University, Perth, WA, Australia.*

[2] *Centre for Computational and Systems Medicine, Health Futures Institute, Murdoch University, Perth, WA, Australia.*

[3] *Biological Chemistry, Biomedical Sciences Division, Faculty of Medicine, Imperial College of Science, Technology and Medicine, London, UK*

[4] *Pfizer Global R&D, Ann Arbor, Michigan, USA*

[5] *Bristol-Myers-Squibb Company, Princeton, New Jersey, USA*

[6] *The Pharmacia Corporation, Kalamazoo, Michigan, USA*

[7] *Department of Metabolism, Digestion and Reproduction, Faculty of Medicine, Imperial College London, London, UK.*

[8] *Institute of Global Health Innovation, Faculty of Medicine, Imperial College London, London, UK.*

[9] *Chemistry Department, Universidad del Valle, 76001, Cali, Colombia.*

*Correspondence to Julien.wist@murdoch.edu.au (database and computation); Elaine.Holmes@murdoch.edu.au (protocols and data analysis) and Jeremy.Nicholson@murdoch.edu.au (study design and toxicology)

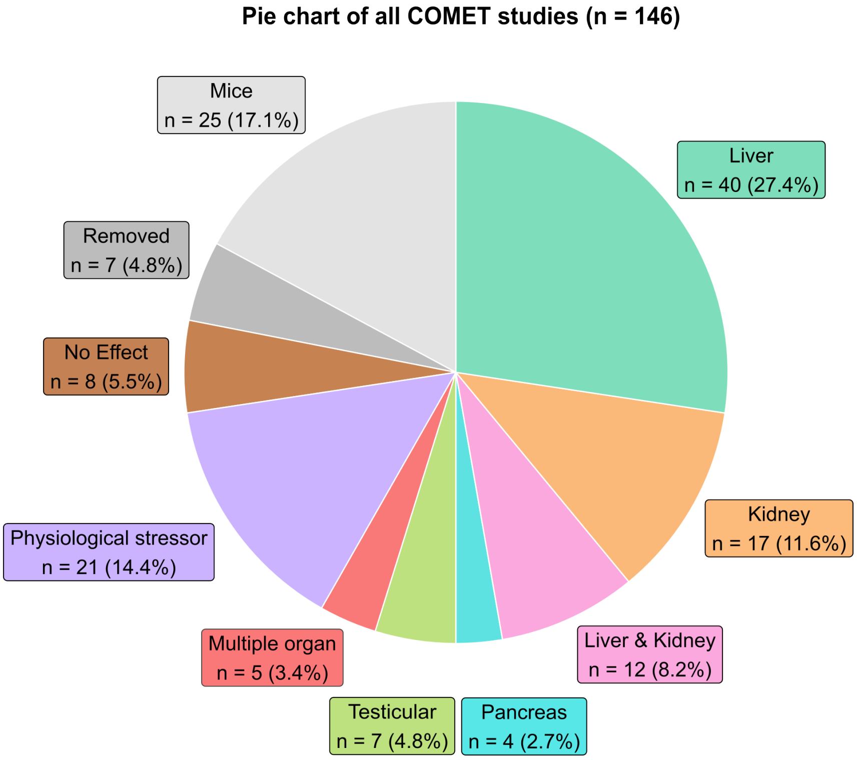

Figure S1. Pie chart of all toxin studies (n = 146) in the COMET project. There was a total of 120 studies using Sprague Dawley rats (Crl:CD (SD) IGS BR), 1 study using Hans Wistar rats (HanBrl:WIST(SPF)), and 25 studies using mice (B6C3F1).

The 25 mice studies were excluded from the paper along with 7 rat studies (1 that used Hans Wistar rats; 3 that failed and were each repeated as a separate study; 1 that was a surgical pilot study with no standard COMET data; and 2 that were a single dose and multidose study of WY14,643).

Of the remaining 114 studies, 6 examined the effects of hydrazine, 2 of cadmium chloride, 2 of acetaminophen and 2 of rosiglitazone. Therefore, there was a total of 106 different toxic compounds and mechanisms eliciting a toxic response (n = 85 toxins; n = 21 physiological stressors) that were reported in this paper.

Standard Protocol for Toxicology Studies

Animals and Maintenance

**Species/strain**	Rat/Sprague-Dawley (Crl:CD(SD)IGS BR).
**Number/sex animals**	10 males per group (spare animals available).
**Supplier**	Charles River Ltd.
**Requisition No.**	
**Age/weight ordered**	6-8 wks at start of dosing. Assign to groups randomly.
**Caging**	Animals housed either individually or in small groups (5 per cage). 1 rat per metabolism cage from 48h before dosing.<br><br>Collection portions of all cages will be rinsed thoroughly with tap-water daily. All metabolism cages will be cleaned at least once and preferably more frequently during the study period on day 3-4 using standard cage washing procedures.
**Diet/water**	Purina chow 5002 (Record lot number).<br><br>Diet and drinking water will be freely available. Water source (tap, D/D etc) to be recorded.<br><br>Available information indicates that no contaminant is expected to be present in the diet or drinking water at a concentration that might affect the outcome of the study.
**Environment**	Controls set at 21±2°C, 55±10% relative humidity. Fluorescent lighting 06.00 to

	18.00 ± 1h. Deviations from these conditions will be reported.
**Animal Room Acclimatisation**	At least 5-7 days before allocation.
**Metab. Cage Acclimatisation**	48 hours prior to dosing animals will be placed in metabolism cages. The first 24 hours in the age will serve for acclimatisation and urine samples will not be collected.
**Acceptance on to study**	After clinical examination, before allocation.
**Allocation**	Arbitrarily assigned.
**Identification**	Mark with unique identifier or use implanted chip.
**Baseline collection period**	24h period immediately prior to dose.
**Replacements**	Spare animals may be used to replace any animals killed or dying on the day of dosing, with justification, after which they will no longer be considered part of the study.

Compound and Formulation

**Chemical Name and Structure**	To be Supplied.
**Rationale for dosing**	State rationale for both high and low dose levels. High dose should induce overt toxicity but with 100 % survival. Low dose should induce minimal clinical pathology/ histopathology – or 10% of high dose if insufficient information.
**Test article**	Use dose based on active moiety.
**Storage of test article**	Ambient temperature, protected from light if test substance does not require other conditions.
**Physical nature of formulations**	Solutions in saline, corn oil, methyl cellulose or approved vehicles.
**pH & Osmolality**	If necessary for saline solutions, adjusted to pH 6.5-7.5 and 250-350 mOsm/kg respectively by adding NaOH, HCl or NaCl as appropriate for i.p. administration. For oral dosing adjust pH to the physiologic range.
**Frequency of reformulation**	Formulations will be prepared, shortly before dosing – 1 ml to be retained for NMR analysis. Send to IC in dry ice with urine samples.

Groups and Doses

10 animals will be assigned to each group (control, low dose and high dose for each compound, unless the control group is shared for two or more compounds dosed in the same study).
5 animals from each group will be sacrificed at 48h p.d. (Subgroup A) and the remaining 5 animals (Subgroup B) will be sacrificed at 168h p.d.

Example of group sizes and numbering.

Compound	Vehicle (group)	Group number	No. animals in subgroup A (animal numbers) Sacrificed at 48h p.d.	No. animals in subgroup B (animal numbers) Sacrificed at 168h p.d.
None - Control	e.g. 0.9% saline	1	5 (1-5)	5 (6-10)
Compound A low dose	0.9% saline	2	5 (11-15)	5 (16-20)
Compound A high dose	0.9% saline	3	5 (21-25)	5 (26-30)

Dose administration

**Route**	p.o. if possible (i.p and i.v. are the 2nd and 3rd choices of route).
**Method**	Single administration.
**Volume (test and vehicle control)**	10 mL/kg body weight for oral dose, 10 mL/kg body weight for i.p. dose; the volume received by each animal will be calculated from the individual body weight on the day of dosing.
**Frequency/duration of treatment**	Single dose on day 1.

Measurements

Clinical observations

**- Throughout study**	General check of the condition and behaviour of all animals, availability of food and water, and gross environmental conditions, will be made.
**- Pre-test**	A detailed physical examination of all animals will be made.
**- During treatment**	All animals will be examined and signs recorded daily before dosing, as soon as practicable after dosing, at a reasonable time after dosing, and once daily thereafter.
**- Additional observations**	Additional observations will be made if warranted by findings.
**- Animal welfare**	Appropriate action will be taken if any animal is found to be in poor condition or is showing signs of pain or distress.
**Deaths**	The circumstances in which any animal is killed or dies during the treatment period (including those that are replaced and satellite animals) will be recorded, a necropsy performed and the tissues listed under **Terminal Studies** retained for possible examination.
**Body weights**	Subgroup B animals - daily, Pre-test (24h period 1-3 days prior to dosing) and at least every 3 days prior to necropsy but preferably more frequently.<br><br>Subgroup A animals - daily, Pre-test (24h period 1-3 days prior to dosing), day 1 and before necropsy on day 2.

Clinical Pathology

All data are to be recorded in an Excel template and send to IC together with the urine samples.

Blood sampling- time

A single blood sample will be collected 24h p.d. and 2 samples at necropsy (48h p.d.or 168h p.d.). The additional serum sample collected at termination (approximately 1 ml) will be shipped to IC (See Below).

Method

Approximately 0.80 mL per sample into glass microcentrifuge tubes, by puncture of tail vein or usual method. Serum or plasma will be analysed for clinical pathology parameters according to the requirements of each laboratory. Store at -70° C

Blood Chemistry

(on serum/plasma samples) All measurements to be quoted in I/U

Parameters measured
(listed in order of preference)

creatinine
blood urea nitrogen
alanine aminotransferase
aspartate aminotransferase
alkaline phosphatase
gamma glutamyl transferase
glucose
sodium
potassium
calcium
phosphorous
albumin
total protein
total billirubin

Urinalysis

All animals.

Method

Urine will be collected into labelled tubes maintained at (-2 to +4 °C) and will be stored frozen until they are analysed. Urine will be collected into tubes containing sodium azide (1

ml of 1% solution) over a period of 8 days which includes a 1 day baseline collection.

Day of Study	Time relative to dosing (h)		Sample label[a]
	Subgroup A	Subgroup B	
Pre-test	0-8h	0-8h	-16
Pre-test	8-24h	8-24h	0
1	0-8h	0-8h	8
1	8-24h	8-24h	24
2	24-48h	24-48h	48
3		48-72h	72
4		72-96h	96
5		96-120h	120
6		120-144h	144
7		144-168h	168

[s] Use this label for the data sheets

Samples should be centrifuged at 3000 rpm (500g) for 10 mins and the supernatant removed and stored at -70°C prior to shipping to IC.

Parameters measured

Note: Volume and pH measurements are necessary for each sample sent to IC.

volume
pH
Osmolality

For known nephrotoxins, the following parameters should also be measured:

glucose (quantitative if possible)
protein (quantitative if possible)

All measurements to be quoted in I/U

Assay methods to be recorded and normal ranges supplied.

Urine Sample Priority

The following list indicates the priority in which urine samples should be allocated for the various analyses in cases where there may be insufficient sample volume:

1. Imperial College (at least 2 mL)
2. Clinical Pathology Sample
3. Internal NMR Sample
4. Back-up IC sample (at least 2 mL)

Terminal studies

Procedure before necropsy

All animals will be killed (Subgroup A at 48h p.d., subgroup B at 168h p.d.), by appropriate methods which will be recorded (CO_2 preferred). Animals will not be fasted prior to necropsy.

Macroscopic examination

Detailed external and internal examination of all animals that are killed or die during the treatment period (including those that are replaced).

Organ weights

The following will be dissected free of fat and connective tissue and weighed whole, paired organs together (all animals).

Kidneys
Liver
Brain
Organs of interest (i.e. target organs/tissues)

Representative samples of the following, preserved in 10% buffered formol saline.

Terminal Serum Sample

Approximately 1 mL of serum will be collected from all animals at termination (both 48 and 168 hours) frozen at -70° C. for shipment to IC.

Microscopic examination

**Tissues/organs processed**	All those listed from all animals.
**Treatment of tissues**	Target organs processed through to paraffin wax, sectioned, stained with haematoxylin and eosin, and examined microscopically. Other tissues processed at discretion of company.<br><br>Livers and kidneys will removed and weighed a rapidly as possible and processed for shipment to IC immediately thereafter as described below.
**Kidneys**	Left kidney - TS sections (~ 4mm x 4mm) of cortex and papilla to be retained, snap frozen in liquid nitrogen and stored at -70°C, for examination by ^{1}H MAS NMR spectroscopy from all animals. Right kidney and remaining sample of left kidney, to be processed for histology as detailed above.
**Liver**	Samples of liver (left lateral lobe) to be snap frozen in liquid nitrogen (~ 4mm x 4mm) and stored at -70°C, for examination by ^{1}H MAS NMR spectroscopy from all animals. Remaining sample of liver to be processed for histology as detailed above.
**Other tissues of interest**	Samples (~ 4mm x 4mm) to be snap frozen in liquid nitrogen and stored at -70°C, for examination by ^{1}H MAS NMR spectroscopy from all animals. Remaining sample to be processed for histology as detailed above.<br><br>Further sections may be stained by additional methods at the discretion of the Study Pathologist.

Shipment Details

Suggested Shipping Containers:

Urine: Corning Cryogenic Vial, round bottom, freestanding, threaded stopper with silicone rubber washer and 4 mL capacity (part #: 430662)

Serum: Capped eppendorf tubes or equivalent.

Tissues: #2190 (2.0) mL) Cryo-Stor Vials, Perfector Scientific PO Box 91 Atascadero, CA 93423 805-366-8497

Dose Solutions: #2190 (2.0) mL) Cryo-Stor Vials, Perfector Scientific PO Box 91 Atascadero, CA 93423 805-366-8497

Note: Alternative containers are to be approved by IC prior to shipment

Shipping Procedure:

A sufficient quantity of bar code labels for all samples to be shipped will be requested from IC. Each company will maintain a cross-reference of bar code label and internal sample identification. A hardcopy of a spreadsheet containing all relevant sample identification information and results will be shipped with samples. Missing samples are to be clearly labelled on the spreadsheet. All urine, tissue and serum samples along with samples of dosing solutions, from any individual study should be shipped together. Prior to shipment, all three individuals listed as contacts below are to be informed of the availability of samples for shipment. Samples will not be shipped until approval is given by IC. Samples should be shipped in racks or other such devices that maintain the temporal order of collection.

Contacts:

Dr. Mary Bollard / Dr. Tim Ebbels & Nahid Ashby
Biological Chemistry,
Biomedical Sciences Division,
Imperial College of Science, Technology and Medicine,
University of London,
Sir Alexander Fleming Building,
South Kensington,
London, SW7 2AZ, UK.

Suggested Courier:

Biocair International
The business Centre
Church End
Cherry Hinton
Cambridge CB1 3LD
01223 245223
(biocair@dial.pipex.com)

David Rushall (USA/Europe)
Vanessa Webb / Dianne Brownlow (UK)

Monday – Wednesday

Supplementary Table S1. Number and proportion of missing values that were imputed for each clinical parameter from a total of 26546 urine samples and 6473 serum samples.

**Clinical parameter**	**Number of missing values (urine = 26546; serum = 6473)**	**Proportion of missing values (%)**
Urine Total Volume (mL)	740	2.79
Urine Osmolality (mOsm/L)	7921	29.84
Urine pH	1986	7.48
Urine Protein (g/L)	4693	17.68
Urine Glucose (mmol/L)	6150	23.17
Serum Creatinine (umol/L)	274	4.23
Serum Urea Nitrogen (umol/L)	291	4.5
Serum ALT(IU/L)	337	5.21
Serum AST (IU/L)	280	4.33
Serum Glucose (mmol/L)	1202	18.57
Serum Sodium (mmol/L)	736	11.37
Serum Potassium (mmol/L)	760	11.74
Serum Calcium (mmol/L)	361	5.58
Serum Phosphate (mmol/L)	397	6.13
Serum Albumin (g/L)	365	5.64
Serum Total Protein (g/L)	356	5.5
Serum Total Bilirubin (umol/L)	1131	17.47

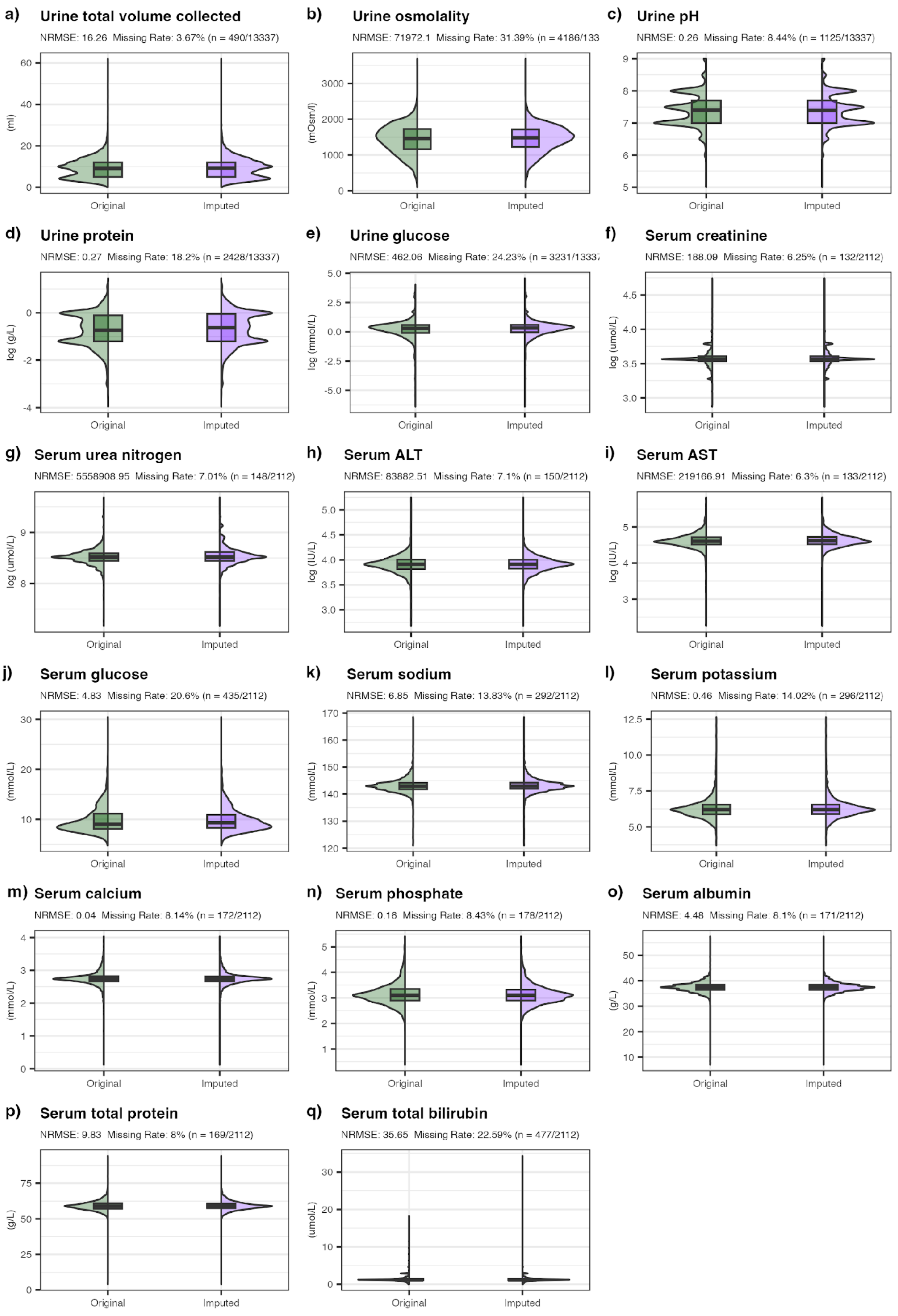

Figure S2. Half violin boxplots showing the distribution of the control samples pre-imputation (green) and post-imputation (purple) for each clinical parameter. The percentage and number of missing values, along with the Normalised Root Mean Square Error (NRMSE) value for each parameter is provided.

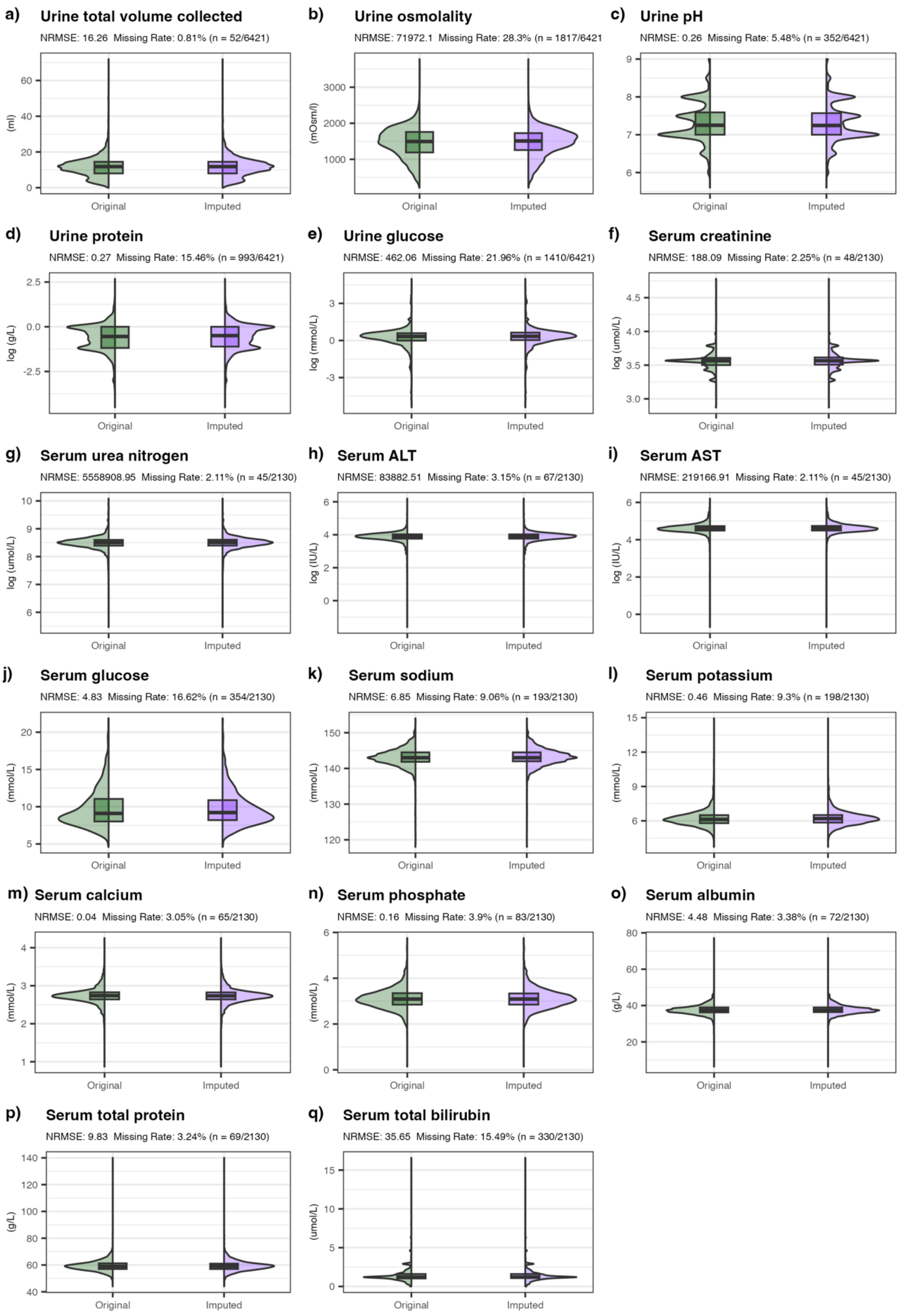

Figure S3. Half violin boxplots showing the distribution of the low dosed samples pre-imputation (green) and post-imputation (purple) for each clinical parameter. The percentage and number of missing values, along with the Normalised Root Mean Square Error (NRMSE) value for each parameter is provided.

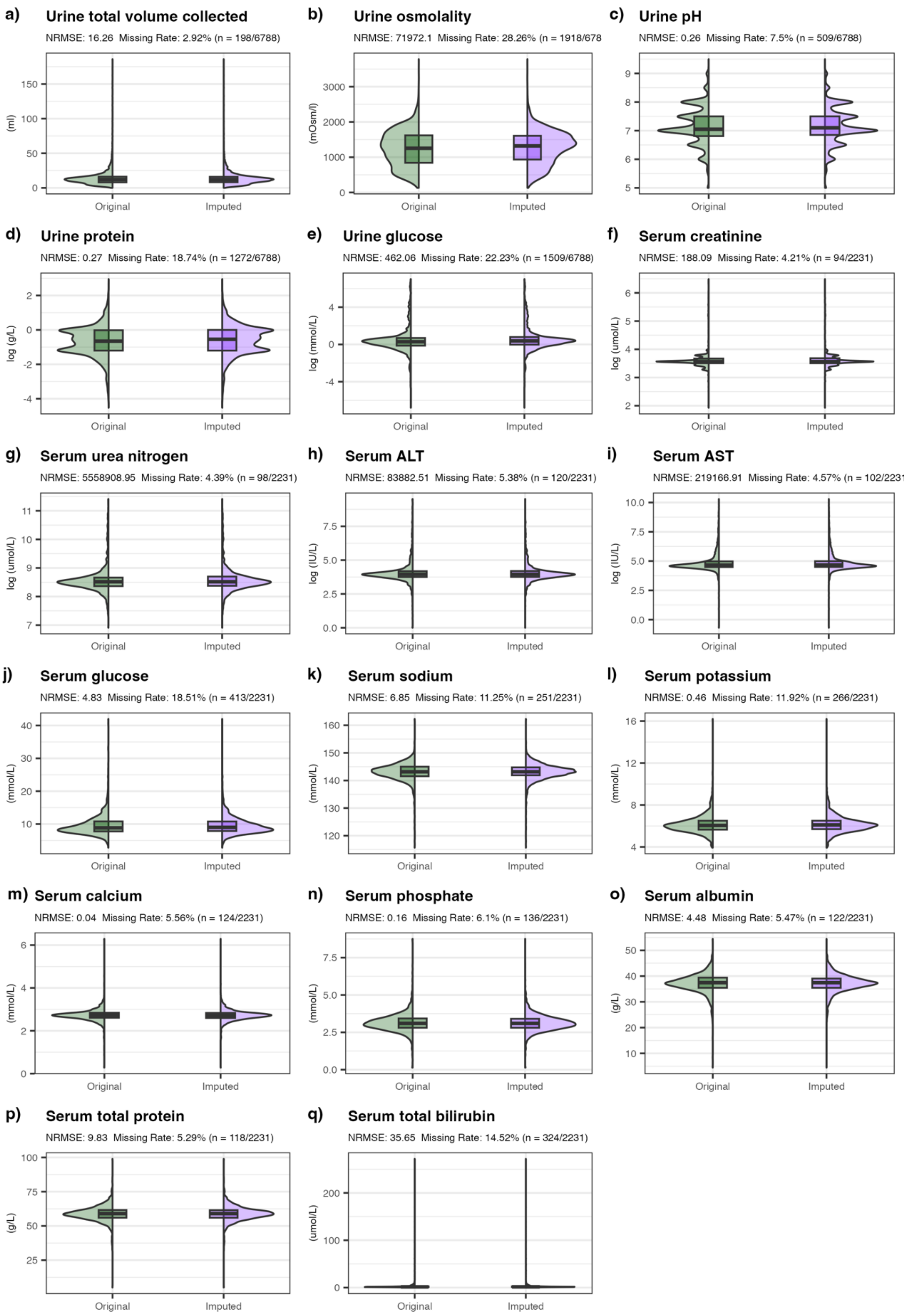

Figure S4. Half violin boxplots showing the distribution of the high dosed samples pre-imputation (green) and post-imputation (purple) for each clinical parameter. The percentage and number of missing values, along with the Normalised Root Mean Square Error (NRMSE) value for each parameter is provided.

Supplementary Table S2. Summary statistics for control rats for each clinical chemistry parameter.

**Parameter**	**Median (min - max)**	**Mean (± SD)**
Urine Total Volume Collected (mL)	9.4 (0 - 62)	9.36 (± 4.83)
Urine Osmolality (mOsm/L)	1487 (101 - 3684)	1456.41 (± 360.12)
Urine pH	7.39 (5 - 9)	7.39 (± 0.49)
Urine Protein (g/L)	0.54 (0 - 4.3)	0.64 (± 0.42)
Urine Glucose (mmol/L)	1.39 (0 - 96.79)	1.83 (± 3.1)
Serum Creatinine (umol/L)	35.36 (0 - 114.92)	35.88 (± 4.69)
Serum Urea Nitrogen (umol/L)	5000.47 (1430 - 16000)	5237.61 (± 1134.73)
Serum Alanine Aminotransferase (IU/L)	50 (14.5 - 190)	50.7 (± 9.23)
Serum Aspartate Aminotransferase (IU/L)	101 (9.5 - 336)	104.61 (± 21.79)
Serum Glucose (mmol/L)	9.36 (4.8 - 30.47)	9.89 (± 2.37)
Serum Sodium (mmol/L)	143 (121 - 168.55)	143.15 (± 2.58)
Serum Potassium (mmol/L)	6.2 (4.03 - 12.65)	6.3 (± 0.68)
Serum Calcium (mmol/L)	2.74 (0.12 - 4.04)	2.75 (± 0.21)
Serum Phosphate (mmol/L)	3.11 (0.38 - 5.41)	3.14 (± 0.42)
Serum Albumin (g/L)	37.45 (6.95 - 57.45)	37.43 (± 2.1)
Serum Total Protein (g/L)	59 (4 - 94.35)	59.14 (± 3.53)
Serum Total Bilirubin (umol/L)	1.2 (0 - 34.34)	1.42 (± 1.1)

Supplementary Table S3. Summary statistics for serum calcium (mmol/L) at 24 hrs, 48 hrs and 168 hrs post dose vehicle.

Target organ	Toxin	24 h post dose		48 h post dose		168 h post dose	
		**Median (min - max)**	**Mean (± SD)**	**Median (min - max)**	**Mean (± SD)**	**Median (min - max)**	**Mean (± SD)**
Liver	[E] 1,1-Dichloroethylene	2.76 (2.64 - 2.89)	2.76 (± 0.07)	2.72 (2.59 - 2.76)	2.7 (± 0.07)	2.72 (2.59 - 2.81)	2.72 (± 0.08)
Liver	[E] 1,2,3,4,5,6-hexachlorocyclohexane	2.73 (2.6 - 2.89)	2.74 (± 0.1)	2.81 (2.74 - 2.84)	2.79 (± 0.04)	2.74 (2.62 - 2.74)	2.71 (± 0.05)
Liver	[B] 1-Fluoropentane	2.75 (2.65 - 2.9)	2.75 (± 0.07)	2.7 (2.7 - 2.75)	2.72 (± 0.02)	2.75 (2.73 - 2.8)	2.76 (± 0.03)
Liver	[B] 2,4,6-Trihydroxyacetophenone (THA)	2.76 (2.69 - 2.81)	2.77 (± 0.04)	2.72 (2.64 - 2.76)	2.71 (± 0.05)	2.67 (2.57 - 2.74)	2.67 (± 0.06)
Liver	[B] 4-Amino-2,6-dichlorophenol (ADCP)	2.79 (2.72 - 2.81)	2.77 (± 0.04)	2.77 (2.73 - 2.78)	2.76 (± 0.02)	2.77 (2.74 - 2.79)	2.77 (± 0.02)
Liver	[C] Aflatoxin	2.76 (2.64 - 2.84)	2.74 (± 0.08)	2.87 (2.72 - 2.99)	2.85 (± 0.1)	2.68 (2.61 - 2.76)	2.67 (± 0.06)
Liver	[C] Allyl alcohol	2.75 (2.71 - 2.9)	2.78 (± 0.06)	2.67 (2.54 - 2.75)	2.64 (± 0.08)	2.59 (2.56 - 2.82)	2.64 (± 0.11)
Liver	[C] Allyl formate	2.72 (2.65 - 2.81)	2.73 (± 0.05)	2.74 (2.69 - 2.78)	2.74 (± 0.04)	2.81 (2.72 - 2.83)	2.78 (± 0.05)
Liver	[B] Azathioprine	2.79 (2.72 - 2.81)	2.77 (± 0.04)	2.77 (2.73 - 2.78)	2.76 (± 0.02)	2.77 (2.74 - 2.79)	2.77 (± 0.02)
Liver	[B] Bromobenzene	2.74 (2.72 - 2.84)	2.75 (± 0.03)	2.62 (2.59 - 2.69)	2.63 (± 0.04)	2.74 (2.69 - 2.84)	2.75 (± 0.06)
Liver	[C] Butylated hydroxytoluene	2.71 (2.51 - 2.93)	2.69 (± 0.14)	2.82 (2.74 - 3.22)	2.9 (± 0.19)	2.81 (2.45 - 2.93)	2.74 (± 0.18)
Liver	[D] Carbon tetrachloride	2.33 (2.06 - 2.67)	2.36 (± 0.24)	3.12 (3.08 - 3.22)	3.14 (± 0.06)	2.77 (2.66 - 2.85)	2.76 (± 0.07)
Liver	[C] Chlorpromazine	2.88 (2.01 - 3.19)	2.78 (± 0.39)	2.74 (2.56 - 2.86)	2.73 (± 0.11)	2.71 (2.61 - 2.74)	2.69 (± 0.05)
Liver	[B] Clofibrate	2.75 (2.67 - 2.84)	2.76 (± 0.06)	2.74 (2.64 - 2.79)	2.73 (± 0.07)	2.72 (2.62 - 2.81)	2.71 (± 0.07)
Liver	[B] Cyproterone acetate	2.84 (2.74 - 2.86)	2.82 (± 0.05)	2.67 (2.54 - 2.72)	2.65 (± 0.07)	2.72 (2.64 - 2.74)	2.7 (± 0.04)
Liver	[A] D-galactosamine	2.42 (2.17 - 2.74)	2.39 (± 0.18)	2.89 (2.77 - 2.97)	2.88 (± 0.08)	2.97 (2.74 - 3.32)	2.98 (± 0.24)
Liver	[B] Diethylhexylphthalate (DEHP)			2.74 (2.69 - 2.79)	2.74 (± 0.05)	2.74 (2.72 - 2.81)	2.76 (± 0.05)
Liver	[C] Dimethylformamide (DMF)	2.74 (2.68 - 2.87)	2.76 (± 0.06)	2.81 (2.74 - 2.85)	2.8 (± 0.04)	2.7 (2.56 - 2.81)	2.69 (± 0.09)
Liver	[C] Dimethylnitrosamine (DMN)	2.72 (2.63 - 2.84)	2.71 (± 0.06)	2.82 (2.68 - 2.9)	2.82 (± 0.09)	2.78 (2.69 - 2.98)	2.81 (± 0.12)
Liver	[A] Gadolinium chloride	2.52 (2.42 - 2.62)	2.52 (± 0.07)	2.96 (2.92 - 3.02)	2.96 (± 0.04)	2.96 (2.87 - 3.14)	2.97 (± 0.11)

Target organ	Toxin	24 h post dose		48 h post dose		168 h post dose	
		Median (min - max)	Mean (± SD)	Median (min - max)	Mean (± SD)	Median (min - max)	Mean (± SD)
Liver	[A, B, C, D, F] Hydrazine	2.67 (2.08 - 2.91)	2.64 (± 0.18)	2.75 (2.62 - 3.03)	2.79 (± 0.11)	2.79 (2.63 - 3.16)	2.81 (± 0.14)
Liver	[E] Hydrazine	2.69 (2.09 - 3.09)	2.7 (± 0.32)	2.69 (2.59 - 2.99)	2.71 (± 0.16)	2.99 (2.69 - 3.39)	3.03 (± 0.3)
Liver	[E] Indomethacin	2.72 (2.42 - 2.81)	2.69 (± 0.11)	2.86 (2.69 - 2.89)	2.83 (± 0.08)	2.74 (2.69 - 2.86)	2.75 (± 0.07)
Liver	[E] Ketoconazole			2.83 (2.75 - 2.9)	2.82 (± 0.06)	2.68 (2.6 - 2.73)	2.67 (± 0.05)
Liver	[C] Lead acetate	2.92 (2.63 - 3.4)	2.92 (± 0.22)	2.63 (2.6 - 2.71)	2.65 (± 0.05)	2.73 (2.68 - 2.75)	2.72 (± 0.03)
Liver	[A] Lipopolysaccharide (LPS)	2.58 (2.4 - 2.65)	2.56 (± 0.07)	3.2 (3.05 - 3.35)	3.2 (± 0.12)	3.13 (2.83 - 3.6)	3.15 (± 0.28)
Liver	[B] Methapyrilene	2.74 (2.64 - 2.79)	2.73 (± 0.05)	2.74 (2.69 - 2.79)	2.74 (± 0.04)	2.69 (2.62 - 2.74)	2.69 (± 0.05)
Liver	[E] Methylene dianiline	2.65 (2.5 - 2.73)	2.64 (± 0.07)	2.78 (2.75 - 2.88)	2.79 (± 0.05)	2.83 (2.78 - 2.93)	2.83 (± 0.06)
Liver	[C] Monocrotaline	2.7 (2.58 - 3.02)	2.72 (± 0.12)	2.78 (2.7 - 2.91)	2.79 (± 0.09)	2.82 (2.7 - 2.93)	2.8 (± 0.09)
Liver	[C] N-methylformamide (NMF)	2.78 (2.71 - 2.82)	2.77 (± 0.04)	2.69 (2.68 - 2.77)	2.72 (± 0.04)	2.68 (2.64 - 2.7)	2.68 (± 0.02)
Liver	[D] Phalloidin (chronic)			2.56 (2.54 - 2.67)	2.58 (± 0.05)	2.89 (2.81 - 2.94)	2.89 (± 0.05)
Liver	[E] Phenyl diisothiocyanate	2.74 (2.64 - 2.84)	2.74 (± 0.07)	2.74 (2.67 - 2.76)	2.73 (± 0.04)	2.62 (2.54 - 2.76)	2.63 (± 0.08)
Liver	[E] Phenyl isothiocyanate	2.65 (2.48 - 2.74)	2.64 (± 0.07)	2.76 (2.74 - 2.79)	2.76 (± 0.02)	2.74 (2.72 - 2.79)	2.74 (± 0.03)
Liver	[B] Retinyl palmitate	2.79 (2.72 - 2.81)	2.77 (± 0.04)	2.77 (2.73 - 2.78)	2.76 (± 0.02)	2.77 (2.74 - 2.79)	2.77 (± 0.02)
Liver	[B] Sodium Valproate			2.72 (2.69 - 2.77)	2.73 (± 0.03)	2.74 (2.69 - 2.84)	2.77 (± 0.07)
Liver	[C] a-Naphthylisothiocyanate (ANIT)	2.74 (2.53 - 2.84)	2.73 (± 0.1)	2.77 (2.71 - 2.79)	2.75 (± 0.04)	2.73 (2.68 - 2.84)	2.75 (± 0.07)
Kidney	[D] 2-Bromophenol	2.53 (2.4 - 2.57)	2.5 (± 0.05)	3.3 (2.92 - 3.72)	3.32 (± 0.34)	3.35 (3.12 - 3.55)	3.33 (± 0.17)
Kidney	[E] 3,5-Dichloroaniline hydrochloride	2.75 (2.62 - 2.84)	2.76 (± 0.06)	2.76 (2.72 - 2.79)	2.75 (± 0.03)	2.74 (2.54 - 2.76)	2.69 (± 0.09)
Kidney	[E] Atractyloside	2.34 (2.19 - 2.61)	2.35 (± 0.11)	2.94 (2.81 - 2.94)	2.9 (± 0.06)	2.81 (2.74 - 2.96)	2.82 (± 0.09)
Kidney	[D] Bromoethylamine hydrobromide	2.64 (2.58 - 2.73)	2.64 (± 0.05)	2.96 (2.75 - 2.99)	2.91 (± 0.1)	2.96 (2.78 - 3.08)	2.94 (± 0.12)

Target organ	Toxin	24 h post dose		48 h post dose		168 h post dose	
		Median (min - max)	Mean (± SD)	Median (min - max)	Mean (± SD)	Median (min - max)	Mean (± SD)
Kidney	[D] Cephaloridine	2.64 (2.52 - 2.85)	2.66 (± 0.09)	3.28 (2.74 - 3.32)	3.17 (± 0.25)	2.97 (2.72 - 3.19)	2.98 (± 0.17)
Kidney	[B] Chlorethanamine	2.78 (2.72 - 2.92)	2.8 (± 0.06)	2.72 (2.62 - 2.79)	2.7 (± 0.07)	2.74 (2.64 - 2.74)	2.71 (± 0.04)
Kidney	[A] Cisplatin	2.57 (2.43 - 2.73)	2.58 (± 0.09)	3.27 (2.9 - 3.41)	3.19 (± 0.19)	2.94 (2.75 - 3.13)	2.95 (± 0.14)
Kidney	[A] D-limonene (chronic)						
Kidney	[E] Dichlorophenyl succinimide	2.65 (2.5 - 2.73)	2.64 (± 0.07)	2.78 (2.75 - 2.88)	2.79 (± 0.05)	2.83 (2.78 - 2.93)	2.83 (± 0.06)
Kidney	[D] Ethylene glycol	2.52 (2.3 - 2.74)	2.54 (± 0.14)	2.97 (2.91 - 3.2)	3.04 (± 0.13)	2.89 (2.51 - 3)	2.82 (± 0.19)
Kidney	[A] Folic acid	2.3 (2.18 - 2.72)	2.38 (± 0.2)	2.81 (2.73 - 3.04)	2.85 (± 0.13)	2.78 (2.74 - 3.14)	2.84 (± 0.17)
Kidney	[A] Gentamicin	2.56 (2.43 - 2.68)	2.56 (± 0.07)	3.02 (2.74 - 3.18)	3 (± 0.16)	3.08 (2.72 - 3.37)	3.06 (± 0.23)
Kidney	[B] Maleic acid	2.79 (2.72 - 2.81)	2.77 (± 0.04)	2.77 (2.73 - 2.78)	2.76 (± 0.02)	2.77 (2.74 - 2.79)	2.77 (± 0.02)
Kidney	[A] N-phenylanthranilic acid (chronic)	2.63 (2.5 - 3.08)	2.7 (± 0.21)	2.73 (2.3 - 3.43)	2.8 (± 0.41)	2.65 (2.08 - 3.08)	2.67 (± 0.4)
Kidney	[D] Para-aminophenol	2.69 (2.49 - 2.74)	2.67 (± 0.07)	3.24 (2.89 - 3.37)	3.16 (± 0.21)	3.2 (2.91 - 3.24)	3.15 (± 0.14)
Kidney	[A] Puromycin			2.73 (2.66 - 2.78)	2.73 (± 0.05)	3.08 (2.68 - 3.51)	3.03 (± 0.34)
Kidney	[B] Vancomycin hydrochloride			2.74 (2.74 - 2.94)	2.8 (± 0.09)	2.74 (2.69 - 2.79)	2.74 (± 0.04)
Liver & Kidney	[E] Acetaminophen	2.63 (2.59 - 2.74)	2.65 (± 0.05)	2.84 (2.76 - 2.89)	2.82 (± 0.06)	2.79 (2.74 - 2.86)	2.79 (± 0.05)
Liver & Kidney	[B] Aurothiomalate	2.79 (2.72 - 2.81)	2.77 (± 0.04)	2.77 (2.73 - 2.78)	2.76 (± 0.02)	2.77 (2.74 - 2.79)	2.77 (± 0.02)
Liver & Kidney	[C] Chloroform	2.77 (2.51 - 3.24)	2.78 (± 0.2)	2.74 (2.68 - 2.81)	2.74 (± 0.05)	2.74 (2.63 - 2.92)	2.74 (± 0.11)
Liver & Kidney	[D] Cyclosporin	2.59 (2.43 - 2.71)	2.56 (± 0.09)	3.06 (2.84 - 3.16)	3.03 (± 0.13)	3.08 (2.78 - 3.33)	3.07 (± 0.2)
Liver & Kidney	[D] Dichlorobenzene	2.46 (2.17 - 2.59)	2.4 (± 0.14)	3.04 (3 - 3.3)	3.08 (± 0.13)	3.16 (2.89 - 3.21)	3.09 (± 0.15)
Liver & Kidney	[C] Ethionine	2.67 (1.15 - 2.83)	2.42 (± 0.56)	2.79 (2.7 - 2.83)	2.79 (± 0.05)	2.74 (2.66 - 2.76)	2.73 (± 0.04)
Liver & Kidney	[B] Hexachlorobutadiene (HCBD)			2.7 (2.68 - 2.8)	2.72 (± 0.05)	2.8 (2.73 - 2.88)	2.8 (± 0.06)

Target organ	Toxin	24 h post dose		48 h post dose		168 h post dose	
		**Median (min - max)**	**Mean (± SD)**	**Median (min - max)**	**Mean (± SD)**	**Median (min - max)**	**Mean (± SD)**
Liver & Kidney	[B] Mercuric chloride	2.79 (2.62 - 2.89)	2.79 (± 0.09)	2.74 (2.69 - 2.77)	2.73 (± 0.04)	2.74 (2.72 - 2.79)	2.75 (± 0.03)
Liver & Kidney	[E] Microcystin-LR			2.76 (2.72 - 2.84)	2.78 (± 0.06)	2.72 (2.64 - 2.74)	2.7 (± 0.05)
Liver & Kidney	[E] Rotenone	2.69 (2.64 - 2.81)	2.7 (± 0.06)	2.76 (2.74 - 2.84)	2.77 (± 0.04)	2.79 (2.76 - 2.81)	2.79 (± 0.02)
Liver & Kidney	[E] S-(1,2-dichlorovinyl)-cysteine (DCVC)	2.52 (2.17 - 2.67)	2.5 (± 0.14)	3.04 (2.99 - 3.14)	3.05 (± 0.06)	2.99 (2.81 - 3.04)	2.95 (± 0.09)
Liver & Kidney	[D] Thioacetamide	2.58 (2.24 - 2.65)	2.48 (± 0.17)	2.83 (2.75 - 2.94)	2.84 (± 0.07)	2.82 (2.68 - 2.84)	2.78 (± 0.07)
Pancreas	[E] 1-Cyano-2-hydroxy-3-butene	2.74 (2.35 - 2.83)	2.66 (± 0.17)	2.78 (2.65 - 2.83)	2.75 (± 0.08)	2.73 (2.73 - 2.8)	2.75 (± 0.03)
Pancreas	[C] Caerulin	2.74 (2.67 - 2.98)	2.77 (± 0.09)	2.9 (2.6 - 3.15)	2.86 (± 0.21)	2.71 (2.67 - 2.79)	2.72 (± 0.05)
Pancreas	[E] L-arginine	2.62 (2.43 - 2.83)	2.61 (± 0.12)	2.88 (2.73 - 2.93)	2.84 (± 0.08)	2.83 (2.75 - 2.85)	2.82 (± 0.04)
Pancreas	[B] Streptozotocin	2.72 (2.64 - 2.92)	2.73 (± 0.08)	2.77 (2.74 - 2.84)	2.78 (± 0.04)	2.79 (2.72 - 2.84)	2.78 (± 0.05)
Testicular	[D] 1,3-Dinitrobenzene	2.33 (2.17 - 2.61)	2.33 (± 0.12)	3.18 (3.08 - 3.41)	3.21 (± 0.13)	3.14 (2.74 - 3.18)	3.01 (± 0.2)
Testicular	[C] Cadmium chloride	2.72 (2.58 - 2.8)	2.71 (± 0.06)	3.03 (2.92 - 3.22)	3.07 (± 0.13)	2.9 (2.72 - 3.03)	2.87 (± 0.14)
Testicular	[D] Cadmium chloride	2.56 (2.37 - 2.68)	2.54 (± 0.1)	2.83 (2.8 - 3.05)	2.89 (± 0.11)	2.9 (2.88 - 2.97)	2.91 (± 0.04)
Testicular	[D] Carbendazim	2.58 (2.46 - 2.7)	2.58 (± 0.08)	3.06 (2.79 - 3.2)	3.03 (± 0.15)	2.96 (2.8 - 3.36)	3 (± 0.21)
Testicular	[D] Di-n-pentyl-phthalate	2.65 (2.58 - 2.7)	2.65 (± 0.04)	2.84 (2.8 - 3.15)	2.91 (± 0.14)	2.86 (2.78 - 2.89)	2.85 (± 0.04)
Testicular	[D] Ethane dimethane sulfonate (EDS)	2.71 (2.66 - 2.79)	2.71 (± 0.04)	2.79 (2.69 - 2.95)	2.79 (± 0.1)	3.47 (3.25 - 3.56)	3.41 (± 0.14)
Testicular	[D] Methoxyacetic acid	2.6 (2.54 - 2.71)	2.61 (± 0.05)	2.99 (2.94 - 3.28)	3.04 (± 0.14)	2.91 (2.77 - 3.22)	2.96 (± 0.18)
Multiple organ	[B] Adriamycin	2.74 (2.67 - 2.91)	2.76 (± 0.07)	2.76 (2.71 - 2.81)	2.76 (± 0.04)	2.74 (2.67 - 2.86)	2.75 (± 0.08)
Multiple organ	[C] Amphotericin B	2.74 (2.61 - 2.81)	2.73 (± 0.05)	2.78 (2.61 - 2.9)	2.78 (± 0.11)	2.71 (2.6 - 2.77)	2.7 (± 0.07)
Multiple organ	[C] Azaserine	2.76 (2.7 - 2.83)	2.76 (± 0.04)	2.75 (2.7 - 2.85)	2.75 (± 0.06)	2.61 (2.52 - 2.69)	2.6 (± 0.07)
Multiple organ	[A] Dexamethasone	2.58 (2.43 - 2.63)	2.56 (± 0.06)	2.92 (2.86 - 3.24)	2.99 (± 0.16)	3.89 (2.93 - 4.04)	3.62 (± 0.47)
Multiple organ	[E] Mitomycin-C	2.8 (2.62 - 2.94)	2.79 (± 0.11)	2.74 (2.67 - 2.81)	2.73 (± 0.06)	2.72 (2.59 - 2.74)	2.69 (± 0.06)

Target organ	Toxin	24 h post dose		48 h post dose		168 h post dose	
		Median (min - max)	Mean (± SD)	Median (min - max)	Mean (± SD)	Median (min - max)	Mean (± SD)
Physiological stressor	[C] 1,1-Dichloroethylene & maleic acid	2.85 (2.7 - 3.2)	2.86 (± 0.14)	2.72 (2.62 - 2.76)	2.71 (± 0.05)	2.72 (2.68 - 2.78)	2.72 (± 0.04)
Physiological stressor	[C] 2,4-Dinitrophenol	2.67 (2.54 - 2.83)	2.69 (± 0.09)	2.75 (2.67 - 2.79)	2.74 (± 0.05)	2.82 (2.59 - 2.84)	2.76 (± 0.1)
Physiological stressor	[B] 4-Pentenoic acid	2.76 (2.69 - 2.84)	2.76 (± 0.05)	2.67 (2.54 - 2.74)	2.66 (± 0.08)	2.76 (2.57 - 2.76)	2.71 (± 0.09)
Physiological stressor	[D] Acetazolamide	2.61 (2.5 - 2.74)	2.61 (± 0.09)	2.91 (2.74 - 3.41)	3 (± 0.26)	3.06 (2.82 - 3.32)	3.06 (± 0.18)
Physiological stressor	[C] Acivicin	2.78 (2.63 - 3.03)	2.77 (± 0.12)	2.75 (2.63 - 2.93)	2.77 (± 0.11)	2.67 (2.56 - 2.79)	2.67 (± 0.09)
Physiological stressor	[E] Ammonium chloride			2.74 (2.72 - 2.81)	2.74 (± 0.04)	2.74 (2.67 - 2.76)	2.74 (± 0.04)
Physiological stressor	[D] Carboplatin	2.44 (2.29 - 2.56)	2.44 (± 0.08)	3.02 (2.92 - 3.36)	3.07 (± 0.17)	3.11 (2.98 - 3.23)	3.11 (± 0.1)
Physiological stressor	[A] Choline and choline/methionine deficiency (chronic)	2.64 (2.37 - 2.99)	2.65 (± 0.2)	2.94 (2.74 - 3.04)	2.88 (± 0.13)	2.82 (2.62 - 2.92)	2.79 (± 0.13)
Physiological stressor	[B] Food restriction (chronic)	2.74 (2.67 - 2.84)	2.74 (± 0.07)	2.83 (2.69 - 2.86)	2.8 (± 0.08)	2.74 (2.74 - 2.86)	2.77 (± 0.05)
Physiological stressor	[D] Furosemide	2.55 (2.5 - 2.64)	2.56 (± 0.05)	3.02 (2.85 - 3.27)	3.05 (± 0.15)	3.16 (3.04 - 3.4)	3.22 (± 0.16)
Physiological stressor	[B] Insulin	2.74 (2.69 - 2.79)	2.75 (± 0.03)	2.79 (2.74 - 2.84)	2.8 (± 0.04)	2.67 (2.62 - 2.74)	2.68 (± 0.05)
Physiological stressor	[E] Methotrexate	2.76 (0.12 - 2.81)	2.5 (± 0.84)	2.69 (2.64 - 2.79)	2.7 (± 0.06)	2.74 (2.74 - 2.79)	2.75 (± 0.02)
Physiological stressor	[A] Partial hepatectomy	2.7 (2.62 - 2.79)	2.71 (± 0.06)	2.81 (2.64 - 3.06)	2.82 (± 0.15)	2.79 (2.72 - 2.89)	2.78 (± 0.07)
Physiological stressor	[A] Phenobarbital (chronic)	2.52 (2.4 - 2.67)	2.53 (± 0.09)	2.99 (2.82 - 3.01)	2.96 (± 0.08)	3.07 (2.89 - 3.51)	3.17 (± 0.29)
Physiological stressor	[A] Pregnenolone 16 alpha carbonitrile (chronic)						
Physiological stressor	[A] Probenecid	2.56 (2.49 - 2.66)	2.56 (± 0.06)	3.11 (2.82 - 3.21)	3.05 (± 0.15)	2.94 (2.84 - 2.96)	2.91 (± 0.05)
Physiological stressor	[C] Rosiglitazone	2.82 (2.66 - 3.03)	2.82 (± 0.12)	2.72 (2.7 - 2.78)	2.72 (± 0.03)	2.74 (2.63 - 2.86)	2.72 (± 0.09)
Physiological stressor	[C] Rosiglitazone (chronic)	2.74 (2.52 - 2.85)	2.71 (± 0.09)				
Physiological stressor	[E] Sodium bicarbonate	2.61 (2.54 - 2.67)	2.61 (± 0.04)				
Physiological stressor	[A] Unilateral nephrectomy	2.69 (2.6 - 2.8)	2.68 (± 0.06)	2.93 (2.8 - 3.28)	2.97 (± 0.19)	2.75 (2.73 - 2.83)	2.78 (± 0.05)

Target organ	Toxin	24 h post dose		48 h post dose		168 h post dose	
		Median (min - max)	Mean (± SD)	Median (min - max)	Mean (± SD)	Median (min - max)	Mean (± SD)
Physiological stressor	[B] Water deprivation (chronic)	2.76 (2.57 - 2.84)	2.74 (± 0.08)	2.7 (2.57 - 2.74)	2.68 (± 0.07)	2.75 (2.59 - 2.86)	2.74 (± 0.09)
No Effect	[E] Acetaminophen (chronic)	2.75 (2.73 - 2.8)	2.77 (± 0.03)			2.7 (2.65 - 2.8)	2.71 (± 0.06)
No Effect	[C] Buthionine sulphoxime	2.76 (2.66 - 3.02)	2.8 (± 0.13)	2.81 (2.66 - 2.83)	2.76 (± 0.07)	2.69 (2.54 - 2.83)	2.69 (± 0.1)
No Effect	[C] Ferrous sulphate	2.71 (2.55 - 2.83)	2.7 (± 0.09)	2.74 (2.64 - 2.8)	2.72 (± 0.06)	2.77 (2.67 - 2.79)	2.75 (± 0.05)
No Effect	[B] Ifosfamide	2.78 (2.58 - 2.88)	2.77 (± 0.08)	2.68 (2.55 - 2.7)	2.65 (± 0.06)	2.73 (2.58 - 2.78)	2.71 (± 0.08)
No Effect	[B] Lithocholic acid	2.76 (2.68 - 2.85)	2.76 (± 0.06)	2.73 (2.55 - 2.78)	2.69 (± 0.09)	2.68 (2.68 - 2.8)	2.72 (± 0.06)
No Effect	[E] Paraquat			2.81 (2.74 - 3.06)	2.84 (± 0.13)	2.69 (2.67 - 2.74)	2.7 (± 0.03)
No Effect	[D] Potassium dichromate	2.04 (1.76 - 2.62)	2.06 (± 0.23)	3.18 (2.74 - 3.48)	3.17 (± 0.3)	3.28 (2.79 - 3.37)	3.18 (± 0.24)
No Effect	[C] Trichlorethylene	2.66 (2.57 - 2.82)	2.68 (± 0.08)	2.79 (2.74 - 2.87)	2.81 (± 0.06)	2.79 (2.7 - 2.89)	2.78 (± 0.08)

A-F: Indicates Pharmaceutical Company & sample origin

Supplementary Table S4. Summary statistics for serum sodium (mmol/L) at 24 hrs, 48 hrs and 168 hrs post dose vehicle.

Target organ	Toxin	24 h post dose		48 h post dose		168 h post dose	
		Median (min - max)	Mean (± SD)	Median (min - max)	Mean (± SD)	Median (min - max)	Mean (± SD)
Liver	[E] 1,1-Dichloroethylene	143.84 (141.75 - 152)	144.45 (± 2.78)	143 (141 - 144)	142.8 (± 1.1)	143 (142 - 144)	143.2 (± 0.84)
Liver	[E] 1,2,3,4,5,6-hexachlorocyclohexane	144.46 (142.94 - 146.14)	144.52 (± 0.98)	144 (144 - 144)	144 (± 0)	142 (140 - 142)	141.4 (± 0.89)
Liver	[B] 1-Fluoropentane	143 (141 - 146)	143.2 (± 1.75)	143 (142 - 145)	143.4 (± 1.14)	141 (141 - 144)	141.8 (± 1.3)
Liver	[B] 2,4,6-Trihydroxyacetophenone (THA)	143.5 (142 - 146)	143.8 (± 1.48)	143 (143 - 144)	143.4 (± 0.55)	142 (141 - 143)	142 (± 0.71)
Liver	[B] 4-Amino-2,6-dichlorophenol (ADCP)	142.56 (142.17 - 144.33)	142.89 (± 0.81)	142.3 (142.25 - 143.05)	142.59 (± 0.42)	142.07 (141.52 - 142.22)	141.93 (± 0.34)
Liver	[C] Aflatoxin	143.2 (140.45 - 145.55)	143.38 (± 1.5)	141.85 (141.15 - 144.35)	142.43 (± 1.3)	141.55 (139.55 - 145.55)	142.09 (± 2.19)

Target organ	Toxin	24 h post dose		48 h post dose		168 h post dose	
		Median (min - max)	Mean (± SD)	Median (min - max)	Mean (± SD)	Median (min - max)	Mean (± SD)
Liver	[C] Allyl alcohol	142.15 (139.7 - 145.2)	142.05 (± 1.68)	143.1 (141.5 - 146.7)	143.9 (± 2.07)	144.8 (142.3 - 145.6)	144.16 (± 1.36)
Liver	[C] Allyl formate	142.75 (140.3 - 144.7)	142.6 (± 1.5)	142.3 (140.5 - 143.1)	142.2 (± 1.07)	144.1 (142.9 - 145.5)	144.3 (± 1.1)
Liver	[B] Azathioprine	142.56 (142.17 - 144.33)	142.89 (± 0.81)	142.3 (142.25 - 143.05)	142.59 (± 0.42)	142.07 (141.52 - 142.22)	141.93 (± 0.34)
Liver	[B] Bromobenzene	143 (141 - 146)	143.3 (± 1.49)	140 (139 - 142)	140.4 (± 1.52)	145 (142 - 146)	144.6 (± 1.67)
Liver	[C] Butylated hydroxytoluene	143.35 (141.55 - 145.45)	143.64 (± 1.38)	142.15 (140.35 - 142.75)	141.73 (± 1)	143.35 (140.35 - 144.05)	142.47 (± 1.69)
Liver	[D] Carbon tetrachloride	144 (140.6 - 149)	143.98 (± 2.44)	141 (140 - 143)	141.4 (± 1.14)	143 (139 - 149)	143 (± 3.94)
Liver	[C] Chlorpromazine	143.1 (140.95 - 144.75)	142.95 (± 1.29)	144.25 (142.65 - 145.75)	144.09 (± 1.23)	142.15 (140.85 - 143.95)	142.19 (± 1.27)
Liver	[B] Clofibrate	142.5 (141 - 145)	143 (± 1.41)	144 (143 - 144)	143.6 (± 0.55)	143 (142 - 144)	143.2 (± 0.84)
Liver	[B] Cyproterone acetate	143.5 (142 - 144)	143.4 (± 0.7)	143 (141 - 144)	142.6 (± 1.14)	142 (141 - 143)	141.8 (± 0.84)
Liver	[A] D-galactosamine	140.9 (136.35 - 144.25)	140.73 (± 2.9)	146.85 (145.65 - 148.55)	146.91 (± 1.04)	141.75 (126.95 - 147.15)	138.17 (± 8.5)
Liver	[B] Diethylhexylphthalate (DEHP)			143 (140 - 145)	143 (± 1.87)	142 (142 - 143)	142.4 (± 0.55)
Liver	[C] Dimethylformamide (DMF)	141.75 (139.75 - 143.75)	141.93 (± 1.34)	142.95 (141.45 - 143.25)	142.63 (± 0.72)	144.55 (144.15 - 146.85)	145.31 (± 1.42)
Liver	[C] Dimethylnitrosamine (DMN)	140.85 (139.35 - 144.35)	141.32 (± 1.65)	143.15 (142.55 - 144.35)	143.43 (± 0.75)	143.55 (142.95 - 145.55)	144.15 (± 1.13)
Liver	[A] Gadolinium chloride	142.15 (140.5 - 145.9)	142.35 (± 1.45)	143.3 (141.4 - 144.2)	142.98 (± 1.03)	145.7 (143.9 - 147.8)	146 (± 1.54)
Liver	[A, B, C, D, F] Hydrazine	143 (140.25 - 149.85)	143.53 (± 2.48)	142.8 (139.05 - 146.5)	142.74 (± 2.04)	143 (137 - 150)	143.07 (± 2.85)
Liver	[E] Hydrazine	143 (143 - 145)	143.5 (± 0.85)	145 (142 - 145)	144.2 (± 1.3)	143 (140 - 144)	142.6 (± 1.52)
Liver	[E] Indomethacin	144.69 (141.94 - 146.64)	144.36 (± 1.65)	142 (138 - 144)	141.6 (± 2.3)	143 (142 - 144)	142.8 (± 0.84)
Liver	[E] Ketoconazole			143 (142 - 144)	143.2 (± 0.84)	142 (142 - 145)	143 (± 1.41)
Liver	[C] Lead acetate	142.55 (140.95 - 145.25)	142.71 (± 1.36)	141.15 (139.15 - 145.25)	141.83 (± 2.4)	146.45 (143.65 - 147.35)	145.71 (± 1.78)
Liver	[A] Lipopolysaccharide (LPS)	141.4 (139.6 - 143.7)	141.51 (± 1.18)	145.5 (143.3 - 147.1)	145.16 (± 1.52)	145.9 (142.7 - 148.7)	146.08 (± 2.27)

Target organ	Toxin	24 h post dose		48 h post dose		168 h post dose	
		Median (min - max)	Mean (± SD)	Median (min - max)	Mean (± SD)	Median (min - max)	Mean (± SD)
Liver	[B] Methapyrilene	141 (140 - 144)	141.5 (± 1.51)	145 (142 - 146)	144.4 (± 1.52)	144 (142 - 145)	144 (± 1.22)
Liver	[E] Methylene dianiline	144.52 (141.12 - 145.61)	143.97 (± 1.56)	143 (142 - 144)	143 (± 1)	143 (141 - 143)	142.4 (± 0.89)
Liver	[C] Monocrotaline	142.35 (139.45 - 143.55)	142.19 (± 1.23)	145.85 (142.95 - 147.15)	145.35 (± 1.64)	143.05 (141.15 - 144.55)	143.15 (± 1.33)
Liver	[C] N-methylformamide (NMF)	141.7 (139.8 - 144.4)	141.98 (± 1.55)	143.3 (141.9 - 144.2)	143.16 (± 0.87)	143.2 (140.9 - 144)	142.96 (± 1.2)
Liver	[D] Phalloidin (chronic)			141.05 (140.25 - 142.85)	141.41 (± 1.1)	148.25 (143.15 - 151.55)	147.13 (± 3.39)
Liver	[E] Phenyl diisothiocyanate	144.64 (143.51 - 145.59)	144.43 (± 0.74)	142.5 (141.5 - 145.5)	142.9 (± 1.67)	143.5 (142.5 - 143.5)	143.1 (± 0.55)
Liver	[E] Phenyl isothiocyanate	143.14 (142.35 - 145.14)	143.34 (± 0.89)	143 (141 - 144)	142.6 (± 1.14)	143 (142 - 144)	143.2 (± 0.84)
Liver	[B] Retinyl palmitate	142.56 (142.17 - 144.33)	142.89 (± 0.81)	142.3 (142.25 - 143.05)	142.59 (± 0.42)	142.07 (141.52 - 142.22)	141.93 (± 0.34)
Liver	[B] Sodium Valproate			142.5 (140.5 - 144.5)	142.5 (± 1.41)	143.5 (142.5 - 145.5)	143.9 (± 1.14)
Liver	[C] a-Naphthylisothiocyanate (ANIT)	143.7 (142.9 - 145.3)	143.92 (± 0.78)	142.2 (140.3 - 143.1)	142 (± 1.05)	142.2 (139.4 - 142.8)	141.46 (± 1.42)
Kidney	[D] 2-Bromophenol	141 (140 - 143)	141.4 (± 0.84)	144 (143 - 145)	144.2 (± 0.84)	146 (143 - 147)	145.6 (± 1.67)
Kidney	[E] 3,5-Dichloroaniline hydrochloride	144.77 (142.12 - 146.49)	144.44 (± 1.73)	144.5 (143.5 - 147.5)	144.7 (± 1.64)	141.5 (141.5 - 142.5)	141.9 (± 0.55)
Kidney	[E] Atractyloside	144.48 (142.39 - 145.24)	144.23 (± 0.88)	142.5 (142.5 - 144.5)	143.1 (± 0.89)	143.5 (142.5 - 143.5)	143.1 (± 0.55)
Kidney	[D] Bromoethylamine hydrobromide	143.5 (142 - 152)	145.7 (± 4)	143 (139 - 144)	142.4 (± 2.07)	143 (140 - 145)	143.12 (± 1.96)
Kidney	[D] Cephaloridine	141 (141 - 143)	141.6 (± 0.84)	147 (143.42 - 150)	147.28 (± 2.52)	147 (144 - 148)	146.6 (± 1.67)
Kidney	[B] Chlorethanamine	143 (141 - 146)	143.4 (± 1.26)	141 (140 - 142)	141 (± 0.71)	142 (141 - 143)	142.2 (± 0.84)
Kidney	[A] Cisplatin	143 (139.5 - 149.5)	143.1 (± 2.8)	144.5 (143.5 - 145.5)	144.5 (± 0.71)	139.5 (139.5 - 140.5)	139.9 (± 0.55)
Kidney	[A] D-limonene (chronic)						
Kidney	[E] Dichlorophenyl succinimide	144.52 (141.12 - 145.61)	143.97 (± 1.56)	143 (142 - 144)	143 (± 1)	143 (141 - 143)	142.4 (± 0.89)
Kidney	[D] Ethylene glycol	140.5 (138.5 - 143.5)	140.8 (± 1.49)	144.5 (142.5 - 144.5)	143.9 (± 0.89)	145.5 (143.5 - 148.5)	145.7 (± 1.79)

Target organ	Toxin	24 h post dose		48 h post dose		168 h post dose	
		Median (min - max)	Mean (± SD)	Median (min - max)	Mean (± SD)	Median (min - max)	Mean (± SD)
Kidney	[A] Folic acid	141 (138 - 144.11)	141.49 (± 2.31)	144 (140 - 144.29)	143.06 (± 1.78)	146 (143 - 149)	146.4 (± 2.61)
Kidney	[A] Gentamicin	139 (137 - 141.62)	139.26 (± 1.5)	146 (143 - 148)	145.6 (± 2.07)	146 (146 - 147)	146.4 (± 0.55)
Kidney	[B] Maleic acid	142.56 (142.17 - 144.33)	142.89 (± 0.81)	142.3 (142.25 - 143.05)	142.59 (± 0.42)	142.07 (141.52 - 142.22)	141.93 (± 0.34)
Kidney	[A] N-phenylanthranilic acid (chronic)	137.75 (135.3 - 144.41)	138.52 (± 2.97)	144.7 (141.8 - 145.5)	144.2 (± 1.42)	145.9 (144.4 - 146.5)	145.74 (± 0.81)
Kidney	[D] Para-aminophenol	141 (139 - 142)	140.9 (± 1.2)	147 (144 - 151)	147.4 (± 2.7)	146 (145 - 147)	146 (± 0.71)
Kidney	[A] Puromycin			141.5 (140.5 - 143.5)	141.9 (± 1.14)	143.5 (142.5 - 146.5)	144.3 (± 1.64)
Kidney	[B] Vancomycin hydrochloride			143.5 (141.5 - 145.5)	143.3 (± 1.48)	142.5 (140.5 - 144.5)	142.5 (± 1.58)
Liver & Kidney	[E] Acetaminophen	143.68 (141.85 - 144.96)	143.69 (± 0.96)	142 (141 - 144)	142.2 (± 1.3)	144 (143 - 145)	143.8 (± 0.84)
Liver & Kidney	[B] Aurothiomalate	142.56 (142.17 - 144.33)	142.89 (± 0.81)	142.3 (142.25 - 143.05)	142.59 (± 0.42)	142.07 (141.52 - 142.22)	141.93 (± 0.34)
Liver & Kidney	[C] Chloroform	143.05 (142 - 143.8)	143.02 (± 0.68)	144.7 (143.2 - 145)	144.42 (± 0.72)	141.9 (140.3 - 142.6)	141.72 (± 0.85)
Liver & Kidney	[D] Cyclosporin	141.5 (140.5 - 143.5)	141.7 (± 1.03)	144.5 (144.5 - 145.5)	144.9 (± 0.55)	146.5 (142.5 - 147.5)	145.7 (± 2.17)
Liver & Kidney	[D] Dichlorobenzene	142 (136 - 144)	140.1 (± 3.18)	144 (143 - 147)	144.6 (± 1.52)	143 (142 - 145)	143.6 (± 1.34)
Liver & Kidney	[C] Ethionine	144.1 (140.75 - 148.15)	143.91 (± 2.14)	141.65 (138.95 - 145.25)	141.95 (± 2.24)	142.85 (141.55 - 144.15)	142.69 (± 1.1)
Liver & Kidney	[B] Hexachlorobutadiene (HCBD)			142 (142 - 144)	142.8 (± 1.1)	143 (141 - 144)	143 (± 1.22)
Liver & Kidney	[B] Mercuric chloride	144 (142 - 145)	143.5 (± 1.08)	141 (139 - 143)	141.2 (± 1.79)	142 (142 - 144)	142.6 (± 0.89)
Liver & Kidney	[E] Microcystin-LR			143 (143 - 145)	143.6 (± 0.89)	143 (142 - 143)	142.6 (± 0.55)
Liver & Kidney	[E] Rotenone	143.74 (142.1 - 146.53)	144.05 (± 1.49)	143 (141 - 143)	142.4 (± 0.89)	143 (141 - 144)	142.8 (± 1.1)
Liver & Kidney	[E] S-(1,2-dichlorovinyl)-cysteine (DCVC)	142.92 (142.46 - 144.14)	143.12 (± 0.53)	144 (143 - 145)	143.8 (± 0.84)	142 (141 - 144)	142.2 (± 1.3)
Liver & Kidney	[D] Thioacetamide	140 (121 - 141)	137.9 (± 5.97)	148 (145 - 149)	147.4 (± 1.52)	147 (146 - 149)	147.4 (± 1.52)
Pancreas	[E] 1-Cyano-2-hydroxy-3-butene	144.87 (141.48 - 145.82)	144.42 (± 1.31)	143 (141 - 143)	142.4 (± 0.89)	144 (143 - 145)	144 (± 0.71)

Target organ	Toxin	24 h post dose		48 h post dose		168 h post dose	
		Median (min - max)	Mean (± SD)	Median (min - max)	Mean (± SD)	Median (min - max)	Mean (± SD)
Pancreas	[C] Caerulin	140.65 (139.55 - 143.05)	141 (± 1.14)	146.45 (143.95 - 148.15)	145.89 (± 1.76)	144.25 (142.95 - 146.15)	144.23 (± 1.2)
Pancreas	[E] L-arginine	143.66 (139.69 - 144.54)	143.1 (± 1.48)	143 (142 - 144)	143 (± 1)	143 (142 - 144)	143 (± 1)
Pancreas	[B] Streptozotocin	143.5 (141 - 146)	143.7 (± 2.26)	144 (143 - 144)	143.6 (± 0.55)	142 (141 - 145)	142.6 (± 1.52)
Testicular	[D] 1,3-Dinitrobenzene	140.5 (139 - 142)	140.49 (± 1.06)	144 (143 - 145)	144 (± 0.71)	148 (148 - 150)	148.6 (± 0.89)
Testicular	[C] Cadmium chloride	140.95 (135.75 - 147.85)	141.56 (± 3.75)	151.65 (142.05 - 157.65)	151.43 (± 5.81)	143.25 (137.75 - 144.85)	142.49 (± 2.75)
Testicular	[D] Cadmium chloride	141.95 (140.4 - 143.3)	141.88 (± 0.95)	144.6 (142.2 - 146.4)	144.6 (± 1.67)	145.4 (143.3 - 145.9)	145 (± 1)
Testicular	[D] Carbendazim	142 (141 - 145)	142.4 (± 1.35)	145 (141 - 146)	143.8 (± 2.17)	144 (143 - 146)	144.2 (± 1.3)
Testicular	[D] Di-n-pentyl-phthalate	140.8 (139.7 - 142.3)	140.94 (± 0.78)	145.1 (143.7 - 146.4)	145.14 (± 0.99)	147.2 (146.5 - 154.3)	148.9 (± 3.2)
Testicular	[D] Ethane dimethane sulfonate (EDS)	141.5 (139 - 143)	141.3 (± 1.34)	149 (143.38 - 155)	149.08 (± 4.17)	143 (143 - 145)	143.6 (± 0.89)
Testicular	[D] Methoxyacetic acid	140.6 (139.75 - 142.75)	140.77 (± 0.88)	146.55 (145.15 - 148.15)	146.65 (± 1.16)	147.15 (143.25 - 149.95)	146.47 (± 2.6)
Multiple organ	[B] Adriamycin	143 (142 - 144)	142.8 (± 0.79)	143 (143 - 145)	143.6 (± 0.89)	143 (142 - 144)	142.8 (± 0.84)
Multiple organ	[C] Amphotericin B	143.75 (142.65 - 145.15)	143.86 (± 0.7)	142.15 (140.55 - 144.05)	142.25 (± 1.29)	141.15 (139.25 - 142.45)	141.07 (± 1.2)
Multiple organ	[C] Azaserine	142.9 (141.65 - 144.35)	142.94 (± 0.85)	141.75 (139.95 - 143.45)	141.85 (± 1.44)	144.35 (142.45 - 145.45)	144.01 (± 1.23)
Multiple organ	[A] Dexamethasone	141 (139.5 - 141.5)	140.8 (± 0.82)	146.5 (144.5 - 147.5)	146.3 (± 1.3)	151.5 (146.5 - 152.5)	150.7 (± 2.39)
Multiple organ	[E] Mitomycin-C	144.43 (143.29 - 147.17)	144.63 (± 1.24)	144 (141 - 144)	143.2 (± 1.3)	142 (141 - 143)	142.2 (± 0.84)
Physiological stressor	[C] 1,1-Dichloroethylene & maleic acid	143 (142 - 148.9)	144.11 (± 2.15)	143.3 (143.1 - 144.9)	143.76 (± 0.77)	141 (140 - 142.2)	141.2 (± 0.98)
Physiological stressor	[C] 2,4-Dinitrophenol	142 (140.2 - 144.7)	142.21 (± 1.32)	143.4 (140.4 - 145.5)	143.24 (± 1.84)	146.7 (142.9 - 148.7)	146.28 (± 2.11)
Physiological stressor	[B] 4-Pentenoic acid	144 (142 - 145)	144 (± 0.94)	140 (138 - 140)	139.4 (± 0.89)	143 (142 - 144)	142.8 (± 0.84)
Physiological stressor	[D] Acetazolamide	143.5 (139.96 - 146.5)	143.75 (± 2.11)	143.8 (139.5 - 146.5)	143.64 (± 2.61)	140.5 (138.5 - 142.5)	140.5 (± 1.58)
Physiological stressor	[C] Acivicin	144.25 (140.65 - 146.35)	143.73 (± 1.89)	142.85 (141.05 - 143.25)	142.41 (± 0.97)	141.85 (140.95 - 143.25)	141.97 (± 0.88)
Physiological stressor	[E] Ammonium chloride			143 (143 - 145)	143.4 (± 0.89)	144 (142 - 144)	143.4 (± 0.89)

Target organ	Toxin	24 h post dose		48 h post dose		168 h post dose	
		Median (min - max)	Mean (± SD)	Median (min - max)	Mean (± SD)	Median (min - max)	Mean (± SD)
Physiological stressor	[D] Carboplatin	140.5 (139 - 147)	140.9 (± 2.28)	145 (143 - 146)	144.6 (± 1.14)	145 (143 - 146)	144.6 (± 1.14)
Physiological stressor	[A] Choline and choline/methionine deficiency (chronic)	130.5 (121 - 143)	132.21 (± 9)	146 (143 - 148)	145.2 (± 2.17)	144 (142 - 147)	144.4 (± 2.07)
Physiological stressor	[B] Food restriction (chronic)	142.5 (139.5 - 150.5)	143.28 (± 3.07)	144 (141.5 - 144.5)	143.5 (± 1.41)	142.5 (141.5 - 144.5)	142.9 (± 1.14)
Physiological stressor	[D] Furosemide	142.5 (140.5 - 143.5)	142 (± 0.97)	145.5 (138.5 - 146.5)	144.1 (± 3.21)	148.5 (146.5 - 149.5)	148.1 (± 1.14)
Physiological stressor	[B] Insulin	142.5 (141 - 144)	142.6 (± 0.97)	146 (145 - 147)	146.2 (± 0.84)	143 (142 - 144)	142.8 (± 0.84)
Physiological stressor	[E] Methotrexate	143.48 (143.05 - 145.86)	143.87 (± 0.96)	144 (143 - 146)	144.4 (± 1.14)	143 (142 - 143)	142.6 (± 0.55)
Physiological stressor	[A] Partial hepatectomy	142.05 (140 - 144.2)	142.12 (± 1.29)	144.18 (141.3 - 145.9)	143.58 (± 1.83)	144 (143 - 145.7)	144.06 (± 1.03)
Physiological stressor	[A] Phenobarbital (chronic)	142 (141 - 143)	142.1 (± 0.57)	148 (146 - 149)	147.6 (± 1.14)	146 (143 - 149)	145.8 (± 2.39)
Physiological stressor	[A] Pregnenolone 16 alpha carbonitrile (chronic)						
Physiological stressor	[A] Probenecid	141.9 (139.7 - 143.1)	141.7 (± 1.18)	146.2 (141.1 - 147.5)	145.14 (± 2.5)	146.6 (144.3 - 148.1)	146.48 (± 1.38)
Physiological stressor	[C] Rosiglitazone	142.65 (139.45 - 145.95)	142.75 (± 1.82)	143.15 (142.75 - 143.65)	143.13 (± 0.36)	143.45 (139.35 - 144.15)	142.33 (± 2.15)
Physiological stressor	[C] Rosiglitazone (chronic)	143 (139.45 - 144.55)	142.69 (± 1.49)				
Physiological stressor	[E] Sodium bicarbonate	144.37 (143.8 - 145.12)	144.48 (± 0.43)				
Physiological stressor	[A] Unilateral nephrectomy	142.45 (141.15 - 143.85)	142.56 (± 0.75)	147.45 (143.65 - 168.55)	151.27 (± 10.27)	143.05 (142.55 - 144.95)	143.59 (± 1.21)
Physiological stressor	[B] Water deprivation (chronic)	143 (141 - 144.01)	142.81 (± 1.03)	143.18 (142 - 147)	143.73 (± 2.01)	144 (142.07 - 145)	143.68 (± 1.01)
No Effect	[E] Acetaminophen (chronic)	144 (143 - 144)	143.6 (± 0.55)			142 (141 - 144)	142.2 (± 1.3)
No Effect	[C] Buthionine sulphoxime	142.15 (138.4 - 144.9)	142.03 (± 2.42)	146.6 (140.2 - 148)	144.88 (± 3.51)	143.3 (142.1 - 145.1)	143.64 (± 1.36)
No Effect	[C] Ferrous sulphate	142.15 (140.5 - 143.9)	142.18 (± 1.28)	144.6 (144 - 144.6)	144.44 (± 0.26)	142.6 (141.7 - 144)	142.77 (± 0.91)
No Effect	[B] Ifosfamide	143 (142 - 144)	143.2 (± 0.63)	142 (142 - 142)	142 (± 0)	142 (142 - 145)	142.8 (± 1.3)
No Effect	[B] Lithocholic acid	143 (142 - 145)	143.4 (± 0.84)	142 (140 - 142)	141.6 (± 0.89)	144 (141 - 144)	143.2 (± 1.3)

Target organ	Toxin	24 h post dose		48 h post dose		168 h post dose	
		Median (min - max)	Mean (± SD)	Median (min - max)	Mean (± SD)	Median (min - max)	Mean (± SD)
No Effect	[E] Paraquat			141.5 (141.5 - 144.5)	142.7 (± 1.64)	143.5 (142.5 - 143.5)	143.1 (± 0.55)
No Effect	[D] Potassium dichromate	141.5 (138 - 147)	141.89 (± 2.41)	144 (142 - 150)	145 (± 3)	144 (142 - 146)	144.2 (± 1.48)
No Effect	[C] Trichlorethylene	140.95 (139.05 - 143.25)	140.98 (± 1.11)	146.25 (145.45 - 148.35)	146.65 (± 1.34)	147.15 (142.75 - 148.85)	146.61 (± 2.3)

A-F: Indicates Pharmaceutical Company & sample origin

Supplementary Table S5. Summary statistics for serum potassium (mmol/L) at 24 hrs, 48 hrs and 168 hrs post dose vehicle.

Target organ	Toxin	24 h post dose		48 h post dose		168 h post dose	
		Median (min - max)	Mean (± SD)	Median (min - max)	Mean (± SD)	Median (min - max)	Mean (± SD)
Liver	[E] 1,1-Dichloroethylene	6.37 (6 - 7.1)	6.46 (± 0.34)	6.1 (5.4 - 6.4)	5.98 (± 0.39)	6.5 (5.8 - 6.8)	6.38 (± 0.46)
Liver	[E] 1,2,3,4,5,6-hexachlorocyclohexane	6.47 (6.14 - 7.11)	6.51 (± 0.31)	5.95 (5.85 - 6.45)	6.05 (± 0.25)	6.35 (5.75 - 6.75)	6.33 (± 0.38)
Liver	[B] 1-Fluoropentane	6.5 (5.7 - 7.1)	6.49 (± 0.4)	5.8 (5.5 - 6)	5.74 (± 0.19)	6.2 (6 - 6.3)	6.16 (± 0.11)
Liver	[B] 2,4,6-Trihydroxyacetophenone (THA)	6.35 (5.8 - 6.9)	6.32 (± 0.34)	6 (5.7 - 6.3)	6.02 (± 0.24)	6.2 (6 - 6.7)	6.3 (± 0.33)
Liver	[B] 4-Amino-2,6-dichlorophenol (ADCP)	6.17 (5.81 - 7.35)	6.35 (± 0.47)	6.1 (5.99 - 6.13)	6.08 (± 0.05)	6.21 (6.09 - 6.41)	6.21 (± 0.13)
Liver	[C] Aflatoxin	6.19 (5.52 - 6.54)	6.08 (± 0.34)	5.84 (5.62 - 6.28)	5.9 (± 0.29)	6.42 (6.15 - 6.5)	6.37 (± 0.14)
Liver	[C] Allyl alcohol	6.44 (5.82 - 7.7)	6.55 (± 0.53)	5.92 (5.16 - 6.35)	5.76 (± 0.48)	6.03 (5.68 - 6.38)	6.03 (± 0.25)
Liver	[C] Allyl formate	6.2 (5.63 - 7.29)	6.23 (± 0.46)	5.82 (5.27 - 5.95)	5.74 (± 0.28)	6.97 (6.39 - 7.69)	7 (± 0.49)
Liver	[B] Azathioprine	6.17 (5.81 - 7.35)	6.35 (± 0.47)	6.1 (5.99 - 6.13)	6.08 (± 0.05)	6.21 (6.09 - 6.41)	6.21 (± 0.13)
Liver	[B] Bromobenzene	6.25 (6 - 6.7)	6.29 (± 0.23)	6 (5.8 - 6.9)	6.16 (± 0.44)	6.2 (5.9 - 6.5)	6.22 (± 0.24)
Liver	[C] Butylated hydroxytoluene	6.54 (5.67 - 7.48)	6.56 (± 0.58)	5.7 (5.42 - 6.41)	5.85 (± 0.41)	5.92 (5.31 - 6.33)	5.86 (± 0.39)
Liver	[D] Carbon tetrachloride	6.12 (5.15 - 6.55)	6.06 (± 0.42)	6.15 (5.45 - 6.85)	6.17 (± 0.5)	6.35 (5.25 - 7.35)	6.19 (± 0.8)
Liver	[C] Chlorpromazine	6.24 (5.68 - 6.74)	6.24 (± 0.42)	6.18 (5.88 - 6.4)	6.2 (± 0.2)	6.23 (5.88 - 6.7)	6.24 (± 0.3)

Target organ	Toxin	24 h post dose		48 h post dose		168 h post dose	
		Median (min - max)	Mean (± SD)	Median (min - max)	Mean (± SD)	Median (min - max)	Mean (± SD)
Liver	[B] Clofibrate	6.25 (6 - 6.7)	6.3 (± 0.22)	6.3 (5.9 - 7.3)	6.34 (± 0.57)	5.9 (5.4 - 6.3)	5.9 (± 0.32)
Liver	[B] Cyproterone acetate	6.3 (6 - 6.6)	6.29 (± 0.19)	6.1 (5.8 - 6.3)	6.04 (± 0.23)	6.1 (5.7 - 6.4)	6.12 (± 0.27)
Liver	[A] D-galactosamine	6.28 (5.05 - 7.5)	6.36 (± 0.71)	5.97 (5.73 - 6.42)	6.03 (± 0.25)	6.18 (5.1 - 6.9)	6.01 (± 0.78)
Liver	[B] Diethylhexylphthalate (DEHP)			5.9 (5.4 - 6.3)	5.88 (± 0.35)	6.4 (6 - 6.7)	6.36 (± 0.25)
Liver	[C] Dimethylformamide (DMF)	6.44 (5.9 - 7.1)	6.43 (± 0.4)	5.84 (5.38 - 6.48)	5.88 (± 0.4)	6.18 (5.61 - 6.78)	6.16 (± 0.44)
Liver	[C] Dimethylnitrosamine (DMN)	6.2 (5.34 - 7.09)	6.2 (± 0.51)	6.36 (5.93 - 6.88)	6.4 (± 0.4)	6.05 (5.81 - 7.26)	6.34 (± 0.58)
Liver	[A] Gadolinium chloride	5.78 (5.24 - 6.48)	5.84 (± 0.37)	7 (6.7 - 8.78)	7.29 (± 0.85)	6.38 (5.14 - 7.5)	6.27 (± 0.89)
Liver	[A, B, C, D, F] Hydrazine	6 (5.28 - 6.92)	6.01 (± 0.36)	6.56 (5.45 - 8.15)	6.6 (± 0.63)	6.45 (5.3 - 8.1)	6.62 (± 0.78)
Liver	[E] Hydrazine	8.85 (6.95 - 12.65)	9.25 (± 2.08)	5.15 (4.95 - 5.35)	5.15 (± 0.16)	5.05 (4.55 - 5.45)	4.99 (± 0.38)
Liver	[E] Indomethacin	6.56 (6.35 - 7.35)	6.68 (± 0.31)	6.15 (5.55 - 6.25)	5.95 (± 0.32)	6.25 (5.55 - 6.75)	6.13 (± 0.48)
Liver	[E] Ketoconazole			6.05 (5.65 - 6.25)	6.01 (± 0.23)	6.85 (6.05 - 7.15)	6.67 (± 0.45)
Liver	[C] Lead acetate	6.3 (5.34 - 7.06)	6.3 (± 0.62)	5.16 (5 - 5.63)	5.3 (± 0.29)	7.48 (6.18 - 7.78)	7.22 (± 0.62)
Liver	[A] Lipopolysaccharide (LPS)	6.05 (5.38 - 6.58)	5.97 (± 0.38)	6.95 (6.06 - 9.13)	7.44 (± 1.37)	6.36 (6.14 - 11.32)	7.48 (± 2.2)
Liver	[B] Methapyrilene	7.15 (6.65 - 7.75)	7.13 (± 0.32)	5.45 (5.15 - 5.75)	5.47 (± 0.24)	5.45 (5.15 - 5.65)	5.41 (± 0.21)
Liver	[E] Methylene dianiline	6.28 (5.88 - 6.9)	6.32 (± 0.3)	6.2 (6.1 - 6.7)	6.36 (± 0.27)	5.9 (5.8 - 6.3)	6.02 (± 0.22)
Liver	[C] Monocrotaline	6.24 (5.08 - 7.2)	6.14 (± 0.65)	6.42 (5.8 - 6.73)	6.32 (± 0.36)	6.07 (5.02 - 6.39)	5.76 (± 0.65)
Liver	[C] N-methylformamide (NMF)	6.35 (5.36 - 7.13)	6.33 (± 0.6)	6.06 (5.02 - 6.84)	6.06 (± 0.72)	5.81 (4.84 - 6.4)	5.71 (± 0.56)
Liver	[D] Phalloidin (chronic)			5.73 (5.39 - 6.56)	5.83 (± 0.46)	6.83 (6.06 - 7.27)	6.72 (± 0.51)
Liver	[E] Phenyl diisothiocyanate	6.45 (6.15 - 7.13)	6.5 (± 0.28)	6.2 (5.7 - 6.4)	6.14 (± 0.27)	6.2 (5.5 - 6.7)	6.18 (± 0.47)
Liver	[E] Phenyl isothiocyanate	6.22 (5.94 - 6.62)	6.24 (± 0.21)	6.65 (6.05 - 6.75)	6.49 (± 0.29)	5.85 (5.75 - 6.65)	6.01 (± 0.38)

Target organ	Toxin	24 h post dose		48 h post dose		168 h post dose	
		Median (min - max)	Mean (± SD)	Median (min - max)	Mean (± SD)	Median (min - max)	Mean (± SD)
Liver	[B] Retinyl palmitate	6.17 (5.81 - 7.35)	6.35 (± 0.47)	6.1 (5.99 - 6.13)	6.08 (± 0.05)	6.21 (6.09 - 6.41)	6.21 (± 0.13)
Liver	[B] Sodium Valproate			6.2 (6 - 6.6)	6.24 (± 0.22)	6.3 (5.7 - 6.5)	6.16 (± 0.34)
Liver	[C] a-Naphthylisothiocyanate (ANIT)	6.2 (4.85 - 6.66)	6.04 (± 0.53)	5.89 (4.9 - 6.35)	5.72 (± 0.54)	6.53 (6.06 - 6.88)	6.48 (± 0.3)
Kidney	[D] 2-Bromophenol	6.1 (5.1 - 6.6)	6 (± 0.51)	6.7 (6 - 8.2)	6.98 (± 1.02)	6.9 (5.8 - 8.28)	7.1 (± 0.92)
Kidney	[E] 3,5-Dichloroaniline hydrochloride	6.45 (5.7 - 6.68)	6.39 (± 0.29)	6.05 (5.15 - 6.25)	5.91 (± 0.44)	6.25 (6.15 - 6.65)	6.33 (± 0.19)
Kidney	[E] Atractyloside	6.47 (6.13 - 6.83)	6.48 (± 0.22)	6.3 (6 - 6.5)	6.28 (± 0.19)	6.2 (5.9 - 6.4)	6.14 (± 0.19)
Kidney	[D] Bromoethylamine hydrobromide	6.25 (5.5 - 7)	6.29 (± 0.44)	6.9 (5.5 - 8.8)	6.78 (± 1.32)	5.8 (5.4 - 6.28)	5.84 (± 0.36)
Kidney	[D] Cephaloridine	6.4 (5.8 - 7)	6.38 (± 0.34)	6.2 (5.5 - 6.7)	6.11 (± 0.59)	5.7 (5.4 - 7)	5.9 (± 0.63)
Kidney	[B] Chlorethanamine	6.2 (5.7 - 6.6)	6.16 (± 0.24)	6 (5.7 - 6.1)	5.92 (± 0.16)	6.2 (6.2 - 6.4)	6.26 (± 0.09)
Kidney	[A] Cisplatin	6.1 (5.7 - 7.1)	6.17 (± 0.4)	6.6 (5.9 - 9.6)	7.14 (± 1.49)	7.6 (6.2 - 8.8)	7.58 (± 0.98)
Kidney	[A] D-limonene (chronic)						
Kidney	[E] Dichlorophenyl succinimide	6.28 (5.88 - 6.9)	6.32 (± 0.3)	6.2 (6.1 - 6.7)	6.36 (± 0.27)	5.9 (5.8 - 6.3)	6.02 (± 0.22)
Kidney	[D] Ethylene glycol	6.2 (5.8 - 6.7)	6.24 (± 0.25)	6.6 (5.5 - 6.8)	6.32 (± 0.55)	7.1 (5.5 - 7.2)	6.58 (± 0.82)
Kidney	[A] Folic acid	5.95 (5.2 - 7.2)	6 (± 0.66)	6.3 (5.95 - 8.1)	6.57 (± 0.87)	6.6 (5.5 - 7.7)	6.54 (± 0.84)
Kidney	[A] Gentamicin	6.15 (5.5 - 6.4)	6.04 (± 0.28)	6.1 (5.5 - 8.7)	6.62 (± 1.27)	7.9 (6.6 - 8.2)	7.62 (± 0.67)
Kidney	[B] Maleic acid	6.17 (5.81 - 7.35)	6.35 (± 0.47)	6.1 (5.99 - 6.13)	6.08 (± 0.05)	6.21 (6.09 - 6.41)	6.21 (± 0.13)
Kidney	[A] N-phenylanthranilic acid (chronic)	6.57 (5.99 - 7.74)	6.66 (± 0.6)	6.11 (5.53 - 6.72)	6.04 (± 0.49)	5.69 (4.76 - 5.75)	5.41 (± 0.43)
Kidney	[D] Para-aminophenol	5.85 (5.55 - 6.45)	5.85 (± 0.25)	6.55 (5.85 - 7.05)	6.51 (± 0.43)	7.85 (6.65 - 8.15)	7.61 (± 0.65)
Kidney	[A] Puromycin			5.9 (5 - 7)	6.1 (± 0.88)	6.5 (4.6 - 7.5)	6.3 (± 1.18)
Kidney	[B] Vancomycin hydrochloride			6.2 (5.8 - 6.6)	6.14 (± 0.31)	6.2 (6 - 6.4)	6.16 (± 0.17)

Target organ	Toxin	24 h post dose		48 h post dose		168 h post dose	
		Median (min - max)	Mean (± SD)	Median (min - max)	Mean (± SD)	Median (min - max)	Mean (± SD)
Liver & Kidney	[E] Acetaminophen	6.96 (5.95 - 7.46)	6.77 (± 0.56)	6.15 (5.95 - 6.35)	6.13 (± 0.18)	6.25 (5.65 - 6.75)	6.15 (± 0.45)
Liver & Kidney	[B] Aurothiomalate	6.17 (5.81 - 7.35)	6.35 (± 0.47)	6.1 (5.99 - 6.13)	6.08 (± 0.05)	6.21 (6.09 - 6.41)	6.21 (± 0.13)
Liver & Kidney	[C] Chloroform	6.04 (5.53 - 6.96)	6.11 (± 0.49)	5.98 (5.81 - 7.78)	6.4 (± 0.82)	6.77 (6.18 - 7.02)	6.66 (± 0.39)
Liver & Kidney	[D] Cyclosporin	5.4 (5.1 - 6.6)	5.64 (± 0.53)	6.7 (6.3 - 7.3)	6.76 (± 0.4)	6.9 (6.1 - 7.4)	6.87 (± 0.56)
Liver & Kidney	[D] Dichlorobenzene	5.9 (5 - 6.8)	5.88 (± 0.51)	6.4 (5.1 - 7.4)	6.12 (± 0.97)	6.5 (6.3 - 7.2)	6.64 (± 0.36)
Liver & Kidney	[C] Ethionine	6.7 (5.68 - 7.11)	6.46 (± 0.48)	5.77 (4.93 - 6.36)	5.78 (± 0.54)	6.15 (5.82 - 6.57)	6.19 (± 0.27)
Liver & Kidney	[B] Hexachlorobutadiene (HCBD)			6.05 (5.85 - 6.25)	6.09 (± 0.17)	6.45 (5.35 - 6.85)	6.27 (± 0.57)
Liver & Kidney	[B] Mercuric chloride	6.4 (5.8 - 6.8)	6.36 (± 0.28)	5.7 (5.7 - 6)	5.78 (± 0.13)	6 (5.8 - 6.2)	6.02 (± 0.18)
Liver & Kidney	[E] Microcystin-LR			6.3 (6.1 - 6.5)	6.28 (± 0.15)	6.2 (6 - 7)	6.3 (± 0.4)
Liver & Kidney	[E] Rotenone	6.34 (5.88 - 6.56)	6.32 (± 0.22)	6.2 (5.3 - 6.4)	6.08 (± 0.44)	6.1 (6 - 6.7)	6.26 (± 0.32)
Liver & Kidney	[E] S-(1,2-dichlorovinyl)-cysteine (DCVC)	6.4 (5.82 - 6.84)	6.35 (± 0.31)	6 (5.9 - 6.6)	6.14 (± 0.28)	6.4 (6.1 - 6.5)	6.34 (± 0.18)
Liver & Kidney	[D] Thioacetamide	6.15 (5 - 6.7)	6.02 (± 0.52)	6.2 (5.1 - 6.6)	6.02 (± 0.59)	6.4 (5.6 - 6.6)	6.12 (± 0.48)
Pancreas	[E] 1-Cyano-2-hydroxy-3-butene	6.54 (6.14 - 6.68)	6.5 (± 0.17)	5.7 (5.5 - 6.9)	6.06 (± 0.68)	6.2 (6.1 - 6.6)	6.28 (± 0.19)
Pancreas	[C] Caerulin	6.12 (5.87 - 6.75)	6.18 (± 0.26)	6.31 (5.81 - 6.66)	6.27 (± 0.31)	6.37 (5.78 - 6.96)	6.29 (± 0.49)
Pancreas	[E] L-arginine	6.57 (5.98 - 7.45)	6.57 (± 0.38)	6.2 (6.1 - 7.2)	6.56 (± 0.54)	6.2 (5.8 - 6.5)	6.16 (± 0.27)
Pancreas	[B] Streptozotocin	6.6 (6.1 - 7.2)	6.56 (± 0.34)	6.1 (6 - 6.4)	6.14 (± 0.17)	5.9 (5.8 - 6.1)	5.92 (± 0.13)
Testicular	[D] 1,3-Dinitrobenzene	5.9 (5.5 - 6.9)	5.96 (± 0.41)	8 (6.9 - 9)	7.86 (± 0.81)	6 (5.7 - 6.7)	6.16 (± 0.42)
Testicular	[C] Cadmium chloride	6.25 (5.44 - 6.85)	6.17 (± 0.46)	6.16 (5.94 - 6.89)	6.28 (± 0.38)	5.86 (5.54 - 6.88)	6.08 (± 0.55)
Testicular	[D] Cadmium chloride	5.97 (5.48 - 6.35)	5.95 (± 0.27)	6.53 (5.41 - 7.52)	6.5 (± 0.76)	7.11 (6.01 - 7.44)	6.86 (± 0.62)
Testicular	[D] Carbendazim	5.85 (5.5 - 6.6)	5.89 (± 0.36)	7.1 (6.2 - 9.6)	7.72 (± 1.62)	8.8 (7.7 - 9.2)	8.58 (± 0.62)

Target organ	Toxin	24 h post dose		48 h post dose		168 h post dose	
		Median (min - max)	Mean (± SD)	Median (min - max)	Mean (± SD)	Median (min - max)	Mean (± SD)
Testicular	[D] Di-n-pentyl-phthalate	5.89 (5.53 - 6.23)	5.91 (± 0.26)	7.19 (6.2 - 10.05)	7.62 (± 1.48)	6.57 (5.77 - 7.59)	6.59 (± 0.65)
Testicular	[D] Ethane dimethane sulfonate (EDS)	5.55 (4.5 - 6.5)	5.67 (± 0.6)	6.3 (6.1 - 8.5)	6.8 (± 1)	8.2 (6.2 - 8.8)	7.72 (± 1.03)
Testicular	[D] Methoxyacetic acid	5.87 (5.32 - 6.46)	5.9 (± 0.33)	6.3 (5.22 - 7.32)	6.21 (± 0.82)	7.12 (6.24 - 7.92)	7.09 (± 0.61)
Multiple organ	[B] Adriamycin	6.3 (5.5 - 7.1)	6.28 (± 0.43)	5.8 (5.5 - 7)	6.06 (± 0.6)	6 (5.9 - 6.6)	6.14 (± 0.28)
Multiple organ	[C] Amphotericin B	6.2 (5.33 - 7.02)	6.25 (± 0.46)	6.18 (6.01 - 6.69)	6.26 (± 0.29)	6.56 (5.58 - 6.69)	6.23 (± 0.54)
Multiple organ	[C] Azaserine	6.44 (5.33 - 6.85)	6.33 (± 0.44)	6.14 (5.45 - 6.25)	5.99 (± 0.32)	5.98 (5.25 - 6.46)	5.98 (± 0.48)
Multiple organ	[A] Dexamethasone	5.6 (5.1 - 5.9)	5.55 (± 0.31)	8 (6.8 - 8.8)	7.82 (± 0.74)	7.9 (6.5 - 8.8)	7.82 (± 1.02)
Multiple organ	[E] Mitomycin-C	6.47 (6.24 - 6.9)	6.47 (± 0.21)	6.2 (5.8 - 6.6)	6.24 (± 0.3)	5.9 (5.5 - 6.2)	5.92 (± 0.29)
Physiological stressor	[C] 1,1-Dichloroethylene & maleic acid	6.2 (5.39 - 6.56)	6.14 (± 0.37)	5.78 (5.22 - 6.25)	5.67 (± 0.42)	6.47 (5.63 - 6.64)	6.19 (± 0.49)
Physiological stressor	[C] 2,4-Dinitrophenol	6.32 (5.44 - 7.62)	6.33 (± 0.6)	5.83 (5.48 - 6.19)	5.83 (± 0.32)	6.99 (6.04 - 7.35)	6.85 (± 0.5)
Physiological stressor	[B] 4-Pentenoic acid	6.25 (5.45 - 6.55)	6.14 (± 0.29)	5.95 (5.65 - 6.05)	5.89 (± 0.15)	7.25 (5.85 - 8.35)	7.09 (± 0.97)
Physiological stressor	[D] Acetazolamide	6.16 (5.55 - 6.55)	6.12 (± 0.35)	6.62 (6.08 - 6.95)	6.51 (± 0.35)	6.05 (5.45 - 6.95)	6.15 (± 0.55)
Physiological stressor	[C] Acivicin	6.04 (5.78 - 6.95)	6.2 (± 0.38)	6.08 (5.85 - 6.75)	6.26 (± 0.38)	6.55 (6.01 - 6.91)	6.53 (± 0.33)
Physiological stressor	[E] Ammonium chloride			5.9 (5.7 - 6.6)	6.12 (± 0.4)	6.3 (5.6 - 6.7)	6.22 (± 0.41)
Physiological stressor	[D] Carboplatin	5.5 (4.5 - 6.2)	5.43 (± 0.45)	8.14 (6.4 - 9.8)	8.19 (± 1.52)	7.6 (6.7 - 9.5)	7.88 (± 1.04)
Physiological stressor	[A] Choline and choline/methionine deficiency (chronic)	7 (5.7 - 8.4)	6.93 (± 0.99)	6.2 (5.6 - 6.4)	6.02 (± 0.35)	5.8 (5.7 - 7.1)	6.2 (± 0.62)
Physiological stressor	[B] Food restriction (chronic)	6.2 (5 - 6.6)	6.11 (± 0.49)	6.2 (6 - 6.4)	6.2 (± 0.18)	6.2 (6.1 - 6.4)	6.26 (± 0.13)
Physiological stressor	[D] Furosemide	6.15 (5.75 - 6.65)	6.15 (± 0.25)	7.25 (6.05 - 8.05)	7.23 (± 0.77)	5.95 (5.85 - 8.05)	6.71 (± 1.13)
Physiological stressor	[B] Insulin	7.1 (6.7 - 7.6)	7.18 (± 0.31)	5.4 (5.2 - 5.7)	5.44 (± 0.21)	5.4 (5.2 - 5.6)	5.4 (± 0.16)
Physiological stressor	[E] Methotrexate	6.49 (5.83 - 7.33)	6.49 (± 0.41)	6.1 (5.9 - 6.5)	6.2 (± 0.28)	6.3 (5.7 - 6.6)	6.2 (± 0.33)
Physiological stressor	[A] Partial hepatectomy	6.21 (5.81 - 6.51)	6.17 (± 0.2)	5.76 (5.66 - 6.8)	6.04 (± 0.48)	6.2 (6.07 - 6.61)	6.27 (± 0.21)

Target organ	Toxin	24 h post dose		48 h post dose		168 h post dose	
		Median (min - max)	Mean (± SD)	Median (min - max)	Mean (± SD)	Median (min - max)	Mean (± SD)
Physiological stressor	[A] Phenobarbital (chronic)	5.9 (5.6 - 6.3)	5.96 (± 0.23)	7.3 (5.9 - 7.6)	6.9 (± 0.72)	6.8 (5.8 - 7.4)	6.72 (± 0.59)
Physiological stressor	[A] Pregnenolone 16 alpha carbonitrile (chronic)						
Physiological stressor	[A] Probenecid	6.06 (5.44 - 7.08)	6.26 (± 0.59)	6.81 (4.03 - 8.79)	6.56 (± 1.71)	5.62 (4.77 - 7.69)	5.92 (± 1.24)
Physiological stressor	[C] Rosiglitazone	6.22 (5.58 - 6.92)	6.28 (± 0.39)	6 (5.92 - 6.42)	6.11 (± 0.22)	5.9 (5.35 - 6.53)	5.98 (± 0.46)
Physiological stressor	[C] Rosiglitazone (chronic)	6.2 (5.78 - 6.62)	6.18 (± 0.31)				
Physiological stressor	[E] Sodium bicarbonate	6.34 (6.09 - 6.53)	6.32 (± 0.16)				
Physiological stressor	[A] Unilateral nephrectomy	6.28 (5.64 - 6.85)	6.24 (± 0.33)	5.89 (5.84 - 6.58)	6.02 (± 0.31)	6.2 (5.92 - 7.66)	6.44 (± 0.71)
Physiological stressor	[B] Water deprivation (chronic)	6.3 (5.9 - 6.5)	6.25 (± 0.16)	6 (5.7 - 6.2)	5.98 (± 0.17)	6.16 (6.1 - 6.8)	6.25 (± 0.27)
No Effect	[E] Acetaminophen (chronic)	6.1 (5.9 - 6.3)	6.14 (± 0.17)			6.4 (5.4 - 6.7)	6.22 (± 0.51)
No Effect	[C] Buthionine sulphoxime	6.49 (5.82 - 7.11)	6.46 (± 0.46)	5.8 (5.36 - 6.64)	5.89 (± 0.49)	6.2 (5.61 - 6.42)	6.03 (± 0.4)
No Effect	[C] Ferrous sulphate	6.42 (5.38 - 6.83)	6.33 (± 0.5)	6.13 (5.45 - 6.38)	5.92 (± 0.43)	6.46 (6.17 - 6.59)	6.38 (± 0.18)
No Effect	[B] Ifosfamide	6.15 (5.8 - 6.8)	6.2 (± 0.32)	6.2 (5.7 - 6.5)	6.14 (± 0.29)	6.2 (5.8 - 6.3)	6.14 (± 0.21)
No Effect	[B] Lithocholic acid	6.2 (5.7 - 6.6)	6.2 (± 0.23)	6.2 (6 - 6.4)	6.2 (± 0.2)	6.3 (5.9 - 6.6)	6.28 (± 0.26)
No Effect	[E] Paraquat			6.5 (5.5 - 7.6)	6.5 (± 0.76)	6.1 (5.7 - 6.2)	5.98 (± 0.26)
No Effect	[D] Potassium dichromate	6.08 (5.2 - 6.6)	5.97 (± 0.48)	6.2 (5.7 - 9.3)	6.64 (± 1.5)	6.6 (5.9 - 8)	6.64 (± 0.84)
No Effect	[C] Trichlorethylene	6.09 (5.34 - 6.44)	6.01 (± 0.35)	6.22 (6.02 - 6.56)	6.24 (± 0.24)	6.67 (6.13 - 6.92)	6.62 (± 0.32)

A-F: Indicates Pharmaceutical Company & sample origin

Supplementary Table S6. Summary statistics for serum phosphate (mmol/L) at 24 hrs, 48 hrs and 168 hrs post dose vehicle.

**Target organ**	**Toxin**	**24 h post dose**		**48 h post dose**		**168 h post dose**	
		**Median (min - max)**	**Mean (± SD)**	**Median (min - max)**	**Mean (± SD)**	**Median (min - max)**	**Mean (± SD)**
Liver	[E] 1,1-Dichloroethylene	3.14 (2.75 - 3.36)	3.14 (± 0.17)	2.85 (2.65 - 3.49)	3 (± 0.34)	2.85 (2.72 - 3.11)	2.88 (± 0.16)
Liver	[E] 1,2,3,4,5,6-hexachlorocyclohexane	3.32 (3.07 - 3.56)	3.29 (± 0.16)	2.85 (2.81 - 3.36)	3.01 (± 0.26)	2.62 (2.56 - 3.07)	2.73 (± 0.21)
Liver	[B] 1-Fluoropentane	3.25 (2.86 - 3.41)	3.22 (± 0.16)	2.96 (2.86 - 2.99)	2.95 (± 0.05)	2.9 (2.7 - 3.25)	2.98 (± 0.23)
Liver	[B] 2,4,6-Trihydroxyacetophenone (THA)	3.2 (2.88 - 3.4)	3.16 (± 0.16)	2.85 (2.72 - 3.11)	2.91 (± 0.18)	2.72 (2.56 - 3.23)	2.88 (± 0.33)
Liver	[B] 4-Amino-2,6-dichlorophenol (ADCP)	2.92 (2.8 - 2.96)	2.91 (± 0.05)	2.9 (2.76 - 3.12)	2.96 (± 0.15)	3.21 (3.21 - 3.29)	3.23 (± 0.04)
Liver	[C] Aflatoxin	2.97 (2.66 - 3.37)	3 (± 0.21)	3.03 (2.94 - 3.13)	3.04 (± 0.08)	3.49 (3.28 - 4.06)	3.62 (± 0.3)
Liver	[C] Allyl alcohol	3.34 (2.69 - 3.51)	3.26 (± 0.25)	2.87 (2.7 - 3.21)	2.94 (± 0.2)	2.93 (2.7 - 3.68)	3.05 (± 0.37)
Liver	[C] Allyl formate	3.22 (2.94 - 3.62)	3.25 (± 0.2)	3.08 (2.97 - 3.4)	3.17 (± 0.19)	2.96 (2.63 - 3.04)	2.91 (± 0.17)
Liver	[B] Azathioprine	2.92 (2.8 - 2.96)	2.91 (± 0.05)	2.9 (2.76 - 3.12)	2.96 (± 0.15)	3.21 (3.21 - 3.29)	3.23 (± 0.04)
Liver	[B] Bromobenzene	3.11 (2.85 - 3.46)	3.12 (± 0.18)	3.04 (2.98 - 3.2)	3.07 (± 0.09)	3.07 (2.81 - 3.2)	3.03 (± 0.17)
Liver	[C] Butylated hydroxytoluene	3.2 (2.82 - 3.52)	3.19 (± 0.24)	3.14 (2.97 - 3.96)	3.31 (± 0.39)	3.03 (2.88 - 3.23)	3.01 (± 0.14)
Liver	[D] Carbon tetrachloride	2.51 (1.81 - 2.98)	2.53 (± 0.39)	3.21 (3.11 - 3.51)	3.27 (± 0.18)	3.11 (2.91 - 3.61)	3.17 (± 0.26)
Liver	[C] Chlorpromazine	3.68 (3.09 - 4.24)	3.64 (± 0.43)	2.98 (2.92 - 3.31)	3.03 (± 0.16)	2.67 (2.29 - 3)	2.68 (± 0.27)
Liver	[B] Clofibrate	3.32 (2.96 - 3.44)	3.25 (± 0.16)	2.99 (2.9 - 3.19)	3.02 (± 0.12)	3.06 (2.8 - 3.12)	3.01 (± 0.14)
Liver	[B] Cyproterone acetate	3.27 (3.04 - 3.49)	3.29 (± 0.16)	2.98 (2.81 - 3.27)	3 (± 0.17)	2.88 (2.85 - 3.11)	2.94 (± 0.12)
Liver	[A] D-galactosamine	2.9 (2.73 - 3.06)	2.88 (± 0.13)	3.67 (3.61 - 3.7)	3.67 (± 0.04)	3.48 (3.15 - 4.09)	3.49 (± 0.38)
Liver	[B] Diethylhexylphthalate (DEHP)			3.09 (2.93 - 3.54)	3.16 (± 0.25)	3.12 (3.06 - 3.38)	3.15 (± 0.13)
Liver	[C] Dimethylformamide (DMF)	3.14 (2.95 - 3.41)	3.17 (± 0.16)	2.74 (2.58 - 3.19)	2.78 (± 0.24)	3.05 (2.62 - 3.3)	3.01 (± 0.28)
Liver	[C] Dimethylnitrosamine (DMN)	2.95 (2.83 - 3.25)	2.99 (± 0.12)	3.5 (3.43 - 3.91)	3.59 (± 0.19)	3.28 (2.79 - 3.34)	3.11 (± 0.28)
Liver	[A] Gadolinium chloride	2.87 (2.57 - 3.07)	2.88 (± 0.17)	3.62 (3.33 - 3.81)	3.63 (± 0.2)	3.43 (3.14 - 3.98)	3.45 (± 0.33)

Target organ	Toxin	24 h post dose		48 h post dose		168 h post dose	
		Median (min - max)	Mean (± SD)	Median (min - max)	Mean (± SD)	Median (min - max)	Mean (± SD)
Liver	[A, B, C, D, F] Hydrazine	3 (2.51 - 3.67)	3.03 (± 0.26)	3.27 (2.87 - 3.96)	3.37 (± 0.32)	3.1 (2.73 - 4.01)	3.14 (± 0.36)
Liver	[E] Hydrazine	3.51 (3.25 - 3.69)	3.48 (± 0.16)	3.08 (2.84 - 3.2)	3.06 (± 0.15)	3.19 (2.91 - 3.44)	3.15 (± 0.21)
Liver	[E] Indomethacin	3.3 (2.99 - 3.7)	3.31 (± 0.2)	2.99 (2.83 - 3.06)	2.95 (± 0.1)	2.86 (2.48 - 3.22)	2.84 (± 0.26)
Liver	[E] Ketoconazole			2.85 (2.78 - 3.14)	2.93 (± 0.15)	3.36 (3.07 - 3.46)	3.3 (± 0.16)
Liver	[C] Lead acetate	3.29 (2.85 - 3.67)	3.26 (± 0.29)	2.76 (2.44 - 2.82)	2.67 (± 0.16)	3.14 (2.93 - 3.29)	3.13 (± 0.14)
Liver	[A] Lipopolysaccharide (LPS)	2.77 (2.62 - 3.07)	2.8 (± 0.16)	4.04 (3.62 - 4.56)	4.13 (± 0.4)	3.23 (3.14 - 4.14)	3.47 (± 0.43)
Liver	[B] Methapyrilene	3.28 (2.59 - 4.01)	3.32 (± 0.4)	3.07 (2.62 - 3.17)	2.94 (± 0.25)	2.91 (2.72 - 3.23)	2.93 (± 0.19)
Liver	[E] Methylene dianiline	3.14 (2.8 - 3.51)	3.17 (± 0.23)	3.19 (2.83 - 3.38)	3.11 (± 0.25)	2.86 (2.48 - 3.25)	2.9 (± 0.29)
Liver	[C] Monocrotaline	3.16 (2.92 - 3.72)	3.21 (± 0.24)	3.32 (2.9 - 3.44)	3.2 (± 0.24)	3.02 (2.92 - 3.11)	3.01 (± 0.07)
Liver	[C] N-methylformamide (NMF)	3.3 (2.79 - 3.81)	3.32 (± 0.3)	3.02 (2.65 - 3.11)	2.96 (± 0.19)	2.9 (2.66 - 3.3)	2.94 (± 0.23)
Liver	[D] Phalloidin (chronic)			3.01 (2.93 - 3.26)	3.03 (± 0.13)	3.24 (2.91 - 4.09)	3.36 (± 0.44)
Liver	[E] Phenyl diisothiocyanate	3.27 (3.17 - 3.62)	3.28 (± 0.13)	2.78 (2.56 - 3.04)	2.78 (± 0.19)	2.65 (2.49 - 3.04)	2.72 (± 0.24)
Liver	[E] Phenyl isothiocyanate	3.01 (2.59 - 3.27)	3.01 (± 0.22)	3.23 (2.85 - 3.4)	3.12 (± 0.23)	3.14 (3.07 - 3.27)	3.15 (± 0.07)
Liver	[B] Retinyl palmitate	2.92 (2.8 - 2.96)	2.91 (± 0.05)	2.9 (2.76 - 3.12)	2.96 (± 0.15)	3.21 (3.21 - 3.29)	3.23 (± 0.04)
Liver	[B] Sodium Valproate			3.11 (3.07 - 3.24)	3.14 (± 0.07)	3.07 (2.88 - 3.33)	3.09 (± 0.17)
Liver	[C] a-Naphthylisothiocyanate (ANIT)	3.2 (2.88 - 4)	3.23 (± 0.35)	3.07 (2.98 - 3.6)	3.16 (± 0.25)	3.08 (2.75 - 3.39)	3.07 (± 0.24)
Kidney	[D] 2-Bromophenol	2.76 (2.46 - 2.86)	2.72 (± 0.12)	3.86 (3.36 - 4.76)	4.04 (± 0.6)	3.86 (3.36 - 4.96)	3.94 (± 0.62)
Kidney	[E] 3,5-Dichloroaniline hydrochloride	3.33 (2.78 - 3.46)	3.26 (± 0.21)	3.11 (2.36 - 3.14)	2.96 (± 0.33)	3.07 (2.91 - 3.23)	3.07 (± 0.12)
Kidney	[E] Atractyloside	3.14 (2.94 - 3.33)	3.17 (± 0.13)	3.14 (2.85 - 3.56)	3.2 (± 0.3)	2.91 (2.62 - 3.53)	2.96 (± 0.36)
Kidney	[D] Bromoethylamine hydrobromide	3.01 (2.61 - 3.11)	2.93 (± 0.15)	3.91 (3.41 - 4.91)	3.99 (± 0.58)	3.91 (3.22 - 4.71)	3.85 (± 0.61)

Target organ	Toxin	24 h post dose		48 h post dose		168 h post dose	
		Median (min - max)	Mean (± SD)	Median (min - max)	Mean (± SD)	Median (min - max)	Mean (± SD)
Kidney	[D] Cephaloridine	3.01 (2.71 - 3.11)	3.01 (± 0.12)	4.11 (3.25 - 4.21)	3.91 (± 0.39)	3.71 (3.51 - 3.81)	3.65 (± 0.13)
Kidney	[B] Chlorethanamine	3.29 (2.91 - 3.46)	3.25 (± 0.18)	3.01 (2.94 - 3.17)	3.03 (± 0.09)	2.91 (2.75 - 3.14)	2.92 (± 0.16)
Kidney	[A] Cisplatin	2.61 (2.41 - 2.81)	2.58 (± 0.14)	3.71 (3.41 - 5.31)	4.15 (± 0.79)	3.71 (3.71 - 3.91)	3.77 (± 0.09)
Kidney	[A] D-limonene (chronic)						
Kidney	[E] Dichlorophenyl succinimide	3.14 (2.8 - 3.51)	3.17 (± 0.23)	3.19 (2.83 - 3.38)	3.11 (± 0.25)	2.86 (2.48 - 3.25)	2.9 (± 0.29)
Kidney	[D] Ethylene glycol	2.8 (2.51 - 3.11)	2.77 (± 0.19)	3.41 (3.11 - 3.81)	3.41 (± 0.27)	3.21 (3.01 - 3.41)	3.21 (± 0.16)
Kidney	[A] Folic acid	2.56 (2.36 - 3.08)	2.63 (± 0.3)	3.96 (3.22 - 4.46)	3.85 (± 0.47)	3.26 (3.06 - 4.06)	3.38 (± 0.4)
Kidney	[A] Gentamicin	2.71 (2.51 - 3.01)	2.75 (± 0.19)	3.51 (3.11 - 4.11)	3.63 (± 0.42)	3.51 (2.81 - 3.51)	3.29 (± 0.32)
Kidney	[B] Maleic acid	2.92 (2.8 - 2.96)	2.91 (± 0.05)	2.9 (2.76 - 3.12)	2.96 (± 0.15)	3.21 (3.21 - 3.29)	3.23 (± 0.04)
Kidney	[A] N-phenylanthranilic acid (chronic)	2.28 (2.07 - 3.06)	2.42 (± 0.36)	3.53 (3.3 - 3.75)	3.55 (± 0.17)	3.2 (3.11 - 3.33)	3.22 (± 0.11)
Kidney	[D] Para-aminophenol	2.91 (2.71 - 3.11)	2.93 (± 0.14)	3.61 (3.51 - 4.11)	3.71 (± 0.23)	4.31 (3.81 - 5.41)	4.49 (± 0.61)
Kidney	[A] Puromycin			2.76 (2.66 - 3.36)	2.88 (± 0.29)	3.26 (2.66 - 3.66)	3.24 (± 0.36)
Kidney	[B] Vancomycin hydrochloride			3.11 (2.88 - 3.33)	3.09 (± 0.18)	3.11 (2.85 - 3.2)	3.08 (± 0.15)
Liver & Kidney	[E] Acetaminophen	3.3 (2.98 - 3.72)	3.3 (± 0.21)	3.01 (2.59 - 3.17)	2.98 (± 0.23)	2.75 (2.46 - 2.91)	2.73 (± 0.17)
Liver & Kidney	[B] Aurothiomalate	2.92 (2.8 - 2.96)	2.91 (± 0.05)	2.9 (2.76 - 3.12)	2.96 (± 0.15)	3.21 (3.21 - 3.29)	3.23 (± 0.04)
Liver & Kidney	[C] Chloroform	3.13 (2.92 - 3.62)	3.19 (± 0.19)	3.04 (2.85 - 3.13)	3.01 (± 0.12)	3.12 (2.92 - 3.37)	3.13 (± 0.16)
Liver & Kidney	[D] Cyclosporin	2.56 (2.31 - 2.91)	2.56 (± 0.16)	3.71 (3.31 - 4.01)	3.69 (± 0.25)	3.71 (3.41 - 4.61)	3.87 (± 0.47)
Liver & Kidney	[D] Dichlorobenzene	2.71 (2.41 - 3.01)	2.72 (± 0.19)	3.71 (3.21 - 4.01)	3.67 (± 0.32)	3.81 (3.51 - 4.41)	3.89 (± 0.34)
Liver & Kidney	[C] Ethionine	3.36 (2.96 - 3.74)	3.36 (± 0.28)	3.09 (2.85 - 3.32)	3.04 (± 0.19)	3.04 (2.74 - 3.23)	3.01 (± 0.18)
Liver & Kidney	[B] Hexachlorobutadiene (HCBD)			3.06 (3.02 - 3.12)	3.06 (± 0.04)	3.19 (3.02 - 3.28)	3.16 (± 0.1)

Target organ	Toxin	24 h post dose		48 h post dose		168 h post dose	
		Median (min - max)	Mean (± SD)	Median (min - max)	Mean (± SD)	Median (min - max)	Mean (± SD)
Liver & Kidney	[B] Mercuric chloride	3.16 (2.91 - 3.46)	3.16 (± 0.17)	3.07 (2.98 - 3.3)	3.11 (± 0.13)	2.85 (2.69 - 3.2)	2.9 (± 0.22)
Liver & Kidney	[E] Microcystin-LR			3.15 (3.09 - 3.28)	3.19 (± 0.09)	2.9 (2.51 - 3.15)	2.84 (± 0.26)
Liver & Kidney	[E] Rotenone	3.04 (2.59 - 3.59)	3.08 (± 0.29)	3.3 (3.11 - 3.33)	3.24 (± 0.1)	3.11 (2.91 - 3.2)	3.06 (± 0.12)
Liver & Kidney	[E] S-(1,2-dichlorovinyl)-cysteine (DCVC)	3.25 (3.02 - 3.48)	3.25 (± 0.16)	3.09 (2.93 - 3.22)	3.1 (± 0.11)	2.96 (2.93 - 3.12)	3 (± 0.09)
Liver & Kidney	[D] Thioacetamide	2.64 (2.26 - 2.87)	2.58 (± 0.21)	3.16 (3.16 - 3.56)	3.29 (± 0.18)	3.26 (2.86 - 3.46)	3.22 (± 0.26)
Pancreas	[E] 1-Cyano-2-hydroxy-3-butene	3.07 (2.79 - 3.4)	3.1 (± 0.18)	3.46 (3.11 - 3.62)	3.43 (± 0.2)	2.94 (2.72 - 3.2)	2.96 (± 0.18)
Pancreas	[C] Caerulin	3.15 (2.86 - 3.53)	3.2 (± 0.2)	3.05 (2.62 - 3.63)	3.1 (± 0.39)	3.08 (2.66 - 3.27)	3 (± 0.23)
Pancreas	[E] L-arginine	3.22 (2.81 - 3.46)	3.17 (± 0.22)	3.14 (2.85 - 3.98)	3.32 (± 0.45)	2.85 (2.62 - 3.17)	2.85 (± 0.24)
Pancreas	[B] Streptozotocin	3.16 (3.05 - 3.5)	3.24 (± 0.18)	2.98 (2.79 - 3.11)	2.96 (± 0.14)	3.18 (2.95 - 3.27)	3.15 (± 0.13)
Testicular	[D] 1,3-Dinitrobenzene	2.92 (2.81 - 3.11)	2.96 (± 0.1)	4.41 (4.11 - 4.91)	4.43 (± 0.34)	4.11 (3.11 - 4.51)	3.99 (± 0.53)
Testicular	[C] Cadmium chloride	3.23 (2.84 - 3.78)	3.2 (± 0.28)	3.06 (2.87 - 3.32)	3.08 (± 0.17)	2.98 (2.91 - 3.39)	3.1 (± 0.21)
Testicular	[D] Cadmium chloride	2.69 (2.16 - 2.9)	2.64 (± 0.24)	3.7 (3.31 - 4.35)	3.81 (± 0.46)	4.34 (3.69 - 5.11)	4.34 (± 0.52)
Testicular	[D] Carbendazim	2.66 (2.31 - 2.91)	2.66 (± 0.17)	3.61 (3.31 - 3.91)	3.63 (± 0.24)	3.71 (3.51 - 5.01)	3.97 (± 0.61)
Testicular	[D] Di-n-pentyl-phthalate	2.61 (2.46 - 2.77)	2.59 (± 0.08)	4.1 (3.44 - 4.84)	4.07 (± 0.55)	4.44 (3.78 - 5.03)	4.31 (± 0.51)
Testicular	[D] Ethane dimethane sulfonate (EDS)	2.81 (2.61 - 3.21)	2.81 (± 0.19)	3.31 (3.21 - 3.51)	3.34 (± 0.12)	4.11 (3.11 - 4.41)	3.97 (± 0.52)
Testicular	[D] Methoxyacetic acid	2.81 (2.52 - 3.02)	2.8 (± 0.12)	3.38 (3.2 - 3.51)	3.35 (± 0.14)	4.44 (4.1 - 5.07)	4.55 (± 0.43)
Multiple organ	[B] Adriamycin	3.28 (2.94 - 3.43)	3.24 (± 0.17)	2.69 (2.46 - 3.27)	2.8 (± 0.3)	3.01 (2.85 - 3.2)	3.02 (± 0.14)
Multiple organ	[C] Amphotericin B	3.27 (2.91 - 3.47)	3.23 (± 0.18)	2.9 (2.66 - 3.19)	2.9 (± 0.21)	2.98 (2.78 - 3.2)	3.01 (± 0.17)
Multiple organ	[C] Azaserine	3.16 (3.04 - 3.46)	3.21 (± 0.14)	2.96 (2.32 - 3.12)	2.84 (± 0.32)	3.1 (2.68 - 3.27)	3.01 (± 0.23)
Multiple organ	[A] Dexamethasone	2.56 (2.36 - 2.76)	2.55 (± 0.12)	3.66 (3.46 - 3.86)	3.62 (± 0.17)	3.66 (3.46 - 3.86)	3.62 (± 0.17)
Multiple organ	[E] Mitomycin-C	3.27 (2.75 - 3.62)	3.22 (± 0.22)	2.98 (2.78 - 3.27)	3 (± 0.17)	2.81 (2.59 - 3.2)	2.85 (± 0.22)

Target organ	Toxin	24 h post dose		48 h post dose		168 h post dose	
		Median (min - max)	Mean (± SD)	Median (min - max)	Mean (± SD)	Median (min - max)	Mean (± SD)
Physiological stressor	[C] 1,1-Dichloroethylene & maleic acid	3.33 (2.57 - 3.59)	3.29 (± 0.29)	2.67 (2.17 - 2.7)	2.56 (± 0.22)	3.03 (2.81 - 3.14)	2.99 (± 0.13)
Physiological stressor	[C] 2,4-Dinitrophenol	3.11 (2.71 - 4.29)	3.2 (± 0.45)	2.79 (2.62 - 3.47)	2.91 (± 0.33)	3.28 (3.03 - 3.44)	3.26 (± 0.15)
Physiological stressor	[B] 4-Pentenoic acid	3.04 (2.88 - 3.23)	3.04 (± 0.11)	3.2 (2.91 - 3.49)	3.17 (± 0.23)	3.27 (2.88 - 3.53)	3.23 (± 0.25)
Physiological stressor	[D] Acetazolamide	2.96 (2.81 - 3.21)	2.98 (± 0.14)	3.41 (3.25 - 3.71)	3.43 (± 0.17)	3.41 (3.01 - 3.81)	3.37 (± 0.3)
Physiological stressor	[C] Acivicin	3.29 (3.1 - 3.82)	3.33 (± 0.23)	2.94 (2.84 - 3.08)	2.93 (± 0.1)	2.98 (2.69 - 3.39)	2.99 (± 0.25)
Physiological stressor	[E] Ammonium chloride			3.02 (2.86 - 3.38)	3.04 (± 0.21)	3.22 (2.93 - 3.28)	3.15 (± 0.14)
Physiological stressor	[D] Carboplatin	2.41 (1.96 - 2.96)	2.44 (± 0.32)	4.26 (3.26 - 5.36)	4.24 (± 0.76)	4.06 (3.76 - 4.66)	4.14 (± 0.34)
Physiological stressor	[A] Choline and choline/methionine deficiency (chronic)	2.58 (2.18 - 2.86)	2.56 (± 0.23)	3.61 (3.11 - 3.71)	3.55 (± 0.25)	3.47 (3.15 - 3.7)	3.47 (± 0.2)
Physiological stressor	[B] Food restriction (chronic)	3.11 (2.94 - 3.4)	3.12 (± 0.14)	3.02 (2.78 - 3.14)	2.99 (± 0.15)	3.07 (2.88 - 3.2)	3.07 (± 0.14)
Physiological stressor	[D] Furosemide	2.66 (2.46 - 2.96)	2.69 (± 0.13)	3.66 (3.46 - 4.16)	3.8 (± 0.34)	3.76 (3.26 - 4.16)	3.7 (± 0.35)
Physiological stressor	[B] Insulin	3.25 (2.99 - 3.6)	3.28 (± 0.21)	3.09 (2.89 - 3.18)	3.05 (± 0.11)	2.76 (2.76 - 3.09)	2.87 (± 0.16)
Physiological stressor	[E] Methotrexate	3.41 (0.38 - 4.64)	3.28 (± 1.1)	3.06 (2.7 - 3.12)	2.97 (± 0.18)	2.96 (2.8 - 3.09)	2.94 (± 0.11)
Physiological stressor	[A] Partial hepatectomy	2.91 (2.59 - 3.59)	2.99 (± 0.32)	3.73 (3.11 - 3.78)	3.55 (± 0.31)	3.11 (2.52 - 3.33)	2.97 (± 0.33)
Physiological stressor	[A] Phenobarbital (chronic)	2.81 (2.61 - 3.11)	2.83 (± 0.17)	3.81 (3.41 - 4.11)	3.77 (± 0.25)	4.21 (2.91 - 4.31)	3.89 (± 0.58)
Physiological stressor	[A] Pregnenolone 16 alpha carbonitrile (chronic)						
Physiological stressor	[A] Probenecid	2.83 (2.49 - 3.11)	2.83 (± 0.19)	3.92 (3.72 - 4.12)	3.94 (± 0.15)	3.32 (3.11 - 4.17)	3.46 (± 0.43)
Physiological stressor	[C] Rosiglitazone	3.57 (3.13 - 3.87)	3.51 (± 0.27)	2.82 (2.58 - 2.97)	2.8 (± 0.14)	2.57 (2.43 - 3.09)	2.72 (± 0.32)
Physiological stressor	[C] Rosiglitazone (chronic)	3.11 (2.57 - 3.2)	3.02 (± 0.21)				
Physiological stressor	[E] Sodium bicarbonate	3.14 (3.02 - 3.2)	3.11 (± 0.06)				
Physiological stressor	[A] Unilateral nephrectomy	2.86 (2.43 - 3.27)	2.87 (± 0.27)	3.4 (3.14 - 4.04)	3.47 (± 0.34)	3.01 (2.81 - 3.53)	3.08 (± 0.27)

Target organ	Toxin	24 h post dose		48 h post dose		168 h post dose	
		Median (min - max)	Mean (± SD)	Median (min - max)	Mean (± SD)	Median (min - max)	Mean (± SD)
Physiological stressor	B Water deprivation (chronic)	3.07 (2.85 - 3.3)	3.1 (± 0.15)	2.94 (2.72 - 3.04)	2.91 (± 0.12)	3.27 (3.2 - 3.3)	3.25 (± 0.04)
No Effect	E Acetaminophen (chronic)	3.09 (3.02 - 3.22)	3.12 (± 0.09)			3.12 (3.06 - 3.44)	3.21 (± 0.17)
No Effect	C Buthionine sulphoxime	3.38 (3.1 - 3.6)	3.34 (± 0.2)	2.8 (2.65 - 3.16)	2.85 (± 0.22)	2.93 (2.47 - 3.07)	2.81 (± 0.28)
No Effect	C Ferrous sulphate	3.12 (2.59 - 3.65)	3.15 (± 0.29)	2.98 (2.85 - 3.15)	2.99 (± 0.11)	3.23 (3 - 3.59)	3.28 (± 0.25)
No Effect	B Ifosfamide	3.23 (3.11 - 3.43)	3.25 (± 0.13)	2.94 (2.78 - 3.17)	2.95 (± 0.15)	2.94 (2.52 - 2.98)	2.85 (± 0.19)
No Effect	B Lithocholic acid	3.15 (2.85 - 3.62)	3.22 (± 0.26)	3.01 (2.78 - 3.11)	3 (± 0.13)	3.11 (2.98 - 3.36)	3.14 (± 0.17)
No Effect	E Paraquat			3.51 (2.7 - 4.38)	3.53 (± 0.61)	3.06 (2.67 - 3.15)	2.95 (± 0.2)
No Effect	D Potassium dichromate	2.36 (2.21 - 2.91)	2.43 (± 0.25)	3.41 (3.11 - 4.31)	3.59 (± 0.51)	3.61 (3.41 - 4.31)	3.75 (± 0.34)
No Effect	C Trichlorethylene	3.13 (2.84 - 3.43)	3.14 (± 0.18)	2.61 (2.22 - 3.26)	2.65 (± 0.39)	3.12 (2.85 - 3.2)	3.04 (± 0.16)

A-F: Indicates Pharmaceutical Company & sample origin

Supplementary Table S7. Summary statistics for serum glucose (mmol/L) at 24 hrs, 48 hrs and 168 hrs post dose vehicle.

Target organ	Toxin	24 h post dose		48 h post dose		168 h post dose	
		Median (min - max)	Mean (± SD)	Median (min - max)	Mean (± SD)	Median (min - max)	Mean (± SD)
Liver	E 1,1-Dichloroethylene	7.83 (5.72 - 8.66)	7.76 (± 0.83)	13.49 (12.16 - 14.6)	13.44 (± 1.12)	14.99 (10.66 - 16.26)	14.28 (± 2.19)
Liver	E 1,2,3,4,5,6-hexachlorocyclohexane	7.81 (7.05 - 11.44)	8.04 (± 1.28)	14.38 (11.16 - 14.54)	13.29 (± 1.59)	13.1 (11.99 - 14.65)	13.41 (± 1.06)
Liver	B 1-Fluoropentane	9.57 (8.97 - 10.43)	9.67 (± 0.48)	10.01 (8.27 - 11.7)	10.03 (± 1.22)	9.95 (8.82 - 12.72)	10.25 (± 1.55)
Liver	B 2,4,6-Trihydroxyacetophenone (THA)	9.28 (7.49 - 10.51)	9.26 (± 0.97)	10.38 (9.21 - 12.9)	10.48 (± 1.48)	10.99 (10.67 - 12.61)	11.32 (± 0.77)
Liver	B 4-Amino-2,6-dichlorophenol (ADCP)	9.99 (9.62 - 11.42)	10.25 (± 0.59)	10.23 (9.6 - 10.48)	10.14 (± 0.36)	10.31 (9.99 - 10.73)	10.31 (± 0.32)
Liver	C Aflatoxin	7.35 (6.4 - 8.1)	7.17 (± 0.53)	8.4 (6.9 - 10)	8.44 (± 1.41)	5.7 (5.4 - 6.8)	5.94 (± 0.56)

Target organ	Toxin	24 h post dose		48 h post dose		168 h post dose	
		Median (min - max)	Mean (± SD)	Median (min - max)	Mean (± SD)	Median (min - max)	Mean (± SD)
Liver	[C] Allyl alcohol	8.27 (7.79 - 8.78)	8.27 (± 0.32)	9.85 (8.78 - 10.29)	9.62 (± 0.71)	8.59 (7.93 - 10.06)	8.85 (± 0.8)
Liver	[C] Allyl formate	8.7 (8.3 - 10.1)	8.82 (± 0.51)	8.6 (7.1 - 13.4)	9.52 (± 2.53)	8.2 (6.8 - 8.2)	7.86 (± 0.61)
Liver	[B] Azathioprine	9.99 (9.62 - 11.42)	10.25 (± 0.59)	10.23 (9.6 - 10.48)	10.14 (± 0.36)	10.31 (9.99 - 10.73)	10.31 (± 0.32)
Liver	[B] Bromobenzene	10.6 (8.84 - 14.15)	11.18 (± 1.72)	12.93 (10.71 - 20.04)	14 (± 3.77)	10.82 (8.44 - 13.43)	10.82 (± 2.08)
Liver	[C] Butylated hydroxytoluene	8.69 (8.01 - 9.76)	8.86 (± 0.67)	10.54 (9.61 - 11.15)	10.4 (± 0.6)	8.66 (7.99 - 13.33)	9.99 (± 2.4)
Liver	[D] Carbon tetrachloride	8.4 (7.78 - 8.58)	8.32 (± 0.25)	12.28 (11.17 - 13.5)	12.48 (± 0.96)	10 (8.84 - 11.49)	10.2 (± 0.99)
Liver	[C] Chlorpromazine	8 (6.66 - 12.2)	8.27 (± 1.55)	8.85 (8.12 - 9.7)	8.95 (± 0.64)	8.18 (7.39 - 8.92)	8.25 (± 0.66)
Liver	[B] Clofibrate	10.18 (9.32 - 10.79)	10.05 (± 0.46)	10.25 (9.64 - 11.32)	10.47 (± 0.65)	10.12 (9.67 - 11.03)	10.23 (± 0.6)
Liver	[B] Cyproterone acetate	9.34 (8.69 - 11.54)	9.63 (± 1.03)	11.75 (8.62 - 13.05)	11.22 (± 1.7)	10.82 (9.41 - 14.68)	11.29 (± 2.02)
Liver	[A] D-galactosamine	8.19 (6.72 - 9.32)	8.12 (± 0.87)	11.04 (10.82 - 12.82)	11.69 (± 1.01)	12.71 (10.93 - 16.93)	13.08 (± 2.38)
Liver	[B] Diethylhexylphthalate (DEHP)			10.82 (7.27 - 12.77)	10.35 (± 2.03)	10.54 (9.27 - 11.77)	10.62 (± 1.01)
Liver	[C] Dimethylformamide (DMF)	7.6 (7 - 8.5)	7.72 (± 0.43)	9.1 (8.3 - 9.3)	8.92 (± 0.41)	8 (7 - 9.5)	8.32 (± 1.06)
Liver	[C] Dimethylnitrosamine (DMN)	7.9 (6.4 - 8.7)	7.73 (± 0.76)	8.2 (7.9 - 8.4)	8.22 (± 0.2)	9 (8.3 - 9.7)	8.99 (± 0.51)
Liver	[A] Gadolinium chloride	9.7 (7.7 - 11.4)	9.71 (± 1.24)	14.6 (13.1 - 15.04)	14.36 (± 0.8)	15.54 (11.1 - 15.65)	13.81 (± 2.45)
Liver	[A, B, C, D, F] Hydrazine	9 (7.46 - 10.72)	9.07 (± 0.76)	10.3 (7.91 - 12.3)	10.32 (± 1.23)	10.27 (7.93 - 14.4)	10.74 (± 1.78)
Liver	[E] Hydrazine	9.47 (7.73 - 10.64)	9.36 (± 0.85)	11.61 (11.08 - 11.83)	11.52 (± 0.3)	11.67 (10.91 - 13.99)	12.08 (± 1.26)
Liver	[E] Indomethacin	7.69 (5.94 - 9.07)	7.54 (± 1.1)	11.1 (8.94 - 14.38)	11.4 (± 1.96)	13.88 (9.66 - 15.71)	13.26 (± 2.47)
Liver	[E] Ketoconazole			12.43 (11.32 - 15.04)	12.8 (± 1.38)	13.16 (10.44 - 16.71)	13.29 (± 2.7)
Liver	[C] Lead acetate	9.2 (7.2 - 10.6)	9.05 (± 0.93)	9.2 (8.4 - 9.7)	9.08 (± 0.5)	7 (6.2 - 7.8)	6.92 (± 0.64)
Liver	[A] Lipopolysaccharide (LPS)	9.3 (8.38 - 11.04)	9.62 (± 0.82)	20.42 (12.77 - 21.81)	18.46 (± 4.11)	12.99 (8.71 - 30.47)	15.88 (± 8.5)

Target organ	Toxin	24 h post dose		48 h post dose		168 h post dose	
		Median (min - max)	Mean (± SD)	Median (min - max)	Mean (± SD)	Median (min - max)	Mean (± SD)
Liver	[B] Methapyrilene	10.16 (8.94 - 10.91)	9.93 (± 0.66)	13.14 (10.61 - 13.44)	12.34 (± 1.36)	10.08 (9.63 - 12.08)	10.63 (± 1.11)
Liver	[E] Methylene dianiline	7.94 (7.11 - 10.94)	8.22 (± 1.12)	11.27 (8.99 - 14.21)	11.45 (± 1.91)	10.32 (8.55 - 13.1)	10.44 (± 1.86)
Liver	[C] Monocrotaline	8.65 (7.9 - 10.6)	8.86 (± 0.91)	10.1 (8.6 - 10.2)	9.7 (± 0.7)	8.9 (8.4 - 12.8)	9.74 (± 1.84)
Liver	[C] N-methylformamide (NMF)	6.85 (6.4 - 9.6)	7.34 (± 0.97)	9.01 (8.64 - 10.13)	9.18 (± 0.57)	8.3 (6.9 - 9.4)	8.16 (± 0.93)
Liver	[D] Phalloidin (chronic)			7.29 (7.11 - 8.72)	7.61 (± 0.68)	9.01 (7.77 - 10.79)	9.12 (± 1.09)
Liver	[E] Phenyl diisothiocyanate	7.24 (6.61 - 8.44)	7.37 (± 0.53)	11.71 (8.94 - 15.1)	12.08 (± 2.72)	12.88 (10.49 - 13.54)	12.59 (± 1.26)
Liver	[E] Phenyl isothiocyanate	7.99 (5.55 - 11.32)	8.34 (± 1.87)	11.16 (9.88 - 12.1)	11.08 (± 1)	12.05 (11.21 - 13.88)	12.13 (± 1.05)
Liver	[B] Retinyl palmitate	9.99 (9.62 - 11.42)	10.25 (± 0.59)	10.23 (9.6 - 10.48)	10.14 (± 0.36)	10.31 (9.99 - 10.73)	10.31 (± 0.32)
Liver	[B] Sodium Valproate			9.05 (8.27 - 11.21)	9.3 (± 1.17)	8.6 (8.21 - 9.38)	8.73 (± 0.43)
Liver	[C] a-Naphthylisothiocyanate (ANIT)	8.48 (7.62 - 10.65)	8.53 (± 0.87)	9.44 (8.84 - 10.16)	9.4 (± 0.56)	8.84 (7.67 - 9.22)	8.7 (± 0.6)
Kidney	[D] 2-Bromophenol	9.05 (8.2 - 9.7)	9.08 (± 0.51)	14.8 (10.8 - 18.1)	14.36 (± 3.25)	14.1 (10.5 - 17.6)	14.18 (± 2.57)
Kidney	[E] 3,5-Dichloroaniline hydrochloride	7.66 (6.38 - 8.16)	7.6 (± 0.51)	11.21 (9.05 - 11.99)	10.82 (± 1.13)	11.6 (10.77 - 13.54)	11.89 (± 1.03)
Kidney	[E] Atractyloside	7.27 (6.77 - 9.49)	7.54 (± 0.8)	11.44 (9.99 - 13.32)	11.51 (± 1.25)	12.16 (9.6 - 13.54)	11.61 (± 1.69)
Kidney	[D] Bromoethylamine hydrobromide	7.25 (6.6 - 8.1)	7.26 (± 0.48)	9 (7.8 - 11.6)	9.14 (± 1.54)	9.5 (8 - 13.3)	9.73 (± 2.11)
Kidney	[D] Cephaloridine	7.9 (7.4 - 8.3)	7.89 (± 0.3)	9.18 (8.5 - 10.8)	9.4 (± 0.86)	8.3 (7.3 - 9.5)	8.3 (± 0.89)
Kidney	[B] Chlorethanamine	9.65 (8.87 - 10.86)	9.73 (± 0.69)	11.73 (10.68 - 12.2)	11.45 (± 0.64)	10.15 (8.64 - 12.29)	10.1 (± 1.39)
Kidney	[A] Cisplatin	8.25 (4.8 - 9.7)	7.92 (± 1.31)	13.1 (10.5 - 14.8)	12.84 (± 1.72)	13.2 (7.9 - 18)	12.98 (± 3.73)
Kidney	[A] D-limonene (chronic)						
Kidney	[E] Dichlorophenyl succinimide	7.94 (7.11 - 10.94)	8.22 (± 1.12)	11.27 (8.99 - 14.21)	11.45 (± 1.91)	10.32 (8.55 - 13.1)	10.44 (± 1.86)
Kidney	[D] Ethylene glycol	7.75 (7.3 - 8.8)	7.8 (± 0.46)	12 (9.9 - 15.9)	12.52 (± 2.2)	11.9 (7.2 - 12.5)	10.42 (± 2.44)

Target organ	Toxin	24 h post dose		48 h post dose		168 h post dose	
		Median (min - max)	Mean (± SD)	Median (min - max)	Mean (± SD)	Median (min - max)	Mean (± SD)
Kidney	[A] Folic acid	8.31 (7.1 - 9.2)	8.17 (± 0.66)	11.49 (9.3 - 13.9)	11.5 (± 1.91)	9.2 (8.1 - 18.2)	10.86 (± 4.22)
Kidney	[A] Gentamicin	8.57 (8.1 - 9.2)	8.58 (± 0.4)	9.7 (7.8 - 11.2)	9.62 (± 1.61)	10.2 (9.3 - 12.2)	10.66 (± 1.27)
Kidney	[B] Maleic acid	9.99 (9.62 - 11.42)	10.25 (± 0.59)	10.23 (9.6 - 10.48)	10.14 (± 0.36)	10.31 (9.99 - 10.73)	10.31 (± 0.32)
Kidney	[A] N-phenylanthranilic acid (chronic)	8.49 (7.66 - 12.82)	8.82 (± 1.5)	13.65 (11.21 - 14.15)	13.2 (± 1.22)	14.87 (13.65 - 16.59)	14.92 (± 1.26)
Kidney	[D] Para-aminophenol	8 (7.3 - 8.8)	8 (± 0.48)	13.4 (8.6 - 15.4)	12.3 (± 2.99)	14.5 (9.4 - 15.6)	13.24 (± 2.56)
Kidney	[A] Puromycin			11.2 (9.4 - 14.2)	11.3 (± 1.78)	9.4 (8.4 - 20.1)	12.08 (± 4.91)
Kidney	[B] Vancomycin hydrochloride			10.06 (7.81 - 11.78)	10.21 (± 1.57)	10.78 (9.04 - 12.18)	10.78 (± 1.21)
Liver & Kidney	[E] Acetaminophen	7.66 (6.38 - 9.77)	7.78 (± 1.07)	11.44 (9.6 - 13.1)	11.69 (± 1.45)	11.71 (9.71 - 12.88)	11.32 (± 1.32)
Liver & Kidney	[B] Aurothiomalate	9.99 (9.62 - 11.42)	10.25 (± 0.59)	10.23 (9.6 - 10.48)	10.14 (± 0.36)	10.31 (9.99 - 10.73)	10.31 (± 0.32)
Liver & Kidney	[C] Chloroform	7.7 (7 - 8.9)	7.79 (± 0.63)	9.4 (8.4 - 11)	9.52 (± 1.06)	8 (7.4 - 9)	8.18 (± 0.63)
Liver & Kidney	[D] Cyclosporin	9.4 (7.7 - 10.1)	9.29 (± 0.72)	12.6 (8.6 - 13.9)	11.34 (± 2.42)	12.2 (8.9 - 16.7)	12.6 (± 3.11)
Liver & Kidney	[D] Dichlorobenzene	9.35 (8.4 - 9.7)	9.2 (± 0.41)	8.5 (8.1 - 14.9)	10.28 (± 2.93)	11.3 (8.3 - 13.6)	11.02 (± 2.56)
Liver & Kidney	[C] Ethionine	7.28 (6.86 - 12.35)	7.9 (± 1.63)	9.69 (9.37 - 14.27)	10.6 (± 2.08)	7.98 (7.32 - 8.38)	7.83 (± 0.48)
Liver & Kidney	[B] Hexachlorobutadiene (HCBD)			8.27 (7.66 - 9.44)	8.54 (± 0.78)	8.94 (8.49 - 10.54)	9.1 (± 0.84)
Liver & Kidney	[B] Mercuric chloride	10.21 (8.33 - 12.32)	10.23 (± 1.2)	8.77 (8.38 - 9.71)	9.05 (± 0.62)	9.49 (8.99 - 10.32)	9.51 (± 0.5)
Liver & Kidney	[E] Microcystin-LR			11.44 (8.66 - 12.99)	11.2 (± 1.58)	10.99 (9.1 - 11.49)	10.51 (± 1.01)
Liver & Kidney	[E] Rotenone	8.83 (7.38 - 10.99)	8.84 (± 0.99)	12.82 (9.83 - 14.1)	12.07 (± 1.98)	13.1 (10.94 - 14.32)	12.5 (± 1.49)
Liver & Kidney	[E] S-(1,2-dichlorovinyl)-cysteine (DCVC)	8.52 (7.33 - 10.82)	8.73 (± 1.1)	10.32 (8.99 - 10.94)	10.14 (± 0.76)	10.77 (9.94 - 12.49)	11.01 (± 0.96)
Liver & Kidney	[D] Thioacetamide	8.12 (7.3 - 9.28)	8.1 (± 0.61)	10.52 (9.4 - 12.6)	10.82 (± 1.17)	9.2 (7.8 - 12.3)	9.7 (± 1.66)
Pancreas	[E] 1-Cyano-2-hydroxy-3-butene	7.91 (6.61 - 9.88)	8.03 (± 0.88)	13.71 (13.04 - 16.65)	14.16 (± 1.44)	13.43 (10.27 - 14.99)	13.11 (± 1.78)

Target organ	Toxin	24 h post dose		48 h post dose		168 h post dose	
		Median (min - max)	Mean (± SD)	Median (min - max)	Mean (± SD)	Median (min - max)	Mean (± SD)
Pancreas	[C] Caerulin	7.9 (7.3 - 9.2)	8.1 (± 0.59)	9.3 (8.1 - 11.7)	9.68 (± 1.35)	8.5 (8.2 - 8.8)	8.46 (± 0.23)
Pancreas	[E] L-arginine	6.86 (6.05 - 7.99)	6.84 (± 0.62)	14.27 (12.71 - 17.6)	14.97 (± 2.05)	11.77 (8.88 - 14.16)	11.97 (± 2.09)
Pancreas	[B] Streptozotocin	10.29 (8.9 - 10.76)	10.18 (± 0.5)	10.07 (9.9 - 10.6)	10.19 (± 0.31)	9.66 (9.23 - 10.14)	9.73 (± 0.38)
Testicular	[D] 1,3-Dinitrobenzene	8.1 (7.1 - 8.83)	7.97 (± 0.52)	17.2 (12.3 - 19.6)	16.26 (± 2.84)	13.7 (12.7 - 18.8)	14.78 (± 2.62)
Testicular	[C] Cadmium chloride	8 (6.5 - 11.3)	8.08 (± 1.54)	8.3 (7.5 - 9.9)	8.58 (± 0.93)	9.6 (7.5 - 11.5)	9.24 (± 1.6)
Testicular	[D] Cadmium chloride	8.3 (7.45 - 9.43)	8.28 (± 0.62)	9.81 (7.57 - 10.7)	9.37 (± 1.19)	8.46 (8.12 - 10.5)	9.12 (± 1.11)
Testicular	[D] Carbendazim	8.8 (7.9 - 9.8)	8.81 (± 0.62)	13.9 (13.1 - 15.5)	14.06 (± 0.89)	14.6 (14.4 - 20.2)	16.06 (± 2.49)
Testicular	[D] Di-n-pentyl-phthalate	9.1 (8.33 - 9.8)	9.03 (± 0.43)	8.43 (7.87 - 9.46)	8.5 (± 0.66)	5.97 (5.1 - 7.42)	6.33 (± 0.98)
Testicular	[D] Ethane dimethane sulfonate (EDS)	8.8 (7.5 - 9.7)	8.7 (± 0.73)	14.7 (10.7 - 15.9)	13.52 (± 2.31)	17.3 (12.5 - 22.7)	17.08 (± 4.21)
Testicular	[D] Methoxyacetic acid	8.13 (7.69 - 9.34)	8.37 (± 0.56)	12 (11.28 - 13.69)	12.29 (± 0.91)	8.67 (5.81 - 17.14)	10.2 (± 4.34)
Multiple organ	[B] Adriamycin	8.65 (8.1 - 11.6)	9.24 (± 1.25)	10.05 (8.71 - 10.71)	9.78 (± 0.79)	9.44 (8.38 - 10.77)	9.47 (± 0.86)
Multiple organ	[C] Amphotericin B	8.15 (7.6 - 9.1)	8.2 (± 0.47)	10.1 (8.8 - 11)	9.86 (± 0.87)	8.1 (7.2 - 9.3)	8.18 (± 0.88)
Multiple organ	[C] Azaserine	8 (7.3 - 8.6)	7.99 (± 0.42)	8.7 (8.3 - 10)	8.86 (± 0.68)	7.8 (7 - 8.1)	7.6 (± 0.47)
Multiple organ	[A] Dexamethasone	9.35 (8.5 - 10.2)	9.43 (± 0.65)	11 (9.4 - 17.1)	11.92 (± 3.18)	13.4 (12.6 - 19.5)	15.1 (± 3.02)
Multiple organ	[E] Mitomycin-C	7.16 (5.61 - 9.29)	7.21 (± 0.95)	12.88 (9.66 - 14.32)	12.41 (± 1.76)	13.1 (10.21 - 13.66)	12.31 (± 1.51)
Physiological stressor	[C] 1,1-Dichloroethylene & maleic acid	8.5 (7.5 - 10.3)	8.63 (± 0.77)	9.1 (7.3 - 11.4)	9.1 (± 1.56)	9.6 (8.5 - 13.2)	9.94 (± 1.89)
Physiological stressor	[C] 2,4-Dinitrophenol	8.3 (6.9 - 11.5)	8.73 (± 1.35)	8.8 (8.4 - 14.9)	10.4 (± 2.75)	9.5 (7.2 - 13.5)	10.34 (± 2.67)
Physiological stressor	[B] 4-Pentenoic acid	10.56 (9.48 - 12.41)	10.89 (± 0.99)	11.88 (9.53 - 16.72)	12.2 (± 2.83)	9.81 (8.98 - 10.5)	9.82 (± 0.57)
Physiological stressor	[D] Acetazolamide	8.7 (8.23 - 9.6)	8.77 (± 0.41)	11.27 (9.18 - 13)	10.99 (± 1.63)	10 (8.2 - 13.2)	10.38 (± 1.8)
Physiological stressor	[C] Acivicin	8.45 (8.1 - 9.7)	8.59 (± 0.46)	9.8 (8.9 - 11)	9.96 (± 0.84)	8.1 (7.4 - 8.9)	8.08 (± 0.54)
Physiological stressor	[E] Ammonium chloride			11.32 (10.21 - 12.49)	11.31 (± 0.82)	11.16 (8.94 - 13.27)	11.02 (± 1.78)

Target organ	Toxin	24 h post dose		48 h post dose		168 h post dose	
		Median (min - max)	Mean (± SD)	Median (min - max)	Mean (± SD)	Median (min - max)	Mean (± SD)
Physiological stressor	[D] Carboplatin	8.5 (8.1 - 9.2)	8.56 (± 0.35)	15.2 (14.1 - 20.7)	16.28 (± 2.67)	14.2 (13.6 - 17.5)	14.8 (± 1.59)
Physiological stressor	[A] Choline and choline/methionine deficiency (chronic)	8.1 (7.16 - 8.94)	8.05 (± 0.52)	12.4 (12 - 15.7)	13.06 (± 1.5)	13.32 (12.15 - 14.37)	13.19 (± 0.93)
Physiological stressor	[B] Food restriction (chronic)	10.57 (8.97 - 13.45)	10.85 (± 1.2)	9.78 (8.82 - 12.73)	10.28 (± 1.69)	9.55 (8.38 - 10.71)	9.47 (± 1.01)
Physiological stressor	[D] Furosemide	7.55 (6.4 - 8.2)	7.41 (± 0.57)	10.5 (9.3 - 15.3)	11.3 (± 2.42)	12.7 (10.4 - 17.9)	13.2 (± 3.1)
Physiological stressor	[B] Insulin	10.09 (9.4 - 11.19)	10.13 (± 0.58)	9.85 (9.06 - 10.08)	9.76 (± 0.4)	10.07 (9.58 - 11.35)	10.25 (± 0.71)
Physiological stressor	[E] Methotrexate	9.21 (6.22 - 13.1)	9.26 (± 2.02)	10.27 (8.55 - 10.71)	9.89 (± 0.86)	11.82 (9.16 - 12.55)	11.34 (± 1.33)
Physiological stressor	[A] Partial hepatectomy	9.05 (8.46 - 10.11)	9.16 (± 0.55)	11.92 (11.44 - 13.3)	12.13 (± 0.75)	11.83 (11.64 - 11.97)	11.81 (± 0.13)
Physiological stressor	[A] Phenobarbital (chronic)	8.1 (7.3 - 8.8)	8.08 (± 0.43)	12.7 (7.1 - 14.2)	11.8 (± 2.75)	18.2 (8.9 - 19.4)	15.52 (± 4.51)
Physiological stressor	[A] Pregnenolone 16 alpha carbonitrile (chronic)						
Physiological stressor	[A] Probenecid	8.94 (7.66 - 10.43)	8.94 (± 0.7)	13.88 (11.6 - 19.76)	14.62 (± 3.18)	13.1 (11.65 - 13.76)	12.88 (± 0.83)
Physiological stressor	[C] Rosiglitazone	8.45 (6.4 - 13.8)	9.04 (± 2.34)	7.8 (7.2 - 8.7)	7.84 (± 0.55)	8.2 (7.7 - 10.1)	8.44 (± 0.99)
Physiological stressor	[C] Rosiglitazone (chronic)	7.45 (6.8 - 8.8)	7.62 (± 0.61)				
Physiological stressor	[E] Sodium bicarbonate	8.47 (8.14 - 9.67)	8.68 (± 0.53)				
Physiological stressor	[A] Unilateral nephrectomy	8.89 (8.37 - 9.51)	8.86 (± 0.39)	12.48 (11.16 - 13.04)	12.17 (± 0.9)	10.9 (9.85 - 12.45)	11.02 (± 0.95)
Physiological stressor	[B] Water deprivation (chronic)	10.14 (9.01 - 11.84)	10.21 (± 0.9)	9.95 (8.84 - 16.5)	10.77 (± 2.85)	9.04 (8.32 - 10.56)	9.27 (± 0.82)
No Effect	[E] Acetaminophen (chronic)	10.55 (9.88 - 11.6)	10.57 (± 0.66)			13.16 (9.88 - 14.49)	12.61 (± 2.03)
No Effect	[C] Buthionine sulphoxime	8.7 (8.2 - 10.1)	8.91 (± 0.66)	9.5 (8.7 - 12.6)	10.2 (± 1.59)	8.5 (6.8 - 9.1)	8.1 (± 0.93)
No Effect	[C] Ferrous sulphate	8.7 (7.7 - 9.4)	8.52 (± 0.64)	8.4 (8.1 - 9.2)	8.56 (± 0.51)	8.16 (6.6 - 9.1)	7.97 (± 1.06)
No Effect	[B] Ifosfamide	9.06 (7.75 - 11.07)	9.21 (± 1)	8.96 (8.2 - 11.7)	9.4 (± 1.34)	10.32 (8.7 - 10.55)	9.89 (± 0.82)
No Effect	[B] Lithocholic acid	9.43 (7.38 - 10.93)	9.24 (± 1.14)	12.9 (9.82 - 18.25)	13.14 (± 3.27)	9.57 (6.71 - 12.67)	9.44 (± 2.15)

Target organ	Toxin	24 h post dose		48 h post dose		168 h post dose	
		Median (min - max)	Mean (± SD)	Median (min - max)	Mean (± SD)	Median (min - max)	Mean (± SD)
No Effect	E Paraquat			14.77 (13.66 - 19.15)	15.35 (± 2.22)	11.6 (10.94 - 13.04)	11.89 (± 0.81)
No Effect	D Potassium dichromate	8.4 (7.9 - 10)	8.6 (± 0.62)	13.8 (10.4 - 15.4)	13.36 (± 1.9)	13.1 (9.2 - 13.8)	12.16 (± 1.86)
No Effect	C Trichlorethylene	7.85 (6.8 - 9.9)	8.13 (± 0.97)	9.3 (7.3 - 9.9)	8.96 (± 1.08)	8.5 (7.8 - 10.4)	8.9 (± 1.11)

A-F: Indicates Pharmaceutical Company & sample origin

Supplementary Table S8. Summary statistics for serum albumin (g/L) at 24 hrs, 48 hrs and 168 hrs post dose vehicle.

Target organ	Toxin	24 h post dose		48 h post dose		168 h post dose	
		Median (min - max)	Mean (± SD)	Median (min - max)	Mean (± SD)	Median (min - max)	Mean (± SD)
Liver	E 1,1-Dichloroethylene	40.45 (38.45 - 43.45)	40.65 (± 1.81)	34.45 (33.45 - 36.45)	34.85 (± 1.14)	35.45 (33.45 - 36.45)	35.25 (± 1.3)
Liver	E 1,2,3,4,5,6-hexachlorocyclohexane	40.45 (36.99 - 43.45)	40.4 (± 1.79)	35.45 (34.45 - 35.45)	35.05 (± 0.55)	37.45 (34.45 - 37.45)	36.45 (± 1.41)
Liver	B 1-Fluoropentane	37.95 (34.95 - 38.95)	37.35 (± 1.43)	36.95 (36.95 - 37.95)	37.35 (± 0.55)	36.95 (34.95 - 39.95)	37.15 (± 1.92)
Liver	B 2,4,6-Trihydroxyacetophenone (THA)	37.45 (36.45 - 40.45)	38.15 (± 1.64)	37.45 (35.45 - 37.45)	36.85 (± 0.89)	37.45 (32.45 - 37.45)	35.85 (± 2.3)
Liver	B 4-Amino-2,6-dichlorophenol (ADCP)	37.02 (36.55 - 37.98)	37.04 (± 0.47)	36.5 (36.3 - 37)	36.58 (± 0.26)	36.45 (36.39 - 36.7)	36.52 (± 0.15)
Liver	C Aflatoxin	37.95 (34.45 - 40.45)	37.65 (± 1.75)	37.45 (35.45 - 37.45)	36.65 (± 1.1)	39.45 (37.45 - 40.45)	39.05 (± 1.14)
Liver	C Allyl alcohol	38.1 (35.5 - 39.6)	37.62 (± 1.5)	38.3 (34.4 - 39.4)	37.46 (± 1.93)	36.7 (34.1 - 37.6)	36.46 (± 1.38)
Liver	C Allyl formate	36.65 (34.15 - 38.45)	36.11 (± 1.64)	37.42 (35.64 - 37.71)	37.02 (± 0.85)	38.95 (37.85 - 39.55)	38.85 (± 0.75)
Liver	B Azathioprine	37.02 (36.55 - 37.98)	37.04 (± 0.47)	36.5 (36.3 - 37)	36.58 (± 0.26)	36.45 (36.39 - 36.7)	36.52 (± 0.15)
Liver	B Bromobenzene	38.45 (35.45 - 41.45)	38.35 (± 1.79)	36.45 (33.45 - 39.45)	36.65 (± 2.39)	36.45 (35.45 - 41.45)	37.45 (± 2.35)
Liver	C Butylated hydroxytoluene	37.2 (35.3 - 39)	37.05 (± 1.07)	38 (35.8 - 38.4)	37.44 (± 1.18)	37.9 (34.6 - 40.4)	37.84 (± 2.08)
Liver	D Carbon tetrachloride	37.45 (36.45 - 38.45)	37.27 (± 0.64)	36.45 (34.45 - 38.45)	36.65 (± 1.79)	38.45 (36.45 - 39.45)	38.25 (± 1.1)
Liver	C Chlorpromazine	38.3 (33.65 - 39.95)	37.76 (± 2.06)	36.55 (35.65 - 38.95)	36.97 (± 1.31)	35.15 (34.65 - 38.65)	36.13 (± 1.78)

Target organ	Toxin	24 h post dose		48 h post dose		168 h post dose	
		Median (min - max)	Mean (± SD)	Median (min - max)	Mean (± SD)	Median (min - max)	Mean (± SD)
Liver	[B] Clofibrate	37.45 (35.45 - 39.45)	37.45 (± 1.33)	36.45 (35.45 - 37.45)	36.45 (± 1)	37.45 (34.45 - 38.45)	36.85 (± 1.52)
Liver	[B] Cyproterone acetate	37.45 (34.95 - 39.95)	37.65 (± 1.77)	33.95 (32.95 - 35.95)	34.15 (± 1.1)	38.95 (37.95 - 41.95)	39.35 (± 1.52)
Liver	[A] D-galactosamine	37.14 (36.37 - 37.88)	37.12 (± 0.49)	37.45 (35.45 - 37.45)	36.85 (± 0.89)	38.45 (34.45 - 41.45)	38.65 (± 2.95)
Liver	[B] Diethylhexylphthalate (DEHP)			37.45 (36.45 - 38.45)	37.45 (± 1)	37.45 (35.45 - 37.45)	36.85 (± 0.89)
Liver	[C] Dimethylformamide (DMF)	37.1 (35.1 - 40.5)	37.54 (± 1.71)	37.8 (36.1 - 38.4)	37.42 (± 1.06)	37.9 (36.7 - 39.1)	37.86 (± 0.98)
Liver	[C] Dimethylnitrosamine (DMN)	37.55 (33.65 - 38.55)	37.13 (± 1.35)	37.45 (36.25 - 37.95)	37.23 (± 0.75)	37.05 (36.05 - 38.95)	37.25 (± 1.18)
Liver	[A] Gadolinium chloride	36.95 (35.95 - 38.95)	37.05 (± 1.2)	37.95 (36.95 - 38.95)	37.75 (± 0.84)	37.95 (35.95 - 39.95)	38.35 (± 1.67)
Liver	[A, B, C, D, F] Hydrazine	37.45 (33.2 - 41.45)	37.11 (± 1.68)	37.45 (32.3 - 40.45)	37.46 (± 1.8)	37.45 (33.45 - 41.45)	38.02 (± 1.79)
Liver	[E] Hydrazine	37.8 (37.6 - 38.1)	37.83 (± 0.15)	37 (36.9 - 37.1)	37 (± 0.07)	37.2 (37.1 - 37.3)	37.22 (± 0.08)
Liver	[E] Indomethacin	40.45 (36.73 - 41.45)	39.94 (± 1.53)	36.45 (34.45 - 37.45)	36.45 (± 1.22)	35.45 (35.45 - 36.45)	35.85 (± 0.55)
Liver	[E] Ketoconazole			38.45 (36.45 - 39.45)	38.25 (± 1.3)	36.45 (35.45 - 37.45)	36.45 (± 1)
Liver	[C] Lead acetate	38.05 (36.3 - 38.6)	37.74 (± 0.86)	34.6 (32.7 - 35.2)	34.4 (± 1.01)	39.1 (35.9 - 41)	38.66 (± 1.92)
Liver	[A] Lipopolysaccharide (LPS)	35.95 (33.45 - 37.45)	35.65 (± 1.32)	38.45 (37.45 - 38.45)	38.05 (± 0.55)	38.45 (37.45 - 40.45)	38.45 (± 1.22)
Liver	[B] Methapyrilene	37.45 (36.45 - 39.45)	37.65 (± 1.03)	36.45 (34.45 - 37.45)	36.25 (± 1.1)	37.45 (34.45 - 38.45)	37.05 (± 1.67)
Liver	[E] Methylene dianiline	38.95 (36.95 - 40.95)	38.95 (± 1.33)	34.95 (34.95 - 37.95)	35.75 (± 1.3)	34.95 (33.95 - 35.95)	34.95 (± 1)
Liver	[C] Monocrotaline	37.15 (33.6 - 38.3)	36.75 (± 1.56)	38.8 (36 - 41.3)	38.74 (± 2.23)	37.5 (36.5 - 39.1)	37.78 (± 1)
Liver	[C] N-methylformamide (NMF)	37.85 (33.5 - 40)	37.41 (± 1.79)	37 (34.7 - 37.3)	36.6 (± 1.08)	37.3 (35.4 - 38.3)	37.14 (± 1.07)
Liver	[D] Phalloidin (chronic)			36.11 (33.53 - 38.23)	36.22 (± 1.74)	39.45 (36.39 - 43.09)	39.46 (± 2.59)
Liver	[E] Phenyl diisothiocyanate	38.45 (38.45 - 41.45)	38.85 (± 0.97)	34.45 (33.45 - 34.45)	34.05 (± 0.55)	36.45 (35.45 - 36.45)	36.05 (± 0.55)
Liver	[E] Phenyl isothiocyanate	38.95 (37.45 - 41.45)	39.25 (± 1.48)	36.45 (35.45 - 37.45)	36.45 (± 0.71)	36.45 (34.45 - 36.45)	35.85 (± 0.89)

Target organ	Toxin	24 h post dose		48 h post dose		168 h post dose	
		Median (min - max)	Mean (± SD)	Median (min - max)	Mean (± SD)	Median (min - max)	Mean (± SD)
Liver	[B] Retinyl palmitate	37.02 (36.55 - 37.98)	37.04 (± 0.47)	36.5 (36.3 - 37)	36.58 (± 0.26)	36.45 (36.39 - 36.7)	36.52 (± 0.15)
Liver	[B] Sodium Valproate			35.95 (35.95 - 37.95)	36.55 (± 0.89)	37.95 (36.95 - 39.95)	38.55 (± 1.34)
Liver	[C] a-Naphthylisothiocyanate (ANIT)	37.45 (34.65 - 57.45)	39.25 (± 6.56)	35.75 (34.75 - 38.85)	36.79 (± 1.92)	37.85 (30.65 - 40.15)	36.75 (± 3.6)
Kidney	[D] 2-Bromophenol	36.45 (34.45 - 40.45)	36.65 (± 1.75)	37.45 (31.45 - 38.45)	36.25 (± 2.95)	39.45 (37.45 - 40.45)	39.05 (± 1.14)
Kidney	[E] 3,5-Dichloroaniline hydrochloride	39.95 (37.95 - 40.95)	39.75 (± 1.03)	34.95 (32.95 - 36.95)	34.95 (± 1.41)	33.95 (33.95 - 34.95)	34.35 (± 0.55)
Kidney	[E] Atractyloside	39.45 (34.45 - 41.45)	38.95 (± 1.96)	37.45 (34.45 - 37.45)	36.65 (± 1.3)	34.45 (31.45 - 35.45)	34.05 (± 1.67)
Kidney	[D] Bromoethylamine hydrobromide	37.45 (33.45 - 39.45)	37.17 (± 1.71)	36.45 (35.45 - 39.45)	36.85 (± 1.67)	37.45 (34.45 - 38.45)	37.08 (± 1.53)
Kidney	[D] Cephaloridine	37.45 (35.45 - 38.45)	37.15 (± 1.16)	37.45 (36.83 - 40.45)	38.33 (± 1.54)	39.45 (37.45 - 41.45)	39.65 (± 1.79)
Kidney	[B] Chlorethanamine	38.45 (36.45 - 39.45)	37.95 (± 1.18)	36.45 (34.45 - 37.45)	36.45 (± 1.22)	37.45 (36.45 - 37.45)	37.05 (± 0.55)
Kidney	[A] Cisplatin	37.45 (34.45 - 41.45)	37.85 (± 1.84)	38.45 (36.45 - 41.45)	38.45 (± 2.12)	37.45 (34.45 - 39.45)	37.45 (± 1.87)
Kidney	[A] D-limonene (chronic)						
Kidney	[E] Dichlorophenyl succinimide	38.95 (36.95 - 40.95)	38.95 (± 1.33)	34.95 (34.95 - 37.95)	35.75 (± 1.3)	34.95 (33.95 - 35.95)	34.95 (± 1)
Kidney	[D] Ethylene glycol	36.45 (35.45 - 38.45)	36.97 (± 0.99)	38.45 (35.45 - 41.45)	38.65 (± 2.17)	38.45 (36.45 - 39.45)	38.05 (± 1.52)
Kidney	[A] Folic acid	37.88 (32.95 - 41.95)	37.38 (± 2.77)	34.95 (33.95 - 37.56)	35.47 (± 1.37)	38.95 (37.95 - 39.95)	38.95 (± 0.71)
Kidney	[A] Gentamicin	35.16 (33.95 - 38.17)	35.71 (± 1.52)	37.95 (34.95 - 38.95)	37.35 (± 1.52)	40.95 (37.95 - 40.95)	39.75 (± 1.64)
Kidney	[B] Maleic acid	37.02 (36.55 - 37.98)	37.04 (± 0.47)	36.5 (36.3 - 37)	36.58 (± 0.26)	36.45 (36.39 - 36.7)	36.52 (± 0.15)
Kidney	[A] N-phenylanthranilic acid (chronic)	36.45 (35.45 - 38.51)	36.75 (± 1.16)	37.45 (36.45 - 38.45)	37.65 (± 0.84)	37.45 (37.45 - 38.45)	37.65 (± 0.45)
Kidney	[D] Para-aminophenol	36.45 (34.45 - 39.45)	36.55 (± 1.52)	39.45 (38.45 - 41.45)	39.49 (± 1.18)	38.45 (36.45 - 39.45)	38.25 (± 1.3)
Kidney	[A] Puromycin			38.45 (34.45 - 38.45)	37.45 (± 1.73)	36.45 (36.45 - 39.45)	37.25 (± 1.3)
Kidney	[B] Vancomycin hydrochloride			37.45 (36.45 - 40.45)	38.05 (± 1.82)	37.45 (36.45 - 40.45)	38.05 (± 1.52)

Target organ	Toxin	24 h post dose		48 h post dose		168 h post dose	
		Median (min - max)	Mean (± SD)	Median (min - max)	Mean (± SD)	Median (min - max)	Mean (± SD)
Liver & Kidney	[E] Acetaminophen	39.95 (37.95 - 42.95)	40.35 (± 1.43)	36.95 (35.95 - 36.95)	36.55 (± 0.55)	36.95 (35.95 - 36.95)	36.75 (± 0.45)
Liver & Kidney	[B] Aurothiomalate	37.02 (36.55 - 37.98)	37.04 (± 0.47)	36.5 (36.3 - 37)	36.58 (± 0.26)	36.45 (36.39 - 36.7)	36.52 (± 0.15)
Liver & Kidney	[C] Chloroform	37.45 (35.5 - 42.1)	37.82 (± 1.81)	36.7 (36.3 - 38)	36.96 (± 0.72)	38.3 (36.5 - 38.9)	38.1 (± 0.94)
Liver & Kidney	[D] Cyclosporin	37.95 (34.45 - 40.45)	37.75 (± 1.89)	36.45 (35.45 - 39.45)	37.25 (± 1.64)	37.45 (36.45 - 40.45)	38.05 (± 1.52)
Liver & Kidney	[D] Dichlorobenzene	35.45 (35.45 - 38.45)	36.35 (± 1.29)	38.45 (36.45 - 39.45)	38.25 (± 1.3)	39.45 (37.45 - 41.45)	39.65 (± 1.48)
Liver & Kidney	[C] Ethionine	37.8 (36.4 - 40.3)	38.01 (± 1.23)	35.4 (34.4 - 35.9)	35.28 (± 0.57)	37.9 (34.5 - 40.7)	37.36 (± 2.56)
Liver & Kidney	[B] Hexachlorobutadiene (HCBD)			37.45 (35.45 - 39.45)	37.45 (± 1.41)	37.45 (36.45 - 39.45)	37.65 (± 1.3)
Liver & Kidney	[B] Mercuric chloride	37.95 (34.95 - 38.95)	37.45 (± 1.51)	36.95 (34.95 - 37.95)	36.55 (± 1.14)	37.95 (34.95 - 38.95)	37.55 (± 1.67)
Liver & Kidney	[E] Microcystin-LR			37.45 (36.45 - 38.45)	37.65 (± 0.84)	37.45 (36.45 - 38.45)	37.45 (± 1)
Liver & Kidney	[E] Rotenone	39.45 (38.45 - 41.45)	39.55 (± 0.74)	35.45 (34.45 - 36.45)	35.45 (± 0.71)	35.45 (33.45 - 36.45)	35.05 (± 1.14)
Liver & Kidney	[E] S-(1,2-dichlorovinyl)-cysteine (DCVC)	38.45 (37.95 - 39.95)	38.65 (± 0.82)	35.95 (35.95 - 36.95)	36.15 (± 0.45)	33.95 (31.95 - 34.95)	33.75 (± 1.3)
Liver & Kidney	[D] Thioacetamide	36.35 (34.95 - 36.95)	36.26 (± 0.64)	38.95 (37.43 - 40.95)	38.84 (± 1.35)	37.95 (35.95 - 39.95)	37.95 (± 1.58)
Pancreas	[E] 1-Cyano-2-hydroxy-3-butene	41.45 (8.45 - 41.45)	37.45 (± 10.26)	37.45 (35.45 - 38.45)	37.05 (± 1.14)	36.45 (35.45 - 37.45)	36.65 (± 0.84)
Pancreas	[C] Caerulin	36.95 (35.45 - 38.45)	36.85 (± 0.97)	35.45 (34.45 - 38.45)	35.65 (± 1.64)	38.45 (37.45 - 39.45)	38.25 (± 0.84)
Pancreas	[E] L-arginine	42.95 (6.95 - 46.95)	39.25 (± 11.48)	36.95 (34.95 - 37.95)	36.55 (± 1.14)	36.95 (35.95 - 36.95)	36.55 (± 0.55)
Pancreas	[B] Streptozotocin	37.45 (34.45 - 41.45)	37.85 (± 2.12)	37.45 (36.45 - 38.45)	37.45 (± 1)	37.45 (36.45 - 38.45)	37.65 (± 0.84)
Testicular	[D] 1,3-Dinitrobenzene	36.45 (33.45 - 37.45)	35.99 (± 1.29)	38.45 (36.45 - 40.45)	38.45 (± 1.41)	39.45 (36.45 - 41.45)	39.25 (± 2.28)
Testicular	[C] Cadmium chloride	37.6 (36 - 38.8)	37.55 (± 0.91)	38.6 (36.1 - 40.3)	38.6 (± 1.79)	35.9 (32.8 - 38.3)	35.72 (± 1.96)
Testicular	[D] Cadmium chloride	37.38 (33.84 - 40.27)	37.39 (± 1.86)	36.33 (35.75 - 39.81)	37.03 (± 1.7)	38.69 (37.33 - 40.19)	38.64 (± 1.12)
Testicular	[D] Carbendazim	37.45 (34.45 - 40.45)	37.05 (± 1.9)	37.45 (35.45 - 38.45)	36.85 (± 1.34)	38.45 (34.45 - 39.45)	37.85 (± 1.95)

Target organ	Toxin	24 h post dose		48 h post dose		168 h post dose	
		Median (min - max)	Mean (± SD)	Median (min - max)	Mean (± SD)	Median (min - max)	Mean (± SD)
Testicular	[D] Di-n-pentyl-phthalate	36.54 (34.57 - 38.49)	36.45 (± 1.23)	38.32 (35.18 - 40.55)	37.86 (± 2.15)	38.72 (37.89 - 39.08)	38.61 (± 0.51)
Testicular	[D] Ethane dimethane sulfonate (EDS)	36.95 (34.45 - 39.45)	36.85 (± 1.35)	38.45 (34.45 - 38.45)	37.45 (± 1.73)	36.45 (36.45 - 40.45)	38.05 (± 2.19)
Testicular	[D] Methoxyacetic acid	36.57 (34.95 - 38.92)	36.65 (± 1.11)	38.39 (36.84 - 40.89)	38.71 (± 1.62)	38.65 (37.44 - 40.49)	38.67 (± 1.3)
Multiple organ	[B] Adriamycin	38.45 (36.45 - 39.45)	38.15 (± 0.95)	37.45 (36.45 - 38.45)	37.45 (± 0.71)	37.45 (36.45 - 37.45)	37.25 (± 0.45)
Multiple organ	[C] Amphotericin B	37.95 (33.95 - 38.95)	37.05 (± 1.6)	36.95 (33.95 - 37.95)	36.55 (± 1.52)	37.95 (36.95 - 38.95)	37.75 (± 0.84)
Multiple organ	[C] Azaserine	37.45 (35.45 - 39.45)	37.45 (± 1.25)	37.45 (34.45 - 38.45)	36.65 (± 1.64)	38.45 (36.45 - 38.45)	38.05 (± 0.89)
Multiple organ	[A] Dexamethasone	37.95 (35.95 - 42.95)	38.65 (± 2.71)	36.95 (35.95 - 36.95)	36.75 (± 0.45)	39.95 (36.95 - 40.95)	39.55 (± 1.67)
Multiple organ	[E] Mitomycin-C	40.45 (37.74 - 42.45)	40.28 (± 1.63)	36.45 (34.45 - 37.45)	35.85 (± 1.34)	35.45 (35.45 - 36.45)	35.85 (± 0.55)
Physiological stressor	[C] 1,1-Dichloroethylene & maleic acid	37.95 (34.95 - 37.95)	37.15 (± 1.14)	35.95 (30.95 - 36.95)	35.35 (± 2.51)	37.95 (36.95 - 37.95)	37.75 (± 0.45)
Physiological stressor	[C] 2,4-Dinitrophenol	37.65 (34.15 - 40.15)	37.52 (± 1.73)	36.25 (31.95 - 37.85)	35.81 (± 2.29)	38.45 (37.05 - 40.45)	38.37 (± 1.32)
Physiological stressor	[B] 4-Pentenoic acid	37.95 (32.95 - 39.95)	37.95 (± 2)	36.95 (35.95 - 36.95)	36.75 (± 0.45)	36.95 (32.95 - 39.95)	36.95 (± 2.74)
Physiological stressor	[D] Acetazolamide	37.95 (35.45 - 39.45)	37.76 (± 1.14)	37.1 (36.45 - 41.45)	37.87 (± 2.04)	37.45 (35.45 - 40.45)	37.45 (± 1.87)
Physiological stressor	[C] Acivicin	37.45 (34.45 - 39.45)	37.45 (± 1.94)	35.45 (34.45 - 38.45)	36.45 (± 1.87)	38.45 (37.45 - 41.45)	38.85 (± 1.67)
Physiological stressor	[E] Ammonium chloride			37.45 (37.45 - 37.45)	37.45 (± 0)	37.45 (37.45 - 37.45)	37.45 (± 0)
Physiological stressor	[D] Carboplatin	37.45 (32.45 - 38.45)	36.75 (± 1.77)	39.45 (35.45 - 39.45)	38.25 (± 1.79)	38.45 (34.45 - 40.45)	38.25 (± 2.28)
Physiological stressor	[A] Choline and choline/methionine deficiency (chronic)	39.7 (36.45 - 49.45)	40.3 (± 3.62)	36.45 (34.45 - 38.45)	36.65 (± 1.48)	35.45 (34.45 - 37.45)	35.45 (± 1.22)
Physiological stressor	[B] Food restriction (chronic)	36.45 (34.45 - 42.45)	37.34 (± 2.37)	37.45 (37.45 - 39.45)	37.95 (± 1)	37.45 (36.45 - 39.45)	37.45 (± 1.22)
Physiological stressor	[D] Furosemide	36.45 (34.45 - 42.45)	36.72 (± 2.41)	37.45 (36.45 - 39.45)	37.65 (± 1.3)	37.45 (36.45 - 39.45)	37.85 (± 1.14)
Physiological stressor	[B] Insulin	36.95 (35.95 - 38.95)	37.35 (± 1.07)	36.95 (36.95 - 39.95)	37.75 (± 1.3)	37.95 (36.95 - 39.95)	38.55 (± 1.34)
Physiological stressor	[E] Methotrexate	38.95 (37.95 - 40.95)	39.35 (± 0.84)	35.95 (34.95 - 36.95)	35.75 (± 0.84)	35.95 (33.95 - 35.95)	35.15 (± 1.1)
Physiological stressor	[A] Partial hepatectomy	37.45 (35.45 - 38.45)	37.45 (± 1.05)	36.45 (35.45 - 38.45)	36.64 (± 1.29)	37.45 (35.45 - 38.45)	37.45 (± 1.22)

Target organ	Toxin	24 h post dose		48 h post dose		168 h post dose	
		Median (min - max)	Mean (± SD)	Median (min - max)	Mean (± SD)	Median (min - max)	Mean (± SD)
Physiological stressor	[A] Phenobarbital (chronic)	37.45 (35.45 - 39.45)	37.65 (± 1.23)	37.45 (35.45 - 39.45)	37.85 (± 1.67)	41.45 (37.45 - 41.45)	40.05 (± 1.95)
Physiological stressor	[A] Pregnenolone 16 alpha carbonitrile (chronic)						
Physiological stressor	[A] Probenecid	37.25 (36.35 - 42.35)	37.96 (± 1.8)	37.85 (33.85 - 39.85)	37.19 (± 2.22)	37.65 (36.85 - 38.65)	37.73 (± 0.73)
Physiological stressor	[C] Rosiglitazone	37.95 (35.95 - 42.95)	37.95 (± 1.94)	35.95 (35.95 - 37.95)	36.75 (± 1.1)	35.95 (33.95 - 37.95)	35.95 (± 1.41)
Physiological stressor	[C] Rosiglitazone (chronic)	37.45 (37.45 - 41.45)	38.55 (± 1.6)				
Physiological stressor	[E] Sodium bicarbonate	37.67 (37.15 - 38.14)	37.66 (± 0.32)				
Physiological stressor	[A] Unilateral nephrectomy	37.45 (33.45 - 38.45)	36.55 (± 1.73)	38.45 (34.45 - 45.45)	39.05 (± 4.04)	39.45 (37.45 - 41.45)	39.25 (± 1.48)
Physiological stressor	[B] Water deprivation (chronic)	37.52 (36.45 - 41.45)	38.06 (± 1.54)	37.95 (35.45 - 40.45)	37.8 (± 1.74)	37.45 (36.45 - 40.45)	37.63 (± 1.46)
No Effect	[E] Acetaminophen (chronic)	36.45 (36.45 - 38.45)	37.05 (± 0.89)			37.45 (36.45 - 37.45)	37.25 (± 0.45)
No Effect	[C] Buthionine sulphoxime	36.45 (34.45 - 38.45)	36.45 (± 1.33)	37.45 (37.45 - 40.45)	38.45 (± 1.41)	38.45 (37.45 - 39.45)	38.45 (± 0.71)
No Effect	[C] Ferrous sulphate	37.85 (35.65 - 39.35)	37.71 (± 1.29)	35.85 (33.75 - 38.35)	36.27 (± 2)	37.23 (36.35 - 40.15)	37.67 (± 1.47)
No Effect	[B] Ifosfamide	37.45 (33.45 - 39.45)	37.15 (± 1.57)	36.45 (35.45 - 37.45)	36.45 (± 1)	36.45 (34.45 - 37.45)	36.25 (± 1.3)
No Effect	[B] Lithocholic acid	38.45 (34.45 - 39.45)	37.85 (± 1.65)	36.45 (32.45 - 38.45)	35.85 (± 2.19)	37.45 (34.45 - 38.45)	36.85 (± 1.52)
No Effect	[E] Paraquat			37.45 (37.45 - 40.45)	38.25 (± 1.3)	37.45 (37.45 - 39.45)	38.05 (± 0.89)
No Effect	[D] Potassium dichromate	36.45 (32.45 - 40.45)	36.25 (± 2.1)	37.45 (35.45 - 39.45)	37.25 (± 1.48)	38.45 (37.45 - 39.45)	38.45 (± 0.71)
No Effect	[C] Trichlorethylene	36.9 (34.2 - 39.7)	36.74 (± 1.92)	36.3 (34.1 - 37.2)	36.04 (± 1.17)	39.6 (37.8 - 41.7)	39.5 (± 1.49)

A-F: Indicates Pharmaceutical Company & sample origin

Supplementary Table S9. Summary statistics for serum total protein (g/L) at 24 hrs, 48 hrs and 168 hrs post dose vehicle.

Target organ	Toxin	24 h post dose		48 h post dose		168 h post dose	
		Median (min - max)	Mean (± SD)	Median (min - max)	Mean (± SD)	Median (min - max)	Mean (± SD)
Liver	[E] 1,1-Dichloroethylene	64 (60.5 - 71.5)	64.7 (± 4.1)	53.5 (51.5 - 57.5)	53.9 (± 2.3)	54.5 (53.5 - 57.5)	54.7 (± 1.64)
Liver	[E] 1,2,3,4,5,6-hexachlorocyclohexane	67 (60.04 - 73)	66.4 (± 3.4)	58 (57 - 59)	57.8 (± 0.84)	57 (57 - 59)	57.6 (± 0.89)
Liver	[B] 1-Fluoropentane	58.5 (56 - 64)	59.2 (± 2.66)	60 (59 - 61)	60 (± 0.71)	59 (57 - 61)	58.8 (± 1.48)
Liver	[B] 2,4,6-Trihydroxyacetophenone (THA)	60.5 (55.5 - 64.5)	59.4 (± 2.64)	60.5 (58.5 - 61.5)	60.3 (± 1.3)	53.5 (52.5 - 57.5)	54.3 (± 2.17)
Liver	[B] 4-Amino-2,6-dichlorophenol (ADCP)	59.03 (58.21 - 59.81)	58.87 (± 0.58)	58.76 (58.52 - 58.87)	58.68 (± 0.16)	58.16 (58.05 - 58.27)	58.16 (± 0.08)
Liver	[C] Aflatoxin	59.3 (56.15 - 61.45)	59.31 (± 1.6)	58.65 (54.25 - 59.15)	57.87 (± 2.04)	60.65 (58.15 - 61.05)	59.81 (± 1.39)
Liver	[C] Allyl alcohol	59.6 (57.3 - 66.8)	60.22 (± 2.81)	57.9 (54.8 - 60.5)	58.04 (± 2.29)	58.4 (55.8 - 59.7)	57.96 (± 1.53)
Liver	[C] Allyl formate	57.15 (53.4 - 60.4)	57.28 (± 2.29)	58.86 (56.75 - 60.56)	58.55 (± 1.44)	60.8 (59 - 61.9)	60.72 (± 1.15)
Liver	[B] Azathioprine	59.03 (58.21 - 59.81)	58.87 (± 0.58)	58.76 (58.52 - 58.87)	58.68 (± 0.16)	58.16 (58.05 - 58.27)	58.16 (± 0.08)
Liver	[B] Bromobenzene	59.5 (57.5 - 64.5)	60 (± 2.12)	57.5 (52.5 - 58.5)	56.5 (± 2.35)	59.5 (56.5 - 63.5)	59.3 (± 2.68)
Liver	[C] Butylated hydroxytoluene	59 (56.3 - 62.8)	59.17 (± 1.87)	59 (56.3 - 59.9)	58.34 (± 1.52)	59.6 (53.6 - 62)	59.02 (± 3.28)
Liver	[D] Carbon tetrachloride	58.78 (56 - 63)	58.74 (± 1.96)	55 (54 - 60)	56.6 (± 2.7)	64 (58 - 65)	62.4 (± 2.88)
Liver	[C] Chlorpromazine	60.4 (56.75 - 65.45)	60.78 (± 2.65)	57.05 (55.45 - 58.25)	57.15 (± 1.14)	57.45 (57.25 - 60.05)	58.29 (± 1.31)
Liver	[B] Clofibrate	59.5 (57 - 63)	59.5 (± 1.9)	58 (57 - 62)	58.6 (± 2.07)	59 (57 - 63)	59.4 (± 2.3)
Liver	[B] Cyproterone acetate	60.5 (58 - 63)	60.6 (± 1.51)	57 (55 - 58)	56.4 (± 1.34)	59 (57 - 62)	59 (± 1.87)
Liver	[A] D-galactosamine	58.12 (57.42 - 58.62)	58.09 (± 0.46)	58 (57 - 59)	58 (± 1)	64 (58 - 71)	64.6 (± 5.41)
Liver	[B] Diethylhexylphthalate (DEHP)			59 (58 - 61)	59.4 (± 1.14)	59 (59 - 61)	59.6 (± 0.89)
Liver	[C] Dimethylformamide (DMF)	59 (56.7 - 62.3)	59.06 (± 2.12)	58.1 (57 - 61.2)	58.82 (± 1.64)	59.1 (58.2 - 62.1)	59.42 (± 1.55)
Liver	[C] Dimethylnitrosamine (DMN)	58.25 (54.75 - 60.05)	58 (± 1.73)	59.15 (57.15 - 60.85)	58.99 (± 1.32)	60.25 (56.95 - 61.95)	59.71 (± 2.11)
Liver	[A] Gadolinium chloride	58.5 (55 - 63)	58.7 (± 2.5)	61 (58 - 63)	60.2 (± 2.17)	62 (58 - 66)	62.2 (± 3.77)

Target organ	Toxin	24 h post dose		48 h post dose		168 h post dose	
		Median (min - max)	Mean (± SD)	Median (min - max)	Mean (± SD)	Median (min - max)	Mean (± SD)
Liver	[A, B, C, D, F] Hydrazine	58 (54.5 - 63.7)	58.49 (± 2.26)	59 (55.3 - 63)	58.96 (± 1.96)	59.2 (54 - 65.5)	59.79 (± 3)
Liver	[E] Hydrazine	59.65 (59.25 - 59.75)	59.53 (± 0.18)	58.55 (58.55 - 58.75)	58.61 (± 0.09)	58.65 (58.35 - 58.75)	58.57 (± 0.16)
Liver	[E] Indomethacin	62.5 (59.24 - 65)	62.32 (± 1.96)	54 (53 - 59)	55 (± 2.55)	56 (53 - 57)	55.4 (± 1.82)
Liver	[E] Ketoconazole			60.5 (56.5 - 61.5)	59.7 (± 2.17)	57.5 (55.5 - 60.5)	57.9 (± 2.07)
Liver	[C] Lead acetate	59.4 (55.15 - 62.55)	59.07 (± 2.35)	54.45 (51.65 - 56.75)	54.51 (± 1.96)	60.35 (57.15 - 63.95)	60.85 (± 2.89)
Liver	[A] Lipopolysaccharide (LPS)	55.5 (51 - 60)	55.1 (± 2.47)	60 (59 - 62)	60.2 (± 1.3)	60 (59 - 65)	60.6 (± 2.51)
Liver	[B] Methapyrilene	60 (56.5 - 61.5)	59.3 (± 1.87)	58.5 (55.5 - 62.5)	58.7 (± 2.49)	59.5 (56.5 - 60.5)	58.7 (± 2.05)
Liver	[E] Methylene dianiline	64.5 (57.5 - 67.5)	63.4 (± 3.11)	57.5 (54.5 - 59.5)	57.1 (± 1.82)	57.5 (54.5 - 58.5)	56.9 (± 1.82)
Liver	[C] Monocrotaline	58.2 (53.95 - 59.55)	57.82 (± 1.81)	59.15 (57.45 - 62.05)	59.39 (± 1.66)	62.55 (58.95 - 62.75)	61.15 (± 2.01)
Liver	[C] N-methylformamide (NMF)	59.25 (55.35 - 64.95)	59.42 (± 2.46)	57.85 (55.45 - 58.85)	57.51 (± 1.29)	60.45 (58.85 - 61.95)	60.37 (± 1.41)
Liver	[D] Phalloidin (chronic)			57.71 (57.17 - 60.64)	58.2 (± 1.39)	62.52 (57.52 - 68.15)	62.34 (± 3.97)
Liver	[E] Phenyl diisothiocyanate	63 (60.5 - 65.5)	63 (± 1.43)	53.5 (51.5 - 55.5)	53.3 (± 1.48)	56.5 (54.5 - 57.5)	56.3 (± 1.3)
Liver	[E] Phenyl isothiocyanate	61 (59 - 65)	61.7 (± 2.26)	57 (54 - 59)	56.8 (± 2.28)	54 (51 - 56)	53.8 (± 1.92)
Liver	[B] Retinyl palmitate	59.03 (58.21 - 59.81)	58.87 (± 0.58)	58.76 (58.52 - 58.87)	58.68 (± 0.16)	58.16 (58.05 - 58.27)	58.16 (± 0.08)
Liver	[B] Sodium Valproate			59 (58 - 61)	59 (± 1.22)	59 (59 - 65)	61 (± 2.83)
Liver	[C] a-Naphthylisothiocyanate (ANIT)	59.75 (55.05 - 94.35)	62.31 (± 11.42)	58.15 (55.55 - 60.05)	58.15 (± 1.76)	58.55 (47.35 - 60.95)	56.71 (± 5.43)
Kidney	[D] 2-Bromophenol	58 (56 - 59)	57.8 (± 1.03)	59 (53 - 60)	58 (± 2.83)	63 (61 - 66)	63.2 (± 1.79)
Kidney	[E] 3,5-Dichloroaniline hydrochloride	64 (60.5 - 65.5)	63.8 (± 1.7)	55.5 (53.5 - 57.5)	55.7 (± 1.79)	54.5 (52.5 - 57.5)	54.5 (± 1.87)
Kidney	[E] Atractyloside	64 (56.5 - 68.5)	63.5 (± 3.3)	57.5 (54.5 - 59.5)	57.3 (± 1.92)	53.5 (51.5 - 57.5)	54.3 (± 2.59)
Kidney	[D] Bromoethylamine hydrobromide	59.26 (57 - 62)	59.44 (± 1.58)	58 (54 - 60)	57.4 (± 2.19)	61 (59 - 63)	60.65 (± 1.62)

Target organ	Toxin	24 h post dose		48 h post dose		168 h post dose	
		Median (min - max)	Mean (± SD)	Median (min - max)	Mean (± SD)	Median (min - max)	Mean (± SD)
Kidney	[D] Cephaloridine	57.5 (55 - 60)	57.2 (± 1.87)	58.22 (57 - 62)	58.84 (± 1.9)	63 (60 - 65)	62.6 (± 2.07)
Kidney	[B] Chlorethanamine	61 (57.5 - 63.5)	60.6 (± 1.85)	57.5 (54.5 - 58.5)	57.3 (± 1.64)	58.5 (56.5 - 59.5)	58.1 (± 1.52)
Kidney	[A] Cisplatin	58.5 (56.5 - 61.5)	58.8 (± 1.77)	59.5 (56.5 - 62.5)	59.5 (± 2.55)	59.5 (56.5 - 65.5)	60.1 (± 3.36)
Kidney	[A] D-limonene (chronic)						
Kidney	[E] Dichlorophenyl succinimide	64.5 (57.5 - 67.5)	63.4 (± 3.11)	57.5 (54.5 - 59.5)	57.1 (± 1.82)	57.5 (54.5 - 58.5)	56.9 (± 1.82)
Kidney	[D] Ethylene glycol	56.5 (56 - 60)	57.42 (± 1.67)	60 (56 - 63)	59.4 (± 2.61)	62 (59 - 64)	61.8 (± 1.92)
Kidney	[A] Folic acid	59 (54 - 66)	59.05 (± 3.35)	59 (56 - 59.16)	58.23 (± 1.33)	61 (61 - 63)	61.6 (± 0.89)
Kidney	[A] Gentamicin	58 (53 - 61)	57.64 (± 2.25)	60 (58 - 62)	60.2 (± 1.79)	65 (63 - 69)	65.8 (± 2.28)
Kidney	[B] Maleic acid	59.03 (58.21 - 59.81)	58.87 (± 0.58)	58.76 (58.52 - 58.87)	58.68 (± 0.16)	58.16 (58.05 - 58.27)	58.16 (± 0.08)
Kidney	[A] N-phenylanthranilic acid (chronic)	59.3 (56 - 62)	59.03 (± 1.97)	60 (59 - 63)	60.2 (± 1.64)	58 (58 - 60)	58.6 (± 0.89)
Kidney	[D] Para-aminophenol	57 (56 - 61)	57.6 (± 1.43)	61 (60 - 63)	61.23 (± 1.27)	62 (59 - 66)	62.4 (± 3.05)
Kidney	[A] Puromycin			59 (55 - 60)	58.4 (± 2.07)	59 (56 - 63)	59 (± 2.55)
Kidney	[B] Vancomycin hydrochloride			59 (57 - 62)	59.4 (± 2.07)	59 (57 - 64)	59.6 (± 2.7)
Liver & Kidney	[E] Acetaminophen	65 (60.5 - 70.5)	65.4 (± 3.03)	56.5 (55.5 - 57.5)	56.5 (± 0.71)	55.5 (52.5 - 57.5)	55.1 (± 2.07)
Liver & Kidney	[B] Aurothiomalate	59.03 (58.21 - 59.81)	58.87 (± 0.58)	58.76 (58.52 - 58.87)	58.68 (± 0.16)	58.16 (58.05 - 58.27)	58.16 (± 0.08)
Liver & Kidney	[C] Chloroform	58.55 (55.55 - 68.35)	59.41 (± 3.63)	59.05 (57.05 - 59.65)	58.43 (± 1.23)	60.55 (56.05 - 63.75)	60.15 (± 2.87)
Liver & Kidney	[D] Cyclosporin	59 (55 - 60)	58.6 (± 1.65)	59 (56 - 60)	58.2 (± 1.64)	62 (59 - 66)	62.2 (± 2.49)
Liver & Kidney	[D] Dichlorobenzene	56 (53.5 - 59.5)	56.4 (± 2.02)	59.5 (57.5 - 61.5)	59.7 (± 1.48)	61.5 (59.5 - 64.5)	62.1 (± 1.95)
Liver & Kidney	[C] Ethionine	60.2 (57.45 - 62.75)	60.19 (± 1.49)	55.95 (54.85 - 56.45)	55.67 (± 0.73)	58.65 (55.75 - 61.05)	58.25 (± 2.16)
Liver & Kidney	[B] Hexachlorobutadiene (HCBD)			58.5 (57.5 - 60.5)	58.9 (± 1.14)	59.5 (57.5 - 63.5)	59.5 (± 2.45)

Target organ	Toxin	24 h post dose		48 h post dose		168 h post dose	
		Median (min - max)	Mean (± SD)	Median (min - max)	Mean (± SD)	Median (min - max)	Mean (± SD)
Liver & Kidney	[B] Mercuric chloride	59.5 (55.5 - 61.5)	59 (± 1.78)	57.5 (57.5 - 60.5)	58.3 (± 1.3)	59.5 (54.5 - 61.5)	58.7 (± 2.77)
Liver & Kidney	[E] Microcystin-LR			61.5 (58.5 - 62.5)	60.7 (± 2.05)	58.5 (56.5 - 59.5)	58.1 (± 1.52)
Liver & Kidney	[E] Rotenone	62.5 (61 - 66)	62.9 (± 1.66)	54 (51 - 57)	54 (± 2.55)	55 (52 - 56)	54.4 (± 1.82)
Liver & Kidney	[E] S-(1,2-dichlorovinyl)-cysteine (DCVC)	62 (59 - 64)	61.7 (± 1.64)	56 (56 - 59)	57 (± 1.41)	54 (53 - 57)	54.6 (± 1.52)
Liver & Kidney	[D] Thioacetamide	57.57 (53 - 58.3)	56.79 (± 1.67)	59.54 (59 - 61)	59.84 (± 0.93)	60 (58 - 63)	60.2 (± 1.79)
Pancreas	[E] 1-Cyano-2-hydroxy-3-butene	64.5 (5 - 68)	58.23 (± 18.92)	58 (54 - 59)	57.4 (± 2.07)	57 (56 - 59)	57.4 (± 1.14)
Pancreas	[C] Caerulin	59.15 (57.7 - 62.9)	59.47 (± 1.69)	56.4 (55.7 - 58.3)	56.92 (± 1.2)	61.3 (59.5 - 63.9)	61.24 (± 1.69)
Pancreas	[E] L-arginine	69 (4 - 80)	63.3 (± 21.22)	57 (55 - 59)	57.2 (± 1.79)	56 (54 - 57)	56 (± 1.22)
Pancreas	[B] Streptozotocin	58.5 (55 - 65)	59.6 (± 3.41)	60 (59 - 63)	60.2 (± 1.64)	59 (57 - 61)	59 (± 1.58)
Testicular	[D] 1,3-Dinitrobenzene	55.5 (50 - 61)	55.38 (± 3.15)	59 (57 - 63)	59.4 (± 2.19)	65 (59 - 68)	63.6 (± 3.91)
Testicular	[C] Cadmium chloride	59.15 (55.45 - 62.65)	59.06 (± 2.19)	61.35 (54.65 - 63.55)	60.23 (± 3.57)	57.25 (53.75 - 62.25)	57.71 (± 3.18)
Testicular	[D] Cadmium chloride	58.89 (53.51 - 63.99)	58.5 (± 2.94)	56.88 (56.38 - 62.64)	58.22 (± 2.66)	60.49 (58.76 - 61.32)	60.18 (± 1.07)
Testicular	[D] Carbendazim	58 (57 - 63)	59 (± 1.94)	59 (57 - 62)	59 (± 1.87)	62 (59 - 65)	61.8 (± 2.17)
Testicular	[D] Di-n-pentyl-phthalate	57.56 (56.36 - 60.82)	58.02 (± 1.46)	58.76 (58.05 - 63.08)	59.84 (± 2.15)	62.46 (59.75 - 66.3)	62.65 (± 2.71)
Testicular	[D] Ethane dimethane sulfonate (EDS)	58 (54 - 61)	57.8 (± 2.15)	59 (55 - 63)	58.4 (± 3.13)	60 (58 - 63)	60.4 (± 2.07)
Testicular	[D] Methoxyacetic acid	58.24 (55.25 - 60.02)	57.74 (± 1.54)	60.92 (55.81 - 64)	59.78 (± 3.62)	61.83 (60.6 - 62.88)	61.71 (± 0.99)
Multiple organ	[B] Adriamycin	60.5 (56 - 63)	60.1 (± 2.13)	59 (55 - 62)	58.6 (± 2.51)	57 (53 - 59)	56.8 (± 2.28)
Multiple organ	[C] Amphotericin B	59.8 (57.5 - 61.7)	59.72 (± 1.52)	59.8 (55 - 59.9)	58.38 (± 2.16)	57.6 (57 - 59.5)	57.84 (± 0.96)
Multiple organ	[C] Azaserine	60.2 (56.9 - 62)	59.73 (± 1.65)	59.2 (56.2 - 60.2)	58.54 (± 1.72)	58.2 (57.1 - 58.8)	58 (± 0.69)
Multiple organ	[A] Dexamethasone	58 (55 - 65)	58.9 (± 3.21)	57 (54 - 60)	57.2 (± 2.77)	62 (58 - 65)	61.8 (± 2.86)
Multiple organ	[E] Mitomycin-C	64 (60 - 66)	63.53 (± 2.42)	55 (53 - 59)	55.6 (± 2.41)	55 (54 - 56)	55 (± 0.71)

Target organ	Toxin	24 h post dose		48 h post dose		168 h post dose	
		Median (min - max)	Mean (± SD)	Median (min - max)	Mean (± SD)	Median (min - max)	Mean (± SD)
Physiological stressor	[C] 1,1-Dichloroethylene & maleic acid	59.45 (56.7 - 66.5)	60.32 (± 2.85)	58.4 (53.2 - 59.8)	57.16 (± 2.8)	58.7 (57.6 - 62.6)	59.24 (± 1.94)
Physiological stressor	[C] 2,4-Dinitrophenol	60.05 (57.9 - 63.6)	59.97 (± 1.79)	57.6 (53.8 - 58.8)	57.28 (± 2.02)	60.7 (57 - 61.8)	59.88 (± 1.9)
Physiological stressor	[B] 4-Pentenoic acid	59 (56 - 64)	59.2 (± 2.66)	57 (55 - 58)	56.8 (± 1.3)	62 (60 - 64)	62.2 (± 1.48)
Physiological stressor	[D] Acetazolamide	59 (57 - 61)	59.09 (± 1.21)	58.22 (56 - 60)	58.15 (± 1.43)	59 (55 - 62)	58.4 (± 2.88)
Physiological stressor	[C] Acivicin	59.85 (56.4 - 63)	59.99 (± 2.34)	57.3 (55.9 - 61.7)	58.7 (± 2.7)	59.2 (57.5 - 63.8)	60.18 (± 2.54)
Physiological stressor	[E] Ammonium chloride			59 (59 - 59)	59 (± 0)	59 (59 - 59)	59 (± 0)
Physiological stressor	[D] Carboplatin	58.5 (56.5 - 60.5)	58.3 (± 1.4)	59.5 (57.5 - 63.5)	60.3 (± 2.59)	60.5 (56.5 - 63.5)	60.7 (± 2.68)
Physiological stressor	[A] Choline and choline/methionine deficiency (chronic)	63.5 (57 - 68.74)	63.26 (± 4.18)	57 (56 - 62)	58.2 (± 2.39)	57 (55 - 62)	57.6 (± 2.97)
Physiological stressor	[B] Food restriction (chronic)	57 (55 - 61)	57.33 (± 2.06)	61 (59 - 64)	61.25 (± 2.22)	59 (57 - 63)	59 (± 2.45)
Physiological stressor	[D] Furosemide	57.5 (54.5 - 66.5)	58 (± 3.37)	59.5 (57.5 - 62.5)	59.5 (± 2.12)	62.5 (59.5 - 63.5)	61.9 (± 1.52)
Physiological stressor	[B] Insulin	59 (56 - 62)	59.3 (± 2.11)	59 (57 - 61)	59 (± 1.58)	59 (58 - 64)	60.4 (± 2.51)
Physiological stressor	[E] Methotrexate	61 (60.5 - 64.5)	61.7 (± 1.48)	53.5 (52.5 - 57.5)	54.1 (± 1.95)	53.5 (50.5 - 56.5)	53.3 (± 2.77)
Physiological stressor	[A] Partial hepatectomy	59 (55 - 62)	58.5 (± 2.32)	57 (55 - 58.45)	56.89 (± 1.42)	60 (57 - 64)	60.6 (± 2.61)
Physiological stressor	[A] Phenobarbital (chronic)	58.5 (54 - 60)	57.4 (± 2.22)	61 (57 - 62)	60.4 (± 2.07)	62 (59 - 66)	62.6 (± 2.88)
Physiological stressor	[A] Pregnenolone 16 alpha carbonitrile (chronic)						
Physiological stressor	[A] Probenecid	58.25 (55.5 - 65.2)	59.03 (± 2.81)	59.4 (53.7 - 61.2)	58.42 (± 3)	59.1 (58.1 - 60.4)	59.36 (± 0.98)
Physiological stressor	[C] Rosiglitazone	61.15 (58.35 - 64.65)	61.31 (± 1.98)	57.95 (56.65 - 59.05)	57.77 (± 0.98)	57.95 (54.85 - 61.35)	57.93 (± 2.34)
Physiological stressor	[C] Rosiglitazone (chronic)	59 (54.6 - 63.2)	59.04 (± 2.35)				
Physiological stressor	[E] Sodium bicarbonate	60.06 (59.4 - 60.5)	59.98 (± 0.45)				
Physiological stressor	[A] Unilateral nephrectomy	57.5 (53 - 62)	57.3 (± 2.5)	59 (55 - 70)	60.6 (± 5.59)	61 (55 - 62)	59.8 (± 2.77)

Target organ	Toxin	24 h post dose		48 h post dose		168 h post dose	
		Median (min - max)	Mean (± SD)	Median (min - max)	Mean (± SD)	Median (min - max)	Mean (± SD)
Physiological stressor	[B] Water deprivation (chronic)	59.96 (55.5 - 68.5)	60.45 (± 3.4)	59.19 (55.5 - 63.5)	59.56 (± 3.39)	57.2 (53.5 - 60.5)	57.07 (± 2.45)
No Effect	[E] Acetaminophen (chronic)	59.5 (58.5 - 60.5)	59.3 (± 0.84)			58.5 (57.5 - 62.5)	59.3 (± 2.17)
No Effect	[C] Buthionine sulphoxime	58.75 (57.5 - 60.8)	58.92 (± 1.09)	57.6 (56.7 - 60.3)	58.2 (± 1.61)	59.8 (57.5 - 61.3)	59.78 (± 1.47)
No Effect	[C] Ferrous sulphate	58.7 (55 - 60.9)	58.16 (± 2)	59 (55.5 - 62.8)	58.56 (± 2.99)	59.2 (58.1 - 63)	60.31 (± 2.15)
No Effect	[B] Ifosfamide	59 (52 - 62)	58.6 (± 2.8)	59 (55 - 59)	58.2 (± 1.79)	57 (53 - 59)	56.2 (± 2.28)
No Effect	[B] Lithocholic acid	59.5 (54 - 66)	59.5 (± 3.41)	60 (56 - 61)	59 (± 2)	59 (55 - 60)	57.6 (± 2.41)
No Effect	[E] Paraquat			59.5 (57.5 - 62.5)	59.5 (± 1.87)	58.5 (57.5 - 61.5)	59.3 (± 1.64)
No Effect	[D] Potassium dichromate	57 (55 - 66)	58.03 (± 3.24)	61 (59 - 62)	60.8 (± 1.1)	60 (56 - 65)	60.8 (± 3.7)
No Effect	[C] Trichlorethylene	58.45 (56.25 - 60.25)	58.21 (± 1.38)	59.65 (56.85 - 60.25)	58.99 (± 1.42)	60.65 (57.35 - 62.95)	60.35 (± 2.04)

A-F: Indicates Pharmaceutical Company & sample origin

Supplementary Table S10. Summary statistics for serum total bilirubin (umol/L) at 24 hrs, 48 hrs and 168 hrs post dose vehicle.

Target organ	Toxin	24 h post dose		48 h post dose		168 h post dose	
		Median (min - max)	Mean (± SD)	Median (min - max)	Mean (± SD)	Median (min - max)	Mean (± SD)
Liver	[E] 1,1-Dichloroethylene	2.91 (1.2 - 6.33)	2.91 (± 1.61)	1.2 (1.2 - 3)	1.73 (± 0.8)	1.46 (1.2 - 2.91)	1.73 (± 0.7)
Liver	[E] 1,2,3,4,5,6-hexachlorocyclohexane	1.77 (1.2 - 2.91)	1.99 (± 0.85)	1.2 (1.2 - 1.37)	1.26 (± 0.08)	1.63 (1.2 - 2.14)	1.56 (± 0.39)
Liver	[B] 1-Fluoropentane	1.2 (0.86 - 1.88)	1.27 (± 0.35)	1.88 (1.54 - 2.4)	1.88 (± 0.36)	0.86 (0.86 - 1.2)	0.93 (± 0.15)
Liver	[B] 2,4,6-Trihydroxyacetophenone (THA)	1.2 (0.86 - 1.71)	1.2 (± 0.3)	1.2 (1.03 - 1.71)	1.3 (± 0.26)	0.86 (0.86 - 1.37)	1.06 (± 0.28)
Liver	[B] 4-Amino-2,6-dichlorophenol (ADCP)	1.3 (1.09 - 1.37)	1.28 (± 0.09)	1.26 (0.88 - 1.53)	1.21 (± 0.28)	1.24 (1.02 - 1.28)	1.19 (± 0.1)
Liver	[C] Aflatoxin	1.25 (0.1 - 2.2)	1.24 (± 0.57)	1.1 (1.1 - 1.4)	1.18 (± 0.13)	1.2 (0.7 - 1.6)	1.2 (± 0.34)

Target organ	Toxin	24 h post dose		48 h post dose		168 h post dose	
		Median (min - max)	Mean (± SD)	Median (min - max)	Mean (± SD)	Median (min - max)	Mean (± SD)
Liver	[C] Allyl alcohol	1.1 (0.9 - 1.6)	1.14 (± 0.25)	0.9 (0.9 - 1.4)	1.1 (± 0.27)	1.3 (1.1 - 2.1)	1.4 (± 0.4)
Liver	[C] Allyl formate	1.2 (0.8 - 1.6)	1.17 (± 0.31)	1.2 (0.9 - 2)	1.38 (± 0.43)	1 (0.8 - 1.5)	1.08 (± 0.28)
Liver	[B] Azathioprine	1.3 (1.09 - 1.37)	1.28 (± 0.09)	1.26 (0.88 - 1.53)	1.21 (± 0.28)	1.24 (1.02 - 1.28)	1.19 (± 0.1)
Liver	[B] Bromobenzene	1.2 (1.2 - 2.06)	1.41 (± 0.3)	2.23 (2.06 - 2.4)	2.19 (± 0.14)	1.2 (1.2 - 1.2)	1.2 (± 0)
Liver	[C] Butylated hydroxytoluene	1.1 (0.6 - 1.4)	1.05 (± 0.3)	1 (0.7 - 1.6)	1.08 (± 0.34)	1.7 (1.4 - 1.9)	1.68 (± 0.23)
Liver	[D] Carbon tetrachloride	1.28 (1.11 - 1.5)	1.3 (± 0.13)	1.16 (1 - 1.24)	1.14 (± 0.09)	1.26 (1.02 - 1.54)	1.25 (± 0.22)
Liver	[C] Chlorpromazine	1.2 (0.9 - 1.4)	1.13 (± 0.21)	1.1 (0.9 - 1.4)	1.1 (± 0.21)	1.8 (0.9 - 2)	1.62 (± 0.43)
Liver	[B] Clofibrate	1.37 (0.69 - 2.06)	1.4 (± 0.35)	1.2 (0.86 - 1.54)	1.2 (± 0.24)	1.03 (0.86 - 1.2)	0.99 (± 0.14)
Liver	[B] Cyproterone acetate	1.71 (1.2 - 3.42)	2.04 (± 0.92)	1.37 (1.2 - 1.88)	1.41 (± 0.28)	1.2 (1.2 - 1.2)	1.2 (± 0)
Liver	[A] D-galactosamine	1.26 (0.9 - 1.78)	1.25 (± 0.28)	1.2 (1.2 - 2.91)	1.88 (± 0.94)	1.2 (1.2 - 2.91)	1.67 (± 0.75)
Liver	[B] Diethylhexylphthalate (DEHP)			1.46 (0.6 - 1.63)	1.25 (± 0.41)	1.11 (1.11 - 1.8)	1.29 (± 0.3)
Liver	[C] Dimethylformamide (DMF)	1 (0.7 - 1.6)	1.04 (± 0.3)	1.8 (1 - 2.3)	1.7 (± 0.54)	1.4 (0.7 - 1.9)	1.34 (± 0.46)
Liver	[C] Dimethylnitrosamine (DMN)	1.4 (0.9 - 2.2)	1.53 (± 0.41)	1.1 (0.8 - 1.7)	1.14 (± 0.35)	0.9 (0.4 - 1.2)	0.82 (± 0.36)
Liver	[A] Gadolinium chloride	1.2 (1.2 - 1.37)	1.22 (± 0.05)	1.2 (1.2 - 1.2)	1.2 (± 0)	1.2 (1.2 - 1.2)	1.2 (± 0)
Liver	[A, B, C, D, F] Hydrazine	1.26 (0.1 - 2.32)	1.3 (± 0.35)	1.2 (0.65 - 2.91)	1.23 (± 0.43)	1.2 (0.77 - 1.8)	1.21 (± 0.22)
Liver	[E] Hydrazine	1.4 (0.9 - 2.2)	1.45 (± 0.34)	1.1 (0.9 - 1.1)	1.04 (± 0.09)	1 (0.9 - 1.2)	1.04 (± 0.15)
Liver	[E] Indomethacin	2.91 (1.2 - 11.46)	3.81 (± 3.43)	1.2 (1.2 - 2.91)	1.54 (± 0.76)	1.2 (1.2 - 2.23)	1.47 (± 0.45)
Liver	[E] Ketoconazole			1.61 (1.2 - 2.91)	1.73 (± 0.7)	1.2 (1.03 - 1.2)	1.17 (± 0.08)
Liver	[C] Lead acetate	1.25 (0.55 - 2.05)	1.22 (± 0.38)	1.15 (0.85 - 1.65)	1.21 (± 0.3)	1.05 (0.75 - 1.75)	1.15 (± 0.38)
Liver	[A] Lipopolysaccharide (LPS)	1.2 (1.2 - 1.54)	1.23 (± 0.11)	1.2 (1.2 - 1.2)	1.2 (± 0)	1.2 (1.2 - 1.54)	1.3 (± 0.15)

Target organ	Toxin	24 h post dose		48 h post dose		168 h post dose	
		Median (min - max)	Mean (± SD)	Median (min - max)	Mean (± SD)	Median (min - max)	Mean (± SD)
Liver	[B] Methapyrilene	1.03 (1.03 - 1.37)	1.11 (± 0.12)	1.37 (1.2 - 1.71)	1.41 (± 0.19)	1.2 (1.03 - 1.71)	1.34 (± 0.28)
Liver	[E] Methylene dianiline	2.06 (1.2 - 8.04)	2.74 (± 2.2)	1.2 (1.2 - 2.91)	1.54 (± 0.76)	1.2 (1.2 - 2.91)	1.88 (± 0.94)
Liver	[C] Monocrotaline	1.2 (0.6 - 1.4)	1.15 (± 0.26)	1.3 (0.7 - 2.1)	1.34 (± 0.5)	1 (0.6 - 1.6)	1.06 (± 0.4)
Liver	[C] N-methylformamide (NMF)	1.25 (0.65 - 2.15)	1.24 (± 0.48)	0.65 (0.65 - 1.25)	0.77 (± 0.27)	1.55 (1.15 - 1.75)	1.51 (± 0.26)
Liver	[D] Phalloidin (chronic)			1 (0.74 - 1.23)	1.02 (± 0.19)	1.34 (1.12 - 1.74)	1.43 (± 0.27)
Liver	[E] Phenyl diisothiocyanate	2.48 (1.2 - 2.91)	2.14 (± 0.85)	1.44 (1.2 - 2.91)	1.69 (± 0.71)	2.91 (1.2 - 2.91)	2.52 (± 0.75)
Liver	[E] Phenyl isothiocyanate	2.48 (1.2 - 9.75)	3.17 (± 2.79)	1.53 (1.2 - 2.31)	1.57 (± 0.46)	1.2 (1.2 - 1.8)	1.32 (± 0.27)
Liver	[B] Retinyl palmitate	1.3 (1.09 - 1.37)	1.28 (± 0.09)	1.26 (0.88 - 1.53)	1.21 (± 0.28)	1.24 (1.02 - 1.28)	1.19 (± 0.1)
Liver	[B] Sodium Valproate			1.2 (0.52 - 1.54)	1.06 (± 0.39)	1.2 (1.2 - 1.54)	1.3 (± 0.15)
Liver	[C] a-Naphthylisothiocyanate (ANIT)	1.2 (1.2 - 2.3)	1.43 (± 0.38)	1.2 (1.2 - 1.7)	1.32 (± 0.22)	1.3 (1.2 - 1.9)	1.4 (± 0.29)
Kidney	[D] 2-Bromophenol	1.15 (1 - 1.58)	1.18 (± 0.2)	1.3 (0.9 - 1.6)	1.31 (± 0.27)	1.09 (0.8 - 1.59)	1.14 (± 0.32)
Kidney	[E] 3,5-Dichloroaniline hydrochloride	1.2 (0.34 - 3.76)	1.37 (± 1.2)	0.34 (0.34 - 2.05)	1.03 (± 0.94)	2.05 (0.34 - 2.05)	1.37 (± 0.94)
Kidney	[E] Atractyloside	1.2 (1.2 - 2.05)	1.34 (± 0.3)	1.29 (1.2 - 2.91)	1.75 (± 0.76)	1.77 (1.2 - 2.91)	2.08 (± 0.78)
Kidney	[D] Bromoethylamine hydrobromide	1.3 (1 - 1.75)	1.36 (± 0.25)	1.03 (0.85 - 1.15)	0.99 (± 0.13)	1.3 (0.85 - 1.55)	1.23 (± 0.28)
Kidney	[D] Cephaloridine	1.23 (0.8 - 1.6)	1.23 (± 0.24)	1.2 (1 - 1.7)	1.3 (± 0.27)	0.9 (0.7 - 1.42)	1.02 (± 0.32)
Kidney	[B] Chlorethanamine	0.86 (0.17 - 1.22)	0.81 (± 0.34)	1.54 (1.2 - 1.54)	1.44 (± 0.15)	2.57 (2.05 - 2.57)	2.43 (± 0.23)
Kidney	[A] Cisplatin	1.3 (0.8 - 5.5)	1.93 (± 1.43)	1.28 (1 - 1.7)	1.29 (± 0.29)	0.8 (0.7 - 1.39)	0.98 (± 0.3)
Kidney	[A] D-limonene (chronic)						
Kidney	[E] Dichlorophenyl succinimide	2.06 (1.2 - 8.04)	2.74 (± 2.2)	1.2 (1.2 - 2.91)	1.54 (± 0.76)	1.2 (1.2 - 2.91)	1.88 (± 0.94)
Kidney	[D] Ethylene glycol	1 (0 - 1.4)	0.7 (± 0.61)	1.3 (1.19 - 1.6)	1.32 (± 0.17)	1.2 (1.1 - 1.3)	1.2 (± 0.1)

Target organ	Toxin	24 h post dose		48 h post dose		168 h post dose	
		Median (min - max)	Mean (± SD)	Median (min - max)	Mean (± SD)	Median (min - max)	Mean (± SD)
Kidney	[A] Folic acid	1.19 (0.9 - 1.6)	1.21 (± 0.22)	1 (0.9 - 1.37)	1.07 (± 0.18)	1.4 (1.27 - 1.8)	1.47 (± 0.22)
Kidney	[A] Gentamicin	1.15 (0.82 - 1.5)	1.15 (± 0.18)	1.44 (1.2 - 1.55)	1.39 (± 0.16)	1.2 (1.1 - 1.41)	1.24 (± 0.12)
Kidney	[B] Maleic acid	1.3 (1.09 - 1.37)	1.28 (± 0.09)	1.26 (0.88 - 1.53)	1.21 (± 0.28)	1.24 (1.02 - 1.28)	1.19 (± 0.1)
Kidney	[A] N-phenylanthranilic acid (chronic)	1.2 (0.92 - 1.72)	1.24 (± 0.2)	1.2 (1.2 - 1.2)	1.2 (± 0)	1.2 (1.2 - 2.91)	1.54 (± 0.76)
Kidney	[D] Para-aminophenol	1.26 (0.9 - 1.7)	1.28 (± 0.22)	1.14 (0.99 - 1.31)	1.15 (± 0.12)	1.15 (1 - 1.3)	1.17 (± 0.13)
Kidney	[A] Puromycin			1.1 (1.1 - 1.2)	1.14 (± 0.05)	1.4 (1.2 - 2)	1.52 (± 0.3)
Kidney	[B] Vancomycin hydrochloride			1.03 (1.03 - 1.54)	1.17 (± 0.22)	1.2 (1.03 - 1.71)	1.34 (± 0.28)
Liver & Kidney	[E] Acetaminophen	2.06 (1.2 - 2.91)	2.06 (± 0.9)	1.2 (1.2 - 2.31)	1.42 (± 0.5)	1.2 (1.2 - 2.91)	1.88 (± 0.94)
Liver & Kidney	[B] Aurothiomalate	1.3 (1.09 - 1.37)	1.28 (± 0.09)	1.26 (0.88 - 1.53)	1.21 (± 0.28)	1.24 (1.02 - 1.28)	1.19 (± 0.1)
Liver & Kidney	[C] Chloroform	1.4 (1.05 - 2.15)	1.48 (± 0.34)	0.75 (0.45 - 1.15)	0.81 (± 0.33)	0.75 (0.35 - 1.35)	0.77 (± 0.38)
Liver & Kidney	[D] Cyclosporin	1.1 (0.8 - 1.4)	1.11 (± 0.18)	1.16 (1.07 - 1.34)	1.2 (± 0.12)	1.2 (1.17 - 1.32)	1.22 (± 0.06)
Liver & Kidney	[D] Dichlorobenzene	1.2 (1.1 - 1.5)	1.25 (± 0.14)	1.4 (1.2 - 1.8)	1.43 (± 0.25)	1.33 (1.06 - 1.88)	1.37 (± 0.31)
Liver & Kidney	[C] Ethionine	1.4 (0.6 - 2)	1.29 (± 0.44)	1.3 (0.9 - 2.3)	1.48 (± 0.57)	1 (0.7 - 1.4)	1 (± 0.27)
Liver & Kidney	[B] Hexachlorobutadiene (HCBD)			0.52 (0.34 - 0.69)	0.52 (± 0.17)	1.88 (1.71 - 1.88)	1.85 (± 0.08)
Liver & Kidney	[B] Mercuric chloride	1.54 (0.52 - 2.06)	1.39 (± 0.42)	1.03 (0.69 - 1.37)	1.03 (± 0.27)	1.03 (0.18 - 1.2)	0.76 (± 0.46)
Liver & Kidney	[E] Microcystin-LR			1.37 (1.2 - 4.62)	1.99 (± 1.48)	2.05 (1.2 - 18.3)	5.03 (± 7.44)
Liver & Kidney	[E] Rotenone	1.2 (1.2 - 2.91)	1.76 (± 0.81)	2.91 (1.2 - 6.33)	2.91 (± 2.09)	1.2 (1.2 - 2.91)	1.88 (± 0.94)
Liver & Kidney	[E] S-(1,2-dichlorovinyl)-cysteine (DCVC)	1.2 (1.2 - 4.62)	2.06 (± 1.21)	1.2 (1.2 - 2.91)	1.54 (± 0.76)	1.2 (1.11 - 2.91)	1.87 (± 0.95)
Liver & Kidney	[D] Thioacetamide	1.2 (1.02 - 1.45)	1.18 (± 0.13)	1.2 (0.99 - 1.4)	1.21 (± 0.15)	1.3 (0.9 - 2)	1.39 (± 0.4)
Pancreas	[E] 1-Cyano-2-hydroxy-3-butene	2.26 (1.2 - 4.62)	2.42 (± 1.35)	1.2 (1.02 - 1.9)	1.32 (± 0.34)	1.2 (1.2 - 2.91)	1.88 (± 0.94)

Target organ	Toxin	24 h post dose		48 h post dose		168 h post dose	
		Median (min - max)	Mean (± SD)	Median (min - max)	Mean (± SD)	Median (min - max)	Mean (± SD)
Pancreas	[C] Caerulin	1.25 (1.15 - 2.05)	1.37 (± 0.27)	1.15 (0.45 - 2.65)	1.29 (± 0.82)	0.55 (0.45 - 1.15)	0.73 (± 0.3)
Pancreas	[E] L-arginine	2.15 (1.2 - 34.34)	5.9 (± 10.17)	1.2 (1.2 - 2.91)	1.56 (± 0.75)	1.2 (1.2 - 2.91)	1.58 (± 0.75)
Pancreas	[B] Streptozotocin	1.28 (0.6 - 1.63)	1.17 (± 0.36)	1.28 (0.6 - 1.45)	1.18 (± 0.35)	0.95 (0.6 - 1.28)	0.98 (± 0.25)
Testicular	[D] 1,3-Dinitrobenzene	1.21 (1.1 - 1.37)	1.22 (± 0.07)	1.4 (1.2 - 1.51)	1.37 (± 0.13)	1.2 (1 - 1.6)	1.23 (± 0.23)
Testicular	[C] Cadmium chloride	0.95 (0.75 - 1.95)	1.2 (± 0.5)	1.05 (0.75 - 1.25)	1.05 (± 0.21)	1.85 (1.15 - 2.45)	1.81 (± 0.49)
Testicular	[D] Cadmium chloride	1.21 (0.81 - 1.56)	1.2 (± 0.21)	1.15 (1.04 - 1.67)	1.29 (± 0.28)	1.21 (0.8 - 1.4)	1.17 (± 0.22)
Testicular	[D] Carbendazim	1.12 (0.9 - 1.7)	1.17 (± 0.24)	1.32 (1.2 - 1.4)	1.32 (± 0.08)	1.23 (1.2 - 1.4)	1.26 (± 0.08)
Testicular	[D] Di-n-pentyl-phthalate	1.2 (0.72 - 1.66)	1.19 (± 0.32)	1.13 (0.79 - 1.34)	1.12 (± 0.21)	1.31 (0.89 - 1.5)	1.23 (± 0.23)
Testicular	[D] Ethane dimethane sulfonate (EDS)	1.22 (0.9 - 1.42)	1.22 (± 0.15)	1.19 (0.9 - 1.33)	1.16 (± 0.16)	1.31 (1.21 - 1.86)	1.43 (± 0.26)
Testicular	[D] Methoxyacetic acid	1.22 (0.86 - 1.83)	1.28 (± 0.28)	0.98 (0.7 - 1.39)	0.96 (± 0.28)	1.23 (1.06 - 1.34)	1.19 (± 0.12)
Multiple organ	[B] Adriamycin	1.2 (0.34 - 1.88)	1.2 (± 0.57)	1.2 (0.86 - 1.54)	1.2 (± 0.27)	1.03 (0.69 - 1.37)	1.03 (± 0.27)
Multiple organ	[C] Amphotericin B	1.25 (0.8 - 1.6)	1.26 (± 0.27)	1.3 (1.1 - 2.3)	1.54 (± 0.5)	0.7 (0.7 - 1.2)	0.86 (± 0.23)
Multiple organ	[C] Azaserine	1.2 (1.2 - 1.4)	1.24 (± 0.08)	1.4 (1.2 - 2.2)	1.64 (± 0.52)	1.5 (1.2 - 1.6)	1.44 (± 0.15)
Multiple organ	[A] Dexamethasone	1.22 (0.75 - 1.7)	1.22 (± 0.24)	1.42 (1.3 - 1.53)	1.41 (± 0.09)	1.1 (1 - 1.37)	1.16 (± 0.16)
Multiple organ	[E] Mitomycin-C	1.73 (1.2 - 3.16)	1.88 (± 0.76)	1.71 (1.2 - 2.91)	2.06 (± 0.8)	1.45 (1.2 - 1.64)	1.42 (± 0.22)
Physiological stressor	[C] 1,1-Dichloroethylene & maleic acid	1.3 (0.8 - 1.8)	1.35 (± 0.33)	1.1 (0.5 - 1.3)	1.02 (± 0.31)	1.1 (0.8 - 1.8)	1.18 (± 0.38)
Physiological stressor	[C] 2,4-Dinitrophenol	1.2 (0.8 - 1.8)	1.21 (± 0.38)	1.3 (1 - 1.9)	1.44 (± 0.39)	1.2 (0.9 - 1.5)	1.2 (± 0.22)
Physiological stressor	[B] 4-Pentenoic acid	1.11 (1.11 - 1.8)	1.25 (± 0.23)	1.29 (1.11 - 1.8)	1.35 (± 0.29)	1.29 (1.11 - 1.46)	1.29 (± 0.17)
Physiological stressor	[D] Acetazolamide	1.2 (0.65 - 2.05)	1.24 (± 0.43)	1.12 (0.65 - 1.18)	1.03 (± 0.22)	1.08 (0.99 - 1.19)	1.08 (± 0.07)
Physiological stressor	[C] Acivicin	1.45 (1.1 - 2)	1.47 (± 0.35)	1.5 (1.1 - 2.1)	1.52 (± 0.4)	1.1 (1.1 - 1.1)	1.1 (± 0)
Physiological stressor	[E] Ammonium chloride			1.2 (1.2 - 2.91)	1.66 (± 0.74)	2.91 (1.2 - 2.91)	2.38 (± 0.78)

Target organ	Toxin	24 h post dose		48 h post dose		168 h post dose	
		Median (min - max)	Mean (± SD)	Median (min - max)	Mean (± SD)	Median (min - max)	Mean (± SD)
Physiological stressor	[D] Carboplatin	1.17 (0.85 - 1.37)	1.13 (± 0.18)	1.2 (0.85 - 1.64)	1.26 (± 0.33)	1.15 (0.85 - 1.45)	1.17 (± 0.22)
Physiological stressor	[A] Choline and choline/methionine deficiency (chronic)	1.2 (1.18 - 1.2)	1.2 (± 0.01)	2.91 (1.2 - 2.91)	2.23 (± 0.94)	1.2 (1.2 - 1.2)	1.2 (± 0)
Physiological stressor	[B] Food restriction (chronic)	1.03 (1.03 - 2.74)	1.39 (± 0.58)	1.29 (1.03 - 1.37)	1.24 (± 0.16)	1.2 (1.2 - 1.54)	1.3 (± 0.15)
Physiological stressor	[D] Furosemide	1.24 (1.08 - 2.1)	1.34 (± 0.31)	1.5 (1.17 - 1.7)	1.48 (± 0.2)	0.9 (0.9 - 2.1)	1.24 (± 0.53)
Physiological stressor	[B] Insulin	1.46 (1.03 - 1.72)	1.41 (± 0.21)	0.86 (0.69 - 1.38)	0.96 (± 0.31)	0.35 (0.01 - 0.69)	0.35 (± 0.27)
Physiological stressor	[E] Methotrexate	2.06 (1.2 - 2.91)	2.06 (± 0.9)	1.2 (1.2 - 2.91)	1.54 (± 0.76)	1.2 (1.2 - 2.91)	1.8 (± 0.83)
Physiological stressor	[A] Partial hepatectomy	1.25 (0.91 - 1.44)	1.23 (± 0.13)	1.36 (1.25 - 1.71)	1.41 (± 0.18)	1.2 (1.07 - 1.64)	1.32 (± 0.25)
Physiological stressor	[A] Phenobarbital (chronic)	1.21 (1 - 1.41)	1.2 (± 0.15)	1.6 (1.1 - 1.67)	1.45 (± 0.25)	1.2 (1.2 - 1.8)	1.44 (± 0.33)
Physiological stressor	[A] Pregnenolone 16 alpha carbonitrile (chronic)						
Physiological stressor	[A] Probenecid	1.2 (1.2 - 1.2)	1.2 (± 0)	1.2 (1.2 - 1.2)	1.2 (± 0)	1.2 (1.2 - 1.2)	1.2 (± 0)
Physiological stressor	[C] Rosiglitazone	1.15 (0.8 - 1.6)	1.19 (± 0.35)	1.5 (0.8 - 1.9)	1.48 (± 0.46)	0.8 (0.8 - 1.9)	1.08 (± 0.48)
Physiological stressor	[C] Rosiglitazone (chronic)	1.2 (0.85 - 1.95)	1.35 (± 0.39)				
Physiological stressor	[E] Sodium bicarbonate	3.23 (2.35 - 3.87)	3.04 (± 0.58)				
Physiological stressor	[A] Unilateral nephrectomy	1.21 (1.09 - 1.45)	1.24 (± 0.12)	1.4 (0.69 - 1.88)	1.35 (± 0.43)	1.32 (1.04 - 1.36)	1.26 (± 0.13)
Physiological stressor	[B] Water deprivation (chronic)	1.2 (1.03 - 2.06)	1.37 (± 0.28)	1.27 (1.03 - 1.54)	1.25 (± 0.2)	1.09 (1.03 - 1.37)	1.14 (± 0.14)
No Effect	[E] Acetaminophen (chronic)	0.34 (0.34 - 3.76)	1.37 (± 1.53)			2.05 (0.34 - 3.76)	1.71 (± 1.43)
No Effect	[C] Buthionine sulphoxime	1.35 (0.75 - 1.75)	1.36 (± 0.3)	1.25 (1.05 - 2.05)	1.45 (± 0.43)	0.75 (0.75 - 1.15)	0.87 (± 0.18)
No Effect	[C] Ferrous sulphate	1.1 (0.8 - 1.9)	1.17 (± 0.4)	1.3 (1 - 1.9)	1.38 (± 0.34)	1 (0.8 - 1.3)	1.04 (± 0.24)
No Effect	[B] Ifosfamide	1.2 (1.03 - 1.71)	1.22 (± 0.19)	1.2 (1.03 - 1.88)	1.34 (± 0.33)	1.2 (1.03 - 1.54)	1.23 (± 0.22)
No Effect	[B] Lithocholic acid	1.29 (1.03 - 1.37)	1.27 (± 0.12)	1.2 (0.52 - 1.2)	0.96 (± 0.33)	1.2 (0.86 - 1.54)	1.2 (± 0.27)

Target organ	Toxin	24 h post dose		48 h post dose		168 h post dose	
		Median (min - max)	Mean (± SD)	Median (min - max)	Mean (± SD)	Median (min - max)	Mean (± SD)
No Effect	E Paraquat			1.2 (1.2 - 9.75)	3.18 (± 3.72)	1.96 (1.2 - 4.62)	2.38 (± 1.44)
No Effect	D Potassium dichromate	1.11 (0.9 - 1.85)	1.22 (± 0.31)	1.14 (1.05 - 1.35)	1.2 (± 0.14)	1.27 (1.15 - 1.34)	1.27 (± 0.08)
No Effect	C Trichlorethylene	1.25 (0.95 - 2.05)	1.3 (± 0.32)	1.35 (0.65 - 2.05)	1.27 (± 0.53)	0.55 (0.25 - 1.25)	0.63 (± 0.38)

A-F: Indicates Pharmaceutical Company & sample origin

Supplementary Table S11. Summary statistics for serum urea nitrogen (umol/L) at 24 hrs, 48 hrs and 168 hrs post dose vehicle.

Target organ	Toxin	24 h post dose		48 h post dose		168 h post dose	
		Median (min - max)	Mean (± SD)	Median (min - max)	Mean (± SD)	Median (min - max)	Mean (± SD)
Liver	E 1,1-Dichloroethylene	4643 (3929 - 6785)	4750.1 (± 842.3)	4286 (3929 - 6785)	4857.2 (± 1145.74)	5000 (5000 - 5714)	5285.6 (± 391.07)
Liver	E 1,2,3,4,5,6-hexachlorocyclohexane	5178.5 (3572 - 7499)	5230.47 (± 1122.34)	3572 (3215 - 4643)	3857.6 (± 586.61)	5357 (4643 - 6428)	5428.4 (± 638.62)
Liver	B 1-Fluoropentane	11010 (11010 - 11010)	11010 (± 0)	7092.85 (6014.52 - 7402.01)	6889.83 (± 551.33)	7168.91 (5552.12 - 7279.63)	6850.38 (± 730.54)
Liver	B 2,4,6-Trihydroxyacetophenone (THA)	5284.8 (3932 - 5534)	5007.12 (± 569.06)	4608.4 (4430.4 - 5498.4)	4878.96 (± 492.39)	4466 (4110 - 5106.8)	4594.16 (± 425.42)
Liver	B 4-Amino-2,6-dichlorophenol (ADCP)	9255.78 (7651.66 - 10003.29)	9020.21 (± 743.22)	7052.06 (7048.06 - 7284.11)	7110.35 (± 101.78)	7476.75 (7409.34 - 7476.75)	7463.27 (± 30.14)
Liver	C Aflatoxin	4930 (4232.3 - 6313.6)	4919.69 (± 582.68)	4825 (4176.3 - 5683.6)	4784.84 (± 576.74)	5469 (5021 - 5973)	5411.12 (± 390.8)
Liver	C Allyl alcohol	5070 (4275 - 5765)	5114 (± 539.89)	5025 (3545 - 6365)	5187 (± 1173.38)	4485 (4095 - 5415)	4669 (± 579.47)
Liver	C Allyl formate	4906.65 (4582.3 - 5721)	4946.79 (± 355.79)	5109.7 (4549.7 - 6108.3)	5116.2 (± 625.13)	5011.7 (4722.3 - 5469)	5102.2 (± 310.89)
Liver	B Azathioprine	9255.78 (7651.66 - 10003.29)	9020.21 (± 743.22)	7052.06 (7048.06 - 7284.11)	7110.35 (± 101.78)	7476.75 (7409.34 - 7476.75)	7463.27 (± 30.14)
Liver	B Bromobenzene	5071.2 (4038.8 - 6174.8)	4960.84 (± 641.78)	4572.8 (4038.8 - 5427.2)	4708.08 (± 536.01)	4964.4 (4750.8 - 5783.2)	5135.28 (± 455.49)
Liver	C Butylated hydroxytoluene	5102.65 (4251 - 5581)	4968.27 (± 429.98)	4969.7 (4727 - 5254.3)	4965 (± 208.38)	4983.7 (4834.3 - 5567)	5114.34 (± 316.04)
Liver	D Carbon tetrachloride	5000 (3900 - 7300)	5060 (± 972.05)	4200 (2500 - 4700)	4020 (± 875.79)	6100 (5300 - 8900)	6780 (± 1425.48)
Liver	C Chlorpromazine	5051.35 (4647.65 - 5758.35)	5122.75 (± 337.41)	4918.35 (4489.05 - 4969.65)	4782.09 (± 231.89)	5198.35 (4960.35 - 5259.05)	5133.95 (± 125.89)
Liver	B Clofibrate	5000 (3451.4 - 6691)	4878.96 (± 916.5)	4483.8 (3914.2 - 5801)	4789.96 (± 853.14)	5017.8 (4875.4 - 5338.2)	5096.12 (± 211.21)

Target organ	Toxin	24 h post dose		48 h post dose		168 h post dose	
		Median (min - max)	Mean (± SD)	Median (min - max)	Mean (± SD)	Median (min - max)	Mean (± SD)
Liver	[B] Cyproterone acetate	4928.8 (3593.8 - 5801)	4875.4 (± 727.07)	5124.6 (4875.4 - 5373.8)	5145.96 (± 199.17)	4555 (4234.6 - 5623)	4861.16 (± 628.12)
Liver	[A] D-galactosamine	5178.5 (4286 - 7499)	5321.3 (± 897.64)	4643 (3572 - 5000)	4500.2 (± 597.38)	5714 (5000 - 6428)	5785.4 (± 529.52)
Liver	[B] Diethylhexylphthalate (DEHP)			4857.6 (3326.8 - 5747.6)	4708.08 (± 902.51)	5000 (4466 - 5925.6)	5106.8 (± 525.63)
Liver	[C] Dimethylformamide (DMF)	5067.65 (4535.65 - 5291.65)	5031.25 (± 207.19)	4656.95 (4395.65 - 4852.95)	4642.05 (± 162.8)	5095.65 (4558.95 - 5417.65)	5079.79 (± 351.36)
Liver	[C] Dimethylnitrosamine (DMN)	4806.3 (4556.7 - 5429.3)	4879.59 (± 257.38)	5168 (4673.3 - 5742)	5174.52 (± 381.98)	5256.7 (4771.3 - 5443.3)	5118.54 (± 296.72)
Liver	[A] Gadolinium chloride	5400 (4700 - 6800)	5490 (± 548.63)	5000 (3900 - 5000)	4640 (± 512.84)	4700 (4300 - 5700)	4880 (± 521.54)
Liver	[A, B, C, D, F] Hydrazine	5000 (3165 - 7175)	5196.11 (± 695.31)	4644 (3215 - 6106.75)	4754.05 (± 801.34)	5000 (4235 - 6071)	5043.88 (± 453.02)
Liver	[E] Hydrazine	9000 (4000 - 16000)	9118.94 (± 3492.18)	4000 (3000 - 5000)	3970.34 (± 710.21)	5000 (4000 - 5093.14)	4618.63 (± 566.01)
Liver	[E] Indomethacin	5000 (1430 - 12497)	5321.3 (± 2798.15)	4643 (3215 - 5357)	4571.6 (± 888.92)	5000 (3929 - 5000)	4714.4 (± 465.47)
Liver	[E] Ketoconazole			5000 (4286 - 6428)	5285.6 (± 924.07)	5000 (3572 - 6071)	4928.6 (± 1022.29)
Liver	[C] Lead acetate	4979 (4831.95 - 5200.65)	4984.12 (± 108.98)	4985.95 (4743.35 - 5163.35)	4971.99 (± 150.57)	5144.65 (4747.95 - 5410.65)	5131.57 (± 240.59)
Liver	[A] Lipopolysaccharide (LPS)	5000 (3900 - 6100)	5090 (± 776.67)	5000 (4300 - 6100)	5100 (± 689.2)	4300 (3900 - 6400)	4780 (± 988.43)
Liver	[B] Methapyrilene	5391.6 (4163.4 - 6085.8)	5281.24 (± 612.3)	4911 (4163.4 - 5836.6)	4996.44 (± 604.47)	4590.6 (3736.2 - 5160.2)	4583.48 (± 538.96)
Liver	[E] Methylene dianiline	5535.5 (4286 - 6785)	5321.3 (± 779.43)	4643 (4286 - 5357)	4714.4 (± 465.47)	4643 (3929 - 6428)	5000 (± 1040.82)
Liver	[C] Monocrotaline	4892.7 (4631.4 - 5424.7)	4921.16 (± 233.04)	4850.7 (4645.4 - 5462)	5009.36 (± 391.56)	5672 (5112 - 6017.4)	5661.76 (± 354.87)
Liver	[C] N-methylformamide (NMF)	5137.65 (4073.65 - 6271.65)	5070.93 (± 581.16)	4297.65 (4223.05 - 5030.35)	4510.49 (± 358.44)	5053.65 (4932.35 - 5380.35)	5112.47 (± 187.77)
Liver	[D] Phalloidin (chronic)			4980 (4600 - 5200)	4978 (± 231.99)	5020 (4460 - 5720)	5044 (± 514.81)
Liver	[E] Phenyl diisothiocyanate	4821.5 (3929 - 6071)	4857.2 (± 587.82)	5000 (4286 - 5357)	4928.6 (± 465.47)	6071 (5000 - 6428)	5928.2 (± 597.38)
Liver	[E] Phenyl isothiocyanate	5000 (4286 - 5714)	5058.13 (± 530.49)	5357 (5000 - 6071)	5428.4 (± 465.47)	5000 (4286 - 5714)	4928.6 (± 529.52)
Liver	[B] Retinyl palmitate	9255.78 (7651.66 - 10003.29)	9020.21 (± 743.22)	7052.06 (7048.06 - 7284.11)	7110.35 (± 101.78)	7476.75 (7409.34 - 7476.75)	7463.27 (± 30.14)
Liver	[B] Sodium Valproate			4857.5 (4252.5 - 5426.5)	4900.1 (± 436.82)	5142.5 (4145.5 - 5604.5)	4964.1 (± 607.77)

Target organ	Toxin	24 h post dose		48 h post dose		168 h post dose	
		Median (min - max)	Mean (± SD)	Median (min - max)	Mean (± SD)	Median (min - max)	Mean (± SD)
Liver	[C] a-Naphthylisothiocyanate (ANIT)	5130 (4735 - 6815)	5394 (± 645.59)	4825 (2415 - 5115)	4295 (± 1140.24)	4525 (4185 - 7465)	5241 (± 1339.41)
Kidney	[D] 2-Bromophenol	4900 (3850 - 5550)	4790 (± 629.29)	4250 (4050 - 6050)	4730 (± 858.49)	5550 (4450 - 6450)	5390 (± 750.33)
Kidney	[E] 3,5-Dichloroaniline hydrochloride	5535.5 (4643 - 7499)	5571.2 (± 877.7)	5357 (4643 - 6785)	5571.2 (± 997.05)	4643 (3929 - 5000)	4500.2 (± 541.42)
Kidney	[E] Atractyloside	4803.65 (3572 - 5714)	4925.03 (± 709.71)	5357 (4286 - 6428)	5428.4 (± 773.96)	4643 (4643 - 5357)	4857.2 (± 319.31)
Kidney	[D] Bromoethylamine hydrobromide	5037 (4073.06 - 7179.09)	5115.55 (± 830.45)	5568.7 (4568.7 - 6498.7)	5584.7 (± 822.64)	4678.7 (4288.7 - 5568.7)	4820.7 (± 489.36)
Kidney	[D] Cephaloridine	4625.5 (3572 - 5000)	4575.4 (± 452.01)	5288 (5178 - 5498)	5306.22 (± 116.85)	5678 (4068 - 6388)	5440 (± 851.04)
Kidney	[B] Chlorethanamine	4840 (4198.5 - 6228.5)	5010.6 (± 697.85)	4839.5 (3771.5 - 5160.5)	4740.1 (± 564.96)	5266.5 (4305.5 - 5587.5)	5117.3 (± 499.43)
Kidney	[A] Cisplatin	5160 (3665 - 6875)	5283 (± 1028.36)	4875 (3445 - 6765)	4961 (± 1189.17)	5165 (4375 - 7265)	5303 (± 1169.11)
Kidney	[A] D-limonene (chronic)						
Kidney	[E] Dichlorophenyl succinimide	5535.5 (4286 - 6785)	5321.3 (± 779.43)	4643 (4286 - 5357)	4714.4 (± 465.47)	4643 (3929 - 6428)	5000 (± 1040.82)
Kidney	[D] Ethylene glycol	4287.09 (3680.17 - 5286.74)	4372.78 (± 493.03)	5675.98 (4505.98 - 6895.98)	5751.98 (± 935.27)	5785.98 (5105.98 - 6495.98)	5811.98 (± 504.81)
Kidney	[A] Folic acid	5233.42 (4500 - 8000)	5448.09 (± 946.46)	3390 (2210 - 5285.59)	3789.12 (± 1238.29)	5100 (4500 - 7000)	5340 (± 989.72)
Kidney	[A] Gentamicin	4290 (4000 - 5186.84)	4470.68 (± 427.23)	5570 (5000 - 6790)	5708 (± 658.61)	6180 (4680 - 6570)	5800 (± 758.98)
Kidney	[B] Maleic acid	9255.78 (7651.66 - 10003.29)	9020.21 (± 743.22)	7052.06 (7048.06 - 7284.11)	7110.35 (± 101.78)	7476.75 (7409.34 - 7476.75)	7463.27 (± 30.14)
Kidney	[A] N-phenylanthranilic acid (chronic)	4764 (3929 - 6071)	4944.07 (± 710.62)	5000 (3572 - 5714)	4714.4 (± 814.09)	5000 (4643 - 5357)	5000 (± 252.44)
Kidney	[D] Para-aminophenol	5000.93 (3715.68 - 6214.78)	4940.24 (± 625.44)	5000 (4320 - 6212.98)	5162.6 (± 701.02)	5000 (4390 - 6320)	5164 (± 743.39)
Kidney	[A] Puromycin			4910 (4480 - 5410)	4896 (± 377.07)	5090 (4590 - 6590)	5332 (± 773.25)
Kidney	[B] Vancomycin hydrochloride			4448.2 (3949.8 - 5231.4)	4597.72 (± 574.25)	5267 (4448.2 - 6548.6)	5281.24 (± 789.89)
Liver & Kidney	[E] Acetaminophen	4643 (3572 - 6071)	4607.3 (± 722.87)	5357 (5000 - 6071)	5499.8 (± 407.04)	5357 (4643 - 6071)	5499.8 (± 597.38)
Liver & Kidney	[B] Aurothiomalate	9255.78 (7651.66 - 10003.29)	9020.21 (± 743.22)	7052.06 (7048.06 - 7284.11)	7110.35 (± 101.78)	7476.75 (7409.34 - 7476.75)	7463.27 (± 30.14)
Liver & Kidney	[C] Chloroform	5130.65 (4689.7 - 5856.3)	5166.13 (± 350.13)	4918.3 (4689.7 - 5091)	4915.54 (± 145.58)	4843.7 (3877.7 - 5333.7)	4789.56 (± 556.62)

Target organ	Toxin	24 h post dose		48 h post dose		168 h post dose	
		Median (min - max)	Mean (± SD)	Median (min - max)	Mean (± SD)	Median (min - max)	Mean (± SD)
Liver & Kidney	[D] Cyclosporin	4805 (3810 - 5590)	4852 (± 518.37)	4310 (3700 - 5090)	4322 (± 550.06)	5700 (5520 - 7020)	6106 (± 700.49)
Liver & Kidney	[D] Dichlorobenzene	4301.48 (3855.21 - 5140.47)	4397.87 (± 385.91)	5749.53 (4859.53 - 5849.53)	5547.53 (± 402.02)	5749.53 (5529.53 - 8959.53)	6447.53 (± 1428.85)
Liver & Kidney	[C] Ethionine	4862.35 (4622.05 - 5289.35)	4879.15 (± 233.84)	5037.35 (3679.35 - 5088.65)	4738.67 (± 603.08)	5009.35 (4990.65 - 5326.65)	5102.67 (± 146.38)
Liver & Kidney	[B] Hexachlorobutadiene (HCBD)			5035.6 (4786.4 - 5676.4)	5113.92 (± 355.47)	4964.4 (4466 - 5569.6)	5000 (± 439.63)
Liver & Kidney	[B] Mercuric chloride	5036 (4466 - 6246)	5117.8 (± 485.17)	5000 (4217 - 6140)	5149.8 (± 753.89)	4858 (4360 - 5072)	4751.4 (± 306.25)
Liver & Kidney	[E] Microcystin-LR			3750.5 (2322.5 - 5178.5)	3893.3 (± 1145.74)	5535.5 (4821.5 - 5892.5)	5464.1 (± 391.07)
Liver & Kidney	[E] Rotenone	4643 (2322.5 - 5535.5)	4393.1 (± 1048.28)	4821.5 (4464.5 - 5178.5)	4892.9 (± 298.69)	5892.5 (5535.5 - 6249.5)	5821.1 (± 298.69)
Liver & Kidney	[E] S-(1,2-dichlorovinyl)-cysteine (DCVC)	5000 (3929 - 5714)	4964.3 (± 617.2)	5000 (4286 - 6785)	5499.8 (± 1059.03)	5000 (4643 - 5000)	4857.2 (± 195.54)
Liver & Kidney	[D] Thioacetamide	4660.5 (3429 - 5321)	4485.6 (± 577.85)	5426 (4496 - 5926)	5321.88 (± 517.97)	6426 (5106 - 7106)	6154 (± 897.09)
Pancreas	[E] 1-Cyano-2-hydroxy-3-butene	5000 (3393.5 - 5892.5)	4892.9 (± 871.22)	5178.5 (4107.5 - 5892.5)	4964.3 (± 695.92)	4821.5 (4464.5 - 6249.5)	5107.1 (± 773.96)
Pancreas	[C] Caerulin	4867 (4514.7 - 5592.7)	4922.08 (± 307.83)	4972 (4281.3 - 5438.7)	4875.88 (± 459.98)	5214.7 (4930 - 5606.7)	5235.22 (± 242.78)
Pancreas	[E] L-arginine	4821.5 (3572 - 5714)	4785.8 (± 827.88)	5000 (4643 - 5714)	5142.8 (± 407.04)	5357 (4286 - 5714)	5071.4 (± 731.63)
Pancreas	[B] Streptozotocin	4982 (3807 - 6264)	4857.6 (± 799.35)	5338 (4519 - 6513)	5580.2 (± 900.15)	4733 (4270 - 5409)	4853.8 (± 442.16)
Testicular	[D] 1,3-Dinitrobenzene	5000 (3800 - 6000)	4899.17 (± 723.3)	4800 (3100 - 5900)	4560 (± 1038.27)	5600 (5100 - 6500)	5660 (± 512.84)
Testicular	[C] Cadmium chloride	4848.3 (4302.35 - 5268.35)	4816.13 (± 299.16)	4950.95 (4638.35 - 5282.35)	4977.13 (± 266.28)	5594.95 (5160.95 - 5921.65)	5510.03 (± 316.83)
Testicular	[D] Cadmium chloride	4830 (4005 - 5765)	4849 (± 577.14)	4995 (3715 - 6065)	4969 (± 836.05)	5855 (4345 - 6225)	5639 (± 770.99)
Testicular	[D] Carbendazim	4600 (3850 - 6350)	4730 (± 695.7)	5050 (4950 - 5650)	5190 (± 279.28)	5250 (4850 - 6450)	5530 (± 653.45)
Testicular	[D] Di-n-pentyl-phthalate	4800 (4285 - 5485)	4789 (± 410.94)	4975 (4895 - 6205)	5249 (± 547.02)	5745 (3895 - 6205)	5341 (± 910.07)
Testicular	[D] Ethane dimethane sulfonate (EDS)	5000 (3600 - 5500)	4820 (± 532.92)	4600 (4100 - 6800)	4960 (± 1083.05)	5500 (4100 - 6900)	5320 (± 1068.64)
Testicular	[D] Methoxyacetic acid	4475 (3865 - 5815)	4660 (± 622.94)	5135 (4545 - 6215)	5315 (± 744.61)	6145 (5005 - 6365)	5919 (± 545.6)
Multiple organ	[B] Adriamycin	4929 (4163.4 - 6655.8)	5085.44 (± 758.54)	5516.2 (4234.6 - 5801)	5245.64 (± 629.12)	4768.6 (4127.8 - 6014.6)	4882.52 (± 702.77)
Multiple organ	[C] Amphotericin B	5039.65 (4530.95 - 5832.95)	5069.97 (± 381.86)	4918.35 (4689.65 - 5011.65)	4851.11 (± 141.96)	5230.95 (4600.95 - 5697.65)	5249.63 (± 415.9)

Target organ	Toxin	24 h post dose		48 h post dose		168 h post dose	
		Median (min - max)	Mean (± SD)	Median (min - max)	Mean (± SD)	Median (min - max)	Mean (± SD)
Multiple organ	[C] Azaserine	5102.7 (4346.65 - 5560.05)	5030.36 (± 392.68)	4976.65 (4612.65 - 5508.65)	5055.99 (± 382.7)	4902.05 (4458.65 - 5644.05)	5011.23 (± 445.29)
Multiple organ	[A] Dexamethasone	4645 (3660 - 6230)	4796 (± 779.03)	5230 (3340 - 5840)	4940 (± 950.55)	5160 (3730 - 6050)	5046 (± 966.76)
Multiple organ	[E] Mitomycin-C	5178.5 (3929 - 6071)	5190.49 (± 625.28)	4643 (4643 - 5000)	4785.8 (± 195.54)	5714 (4643 - 6071)	5357 (± 667.89)
Physiological stressor	[C] 1,1-Dichloroethylene & maleic acid	5000 (4680.35 - 5781.65)	5101.26 (± 400.25)	5235.65 (4894.95 - 5333.65)	5145.11 (± 213.18)	4894.95 (4544.95 - 5366.35)	4903.37 (± 333.83)
Physiological stressor	[C] 2,4-Dinitrophenol	4969.65 (4376.95 - 5445.65)	4969.65 (± 356.97)	5258.95 (4801.65 - 5697.65)	5187.11 (± 370.56)	4974.35 (3868.35 - 5422.35)	4814.73 (± 577.89)
Physiological stressor	[B] 4-Pentenoic acid	5089 (4359.2 - 5961.2)	5124.6 (± 485.59)	4644 (4003.2 - 5605.2)	4715.2 (± 575.69)	5000 (4110 - 6174.8)	5035.6 (± 749.72)
Physiological stressor	[D] Acetazolamide	4875.04 (3821.85 - 5714.03)	4785.79 (± 538.03)	5279.1 (4996.17 - 6106.17)	5504.76 (± 482.74)	6106.17 (4826.17 - 7496.17)	6072.17 (± 965.52)
Physiological stressor	[C] Acivicin	4974.35 (4412 - 5452.7)	4914.62 (± 307.57)	4598.7 (4136.7 - 5457.3)	4754.54 (± 519.82)	5322 (4906.7 - 5578.7)	5247.34 (± 290.74)
Physiological stressor	[E] Ammonium chloride			5000 (4286 - 6071)	4928.6 (± 731.63)	5000 (4643 - 5357)	4928.6 (± 298.69)
Physiological stressor	[D] Carboplatin	4850 (3950 - 5550)	4880 (± 522.92)	5050 (3250 - 7150)	4910 (± 1502.66)	5550 (4150 - 6150)	5190 (± 850.29)
Physiological stressor	[A] Choline and choline/methionine deficiency (chronic)	4463.5 (3571 - 5713)	4606.3 (± 661.49)	5001 (4601 - 6101)	5281 (± 605.81)	5356 (4999 - 5713)	5356 (± 252.44)
Physiological stressor	[B] Food restriction (chronic)	5071.2 (4394.8 - 5747.6)	5000 (± 436.74)	5658.6 (4679.6 - 6068)	5516.2 (± 591.79)	4359.2 (3896.4 - 5356)	4487.36 (± 583.12)
Physiological stressor	[D] Furosemide	4892.9 (4285.97 - 5285.61)	4798.51 (± 383.03)	5387.58 (4897.58 - 6887.58)	5591.58 (± 805.31)	5387.58 (4567.58 - 5677.58)	5183.58 (± 443.99)
Physiological stressor	[B] Insulin	5498.4 (4483.8 - 6228.2)	5370.24 (± 588.2)	5017.8 (4875.4 - 5124.6)	5003.56 (± 89.36)	4555 (4306 - 4591)	4505.2 (± 114.23)
Physiological stressor	[E] Methotrexate	5535.5 (3572 - 6428)	5285.6 (± 978.41)	5000 (3572 - 5714)	4643 (± 874.47)	5000 (4643 - 5714)	5071.4 (± 465.47)
Physiological stressor	[A] Partial hepatectomy	5178.5 (3929 - 5714)	4892.9 (± 694.9)	4286 (4286 - 5000)	4571.6 (± 391.07)	5357 (3929 - 6428)	5428.4 (± 957.93)
Physiological stressor	[A] Phenobarbital (chronic)	5055 (4165 - 6055)	5011 (± 564.53)	4375 (4165 - 5265)	4591 (± 464.47)	5265 (4445 - 6445)	5473 (± 858.91)
Physiological stressor	[A] Pregnenolone 16 alpha carbonitrile (chronic)						
Physiological stressor	[A] Probenecid	5000 (4286 - 5357)	4928.6 (± 328.06)	5000 (4643 - 5357)	5000 (± 357)	5714 (5000 - 5714)	5499.8 (± 319.31)
Physiological stressor	[C] Rosiglitazone	4913.65 (4549.65 - 5342.95)	4894.99 (± 233.35)	4997.65 (4554.35 - 5151.65)	4917.39 (± 263.91)	5137.65 (5006.95 - 5515.65)	5188.05 (± 204.58)
Physiological stressor	[C] Rosiglitazone (chronic)	5000 (4862.4 - 5403.7)	5097.56 (± 212.9)				

Target organ	Toxin	24 h post dose		48 h post dose		168 h post dose	
		Median (min - max)	Mean (± SD)	Median (min - max)	Mean (± SD)	Median (min - max)	Mean (± SD)
Physiological stressor	[E] Sodium bicarbonate	5200.2 (5197.7 - 5338.65)	5243.7 (± 60.25)				
Physiological stressor	[A] Unilateral nephrectomy	5000 (4286 - 6071)	5071.4 (± 526.84)	3929 (3929 - 6785)	4571.6 (± 1246.95)	5000 (5000 - 5357)	5142.8 (± 195.54)
Physiological stressor	[B] Water deprivation (chronic)	5124.6 (4127.8 - 10003.29)	5715.06 (± 1659.75)	5605.2 (4839.8 - 7355.51)	5781.22 (± 942.41)	4982.2 (3665 - 7476.75)	5130.96 (± 1325.38)
No Effect	[E] Acetaminophen (chronic)	5000 (3929 - 5714)	5000 (± 757.31)			5000 (4286 - 5357)	4928.6 (± 391.07)
No Effect	[C] Buthionine sulphoxime	4960.35 (4456.3 - 5235.7)	4884.73 (± 260.81)	5067.7 (4512.3 - 5413)	4948.2 (± 398.67)	5002.3 (4391 - 5058.3)	4846.46 (± 277.34)
No Effect	[C] Ferrous sulphate	5067.7 (4426 - 5434)	4951.01 (± 349.89)	4640.7 (4547.3 - 5144.7)	4775.06 (± 271.47)	5284.7 (4818 - 5571.96)	5246.93 (± 335.42)
No Effect	[B] Ifosfamide	5231.4 (4341.4 - 6513)	5345.32 (± 679.58)	4448.2 (4056.6 - 4982.2)	4569.24 (± 380.6)	5160.2 (4768.6 - 5623)	5145.96 (± 367.04)
No Effect	[B] Lithocholic acid	4982.2 (2917.4 - 5872.2)	4683.16 (± 969.47)	4911 (4056.6 - 5694.2)	5010.68 (± 674.71)	5302.6 (3736.2 - 5587.4)	4804.2 (± 917.35)
No Effect	[E] Paraquat			5178.5 (4821.5 - 6249.5)	5464.1 (± 586.61)	4107.5 (3036.5 - 5535.5)	4178.9 (± 1022.29)
No Effect	[D] Potassium dichromate	5000 (4100 - 7400)	5244.63 (± 944.86)	5000 (4300 - 6500)	5220 (± 858.49)	5600 (5200 - 6200)	5600 (± 393.7)
No Effect	[C] Trichlorethylene	4955.7 (4556.65 - 5364.05)	4952.41 (± 236.8)	4995.35 (4818.05 - 5177.35)	4995.35 (± 127.06)	5303.35 (5182.05 - 5378.05)	5293.09 (± 73.52)

A-F: Indicates Pharmaceutical Company & sample origin

Supplementary Table S12. Summary statistics for serum creatinine (umol/L) at 24 hrs, 48 hrs and 168 hrs post dose vehicle.

Target organ	Toxin	24 h post dose		48 h post dose		168 h post dose	
		Median (min - max)	Mean (± SD)	Median (min - max)	Mean (± SD)	Median (min - max)	Mean (± SD)
Liver	[E] 1,1-Dichloroethylene	35.36 (35.36 - 44.2)	36.24 (± 2.8)	26.52 (26.52 - 35.36)	30.06 (± 4.84)	35.36 (35.36 - 35.36)	35.36 (± 0)
Liver	[E] 1,2,3,4,5,6-hexachlorocyclohexane	35.36 (17.68 - 44.2)	35.74 (± 8.42)	26.52 (26.52 - 35.36)	30.06 (± 4.84)	35.36 (26.52 - 35.36)	33.59 (± 3.95)
Liver	[B] 1-Fluoropentane	30.23 (28.13 - 32.71)	30.3 (± 1.37)	38.01 (38.01 - 38.01)	38.01 (± 0)	38.01 (38.01 - 38.01)	38.01 (± 0)
Liver	[B] 2,4,6-Trihydroxyacetophenone (THA)	35.36 (35.36 - 35.36)	35.36 (± 0)	35.36 (35.36 - 35.36)	35.36 (± 0)	35.36 (35.36 - 35.36)	35.36 (± 0)
Liver	[B] 4-Amino-2,6-dichlorophenol (ADCP)	34.91 (34.58 - 36.3)	35.19 (± 0.55)	35.92 (35.87 - 36.78)	36.12 (± 0.38)	36.04 (35.79 - 36.19)	36.02 (± 0.14)

Target organ	Toxin	24 h post dose		48 h post dose		168 h post dose	
		Median (min - max)	Mean (± SD)	Median (min - max)	Mean (± SD)	Median (min - max)	Mean (± SD)
Liver	[C] Aflatoxin	35.36 (29.36 - 39.36)	35.06 (± 3.59)	32.36 (29.36 - 35.36)	32.56 (± 2.39)	37.36 (36.36 - 46.36)	40.16 (± 4.82)
Liver	[C] Allyl alcohol	32.86 (28.86 - 38.86)	33.56 (± 2.75)	36.86 (30.86 - 39.86)	36.66 (± 3.7)	38.86 (34.86 - 44.86)	38.86 (± 3.94)
Liver	[C] Allyl formate	33.86 (25.36 - 58.36)	36.66 (± 10.7)	37.36 (32.36 - 73.36)	49.36 (± 20.65)	35.36 (35.36 - 39.36)	36.56 (± 1.79)
Liver	[B] Azathioprine	34.91 (34.58 - 36.3)	35.19 (± 0.55)	35.92 (35.87 - 36.78)	36.12 (± 0.38)	36.04 (35.79 - 36.19)	36.02 (± 0.14)
Liver	[B] Bromobenzene	35.36 (35.36 - 35.36)	35.36 (± 0)	35.36 (35.36 - 35.36)	35.36 (± 0)	35.36 (35.36 - 35.36)	35.36 (± 0)
Liver	[C] Butylated hydroxytoluene	34.86 (32.36 - 36.36)	34.56 (± 1.62)	36.36 (30.36 - 38.36)	34.76 (± 3.29)	37.36 (33.36 - 41.36)	37.16 (± 3.03)
Liver	[D] Carbon tetrachloride	33.86 (31.36 - 35.36)	33.66 (± 1.57)	38.36 (37.36 - 41.36)	38.96 (± 1.82)	40.36 (32.36 - 43.36)	39.36 (± 4.18)
Liver	[C] Chlorpromazine	36.36 (31.36 - 43.36)	36.56 (± 3.43)	34.36 (34.36 - 36.36)	34.96 (± 0.89)	32.36 (31.36 - 37.36)	33.76 (± 2.51)
Liver	[B] Clofibrate	35.36 (35.36 - 35.36)	35.36 (± 0)	35.36 (35.36 - 35.36)	35.36 (± 0)	35.36 (26.52 - 35.36)	33.59 (± 3.95)
Liver	[B] Cyproterone acetate	35.36 (35.36 - 44.2)	37.13 (± 3.73)	35.36 (35.36 - 44.2)	37.13 (± 3.95)	35.36 (35.36 - 44.2)	38.9 (± 4.84)
Liver	[A] D-galactosamine	35.36 (35.36 - 35.36)	35.36 (± 0)	35.36 (35.36 - 44.2)	37.13 (± 3.95)	44.2 (35.36 - 53.04)	42.43 (± 7.4)
Liver	[B] Diethylhexylphthalate (DEHP)			35.36 (35.36 - 35.36)	35.36 (± 0)	35.36 (35.36 - 35.36)	35.36 (± 0)
Liver	[C] Dimethylformamide (DMF)	35.36 (33.86 - 36.86)	35.46 (± 1.17)	29.86 (28.86 - 33.86)	30.86 (± 2)	39.86 (35.86 - 42.86)	39.46 (± 3.05)
Liver	[C] Dimethylnitrosamine (DMN)	32.86 (28.36 - 37.36)	33.16 (± 3.05)	35.36 (33.36 - 36.36)	34.96 (± 1.14)	36.36 (35.36 - 37.36)	36.16 (± 0.84)
Liver	[A] Gadolinium chloride	35.36 (29.17 - 41.55)	35.54 (± 3.68)	36.24 (32.71 - 39.78)	36.6 (± 2.84)	34.48 (30.06 - 42.43)	35.54 (± 4.61)
Liver	[A, B, C, D, F] Hydrazine	35.36 (30.06 - 40.86)	35.17 (± 2.44)	35.36 (31.86 - 44.2)	36.6 (± 3.44)	35.36 (31.86 - 44.2)	37.07 (± 3.7)
Liver	[E] Hydrazine	35.51 (35.26 - 35.76)	35.54 (± 0.17)	35.36 (35.26 - 35.36)	35.34 (± 0.04)	35.36 (35.26 - 35.46)	35.34 (± 0.08)
Liver	[E] Indomethacin	44.2 (35.36 - 114.92)	48.62 (± 23.67)	35.36 (35.36 - 35.36)	35.36 (± 0)	35.36 (35.36 - 35.36)	35.36 (± 0)
Liver	[E] Ketoconazole			35.36 (35.36 - 35.36)	35.36 (± 0)	35.36 (35.36 - 44.2)	38.9 (± 4.84)
Liver	[C] Lead acetate	34.86 (31.86 - 38.86)	35.16 (± 1.89)	32.86 (31.86 - 63.86)	38.86 (± 14)	37.86 (36.86 - 42.86)	39.46 (± 2.7)

Target organ	Toxin	24 h post dose		48 h post dose		168 h post dose	
		Median (min - max)	Mean (± SD)	Median (min - max)	Mean (± SD)	Median (min - max)	Mean (± SD)
Liver	[A] Lipopolysaccharide (LPS)	33.15 (30.5 - 36.69)	33.24 (± 2.14)	36.69 (33.15 - 41.99)	37.04 (± 3.22)	37.57 (32.27 - 49.06)	39.16 (± 6.14)
Liver	[B] Methapyrilene	35.36 (26.52 - 35.36)	33.59 (± 3.73)	35.36 (35.36 - 35.36)	35.36 (± 0)	26.52 (26.52 - 35.36)	30.06 (± 4.84)
Liver	[E] Methylene dianiline	44.2 (35.36 - 53.04)	42.43 (± 5.59)	35.36 (35.36 - 44.2)	37.13 (± 3.95)	35.36 (35.36 - 44.2)	37.13 (± 3.95)
Liver	[C] Monocrotaline	35.36 (30.36 - 39.36)	35.16 (± 2.74)	33.36 (32.36 - 39.36)	34.36 (± 2.92)	40.36 (32.36 - 43.36)	39.36 (± 4.18)
Liver	[C] N-methylformamide (NMF)	34.86 (31.36 - 40.36)	35.26 (± 3.51)	31.36 (27.36 - 35.36)	31.56 (± 3.03)	37.36 (36.36 - 38.36)	37.36 (± 0.71)
Liver	[D] Phalloidin (chronic)			34.16 (32.56 - 35.26)	33.96 (± 1.13)	36.26 (35.46 - 40.06)	37 (± 1.79)
Liver	[E] Phenyl diisothiocyanate	35.36 (35.36 - 44.2)	38.9 (± 4.56)	35.36 (35.36 - 35.36)	35.36 (± 0)	44.2 (44.2 - 44.2)	44.2 (± 0)
Liver	[E] Phenyl isothiocyanate	35.36 (35.36 - 44.2)	37.13 (± 3.73)	26.52 (26.52 - 35.36)	30.06 (± 4.84)	26.52 (26.52 - 26.52)	26.52 (± 0)
Liver	[B] Retinyl palmitate	34.91 (34.58 - 36.3)	35.19 (± 0.55)	35.92 (35.87 - 36.78)	36.12 (± 0.38)	36.04 (35.79 - 36.19)	36.02 (± 0.14)
Liver	[B] Sodium Valproate			35.36 (35.36 - 35.36)	35.36 (± 0)	35.36 (35.36 - 35.36)	35.36 (± 0)
Liver	[C] a-Naphthylisothiocyanate (ANIT)	33.36 (29.36 - 36.36)	33.16 (± 2.2)	37.36 (35.36 - 39.36)	37.36 (± 1.58)	38.36 (32.36 - 47.36)	38.36 (± 5.61)
Kidney	[D] 2-Bromophenol	34.36 (32.86 - 36.86)	34.66 (± 1.48)	33.86 (30.86 - 37.86)	34.46 (± 2.88)	39.86 (36.86 - 45.86)	40.86 (± 3.54)
Kidney	[E] 3,5-Dichloroaniline hydrochloride	39.78 (26.52 - 53.04)	38.9 (± 10.38)	35.36 (26.52 - 44.2)	37.13 (± 7.4)	35.36 (26.52 - 53.04)	37.13 (± 9.68)
Kidney	[E] Atractyloside	44.2 (35.36 - 44.2)	42.43 (± 3.73)	35.36 (35.36 - 35.36)	35.36 (± 0)	35.36 (35.36 - 35.36)	35.36 (± 0)
Kidney	[D] Bromoethylamine hydrobromide	34.36 (28.36 - 36.36)	33.76 (± 2.32)	40.36 (34.36 - 45.36)	40.36 (± 4.06)	36.36 (32.36 - 40.36)	36.76 (± 2.97)
Kidney	[D] Cephaloridine	35.36 (34.36 - 37.36)	35.76 (± 1.51)	36.06 (34.36 - 37.36)	35.9 (± 1.12)	33.36 (31.36 - 35.36)	33.76 (± 1.67)
Kidney	[B] Chlorethanamine	35.36 (35.36 - 43.36)	36.16 (± 2.53)	35.36 (35.36 - 35.36)	35.36 (± 0)	35.36 (35.36 - 35.36)	35.36 (± 0)
Kidney	[A] Cisplatin	34.86 (32.36 - 42.36)	35.56 (± 2.86)	36.36 (32.36 - 42.36)	37.16 (± 3.83)	34.36 (32.36 - 36.36)	34.76 (± 1.67)
Kidney	[A] D-limonene (chronic)						
Kidney	[E] Dichlorophenyl succinimide	44.2 (35.36 - 53.04)	42.43 (± 5.59)	35.36 (35.36 - 44.2)	37.13 (± 3.95)	35.36 (35.36 - 44.2)	37.13 (± 3.95)

Target organ	Toxin	24 h post dose		48 h post dose		168 h post dose	
		Median (min - max)	Mean (± SD)	Median (min - max)	Mean (± SD)	Median (min - max)	Mean (± SD)
Kidney	[D] Ethylene glycol	32.86 (29.36 - 34.36)	32.76 (± 1.58)	40.36 (37.36 - 43.36)	40.36 (± 3)	39.36 (36.36 - 42.36)	39.76 (± 2.3)
Kidney	[A] Folic acid	35.36 (29.86 - 39.23)	34.84 (± 2.78)	35.86 (28.86 - 38.86)	34.86 (± 3.81)	36.86 (33.86 - 43.86)	37.66 (± 3.7)
Kidney	[A] Gentamicin	31.36 (26.36 - 36.19)	31.53 (± 2.73)	35.36 (33.36 - 36.36)	35.36 (± 1.22)	38.36 (35.36 - 39.36)	37.76 (± 1.52)
Kidney	[B] Maleic acid	34.91 (34.58 - 36.3)	35.19 (± 0.55)	35.92 (35.87 - 36.78)	36.12 (± 0.38)	36.04 (35.79 - 36.19)	36.02 (± 0.14)
Kidney	[A] N-phenylanthranilic acid (chronic)	34.96 (26.52 - 44.2)	34 (± 7.18)	35.36 (35.36 - 44.2)	38.9 (± 4.84)	35.36 (35.36 - 35.36)	35.36 (± 0)
Kidney	[D] Para-aminophenol	32.36 (28.36 - 35.36)	32.36 (± 2.4)	37.36 (33.36 - 40.64)	36.82 (± 2.71)	35.36 (33.36 - 45.36)	37.96 (± 4.88)
Kidney	[A] Puromycin			34.36 (31.36 - 34.36)	33.36 (± 1.41)	40.36 (36.36 - 41.36)	39.16 (± 2.59)
Kidney	[B] Vancomycin hydrochloride			35.36 (35.36 - 35.36)	35.36 (± 0)	35.36 (35.36 - 35.36)	35.36 (± 0)
Liver & Kidney	[E] Acetaminophen	35.36 (30.94 - 39.78)	35.36 (± 4.66)	39.78 (39.78 - 48.62)	43.32 (± 4.84)	30.94 (30.94 - 30.94)	30.94 (± 0)
Liver & Kidney	[B] Aurothiomalate	34.91 (34.58 - 36.3)	35.19 (± 0.55)	35.92 (35.87 - 36.78)	36.12 (± 0.38)	36.04 (35.79 - 36.19)	36.02 (± 0.14)
Liver & Kidney	[C] Chloroform	35.36 (31.36 - 42.36)	35.86 (± 2.99)	36.36 (31.36 - 39.36)	35.16 (± 3.63)	34.36 (30.36 - 39.36)	34.56 (± 3.83)
Liver & Kidney	[D] Cyclosporin	34.86 (34.36 - 38.36)	35.56 (± 1.62)	37.36 (33.36 - 39.36)	36.96 (± 2.61)	35.36 (33.36 - 38.36)	35.96 (± 1.95)
Liver & Kidney	[D] Dichlorobenzene	35.36 (31.36 - 40.36)	35.46 (± 2.73)	35.36 (33.36 - 42.36)	36.76 (± 3.97)	38.36 (33.36 - 44.36)	38.36 (± 4.06)
Liver & Kidney	[C] Ethionine	35.36 (28.36 - 37.36)	34.56 (± 2.86)	34.36 (30.36 - 36.36)	33.96 (± 2.3)	36.36 (33.36 - 38.36)	36.36 (± 1.87)
Liver & Kidney	[B] Hexachlorobutadiene (HCBD)			35.36 (35.36 - 35.36)	35.36 (± 0)	35.36 (35.36 - 44.2)	37.13 (± 3.95)
Liver & Kidney	[B] Mercuric chloride	35.36 (26.36 - 35.36)	31.76 (± 4.65)	35.36 (26.36 - 35.36)	31.76 (± 4.93)	35.36 (26.36 - 35.36)	31.76 (± 4.93)
Liver & Kidney	[E] Microcystin-LR			35.36 (35.36 - 44.2)	37.13 (± 3.95)	35.36 (35.36 - 44.2)	38.9 (± 4.84)
Liver & Kidney	[E] Rotenone	35.36 (35.36 - 44.2)	38.9 (± 4.56)	35.36 (35.36 - 44.2)	37.13 (± 3.95)	44.2 (35.36 - 44.2)	42.43 (± 3.95)
Liver & Kidney	[E] S-(1,2-dichlorovinyl)-cysteine (DCVC)	35.36 (26.52 - 44.2)	34.48 (± 5.02)	35.36 (26.52 - 35.36)	31.82 (± 4.84)	35.36 (26.52 - 35.36)	33.59 (± 3.95)
Liver & Kidney	[D] Thioacetamide	31.36 (29.86 - 34.86)	31.66 (± 1.55)	36.86 (35.86 - 41.86)	37.86 (± 2.55)	37.86 (36.86 - 37.86)	37.46 (± 0.55)

Target organ	Toxin	24 h post dose		48 h post dose		168 h post dose	
		Median (min - max)	Mean (± SD)	Median (min - max)	Mean (± SD)	Median (min - max)	Mean (± SD)
Pancreas	E 1-Cyano-2-hydroxy-3-butene	39.78 (35.36 - 53.04)	40.66 (± 6.18)	26.52 (26.52 - 35.36)	30.06 (± 4.84)	35.36 (26.52 - 53.04)	38.9 (± 10.08)
Pancreas	C Caerulin	34.36 (29.36 - 39.36)	34.26 (± 3.45)	34.36 (28.36 - 38.36)	33.76 (± 3.71)	38.36 (35.36 - 40.36)	38.16 (± 1.92)
Pancreas	E L-arginine	35.36 (0 - 44.2)	30.06 (± 11.93)	35.36 (35.36 - 35.36)	35.36 (± 0)	44.2 (44.2 - 44.2)	44.2 (± 0)
Pancreas	B Streptozotocin	35.36 (35.36 - 35.36)	35.36 (± 0)	35.36 (35.36 - 35.36)	35.36 (± 0)	35.36 (35.36 - 35.36)	35.36 (± 0)
Testicular	D 1,3-Dinitrobenzene	31.86 (29.36 - 36.61)	32.28 (± 2.29)	37.36 (35.36 - 40.36)	37.56 (± 1.92)	39.36 (37.36 - 43.36)	39.96 (± 2.41)
Testicular	C Cadmium chloride	33.36 (30.86 - 38.86)	34.06 (± 2.35)	40.86 (33.86 - 43.86)	39.06 (± 4.44)	38.86 (32.86 - 43.86)	38.06 (± 4.09)
Testicular	D Cadmium chloride	35.51 (29.91 - 38.31)	35.29 (± 2.45)	31.81 (30.61 - 35.21)	32.21 (± 1.75)	36.81 (35.51 - 40.71)	37.77 (± 2.26)
Testicular	D Carbendazim	33.86 (29.86 - 38.86)	33.66 (± 2.66)	35.86 (33.86 - 38.86)	36.06 (± 1.92)	37.86 (35.86 - 42.86)	38.26 (± 2.7)
Testicular	D Di-n-pentyl-phthalate	35.36 (33.01 - 37.31)	35.23 (± 1.2)	35.41 (34.71 - 37.81)	35.91 (± 1.32)	35.01 (32.31 - 38.41)	35.45 (± 2.3)
Testicular	D Ethane dimethane sulfonate (EDS)	33.36 (31.86 - 37.86)	34.26 (± 2.01)	32.86 (31.86 - 39.86)	34.86 (± 3.39)	39.86 (37.86 - 45.86)	40.66 (± 3.11)
Testicular	D Methoxyacetic acid	34.41 (30.21 - 37.31)	34.09 (± 2.13)	36.91 (34.51 - 41.11)	37.35 (± 2.41)	36.31 (33.41 - 39.71)	36.33 (± 2.27)
Multiple organ	B Adriamycin	35.36 (26.52 - 35.84)	34.67 (± 2.87)	35.36 (35.36 - 35.36)	35.36 (± 0)	35.36 (26.52 - 35.36)	33.59 (± 3.95)
Multiple organ	C Amphotericin B	34.86 (32.86 - 38.86)	35.56 (± 2.16)	35.86 (30.86 - 36.86)	34.46 (± 2.51)	36.86 (34.86 - 37.86)	36.46 (± 1.14)
Multiple organ	C Azaserine	35.36 (31.36 - 38.36)	34.96 (± 2.22)	36.36 (34.36 - 38.36)	36.16 (± 1.79)	39.36 (30.36 - 40.36)	37.36 (± 4.06)
Multiple organ	A Dexamethasone	32.36 (29.36 - 37.36)	32.96 (± 2.63)	36.36 (35.36 - 41.36)	37.96 (± 3.13)	39.36 (33.36 - 44.36)	38.36 (± 4.24)
Multiple organ	E Mitomycin-C	44.2 (35.36 - 53.04)	43.32 (± 6.52)	35.36 (35.36 - 44.2)	37.13 (± 3.95)	35.36 (35.36 - 44.2)	37.13 (± 3.95)
Physiological stressor	C 1,1-Dichloroethylene & maleic acid	32.36 (30.36 - 38.36)	33.36 (± 3.06)	36.36 (33.36 - 38.36)	36.16 (± 1.92)	39.36 (35.36 - 43.36)	39.36 (± 2.83)
Physiological stressor	C 2,4-Dinitrophenol	35.36 (31.36 - 37.36)	34.86 (± 2.22)	36.36 (32.36 - 37.36)	35.16 (± 2.17)	35.36 (32.36 - 37.36)	35.36 (± 1.87)
Physiological stressor	B 4-Pentenoic acid	35.36 (35.36 - 35.36)	35.36 (± 0)	35.36 (35.36 - 35.36)	35.36 (± 0)	26.52 (26.52 - 35.36)	30.06 (± 4.84)
Physiological stressor	D Acetazolamide	32.36 (29.36 - 35.36)	31.76 (± 1.78)	38.36 (36.06 - 40.36)	38.5 (± 1.88)	36.36 (35.36 - 40.36)	37.16 (± 1.92)
Physiological stressor	C Acivicin	35.36 (33.36 - 37.36)	35.16 (± 1.14)	28.36 (25.36 - 34.36)	28.96 (± 3.91)	36.36 (35.36 - 37.36)	36.36 (± 0.71)

Target organ	Toxin	24 h post dose		48 h post dose		168 h post dose	
		Median (min - max)	Mean (± SD)	Median (min - max)	Mean (± SD)	Median (min - max)	Mean (± SD)
Physiological stressor	[E] Ammonium chloride			35.36 (35.36 - 44.2)	37.13 (± 3.95)	35.36 (35.36 - 44.2)	37.13 (± 3.95)
Physiological stressor	[D] Carboplatin	32.36 (29.36 - 35.36)	32.86 (± 2.01)	41.36 (35.36 - 46.36)	41.56 (± 4.21)	41.36 (39.36 - 41.36)	40.76 (± 0.89)
Physiological stressor	[A] Choline and choline/methionine deficiency (chronic)	35.36 (35.36 - 35.36)	35.36 (± 0)	44.2 (35.36 - 44.2)	42.43 (± 3.95)	35.36 (35.36 - 35.36)	35.36 (± 0)
Physiological stressor	[B] Food restriction (chronic)	35.36 (26.52 - 35.36)	34.38 (± 2.95)	35.36 (35.36 - 35.36)	35.36 (± 0)	35.36 (35.36 - 35.36)	35.36 (± 0)
Physiological stressor	[D] Furosemide	32.36 (29.36 - 34.36)	32.36 (± 1.63)	36.36 (36.36 - 38.36)	36.96 (± 0.89)	40.36 (40.36 - 46.36)	41.76 (± 2.61)
Physiological stressor	[B] Insulin	31.1 (26.52 - 35.36)	30.97 (± 4.63)	35.84 (35.84 - 35.84)	35.84 (± 0)	35.84 (26.84 - 35.84)	34.04 (± 4.02)
Physiological stressor	[E] Methotrexate	35.36 (35.36 - 44.2)	36.24 (± 2.8)	26.52 (17.68 - 26.52)	22.98 (± 4.84)	26.52 (17.68 - 35.36)	26.52 (± 6.25)
Physiological stressor	[A] Partial hepatectomy	37.13 (34.03 - 43.76)	37.75 (± 2.91)	34.03 (30.5 - 34.03)	33.15 (± 1.53)	34.03 (33.15 - 41.11)	35.8 (± 3.25)
Physiological stressor	[A] Phenobarbital (chronic)	34.36 (30.86 - 35.86)	34.26 (± 1.43)	35.86 (34.86 - 38.86)	36.26 (± 1.67)	42.86 (40.86 - 45.86)	43.26 (± 1.82)
Physiological stressor	[A] Pregnenolone 16 alpha carbonitrile (chronic)						
Physiological stressor	[A] Probenecid	35.36 (26.52 - 44.2)	34.48 (± 5.02)	35.36 (35.36 - 44.2)	38.9 (± 4.84)	44.2 (44.2 - 44.2)	44.2 (± 0)
Physiological stressor	[C] Rosiglitazone	35.36 (33.36 - 39.36)	35.56 (± 1.75)	35.36 (33.36 - 39.36)	35.76 (± 2.3)	36.36 (35.36 - 38.36)	36.56 (± 1.3)
Physiological stressor	[C] Rosiglitazone (chronic)	35.36 (33.36 - 35.36)	34.66 (± 0.95)				
Physiological stressor	[E] Sodium bicarbonate	42.44 (41.76 - 42.68)	42.3 (± 0.31)				
Physiological stressor	[A] Unilateral nephrectomy	32.71 (28.73 - 37.57)	33.06 (± 2.58)	36.69 (33.15 - 40.22)	36.51 (± 2.96)	39.34 (37.57 - 41.99)	39.51 (± 1.7)
Physiological stressor	[B] Water deprivation (chronic)	35.36 (34.48 - 44.2)	36.84 (± 3.46)	35.36 (35.36 - 44.2)	37.07 (± 3.54)	35.36 (35.36 - 44.2)	36.94 (± 3.57)
No Effect	[E] Acetaminophen (chronic)	35.36 (35.36 - 44.2)	37.13 (± 3.95)			35.36 (35.36 - 44.2)	38.9 (± 4.84)
No Effect	[C] Buthionine sulphoximе	35.36 (30.86 - 40.86)	35.46 (± 3.06)	35.86 (34.86 - 38.86)	36.46 (± 1.82)	32.86 (26.86 - 37.86)	32.66 (± 4.44)
No Effect	[C] Ferrous sulphate	34.86 (30.36 - 36.36)	34.06 (± 1.89)	35.36 (33.36 - 50.36)	38.16 (± 6.91)	38.36 (36.36 - 38.77)	38.04 (± 0.96)

Target organ	Toxin	24 h post dose		48 h post dose		168 h post dose	
		Median (min - max)	Mean (± SD)	Median (min - max)	Mean (± SD)	Median (min - max)	Mean (± SD)
No Effect	[B] Ifosfamide	35.36 (35.36 - 35.36)	35.36 (± 0)	35.36 (35.36 - 35.36)	35.36 (± 0)	35.36 (26.52 - 35.36)	33.59 (± 3.95)
No Effect	[B] Lithocholic acid	35.36 (35.36 - 35.36)	35.36 (± 0)	35.36 (35.36 - 35.36)	35.36 (± 0)	35.36 (35.36 - 35.36)	35.36 (± 0)
No Effect	[E] Paraquat			39.78 (39.78 - 48.62)	41.55 (± 3.95)	30.94 (22.1 - 30.94)	27.4 (± 4.84)
No Effect	[D] Potassium dichromate	35.36 (31.36 - 37.36)	34.81 (± 1.81)	35.36 (34.36 - 40.36)	36.36 (± 2.55)	42.36 (36.36 - 46.36)	41.56 (± 4.15)
No Effect	[C] Trichlorethylene	34.36 (31.86 - 36.86)	34.36 (± 1.84)	34.86 (32.86 - 38.86)	35.86 (± 2.45)	38.86 (37.86 - 43.86)	39.66 (± 2.49)

A-F: Indicates Pharmaceutical Company & sample origin

Supplementary Table S13. Summary statistics for serum alanine aminotransferase (IU/L) at 24 hrs, 48 hrs and 168 hrs post dose vehicle.

Target organ	Toxin	24 h post dose		48 h post dose		168 h post dose	
		Median (min - max)	Mean (± SD)	Median (min - max)	Mean (± SD)	Median (min - max)	Mean (± SD)
Liver	[E] 1,1-Dichloroethylene	53.5 (45.5 - 60.5)	53.6 (± 4.79)	46.5 (44.5 - 50.5)	47.5 (± 2.45)	40.5 (35.5 - 55.5)	42.9 (± 8.26)
Liver	[E] 1,2,3,4,5,6-hexachlorocyclohexane	53 (45 - 70)	53.9 (± 6.87)	45 (42 - 50)	46 (± 3.08)	47 (45 - 50)	47.8 (± 2.17)
Liver	[B] 1-Fluoropentane	43.09 (36.98 - 47)	42.58 (± 3.33)	52.48 (49.45 - 55.49)	52.28 (± 2.61)	54.9 (49.39 - 56.95)	53.94 (± 3.06)
Liver	[B] 2,4,6-Trihydroxyacetophenone (THA)	48.5 (40 - 58)	49 (± 5.68)	51 (48 - 52)	50.8 (± 1.64)	51 (35 - 52)	47.4 (± 7.09)
Liver	[B] 4-Amino-2,6-dichlorophenol (ADCP)	50.43 (45.21 - 52.9)	50.03 (± 2.41)	51.3 (49.16 - 55.54)	51.63 (± 2.35)	47.51 (47.51 - 50.56)	48.66 (± 1.58)
Liver	[C] Aflatoxin	50.3 (43.4 - 65)	51.5 (± 6.63)	53 (42.2 - 57.8)	50.96 (± 7.19)	48.2 (41 - 66.8)	50.36 (± 9.74)
Liver	[C] Allyl alcohol	51.5 (45.5 - 67.7)	54.56 (± 8.87)	54.5 (47.3 - 65.3)	54.98 (± 7.52)	48.5 (43.1 - 50.9)	47.66 (± 3.3)
Liver	[C] Allyl formate	50 (46.1 - 63.5)	52.7 (± 6.37)	50.9 (43.7 - 67.1)	52.58 (± 9.56)	49.1 (47.3 - 71.3)	55.34 (± 10.57)
Liver	[B] Azathioprine	50.43 (45.21 - 52.9)	50.03 (± 2.41)	51.3 (49.16 - 55.54)	51.63 (± 2.35)	47.51 (47.51 - 50.56)	48.66 (± 1.58)
Liver	[B] Bromobenzene	49.5 (44 - 55)	49.9 (± 3.81)	54 (38 - 115)	63 (± 30.29)	50 (41 - 54)	49.6 (± 5.32)
Liver	[C] Butylated hydroxytoluene	50 (43.4 - 62)	50.66 (± 5.44)	55.4 (45.8 - 58.4)	52.88 (± 6.04)	47.6 (45.8 - 66.8)	51.92 (± 8.61)

Target organ	Toxin	24 h post dose		48 h post dose		168 h post dose	
		Median (min - max)	Mean (± SD)	Median (min - max)	Mean (± SD)	Median (min - max)	Mean (± SD)
Liver	[D] Carbon tetrachloride	49.5 (35 - 68)	49.7 (± 9.52)	50 (39 - 60)	50.4 (± 9.18)	58 (45 - 65)	55.8 (± 9.36)
Liver	[C] Chlorpromazine	50.9 (28.1 - 62.3)	49.46 (± 9.05)	49.1 (44.3 - 52.7)	48.98 (± 3.19)	47.9 (42.5 - 58.7)	50.9 (± 6.95)
Liver	[B] Clofibrate	49 (39.5 - 56.5)	48.4 (± 5.47)	52.5 (44.5 - 56.5)	51.3 (± 4.55)	47.5 (39.5 - 60.5)	49.1 (± 7.89)
Liver	[B] Cyproterone acetate	49 (34.5 - 62.5)	49.7 (± 10.28)	43.5 (40.5 - 63.5)	48.5 (± 9.85)	52.5 (46.5 - 53.5)	51.1 (± 2.79)
Liver	[A] D-galactosamine	62.5 (37.5 - 74.5)	59.7 (± 10.55)	37.5 (25.5 - 46.5)	35.7 (± 8.32)	26.5 (16.5 - 61.5)	33.5 (± 17.97)
Liver	[B] Diethylhexylphthalate (DEHP)			50.5 (48.5 - 55.5)	51.5 (± 2.92)	49.5 (46.5 - 56.5)	51.3 (± 4.44)
Liver	[C] Dimethylformamide (DMF)	48.8 (32.3 - 65.3)	50.12 (± 10.32)	58.7 (50.9 - 67.7)	57.86 (± 6.47)	45.5 (38.3 - 71.9)	49.34 (± 13.16)
Liver	[C] Dimethylnitrosamine (DMN)	49.4 (40.4 - 58.4)	48.56 (± 5.64)	50 (44.6 - 53)	49.52 (± 3.16)	50.6 (38.6 - 64.4)	50.84 (± 12.05)
Liver	[A] Gadolinium chloride	50 (31 - 61)	48 (± 9.82)	47 (36 - 75)	49.8 (± 15.25)	55 (38 - 57)	49.2 (± 9.36)
Liver	[A, B, C, D, F] Hydrazine	50 (34.4 - 65)	50.33 (± 6.82)	49.5 (34.5 - 60)	48.78 (± 6.86)	50 (36 - 65)	50.74 (± 7.47)
Liver	[E] Hydrazine	53 (46 - 62)	53.4 (± 5.8)	44 (37 - 73)	48.4 (± 14.15)	51 (41 - 81)	54.6 (± 16.58)
Liver	[E] Indomethacin	52.5 (48.5 - 62.5)	53.3 (± 4.34)	46.5 (41.5 - 51.5)	46.7 (± 3.7)	48.5 (43.5 - 78.5)	54.7 (± 13.99)
Liver	[E] Ketoconazole			51 (49 - 58)	51.8 (± 3.56)	48 (41 - 52)	47 (± 4.47)
Liver	[C] Lead acetate	51.5 (46.7 - 56.3)	51.2 (± 2.63)	49.7 (48.5 - 59.9)	51.38 (± 4.79)	47.9 (45.5 - 56.3)	49.94 (± 4.64)
Liver	[A] Lipopolysaccharide (LPS)	53 (40.5 - 76.5)	53.6 (± 10.28)	52.5 (41.5 - 103.5)	65.1 (± 26.82)	46.5 (35.5 - 57.5)	45.5 (± 8.28)
Liver	[B] Methapyrilene	50 (36.5 - 57.5)	49.9 (± 6.47)	51.5 (47.5 - 69.5)	53.5 (± 9.17)	49.5 (43.5 - 64.5)	51.5 (± 7.84)
Liver	[E] Methylene dianiline	53 (43.5 - 68.5)	54.1 (± 7.06)	49.5 (42.5 - 50.5)	47.1 (± 3.78)	40.5 (40.5 - 50.5)	43.7 (± 4.6)
Liver	[C] Monocrotaline	48.2 (42.2 - 62)	50 (± 6.68)	51.2 (44.6 - 58.4)	51.68 (± 6.08)	50.6 (45.8 - 65)	53.84 (± 8.83)
Liver	[C] N-methylformamide (NMF)	51.2 (33.5 - 58.1)	46.82 (± 9.46)	51.5 (45.5 - 55.1)	51.02 (± 3.48)	47.3 (41.3 - 51.5)	46.46 (± 3.86)
Liver	[D] Phalloidin (chronic)			40.4 (28.3 - 48.3)	39.04 (± 7.43)	57.5 (51.7 - 59.4)	56.38 (± 3.03)

Target organ	Toxin	24 h post dose		48 h post dose		168 h post dose	
		Median (min - max)	Mean (± SD)	Median (min - max)	Mean (± SD)	Median (min - max)	Mean (± SD)
Liver	[E] Phenyl diisothiocyanate	52.5 (44.5 - 59.5)	52.6 (± 3.93)	49.5 (44.5 - 52.5)	48.5 (± 3.39)	44.5 (38.5 - 46.5)	43.5 (± 3)
Liver	[E] Phenyl isothiocyanate	56.5 (48.5 - 62.5)	55 (± 5.52)	47.5 (44.5 - 61.5)	49.5 (± 7.04)	46.5 (42.5 - 52.5)	47.5 (± 4.36)
Liver	[B] Retinyl palmitate	50.43 (45.21 - 52.9)	50.03 (± 2.41)	51.3 (49.16 - 55.54)	51.63 (± 2.35)	47.51 (47.51 - 50.56)	48.66 (± 1.58)
Liver	[B] Sodium Valproate			46 (38 - 63)	47.8 (± 9.83)	52 (48 - 67)	53.8 (± 7.66)
Liver	[C] a-Naphthylisothiocyanate (ANIT)	50 (39.8 - 54.8)	49.64 (± 5.08)	55.4 (47.6 - 70.4)	57.68 (± 9.54)	42.8 (35.6 - 54.8)	43.4 (± 7.07)
Kidney	[D] 2-Bromophenol	49.5 (40.5 - 70.5)	52.2 (± 9.87)	54.5 (45.5 - 76.5)	57.9 (± 13.01)	49.5 (38.5 - 54.5)	47.9 (± 7.2)
Kidney	[E] 3,5-Dichloroaniline hydrochloride	53.5 (40 - 69)	54.7 (± 8.11)	47 (41 - 59)	48.4 (± 7.6)	46 (40 - 49)	45.4 (± 3.36)
Kidney	[E] Atractyloside	55.5 (46.5 - 64.5)	55 (± 5.93)	47.5 (45.5 - 55.5)	48.9 (± 3.85)	46.5 (35.5 - 55.5)	45.9 (± 7.77)
Kidney	[D] Bromoethylamine hydrobromide	53 (45 - 60)	52.9 (± 5.02)	50 (41 - 61)	49.8 (± 7.26)	41 (39 - 46)	42 (± 2.65)
Kidney	[D] Cephaloridine	51.5 (44 - 61)	52.6 (± 5.42)	45 (43 - 57)	49.05 (± 6.53)	47 (45 - 58)	49.8 (± 5.26)
Kidney	[B] Chlorethanamine	48.5 (37 - 58)	48 (± 7.8)	53 (41 - 56)	49.4 (± 7.3)	50 (41 - 57)	49.6 (± 6.69)
Kidney	[A] Cisplatin	46 (34 - 66)	47.4 (± 8.87)	53 (49 - 84)	60.4 (± 14.36)	42 (35 - 69)	50.2 (± 15.22)
Kidney	[A] D-limonene (chronic)						
Kidney	[E] Dichlorophenyl succinimide	53 (43.5 - 68.5)	54.1 (± 7.06)	49.5 (42.5 - 50.5)	47.1 (± 3.78)	40.5 (40.5 - 50.5)	43.7 (± 4.6)
Kidney	[D] Ethylene glycol	46 (35.5 - 70.5)	47.6 (± 10.46)	49.5 (44.5 - 62.5)	52.1 (± 7.64)	58.5 (50.5 - 64.5)	58.3 (± 5.02)
Kidney	[A] Folic acid	54.38 (43 - 60)	52.68 (± 5.15)	51 (46 - 56.14)	51.03 (± 3.85)	48 (45 - 64)	51.2 (± 7.79)
Kidney	[A] Gentamicin	49.5 (38 - 57.5)	48.65 (± 6.89)	49 (47 - 53)	49.6 (± 2.41)	52 (40 - 68)	54.2 (± 10.31)
Kidney	[B] Maleic acid	50.43 (45.21 - 52.9)	50.03 (± 2.41)	51.3 (49.16 - 55.54)	51.63 (± 2.35)	47.51 (47.51 - 50.56)	48.66 (± 1.58)
Kidney	[A] N-phenylanthranilic acid (chronic)	54.06 (42 - 71)	53.99 (± 8.53)	49 (44 - 56)	50.4 (± 4.72)	47 (39 - 78)	51.8 (± 15.42)
Kidney	[D] Para-aminophenol	49 (36 - 59)	47.1 (± 6.98)	53 (45 - 57.03)	52.01 (± 4.37)	50 (41 - 55)	47.6 (± 5.94)

Target organ	Toxin	24 h post dose		48 h post dose		168 h post dose	
		Median (min - max)	Mean (± SD)	Median (min - max)	Mean (± SD)	Median (min - max)	Mean (± SD)
Kidney	[A] Puromycin			52.5 (44.5 - 55.5)	51.3 (± 4.21)	48.5 (43.5 - 57.5)	49.3 (± 5.12)
Kidney	[B] Vancomycin hydrochloride			44 (42 - 56)	47.2 (± 5.76)	51 (42 - 60)	51.6 (± 6.66)
Liver & Kidney	[E] Acetaminophen	56.5 (43 - 107)	59.5 (± 17.88)	50 (42 - 52)	47.2 (± 4.82)	45 (36 - 50)	43.4 (± 5.46)
Liver & Kidney	[B] Aurothiomalate	50.43 (45.21 - 52.9)	50.03 (± 2.41)	51.3 (49.16 - 55.54)	51.63 (± 2.35)	47.51 (47.51 - 50.56)	48.66 (± 1.58)
Liver & Kidney	[C] Chloroform	48.8 (36.5 - 56.9)	47.6 (± 6.65)	54.5 (43.7 - 64.1)	54.86 (± 7.37)	50.9 (46.1 - 53.9)	50.18 (± 3.38)
Liver & Kidney	[D] Cyclosporin	54.5 (43.5 - 64.5)	55.1 (± 5.68)	44.5 (41.5 - 49.5)	44.7 (± 3.11)	39.5 (34.5 - 50.5)	40.9 (± 6.66)
Liver & Kidney	[D] Dichlorobenzene	51 (40.5 - 60.5)	50.7 (± 5.57)	53.5 (38.5 - 62.5)	52.5 (± 8.75)	38.5 (30.5 - 46.5)	38.5 (± 6.32)
Liver & Kidney	[C] Ethionine	49.7 (44.6 - 66.8)	50.84 (± 6.75)	49.4 (39.2 - 57.8)	49.64 (± 7.81)	54.2 (39.2 - 55.4)	50.6 (± 6.8)
Liver & Kidney	[B] Hexachlorobutadiene (HCBD)			55 (47 - 71)	56.8 (± 9.07)	47 (41 - 50)	46.2 (± 4.09)
Liver & Kidney	[B] Mercuric chloride	50 (43 - 66)	50.1 (± 6.52)	52 (46 - 58)	52.4 (± 5.55)	49 (43 - 61)	51 (± 7.07)
Liver & Kidney	[E] Microcystin-LR			51 (46 - 58)	52.2 (± 5.17)	47 (41 - 59)	48.6 (± 7.09)
Liver & Kidney	[E] Rotenone	55.5 (51.5 - 61.5)	56.2 (± 4.19)	45.5 (41.5 - 47.5)	44.9 (± 2.41)	43.5 (42.5 - 48.5)	44.3 (± 2.49)
Liver & Kidney	[E] S-(1,2-dichlorovinyl)-cysteine (DCVC)	54.5 (49 - 64)	54.6 (± 4.55)	49 (46 - 61)	50.8 (± 5.89)	47 (41 - 50)	46.6 (± 3.51)
Liver & Kidney	[D] Thioacetamide	49 (43.5 - 63.5)	51.2 (± 7.13)	57.5 (44.5 - 82.5)	60.9 (± 14.43)	47.5 (45.5 - 58.5)	49.5 (± 5.43)
Pancreas	[E] 1-Cyano-2-hydroxy-3-butene	50.5 (14.5 - 60.5)	48.4 (± 12.52)	51.5 (47.5 - 74.5)	55.3 (± 11.08)	46.5 (43.5 - 60.5)	48.7 (± 7.05)
Pancreas	[C] Caerulin	51.2 (38.9 - 58.1)	49.52 (± 7.17)	49.1 (43.7 - 54.5)	49.1 (± 4.18)	49.1 (44.9 - 58.7)	51.86 (± 5.96)
Pancreas	[E] L-arginine	51 (42 - 67)	51.4 (± 7.63)	42 (36 - 54)	44.2 (± 7.56)	49 (37 - 61)	49.8 (± 9.88)
Pancreas	[B] Streptozotocin	48.5 (39 - 66)	50 (± 8.46)	52 (40 - 67)	53.4 (± 10.21)	51 (45 - 54)	49.8 (± 3.7)
Testicular	[D] 1,3-Dinitrobenzene	49 (31 - 61)	47.36 (± 8.66)	51 (41 - 76)	55.2 (± 13.72)	52 (41 - 75)	54.6 (± 12.66)
Testicular	[C] Cadmium chloride	48.5 (34.4 - 67.4)	49.22 (± 10.49)	52.4 (45.2 - 68.6)	55.4 (± 9.48)	46.4 (35 - 57.2)	46.76 (± 8.96)

Target organ	Toxin	24 h post dose		48 h post dose		168 h post dose	
		Median (min - max)	Mean (± SD)	Median (min - max)	Mean (± SD)	Median (min - max)	Mean (± SD)
Testicular	[D] Cadmium chloride	48.55 (44.5 - 60.5)	50.11 (± 4.83)	50.5 (43.3 - 56.9)	50.9 (± 5.03)	53 (46 - 65.5)	53.16 (± 7.7)
Testicular	[D] Carbendazim	51 (34.5 - 56.5)	47.4 (± 8.45)	49.5 (46.5 - 72.5)	54.1 (± 10.6)	49.5 (42.5 - 61.5)	50.3 (± 7.6)
Testicular	[D] Di-n-pentyl-phthalate	48.55 (38.8 - 56.7)	48.17 (± 5.36)	52.1 (45.4 - 69.9)	56.26 (± 9.66)	50.8 (47.1 - 60.8)	52.22 (± 5.67)
Testicular	[D] Ethane dimethane sulfonate (EDS)	44 (38 - 67)	47.2 (± 9.39)	45 (42 - 59)	48.6 (± 7.16)	55 (48 - 60)	55.2 (± 4.55)
Testicular	[D] Methoxyacetic acid	47.9 (36.45 - 56.25)	47.64 (± 5.66)	58.95 (44.35 - 67.85)	55.37 (± 9.91)	65.05 (48.75 - 73.15)	62.31 (± 10.07)
Multiple organ	[B] Adriamycin	54.5 (33.5 - 64.5)	50.2 (± 11.48)	48.5 (32.5 - 54.5)	46.3 (± 8.26)	50.5 (45.5 - 58.5)	50.9 (± 5.03)
Multiple organ	[C] Amphotericin B	48.8 (40.4 - 63.2)	48.98 (± 6.01)	51.8 (42.2 - 57.2)	51.44 (± 6.11)	53 (48.2 - 58.4)	52.88 (± 4.01)
Multiple organ	[C] Azaserine	51.2 (42.8 - 66.8)	53.24 (± 9.01)	50.6 (42.8 - 68.6)	52.64 (± 9.55)	41 (37.4 - 50)	43.64 (± 5.95)
Multiple organ	[A] Dexamethasone	44.5 (40 - 55)	46.2 (± 5.03)	50 (47 - 53)	50 (± 2.55)	67 (59 - 85)	69.4 (± 9.91)
Multiple organ	[E] Mitomycin-C	51 (45 - 64)	52 (± 5.85)	49 (40 - 54)	47.6 (± 5.59)	49 (43 - 53)	48.4 (± 3.97)
Physiological stressor	[C] 1,1-Dichloroethylene & maleic acid	49.4 (38.6 - 62.6)	50.3 (± 7.92)	50 (35.6 - 62)	50.12 (± 11.26)	50.6 (35 - 61.4)	50.36 (± 9.8)
Physiological stressor	[C] 2,4-Dinitrophenol	47.3 (34.1 - 63.5)	49.1 (± 8.6)	50.9 (40.1 - 63.5)	51.5 (± 8.38)	50.3 (38.3 - 59.3)	49.34 (± 7.53)
Physiological stressor	[B] 4-Pentenoic acid	49.5 (40 - 70)	49.6 (± 8.51)	48 (38 - 54)	47.8 (± 6.02)	54 (42 - 63)	54.4 (± 8.08)
Physiological stressor	[D] Acetazolamide	49.5 (41 - 67)	51.1 (± 7.74)	55.23 (50 - 58)	54.25 (± 3.58)	49 (38 - 63)	48.4 (± 9.37)
Physiological stressor	[C] Acivicin	47.3 (29.9 - 64.1)	48.2 (± 10.72)	50.3 (33.5 - 68.9)	49.7 (± 13.78)	52.7 (38.9 - 58.1)	49.34 (± 8.22)
Physiological stressor	[E] Ammonium chloride			85.5 (34.5 - 177.5)	91.1 (± 53.56)	50.65 (27.5 - 89.5)	51.82 (± 23.75)
Physiological stressor	[D] Carboplatin	48 (42 - 55)	47.8 (± 4.13)	49 (41 - 84)	53.2 (± 17.68)	56 (54 - 73)	60.8 (± 8.35)
Physiological stressor	[A] Choline and choline/methionine deficiency (chronic)	46 (31 - 60)	44.8 (± 9.13)	53 (38 - 58)	51.2 (± 7.85)	51 (43 - 54)	50.2 (± 4.32)
Physiological stressor	[B] Food restriction (chronic)	49 (37 - 54)	46.78 (± 6.89)	51 (50 - 55)	51.75 (± 2.22)	50 (42 - 63)	50.6 (± 8.14)
Physiological stressor	[D] Furosemide	53.5 (39.5 - 63.5)	53 (± 7.23)	47.5 (44.5 - 51.5)	48.1 (± 2.61)	44.5 (40.5 - 54.5)	46.3 (± 5.22)
Physiological stressor	[B] Insulin	50.5 (46 - 62)	52 (± 4.97)	46 (44 - 48)	46.2 (± 1.48)	57 (51 - 59)	55.4 (± 3.65)

Target organ	Toxin	24 h post dose		48 h post dose		168 h post dose	
		Median (min - max)	Mean (± SD)	Median (min - max)	Mean (± SD)	Median (min - max)	Mean (± SD)
Physiological stressor	[E] Methotrexate	53 (42.5 - 82.5)	55.7 (± 11.22)	47.5 (39.5 - 95.5)	57.7 (± 23.56)	42.5 (36.5 - 56.5)	44.9 (± 7.57)
Physiological stressor	[A] Partial hepatectomy	50.5 (46 - 71)	52.7 (± 7.72)	49 (42 - 54)	48.62 (± 4.79)	50 (38 - 74)	51.6 (± 13.45)
Physiological stressor	[A] Phenobarbital (chronic)	48 (40 - 63)	48.2 (± 7.27)	48 (39 - 55)	47 (± 6.52)	54.19 (45 - 61)	54.64 (± 6.18)
Physiological stressor	[A] Pregnenolone 16 alpha carbonitrile (chronic)						
Physiological stressor	[A] Probenecid	51 (42 - 190)	65.6 (± 44.18)	50 (44 - 70)	53.6 (± 9.96)	48 (45 - 66)	53.8 (± 9.88)
Physiological stressor	[C] Rosiglitazone	54.5 (40.1 - 68.9)	53 (± 8.47)	45.5 (37.7 - 50.9)	44.54 (± 5.8)	46.7 (33.5 - 62.3)	47.54 (± 11.13)
Physiological stressor	[C] Rosiglitazone (chronic)	50 (43.4 - 61.4)	51.38 (± 5.71)				
Physiological stressor	[E] Sodium bicarbonate	55.46 (52.43 - 56.45)	54.84 (± 1.72)				
Physiological stressor	[A] Unilateral nephrectomy	50.5 (44.5 - 64.5)	52.3 (± 6.14)	47.5 (31.5 - 50.5)	42.9 (± 8.05)	54.5 (48.5 - 64.5)	55.3 (± 6.14)
Physiological stressor	[B] Water deprivation (chronic)	50 (44 - 57)	50.04 (± 4)	51 (44 - 53)	49.33 (± 3.83)	46.26 (40 - 52)	46.42 (± 4.34)
No Effect	[E] Acetaminophen (chronic)	51 (44 - 53)	49.2 (± 3.63)			49 (43 - 52)	48.6 (± 3.51)
No Effect	[C] Buthionine sulphoxime	47.9 (41.9 - 52.7)	48.02 (± 4.09)	49.7 (43.1 - 55.7)	48.98 (± 4.75)	50.9 (45.5 - 55.7)	50.78 (± 3.63)
No Effect	[C] Ferrous sulphate	47.9 (33.2 - 52.4)	45.26 (± 7.39)	56.6 (47.6 - 65.6)	56.24 (± 7.55)	53.06 (39.8 - 62.6)	52.41 (± 8.27)
No Effect	[B] Ifosfamide	50.5 (46.5 - 57.5)	51.7 (± 4.16)	50.5 (47.5 - 57.5)	51.7 (± 4.21)	49.5 (42.5 - 54.5)	49.5 (± 4.58)
No Effect	[B] Lithocholic acid	49.5 (44.5 - 61.5)	51.2 (± 6.06)	49.5 (45.5 - 57.5)	50.3 (± 4.44)	55.5 (49.5 - 65.5)	56.9 (± 5.98)
No Effect	[E] Paraquat			47.5 (40.5 - 56.5)	48.1 (± 5.68)	52.5 (47.5 - 62.5)	53.7 (± 5.54)
No Effect	[D] Potassium dichromate	49 (21 - 55)	45.98 (± 10.3)	61 (35 - 68)	56 (± 12.92)	48 (40 - 55)	47.2 (± 7.05)
No Effect	[C] Trichlorethylene	48.8 (32 - 60.8)	47.18 (± 9.56)	46.4 (43.4 - 68)	53.24 (± 11.95)	56.6 (44 - 68.6)	56.84 (± 8.78)

A-F: Indicates Pharmaceutical Company & sample origin

Supplementary Table S14. Summary statistics for serum aspartate aminotransferase (IU/L) at 24 hrs, 48 hrs and 168 hrs post dose vehicle.

Target organ	Toxin	24 h post dose		48 h post dose		168 h post dose	
		Median (min - max)	Mean (± SD)	Median (min - max)	Mean (± SD)	Median (min - max)	Mean (± SD)
Liver	[E] 1,1-Dichloroethylene	110.5 (86.5 - 126.5)	109.7 (± 11.17)	98.5 (88.5 - 99.5)	96.9 (± 4.72)	93.5 (90.5 - 103.5)	95.1 (± 4.93)
Liver	[E] 1,2,3,4,5,6-hexachlorocyclohexane	116 (73.5 - 198.5)	117.1 (± 36.02)	81.5 (71.5 - 86.5)	80.9 (± 6.27)	128.5 (79.5 - 138.5)	113.9 (± 27.71)
Liver	[B] 1-Fluoropentane	87.5 (78 - 95)	87.3 (± 5.87)	112 (105 - 123)	113 (± 7.91)	120 (106 - 148)	123.8 (± 15.5)
Liver	[B] 2,4,6-Trihydroxyacetophenone (THA)	111.5 (85.5 - 167.5)	116.5 (± 30.48)	94.5 (86.5 - 100.5)	94.5 (± 5.79)	101.5 (79.5 - 128.5)	100.3 (± 18.57)
Liver	[B] 4-Amino-2,6-dichlorophenol (ADCP)	108.75 (108.65 - 112.45)	109.5 (± 1.53)	112.35 (111.2 - 129.15)	115.54 (± 7.64)	125.25 (125.25 - 125.45)	125.33 (± 0.11)
Liver	[C] Aflatoxin	91.9 (73.6 - 116.2)	94.42 (± 14.01)	95.8 (58.6 - 116.2)	85.96 (± 25.39)	137.2 (125.2 - 187.6)	151.72 (± 29.65)
Liver	[C] Allyl alcohol	95.8 (80.8 - 105.4)	94.96 (± 7.69)	103 (90.4 - 103.6)	100.24 (± 5.64)	102.4 (93.4 - 130.6)	107.32 (± 14.07)
Liver	[C] Allyl formate	85.3 (79.9 - 112.3)	89.56 (± 10.39)	109.9 (100.9 - 139.9)	115.42 (± 15.67)	106.3 (91.3 - 126.7)	107.02 (± 12.7)
Liver	[B] Azathioprine	108.75 (108.65 - 112.45)	109.5 (± 1.53)	112.35 (111.2 - 129.15)	115.54 (± 7.64)	125.25 (125.25 - 125.45)	125.33 (± 0.11)
Liver	[B] Bromobenzene	100 (96.5 - 132.5)	106.1 (± 11.92)	128.5 (90.5 - 215.5)	136.5 (± 47.31)	89.5 (88.5 - 100.5)	92.5 (± 5.05)
Liver	[C] Butylated hydroxytoluene	103.3 (83.2 - 131.8)	106.72 (± 15.24)	91 (79.6 - 102.4)	91.24 (± 8.24)	103.6 (89.8 - 135.4)	105.64 (± 17.62)
Liver	[D] Carbon tetrachloride	99.5 (88 - 125)	99.9 (± 9.99)	111 (87 - 115)	105 (± 11.83)	101 (99 - 111)	104.4 (± 6.07)
Liver	[C] Chlorpromazine	106.3 (93.1 - 183.1)	117.52 (± 28.47)	99.1 (84.1 - 111.1)	96.34 (± 11.1)	91.3 (85.9 - 124.9)	97.3 (± 15.77)
Liver	[B] Clofibrate	101.5 (92.5 - 132.5)	103.9 (± 11.06)	99.5 (92.5 - 132.5)	107.5 (± 16.42)	97.5 (84.5 - 107.5)	96.5 (± 10.79)
Liver	[B] Cyproterone acetate	111.5 (86.5 - 123.5)	106.9 (± 15.25)	92.5 (87.5 - 141.5)	106.9 (± 23.94)	97.5 (93.5 - 103.5)	97.9 (± 3.85)
Liver	[A] D-galactosamine	121.5 (102 - 136)	120.1 (± 11.3)	87 (79 - 89)	84.6 (± 5.18)	78 (56 - 98)	75.2 (± 15.72)
Liver	[B] Diethylhexylphthalate (DEHP)			100 (92 - 118)	103.6 (± 10.64)	100 (94 - 102)	98.8 (± 3.03)
Liver	[C] Dimethylformamide (DMF)	100 (74.2 - 139)	101.98 (± 21.36)	93.4 (86.2 - 127.6)	102.4 (± 16.89)	106 (82 - 153.4)	108.88 (± 27.82)
Liver	[C] Dimethylnitrosamine (DMN)	95.5 (88.9 - 106.3)	97.36 (± 6.15)	115.3 (105.7 - 143.5)	122.38 (± 18.05)	97.9 (92.5 - 106.9)	99.34 (± 5.43)
Liver	[A] Gadolinium chloride	120.5 (96.5 - 238.5)	141.7 (± 48.35)	53.5 (27.5 - 103.5)	63.9 (± 30.62)	32.5 (25.5 - 50.5)	36.9 (± 10.26)

Target organ	Toxin	24 h post dose		48 h post dose		168 h post dose	
		Median (min - max)	Mean (± SD)	Median (min - max)	Mean (± SD)	Median (min - max)	Mean (± SD)
Liver	[A, B, C, D, F] Hydrazine	101.5 (57.5 - 199.5)	106.48 (± 22.87)	111.5 (69 - 149.5)	107.58 (± 20.13)	82.5 (25.5 - 143)	85.11 (± 28.16)
Liver	[E] Hydrazine	110.5 (80 - 190)	123.5 (± 36.29)	88 (63 - 157)	93.8 (± 37.25)	103 (63 - 129)	97.4 (± 28.61)
Liver	[E] Indomethacin	129.5 (86 - 160)	126.3 (± 19.64)	70 (57 - 90)	73.4 (± 12.58)	82 (61 - 126)	86.6 (± 24.2)
Liver	[E] Ketoconazole			99 (84 - 104)	95.4 (± 9.29)	101 (94 - 149)	108.2 (± 23.08)
Liver	[C] Lead acetate	98.2 (86.8 - 110.2)	98.86 (± 7.32)	103.6 (89.2 - 119.2)	102.88 (± 11.12)	103 (94 - 113.2)	102.28 (± 7.57)
Liver	[A] Lipopolysaccharide (LPS)	151.5 (89 - 181)	139 (± 34.61)	85 (64 - 158)	98.2 (± 36.19)	79 (71 - 136)	89 (± 26.64)
Liver	[B] Methapyrilene	101 (93.5 - 124.5)	105.8 (± 11.45)	100.5 (92.5 - 182.5)	114.1 (± 38.45)	98.5 (93.5 - 107.5)	100.5 (± 5.52)
Liver	[E] Methylene dianiline	109 (88.5 - 163.5)	115.6 (± 23.05)	97.5 (80.5 - 138.5)	104.5 (± 26.03)	84.5 (74.5 - 97.5)	85.3 (± 9.04)
Liver	[C] Monocrotaline	97.6 (87.4 - 118)	99.58 (± 11.24)	97.6 (90.4 - 121.6)	103 (± 14.21)	101.2 (96.4 - 135.4)	107.56 (± 15.78)
Liver	[C] N-methylformamide (NMF)	94.3 (83.5 - 123.1)	98.14 (± 12.25)	98.5 (90.7 - 121.3)	102.82 (± 12.29)	127.3 (113.5 - 152.5)	130.54 (± 15.05)
Liver	[D] Phalloidin (chronic)			95.7 (81.8 - 113.5)	96.7 (± 11.82)	100.2 (97.9 - 107.6)	102 (± 3.95)
Liver	[E] Phenyl diisothiocyanate	100.5 (92.5 - 131.5)	103.8 (± 11.66)	84.5 (76.5 - 97.5)	87.1 (± 8.71)	108.5 (101.5 - 119.5)	108.9 (± 7.13)
Liver	[E] Phenyl isothiocyanate	109.33 (100 - 120)	110.47 (± 8.93)	84 (77 - 108)	90.4 (± 13.01)	93 (66 - 119)	88.6 (± 21.45)
Liver	[B] Retinyl palmitate	108.75 (108.65 - 112.45)	109.5 (± 1.53)	112.35 (111.2 - 129.15)	115.54 (± 7.64)	125.25 (125.25 - 125.45)	125.33 (± 0.11)
Liver	[B] Sodium Valproate			97.5 (77.5 - 104.5)	93.5 (± 11.6)	102.5 (93.5 - 139.5)	109.9 (± 18.69)
Liver	[C] a-Naphthylisothiocyanate (ANIT)	100 (80.5 - 109.3)	98.14 (± 8.07)	110.5 (97.9 - 117.7)	107.86 (± 8.93)	93.7 (89.5 - 114.1)	99.82 (± 10.86)
Kidney	[D] 2-Bromophenol	99 (81 - 157)	102.2 (± 22.34)	117 (97 - 196)	128.6 (± 41.02)	95 (72 - 120)	98 (± 18.8)
Kidney	[E] 3,5-Dichloroaniline hydrochloride	104.5 (85 - 130)	105.3 (± 13.61)	102 (90 - 118)	102.4 (± 10.04)	93 (85 - 94)	90.6 (± 3.91)
Kidney	[E] Atractyloside	104.75 (88 - 125)	105.75 (± 11.15)	82 (74 - 99)	83.4 (± 9.94)	104 (86 - 108)	100.6 (± 8.65)
Kidney	[D] Bromoethylamine hydrobromide	130 (108 - 153)	130.6 (± 14.34)	84 (76 - 92)	83 (± 6.32)	82 (65 - 86)	78 (± 8.51)

Target organ	Toxin	24 h post dose		48 h post dose		168 h post dose	
		Median (min - max)	Mean (± SD)	Median (min - max)	Mean (± SD)	Median (min - max)	Mean (± SD)
Kidney	[D] Cephaloridine	113.5 (89 - 130)	110.7 (± 12.06)	87 (78 - 116.15)	93.83 (± 16.1)	93 (81 - 100)	90.2 (± 8.76)
Kidney	[B] Chlorethanamine	106.5 (87 - 147)	106.8 (± 16.72)	96 (87 - 100)	94.6 (± 5.18)	100 (95 - 112)	103.4 (± 7.27)
Kidney	[A] Cisplatin	105 (89.5 - 129.5)	106.9 (± 14.24)	99.5 (86.5 - 135.5)	107.1 (± 20.33)	97.5 (68.5 - 139.5)	103.3 (± 34.43)
Kidney	[A] D-limonene (chronic)						
Kidney	[E] Dichlorophenyl succinimide	109 (88.5 - 163.5)	115.6 (± 23.05)	97.5 (80.5 - 138.5)	104.5 (± 26.03)	84.5 (74.5 - 97.5)	85.3 (± 9.04)
Kidney	[D] Ethylene glycol	107.5 (99 - 129)	111.3 (± 10.83)	93 (92 - 103)	96.2 (± 4.97)	90 (82 - 99)	90.6 (± 8.26)
Kidney	[A] Folic acid	116.89 (99 - 158)	122.5 (± 17.16)	99 (89 - 175)	113.79 (± 34.95)	90 (88 - 101)	92.6 (± 5.73)
Kidney	[A] Gentamicin	99 (85 - 119.15)	100.11 (± 8.72)	100 (97 - 107)	102 (± 4.3)	117 (96 - 136)	117.2 (± 15.16)
Kidney	[B] Maleic acid	108.75 (108.65 - 112.45)	109.5 (± 1.53)	112.35 (111.2 - 129.15)	115.54 (± 7.64)	125.25 (125.25 - 125.45)	125.33 (± 0.11)
Kidney	[A] N-phenylanthranilic acid (chronic)	94 (83.5 - 145.99)	102.7 (± 20.71)	103.5 (97.5 - 188.5)	118.7 (± 39.12)	103.5 (96.5 - 119.5)	105.7 (± 8.87)
Kidney	[D] Para-aminophenol	112 (90 - 149)	113.2 (± 19.31)	95 (87 - 147.09)	103.62 (± 24.65)	100 (72 - 116)	98.4 (± 17.87)
Kidney	[A] Puromycin			102.5 (96.5 - 107.5)	101.7 (± 4.66)	96.5 (90.5 - 105.5)	98.1 (± 6.66)
Kidney	[B] Vancomycin hydrochloride			105 (97 - 119)	106.2 (± 8.23)	97 (94 - 111)	99.2 (± 6.76)
Liver & Kidney	[E] Acetaminophen	108 (90 - 192)	114 (± 29.44)	94 (88 - 102)	94.8 (± 5.17)	105 (80 - 150)	110.2 (± 27.89)
Liver & Kidney	[B] Aurothiomalate	108.75 (108.65 - 112.45)	109.5 (± 1.53)	112.35 (111.2 - 129.15)	115.54 (± 7.64)	125.25 (125.25 - 125.45)	125.33 (± 0.11)
Liver & Kidney	[C] Chloroform	101.2 (79.6 - 121)	101.32 (± 11.97)	97 (79.6 - 119.8)	101.08 (± 17.67)	98.8 (82 - 121)	97.6 (± 16.43)
Liver & Kidney	[D] Cyclosporin	106.5 (83 - 119)	105.4 (± 10.6)	92 (89 - 106)	95 (± 6.82)	87 (76 - 134)	92.4 (± 23.8)
Liver & Kidney	[D] Dichlorobenzene	112 (93 - 118)	108.2 (± 8.83)	89 (80 - 140)	102.4 (± 24.81)	86 (82 - 98)	87.8 (± 6.02)
Liver & Kidney	[C] Ethionine	106 (75.1 - 139.9)	102.34 (± 19.21)	111.1 (69.1 - 150.7)	114.1 (± 32.58)	97.3 (74.5 - 100.3)	89.26 (± 12.47)
Liver & Kidney	[B] Hexachlorobutadiene (HCBD)			104 (92 - 131)	107.2 (± 14.38)	94 (91 - 104)	95.8 (± 5.07)

Target organ	Toxin	24 h post dose		48 h post dose		168 h post dose	
		Median (min - max)	Mean (± SD)	Median (min - max)	Mean (± SD)	Median (min - max)	Mean (± SD)
Liver & Kidney	[B] Mercuric chloride	97.5 (90 - 114)	101.2 (± 9.27)	112 (96 - 128)	110 (± 13.62)	102 (88 - 124)	102.4 (± 13.37)
Liver & Kidney	[E] Microcystin-LR			96.5 (85.5 - 126.5)	100.3 (± 16.95)	100.5 (96.5 - 104.5)	100.5 (± 2.92)
Liver & Kidney	[E] Rotenone	106.5 (94 - 126)	108.1 (± 12.53)	95 (82 - 116)	94.8 (± 13.81)	102 (91 - 113)	101.4 (± 9.45)
Liver & Kidney	[E] S-(1,2-dichlorovinyl)-cysteine (DCVC)	104 (95 - 141)	109.7 (± 16.57)	97 (90 - 108)	96.8 (± 7.19)	103 (82 - 107)	98 (± 10.17)
Liver & Kidney	[D] Thioacetamide	107 (91.5 - 118.5)	106.4 (± 8.62)	94.5 (86.5 - 178.5)	111.3 (± 38.21)	92.5 (87.5 - 106.5)	94.5 (± 7.31)
Pancreas	[E] 1-Cyano-2-hydroxy-3-butene	103.5 (9.5 - 170.5)	100.4 (± 39.98)	100.5 (89.5 - 115.5)	101.3 (± 11.23)	93.5 (77.5 - 121.5)	98.3 (± 16.77)
Pancreas	[C] Caerulin	98.2 (83.2 - 118.6)	99.1 (± 10.76)	130.6 (81.4 - 173.2)	121.48 (± 37.04)	104.8 (90.4 - 113.8)	102.64 (± 9.11)
Pancreas	[E] L-arginine	111 (78 - 131)	110 (± 15.92)	70 (51 - 92)	71.4 (± 16.82)	96 (69 - 176)	106.6 (± 40.91)
Pancreas	[B] Streptozotocin	103 (92 - 136)	105.9 (± 13.49)	106 (86 - 123)	104 (± 13.4)	98 (84 - 123)	98.8 (± 15.22)
Testicular	[D] 1,3-Dinitrobenzene	95.5 (76 - 124)	97.83 (± 15.54)	117 (98 - 140)	116.8 (± 18.19)	107 (92 - 149)	116.6 (± 25.05)
Testicular	[C] Cadmium chloride	96.7 (77.5 - 145.9)	104.8 (± 20.26)	101.5 (89.5 - 136.9)	106.9 (± 19.14)	111.7 (83.5 - 143.5)	109.9 (± 22.16)
Testicular	[D] Cadmium chloride	100 (82.2 - 106.7)	96.13 (± 9.36)	125.2 (81 - 170.5)	125.46 (± 36.96)	90.6 (85.9 - 117.9)	97.98 (± 13.96)
Testicular	[D] Carbendazim	102.5 (93 - 112)	101.7 (± 6.6)	100 (97 - 178)	117.6 (± 34.43)	98 (93 - 154)	111.4 (± 25.88)
Testicular	[D] Di-n-pentyl-phthalate	92.4 (86 - 98.4)	92.04 (± 4.65)	168.1 (128.1 - 191)	160.46 (± 25.07)	129 (101.6 - 162)	131.4 (± 21.64)
Testicular	[D] Ethane dimethane sulfonate (EDS)	93.5 (84 - 140)	97.8 (± 16.08)	100 (73 - 113)	96.6 (± 14.64)	119 (108 - 336)	159.2 (± 98.97)
Testicular	[D] Methoxyacetic acid	94.45 (78.95 - 107.35)	92.88 (± 9.13)	118.45 (93.05 - 132.15)	111.87 (± 16.69)	168.75 (109.75 - 203.25)	167.69 (± 37.03)
Multiple organ	[B] Adriamycin	97 (79.5 - 127.5)	101.3 (± 17.22)	107.5 (96.5 - 146.5)	112.9 (± 19.42)	99.5 (86.5 - 104.5)	96.7 (± 7.16)
Multiple organ	[C] Amphotericin B	106 (72.7 - 135.7)	101.8 (± 22.76)	95.5 (88.9 - 106.9)	97.18 (± 7.95)	96.1 (81.7 - 138.7)	107.98 (± 24.75)
Multiple organ	[C] Azaserine	103.6 (76.6 - 150.4)	108.28 (± 25.08)	113.2 (82 - 119.8)	104.2 (± 16.64)	97.6 (83.2 - 101.2)	94.36 (± 7.29)
Multiple organ	[A] Dexamethasone	97.5 (91 - 100)	96.3 (± 3.33)	106 (99 - 109)	105.8 (± 4.09)	128 (116 - 136)	127 (± 8.12)
Multiple organ	[E] Mitomycin-C	101 (93 - 120)	104.5 (± 9.01)	92 (84 - 104)	92.6 (± 7.4)	105 (97 - 119)	106.6 (± 9.71)

Target organ	Toxin	24 h post dose		48 h post dose		168 h post dose	
		Median (min - max)	Mean (± SD)	Median (min - max)	Mean (± SD)	Median (min - max)	Mean (± SD)
Physiological stressor	[C] 1,1-Dichloroethylene & maleic acid	104.5 (82.6 - 186.4)	115.84 (± 34.81)	92.8 (87.4 - 109.6)	96.16 (± 8.66)	99.4 (88.6 - 137.2)	108.88 (± 23.38)
Physiological stressor	[C] 2,4-Dinitrophenol	96.1 (85.3 - 128.5)	100.36 (± 15.12)	104.5 (100.3 - 127.9)	108.22 (± 11.24)	87.7 (76.3 - 128.5)	95.74 (± 22.45)
Physiological stressor	[B] 4-Pentenoic acid	92 (77 - 114)	95.3 (± 13.86)	82 (77 - 112)	88.4 (± 14.01)	124 (106 - 148)	124.8 (± 16.02)
Physiological stressor	[D] Acetazolamide	108.5 (99 - 128)	110.3 (± 10.04)	87 (66 - 116.15)	93.43 (± 21)	76 (69 - 87)	76.2 (± 7.66)
Physiological stressor	[C] Acivicin	100 (83.5 - 123.1)	102.88 (± 11.26)	91.9 (86.5 - 108.7)	94.3 (± 9.15)	125.5 (90.7 - 141.1)	120.34 (± 21.95)
Physiological stressor	[E] Ammonium chloride			107 (99 - 127)	112 (± 13.56)	98 (96 - 105)	99 (± 3.46)
Physiological stressor	[D] Carboplatin	95.5 (82 - 111)	97.3 (± 10.13)	114 (97 - 169)	124.4 (± 29.97)	98 (92 - 125)	102.6 (± 13.78)
Physiological stressor	[A] Choline and choline/methionine deficiency (chronic)	88.5 (74.5 - 116.5)	91.7 (± 14.63)	105.5 (101.5 - 118.5)	107.5 (± 6.89)	106.5 (82.5 - 113.5)	102.7 (± 12.77)
Physiological stressor	[B] Food restriction (chronic)	103 (80 - 122)	103.89 (± 13.2)	101.5 (96 - 119)	104.5 (± 10.02)	97 (83 - 108)	96.2 (± 9.04)
Physiological stressor	[D] Furosemide	123.5 (97.5 - 147.5)	122.1 (± 16.09)	86.5 (78.5 - 105.5)	88.9 (± 11.28)	68.5 (65.5 - 86.5)	72.7 (± 8.38)
Physiological stressor	[B] Insulin	96 (77 - 132)	100.5 (± 16.7)	93 (87 - 104)	95.4 (± 7.3)	102 (97 - 111)	103.6 (± 5.46)
Physiological stressor	[E] Methotrexate	103 (92.5 - 156.5)	109.4 (± 19.06)	118.5 (88.5 - 150.5)	116.9 (± 28.33)	90.5 (77.5 - 100.5)	90.7 (± 9.04)
Physiological stressor	[A] Partial hepatectomy	112 (94 - 145)	113.5 (± 15.02)	93 (86 - 177)	111.07 (± 37.58)	83 (68 - 86)	78.6 (± 8.05)
Physiological stressor	[A] Phenobarbital (chronic)	111.5 (96 - 138)	112.6 (± 14.67)	74 (72 - 106)	80 (± 14.58)	100 (73 - 116.35)	95.07 (± 16.36)
Physiological stressor	[A] Pregnenolone 16 alpha carbonitrile (chronic)						
Physiological stressor	[A] Probenecid	102 (87 - 271)	117.1 (± 54.75)	99 (76 - 117)	98.8 (± 15.09)	96 (90 - 133)	104.4 (± 17.98)
Physiological stressor	[C] Rosiglitazone	100 (59.2 - 137.8)	103.36 (± 21.76)	81.4 (76 - 115.6)	92.08 (± 17.78)	103.6 (83.8 - 112.6)	100.36 (± 10.6)
Physiological stressor	[C] Rosiglitazone (chronic)	100 (84.4 - 155.8)	106 (± 22.47)				
Physiological stressor	[E] Sodium bicarbonate	143.35 (143.15 - 143.9)	143.39 (± 0.28)				
Physiological stressor	[A] Unilateral nephrectomy	101.5 (75 - 179)	109.4 (± 27.71)	90 (79 - 131)	98.6 (± 21.38)	98 (80 - 118)	95.6 (± 15.18)

Target organ	Toxin	24 h post dose		48 h post dose		168 h post dose	
		Median (min - max)	Mean (± SD)	Median (min - max)	Mean (± SD)	Median (min - max)	Mean (± SD)
Physiological stressor	[B] Water deprivation (chronic)	103 (96 - 121)	105.27 (± 7.48)	106 (87 - 129.15)	106.36 (± 14.13)	94.5 (76 - 125.25)	95.04 (± 17.13)
No Effect	[E] Acetaminophen (chronic)	105 (99 - 107)	103.4 (± 3.65)			99 (96 - 107)	99.8 (± 4.32)
No Effect	[C] Buthionine sulphoxime	91.6 (78.1 - 100.9)	91.6 (± 7.01)	108.1 (104.5 - 115.3)	108.46 (± 4.48)	108.1 (99.1 - 128.5)	109.9 (± 11.2)
No Effect	[C] Ferrous sulphate	95.8 (75.4 - 141.4)	102.64 (± 23.39)	101.8 (92.8 - 174.4)	120.16 (± 33.53)	98.2 (78.4 - 132.6)	104.36 (± 23.38)
No Effect	[B] Ifosfamide	99.5 (78 - 118)	99.5 (± 13.57)	101 (84 - 106)	97.8 (± 9.26)	100 (84 - 104)	95 (± 8.89)
No Effect	[B] Lithocholic acid	103 (90.5 - 126.5)	106.1 (± 12)	94.5 (86.5 - 122.5)	99.1 (± 13.89)	104.5 (95.5 - 113.5)	104.1 (± 6.99)
No Effect	[E] Paraquat			86.5 (84.5 - 93.5)	88.5 (± 3.81)	109.5 (106.5 - 132.5)	113.3 (± 10.8)
No Effect	[D] Potassium dichromate	99 (85 - 122.25)	99.83 (± 11)	114 (99 - 185)	132.4 (± 37.33)	124 (98 - 135)	117 (± 17.33)
No Effect	[C] Trichlorethylene	93.7 (79.6 - 105.4)	92.38 (± 10.37)	125.8 (83.8 - 142)	115.12 (± 23.73)	104.2 (77.2 - 113.2)	97.24 (± 16.51)

A-F: Indicates Pharmaceutical Company & sample origin

Supplementary Table S15. Summary statistics for urine total volume collected (mL) at 24 hrs, 48 hrs and 168 hrs post dose vehicle.

Target organ	Toxin	24 h post dose		48 h post dose		168 h post dose	
		Median (min - max)	Mean (± SD)	Median (min - max)	Mean (± SD)	Median (min - max)	Mean (± SD)
Liver	[E] 1,1-Dichloroethylene	6.5 (4.5 - 9)	6.7 (± 1.21)	11 (7 - 30)	11.85 (± 6.7)	12 (9 - 14)	11.5 (± 2.18)
Liver	[E] 1,2,3,4,5,6-hexachlorocyclohexane	10 (6 - 13.5)	9.8 (± 2.54)	11 (9.5 - 50)	15.75 (± 12.23)	13 (11 - 20)	15 (± 4.18)
Liver	[B] 1-Fluoropentane	11.3 (8 - 20)	11.88 (± 3.27)	14.8 (11 - 21.2)	15.22 (± 3.32)	14.6 (11.2 - 23)	15.44 (± 4.52)
Liver	[B] 2,4,6-Trihydroxyacetophenone (THA)	8.9 (3 - 13)	8.66 (± 2.6)	11.2 (6.6 - 17)	11.52 (± 2.88)	13 (8.6 - 15.2)	11.72 (± 2.98)
Liver	[B] 4-Amino-2,6-dichlorophenol (ADCP)	9.99 (7.68 - 11.64)	9.83 (± 0.99)	10.64 (8.87 - 13.53)	11.03 (± 1.5)	14.82 (10.75 - 16.2)	14.13 (± 2.37)
Liver	[C] Aflatoxin	9.5 (6 - 11)	9.2 (± 1.55)	10.5 (4 - 12)	9.9 (± 2.6)	10 (8 - 12)	10.2 (± 1.79)

Target organ	Toxin	24 h post dose		48 h post dose		168 h post dose	
		Median (min - max)	Mean (± SD)	Median (min - max)	Mean (± SD)	Median (min - max)	Mean (± SD)
Liver	[C] Allyl alcohol	12 (8 - 20)	12.5 (± 3.98)	9 (8 - 18)	10.33 (± 3.88)	10 (8 - 13)	10.16 (± 1.8)
Liver	[C] Allyl formate	12 (8 - 14)	11.4 (± 1.65)	12 (10 - 14)	12 (± 1.56)	15 (14 - 21)	16.2 (± 2.77)
Liver	[B] Azathioprine	9.99 (7.68 - 11.64)	9.83 (± 0.99)	10.64 (8.87 - 13.53)	11.03 (± 1.5)	14.82 (10.75 - 16.2)	14.13 (± 2.37)
Liver	[B] Bromobenzene	7.75 (6.6 - 9.8)	7.86 (± 1.01)	12.1 (4 - 14.8)	11.54 (± 3.02)	12.4 (12 - 14.8)	13.14 (± 1.31)
Liver	[C] Butylated hydroxytoluene	10 (7 - 13)	9.6 (± 1.58)	14 (12 - 18)	14.5 (± 2.37)	16 (14 - 20)	16.6 (± 2.41)
Liver	[D] Carbon tetrachloride	9 (4 - 15)	8.7 (± 3.02)	9.95 (7.8 - 16)	10.95 (± 2.49)	14 (12 - 18)	14.4 (± 2.61)
Liver	[C] Chlorpromazine	10 (8 - 14)	10.4 (± 1.58)	10 (8 - 18)	11.2 (± 2.97)	14 (12 - 15)	13.4 (± 1.34)
Liver	[B] Clofibrate	10.1 (8.2 - 14.4)	10.52 (± 2.09)	13.35 (11.8 - 17)	13.83 (± 1.47)	13.8 (13 - 15.2)	14.04 (± 1.01)
Liver	[B] Cyproterone acetate	7 (4.8 - 10.4)	7.26 (± 1.82)	10 (7.2 - 13.4)	9.95 (± 1.76)	12.2 (9.4 - 15.8)	12.56 (± 2.71)
Liver	[A] D-galactosamine	7.3 (5 - 12)	7.76 (± 2.2)	8.1 (4.1 - 18.3)	8.83 (± 3.76)	14 (12 - 17.6)	14.4 (± 2.38)
Liver	[B] Diethylhexylphthalate (DEHP)	9.8 (5.8 - 14.6)	10.32 (± 2.77)	14.3 (11.8 - 18)	14.56 (± 2.11)	11.6 (10.4 - 18)	13.4 (± 3.32)
Liver	[C] Dimethylformamide (DMF)	10 (8 - 12)	10.1 (± 1.2)	12 (7 - 14)	11.5 (± 2.07)	14 (12 - 15)	13.4 (± 1.34)
Liver	[C] Dimethylnitrosamine (DMN)	10 (8 - 11)	9.5 (± 0.97)	10 (3 - 14)	10.3 (± 2.91)	16 (12 - 17)	15.4 (± 2.07)
Liver	[A] Gadolinium chloride	10.4 (7.3 - 21.2)	11.29 (± 4.28)	11.1 (5.8 - 19)	11.5 (± 4.08)	11.2 (8.2 - 15.4)	11.96 (± 3.15)
Liver	[A, B, C, D, F] Hydrazine	10.7 (3.7 - 26.9)	11.38 (± 4.83)	12 (5.8 - 25.2)	12.9 (± 4.59)	14 (5 - 23)	13.98 (± 4.26)
Liver	[E] Hydrazine	12.5 (10 - 16)	12.3 (± 1.97)	10 (5 - 15)	9.25 (± 2.66)	15 (10 - 20)	15.2 (± 4.76)
Liver	[E] Indomethacin	9.75 (9 - 12.5)	10.15 (± 1.27)	12.25 (9.5 - 15)	12.3 (± 1.9)	14 (12.5 - 18)	14.9 (± 2.13)
Liver	[E] Ketoconazole	11.15 (7 - 15)	10.73 (± 2.54)	14 (8.8 - 18)	13.68 (± 2.87)	13 (10 - 20)	13.6 (± 3.78)
Liver	[C] Lead acetate	12 (8 - 18)	12.3 (± 2.91)	14 (6 - 22)	13.9 (± 5.09)	17 (12 - 24)	18 (± 4.42)
Liver	[A] Lipopolysaccharide (LPS)	9.65 (8.2 - 13.2)	10.44 (± 1.89)	14.1 (9.7 - 18.7)	14.15 (± 2.89)	14 (6.4 - 25.2)	16 (± 7.61)
Liver	[B] Methapyrilene	8 (5.2 - 15.8)	9.08 (± 3.17)	15.15 (2.6 - 17)	12.39 (± 4.96)	15.4 (12.6 - 18)	15.12 (± 2.39)

Target organ	Toxin	24 h post dose		48 h post dose		168 h post dose	
		Median (min - max)	Mean (± SD)	Median (min - max)	Mean (± SD)	Median (min - max)	Mean (± SD)
Liver	[E] Methylene dianiline	8.5 (6.5 - 13)	9 (± 2.39)	12 (8 - 14.5)	11.75 (± 2.28)	13 (7.5 - 20)	13.5 (± 4.77)
Liver	[C] Monocrotaline	11 (10 - 14)	11.5 (± 1.27)	12 (10 - 15)	12.2 (± 1.55)	15 (11 - 19)	15.2 (± 3.03)
Liver	[C] N-methylformamide (NMF)	11 (8 - 18)	11.9 (± 3.48)	12.5 (8 - 26)	13.5 (± 5.21)	16 (10 - 16)	14 (± 2.83)
Liver	[D] Phalloidin (chronic)	10.5 (9 - 14)	10.94 (± 1.65)	9.75 (7.5 - 14)	10.15 (± 2.08)	13.5 (12.85 - 15.5)	13.84 (± 1.01)
Liver	[E] Phenyl diisothiocyanate	8 (6.5 - 14)	8.8 (± 2.52)	10.25 (6.5 - 14)	10.1 (± 2.38)	13 (10 - 16.5)	13.4 (± 2.82)
Liver	[E] Phenyl isothiocyanate	9 (6.5 - 11)	8.9 (± 1.68)	11 (6.5 - 17)	11.3 (± 2.97)	12.5 (10.5 - 14)	12.4 (± 1.29)
Liver	[B] Retinyl palmitate	9.99 (7.68 - 11.64)	9.83 (± 0.99)	10.64 (8.87 - 13.53)	11.03 (± 1.5)	14.82 (10.75 - 16.2)	14.13 (± 2.37)
Liver	[B] Sodium Valproate	11.3 (8.6 - 13.2)	11.12 (± 1.49)	15.1 (9.8 - 17.2)	14.44 (± 2.49)	14.2 (13 - 19.2)	15.24 (± 2.64)
Liver	[C] a-Naphthylisothiocyanate (ANIT)	8.5 (7 - 10)	8.6 (± 0.97)	9.5 (8 - 12)	9.5 (± 1.18)	8 (7 - 10)	8.4 (± 1.14)
Kidney	[D] 2-Bromophenol	8.25 (7 - 12)	8.65 (± 1.58)	9 (8 - 12)	9.5 (± 1.65)	11 (8 - 16)	11.5 (± 3.46)
Kidney	[E] 3,5-Dichloroaniline hydrochloride	9.5 (6.5 - 14)	9.95 (± 2.35)	12 (9.5 - 16)	12.45 (± 2.66)	12 (9.5 - 16)	12.5 (± 2.92)
Kidney	[E] Atractyloside	12.25 (7 - 15)	11.75 (± 2.45)	13.25 (8.5 - 17)	13.2 (± 2.71)	16 (12 - 25)	16.6 (± 5.37)
Kidney	[D] Bromoethylamine hydrobromide	12 (7.8 - 18)	12.18 (± 3.15)	12.5 (6.8 - 24)	13.58 (± 4.91)	14 (11 - 18)	14.2 (± 2.86)
Kidney	[D] Cephaloridine	8.65 (1.5 - 14)	8.8 (± 3.73)	10.5 (8 - 20)	11.8 (± 3.71)	15 (12 - 16)	14.2 (± 2.05)
Kidney	[B] Chlorethanamine	10.1 (8.8 - 17)	11.18 (± 2.73)	14.6 (12.2 - 21.2)	14.97 (± 2.63)	16.6 (12.6 - 20)	16.76 (± 2.78)
Kidney	[A] Cisplatin	8.8 (4.8 - 14)	8.96 (± 2.89)	10.5 (6.8 - 18)	11.26 (± 2.91)	12 (7 - 15)	11.2 (± 3.19)
Kidney	[A] D-limonene (chronic)	9.5 (7 - 16)	10 (± 2.75)	9.5 (6 - 17)	10.6 (± 3.78)	15 (9 - 28)	18.4 (± 9.02)
Kidney	[E] Dichlorophenyl succinimide	8.5 (6.5 - 13)	9 (± 2.39)	12 (8 - 14.5)	11.75 (± 2.28)	13 (7.5 - 20)	13.5 (± 4.77)
Kidney	[D] Ethylene glycol	8.75 (7 - 19)	10.45 (± 3.79)	12 (10 - 16)	11.7 (± 1.89)	13 (9 - 17)	13.2 (± 3.77)
Kidney	[A] Folic acid	9.55 (1.9 - 12)	9.26 (± 3.05)	11 (8.1 - 15)	11.15 (± 2.07)	14 (8 - 16)	12.8 (± 3.03)
Kidney	[A] Gentamicin	12 (10 - 18)	12.6 (± 2.5)	13.5 (10 - 18)	13.3 (± 2.26)	13 (8.2 - 24)	14.48 (± 6.57)

Target organ	Toxin	24 h post dose		48 h post dose		168 h post dose	
		Median (min - max)	Mean (± SD)	Median (min - max)	Mean (± SD)	Median (min - max)	Mean (± SD)
Kidney	[B] Maleic acid	9.99 (7.68 - 11.64)	9.83 (± 0.99)	10.64 (8.87 - 13.53)	11.03 (± 1.5)	14.82 (10.75 - 16.2)	14.13 (± 2.37)
Kidney	[A] N-phenylanthranilic acid (chronic)	9.3 (6.8 - 18.2)	10.06 (± 3.64)	9.4 (7 - 13.6)	9.44 (± 2.06)	17 (7.6 - 18.6)	14.76 (± 4.77)
Kidney	[D] Para-aminophenol	11 (8.2 - 16)	11.92 (± 2.61)	15 (10 - 20)	14.9 (± 3.6)	17 (12 - 18)	15.6 (± 2.51)
Kidney	[A] Puromycin	12 (9.8 - 15)	11.78 (± 1.84)	12 (10 - 14)	11.7 (± 1.49)	14 (14 - 16)	14.6 (± 0.89)
Kidney	[B] Vancomycin hydrochloride	8 (2 - 16.2)	7.5 (± 5)	11.6 (4 - 14.6)	10.72 (± 3.74)	14.2 (12.8 - 15)	14 (± 0.97)
Liver & Kidney	[E] Acetaminophen	10 (7 - 14)	10.45 (± 2.33)	13.75 (9 - 16)	13.3 (± 2.44)	14.5 (9.5 - 17)	13.5 (± 3.34)
Liver & Kidney	[B] Aurothiomalate	9.99 (7.68 - 11.64)	9.83 (± 0.99)	10.64 (8.87 - 13.53)	11.03 (± 1.5)	14.82 (10.75 - 16.2)	14.13 (± 2.37)
Liver & Kidney	[C] Chloroform	8 (6 - 12)	8.1 (± 1.85)	12 (10 - 25)	13.5 (± 4.67)	18 (10 - 22)	17.6 (± 4.98)
Liver & Kidney	[D] Cyclosporin	10 (8.2 - 16)	10.47 (± 2.22)	12 (7 - 22)	12.7 (± 4.45)	17 (16 - 20)	17.6 (± 1.52)
Liver & Kidney	[D] Dichlorobenzene	7.7 (5.8 - 9.5)	7.39 (± 1.15)	10.5 (8.5 - 12)	10.7 (± 1.27)	14 (10 - 15)	13 (± 2)
Liver & Kidney	[C] Ethionine	8 (7 - 12)	8.2 (± 1.55)	11 (8 - 20)	12.1 (± 3.9)	12 (10 - 16)	12.4 (± 2.61)
Liver & Kidney	[B] Hexachlorobutadiene (HCBD)	8.9 (6.2 - 11.8)	8.9 (± 2)	14.6 (11 - 22)	15.5 (± 3.21)	17.2 (12 - 22.2)	17.4 (± 3.8)
Liver & Kidney	[B] Mercuric chloride	11.5 (6 - 15.8)	11.12 (± 2.67)	11.7 (7.4 - 17.6)	12.18 (± 2.95)	15.6 (9.2 - 20.2)	14.96 (± 4.01)
Liver & Kidney	[E] Microcystin-LR	10 (8.5 - 13)	10.25 (± 1.38)	12.5 (10 - 18)	13.1 (± 2.28)	13 (10.5 - 17)	13.1 (± 2.61)
Liver & Kidney	[E] Rotenone	7.5 (6.5 - 12.5)	8.2 (± 1.81)	12.25 (9 - 17)	12.2 (± 2.78)	14 (11 - 22)	15.2 (± 4.21)
Liver & Kidney	[E] S-(1,2-dichlorovinyl)-cysteine (DCVC)	10 (8.5 - 13)	10.15 (± 1.31)	13 (9.5 - 14.5)	12.6 (± 1.7)	12.5 (11 - 16)	13.1 (± 1.95)
Liver & Kidney	[D] Thioacetamide	10 (2.5 - 15)	9.95 (± 3.64)	15 (9.5 - 20)	14.85 (± 2.98)	20 (19 - 23)	21 (± 1.87)
Pancreas	[E] 1-Cyano-2-hydroxy-3-butene	10 (8 - 14)	10.45 (± 1.67)	13.5 (9.5 - 15)	12.95 (± 1.98)	15 (11 - 17)	14 (± 2.45)
Pancreas	[C] Caerulin	10 (3 - 12)	9.4 (± 2.46)	10 (10 - 11)	10.4 (± 0.52)	14 (13 - 21)	16.4 (± 3.78)
Pancreas	[E] L-arginine	9.5 (7 - 15)	10.15 (± 2.47)	13 (8 - 16)	13 (± 2.79)	15.5 (11 - 22.5)	15.7 (± 4.62)
Pancreas	[B] Streptozotocin	9.5 (8 - 13)	9.83 (± 1.56)	13.6 (9.6 - 16.2)	13.43 (± 1.94)	13 (9.8 - 15.4)	12.72 (± 2.13)

Target organ	Toxin	24 h post dose		48 h post dose		168 h post dose	
		Median (min - max)	Mean (± SD)	Median (min - max)	Mean (± SD)	Median (min - max)	Mean (± SD)
Testicular	[D] 1,3-Dinitrobenzene	9.55 (9 - 12)	10.04 (± 1.19)	15.75 (4 - 31)	15.75 (± 6.71)	18 (14 - 26)	18.4 (± 4.72)
Testicular	[C] Cadmium chloride	9.5 (9 - 13)	10 (± 1.41)	10 (8 - 13)	9.9 (± 1.29)	12 (10 - 14)	12.2 (± 1.79)
Testicular	[D] Cadmium chloride	8.75 (5.5 - 12)	8.66 (± 1.79)	10.25 (7 - 12)	9.67 (± 2.06)	15 (11 - 19)	14.7 (± 3.27)
Testicular	[D] Carbendazim	7.75 (6 - 18)	9.27 (± 3.81)	11.5 (7.2 - 28)	13.09 (± 5.94)	14 (10 - 22)	14.8 (± 4.6)
Testicular	[D] Di-n-pentyl-phthalate	7.9 (6 - 11.5)	8.22 (± 1.73)	10.75 (9.5 - 15.2)	11.39 (± 2.05)	17.4 (15.2 - 18)	17.1 (± 1.09)
Testicular	[D] Ethane dimethane sulfonate (EDS)	15 (11 - 20)	15.5 (± 2.64)	12.5 (9.1 - 17)	12.59 (± 2.8)	14 (10 - 18)	13.8 (± 2.86)
Testicular	[D] Methoxyacetic acid	7.45 (6.4 - 12)	8.19 (± 1.85)	11 (8.2 - 18)	11.34 (± 2.79)	13 (8.9 - 15.4)	12.76 (± 2.37)
Multiple organ	[B] Adriamycin	9.3 (5.4 - 16)	9.67 (± 3.18)	11.55 (3.6 - 15)	10.73 (± 3.43)	11 (10.4 - 15)	12.4 (± 2.21)
Multiple organ	[C] Amphotericin B	10 (6 - 12)	10.1 (± 1.79)	10 (8.5 - 13)	10.45 (± 1.64)	12 (7 - 33)	15.4 (± 10.16)
Multiple organ	[C] Azaserine	10 (9 - 13)	10.7 (± 1.25)	12 (10 - 34)	14.6 (± 7.14)	12 (10 - 16)	12.6 (± 2.19)
Multiple organ	[A] Dexamethasone	10.5 (6 - 14)	10.13 (± 2.43)	14 (8 - 16)	12.6 (± 3.27)	14 (12 - 18)	14.8 (± 2.28)
Multiple organ	[E] Mitomycin-C	13 (8.5 - 18)	12.8 (± 3.45)	17.5 (11 - 49)	20.7 (± 11.73)	13 (12 - 18)	14.4 (± 2.51)
Physiological stressor	[C] 1,1-Dichloroethylene & maleic acid	10 (8 - 12)	9.9 (± 1.45)	12 (8 - 19)	12.4 (± 3.2)	13 (11 - 18)	13.8 (± 2.77)
Physiological stressor	[C] 2,4-Dinitrophenol	10.5 (7 - 17)	10.7 (± 2.71)	11.5 (9 - 17)	11.6 (± 2.27)	15 (13 - 18)	15.4 (± 2.07)
Physiological stressor	[B] 4-Pentenoic acid	10.2 (4 - 16)	9.37 (± 3.94)	12 (2.4 - 22)	11.34 (± 5.57)	9.8 (5.6 - 13)	9.44 (± 2.64)
Physiological stressor	[D] Acetazolamide	9.35 (5 - 18)	10.38 (± 3.53)	12 (10 - 18)	12.7 (± 2.45)	11 (9 - 14)	11.4 (± 1.82)
Physiological stressor	[C] Acivicin	9 (7 - 14)	9.7 (± 2.26)	11 (9 - 15)	11.4 (± 2.01)	12 (8 - 13)	11.6 (± 2.07)
Physiological stressor	[E] Ammonium chloride	12.5 (8 - 17)	11.85 (± 3.06)	12.5 (9.5 - 15)	12.35 (± 2.43)	11.59 (10.24 - 14.95)	12.34 (± 1.98)
Physiological stressor	[D] Carboplatin	10 (9.1 - 14)	10.75 (± 1.64)	12 (8.2 - 17)	12.52 (± 2.76)	14 (9 - 16)	12.8 (± 2.77)
Physiological stressor	[A] Choline and choline/methionine deficiency (chronic)	9.1 (7.9 - 14.4)	10.03 (± 2.18)	10 (7.4 - 17)	11.16 (± 3.13)	12 (11 - 15)	12.8 (± 1.64)
Physiological stressor	[B] Food restriction (chronic)	9.4 (2.8 - 13.2)	8.98 (± 2.83)	13.5 (6 - 15)	11.61 (± 3.2)	12.8 (10 - 20.2)	14.44 (± 4.04)

Target organ	Toxin	24 h post dose		48 h post dose		168 h post dose	
		Median (min - max)	Mean (± SD)	Median (min - max)	Mean (± SD)	Median (min - max)	Mean (± SD)
Physiological stressor	[D] Furosemide	7.8 (5.2 - 13)	8.34 (± 2.56)	10 (5.8 - 15)	10.12 (± 2.95)	14 (10 - 20)	14.6 (± 3.97)
Physiological stressor	[B] Insulin	12.15 (8.8 - 14.6)	12 (± 1.92)	14.35 (5.8 - 17.2)	13.72 (± 3.59)	13.6 (9.8 - 18.4)	14.12 (± 3.26)
Physiological stressor	[E] Methotrexate	9.75 (7.5 - 16)	10.35 (± 2.57)	13.5 (9 - 16)	12.75 (± 2.68)	14 (12 - 18)	14.1 (± 2.36)
Physiological stressor	[A] Partial hepatectomy	7.5 (5.5 - 15.5)	8.5 (± 3.14)	8.25 (5.5 - 18.5)	9.75 (± 4.34)	12 (9.5 - 22)	13.6 (± 4.87)
Physiological stressor	[A] Phenobarbital (chronic)	9 (7 - 14)	9.9 (± 2.13)	10 (6 - 20)	11.6 (± 4.5)	15 (10 - 19)	14.8 (± 3.27)
Physiological stressor	[A] Pregnenolone 16 alpha carbonitrile (chronic)	12 (9 - 17)	12.2 (± 2.39)	13.5 (9 - 16)	12.9 (± 2.33)	14 (12 - 19)	14.8 (± 3.11)
Physiological stressor	[A] Probenecid	11.1 (2.7 - 14.6)	10.55 (± 3.17)	12.1 (10.1 - 17.2)	12.7 (± 2.14)	15.2 (10.8 - 17.8)	15.04 (± 2.65)
Physiological stressor	[C] Rosiglitazone	11 (2 - 15)	9.6 (± 4.5)	12 (8 - 20)	12.5 (± 3.21)	12 (10 - 20)	13.4 (± 3.97)
Physiological stressor	[C] Rosiglitazone (chronic)	10 (7 - 12)	9.95 (± 1.57)	11.5 (5 - 16)	11.6 (± 3.27)	12 (12 - 14)	12.4 (± 0.89)
Physiological stressor	[E] Sodium bicarbonate	9.5 (8 - 15)	10.1 (± 2)	10.75 (8 - 50)	16.1 (± 12.75)	15 (11 - 21)	15.7 (± 3.5)
Physiological stressor	[A] Unilateral nephrectomy	7 (5 - 9.5)	7.1 (± 1.54)	8.75 (4.5 - 11.5)	8.4 (± 2.14)	15.5 (10.5 - 22.5)	16.1 (± 4.76)
Physiological stressor	[B] Water deprivation (chronic)	10.41 (8 - 15.6)	10.73 (± 2.18)	13.7 (9.6 - 20.8)	14.2 (± 3.13)	13.4 (11.2 - 15.2)	13.09 (± 1.49)
No Effect	[E] Acetaminophen (chronic)	10 (8 - 10)	9.6 (± 0.7)	10 (7 - 20)	11.1 (± 3.61)	15.5 (11.5 - 25)	16.8 (± 4.67)
No Effect	[C] Buthionine sulphoxime	12 (8 - 14)	11.5 (± 1.78)	12.5 (6 - 16)	12.2 (± 2.7)	14 (10 - 20)	14 (± 3.74)
No Effect	[C] Ferrous sulphate	11 (9 - 17)	11.7 (± 2.67)	14 (8 - 17)	13.32 (± 2.6)	15 (11 - 18)	15 (± 2.74)
No Effect	[B] Ifosfamide	8.9 (5.4 - 22.6)	10.22 (± 5.02)	13.3 (8.6 - 33.6)	15.03 (± 7.09)	15.4 (11 - 31.8)	18.16 (± 7.99)
No Effect	[B] Lithocholic acid	10.5 (3 - 14.2)	9.86 (± 2.97)	13.9 (9.4 - 26)	14.98 (± 4.48)	16.2 (8.6 - 16.6)	14.24 (± 3.41)
No Effect	[E] Paraquat	10.5 (7.25 - 13.75)	10.47 (± 2.19)	13.38 (9 - 18.5)	13.22 (± 2.86)	18 (12 - 21)	16.2 (± 4.02)
No Effect	[D] Potassium dichromate	11 (4 - 15)	10.8 (± 3.33)	14.5 (10 - 22)	14.7 (± 3.77)	16 (14 - 21)	16.6 (± 2.7)
No Effect	[C] Trichlorethylene	10.5 (8 - 15)	10.6 (± 2.22)	12 (8 - 16)	11.8 (± 2.74)	14 (6 - 18)	13 (± 4.47)

A-F: Indicates Pharmaceutical Company & sample origin

Supplementary Table S16. Summary statistics for urine pH at 24 hrs, 48 hrs and 168 hrs post dose vehicle.

Target organ	Toxin	24 h post dose		48 h post dose		168 h post dose	
		Median (min - max)	Mean (± SD)	Median (min - max)	Mean (± SD)	Median (min - max)	Mean (± SD)
Liver	E 1,1-Dichloroethylene	6.3 (6.1 - 6.6)	6.34 (± 0.16)	7.15 (6.6 - 7.5)	7.11 (± 0.31)	7.1 (6.8 - 7.6)	7.22 (± 0.33)
Liver	E 1,2,3,4,5,6-hexachlorocyclohexane	6.85 (6.4 - 7.5)	6.87 (± 0.37)	7 (6.6 - 7.5)	7.02 (± 0.31)	7 (6.9 - 7.4)	7.08 (± 0.19)
Liver	B 1-Fluoropentane	7.5 (7 - 8)	7.5 (± 0.53)	8 (7 - 8)	7.6 (± 0.52)	8 (7 - 8)	7.6 (± 0.55)
Liver	B 2,4,6-Trihydroxyacetophenone (THA)	7 (6.5 - 8)	7 (± 0.41)	7 (6.5 - 8)	7.05 (± 0.37)	7 (6.5 - 7)	6.8 (± 0.27)
Liver	B 4-Amino-2,6-dichlorophenol (ADCP)	7.27 (7 - 7.5)	7.24 (± 0.15)	7.3 (7.14 - 7.82)	7.37 (± 0.23)	7.23 (6.78 - 7.8)	7.28 (± 0.37)
Liver	C Aflatoxin	7.5 (7.5 - 8)	7.7 (± 0.26)	7.75 (7.5 - 8.5)	7.8 (± 0.35)	7.5 (7 - 8.5)	7.5 (± 0.61)
Liver	C Allyl alcohol	7.75 (7 - 8)	7.7 (± 0.35)	7.5 (7.5 - 8.5)	7.75 (± 0.42)	7.5 (7 - 7.5)	7.32 (± 0.25)
Liver	C Allyl formate	7.5 (7.5 - 8)	7.6 (± 0.21)	7.5 (7 - 8)	7.6 (± 0.32)	7.5 (7.5 - 8.5)	7.7 (± 0.45)
Liver	B Azathioprine	7.27 (7 - 7.5)	7.24 (± 0.15)	7.3 (7.14 - 7.82)	7.37 (± 0.23)	7.23 (6.78 - 7.8)	7.28 (± 0.37)
Liver	B Bromobenzene	6 (6 - 6.5)	6.15 (± 0.24)	7 (7 - 8)	7.3 (± 0.48)	8 (7 - 8)	7.8 (± 0.45)
Liver	C Butylated hydroxytoluene	6.5 (6.5 - 6.5)	6.5 (± 0)	7 (7 - 8)	7.25 (± 0.35)	7.5 (7 - 8)	7.5 (± 0.35)
Liver	D Carbon tetrachloride	6 (5 - 7)	6 (± 0.47)	7 (7 - 8)	7.4 (± 0.52)	6 (6 - 7)	6.4 (± 0.55)
Liver	C Chlorpromazine	7.5 (7.5 - 8)	7.7 (± 0.26)	7.5 (7 - 8)	7.6 (± 0.39)	7 (7 - 8)	7.3 (± 0.45)
Liver	B Clofibrate	7.5 (7 - 8)	7.5 (± 0.53)	8 (7 - 8)	7.7 (± 0.48)	9 (8 - 9)	8.8 (± 0.45)
Liver	B Cyproterone acetate	7 (6.5 - 8)	7 (± 0.41)	7 (7 - 8)	7.4 (± 0.52)	7 (7 - 8)	7.2 (± 0.45)
Liver	A D-galactosamine	6.5 (6 - 7)	6.65 (± 0.34)	6.75 (6.5 - 7.5)	6.8 (± 0.35)	7 (6.5 - 7.5)	7 (± 0.35)
Liver	B Diethylhexylphthalate (DEHP)	7 (7 - 8)	7.4 (± 0.52)	8 (7 - 8)	7.6 (± 0.52)	8 (7 - 8)	7.8 (± 0.45)
Liver	C Dimethylformamide (DMF)	7.25 (7 - 7.5)	7.25 (± 0.26)	7 (6.5 - 8)	7.1 (± 0.39)	7 (6.5 - 7.5)	7 (± 0.35)
Liver	C Dimethylnitrosamine (DMN)	7.5 (7 - 8)	7.35 (± 0.34)	7.5 (7 - 8)	7.54 (± 0.28)	7 (7 - 8)	7.3 (± 0.45)

Target organ	Toxin	24 h post dose		48 h post dose		168 h post dose	
		Median (min - max)	Mean (± SD)	Median (min - max)	Mean (± SD)	Median (min - max)	Mean (± SD)
Liver	[A] Gadolinium chloride	7 (6.5 - 7.5)	7 (± 0.33)	7 (6.5 - 8)	7.1 (± 0.39)	7.5 (7 - 7.5)	7.3 (± 0.27)
Liver	[A, B, C, D, F] Hydrazine	7.53 (7 - 8.5)	7.59 (± 0.48)	7.75 (6.95 - 8.5)	7.76 (± 0.63)	8 (7 - 8.5)	7.81 (± 0.57)
Liver	[E] Hydrazine	7 (7 - 8)	7.1 (± 0.32)	7 (7 - 7)	7 (± 0)	7 (7 - 7)	7 (± 0)
Liver	[E] Indomethacin	7.35 (7 - 7.7)	7.37 (± 0.26)	7.35 (7 - 8.1)	7.37 (± 0.32)	7.4 (7 - 7.6)	7.38 (± 0.23)
Liver	[E] Ketoconazole	7.5 (7.1 - 7.9)	7.53 (± 0.26)	7.4 (7 - 7.7)	7.4 (± 0.25)	7.7 (7.1 - 7.9)	7.6 (± 0.32)
Liver	[C] Lead acetate	8 (7 - 8)	7.75 (± 0.35)	8 (7.5 - 8.5)	8.05 (± 0.28)	8 (7 - 8.5)	7.9 (± 0.55)
Liver	[A] Lipopolysaccharide (LPS)	7 (7 - 7.5)	7.15 (± 0.24)	7 (7 - 7.5)	7.1 (± 0.21)	7 (6.5 - 7.5)	7 (± 0.35)
Liver	[B] Methapyrilene	7.72 (7.1 - 8.1)	7.57 (± 0.38)	7.44 (7.13 - 7.8)	7.42 (± 0.21)	7.6 (7.25 - 7.9)	7.59 (± 0.27)
Liver	[E] Methylene dianiline	6.6 (6.3 - 7.1)	6.63 (± 0.22)	7.55 (7.2 - 8)	7.56 (± 0.22)	7.6 (7.5 - 7.8)	7.62 (± 0.11)
Liver	[C] Monocrotaline	7.75 (7.5 - 8)	7.75 (± 0.26)	7.5 (6.5 - 7.5)	7.35 (± 0.34)	7.5 (7.5 - 8)	7.6 (± 0.22)
Liver	[C] N-methylformamide (NMF)	7.5 (7 - 8)	7.5 (± 0.41)	7.5 (6.5 - 8)	7.35 (± 0.47)	7.5 (7.5 - 8)	7.7 (± 0.27)
Liver	[D] Phalloidin (chronic)	7.16 (6.91 - 7.44)	7.19 (± 0.16)	7.21 (6.94 - 7.35)	7.19 (± 0.12)	7.13 (6.9 - 7.21)	7.09 (± 0.12)
Liver	[E] Phenyl diisothiocyanate	6.8 (6 - 7.3)	6.8 (± 0.38)	6.8 (6.4 - 7.3)	6.84 (± 0.29)	7.1 (6.6 - 7.4)	7.04 (± 0.3)
Liver	[E] Phenyl isothiocyanate	6.55 (6.1 - 7.5)	6.69 (± 0.42)	6.85 (6.7 - 7.5)	6.95 (± 0.31)	7.2 (6.7 - 7.6)	7.14 (± 0.35)
Liver	[B] Retinyl palmitate	7.27 (7 - 7.5)	7.24 (± 0.15)	7.3 (7.14 - 7.82)	7.37 (± 0.23)	7.23 (6.78 - 7.8)	7.28 (± 0.37)
Liver	[B] Sodium Valproate	8 (7 - 8)	7.6 (± 0.52)	8 (7 - 9)	8.1 (± 0.57)	8 (7 - 8)	7.8 (± 0.45)
Liver	[C] a-Naphthylisothiocyanate (ANIT)	6.5 (6.5 - 7)	6.55 (± 0.16)	7.5 (7 - 7.5)	7.3 (± 0.26)	7.5 (7.5 - 8)	7.6 (± 0.22)
Kidney	[D] 2-Bromophenol	7.07 (6.92 - 7.35)	7.1 (± 0.14)	7.26 (7.17 - 7.6)	7.3 (± 0.14)	7.32 (7.22 - 7.35)	7.29 (± 0.06)
Kidney	[E] 3,5-Dichloroaniline hydrochloride	7.35 (7 - 7.7)	7.36 (± 0.27)	7.25 (6.9 - 7.5)	7.23 (± 0.21)	7.4 (7.2 - 7.6)	7.4 (± 0.14)
Kidney	[E] Atractyloside	7.4 (7 - 7.6)	7.35 (± 0.2)	7.4 (7.1 - 7.6)	7.38 (± 0.15)	7.4 (7.1 - 7.7)	7.42 (± 0.24)
Kidney	[D] Bromoethylamine hydrobromide	7.3 (6.9 - 7.5)	7.25 (± 0.2)	6.9 (6.3 - 7.4)	6.92 (± 0.29)	7 (6.9 - 7.3)	7.02 (± 0.16)

Target organ	Toxin	24 h post dose		48 h post dose		168 h post dose	
		Median (min - max)	Mean (± SD)	Median (min - max)	Mean (± SD)	Median (min - max)	Mean (± SD)
Kidney	[D] Cephaloridine	7.35 (6.85 - 7.75)	7.37 (± 0.31)	7.24 (6.84 - 7.56)	7.24 (± 0.19)	7.41 (7.23 - 7.53)	7.37 (± 0.12)
Kidney	[B] Chlorethanamine	7 (7 - 8)	7.3 (± 0.48)	7 (7 - 8)	7.4 (± 0.52)	7 (7 - 8)	7.4 (± 0.55)
Kidney	[A] Cisplatin	7.24 (6.49 - 7.62)	7.17 (± 0.34)	7.06 (6.56 - 7.52)	7.04 (± 0.32)	6.9 (6.76 - 7.26)	6.99 (± 0.22)
Kidney	[A] D-limonene (chronic)	6.58 (6.23 - 7.03)	6.59 (± 0.21)	7.27 (6.99 - 7.54)	7.27 (± 0.16)	7.36 (7.28 - 7.69)	7.42 (± 0.16)
Kidney	[E] Dichlorophenyl succinimide	6.6 (6.3 - 7.1)	6.63 (± 0.22)	7.55 (7.2 - 8)	7.56 (± 0.22)	7.6 (7.5 - 7.8)	7.62 (± 0.11)
Kidney	[D] Ethylene glycol	7.34 (6.86 - 7.79)	7.3 (± 0.27)	7.16 (6.88 - 7.39)	7.12 (± 0.2)	7.19 (6.98 - 7.39)	7.16 (± 0.16)
Kidney	[A] Folic acid	7.44 (6.85 - 7.55)	7.34 (± 0.25)	7.28 (6.85 - 7.46)	7.24 (± 0.22)	7.16 (6.79 - 7.39)	7.13 (± 0.22)
Kidney	[A] Gentamicin	7.22 (6.84 - 7.61)	7.28 (± 0.23)	7.1 (6.77 - 7.55)	7.15 (± 0.25)	7.02 (6.03 - 7.5)	6.8 (± 0.57)
Kidney	[B] Maleic acid	7.27 (7 - 7.5)	7.24 (± 0.15)	7.3 (7.14 - 7.82)	7.37 (± 0.23)	7.23 (6.78 - 7.8)	7.28 (± 0.37)
Kidney	[A] N-phenylanthranilic acid (chronic)	7 (7 - 8)	7.15 (± 0.34)	7.5 (7 - 8)	7.5 (± 0.24)	7.5 (7.5 - 7.5)	7.5 (± 0)
Kidney	[D] Para-aminophenol	7.3 (6.35 - 7.51)	7.22 (± 0.33)	7.18 (6.4 - 7.35)	7.1 (± 0.27)	6.89 (6.38 - 7.09)	6.85 (± 0.28)
Kidney	[A] Puromycin	7.06 (6.78 - 7.52)	7.11 (± 0.21)	7.08 (6.8 - 7.5)	7.1 (± 0.2)	7.3 (6.88 - 7.34)	7.19 (± 0.2)
Kidney	[B] Vancomycin hydrochloride	7 (6.5 - 8)	7.35 (± 0.58)	7 (7 - 8)	7.4 (± 0.52)	7 (7 - 7)	7 (± 0)
Liver & Kidney	[E] Acetaminophen	7.05 (6.8 - 7.4)	7.09 (± 0.19)	7 (6.9 - 7.2)	7.03 (± 0.13)	7.1 (6.8 - 7.5)	7.12 (± 0.29)
Liver & Kidney	[B] Aurothiomalate	7.27 (7 - 7.5)	7.24 (± 0.15)	7.3 (7.14 - 7.82)	7.37 (± 0.23)	7.23 (6.78 - 7.8)	7.28 (± 0.37)
Liver & Kidney	[C] Chloroform	6.5 (6 - 7.5)	6.65 (± 0.47)	7.5 (7 - 8)	7.55 (± 0.44)	7.46 (7 - 8)	7.39 (± 0.42)
Liver & Kidney	[D] Cyclosporin	6.88 (6.5 - 7.51)	6.95 (± 0.32)	7.48 (7.05 - 7.8)	7.46 (± 0.22)	7.2 (7.05 - 7.63)	7.28 (± 0.24)
Liver & Kidney	[D] Dichlorobenzene	6.84 (6.5 - 7.11)	6.84 (± 0.19)	7.5 (7.16 - 7.97)	7.47 (± 0.26)	7.11 (7.01 - 7.32)	7.17 (± 0.14)
Liver & Kidney	[C] Ethionine	7 (7 - 8)	7.15 (± 0.34)	7.5 (7 - 8)	7.35 (± 0.34)	7 (7 - 7.5)	7.2 (± 0.27)
Liver & Kidney	[B] Hexachlorobutadiene (HCBD)	6.5 (6 - 7)	6.45 (± 0.44)	8 (7 - 9)	8.2 (± 0.63)	8 (7 - 9)	8 (± 1)
Liver & Kidney	[B] Mercuric chloride	7.5 (7 - 8)	7.5 (± 0.53)	8 (7 - 8)	7.7 (± 0.48)	8 (8 - 8.5)	8.1 (± 0.22)

Target organ	Toxin	24 h post dose		48 h post dose		168 h post dose	
		Median (min - max)	Mean (± SD)	Median (min - max)	Mean (± SD)	Median (min - max)	Mean (± SD)
Liver & Kidney	[E] Microcystin-LR	7.6 (7.3 - 7.9)	7.57 (± 0.2)	7.45 (7 - 7.9)	7.45 (± 0.27)	7.5 (7.2 - 7.7)	7.48 (± 0.19)
Liver & Kidney	[E] Rotenone	6.6 (6.1 - 6.8)	6.56 (± 0.2)	7.4 (7.1 - 7.8)	7.45 (± 0.25)	7.3 (7.2 - 7.6)	7.36 (± 0.15)
Liver & Kidney	[E] S-(1,2-dichlorovinyl)-cysteine (DCVC)	7.05 (6.8 - 7.6)	7.17 (± 0.31)	7.2 (6.7 - 7.5)	7.19 (± 0.23)	7.4 (7.1 - 7.6)	7.38 (± 0.19)
Liver & Kidney	[D] Thioacetamide	7.42 (7.08 - 7.69)	7.4 (± 0.22)	7.35 (7.14 - 7.72)	7.37 (± 0.17)	7.21 (6.92 - 7.41)	7.19 (± 0.19)
Pancreas	[E] 1-Cyano-2-hydroxy-3-butene	7.5 (7.1 - 7.9)	7.47 (± 0.23)	7.55 (7.2 - 7.8)	7.55 (± 0.21)	7.6 (7.2 - 8)	7.64 (± 0.32)
Pancreas	[C] Caerulin	7.5 (7.5 - 8)	7.65 (± 0.24)	7.5 (7.5 - 8)	7.7 (± 0.26)	8 (7.5 - 8)	7.8 (± 0.27)
Pancreas	[E] L-arginine	7.25 (6.9 - 7.8)	7.33 (± 0.3)	7.1 (6.8 - 7.4)	7.1 (± 0.18)	7.6 (7.1 - 7.8)	7.54 (± 0.3)
Pancreas	[B] Streptozotocin	8 (7 - 8)	7.7 (± 0.48)	8 (7 - 8)	7.8 (± 0.42)	8 (8 - 8)	8 (± 0)
Testicular	[D] 1,3-Dinitrobenzene	6.53 (6.24 - 6.79)	6.54 (± 0.19)	7.24 (6.86 - 7.48)	7.24 (± 0.19)	7.28 (7.15 - 7.3)	7.25 (± 0.06)
Testicular	[C] Cadmium chloride	7.5 (7 - 8)	7.5 (± 0.33)	7.5 (6.5 - 8)	7.5 (± 0.47)	7.5 (7 - 8)	7.6 (± 0.42)
Testicular	[D] Cadmium chloride	7.62 (7.32 - 7.86)	7.62 (± 0.17)	7.52 (7.19 - 7.75)	7.51 (± 0.18)	7.33 (6.93 - 7.54)	7.26 (± 0.23)
Testicular	[D] Carbendazim	6.48 (6.32 - 6.78)	6.54 (± 0.18)	7.27 (6.82 - 7.46)	7.2 (± 0.22)	7.35 (7.21 - 7.42)	7.34 (± 0.09)
Testicular	[D] Di-n-pentyl-phthalate	6.29 (6.1 - 6.78)	6.41 (± 0.25)	7.06 (6.58 - 7.5)	7.02 (± 0.26)	6.92 (6.76 - 7.36)	7.05 (± 0.27)
Testicular	[D] Ethane dimethane sulfonate (EDS)	7.22 (6.95 - 7.4)	7.2 (± 0.15)	7.14 (6.99 - 7.36)	7.18 (± 0.12)	7.36 (7.22 - 7.63)	7.4 (± 0.17)
Testicular	[D] Methoxyacetic acid	7.62 (6.91 - 7.9)	7.56 (± 0.29)	7.31 (6.76 - 7.44)	7.22 (± 0.23)	7.09 (6.91 - 7.5)	7.17 (± 0.28)
Multiple organ	[B] Adriamycin	7 (7 - 8)	7.2 (± 0.42)	7 (7 - 8)	7.27 (± 0.41)	7 (7 - 8)	7.4 (± 0.55)
Multiple organ	[C] Amphotericin B	7.75 (7 - 8)	7.7 (± 0.35)	8 (6.5 - 8)	7.7 (± 0.48)	8 (7 - 8)	7.7 (± 0.45)
Multiple organ	[C] Azaserine	7.75 (7.5 - 8.5)	7.8 (± 0.35)	7.5 (7 - 8)	7.65 (± 0.34)	7 (7 - 7.5)	7.2 (± 0.27)
Multiple organ	[A] Dexamethasone	7.12 (6.7 - 7.54)	7.12 (± 0.31)	7.19 (6.7 - 7.41)	7.15 (± 0.23)	7.27 (7.21 - 7.4)	7.29 (± 0.08)
Multiple organ	[E] Mitomycin-C	7.2 (6.9 - 7.4)	7.16 (± 0.16)	7.25 (6.8 - 7.5)	7.21 (± 0.23)	7.4 (7.3 - 7.5)	7.4 (± 0.07)
Physiological stressor	[C] 1,1-Dichloroethylene & maleic acid	7.5 (7 - 8)	7.45 (± 0.37)	7.75 (7 - 8)	7.7 (± 0.35)	7.5 (7 - 8)	7.4 (± 0.42)

Target organ	Toxin	24 h post dose		48 h post dose		168 h post dose	
		Median (min - max)	Mean (± SD)	Median (min - max)	Mean (± SD)	Median (min - max)	Mean (± SD)
Physiological stressor	[C] 2,4-Dinitrophenol	7.5 (7.5 - 8)	7.7 (± 0.26)	7.5 (6.5 - 8)	7.6 (± 0.46)	7.5 (7.5 - 8)	7.6 (± 0.22)
Physiological stressor	[B] 4-Pentenoic acid	7 (7 - 8)	7.2 (± 0.42)	7 (6.5 - 9)	7.35 (± 0.75)	7 (7 - 7)	7 (± 0)
Physiological stressor	[D] Acetazolamide	7.14 (6.82 - 7.65)	7.15 (± 0.23)	7.04 (6.71 - 7.52)	7.08 (± 0.21)	7.11 (7 - 7.39)	7.16 (± 0.16)
Physiological stressor	[C] Acivicin	7.75 (7 - 8)	7.6 (± 0.46)	7.5 (6.5 - 8)	7.4 (± 0.46)	7.5 (7 - 8)	7.4 (± 0.42)
Physiological stressor	[E] Ammonium chloride	7.4 (6.9 - 7.7)	7.37 (± 0.22)	7.65 (7 - 8.1)	7.57 (± 0.35)	7.25 (7.1 - 7.33)	7.24 (± 0.08)
Physiological stressor	[D] Carboplatin	7.54 (7.12 - 7.73)	7.47 (± 0.25)	7.3 (6.84 - 7.49)	7.23 (± 0.25)	7.46 (7.22 - 7.59)	7.41 (± 0.17)
Physiological stressor	[A] Choline and choline/methionine deficiency (chronic)	7 (6.5 - 7.5)	7.15 (± 0.34)	7 (7 - 7.5)	7.2 (± 0.26)	7.5 (7 - 7.5)	7.3 (± 0.27)
Physiological stressor	[B] Food restriction (chronic)	7 (6 - 7)	6.83 (± 0.35)	7 (6.84 - 8)	7.1 (± 0.34)	7 (7 - 7)	7 (± 0)
Physiological stressor	[D] Furosemide	7.35 (6.64 - 7.69)	7.31 (± 0.3)	7.22 (6.47 - 7.57)	7.13 (± 0.32)	7.26 (6.78 - 7.61)	7.23 (± 0.3)
Physiological stressor	[B] Insulin	7 (6.5 - 8)	7.25 (± 0.54)	7 (7 - 8)	7.4 (± 0.52)	7 (7 - 7)	7 (± 0)
Physiological stressor	[E] Methotrexate	6.95 (6.5 - 7.3)	6.91 (± 0.26)	6.9 (6.7 - 7.3)	6.96 (± 0.21)	7 (6.9 - 7.6)	7.16 (± 0.32)
Physiological stressor	[A] Partial hepatectomy	8 (7.5 - 8)	7.8 (± 0.26)	8 (7.5 - 8)	7.85 (± 0.24)	8 (7.5 - 8)	7.8 (± 0.27)
Physiological stressor	[A] Phenobarbital (chronic)	7.12 (6.44 - 7.65)	7.12 (± 0.37)	7.14 (6.3 - 7.6)	7.03 (± 0.39)	6.83 (6.18 - 7.35)	6.8 (± 0.42)
Physiological stressor	[A] Pregnenolone 16 alpha carbonitrile (chronic)	7.01 (6.65 - 7.4)	6.97 (± 0.22)	7.04 (6.73 - 7.21)	6.99 (± 0.15)	6.98 (6.81 - 7.03)	6.96 (± 0.09)
Physiological stressor	[A] Probenecid	7.5 (7 - 8)	7.55 (± 0.37)	7.5 (7 - 8.5)	7.4 (± 0.46)	7.5 (7 - 8)	7.5 (± 0.35)
Physiological stressor	[C] Rosiglitazone	7.5 (6.75 - 7.5)	7.32 (± 0.29)	7.5 (7 - 8)	7.5 (± 0.41)	7.5 (7 - 7.5)	7.3 (± 0.27)
Physiological stressor	[C] Rosiglitazone (chronic)	7.5 (7 - 8)	7.35 (± 0.34)	7.25 (7 - 8)	7.3 (± 0.35)	7 (6.5 - 7.5)	7 (± 0.35)
Physiological stressor	[E] Sodium bicarbonate	7.2 (6.7 - 7.7)	7.2 (± 0.31)	7.25 (6.9 - 7.5)	7.22 (± 0.19)	7.25 (7 - 7.7)	7.26 (± 0.23)
Physiological stressor	[A] Unilateral nephrectomy	7.5 (7 - 8)	7.4 (± 0.39)	7.5 (7 - 8)	7.35 (± 0.34)	7.5 (7 - 8)	7.5 (± 0.35)
Physiological stressor	[B] Water deprivation (chronic)	7 (6.5 - 8)	7.04 (± 0.38)	7 (6.5 - 8)	7.25 (± 0.49)	7.5 (6.99 - 8)	7.5 (± 0.55)
No Effect	[E] Acetaminophen (chronic)	7.05 (6.6 - 7.6)	7.05 (± 0.3)	7 (6.7 - 7.4)	6.98 (± 0.21)	7.05 (6.8 - 7.4)	7.07 (± 0.18)

Target organ	Toxin	24 h post dose		48 h post dose		168 h post dose	
		Median (min - max)	Mean (± SD)	Median (min - max)	Mean (± SD)	Median (min - max)	Mean (± SD)
No Effect	C Buthionine sulphoxime	7 (6.5 - 7.5)	7.15 (± 0.34)	7.5 (7 - 8)	7.45 (± 0.37)	7 (7 - 8)	7.3 (± 0.45)
No Effect	C Ferrous sulphate	7.25 (7 - 8)	7.35 (± 0.41)	7.5 (7 - 8)	7.6 (± 0.32)	7.5 (7 - 8)	7.5 (± 0.35)
No Effect	B Ifosfamide	7 (7 - 8.5)	7.35 (± 0.58)	8 (7 - 8)	7.6 (± 0.52)	8 (7 - 8.5)	7.9 (± 0.55)
No Effect	B Lithocholic acid	8 (7 - 8.5)	7.7 (± 0.54)	7.5 (7 - 8)	7.5 (± 0.53)	7 (7 - 8)	7.4 (± 0.55)
No Effect	E Paraquat	7.55 (7.1 - 7.8)	7.5 (± 0.19)	7.4 (7.1 - 7.7)	7.42 (± 0.16)	7.6 (7 - 7.8)	7.5 (± 0.3)
No Effect	D Potassium dichromate	7.04 (6.36 - 7.39)	7.03 (± 0.28)	7.1 (6.8 - 7.55)	7.11 (± 0.25)	7.27 (6.98 - 7.55)	7.28 (± 0.22)
No Effect	C Trichlorethylene	7.5 (6.5 - 8.5)	7.45 (± 0.5)	7.5 (7 - 8)	7.6 (± 0.39)	7.5 (7 - 7.5)	7.4 (± 0.22)

A-F: Indicates Pharmaceutical Company & sample origin

Supplementary Table S17. Summary statistics for urine osmolality (mOsm/L) at 24 hrs, 48 hrs and 168 hrs post dose vehicle.

Target organ	Toxin	24 h post dose		48 h post dose		168 h post dose	
		Median (min - max)	Mean (± SD)	Median (min - max)	Mean (± SD)	Median (min - max)	Mean (± SD)
Liver	E 1,1-Dichloroethylene	1886.5 (1462 - 2106)	1840.8 (± 210)	1846.5 (703 - 2354)	1727.9 (± 442.77)	1733 (1445 - 2020)	1724.2 (± 219.66)
Liver	E 1,2,3,4,5,6-hexachlorocyclohexane	1589 (1442 - 1980)	1640.2 (± 191.79)	1596.5 (512 - 2142)	1540.8 (± 410.92)	1714.51 (1482.2 - 2045.5)	1709.54 (± 229.85)
Liver	B 1-Fluoropentane	1772 (1182 - 2180)	1759 (± 285.24)	1533 (1230 - 2110)	1567.4 (± 306.98)	1842 (1081 - 3684)	2029.8 (± 996.6)
Liver	B 2,4,6-Trihydroxyacetophenone (THA)	1916 (1590 - 2188)	1912.2 (± 182.94)	1970 (1682 - 2252)	1955.8 (± 184.91)	1856 (1704 - 2052)	1838.8 (± 144)
Liver	B 4-Amino-2,6-dichlorophenol (ADCP)	1856.93 (1667.3 - 1955.7)	1832.6 (± 100.22)	1837.55 (1525.8 - 1956.1)	1791.93 (± 141.2)	1677.72 (1619.1 - 1983.7)	1751.9 (± 147.41)
Liver	C Aflatoxin	1554.2 (1486 - 1822.7)	1571.69 (± 98.59)	1579.15 (1166.62 - 1904.1)	1589.97 (± 194.99)	1673.87 (1622.93 - 1860.7)	1697.2 (± 95.35)
Liver	C Allyl alcohol	1365.12 (1028.7 - 1603.6)	1391.8 (± 174.27)	1692.4 (1265.3 - 1868.35)	1649.59 (± 243.98)	1818.4 (1708.5 - 1849.5)	1799.25 (± 56.53)
Liver	C Allyl formate	1528.75 (1411.08 - 1764.9)	1534.07 (± 109.53)	1573.1 (1467.83 - 1831.5)	1602.16 (± 129.05)	1501.45 (1207.1 - 1593.02)	1431.26 (± 161.31)
Liver	B Azathioprine	1856.93 (1667.3 - 1955.7)	1832.6 (± 100.22)	1837.55 (1525.8 - 1956.1)	1791.93 (± 141.2)	1677.72 (1619.1 - 1983.7)	1751.9 (± 147.41)
Liver	B Bromobenzene	2222 (1551 - 2555)	2178.7 (± 306.68)	1807 (1667 - 2399)	1896 (± 226.56)	2191 (1896 - 2295)	2138.2 (± 149.48)

Target organ	Toxin	24 h post dose		48 h post dose		168 h post dose	
		Median (min - max)	Mean (± SD)	Median (min - max)	Mean (± SD)	Median (min - max)	Mean (± SD)
Liver	[C] Butylated hydroxytoluene	1575.15 (1315.42 - 1710.5)	1553.74 (± 135.42)	1473.35 (1251.15 - 1756.7)	1514.81 (± 183.54)	1390.92 (1129.9 - 1593.02)	1384.39 (± 171.11)
Liver	[D] Carbon tetrachloride	1766 (1066 - 1985)	1646.15 (± 312.85)	1722.8 (1159 - 1981)	1680.66 (± 265.67)	1754.2 (1412 - 1970)	1704.24 (± 208.02)
Liver	[C] Chlorpromazine	1553.4 (1370 - 1726.1)	1580.39 (± 112.09)	1667.93 (1229.4 - 1853.55)	1640.6 (± 197.63)	1493.37 (1381.3 - 1653)	1502.35 (± 108.99)
Liver	[B] Clofibrate	1671.5 (1115 - 2169)	1704 (± 300.95)	1648 (1155 - 2058)	1601.1 (± 261.59)	1561 (1313 - 1698)	1546 (± 154.06)
Liver	[B] Cyproterone acetate	1903 (1472 - 2502)	1966.4 (± 367.31)	1842 (1608 - 2420)	1916 (± 277.98)	1880 (1572 - 2240)	1848.4 (± 263.61)
Liver	[A] D-galactosamine	1506.5 (1107 - 2174)	1565.7 (± 316.41)	1526 (941 - 1998)	1516.7 (± 285.47)	1488 (1090 - 1772)	1421.6 (± 275.42)
Liver	[B] Diethylhexylphthalate (DEHP)	1752 (1190 - 2091)	1687 (± 262.92)	1515.5 (1271 - 1992)	1573.4 (± 253.72)	1663 (1146 - 1816)	1584.96 (± 255.43)
Liver	[C] Dimethylformamide (DMF)	1555.4 (1456.8 - 1829.4)	1582.15 (± 102.12)	1679.85 (1494.6 - 1770)	1650.3 (± 99.6)	1564.2 (1332.4 - 1678.7)	1543.8 (± 135.38)
Liver	[C] Dimethylnitrosamine (DMN)	1622.55 (1379.8 - 1687.9)	1590.6 (± 107.89)	1525.38 (1284.73 - 1722.2)	1543 (± 132.46)	1413.2 (1331.9 - 1660.9)	1450.52 (± 126.6)
Liver	[A] Gadolinium chloride	1477.5 (966 - 1829)	1433.1 (± 266.26)	1339.5 (887 - 1788)	1353.5 (± 288.19)	1680 (1161 - 1940)	1585.2 (± 293.75)
Liver	[A, B, C, D, F] Hydrazine	1274.6 (393 - 1912)	1163.3 (± 425.4)	1493.1 (492 - 1938)	1459 (± 331.79)	1587 (619 - 2130.98)	1456.52 (± 439.21)
Liver	[E] Hydrazine	1612 (1326 - 2115)	1609.9 (± 231.43)	1851 (756 - 2424)	1787.6 (± 414.49)	1367 (1212 - 2140)	1619.4 (± 461.39)
Liver	[E] Indomethacin	1455.5 (1203 - 1646)	1444.8 (± 146.75)	1483.5 (1277 - 1807)	1539.8 (± 201.34)	1477 (1075 - 1700)	1426.4 (± 256.53)
Liver	[E] Ketoconazole	1579.5 (1356 - 2092)	1664.3 (± 264.03)	1640.5 (1306 - 2306)	1715.4 (± 306.91)	1697 (1345 - 2106)	1675.6 (± 285.34)
Liver	[C] Lead acetate	1510.4 (1200.4 - 1749.7)	1490.33 (± 150.3)	1490.55 (1170.8 - 1858)	1457.27 (± 210)	1385.2 (933 - 1762.1)	1350.16 (± 310.68)
Liver	[A] Lipopolysaccharide (LPS)	1487 (1237 - 1789)	1478.8 (± 178.54)	1295.5 (1016 - 1718)	1315 (± 218.77)	1238 (728 - 1636)	1186 (± 330.59)
Liver	[B] Methapyrilene	1448 (602 - 2044)	1368.1 (± 569.88)	1495 (892 - 1814)	1474.8 (± 244.24)	1636 (1188 - 1780)	1590.8 (± 240.61)
Liver	[E] Methylene dianiline	1782.5 (1164 - 2114)	1729.8 (± 305.34)	1637 (1343 - 1944)	1644.8 (± 187.99)	1762 (1250 - 2084)	1680.4 (± 376.68)
Liver	[C] Monocrotaline	1561.1 (1304.1 - 1776.1)	1548.71 (± 122.27)	1689.7 (1325.53 - 1796.9)	1620.82 (± 169.06)	1543.95 (1320.8 - 1639.8)	1504.31 (± 123.98)
Liver	[C] N-methylformamide (NMF)	1635.75 (1247.8 - 1861.2)	1597.1 (± 194.72)	1542.58 (1266.05 - 1840.1)	1542.85 (± 202.28)	1474.1 (1379.3 - 1753.62)	1523.4 (± 161.46)
Liver	[D] Phalloidin (chronic)	1723.5 (1543 - 2160)	1806.5 (± 227.64)	1814.5 (1445 - 2065)	1778.2 (± 187.04)	1822.3 (1685.7 - 1869.6)	1784.38 (± 79.81)
Liver	[E] Phenyl diisothiocyanate	1785 (1144 - 2084)	1739.6 (± 297.53)	1838.5 (1172 - 2208)	1800 (± 363.36)	1680 (1217 - 2152)	1701.2 (± 342.65)

Target organ	Toxin	24 h post dose		48 h post dose		168 h post dose	
		Median (min - max)	Mean (± SD)	Median (min - max)	Mean (± SD)	Median (min - max)	Mean (± SD)
Liver	[E] Phenyl isothiocyanate	870.5 (565 - 1139)	881.6 (± 190.97)	1813 (1302 - 2286)	1802 (± 270.28)	1787 (1204 - 1955)	1644.2 (± 304.29)
Liver	[B] Retinyl palmitate	1856.93 (1667.3 - 1955.7)	1832.6 (± 100.22)	1837.55 (1525.8 - 1956.1)	1791.93 (± 141.2)	1677.72 (1619.1 - 1983.7)	1751.9 (± 147.41)
Liver	[B] Sodium Valproate	1926 (1623 - 2195)	1897.5 (± 194.66)	1859 (1397 - 2210)	1799.1 (± 266.63)	1772 (1436 - 2066)	1801.4 (± 243.79)
Liver	[C] a-Naphthylisothiocyanate (ANIT)	1678.95 (1428.5 - 1850.67)	1671.82 (± 124.05)	1676.65 (1558 - 1859.6)	1702.59 (± 94.91)	1789.6 (1751.82 - 1795.2)	1783 (± 18.12)
Kidney	[D] 2-Bromophenol	1733.01 (1339 - 1988)	1713 (± 196.69)	1906.45 (1601 - 1947)	1837.97 (± 129.82)	1744.35 (1438 - 1838)	1678.55 (± 157.84)
Kidney	[E] 3,5-Dichloroaniline hydrochloride	1417 (1169 - 2120)	1574.3 (± 342.52)	1652.5 (1209 - 2070)	1622.2 (± 286.73)	1761 (1285 - 1983)	1675.6 (± 318.22)
Kidney	[E] Atractyloside	1527.5 (1131 - 2100)	1591.5 (± 289.3)	1602 (1375 - 2000)	1642.3 (± 217.26)	1370 (903 - 1666)	1361.4 (± 283.83)
Kidney	[D] Bromoethylamine hydrobromide	1265 (995 - 1665)	1312.3 (± 247.39)	1592.5 (979 - 1906)	1528.6 (± 287.33)	1744.63 (1463 - 1775)	1691.68 (± 128.84)
Kidney	[D] Cephaloridine	1494.5 (1069 - 1914)	1483.32 (± 304.28)	1824.25 (887 - 1987)	1663.35 (± 345.54)	1485 (1384 - 1683)	1511.6 (± 123.46)
Kidney	[B] Chlorethanamine	1720 (1102 - 1916)	1647.1 (± 245.23)	1597.5 (1166 - 1884)	1559.5 (± 210.86)	1537 (1282 - 1916)	1525 (± 247.63)
Kidney	[A] Cisplatin	1509.5 (1100 - 1924)	1556.96 (± 237.6)	1818.37 (1208 - 1896)	1741.1 (± 207.54)	1787 (1721 - 1906)	1804.75 (± 70.67)
Kidney	[A] D-limonene (chronic)	1759.33 (1162 - 1958)	1695.19 (± 247.87)	1560.8 (1009 - 1872)	1520.79 (± 282.04)	1611.7 (1063 - 1964)	1501.98 (± 376.16)
Kidney	[E] Dichlorophenyl succinimide	1782.5 (1164 - 2114)	1729.8 (± 305.34)	1637 (1343 - 1944)	1644.8 (± 187.99)	1762 (1250 - 2084)	1680.4 (± 376.68)
Kidney	[D] Ethylene glycol	1413 (979 - 1849)	1427.68 (± 265.68)	1332.5 (801 - 1838)	1283.8 (± 340.89)	1820.1 (1282 - 1925.1)	1726.84 (± 255.48)
Kidney	[A] Folic acid	1582.12 (1252 - 1822)	1551.92 (± 212.71)	1537 (1264 - 1875)	1576.46 (± 231.57)	1666 (1500 - 1915.9)	1672.18 (± 159.03)
Kidney	[A] Gentamicin	1431.5 (1111 - 1900)	1509.38 (± 284.82)	1673.5 (1296 - 1878)	1666.62 (± 187.73)	1269 (709 - 1615)	1291.8 (± 367.29)
Kidney	[B] Maleic acid	1856.93 (1667.3 - 1955.7)	1832.6 (± 100.22)	1837.55 (1525.8 - 1956.1)	1791.93 (± 141.2)	1677.72 (1619.1 - 1983.7)	1751.9 (± 147.41)
Kidney	[A] N-phenylanthranilic acid (chronic)	1296 (762 - 1626)	1252.2 (± 317.02)	1309.5 (879 - 1698)	1329.8 (± 249.6)	1153 (1040 - 1770)	1285 (± 294.66)
Kidney	[D] Para-aminophenol	1313 (995 - 1690)	1339.35 (± 215)	1431 (1151 - 1914)	1486.82 (± 276.49)	1650 (1503 - 1962)	1712.82 (± 193.19)
Kidney	[A] Puromycin	1448.5 (684 - 1874)	1456.2 (± 369.37)	1762.14 (1511 - 1957)	1746.15 (± 141.89)	1748 (1536 - 1926)	1734 (± 151.48)
Kidney	[B] Vancomycin hydrochloride	1568.5 (978 - 2355)	1601 (± 445.15)	1609 (799 - 1850)	1502.9 (± 342.84)	1471 (1456 - 1654)	1539 (± 101.09)
Liver & Kidney	[E] Acetaminophen	1609.5 (760 - 2126)	1526 (± 398.7)	1468.5 (937 - 2196)	1493.7 (± 317.08)	1385 (1058 - 1932)	1492.6 (± 399.45)

Target organ	Toxin	24 h post dose		48 h post dose		168 h post dose	
		**Median (min - max)**	**Mean (± SD)**	**Median (min - max)**	**Mean (± SD)**	**Median (min - max)**	**Mean (± SD)**
Liver & Kidney	[B] Aurothiomalate	1856.93 (1667.3 - 1955.7)	1832.6 (± 100.22)	1837.55 (1525.8 - 1956.1)	1791.93 (± 141.2)	1677.72 (1619.1 - 1983.7)	1751.9 (± 147.41)
Liver & Kidney	[C] Chloroform	1547 (1422.8 - 1726.1)	1561.04 (± 95.07)	1538.58 (840.1 - 1815.4)	1486.8 (± 273.86)	1378.4 (1089.9 - 1791.4)	1411.06 (± 300.89)
Liver & Kidney	[D] Cyclosporin	1616.8 (1072 - 1926)	1574.61 (± 255.41)	1742.43 (1031 - 1952)	1626.37 (± 316.61)	1524 (1398 - 1699)	1542.2 (± 137.83)
Liver & Kidney	[D] Dichlorobenzene	1832.28 (1541.7 - 1954)	1769.88 (± 172.15)	1811.4 (1657 - 1983)	1825.64 (± 107.4)	1790.63 (1713 - 1889)	1801.51 (± 80.45)
Liver & Kidney	[C] Ethionine	1606 (1537.4 - 1792.75)	1624.82 (± 85.34)	1701.97 (1229.4 - 1858)	1633.06 (± 207.22)	1756.22 (1461.7 - 1783.2)	1698.6 (± 133.52)
Liver & Kidney	[B] Hexachlorobutadiene (HCBD)	1890 (1450 - 2148)	1831.6 (± 253.06)	1649 (1229 - 1851)	1566.4 (± 222.91)	1434 (1028 - 1864)	1491.2 (± 338.54)
Liver & Kidney	[B] Mercuric chloride	1885 (1183 - 2548)	1853.8 (± 358.71)	1904.5 (1197 - 2518)	1915 (± 350.93)	1547 (1074 - 1992)	1574.2 (± 343.45)
Liver & Kidney	[E] Microcystin-LR	1494.5 (1214 - 1865)	1510 (± 197.6)	1582 (1073 - 1929)	1574.6 (± 244.09)	1729 (1596 - 2218)	1813.2 (± 242.01)
Liver & Kidney	[E] Rotenone	1720.5 (1360 - 2072)	1719.5 (± 211.06)	1474.5 (1042 - 2006)	1534.4 (± 286.89)	1638 (1169 - 1985)	1603 (± 292.05)
Liver & Kidney	[E] S-(1,2-dichlorovinyl)-cysteine (DCVC)	1570.5 (1431 - 1996)	1606.5 (± 170.02)	1535.5 (1380 - 1786)	1562 (± 129.91)	1493 (1252 - 1849)	1565.8 (± 241.26)
Liver & Kidney	[D] Thioacetamide	1195.5 (872 - 1643)	1264.4 (± 256.74)	1556 (1172 - 1983)	1565.4 (± 282.83)	1078 (866 - 1287)	1088.8 (± 168.56)
Pancreas	[E] 1-Cyano-2-hydroxy-3-butene	1501 (1263 - 1849)	1526.8 (± 159.13)	1523.5 (1331 - 1927)	1588 (± 217.21)	1636 (1340 - 1910)	1618.4 (± 206.92)
Pancreas	[C] Caerulin	1503.8 (1258.3 - 1671.7)	1498.01 (± 113.25)	1621.35 (1538.2 - 1847.18)	1636.88 (± 84.72)	1575.7 (1174.3 - 1659.12)	1462.97 (± 211.04)
Pancreas	[E] L-arginine	1651.5 (1157 - 1933)	1590.5 (± 278.55)	1647 (1116 - 1925)	1599.3 (± 266.98)	1625 (1033 - 1884)	1556 (± 332.83)
Pancreas	[B] Streptozotocin	1806 (1490 - 2035)	1793.5 (± 156.87)	1604 (1371 - 1825)	1594.2 (± 147.69)	1796 (1473 - 1955)	1733.6 (± 183.18)
Testicular	[D] 1,3-Dinitrobenzene	1767.6 (1053 - 1888.3)	1677.95 (± 267.85)	1694 (1143 - 1872)	1625.4 (± 223.9)	1470 (1156 - 1782)	1451.8 (± 240.99)
Testicular	[C] Cadmium chloride	1575.58 (1342.47 - 1818.1)	1590.65 (± 128.95)	1736.65 (1420.75 - 1853.55)	1696.07 (± 147.78)	1567.92 (1444.5 - 1789.42)	1622.27 (± 156.21)
Testicular	[D] Cadmium chloride	1715 (1386 - 2125)	1748.4 (± 263.47)	1973 (1520 - 2147)	1902.1 (± 220.73)	1715 (1348 - 2036)	1715 (± 300.34)
Testicular	[D] Carbendazim	1892.3 (995 - 1991)	1741.41 (± 368.28)	1808.05 (981 - 1996)	1725.67 (± 311.42)	1751.2 (1257 - 1922.55)	1707.35 (± 272.36)
Testicular	[D] Di-n-pentyl-phthalate	1682 (1290 - 2102)	1704 (± 275.4)	1718.5 (1273 - 2332)	1781.2 (± 372.49)	1556 (1367 - 1717)	1550 (± 150.62)
Testicular	[D] Ethane dimethane sulfonate (EDS)	1799 (1358 - 1950)	1741.9 (± 212.84)	1675.53 (1513 - 1937.1)	1700.1 (± 144.78)	1831 (1399 - 1982)	1750.08 (± 220.17)
Testicular	[D] Methoxyacetic acid	1796.5 (1057 - 2296)	1732.8 (± 325.09)	1547 (809 - 2004)	1535.2 (± 298.47)	1710 (1234 - 2103)	1670.8 (± 324.39)

Target organ	Toxin	24 h post dose		48 h post dose		168 h post dose	
		Median (min - max)	Mean (± SD)	Median (min - max)	Mean (± SD)	Median (min - max)	Mean (± SD)
Multiple organ	[B] Adriamycin	1669.5 (1115 - 2126)	1633.9 (± 286.18)	1703.85 (1401 - 1864)	1675.07 (± 159.55)	1716 (1410 - 1795)	1654.2 (± 161.67)
Multiple organ	[C] Amphotericin B	1551.51 (1425 - 1681.15)	1545.49 (± 95.27)	1609.96 (1563.08 - 1831.5)	1647.07 (± 98.35)	1666.5 (799.4 - 1795.7)	1505.11 (± 401.02)
Multiple organ	[C] Azaserine	1532.3 (1137 - 1558.44)	1455.78 (± 144.91)	1565.43 (734.1 - 1845.9)	1494.69 (± 296.48)	1640.7 (1571.2 - 1804.3)	1659.02 (± 86.61)
Multiple organ	[A] Dexamethasone	1643.5 (1287 - 1794)	1572.27 (± 186.59)	1695.9 (1440 - 1953)	1675.83 (± 185.68)	1737.2 (1294 - 1923)	1679.84 (± 246.48)
Multiple organ	[E] Mitomycin-C	1582 (1077 - 2124)	1513.4 (± 371.82)	1287 (512 - 2026)	1314.9 (± 480)	1693 (1424 - 1863)	1645.8 (± 193.9)
Physiological stressor	[C] 1,1-Dichloroethylene & maleic acid	1567.45 (1445.2 - 1685.15)	1566.22 (± 77.34)	1488.56 (898.5 - 1868.35)	1495.33 (± 293.33)	1578.69 (1308.35 - 1721.3)	1540.47 (± 163.85)
Physiological stressor	[C] 2,4-Dinitrophenol	1544.62 (1290.35 - 1771.97)	1533.56 (± 151.92)	1560.35 (1372 - 1846.6)	1577.38 (± 175.34)	1361.3 (1222 - 1506.85)	1381.26 (± 114.05)
Physiological stressor	[B] 4-Pentenoic acid	1364 (764 - 1954)	1417.6 (± 439.38)	1680 (1335 - 1874)	1654.4 (± 149.91)	2034 (1803 - 2436)	2089.8 (± 237.72)
Physiological stressor	[D] Acetazolamide	1721.5 (1044 - 1965)	1618.67 (± 297.06)	1794.4 (1345 - 1980)	1736.78 (± 212.16)	1867 (1775.97 - 1942)	1866.95 (± 72.62)
Physiological stressor	[C] Acivicin	1705.8 (1310.8 - 1792)	1631.48 (± 163.36)	1562.12 (1388.3 - 1788.1)	1586.13 (± 127.1)	1759.5 (1647 - 1839.02)	1748.97 (± 78.16)
Physiological stressor	[E] Ammonium chloride	1556 (1149 - 1828)	1560.6 (± 223.15)	1608 (1341 - 1859)	1629.2 (± 173.19)	1863.1 (1558.95 - 1979.5)	1803.11 (± 187.01)
Physiological stressor	[D] Carboplatin	1710.15 (1244 - 1944)	1663.19 (± 233.33)	1812 (1219 - 1962)	1720.79 (± 238.99)	1835 (1562 - 1965.1)	1818.54 (± 162.75)
Physiological stressor	[A] Choline and choline/methionine deficiency (chronic)	1471 (1010 - 1844)	1422.5 (± 264.26)	1433.5 (992 - 1776)	1444.9 (± 258.27)	1505 (1236 - 1742)	1491 (± 180.33)
Physiological stressor	[B] Food restriction (chronic)	1094 (650 - 1493)	1034.22 (± 261.15)	396 (217 - 2173.4)	754.81 (± 741.13)	1628 (1301 - 2069)	1653.8 (± 296.5)
Physiological stressor	[D] Furosemide	1785.73 (1310 - 2062.7)	1774.28 (± 226.84)	1826.35 (1454 - 1994)	1783 (± 154.29)	1796 (1306 - 1957)	1733.4 (± 249.06)
Physiological stressor	[B] Insulin	1418 (1206 - 1914)	1441 (± 197.14)	1339.5 (1127 - 1992)	1385.1 (± 238.87)	1569 (1381 - 1948)	1620.2 (± 209.07)
Physiological stressor	[E] Methotrexate	1674.5 (1308 - 1971)	1675.1 (± 225.42)	1660.5 (1410 - 2078)	1684.4 (± 242.37)	1717 (1412 - 1871)	1681.4 (± 175.42)
Physiological stressor	[A] Partial hepatectomy	1396.5 (770 - 1593)	1331.9 (± 278.35)	1247 (746 - 1720)	1256.1 (± 305.55)	1517 (794 - 1579)	1340.6 (± 327.47)
Physiological stressor	[A] Phenobarbital (chronic)	1725.33 (1451 - 1986)	1720.88 (± 175.35)	1715 (1274 - 1974)	1703.09 (± 228.2)	1835.32 (1658 - 1960.2)	1816.5 (± 124.3)
Physiological stressor	[A] Pregnenolone 16 alpha carbonitrile (chronic)	1549.5 (1191 - 1878)	1577.9 (± 253)	1468 (1260 - 1942)	1568.59 (± 272.77)	1658.3 (1282 - 1887.4)	1624.26 (± 218.93)
Physiological stressor	[A] Probenecid	1417 (540 - 1647)	1347.6 (± 343.3)	1481.5 (1178 - 1799)	1480.9 (± 190.14)	1420 (1121 - 1569)	1371.6 (± 169.18)
Physiological stressor	[C] Rosiglitazone	1503.62 (1290.3 - 1849.2)	1526.37 (± 155.8)	1522.94 (959.3 - 1776.3)	1482.9 (± 213.68)	1510.23 (1225.22 - 1612.53)	1456.39 (± 152.31)

Target organ	Toxin	24 h post dose		48 h post dose		168 h post dose	
		Median (min - max)	Mean (± SD)	Median (min - max)	Mean (± SD)	Median (min - max)	Mean (± SD)
Physiological stressor	C Rosiglitazone (chronic)	1629.2 (1342.6 - 1803.35)	1588.31 (± 130.69)	1576.65 (1254.5 - 1807.1)	1564.74 (± 169.24)	1686.69 (1489.27 - 1863.1)	1670.69 (± 153.03)
Physiological stressor	E Sodium bicarbonate	1533.5 (1199 - 1906)	1557.5 (± 203.33)	1461.5 (305 - 2026)	1310.3 (± 564.38)	1667.5 (1184 - 2226)	1660.7 (± 297.07)
Physiological stressor	A Unilateral nephrectomy	1427.5 (1100 - 1589)	1406.9 (± 159.91)	1388 (977 - 1503)	1337.1 (± 158.01)	1367 (910 - 1626)	1300 (± 322.4)
Physiological stressor	B Water deprivation (chronic)	2105.5 (1450 - 2570)	2048.8 (± 323.41)	1814.5 (1335 - 2225)	1825.74 (± 253.28)	1878.89 (1605 - 1917)	1825.13 (± 122.42)
No Effect	E Acetaminophen (chronic)	1644.57 (1427.78 - 1766)	1622.3 (± 114.69)	1652 (1040 - 2326)	1635.1 (± 349.4)	1570 (1108 - 1895)	1526 (± 275)
No Effect	C Buthionine sulphoxime	1437.8 (1158.7 - 1723.8)	1443.19 (± 147.81)	1570.65 (1340.47 - 1742.2)	1550.07 (± 118.33)	1652.7 (1221.5 - 1813.7)	1604.66 (± 231.88)
No Effect	C Ferrous sulphate	1593.25 (1293 - 1752.4)	1556.27 (± 139.38)	1502.91 (1227.65 - 1858.1)	1538.12 (± 180.55)	1451.35 (1349.2 - 1741.02)	1510.44 (± 158.6)
No Effect	B Ifosfamide	1656 (1276 - 2224)	1732 (± 313.39)	1636 (570 - 2150)	1566.8 (± 495.35)	1534 (992 - 2034)	1466.4 (± 391.6)
No Effect	B Lithocholic acid	1465 (590 - 1808)	1359.59 (± 360.05)	1402 (856 - 1708)	1396 (± 231.71)	1546 (1450 - 1822)	1584.4 (± 143.15)
No Effect	E Paraquat	1665 (1114 - 2042)	1657.5 (± 274.32)	1585 (1320 - 2058)	1649.4 (± 231.93)	1348 (1114 - 1599)	1364.8 (± 192.75)
No Effect	D Potassium dichromate	1320 (1191 - 1774.1)	1427.91 (± 232.53)	1388.5 (1129 - 1934)	1468.4 (± 297.23)	1511 (1280 - 1711)	1499.6 (± 205.13)
No Effect	C Trichlorethylene	1652.35 (1305.8 - 1774.1)	1631 (± 143.22)	1646.15 (1296.4 - 1853.55)	1622.33 (± 209.56)	1571.12 (1238 - 1778.5)	1498.39 (± 218.14)

A-F: Indicates Pharmaceutical Company & sample origin

Supplementary Table S18. Summary statistics for urine glucose (mmol/L) at 24 hrs, 48 hrs and 168 hrs post dose vehicle.

Target organ	Toxin	24 h post dose		48 h post dose		168 h post dose	
		Median (min - max)	Mean (± SD)	Median (min - max)	Mean (± SD)	Median (min - max)	Mean (± SD)
Liver	E 1,1-Dichloroethylene	1.94 (1.44 - 3.39)	2.13 (± 0.61)	1.75 (0.78 - 2.33)	1.78 (± 0.44)	1.83 (1.28 - 2)	1.75 (± 0.28)
Liver	E 1,2,3,4,5,6-hexachlorocyclohexane	1.44 (0.83 - 1.78)	1.37 (± 0.29)	1.33 (0.56 - 2.05)	1.38 (± 0.43)	1.93 (1.76 - 2.33)	2 (± 0.23)
Liver	B 1-Fluoropentane	1.32 (1.1 - 6.3)	1.81 (± 1.59)	1.25 (0.83 - 4.9)	1.51 (± 1.21)	1.34 (0.99 - 1.54)	1.31 (± 0.24)
Liver	B 2,4,6-Trihydroxyacetophenone (THA)	1.42 (1.09 - 2.1)	1.49 (± 0.32)	1.42 (1.09 - 1.96)	1.44 (± 0.29)	1.32 (1.15 - 1.72)	1.36 (± 0.23)
Liver	B 4-Amino-2,6-dichlorophenol (ADCP)	1.49 (1.22 - 1.71)	1.49 (± 0.16)	1.46 (1.3 - 1.76)	1.48 (± 0.15)	1.27 (1.05 - 4.59)	1.92 (± 1.5)

Target organ	Toxin	24 h post dose		48 h post dose		168 h post dose	
		Median (min - max)	Mean (± SD)	Median (min - max)	Mean (± SD)	Median (min - max)	Mean (± SD)
Liver	[C] Aflatoxin	1.41 (0.88 - 2.13)	1.43 (± 0.32)	1.54 (0.7 - 2.69)	1.58 (± 0.59)	1.79 (1.37 - 3.41)	1.97 (± 0.83)
Liver	[C] Allyl alcohol	3.68 (0.8 - 5.5)	3.42 (± 2.2)	2.42 (1.35 - 5.5)	2.74 (± 1.42)	2.58 (2.17 - 3.19)	2.63 (± 0.37)
Liver	[C] Allyl formate	1.48 (1.26 - 2.1)	1.55 (± 0.26)	1.88 (1.41 - 2.34)	1.9 (± 0.31)	1.09 (1.05 - 1.69)	1.24 (± 0.27)
Liver	[B] Azathioprine	1.49 (1.22 - 1.71)	1.49 (± 0.16)	1.46 (1.3 - 1.76)	1.48 (± 0.15)	1.27 (1.05 - 4.59)	1.92 (± 1.5)
Liver	[B] Bromobenzene	1.85 (1.47 - 2.01)	1.81 (± 0.15)	1.3 (1.07 - 2.14)	1.41 (± 0.29)	1.46 (1.33 - 1.57)	1.45 (± 0.08)
Liver	[C] Butylated hydroxytoluene	4.35 (2.16 - 5.5)	4.1 (± 1.36)	2.27 (1.14 - 5.5)	2.81 (± 1.5)	1.3 (1.14 - 1.69)	1.36 (± 0.23)
Liver	[D] Carbon tetrachloride	1.95 (0.9 - 3.9)	2.11 (± 0.87)	2.15 (1.5 - 2.7)	2.15 (± 0.35)	1.4 (1.2 - 2)	1.56 (± 0.33)
Liver	[C] Chlorpromazine	1.71 (1.35 - 2.5)	1.8 (± 0.39)	2.16 (1.5 - 3.5)	2.21 (± 0.59)	1.44 (1.07 - 1.93)	1.46 (± 0.31)
Liver	[B] Clofibrate	1.31 (0.79 - 1.68)	1.27 (± 0.26)	1.09 (0.73 - 1.4)	1.1 (± 0.2)	1.04 (0.67 - 1.27)	1.01 (± 0.24)
Liver	[B] Cyproterone acetate	1.51 (0.94 - 1.82)	1.46 (± 0.3)	1.33 (0.89 - 1.89)	1.37 (± 0.27)	1.19 (1.05 - 1.52)	1.26 (± 0.19)
Liver	[A] D-galactosamine	1.67 (1.11 - 3.05)	1.75 (± 0.55)	1.64 (0.94 - 3.61)	1.83 (± 0.71)	1.28 (0.89 - 1.44)	1.18 (± 0.25)
Liver	[B] Diethylhexylphthalate (DEHP)	1.3 (0.76 - 1.56)	1.25 (± 0.26)	1.07 (0.84 - 1.43)	1.12 (± 0.19)	1.23 (0.84 - 1.58)	1.18 (± 0.28)
Liver	[C] Dimethylformamide (DMF)	1.82 (1.26 - 2.71)	1.9 (± 0.46)	2.1 (1.72 - 5.5)	2.52 (± 1.14)	1.62 (1.37 - 1.99)	1.66 (± 0.29)
Liver	[C] Dimethylnitrosamine (DMN)	1.76 (1.03 - 2.38)	1.73 (± 0.4)	1.93 (1.35 - 4.5)	2.12 (± 0.93)	1.29 (1.11 - 1.88)	1.42 (± 0.3)
Liver	[A] Gadolinium chloride	1.53 (0.83 - 2.05)	1.44 (± 0.37)	1.39 (0.72 - 1.83)	1.38 (± 0.38)	3 (2 - 3.5)	2.71 (± 0.68)
Liver	[A, B, C, D, F] Hydrazine	1.3 (0.01 - 27)	5.29 (± 8.75)	1.73 (0 - 25)	5.13 (± 7.84)	1.22 (0 - 34)	6 (± 10.44)
Liver	[E] Hydrazine	26 (18 - 43)	26.2 (± 7.44)	29.5 (5 - 56)	29.9 (± 12.97)	20 (14 - 43)	23.8 (± 12.17)
Liver	[E] Indomethacin	1.55 (1.28 - 2)	1.59 (± 0.24)	1.17 (1 - 1.67)	1.23 (± 0.19)	1.28 (1 - 1.89)	1.38 (± 0.39)
Liver	[E] Ketoconazole	1.33 (1.05 - 2.22)	1.54 (± 0.48)	1.36 (1 - 2.22)	1.46 (± 0.42)	1.39 (1.05 - 2.11)	1.48 (± 0.44)
Liver	[C] Lead acetate	1.45 (0.96 - 2.08)	1.49 (± 0.34)	1.5 (1.08 - 5.5)	2.08 (± 1.36)	1.31 (0.78 - 1.86)	1.32 (± 0.38)
Liver	[A] Lipopolysaccharide (LPS)	1.44 (1.22 - 2)	1.47 (± 0.21)	1.05 (0.78 - 1.61)	1.14 (± 0.27)	1.22 (0.22 - 1.78)	1.1 (± 0.57)

Target organ	Toxin	24 h post dose		48 h post dose		168 h post dose	
		Median (min - max)	Mean (± SD)	Median (min - max)	Mean (± SD)	Median (min - max)	Mean (± SD)
Liver	[B] Methapyrilene	1 (0.36 - 1.66)	0.98 (± 0.5)	1.29 (0.67 - 1.5)	1.25 (± 0.24)	1.19 (1.01 - 1.27)	1.18 (± 0.1)
Liver	[E] Methylene dianiline	1.58 (1.17 - 2.39)	1.63 (± 0.38)	1.55 (1.17 - 2.16)	1.6 (± 0.33)	1.55 (1.05 - 1.67)	1.47 (± 0.24)
Liver	[C] Monocrotaline	1.71 (1.4 - 5.5)	2.49 (± 1.63)	2.2 (1.23 - 2.52)	2.06 (± 0.37)	1.38 (1.09 - 1.88)	1.48 (± 0.33)
Liver	[C] N-methylformamide (NMF)	1.86 (1.37 - 2.91)	1.95 (± 0.48)	1.97 (1.27 - 3.89)	2.19 (± 0.85)	1.4 (1.02 - 2.22)	1.53 (± 0.5)
Liver	[D] Phalloidin (chronic)	1.23 (1.01 - 1.71)	1.31 (± 0.24)	1.23 (0.91 - 1.52)	1.26 (± 0.21)	1.74 (1.44 - 2.13)	1.76 (± 0.26)
Liver	[E] Phenyl diisothiocyanate	1.44 (0.78 - 2.11)	1.44 (± 0.4)	1.64 (0.83 - 2.11)	1.53 (± 0.43)	1.55 (0.89 - 2.44)	1.61 (± 0.56)
Liver	[E] Phenyl isothiocyanate	0.64 (0.33 - 0.94)	0.68 (± 0.21)	1.72 (1.05 - 2.44)	1.72 (± 0.42)	1.72 (0.83 - 1.78)	1.51 (± 0.39)
Liver	[B] Retinyl palmitate	1.49 (1.22 - 1.71)	1.49 (± 0.16)	1.46 (1.3 - 1.76)	1.48 (± 0.15)	1.27 (1.05 - 4.59)	1.92 (± 1.5)
Liver	[B] Sodium Valproate	1.44 (1.13 - 1.7)	1.44 (± 0.19)	1.35 (0.9 - 1.75)	1.34 (± 0.29)	1.31 (0.88 - 1.46)	1.24 (± 0.24)
Liver	[C] a-Naphthylisothiocyanate (ANIT)	4.57 (1.69 - 5.5)	4.01 (± 1.66)	2.59 (1.74 - 5.5)	3.54 (± 1.71)	5.5 (2.58 - 5.5)	4.4 (± 1.52)
Kidney	[D] 2-Bromophenol	1.55 (1 - 1.9)	1.52 (± 0.3)	2.05 (1.5 - 4)	2.2 (± 0.71)	1.6 (1.2 - 2.1)	1.64 (± 0.32)
Kidney	[E] 3,5-Dichloroaniline hydrochloride	1.36 (0.83 - 1.89)	1.39 (± 0.33)	1.53 (1 - 2)	1.51 (± 0.33)	1.55 (1 - 1.61)	1.38 (± 0.28)
Kidney	[E] Atractyloside	1.36 (1 - 1.72)	1.35 (± 0.25)	1.36 (1.05 - 1.72)	1.36 (± 0.2)	1.28 (0.61 - 1.33)	1.13 (± 0.31)
Kidney	[D] Bromoethylamine hydrobromide	1.15 (0.7 - 1.6)	1.14 (± 0.26)	1.5 (0.8 - 1.7)	1.41 (± 0.32)	2 (1 - 2.2)	1.72 (± 0.5)
Kidney	[D] Cephaloridine	1.35 (0.9 - 2.4)	1.38 (± 0.48)	1.55 (0.8 - 2.4)	1.64 (± 0.53)	1.3 (1 - 2.5)	1.58 (± 0.68)
Kidney	[B] Chlorethanamine	1.11 (0.61 - 1.69)	1.16 (± 0.3)	1.02 (0.69 - 1.47)	1.06 (± 0.24)	0.91 (0.8 - 1.22)	0.96 (± 0.16)
Kidney	[A] Cisplatin	1.75 (0.9 - 2.2)	1.69 (± 0.42)	2.1 (1 - 4.1)	2.16 (± 0.87)	2 (1.5 - 2.5)	1.92 (± 0.43)
Kidney	[A] D-limonene (chronic)	2.15 (0.9 - 4.5)	2.45 (± 1.13)	1.55 (0.9 - 3)	1.74 (± 0.73)	1.7 (1 - 3.4)	1.9 (± 0.9)
Kidney	[E] Dichlorophenyl succinimide	0.6 (0.41 - 0.82)	0.63 (± 0.13)	0.62 (0.37 - 0.73)	0.59 (± 0.12)	0.85 (0.62 - 1.16)	0.92 (± 0.23)
Kidney	[D] Ethylene glycol	2 (1.2 - 2.9)	1.91 (± 0.47)	1.5 (1.2 - 2)	1.52 (± 0.25)	2.6 (1.6 - 5.3)	2.92 (± 1.45)
Kidney	[A] Folic acid	1.2 (0.8 - 2)	1.26 (± 0.35)	1.7 (1 - 2.7)	1.7 (± 0.52)	2 (1.6 - 2.6)	2.06 (± 0.48)

Target organ	Toxin	24 h post dose		48 h post dose		168 h post dose	
		Median (min - max)	Mean (± SD)	Median (min - max)	Mean (± SD)	Median (min - max)	Mean (± SD)
Kidney	[A] Gentamicin	1.45 (0.9 - 4.1)	1.76 (± 0.95)	1.75 (1.2 - 3.7)	2.04 (± 0.82)	2.6 (0.9 - 3.3)	2.2 (± 0.97)
Kidney	[B] Maleic acid	1.49 (1.22 - 1.71)	1.49 (± 0.16)	1.46 (1.3 - 1.76)	1.48 (± 0.15)	1.27 (1.05 - 4.59)	1.92 (± 1.5)
Kidney	[A] N-phenylanthranilic acid (chronic)	1.22 (0.56 - 1.89)	1.2 (± 0.39)	1.58 (0.72 - 1.89)	1.48 (± 0.38)	0.94 (0.72 - 1.44)	1.07 (± 0.29)
Kidney	[D] Para-aminophenol	1.45 (1 - 2.3)	1.44 (± 0.43)	1.8 (1 - 3.4)	1.84 (± 0.66)	2 (1.8 - 2.6)	2.04 (± 0.33)
Kidney	[A] Puromycin	1.25 (0.4 - 1.7)	1.16 (± 0.37)	1.85 (1.1 - 2.1)	1.75 (± 0.32)	1.7 (1.4 - 2)	1.66 (± 0.23)
Kidney	[B] Vancomycin hydrochloride	1.15 (0.62 - 2.19)	1.24 (± 0.57)	1.16 (0.61 - 1.32)	1.1 (± 0.23)	1.02 (0.93 - 1.17)	1.06 (± 0.1)
Liver & Kidney	[E] Acetaminophen	1.55 (0.67 - 2.39)	1.64 (± 0.49)	1.39 (0.5 - 2.33)	1.43 (± 0.46)	1.17 (0.83 - 1.89)	1.34 (± 0.49)
Liver & Kidney	[B] Aurothiomalate	1.49 (1.22 - 1.71)	1.49 (± 0.16)	1.46 (1.3 - 1.76)	1.48 (± 0.15)	1.27 (1.05 - 4.59)	1.92 (± 1.5)
Liver & Kidney	[C] Chloroform	2.88 (1.3 - 5.42)	3.42 (± 1.41)	1.81 (0.65 - 3.76)	1.98 (± 0.9)	1.64 (1.01 - 2.79)	1.7 (± 0.71)
Liver & Kidney	[D] Cyclosporin	1.85 (1.1 - 3.6)	1.93 (± 0.75)	1.5 (1 - 3.2)	1.85 (± 0.81)	1.5 (1.2 - 1.9)	1.5 (± 0.31)
Liver & Kidney	[D] Dichlorobenzene	3.7 (2.9 - 6.9)	3.91 (± 1.14)	4 (2.9 - 7.8)	4.28 (± 1.42)	2.8 (2.2 - 3.7)	2.96 (± 0.63)
Liver & Kidney	[C] Ethionine	2.19 (1.65 - 2.86)	2.22 (± 0.34)	2.14 (1.71 - 3.3)	2.23 (± 0.44)	1.83 (1.42 - 2.58)	1.96 (± 0.45)
Liver & Kidney	[B] Hexachlorobutadiene (HCBD)	1.34 (0.98 - 2.04)	1.38 (± 0.3)	1.12 (0.75 - 1.62)	1.15 (± 0.28)	0.93 (0.78 - 1.29)	1.03 (± 0.23)
Liver & Kidney	[B] Mercuric chloride	1.33 (0.63 - 1.89)	1.34 (± 0.42)	1.45 (0.83 - 1.85)	1.47 (± 0.33)	1.03 (0.65 - 1.43)	1.03 (± 0.28)
Liver & Kidney	[E] Microcystin-LR	2.22 (1.28 - 3.44)	2.29 (± 0.6)	2.44 (1.55 - 3.28)	2.41 (± 0.48)	4.33 (3.5 - 4.66)	4.19 (± 0.47)
Liver & Kidney	[E] Rotenone	1.67 (1.11 - 3.44)	1.82 (± 0.61)	1.47 (1 - 2.39)	1.54 (± 0.39)	1.5 (0.94 - 2.28)	1.57 (± 0.49)
Liver & Kidney	[E] S-(1,2-dichlorovinyl)-cysteine (DCVC)	1.53 (1.11 - 2.61)	1.53 (± 0.43)	1.47 (1.28 - 2.44)	1.57 (± 0.35)	1.17 (0.83 - 1.61)	1.21 (± 0.3)
Liver & Kidney	[D] Thioacetamide	1.15 (0.7 - 1.5)	1.1 (± 0.25)	1.4 (0.9 - 1.8)	1.39 (± 0.32)	1 (0.8 - 1.4)	1.06 (± 0.24)
Pancreas	[E] 1-Cyano-2-hydroxy-3-butene	1.47 (1.17 - 1.89)	1.52 (± 0.21)	1.67 (1.33 - 2)	1.67 (± 0.2)	1.61 (1.44 - 1.94)	1.64 (± 0.19)
Pancreas	[C] Caerulin	1.27 (0.92 - 1.59)	1.27 (± 0.2)	1.6 (1.44 - 2.02)	1.66 (± 0.19)	1.52 (1.18 - 2.59)	1.67 (± 0.54)
Pancreas	[E] L-arginine	1.39 (0.89 - 1.78)	1.35 (± 0.29)	1.42 (1 - 1.94)	1.45 (± 0.27)	1.22 (0.78 - 1.44)	1.17 (± 0.25)

Target organ	Toxin	24 h post dose		48 h post dose		168 h post dose	
		Median (min - max)	Mean (± SD)	Median (min - max)	Mean (± SD)	Median (min - max)	Mean (± SD)
Pancreas	[B] Streptozotocin	1.45 (1.27 - 2.23)	1.53 (± 0.32)	1.13 (0.97 - 1.5)	1.2 (± 0.21)	1.14 (0.98 - 1.64)	1.23 (± 0.25)
Testicular	[D] 1,3-Dinitrobenzene	2 (1.4 - 2.7)	2.11 (± 0.4)	1.7 (1.2 - 2.7)	1.8 (± 0.44)	1.3 (1.1 - 2.1)	1.5 (± 0.43)
Testicular	[C] Cadmium chloride	1.97 (1.13 - 2.49)	1.9 (± 0.44)	2.54 (1.22 - 3.21)	2.44 (± 0.61)	2.63 (1.58 - 3.1)	2.4 (± 0.63)
Testicular	[D] Cadmium chloride	1.27 (0.96 - 1.95)	1.34 (± 0.32)	1.62 (1.19 - 2.19)	1.69 (± 0.36)	1.22 (0.95 - 1.94)	1.41 (± 0.43)
Testicular	[D] Carbendazim	2.05 (0.9 - 3.5)	2.15 (± 0.67)	1.9 (0.7 - 2.6)	1.81 (± 0.49)	1.7 (1.1 - 1.8)	1.54 (± 0.29)
Testicular	[D] Di-n-pentyl-phthalate	0.74 (0.44 - 2.19)	0.86 (± 0.53)	1.94 (1.34 - 2.79)	1.95 (± 0.51)	1.08 (0.94 - 1.35)	1.14 (± 0.17)
Testicular	[D] Ethane dimethane sulfonate (EDS)	1.45 (1 - 1.8)	1.4 (± 0.27)	2.35 (1.1 - 3.1)	2.27 (± 0.61)	1.6 (1 - 2.5)	1.62 (± 0.56)
Testicular	[D] Methoxyacetic acid	1.47 (0.66 - 2.18)	1.45 (± 0.41)	1.23 (0.6 - 1.63)	1.21 (± 0.3)	1.32 (0.72 - 1.83)	1.25 (± 0.41)
Multiple organ	[B] Adriamycin	1.15 (0.8 - 1.32)	1.12 (± 0.16)	1.32 (1.05 - 1.72)	1.3 (± 0.22)	1.23 (1.03 - 1.25)	1.17 (± 0.1)
Multiple organ	[C] Amphotericin B	1.45 (1.17 - 1.97)	1.51 (± 0.25)	1.7 (1.37 - 2.38)	1.76 (± 0.31)	1.73 (0.79 - 1.78)	1.54 (± 0.42)
Multiple organ	[C] Azaserine	1.31 (0.78 - 1.77)	1.29 (± 0.3)	1.68 (0.52 - 2.38)	1.63 (± 0.49)	1.94 (1.5 - 3)	2.06 (± 0.56)
Multiple organ	[A] Dexamethasone	1.9 (1.3 - 3.1)	1.92 (± 0.51)	2.7 (1.7 - 5.4)	2.83 (± 1.05)	2.3 (1.3 - 2.9)	2.14 (± 0.64)
Multiple organ	[E] Mitomycin-C	1.19 (0.78 - 1.94)	1.27 (± 0.42)	1.19 (0.28 - 1.61)	1.12 (± 0.44)	1.17 (0.78 - 1.39)	1.14 (± 0.22)
Physiological stressor	[C] 1,1-Dichloroethylene & maleic acid	1.56 (1.14 - 1.9)	1.56 (± 0.22)	1.42 (0.61 - 2.27)	1.53 (± 0.53)	1.44 (1.04 - 1.77)	1.41 (± 0.28)
Physiological stressor	[C] 2,4-Dinitrophenol	1.7 (1.09 - 3.63)	1.84 (± 0.71)	1.5 (1.26 - 2.29)	1.64 (± 0.33)	1.1 (0.93 - 1.19)	1.07 (± 0.12)
Physiological stressor	[B] 4-Pentenoic acid	0.96 (0.63 - 1.57)	1.08 (± 0.35)	1.24 (0.91 - 1.34)	1.22 (± 0.13)	1.51 (1.45 - 1.93)	1.6 (± 0.2)
Physiological stressor	[D] Acetazolamide	1.5 (1.1 - 2.4)	1.59 (± 0.4)	1.8 (1.4 - 3.8)	2.22 (± 0.85)	3.9 (2.9 - 6.8)	4.44 (± 1.5)
Physiological stressor	[C] Acivicin	2.09 (1.01 - 4.05)	2.21 (± 0.83)	1.89 (1.05 - 2.21)	1.71 (± 0.39)	2.22 (1.44 - 2.26)	2.06 (± 0.35)
Physiological stressor	[E] Ammonium chloride	1.55 (1.11 - 1.78)	1.52 (± 0.21)	1.55 (1.17 - 1.83)	1.54 (± 0.19)	1.65 (1.52 - 2.01)	1.72 (± 0.21)
Physiological stressor	[D] Carboplatin	1.55 (1 - 1.9)	1.46 (± 0.3)	1.65 (1.2 - 2.1)	1.63 (± 0.34)	1.6 (1.4 - 2)	1.62 (± 0.23)
Physiological stressor	[A] Choline and choline/methionine deficiency (chronic)	1.19 (0.72 - 1.67)	1.19 (± 0.3)	1.19 (0.67 - 1.44)	1.13 (± 0.26)	1.22 (0.94 - 1.5)	1.19 (± 0.22)

Target organ	Toxin	24 h post dose		48 h post dose		168 h post dose	
		Median (min - max)	Mean (± SD)	Median (min - max)	Mean (± SD)	Median (min - max)	Mean (± SD)
Physiological stressor	[B] Food restriction (chronic)	1.4 (0.95 - 2.76)	1.52 (± 0.55)	1.34 (0.85 - 2.03)	1.37 (± 0.38)	1.02 (0.87 - 1.43)	1.14 (± 0.25)
Physiological stressor	[D] Furosemide	2.4 (1.1 - 3)	2.29 (± 0.59)	3.05 (1.8 - 4.5)	3.1 (± 0.9)	2.1 (1.3 - 2.4)	2.02 (± 0.42)
Physiological stressor	[B] Insulin	0.93 (0.7 - 1.27)	0.93 (± 0.15)	0.87 (0.75 - 1.47)	0.93 (± 0.21)	1.21 (1.04 - 1.65)	1.27 (± 0.24)
Physiological stressor	[E] Methotrexate	1.67 (1.22 - 2.22)	1.62 (± 0.32)	1.67 (1.22 - 1.94)	1.59 (± 0.25)	2.22 (1.78 - 2.72)	2.15 (± 0.4)
Physiological stressor	[A] Partial hepatectomy	0.11 (0.07 - 0.15)	0.11 (± 0.03)	0.1 (0.07 - 0.13)	0.1 (± 0.02)	0.09 (0.06 - 0.14)	0.1 (± 0.03)
Physiological stressor	[A] Phenobarbital (chronic)	2.35 (1.7 - 3.5)	2.37 (± 0.53)	2 (1.6 - 3.6)	2.13 (± 0.61)	2.1 (2 - 3.2)	2.32 (± 0.51)
Physiological stressor	[A] Pregnenolone 16 alpha carbonitrile (chronic)	1.8 (1.3 - 2.1)	1.77 (± 0.27)	1.95 (1.2 - 2.3)	1.84 (± 0.34)	2 (1.2 - 2.3)	1.76 (± 0.48)
Physiological stressor	[A] Probenecid	1.11 (0.33 - 1.5)	1.1 (± 0.36)	1.42 (0.89 - 1.72)	1.42 (± 0.25)	1.28 (0.94 - 1.39)	1.18 (± 0.22)
Physiological stressor	[C] Rosiglitazone	1.43 (0.97 - 2.94)	1.74 (± 0.73)	1.37 (0.83 - 5.22)	1.7 (± 1.26)	1.41 (0.39 - 1.87)	1.25 (± 0.55)
Physiological stressor	[C] Rosiglitazone (chronic)	1.58 (1.02 - 2.04)	1.51 (± 0.3)	1.58 (1.08 - 2.17)	1.69 (± 0.42)	1.55 (0.92 - 2.12)	1.48 (± 0.46)
Physiological stressor	[E] Sodium bicarbonate	1.36 (1 - 2.05)	1.44 (± 0.28)	1.58 (0.28 - 2.05)	1.43 (± 0.58)	1.39 (1.17 - 2)	1.47 (± 0.28)
Physiological stressor	[A] Unilateral nephrectomy	0.13 (0.1 - 0.17)	0.13 (± 0.03)	0.26 (0.13 - 0.63)	0.3 (± 0.15)	0.13 (0.08 - 0.17)	0.12 (± 0.03)
Physiological stressor	[B] Water deprivation (chronic)	1.6 (1.24 - 2.49)	1.64 (± 0.33)	1.4 (1.07 - 1.98)	1.42 (± 0.23)	1.35 (1.07 - 1.69)	1.37 (± 0.2)
No Effect	[E] Acetaminophen (chronic)	1.33 (1.05 - 2.66)	1.45 (± 0.49)	1.36 (0.67 - 2)	1.38 (± 0.39)	1.36 (0.83 - 1.94)	1.4 (± 0.34)
No Effect	[C] Buthionine sulphoxime	1.28 (0.8 - 2.45)	1.38 (± 0.49)	1.48 (1.05 - 2.76)	1.54 (± 0.48)	1.59 (1.57 - 1.99)	1.68 (± 0.18)
No Effect	[C] Ferrous sulphate	1.83 (1.34 - 2.78)	1.91 (± 0.52)	2.03 (1.1 - 3.39)	2.14 (± 0.7)	1.59 (1.09 - 2.67)	1.68 (± 0.61)
No Effect	[B] Ifosfamide	1.16 (0.92 - 1.68)	1.24 (± 0.28)	1.36 (0.37 - 1.6)	1.24 (± 0.39)	1.04 (0.76 - 1.47)	1.05 (± 0.26)
No Effect	[B] Lithocholic acid	1.03 (0.74 - 1.45)	1.05 (± 0.22)	0.99 (0.69 - 1.31)	1 (± 0.19)	1.1 (1 - 1.34)	1.13 (± 0.13)
No Effect	[E] Paraquat	1.36 (1.11 - 2.06)	1.43 (± 0.31)	1.36 (0.89 - 1.94)	1.37 (± 0.35)	0.94 (0.56 - 1.06)	0.87 (± 0.2)
No Effect	[D] Potassium dichromate	1.15 (0.9 - 3.6)	1.42 (± 0.83)	1.3 (0.8 - 2.4)	1.44 (± 0.5)	1.4 (1.2 - 2.3)	1.68 (± 0.57)
No Effect	[C] Trichlorethylene	1.94 (1.41 - 2.72)	1.92 (± 0.42)	2.39 (1.57 - 5.5)	2.9 (± 1.4)	1.76 (1.16 - 2.11)	1.64 (± 0.41)

Target organ	Toxin	24 h post dose		48 h post dose		168 h post dose	
		Median (min - max)	Mean (± SD)	Median (min - max)	Mean (± SD)	Median (min - max)	Mean (± SD)

A-F: Indicates Pharmaceutical Company & sample origin

Supplementary Table S19. Summary statistics for urine protein (g/L) at 24 hrs, 48 hrs and 168 hrs post dose vehicle.

Target organ	Toxin	24 h post dose		48 h post dose		168 h post dose	
		Median (min - max)	Mean (± SD)	Median (min - max)	Mean (± SD)	Median (min - max)	Mean (± SD)
Liver	[E] 1,1-Dichloroethylene	0.76 (0.43 - 0.98)	0.71 (± 0.19)	0.56 (0.33 - 0.99)	0.6 (± 0.19)	1.07 (0.7 - 1.23)	1 (± 0.23)
Liver	[E] 1,2,3,4,5,6-hexachlorocyclohexane	0.6 (0.39 - 1.05)	0.64 (± 0.19)	0.68 (0.33 - 2.61)	0.85 (± 0.64)	0.84 (0.74 - 0.98)	0.86 (± 0.12)
Liver	[B] 1-Fluoropentane	1.05 (0.54 - 1.72)	1.06 (± 0.29)	0.74 (0.6 - 1.37)	0.9 (± 0.33)	1 (0.69 - 1.48)	1.05 (± 0.29)
Liver	[B] 2,4,6-Trihydroxyacetophenone (THA)	0.76 (0.61 - 0.95)	0.76 (± 0.13)	0.69 (0.53 - 0.85)	0.7 (± 0.09)	0.87 (0.63 - 0.89)	0.81 (± 0.11)
Liver	[B] 4-Amino-2,6-dichlorophenol (ADCP)	1.1 (0.82 - 1.62)	1.18 (± 0.27)	1.16 (0.9 - 1.54)	1.23 (± 0.21)	1.06 (0.96 - 1.57)	1.19 (± 0.27)
Liver	[C] Aflatoxin	1 (1 - 1)	1 (± 0)	1 (0.3 - 1)	0.86 (± 0.3)	1 (1 - 1)	1 (± 0)
Liver	[C] Allyl alcohol	1 (0.3 - 1.33)	0.75 (± 0.4)	1 (0.3 - 1)	0.77 (± 0.36)	1 (1 - 1.24)	1.05 (± 0.11)
Liver	[C] Allyl formate	1 (1 - 1)	1 (± 0)	1 (0.3 - 1)	0.93 (± 0.22)	1 (0.3 - 1)	0.86 (± 0.31)
Liver	[B] Azathioprine	1.1 (0.82 - 1.62)	1.18 (± 0.27)	1.16 (0.9 - 1.54)	1.23 (± 0.21)	1.06 (0.96 - 1.57)	1.19 (± 0.27)
Liver	[B] Bromobenzene	1.5 (0.99 - 2.02)	1.54 (± 0.35)	1.22 (0.99 - 2.06)	1.25 (± 0.31)	1.37 (1.15 - 1.44)	1.34 (± 0.12)
Liver	[C] Butylated hydroxytoluene	0.3 (0.3 - 1)	0.45 (± 0.29)	0.3 (0.3 - 1)	0.44 (± 0.27)	0.3 (0.3 - 1)	0.58 (± 0.38)
Liver	[D] Carbon tetrachloride	0.65 (0.25 - 1.19)	0.66 (± 0.28)	0.64 (0.35 - 1.97)	0.83 (± 0.56)	1.1 (0.45 - 1.96)	1.24 (± 0.64)
Liver	[C] Chlorpromazine	0.3 (0.3 - 0.68)	0.34 (± 0.12)	0.65 (0.3 - 1)	0.65 (± 0.37)	0.3 (0.3 - 1)	0.44 (± 0.31)
Liver	[B] Clofibrate	1 (0.27 - 1.71)	1.06 (± 0.45)	1.04 (0.37 - 1.75)	1.1 (± 0.47)	1.2 (0.76 - 1.37)	1.09 (± 0.3)
Liver	[B] Cyproterone acetate	0.95 (0.54 - 3)	1.11 (± 0.73)	0.95 (0.5 - 3.51)	1.17 (± 0.87)	1.17 (0.68 - 1.48)	1.12 (± 0.3)
Liver	[A] D-galactosamine	0.56 (0.3 - 1.39)	0.66 (± 0.32)	0.64 (0.25 - 1.53)	0.71 (± 0.35)	0.84 (0.49 - 1.29)	0.89 (± 0.31)

Target organ	Toxin	24 h post dose		48 h post dose		168 h post dose	
		Median (min - max)	Mean (± SD)	Median (min - max)	Mean (± SD)	Median (min - max)	Mean (± SD)
Liver	[B] Diethylhexylphthalate (DEHP)	0.89 (0.45 - 1.26)	0.93 (± 0.22)	0.95 (0.62 - 1.13)	0.91 (± 0.2)	1.06 (0.67 - 1.58)	1.08 (± 0.34)
Liver	[C] Dimethylformamide (DMF)	0.65 (0.3 - 1)	0.65 (± 0.37)	0.3 (0.3 - 1)	0.51 (± 0.34)	1 (0.3 - 1)	0.72 (± 0.38)
Liver	[C] Dimethylnitrosamine (DMN)	0.3 (0.3 - 1)	0.44 (± 0.3)	0.3 (0 - 1)	0.48 (± 0.37)	0.3 (0.3 - 1.35)	0.51 (± 0.47)
Liver	[A] Gadolinium chloride	1.12 (0.55 - 2.06)	1.15 (± 0.47)	1.24 (0.43 - 1.61)	1.11 (± 0.44)	1.2 (0.85 - 1.82)	1.3 (± 0.38)
Liver	[A, B, C, D, F] Hydrazine	0.56 (0.05 - 1.76)	0.59 (± 0.46)	0.48 (0.05 - 2)	0.58 (± 0.5)	0.8 (0.05 - 3.1)	0.82 (± 0.67)
Liver	[E] Hydrazine	0.08 (0.04 - 0.11)	0.08 (± 0.02)	0.09 (0.03 - 0.12)	0.09 (± 0.03)	0.08 (0.07 - 0.12)	0.09 (± 0.02)
Liver	[E] Indomethacin	0.5 (0.31 - 0.67)	0.51 (± 0.1)	0.58 (0.31 - 0.7)	0.54 (± 0.11)	0.83 (0.67 - 1.06)	0.84 (± 0.14)
Liver	[E] Ketoconazole	0.46 (0.27 - 0.57)	0.44 (± 0.1)	0.54 (0.3 - 0.7)	0.52 (± 0.13)	0.9 (0.49 - 1.03)	0.85 (± 0.22)
Liver	[C] Lead acetate	0.3 (0.3 - 3)	0.64 (± 0.86)	0.38 (0.3 - 3)	0.75 (± 0.84)	0.3 (0.3 - 1)	0.44 (± 0.31)
Liver	[A] Lipopolysaccharide (LPS)	0.78 (0.45 - 1.34)	0.84 (± 0.26)	0.76 (0.31 - 1.36)	0.78 (± 0.31)	0.95 (0.12 - 1.61)	0.9 (± 0.54)
Liver	[B] Methapyrilene	0.72 (0.22 - 1.4)	0.78 (± 0.41)	0.96 (0.55 - 1.37)	0.96 (± 0.22)	1.1 (0.85 - 1.24)	1.07 (± 0.16)
Liver	[E] Methylene dianiline	0.6 (0.41 - 0.82)	0.63 (± 0.13)	0.62 (0.37 - 0.73)	0.59 (± 0.12)	0.85 (0.62 - 1.16)	0.92 (± 0.23)
Liver	[C] Monocrotaline	1 (1 - 1)	1 (± 0)	1 (0.3 - 1)	0.93 (± 0.22)	1 (1 - 1)	1 (± 0)
Liver	[C] N-methylformamide (NMF)	1 (0.3 - 3)	0.99 (± 0.78)	0.65 (0.3 - 1.2)	0.67 (± 0.39)	1 (1 - 1)	1 (± 0)
Liver	[D] Phalloidin (chronic)	0.59 (0.41 - 0.98)	0.6 (± 0.16)	0.67 (0.53 - 1.19)	0.71 (± 0.21)	0.95 (0.85 - 1.08)	0.94 (± 0.09)
Liver	[E] Phenyl diisothiocyanate	0.34 (0.18 - 0.69)	0.37 (± 0.17)	0.41 (0.22 - 0.86)	0.44 (± 0.2)	0.99 (0.58 - 1.2)	0.92 (± 0.23)
Liver	[E] Phenyl isothiocyanate	0.25 (0.17 - 0.39)	0.26 (± 0.08)	0.52 (0.28 - 1.11)	0.56 (± 0.24)	1.04 (0.53 - 1.07)	0.93 (± 0.23)
Liver	[B] Retinyl palmitate	1.1 (0.82 - 1.62)	1.18 (± 0.27)	1.16 (0.9 - 1.54)	1.23 (± 0.21)	1.06 (0.96 - 1.57)	1.19 (± 0.27)
Liver	[B] Sodium Valproate	1.17 (1 - 1.51)	1.23 (± 0.18)	1.05 (0.57 - 1.62)	1.08 (± 0.31)	1.14 (0.61 - 1.28)	1.05 (± 0.26)
Liver	[C] a-Naphthylisothiocyanate (ANIT)	1 (0.3 - 1)	0.86 (± 0.3)	1 (0.3 - 1)	0.93 (± 0.22)	1 (1 - 1)	1 (± 0)
Kidney	[D] 2-Bromophenol	0.26 (0.1 - 0.87)	0.32 (± 0.22)	0.4 (0.16 - 1.88)	0.52 (± 0.5)	0.88 (0.28 - 4.3)	1.33 (± 1.69)

Target organ	Toxin	24 h post dose		48 h post dose		168 h post dose	
		Median (min - max)	Mean (± SD)	Median (min - max)	Mean (± SD)	Median (min - max)	Mean (± SD)
Kidney	[E] 3,5-Dichloroaniline hydrochloride	0.32 (0.23 - 0.47)	0.34 (± 0.08)	0.42 (0.24 - 0.57)	0.39 (± 0.1)	0.86 (0.57 - 0.95)	0.81 (± 0.15)
Kidney	[E] Atractyloside	0.34 (0.22 - 0.62)	0.37 (± 0.11)	0.44 (0.27 - 0.75)	0.45 (± 0.13)	0.88 (0.72 - 0.91)	0.84 (± 0.09)
Kidney	[D] Bromoethylamine hydrobromide	0.2 (0.12 - 0.43)	0.22 (± 0.09)	0.22 (0.16 - 0.68)	0.3 (± 0.17)	0.64 (0.51 - 0.75)	0.64 (± 0.09)
Kidney	[D] Cephaloridine	0.21 (0.13 - 0.53)	0.26 (± 0.12)	0.26 (0.18 - 0.73)	0.31 (± 0.16)	0.56 (0.49 - 2.16)	0.87 (± 0.72)
Kidney	[B] Chlorethanamine	0.66 (0.3 - 1.47)	0.75 (± 0.36)	0.66 (0.44 - 1.23)	0.74 (± 0.28)	0.73 (0.68 - 1.45)	0.89 (± 0.32)
Kidney	[A] Cisplatin	0.6 (0.3 - 0.94)	0.63 (± 0.21)	0.65 (0.37 - 1.26)	0.71 (± 0.25)	1.35 (0.78 - 1.75)	1.29 (± 0.41)
Kidney	[A] D-limonene (chronic)	0.7 (0.38 - 1)	0.69 (± 0.2)	0.57 (0.35 - 0.88)	0.6 (± 0.16)	0.64 (0.53 - 0.76)	0.64 (± 0.12)
Kidney	[E] Dichlorophenyl succinimide	0.6 (0.41 - 0.82)	0.63 (± 0.13)	0.62 (0.37 - 0.73)	0.59 (± 0.12)	0.85 (0.62 - 1.16)	0.92 (± 0.23)
Kidney	[D] Ethylene glycol	0.36 (0.22 - 0.51)	0.36 (± 0.09)	0.34 (0.27 - 0.64)	0.39 (± 0.13)	0.85 (0.7 - 1.72)	0.99 (± 0.41)
Kidney	[A] Folic acid	0.31 (0.18 - 0.57)	0.31 (± 0.11)	0.42 (0.22 - 0.72)	0.42 (± 0.14)	0.64 (0.62 - 2)	0.97 (± 0.59)
Kidney	[A] Gentamicin	0.29 (0.13 - 0.64)	0.32 (± 0.16)	0.4 (0.19 - 0.77)	0.42 (± 0.2)	0.7 (0.18 - 1.22)	0.65 (± 0.39)
Kidney	[B] Maleic acid	1.1 (0.82 - 1.62)	1.18 (± 0.27)	1.16 (0.9 - 1.54)	1.23 (± 0.21)	1.06 (0.96 - 1.57)	1.19 (± 0.27)
Kidney	[A] N-phenylanthranilic acid (chronic)	0.36 (0.12 - 0.54)	0.36 (± 0.14)	0.38 (0.15 - 0.5)	0.35 (± 0.12)	0.29 (0.23 - 1.04)	0.5 (± 0.34)
Kidney	[D] Para-aminophenol	0.31 (0.15 - 0.66)	0.34 (± 0.16)	0.4 (0.2 - 0.82)	0.44 (± 0.19)	0.81 (0.41 - 1.8)	0.92 (± 0.53)
Kidney	[A] Puromycin	0.28 (0.15 - 0.47)	0.3 (± 0.12)	0.45 (0.23 - 0.63)	0.43 (± 0.13)	0.65 (0.54 - 1.4)	0.78 (± 0.35)
Kidney	[B] Vancomycin hydrochloride	0.78 (0.51 - 2.12)	1.02 (± 0.6)	0.76 (0.24 - 1.43)	0.8 (± 0.42)	0.93 (0.81 - 1.08)	0.94 (± 0.11)
Liver & Kidney	[E] Acetaminophen	0.53 (0.27 - 0.62)	0.51 (± 0.1)	0.55 (0.37 - 0.81)	0.57 (± 0.13)	0.85 (0.61 - 1.01)	0.84 (± 0.16)
Liver & Kidney	[B] Aurothiomalate	1.1 (0.82 - 1.62)	1.18 (± 0.27)	1.16 (0.9 - 1.54)	1.23 (± 0.21)	1.06 (0.96 - 1.57)	1.19 (± 0.27)
Liver & Kidney	[C] Chloroform	0.3 (0.3 - 1)	0.37 (± 0.22)	0.3 (0.3 - 1)	0.51 (± 0.34)	1 (0.3 - 3.2)	1.16 (± 1.19)
Liver & Kidney	[D] Cyclosporin	0.29 (0.12 - 0.72)	0.3 (± 0.16)	0.31 (0.17 - 0.92)	0.34 (± 0.21)	0.66 (0.18 - 0.81)	0.62 (± 0.26)
Liver & Kidney	[D] Dichlorobenzene	0.24 (0.17 - 0.39)	0.26 (± 0.07)	0.29 (0.2 - 0.48)	0.3 (± 0.09)	0.64 (0.52 - 1.78)	0.88 (± 0.52)

Target organ	Toxin	24 h post dose		48 h post dose		168 h post dose	
		Median (min - max)	Mean (± SD)	Median (min - max)	Mean (± SD)	Median (min - max)	Mean (± SD)
Liver & Kidney	[C] Ethionine	0.3 (0.3 - 1)	0.55 (± 0.33)	0.3 (0.3 - 1)	0.58 (± 0.36)	1 (0.3 - 1)	0.86 (± 0.31)
Liver & Kidney	[B] Hexachlorobutadiene (HCBD)	1.25 (0.48 - 2.16)	1.22 (± 0.47)	0.74 (0.34 - 1.39)	0.86 (± 0.4)	0.88 (0.44 - 1.5)	0.93 (± 0.49)
Liver & Kidney	[B] Mercuric chloride	1.17 (0.5 - 1.95)	1.17 (± 0.53)	1.45 (0.76 - 2.02)	1.41 (± 0.49)	0.94 (0.64 - 1.37)	1 (± 0.27)
Liver & Kidney	[E] Microcystin-LR	0.4 (0.23 - 0.62)	0.41 (± 0.11)	0.44 (0.28 - 0.59)	0.43 (± 0.09)	0.78 (0.63 - 0.84)	0.75 (± 0.08)
Liver & Kidney	[E] Rotenone	0.58 (0.37 - 1.03)	0.62 (± 0.2)	0.52 (0.33 - 0.88)	0.56 (± 0.16)	0.85 (0.72 - 1.2)	0.88 (± 0.19)
Liver & Kidney	[E] S-(1,2-dichlorovinyl)-cysteine (DCVC)	0.43 (0.35 - 0.62)	0.46 (± 0.11)	0.5 (0.42 - 0.75)	0.54 (± 0.12)	0.99 (0.83 - 1.42)	1.06 (± 0.23)
Liver & Kidney	[D] Thioacetamide	0.26 (0.17 - 0.41)	0.27 (± 0.08)	0.32 (0.26 - 0.55)	0.35 (± 0.09)	0.54 (0.43 - 0.85)	0.59 (± 0.16)
Pancreas	[E] 1-Cyano-2-hydroxy-3-butene	0.46 (0.22 - 0.72)	0.47 (± 0.16)	0.52 (0.3 - 0.87)	0.52 (± 0.17)	0.74 (0.45 - 0.86)	0.7 (± 0.17)
Pancreas	[C] Caerulin	1 (0.3 - 1)	0.79 (± 0.34)	1 (1 - 1)	1 (± 0)	1 (0.3 - 1)	0.86 (± 0.31)
Pancreas	[E] L-arginine	0.63 (0.42 - 1.04)	0.64 (± 0.2)	0.64 (0.47 - 1.07)	0.71 (± 0.21)	0.85 (0.67 - 1.29)	0.9 (± 0.23)
Pancreas	[B] Streptozotocin	1.34 (0.84 - 2.4)	1.42 (± 0.42)	1.21 (0.89 - 1.84)	1.23 (± 0.3)	1.55 (1.21 - 1.59)	1.49 (± 0.16)
Testicular	[D] 1,3-Dinitrobenzene	0.62 (0.48 - 0.95)	0.63 (± 0.16)	0.6 (0.32 - 0.92)	0.63 (± 0.17)	0.69 (0.48 - 0.87)	0.7 (± 0.16)
Testicular	[C] Cadmium chloride	0.3 (0.3 - 1)	0.51 (± 0.34)	1 (0.3 - 1)	0.72 (± 0.36)	1 (0.3 - 1)	0.72 (± 0.38)
Testicular	[D] Cadmium chloride	0.54 (0.24 - 0.85)	0.52 (± 0.2)	0.69 (0.33 - 1.15)	0.72 (± 0.26)	0.93 (0.41 - 1.07)	0.77 (± 0.31)
Testicular	[D] Carbendazim	1.4 (0.66 - 1.7)	1.32 (± 0.35)	0.92 (0.6 - 1.78)	1.06 (± 0.37)	1 (0.67 - 1.9)	1.15 (± 0.49)
Testicular	[D] Di-n-pentyl-phthalate	0.32 (0.18 - 0.74)	0.37 (± 0.2)	0.67 (0.44 - 1.5)	0.82 (± 0.35)	0.65 (0.42 - 1.02)	0.7 (± 0.22)
Testicular	[D] Ethane dimethane sulfonate (EDS)	0.76 (0.42 - 1.07)	0.77 (± 0.2)	1.34 (0.67 - 1.87)	1.32 (± 0.36)	1.37 (0.94 - 1.76)	1.35 (± 0.35)
Testicular	[D] Methoxyacetic acid	0.84 (0.38 - 1.15)	0.8 (± 0.24)	0.76 (0.39 - 1.15)	0.76 (± 0.24)	0.89 (0.5 - 1.19)	0.85 (± 0.26)
Multiple organ	[B] Adriamycin	1.2 (0.48 - 1.69)	1.21 (± 0.37)	1.27 (0.67 - 1.74)	1.23 (± 0.33)	1.17 (0.73 - 1.44)	1.18 (± 0.29)
Multiple organ	[C] Amphotericin B	1 (0.3 - 1)	0.93 (± 0.22)	1 (1 - 3)	1.2 (± 0.63)	1 (0.3 - 1)	0.86 (± 0.31)
Multiple organ	[C] Azaserine	0.3 (0.3 - 1)	0.58 (± 0.36)	1 (0.3 - 1)	0.86 (± 0.3)	1 (0.3 - 1)	0.86 (± 0.31)

Target organ	Toxin	24 h post dose		48 h post dose		168 h post dose	
		Median (min - max)	Mean (± SD)	Median (min - max)	Mean (± SD)	Median (min - max)	Mean (± SD)
Multiple organ	[A] Dexamethasone	0.82 (0.37 - 1.85)	0.83 (± 0.42)	1.17 (0.42 - 2.55)	1.26 (± 0.64)	1.32 (0.81 - 2.4)	1.35 (± 0.65)
Multiple organ	[E] Mitomycin-C	0.5 (0.27 - 0.87)	0.56 (± 0.2)	0.62 (0.28 - 0.9)	0.58 (± 0.21)	1.1 (0.85 - 2.09)	1.27 (± 0.52)
Physiological stressor	[C] 1,1-Dichloroethylene & maleic acid	1 (0.3 - 1)	0.86 (± 0.3)	0.65 (0.3 - 1)	0.65 (± 0.37)	1 (1 - 1)	1 (± 0)
Physiological stressor	[C] 2,4-Dinitrophenol	0.3 (0.3 - 1)	0.46 (± 0.29)	0.3 (0.3 - 1)	0.44 (± 0.3)	1 (0.3 - 1)	0.86 (± 0.31)
Physiological stressor	[B] 4-Pentenoic acid	0.79 (0.37 - 1.29)	0.79 (± 0.31)	1.01 (0.62 - 1.28)	0.98 (± 0.19)	1.36 (1.11 - 1.67)	1.38 (± 0.22)
Physiological stressor	[D] Acetazolamide	0.23 (0.15 - 0.39)	0.26 (± 0.07)	0.34 (0.22 - 0.54)	0.34 (± 0.09)	0.82 (0.52 - 1.64)	0.92 (± 0.44)
Physiological stressor	[C] Acivicin	1 (0.3 - 1)	0.86 (± 0.3)	1 (0.3 - 1)	0.93 (± 0.22)	1 (1 - 1)	1 (± 0)
Physiological stressor	[E] Ammonium chloride	0.56 (0.42 - 0.9)	0.59 (± 0.15)	0.62 (0.45 - 0.9)	0.66 (± 0.15)	0.96 (0.74 - 1.07)	0.92 (± 0.12)
Physiological stressor	[D] Carboplatin	0.27 (0.14 - 0.51)	0.31 (± 0.13)	0.39 (0.18 - 0.61)	0.39 (± 0.15)	0.97 (0.7 - 1.48)	1.11 (± 0.34)
Physiological stressor	[A] Choline and choline/methionine deficiency (chronic)	0.36 (0.23 - 0.63)	0.39 (± 0.13)	0.41 (0.24 - 0.6)	0.41 (± 0.15)	0.97 (0.67 - 1.22)	0.92 (± 0.22)
Physiological stressor	[B] Food restriction (chronic)	1.16 (0.38 - 1.98)	1.18 (± 0.48)	1.03 (0.58 - 1.85)	1.17 (± 0.46)	1.29 (0.63 - 1.88)	1.27 (± 0.54)
Physiological stressor	[D] Furosemide	0.42 (0.21 - 0.67)	0.44 (± 0.18)	0.51 (0.24 - 0.78)	0.51 (± 0.19)	0.79 (0.35 - 1.68)	0.91 (± 0.59)
Physiological stressor	[B] Insulin	0.61 (0.34 - 0.84)	0.6 (± 0.16)	0.5 (0.39 - 1.1)	0.6 (± 0.23)	0.9 (0.62 - 1.46)	0.97 (± 0.31)
Physiological stressor	[E] Methotrexate	0.7 (0.36 - 0.84)	0.65 (± 0.16)	0.81 (0.48 - 0.93)	0.77 (± 0.15)	1.08 (0.99 - 1.36)	1.15 (± 0.17)
Physiological stressor	[A] Partial hepatectomy	0.41 (0.16 - 0.55)	0.39 (± 0.14)	0.32 (0.15 - 0.72)	0.39 (± 0.19)	1.01 (0.31 - 1.18)	0.8 (± 0.4)
Physiological stressor	[A] Phenobarbital (chronic)	0.62 (0.31 - 0.83)	0.63 (± 0.18)	0.76 (0.23 - 0.95)	0.7 (± 0.2)	1.46 (0.37 - 1.84)	1.29 (± 0.57)
Physiological stressor	[A] Pregnenolone 16 alpha carbonitrile (chronic)	0.54 (0.28 - 0.94)	0.59 (± 0.21)	0.48 (0.27 - 0.85)	0.55 (± 0.22)	0.64 (0.32 - 0.88)	0.66 (± 0.23)
Physiological stressor	[A] Probenecid	0.82 (0.09 - 1.48)	0.81 (± 0.41)	0.94 (0.49 - 1.92)	1.05 (± 0.43)	0.98 (0.66 - 1.9)	1.14 (± 0.47)
Physiological stressor	[C] Rosiglitazone	0.9 (0.3 - 1.17)	0.72 (± 0.37)	1 (0.3 - 1)	0.72 (± 0.36)	0.66 (0.3 - 1)	0.65 (± 0.35)
Physiological stressor	[C] Rosiglitazone (chronic)	1 (1 - 1)	1 (± 0)	1 (1 - 1)	1 (± 0)	1 (1 - 3)	1.8 (± 1.1)
Physiological stressor	[E] Sodium bicarbonate	0.23 (0.16 - 0.42)	0.26 (± 0.07)	0.28 (0.1 - 0.44)	0.27 (± 0.1)	0.66 (0.35 - 0.89)	0.63 (± 0.18)

Target organ	Toxin	24 h post dose		48 h post dose		168 h post dose	
		Median (min - max)	Mean (± SD)	Median (min - max)	Mean (± SD)	Median (min - max)	Mean (± SD)
Physiological stressor	[A] Unilateral nephrectomy	0.45 (0.28 - 0.82)	0.52 (± 0.21)	0.48 (0.24 - 1.14)	0.54 (± 0.27)	0.85 (0.45 - 1.17)	0.82 (± 0.33)
Physiological stressor	[B] Water deprivation (chronic)	1.46 (0.88 - 2.13)	1.5 (± 0.39)	1.26 (0.74 - 1.58)	1.26 (± 0.25)	1.15 (0.94 - 1.73)	1.21 (± 0.28)
No Effect	[E] Acetaminophen (chronic)	0.5 (0.37 - 0.83)	0.55 (± 0.14)	0.62 (0.45 - 0.92)	0.63 (± 0.15)	1 (0.78 - 1.17)	1 (± 0.15)
No Effect	[C] Buthionine sulphoxime	0.65 (0.3 - 3)	0.85 (± 0.83)	1 (0.3 - 3)	1.06 (± 0.74)	1 (0.3 - 1)	0.86 (± 0.31)
No Effect	[C] Ferrous sulphate	0.65 (0.3 - 1)	0.65 (± 0.34)	1 (0.3 - 1)	0.78 (± 0.33)	1 (1 - 1)	1 (± 0)
No Effect	[B] Ifosfamide	0.81 (0.38 - 1.2)	0.83 (± 0.27)	0.86 (0.1 - 1.43)	0.84 (± 0.4)	0.81 (0.34 - 0.85)	0.72 (± 0.22)
No Effect	[B] Lithocholic acid	0.83 (0.42 - 1.24)	0.83 (± 0.26)	0.68 (0.28 - 1.16)	0.71 (± 0.23)	0.9 (0.85 - 1.19)	1 (± 0.17)
No Effect	[E] Paraquat	0.52 (0.31 - 0.68)	0.52 (± 0.12)	0.62 (0.35 - 0.78)	0.59 (± 0.14)	0.8 (0.53 - 1.11)	0.84 (± 0.24)
No Effect	[D] Potassium dichromate	0.23 (0.17 - 0.99)	0.33 (± 0.25)	0.34 (0.17 - 0.58)	0.35 (± 0.15)	0.56 (0.32 - 0.71)	0.53 (± 0.15)
No Effect	[C] Trichlorethylene	0.65 (0.3 - 1)	0.65 (± 0.37)	1 (0.3 - 1)	0.93 (± 0.22)	1 (0.3 - 1)	0.86 (± 0.31)

A-F: Indicates Pharmaceutical Company & sample origin

Supplementary Table S20. Summary statistics for serum calcium (mmol/L) at 24 hrs, 48 hrs and 168 hrs post high dose.

Target organ	Toxin	24 h post dose		48 h post dose		168 h post dose	
		Median (min - max)	Mean (± SD)	Median (min - max)	Mean (± SD)	Median (min - max)	Mean (± SD)
Liver	[E] 1,1-Dichloroethylene	2.78 (2.67 - 2.84)	2.76 (± 0.07)	2.76 (2.74 - 2.81)	2.76 (± 0.03)	2.74 (2.64 - 2.76)	2.73 (± 0.05)
Liver	[E] 1,2,3,4,5,6-hexachlorocyclohexane	2.75 (2.67 - 2.81)	2.74 (± 0.06)	2.79 (2.74 - 2.84)	2.79 (± 0.04)	2.76 (2.57 - 2.86)	2.75 (± 0.11)
Liver	[B] 1-Fluoropentane	2.67 (2.55 - 2.83)	2.66 (± 0.08)	2.6 (2.5 - 2.7)	2.61 (± 0.07)	2.75 (2.68 - 2.9)	2.77 (± 0.09)
Liver	[B] 2,4,6-Trihydroxyacetophenone (THA)	2.64 (2.54 - 2.76)	2.64 (± 0.07)	2.64 (2.57 - 2.74)	2.64 (± 0.08)	2.62 (2.59 - 2.69)	2.64 (± 0.04)
Liver	[B] 4-Amino-2,6-dichlorophenol (ADCP)	2.67 (2.61 - 2.76)	2.68 (± 0.05)	2.73 (2.61 - 2.78)	2.71 (± 0.06)	2.71 (2.66 - 2.76)	2.7 (± 0.04)
Liver	[C] Aflatoxin	2.76 (2.51 - 2.9)	2.72 (± 0.11)	2.76 (2.68 - 2.77)	2.75 (± 0.04)	2.65 (2.54 - 2.92)	2.68 (± 0.14)
Liver	[C] Allyl alcohol	2.71 (2.61 - 3.25)	2.76 (± 0.2)	2.63 (2.41 - 2.78)	2.61 (± 0.15)	2.83 (2.55 - 3.01)	2.79 (± 0.17)
Liver	[C] Allyl formate	2.74 (2.59 - 2.89)	2.75 (± 0.09)	2.72 (2.62 - 2.81)	2.72 (± 0.07)	2.79 (2.7 - 2.89)	2.79 (± 0.07)
Liver	[B] Azathioprine	2.71 (2.63 - 2.81)	2.7 (± 0.05)	2.51 (2.46 - 2.61)	2.53 (± 0.06)	2.68 (2.63 - 2.78)	2.7 (± 0.06)
Liver	[B] Bromobenzene	2.72 (2.47 - 2.81)	2.7 (± 0.11)	2.52 (2.34 - 2.57)	2.46 (± 0.1)	2.89 (2.84 - 2.94)	2.88 (± 0.04)
Liver	[C] Butylated hydroxytoluene	2.71 (2.54 - 2.93)	2.73 (± 0.11)	2.74 (2.7 - 2.81)	2.75 (± 0.04)	3 (2.73 - 3.12)	2.97 (± 0.15)
Liver	[D] Carbon tetrachloride	2.48 (2.24 - 2.56)	2.43 (± 0.13)	3.14 (2.99 - 3.2)	3.11 (± 0.09)	2.74 (2.72 - 2.93)	2.78 (± 0.08)
Liver	[C] Chlorpromazine	2.56 (1.1 - 6.29)	2.68 (± 1.46)	2.9 (2.68 - 2.94)	2.83 (± 0.12)	2.75 (2.63 - 2.86)	2.76 (± 0.08)
Liver	[B] Clofibrate	2.75 (2.72 - 2.84)	2.76 (± 0.04)	2.72 (2.64 - 2.76)	2.7 (± 0.05)	2.74 (2.69 - 2.86)	2.75 (± 0.07)
Liver	[B] Cyproterone acetate	2.75 (2.64 - 2.86)	2.76 (± 0.07)	2.47 (2.32 - 2.59)	2.46 (± 0.1)	2.79 (2.67 - 2.89)	2.79 (± 0.08)
Liver	[A] D-galactosamine	2.55 (2.12 - 2.65)	2.44 (± 0.21)	2.69 (2.57 - 2.82)	2.69 (± 0.1)	2.79 (2.49 - 2.89)	2.73 (± 0.18)
Liver	[B] Diethylhexylphthalate (DEHP)			2.67 (2.64 - 2.79)	2.7 (± 0.06)	2.84 (2.74 - 2.86)	2.81 (± 0.05)
Liver	[C] Dimethylformamide (DMF)	2.74 (2.61 - 2.86)	2.75 (± 0.08)	2.84 (2.73 - 2.92)	2.82 (± 0.07)	2.76 (2.7 - 2.81)	2.76 (± 0.05)
Liver	[C] Dimethylnitrosamine (DMN)	2.65 (2.5 - 2.8)	2.65 (± 0.11)	2.5 (2.38 - 2.64)	2.51 (± 0.1)	2.73 (2.63 - 3)	2.8 (± 0.14)
Liver	[A] Gadolinium chloride	2.66 (2.52 - 2.82)	2.66 (± 0.11)	2.99 (2.94 - 3.09)	3 (± 0.07)	3.06 (3.02 - 3.19)	3.08 (± 0.07)

Target organ	Toxin	24 h post dose		48 h post dose		168 h post dose	
		Median (min - max)	Mean (± SD)	Median (min - max)	Mean (± SD)	Median (min - max)	Mean (± SD)
Liver	[A, B, C, D, F] Hydrazine	2.52 (1.9 - 2.91)	2.55 (± 0.22)	2.52 (2.27 - 2.69)	2.51 (± 0.12)	2.81 (2.35 - 3.1)	2.8 (± 0.24)
Liver	[E] Hydrazine	2.29 (2.19 - 2.59)	2.34 (± 0.13)	1.79 (1.69 - 1.99)	1.81 (± 0.15)	2.39 (0.89 - 3.49)	2.21 (± 1)
Liver	[E] Indomethacin	2.24 (1.69 - 2.39)	2.17 (± 0.21)	2.47 (2.39 - 2.81)	2.52 (± 0.18)	2.74 (2.42 - 3.39)	2.79 (± 0.37)
Liver	[E] Ketoconazole			2.78 (2.75 - 2.85)	2.78 (± 0.04)	2.75 (2.6 - 2.83)	2.72 (± 0.09)
Liver	[C] Lead acetate	2.86 (2.3 - 3.41)	2.82 (± 0.32)	2.8 (2.65 - 2.81)	2.76 (± 0.07)	2.71 (2.64 - 2.98)	2.77 (± 0.14)
Liver	[A] Lipopolysaccharide (LPS)	2.62 (2.48 - 2.75)	2.63 (± 0.1)	3.15 (2.85 - 3.33)	3.13 (± 0.17)	3.3 (3.13 - 3.38)	3.29 (± 0.1)
Liver	[B] Methapyrilene	2.67 (2.59 - 2.76)	2.66 (± 0.05)	2.69 (2.59 - 2.79)	2.68 (± 0.09)	2.72 (2.69 - 2.76)	2.73 (± 0.03)
Liver	[E] Methylene dianiline	2.58 (2.53 - 2.75)	2.62 (± 0.09)	2.88 (2.75 - 2.98)	2.86 (± 0.09)	2.83 (2.8 - 2.85)	2.82 (± 0.02)
Liver	[C] Monocrotaline	2.8 (2.6 - 3.08)	2.84 (± 0.15)	2.7 (2.64 - 2.73)	2.69 (± 0.04)	2.91 (2.84 - 3.02)	2.91 (± 0.07)
Liver	[C] N-methylformamide (NMF)	2.68 (2.59 - 2.85)	2.7 (± 0.07)	2.59 (2.56 - 2.75)	2.62 (± 0.08)	2.73 (2.66 - 2.8)	2.73 (± 0.06)
Liver	[D] Phalloidin (chronic)			2.75 (2.7 - 2.81)	2.75 (± 0.04)	2.96 (2.89 - 3.11)	2.97 (± 0.08)
Liver	[E] Phenyl diisothiocyanate	2.48 (2.37 - 2.62)	2.48 (± 0.09)	2.62 (2.49 - 2.76)	2.63 (± 0.11)	2.81 (2.52 - 2.82)	2.7 (± 0.15)
Liver	[E] Phenyl isothiocyanate	2.56 (2.51 - 2.72)	2.59 (± 0.08)	2.72 (2.64 - 2.84)	2.72 (± 0.08)	2.74 (2.69 - 2.94)	2.77 (± 0.1)
Liver	[B] Retinyl palmitate	2.87 (2.68 - 3.01)	2.86 (± 0.08)	2.66 (2.56 - 2.73)	2.64 (± 0.07)	2.76 (2.68 - 2.93)	2.78 (± 0.09)
Liver	[B] Sodium Valproate			1.75 (1.52 - 2.67)	1.95 (± 0.47)	2.79 (2.74 - 2.82)	2.78 (± 0.04)
Liver	[C] a-Naphthylisothiocyanate (ANIT)	2.69 (2.38 - 2.9)	2.67 (± 0.16)	2.83 (2.79 - 3.19)	2.95 (± 0.2)	2.85 (2.74 - 2.97)	2.84 (± 0.09)
Kidney	[D] 2-Bromophenol	2.48 (2.31 - 2.64)	2.49 (± 0.09)	3.31 (2.96 - 4.2)	3.4 (± 0.48)	3.09 (3.03 - 3.18)	3.11 (± 0.07)
Kidney	[E] 3,5-Dichloroaniline hydrochloride	2.47 (2.24 - 2.62)	2.45 (± 0.11)	2.72 (2.62 - 2.74)	2.7 (± 0.05)	2.69 (2.62 - 2.74)	2.69 (± 0.05)
Kidney	[E] Atractyloside	2.84 (2.32 - 3.56)	2.87 (± 0.36)	3.24 (2.96 - 3.44)	3.23 (± 0.2)	2.79 (2.74 - 2.91)	2.82 (± 0.07)
Kidney	[D] Bromoethylamine hydrobromide	2.54 (2.45 - 2.63)	2.54 (± 0.06)	2.83 (2.36 - 3)	2.77 (± 0.24)	2.94 (2.89 - 3.04)	2.95 (± 0.06)

Target organ	Toxin	24 h post dose		48 h post dose		168 h post dose	
		Median (min - max)	Mean (± SD)	Median (min - max)	Mean (± SD)	Median (min - max)	Mean (± SD)
Kidney	[D] Cephaloridine	2.56 (2.49 - 2.85)	2.59 (± 0.12)	3.31 (3.23 - 3.55)	3.37 (± 0.14)	3.12 (3 - 3.24)	3.13 (± 0.1)
Kidney	[B] Chlorethanamine	2.76 (2.67 - 2.87)	2.77 (± 0.06)	2.59 (2.54 - 2.67)	2.6 (± 0.05)	2.82 (2.77 - 2.84)	2.81 (± 0.03)
Kidney	[A] Cisplatin	2.56 (2.48 - 2.76)	2.57 (± 0.08)	3.02 (2.81 - 3.22)	3.03 (± 0.16)	2.89 (2.71 - 3.27)	2.97 (± 0.23)
Kidney	[A] D-limonene (chronic)						
Kidney	[E] Dichlorophenyl succinimide	2.47 (2.3 - 2.58)	2.47 (± 0.1)	2.8 (2.75 - 2.83)	2.8 (± 0.03)	2.85 (2.8 - 2.9)	2.86 (± 0.04)
Kidney	[D] Ethylene glycol	2.54 (2.32 - 2.66)	2.52 (± 0.11)	3.01 (2.89 - 3.28)	3.02 (± 0.16)	3.05 (2.79 - 3.32)	3.08 (± 0.22)
Kidney	[A] Folic acid	2.42 (2.21 - 3.19)	2.5 (± 0.28)	2.82 (2.09 - 3.17)	2.65 (± 0.45)	2.84 (2.82 - 2.94)	2.86 (± 0.05)
Kidney	[A] Gentamicin	2.6 (2.5 - 2.84)	2.64 (± 0.12)	3.04 (2.96 - 3.64)	3.18 (± 0.28)	3 (2.85 - 3.28)	3.03 (± 0.18)
Kidney	[B] Maleic acid	2.69 (2.58 - 2.76)	2.69 (± 0.06)	2.78 (2.68 - 2.83)	2.76 (± 0.06)	2.71 (2.63 - 2.71)	2.69 (± 0.03)
Kidney	[A] N-phenylanthranilic acid (chronic)	2.59 (2.48 - 2.93)	2.62 (± 0.13)	2.78 (2.48 - 3.27)	2.81 (± 0.33)	2.85 (2.68 - 3.2)	2.95 (± 0.23)
Kidney	[D] Para-aminophenol	2.87 (2.64 - 3.4)	2.94 (± 0.26)	3.65 (2.82 - 4.56)	3.64 (± 0.63)	3.03 (2.73 - 3.32)	3.06 (± 0.23)
Kidney	[A] Puromycin			2.87 (2.6 - 2.93)	2.78 (± 0.15)	2.87 (2.63 - 3.21)	2.88 (± 0.25)
Kidney	[B] Vancomycin hydrochloride			2.79 (2.72 - 2.94)	2.81 (± 0.08)	2.84 (2.69 - 2.84)	2.8 (± 0.07)
Liver & Kidney	[E] Acetaminophen	2.52 (2.39 - 2.64)	2.52 (± 0.08)	2.72 (2.69 - 2.81)	2.74 (± 0.05)	2.89 (2.81 - 3.01)	2.9 (± 0.07)
Liver & Kidney	[B] Aurothiomalate	2.82 (1.96 - 3.31)	2.78 (± 0.35)	2.86 (2.61 - 3.01)	2.86 (± 0.16)	2.56 (2.51 - 2.68)	2.59 (± 0.08)
Liver & Kidney	[C] Chloroform	2.74 (2.58 - 3.14)	2.78 (± 0.16)	2.64 (2.57 - 2.76)	2.65 (± 0.08)	2.71 (2.69 - 2.83)	2.74 (± 0.06)
Liver & Kidney	[D] Cyclosporin	2.47 (2.35 - 2.58)	2.46 (± 0.07)	2.93 (2.86 - 3.19)	2.96 (± 0.12)	3.12 (2.94 - 3.35)	3.12 (± 0.16)
Liver & Kidney	[D] Dichlorobenzene	2.35 (2.19 - 2.64)	2.37 (± 0.14)	2.92 (2.83 - 3.21)	3 (± 0.19)	3.2 (3.02 - 3.46)	3.23 (± 0.16)
Liver & Kidney	[C] Ethionine	2.67 (1.18 - 2.84)	2.51 (± 0.49)	2.78 (2.7 - 2.86)	2.78 (± 0.06)	2.77 (2.69 - 2.85)	2.77 (± 0.06)
Liver & Kidney	[B] Hexachlorobutadiene (HCBD)			3.08 (2.98 - 3.33)	3.11 (± 0.14)	2.68 (2.63 - 2.98)	2.73 (± 0.14)

Target organ	Toxin	24 h post dose		48 h post dose		168 h post dose	
		Median (min - max)	Mean (± SD)	Median (min - max)	Mean (± SD)	Median (min - max)	Mean (± SD)
Liver & Kidney	[B] Mercuric chloride	2.91 (2.69 - 3.24)	2.95 (± 0.17)	2.94 (2.69 - 3.04)	2.93 (± 0.14)	2.74 (2.45 - 2.84)	2.71 (± 0.15)
Liver & Kidney	[E] Microcystin-LR			2.62 (2.52 - 2.77)	2.63 (± 0.11)	2.74 (2.67 - 2.82)	2.73 (± 0.06)
Liver & Kidney	[E] Rotenone	2.65 (2.58 - 2.94)	2.7 (± 0.11)	2.88 (2.76 - 3.21)	2.93 (± 0.22)	2.81 (2.81 - 2.84)	2.82 (± 0.01)
Liver & Kidney	[E] S-(1,2-dichlorovinyl)-cysteine (DCVC)	2.47 (2.19 - 2.86)	2.49 (± 0.19)	2.94 (2.91 - 3.01)	2.96 (± 0.05)	3.01 (2.99 - 3.06)	3.02 (± 0.03)
Liver & Kidney	[D] Thioacetamide	2.44 (2.19 - 2.46)	2.37 (± 0.1)	2.79 (2.5 - 2.9)	2.76 (± 0.16)	2.69 (2.65 - 2.91)	2.72 (± 0.11)
Pancreas	[E] 1-Cyano-2-hydroxy-3-butene	2.58 (2.35 - 2.75)	2.56 (± 0.16)	2.65 (2.65 - 2.77)	2.69 (± 0.05)	2.63 (2.55 - 2.73)	2.63 (± 0.07)
Pancreas	[C] Caerulin	2.68 (2.56 - 3)	2.7 (± 0.12)	2.86 (2.65 - 3.05)	2.86 (± 0.17)	2.72 (2.67 - 2.79)	2.73 (± 0.04)
Pancreas	[E] L-arginine	2.6 (0.28 - 3.23)	2.44 (± 0.69)	2.8 (2.7 - 2.9)	2.8 (± 0.08)	2.8 (2.73 - 2.88)	2.8 (± 0.05)
Pancreas	[B] Streptozotocin	2.78 (2.64 - 2.94)	2.79 (± 0.1)	2.94 (2.79 - 3.04)	2.92 (± 0.1)	2.82 (2.72 - 2.89)	2.81 (± 0.06)
Testicular	[D] 1,3-Dinitrobenzene	2.26 (2.19 - 2.4)	2.27 (± 0.06)	2.89 (2.67 - 2.99)	2.84 (± 0.13)	3.06 (2.99 - 3.25)	3.11 (± 0.13)
Testicular	[C] Cadmium chloride	2.55 (2.34 - 2.84)	2.57 (± 0.15)	2.97 (2.8 - 3.14)	2.97 (± 0.12)	2.98 (2.94 - 3.06)	2.99 (± 0.05)
Testicular	[D] Cadmium chloride	2.3 (2.14 - 2.49)	2.29 (± 0.1)	2.76 (2.38 - 2.97)	2.67 (± 0.25)	2.97 (2.93 - 3.06)	2.98 (± 0.05)
Testicular	[D] Carbendazim	2.6 (2.49 - 2.69)	2.59 (± 0.07)	3.03 (2.91 - 3.15)	3.02 (± 0.09)	3.04 (2.91 - 3.42)	3.1 (± 0.19)
Testicular	[D] Di-n-pentyl-phthalate	2.67 (2.59 - 2.74)	2.67 (± 0.05)	2.7 (2.67 - 2.78)	2.72 (± 0.05)	2.8 (2.75 - 2.9)	2.82 (± 0.06)
Testicular	[D] Ethane dimethane sulfonate (EDS)	2.62 (2.57 - 2.65)	2.62 (± 0.02)	2.9 (2.74 - 2.99)	2.89 (± 0.1)	3.41 (3.03 - 3.48)	3.34 (± 0.18)
Testicular	[D] Methoxyacetic acid	2.61 (2.51 - 2.68)	2.61 (± 0.05)	3.02 (2.88 - 3.16)	3.01 (± 0.1)	2.95 (2.8 - 3.2)	2.98 (± 0.15)
Multiple organ	[B] Adriamycin	2.76 (2.64 - 2.81)	2.75 (± 0.06)	2.74 (2.64 - 2.79)	2.72 (± 0.06)	2.69 (2.57 - 2.71)	2.66 (± 0.07)
Multiple organ	[C] Amphotericin B	2.63 (2.16 - 2.88)	2.63 (± 0.22)	2.76 (2.63 - 2.82)	2.74 (± 0.08)	2.75 (2.74 - 2.83)	2.78 (± 0.04)
Multiple organ	[C] Azaserine	2.78 (2.67 - 3.08)	2.79 (± 0.12)	2.88 (2.77 - 2.95)	2.86 (± 0.07)	2.73 (2.55 - 2.93)	2.76 (± 0.16)
Multiple organ	[A] Dexamethasone	2.47 (2.36 - 2.63)	2.48 (± 0.09)	2.97 (2.69 - 4.19)	3.14 (± 0.6)	3.53 (2.86 - 4.11)	3.49 (± 0.51)
Multiple organ	[E] Mitomycin-C	2.65 (2.56 - 2.84)	2.68 (± 0.11)	2.67 (2.64 - 2.74)	2.68 (± 0.04)	2.74 (2.62 - 2.76)	2.71 (± 0.06)

Target organ	Toxin	24 h post dose		48 h post dose		168 h post dose	
		Median (min - max)	Mean (± SD)	Median (min - max)	Mean (± SD)	Median (min - max)	Mean (± SD)
Physiological stressor	[C] 1,1-Dichloroethylene & maleic acid	2.85 (2.76 - 3.26)	2.89 (± 0.15)	2.76 (2.62 - 2.83)	2.74 (± 0.09)	2.79 (2.62 - 2.81)	2.74 (± 0.09)
Physiological stressor	[C] 2,4-Dinitrophenol	2.74 (1.98 - 2.85)	2.69 (± 0.25)	2.76 (2.66 - 2.87)	2.75 (± 0.08)	2.64 (2.29 - 2.68)	2.55 (± 0.17)
Physiological stressor	[B] 4-Pentenoic acid	2.75 (2.67 - 2.89)	2.76 (± 0.07)	2.64 (2.57 - 2.67)	2.64 (± 0.04)	2.76 (2.64 - 2.79)	2.73 (± 0.08)
Physiological stressor	[D] Acetazolamide	2.64 (2.53 - 2.85)	2.66 (± 0.1)	3.23 (2.86 - 3.5)	3.21 (± 0.24)	3.16 (2.98 - 3.62)	3.2 (± 0.25)
Physiological stressor	[C] Acivicin	2.81 (2.67 - 3.34)	2.86 (± 0.2)	2.75 (2.7 - 2.8)	2.75 (± 0.05)	2.68 (2.58 - 2.74)	2.67 (± 0.06)
Physiological stressor	[E] Ammonium chloride			2.74 (2.64 - 2.76)	2.72 (± 0.05)	2.74 (2.72 - 2.79)	2.75 (± 0.03)
Physiological stressor	[D] Carboplatin	2.43 (2.34 - 2.46)	2.43 (± 0.04)	3 (2.92 - 3.2)	3.02 (± 0.12)	3.04 (2.88 - 3.16)	3.04 (± 0.1)
Physiological stressor	[A] Choline and choline/methionine deficiency (chronic)	2.89 (2.49 - 3.39)	2.9 (± 0.31)	2.84 (2.84 - 2.94)	2.88 (± 0.05)	2.82 (2.64 - 2.89)	2.78 (± 0.13)
Physiological stressor	[B] Food restriction (chronic)	2.64 (2.52 - 2.69)	2.64 (± 0.06)	2.67 (2.64 - 2.69)	2.67 (± 0.03)	2.64 (2.57 - 2.67)	2.63 (± 0.04)
Physiological stressor	[D] Furosemide	2.49 (2.36 - 2.56)	2.48 (± 0.06)	3.02 (2.84 - 3.19)	3.03 (± 0.14)	2.94 (2.83 - 3.2)	2.96 (± 0.14)
Physiological stressor	[B] Insulin	2.7 (2.62 - 2.81)	2.7 (± 0.06)	2.72 (2.69 - 2.77)	2.72 (± 0.03)	2.64 (2.62 - 2.74)	2.65 (± 0.05)
Physiological stressor	[E] Methotrexate	2.7 (2.34 - 2.76)	2.67 (± 0.12)	2.64 (2.64 - 2.72)	2.67 (± 0.04)	2.76 (2.72 - 2.79)	2.76 (± 0.03)
Physiological stressor	[A] Partial hepatectomy	2.54 (2.44 - 2.67)	2.54 (± 0.08)	2.62 (2.52 - 2.84)	2.65 (± 0.13)	2.72 (2.59 - 2.86)	2.72 (± 0.1)
Physiological stressor	[A] Phenobarbital (chronic)	2.45 (2.34 - 2.69)	2.47 (± 0.1)	3 (2.91 - 3.1)	3 (± 0.08)	3.03 (3.02 - 3.09)	3.04 (± 0.03)
Physiological stressor	[A] Pregnenolone 16 alpha carbonitrile (chronic)						
Physiological stressor	[A] Probenecid	2.53 (2.44 - 2.64)	2.53 (± 0.06)	3.01 (2.92 - 3.11)	3.01 (± 0.07)	2.82 (2.59 - 2.89)	2.76 (± 0.12)
Physiological stressor	[C] Rosiglitazone	2.72 (2.53 - 2.96)	2.72 (± 0.15)	2.63 (2.45 - 2.68)	2.59 (± 0.1)	2.72 (2.51 - 2.78)	2.68 (± 0.11)
Physiological stressor	[C] Rosiglitazone (chronic)	2.64 (2.42 - 2.74)	2.61 (± 0.1)				
Physiological stressor	[E] Sodium bicarbonate	2.6 (2.58 - 2.64)	2.61 (± 0.02)				
Physiological stressor	[A] Unilateral nephrectomy	2.75 (2.65 - 2.85)	2.75 (± 0.06)	2.8 (2.65 - 3.1)	2.83 (± 0.18)	2.95 (2.85 - 3.03)	2.93 (± 0.08)

Target organ	Toxin	24 h post dose		48 h post dose		168 h post dose	
		Median (min - max)	Mean (± SD)	Median (min - max)	Mean (± SD)	Median (min - max)	Mean (± SD)
Physiological stressor	B Water deprivation (chronic)	2.72 (2.66 - 2.86)	2.74 (± 0.06)	2.74 (2.67 - 2.77)	2.73 (± 0.04)	2.65 (2.54 - 2.89)	2.69 (± 0.12)
No Effect	E Acetaminophen (chronic)	2.78 (2.7 - 2.8)	2.76 (± 0.04)			2.7 (2.68 - 2.8)	2.72 (± 0.05)
No Effect	C Buthionine sulphoxime	2.75 (2.52 - 3.4)	2.82 (± 0.28)	2.69 (2.6 - 2.9)	2.74 (± 0.12)	2.65 (2.56 - 2.73)	2.66 (± 0.06)
No Effect	C Ferrous sulphate	2.67 (2.48 - 2.87)	2.67 (± 0.12)	2.68 (2.52 - 2.84)	2.69 (± 0.12)	2.7 (2.67 - 2.75)	2.71 (± 0.03)
No Effect	B Ifosfamide	2.7 (2.65 - 2.78)	2.7 (± 0.04)	2.48 (2.48 - 2.55)	2.51 (± 0.04)	2.75 (2.65 - 2.8)	2.75 (± 0.06)
No Effect	B Lithocholic acid	2.83 (2.78 - 2.93)	2.83 (± 0.04)	2.65 (2.58 - 2.75)	2.66 (± 0.06)	2.73 (2.68 - 2.83)	2.75 (± 0.06)
No Effect	E Paraquat			2.74 (2.72 - 2.84)	2.76 (± 0.05)	2.76 (2.74 - 2.81)	2.77 (± 0.03)
No Effect	D Potassium dichromate	1.89 (1.61 - 2.13)	1.88 (± 0.16)	3.21 (2.82 - 3.32)	3.16 (± 0.2)	2.99 (2.3 - 3.18)	2.88 (± 0.35)
No Effect	C Trichlorethylene	2.71 (2.63 - 2.94)	2.74 (± 0.1)	2.9 (2.78 - 2.91)	2.86 (± 0.05)	2.75 (2.72 - 2.86)	2.77 (± 0.06)

A-F: Indicates Pharmaceutical Company & sample origin

Supplementary Table S21. Summary statistics for serum sodium (mmol/L) at 24 hrs, 48 hrs and 168 hrs post high dose.

Target organ	Toxin	24 h post dose		48 h post dose		168 h post dose	
		Median (min - max)	Mean (± SD)	Median (min - max)	Mean (± SD)	Median (min - max)	Mean (± SD)
Liver	E 1,1-Dichloroethylene	143.5 (142.24 - 144.88)	143.42 (± 0.81)	144 (143 - 145)	143.8 (± 0.84)	144 (140 - 145)	143.2 (± 2.17)
Liver	E 1,2,3,4,5,6-hexachlorocyclohexane	143.61 (142.98 - 144.51)	143.6 (± 0.55)	144 (142 - 145)	144 (± 1.22)	143 (141 - 144)	142.6 (± 1.14)
Liver	B 1-Fluoropentane	144.5 (142 - 147)	144.8 (± 1.69)	144 (144 - 146)	144.6 (± 0.89)	144 (143 - 144)	143.8 (± 0.45)
Liver	B 2,4,6-Trihydroxyacetophenone (THA)	144.5 (141 - 146)	144.1 (± 1.52)	144 (142 - 145)	144 (± 1.22)	142 (140 - 143)	141.6 (± 1.14)
Liver	B 4-Amino-2,6-dichlorophenol (ADCP)	145.79 (143.29 - 147.29)	145.49 (± 1.48)	146.29 (144.29 - 147.29)	146.09 (± 1.3)	143.29 (142.29 - 143.29)	143.09 (± 0.45)
Liver	C Aflatoxin	146.65 (144.05 - 149.65)	147.08 (± 1.88)	142.95 (140.75 - 145.95)	142.99 (± 2.22)	143.85 (143.45 - 144.95)	144.01 (± 0.59)

Target organ	Toxin	24 h post dose		48 h post dose		168 h post dose	
		Median (min - max)	Mean (± SD)	Median (min - max)	Mean (± SD)	Median (min - max)	Mean (± SD)
Liver	[C] Allyl alcohol	144.25 (141 - 146.2)	144.17 (± 1.49)	144.1 (141.6 - 144.8)	143.44 (± 1.44)	144.3 (143.5 - 147.1)	144.78 (± 1.47)
Liver	[C] Allyl formate	142.6 (139.9 - 144.1)	142.32 (± 1.34)	144 (143.4 - 144.4)	143.98 (± 0.36)	143.8 (143.51 - 146.1)	144.44 (± 1.15)
Liver	[B] Azathioprine	146.29 (143.29 - 148.29)	146.19 (± 1.37)	145.29 (144.29 - 146.29)	145.29 (± 0.71)	144.29 (141.29 - 146.29)	143.89 (± 2.07)
Liver	[B] Bromobenzene	143 (140 - 146)	142.9 (± 1.66)	141 (139 - 145)	141.6 (± 2.19)	143 (141 - 144)	142.6 (± 1.14)
Liver	[C] Butylated hydroxytoluene	142.95 (140.05 - 145.25)	143.05 (± 1.75)	140.45 (139.95 - 141.35)	140.51 (± 0.54)	143.25 (141.15 - 143.95)	142.83 (± 1.23)
Liver	[D] Carbon tetrachloride	142.91 (142.06 - 143.5)	142.82 (± 0.58)	142 (141 - 143)	141.8 (± 0.84)	142 (141 - 145)	142.2 (± 1.64)
Liver	[C] Chlorpromazine	146.1 (144.35 - 148.45)	146.12 (± 1.38)	144.95 (143.35 - 146.35)	144.83 (± 1.08)	143.05 (142.45 - 143.25)	142.95 (± 0.32)
Liver	[B] Clofibrate	143.5 (141 - 145)	143.5 (± 1.51)	143 (140 - 144)	142.4 (± 1.52)	143 (142 - 143)	142.6 (± 0.55)
Liver	[B] Cyproterone acetate	144 (143 - 145)	144 (± 0.94)	144 (142 - 148)	144.2 (± 2.28)	143 (142 - 145)	143.2 (± 1.1)
Liver	[A] D-galactosamine	141.77 (136.65 - 146.55)	141.83 (± 3.36)	143.75 (142.12 - 149.35)	144.86 (± 2.85)	134.75 (115.65 - 147.55)	133.55 (± 14.37)
Liver	[B] Diethylhexylphthalate (DEHP)			143 (140 - 146)	142.8 (± 2.39)	143 (143 - 145)	143.4 (± 0.89)
Liver	[C] Dimethylformamide (DMF)	142.6 (140.55 - 144.35)	142.46 (± 1.05)	143.85 (142.15 - 145.95)	144.07 (± 1.41)	144.85 (140.55 - 146.55)	144.29 (± 2.23)
Liver	[C] Dimethylnitrosamine (DMN)	141.35 (137.75 - 144.25)	141.3 (± 2.02)	143.35 (135.55 - 147.95)	143.19 (± 4.72)	142.65 (140.65 - 145.05)	142.81 (± 2.01)
Liver	[A] Gadolinium chloride	141.5 (139.8 - 143)	141.57 (± 0.86)	143.5 (140.7 - 145.8)	143.4 (± 1.94)	147.3 (145.1 - 148.1)	147.12 (± 1.19)
Liver	[A, B, C, D, F] Hydrazine	145.45 (137 - 150.45)	145.16 (± 2.41)	141.5 (138.25 - 147.5)	142.32 (± 2.78)	144 (140.5 - 147)	143.69 (± 1.94)
Liver	[E] Hydrazine	149 (146 - 150)	148.3 (± 1.25)	144 (143 - 146)	144.25 (± 1.5)	143 (141 - 146)	143 (± 1.87)
Liver	[E] Indomethacin	142 (141.11 - 145)	142.39 (± 1.24)	143 (140 - 145)	142.6 (± 2.07)	144.02 (142 - 147)	144.4 (± 2.07)
Liver	[E] Ketoconazole			144 (142 - 145)	143.6 (± 1.52)	142 (140 - 143)	141.4 (± 1.34)
Liver	[C] Lead acetate	140.7 (135.85 - 144.45)	140.31 (± 3.34)	140.05 (130.55 - 143.7)	139.1 (± 5.13)	143.25 (141.65 - 146.05)	143.47 (± 1.68)
Liver	[A] Lipopolysaccharide (LPS)	143 (139.7 - 144.3)	142.78 (± 1.34)	145.4 (143.62 - 147.2)	145.42 (± 1.27)	147 (145.1 - 148.1)	146.74 (± 1.1)

Target organ	Toxin	24 h post dose		48 h post dose		168 h post dose	
		Median (min - max)	Mean (± SD)	Median (min - max)	Mean (± SD)	Median (min - max)	Mean (± SD)
Liver	B Methapyrilene	142.5 (140 - 144)	142.2 (± 1.23)	143 (143 - 145)	143.6 (± 0.89)	144 (143 - 145)	144 (± 0.71)
Liver	E Methylene dianiline	142.83 (141.24 - 143.66)	142.59 (± 0.75)	144 (142 - 145)	143.6 (± 1.14)	143 (142 - 144)	142.8 (± 0.84)
Liver	C Monocrotaline	142.65 (140.15 - 145.35)	142.46 (± 1.52)	144.75 (143.45 - 146.75)	144.93 (± 1.31)	145.05 (144.05 - 146.95)	145.33 (± 1.17)
Liver	C N-methylformamide (NMF)	142 (140.1 - 143.3)	141.75 (± 1.23)	144.2 (141.5 - 144.8)	143.82 (± 1.33)	143.5 (141.8 - 144.6)	143.46 (± 1.17)
Liver	D Phalloidin (chronic)			141.75 (140.25 - 142.25)	141.35 (± 0.85)	146.85 (145.05 - 149.05)	146.94 (± 1.76)
Liver	E Phenyl diisothiocyanate	143.71 (141.66 - 145.69)	143.63 (± 1.29)	135.5 (133.5 - 140.5)	136.1 (± 2.7)	144.02 (141.5 - 144.51)	143.67 (± 1.25)
Liver	E Phenyl isothiocyanate	142.72 (141.48 - 143.46)	142.59 (± 0.65)	144 (144 - 145)	144.2 (± 0.45)	142 (142 - 145)	143.2 (± 1.64)
Liver	B Retinyl palmitate	144.29 (142.29 - 146.29)	144.49 (± 1.23)	143.29 (142.29 - 144.29)	143.09 (± 0.84)	143.29 (143.29 - 144.29)	143.69 (± 0.55)
Liver	B Sodium Valproate			140.5 (136.5 - 143.59)	140.12 (± 2.73)	142.5 (142.5 - 144.5)	143.11 (± 0.9)
Liver	C a-Naphthylisothiocyanate (ANIT)	144.35 (141.3 - 147.8)	144.7 (± 1.86)	144.2 (142.4 - 144.6)	143.94 (± 0.89)	142.6 (140.5 - 142.8)	142.24 (± 0.98)
Kidney	D 2-Bromophenol	142 (140 - 144)	142 (± 1.15)	143 (141 - 146)	143.4 (± 2.07)	145 (143 - 147)	144.8 (± 1.48)
Kidney	E 3,5-Dichloroaniline hydrochloride	142.66 (141.72 - 143.16)	142.53 (± 0.49)	143.5 (143.5 - 146.5)	144.3 (± 1.3)	142.5 (141.5 - 143.5)	142.7 (± 0.84)
Kidney	E Atractyloside	142.33 (138.5 - 145.06)	142.3 (± 1.65)	143.5 (143.5 - 146.5)	144.3 (± 1.3)	144.5 (143.5 - 144.5)	144.1 (± 0.55)
Kidney	D Bromoethylamine hydrobromide	145 (142 - 149)	144.9 (± 2.03)	141 (140 - 143)	141.2 (± 1.3)	141 (138 - 146.4)	140.88 (± 3.43)
Kidney	D Cephaloridine	141.5 (141 - 143)	141.6 (± 0.7)	147 (147 - 147)	147 (± 0)	146 (146 - 148)	146.6 (± 0.89)
Kidney	B Chlorethanamine	146 (144 - 149)	145.7 (± 1.42)	142 (141 - 144)	142 (± 1.22)	143 (142 - 144)	143 (± 0.71)
Kidney	A Cisplatin	141 (140.5 - 143.44)	141.39 (± 1.09)	142.5 (140.5 - 144.5)	142.5 (± 1.58)	140.5 (140.5 - 142.5)	141.1 (± 0.89)
Kidney	A D-limonene (chronic)						
Kidney	E Dichlorophenyl succinimide	142.12 (140.05 - 144.35)	142.24 (± 1.25)	144 (144 - 146)	144.4 (± 0.89)	144 (144 - 145)	144.2 (± 0.45)
Kidney	D Ethylene glycol	141 (139.5 - 143.5)	141.1 (± 1.58)	141.5 (140.5 - 143.5)	141.5 (± 1.22)	144.5 (143.5 - 149.5)	145.3 (± 2.49)

Target organ	Toxin	24 h post dose		48 h post dose		168 h post dose	
		Median (min - max)	Mean (± SD)	Median (min - max)	Mean (± SD)	Median (min - max)	Mean (± SD)
Kidney	[A] Folic acid	138 (136 - 141)	138.3 (± 1.7)	143.71 (132 - 148)	142.07 (± 5.57)	146 (144.36 - 148)	145.77 (± 1.48)
Kidney	[A] Gentamicin	140 (139 - 142)	140.1 (± 0.99)	147 (142 - 148)	145.6 (± 2.51)	146 (144 - 147)	145.6 (± 1.14)
Kidney	[B] Maleic acid	140.29 (136.29 - 144.29)	140.49 (± 2.57)	142.29 (140.29 - 144.29)	142.29 (± 1.58)	142.29 (142.29 - 144.29)	142.89 (± 0.89)
Kidney	[A] N-phenylanthranilic acid (chronic)	139.1 (138 - 141.95)	139.68 (± 1.54)	145.5 (145.1 - 162.3)	148.88 (± 7.51)	146 (144.22 - 147.7)	145.98 (± 1.67)
Kidney	[D] Para-aminophenol	139 (138 - 140)	139.3 (± 0.67)	145 (139 - 146)	143.8 (± 2.95)	147 (144 - 151)	147.2 (± 2.49)
Kidney	[A] Puromycin			142.5 (141.5 - 143.5)	142.5 (± 0.71)	144.5 (143.5 - 146.5)	144.9 (± 1.52)
Kidney	[B] Vancomycin hydrochloride			142.5 (140.5 - 144.5)	142.7 (± 1.48)	141.5 (141.5 - 145.5)	142.5 (± 1.73)
Liver & Kidney	[E] Acetaminophen	142.84 (142.13 - 143.84)	142.82 (± 0.55)	141 (138 - 142)	140.4 (± 1.52)	144 (144 - 146)	144.6 (± 0.89)
Liver & Kidney	[B] Aurothiomalate	143.29 (139.29 - 148.29)	143.69 (± 3.06)	140.29 (135.29 - 144.29)	140.49 (± 3.56)	144.29 (143.29 - 146.29)	144.69 (± 1.52)
Liver & Kidney	[C] Chloroform	142.7 (138.7 - 148.7)	143.24 (± 2.59)	144.6 (142.8 - 146.8)	144.76 (± 1.42)	142.2 (141.4 - 144.8)	142.82 (± 1.39)
Liver & Kidney	[D] Cyclosporin	139.5 (137.5 - 144.37)	139.79 (± 1.97)	145.01 (142.5 - 147.5)	144.84 (± 1.75)	147.5 (145.4 - 148.5)	146.88 (± 1.37)
Liver & Kidney	[D] Dichlorobenzene	137.5 (135 - 145)	139.1 (± 3.6)	143 (140 - 146)	142.6 (± 2.3)	146 (144 - 146)	145.4 (± 0.89)
Liver & Kidney	[C] Ethionine	141.55 (138.45 - 144.65)	141.44 (± 1.86)	143.35 (141.85 - 144.15)	143.01 (± 1.01)	144.05 (141.95 - 145.65)	143.91 (± 1.46)
Liver & Kidney	[B] Hexachlorobutadiene (HCBD)			141 (139 - 142)	140.6 (± 1.14)	145 (142 - 146)	144 (± 1.87)
Liver & Kidney	[B] Mercuric chloride	143 (134 - 147)	142.5 (± 4.01)	142 (139 - 145)	142.4 (± 2.3)	142.67 (141 - 143.56)	142.45 (± 0.99)
Liver & Kidney	[E] Microcystin-LR			142 (142 - 143.12)	142.42 (± 0.58)	143 (142 - 144.51)	143.3 (± 0.98)
Liver & Kidney	[E] Rotenone	142.98 (142.33 - 144.04)	143.06 (± 0.6)	143.06 (138 - 144)	142.03 (± 2.72)	143.9 (143 - 144.51)	143.72 (± 0.61)
Liver & Kidney	[E] S-(1,2-dichlorovinyl)-cysteine (DCVC)	142.1 (140.82 - 143.99)	142.27 (± 0.85)	143 (143 - 145)	143.6 (± 0.89)	141 (141 - 144)	141.6 (± 1.34)
Liver & Kidney	[D] Thioacetamide	139.5 (138 - 140)	139.2 (± 0.92)	145 (140 - 147)	144 (± 2.92)	149 (145 - 151)	148.2 (± 2.28)
Pancreas	[E] 1-Cyano-2-hydroxy-3-butene	143.14 (141.11 - 144.13)	142.92 (± 0.97)	143 (142 - 143.12)	142.82 (± 0.46)	147 (145 - 148)	147 (± 1.22)

Target organ	Toxin	24 h post dose		48 h post dose		168 h post dose	
		Median (min - max)	Mean (± SD)	Median (min - max)	Mean (± SD)	Median (min - max)	Mean (± SD)
Pancreas	[C] Caerulin	140.4 (138.75 - 143.95)	140.9 (± 1.84)	148.05 (146.85 - 148.25)	147.85 (± 0.58)	141.85 (141.05 - 143.95)	142.34 (± 1.4)
Pancreas	[E] L-arginine	143.02 (138.78 - 144.26)	142.69 (± 1.4)	144 (142 - 147)	144.27 (± 1.53)	145 (142 - 147)	144.6 (± 1.71)
Pancreas	[B] Streptozotocin	141.5 (139 - 145)	141.7 (± 1.77)	142 (141 - 144)	142 (± 1.22)	142 (141 - 147)	143 (± 2.35)
Testicular	[D] 1,3-Dinitrobenzene	140 (138 - 146)	140.4 (± 2.12)	129 (123 - 145)	131.4 (± 8.62)	147 (145 - 148)	147 (± 1.22)
Testicular	[C] Cadmium chloride	141.8 (132.55 - 149.15)	140.52 (± 4.82)	149.55 (138.95 - 153.65)	148.21 (± 5.74)	145.05 (143.25 - 147.25)	145.09 (± 1.48)
Testicular	[D] Cadmium chloride	141.15 (136.7 - 143.9)	140.61 (± 1.99)	143.5 (141.7 - 145.8)	143.52 (± 1.6)	144.3 (142.2 - 145.5)	144.16 (± 1.36)
Testicular	[D] Carbendazim	142.5 (140 - 143)	142.1 (± 1.1)	145 (144 - 148)	145.2 (± 1.64)	144 (142 - 145)	143.8 (± 1.3)
Testicular	[D] Di-n-pentyl-phthalate	142.35 (141.5 - 143.7)	142.46 (± 0.77)	145.4 (144.1 - 148.4)	145.96 (± 1.87)	146.3 (143.9 - 147.2)	145.78 (± 1.35)
Testicular	[D] Ethane dimethane sulfonate (EDS)	141 (139 - 143)	140.8 (± 1.14)	147 (146 - 149)	147.2 (± 1.3)	144 (142 - 144)	143.2 (± 1.1)
Testicular	[D] Methoxyacetic acid	140.05 (138.05 - 140.35)	139.61 (± 0.86)	144.95 (142.35 - 146.15)	144.63 (± 1.53)	145.75 (143.25 - 148.45)	145.85 (± 1.98)
Multiple organ	[B] Adriamycin	142.5 (142 - 145)	142.9 (± 1.1)	143 (141 - 145)	143.2 (± 1.48)	143 (142 - 145)	143.4 (± 1.14)
Multiple organ	[C] Amphotericin B	143.75 (137.75 - 153.85)	144.92 (± 4.87)	143.05 (139.15 - 145.25)	142.82 (± 2.31)	141.05 (139.05 - 143.95)	141.21 (± 1.85)
Multiple organ	[C] Azaserine	140.5 (134.85 - 144.75)	140.31 (± 2.55)	140.55 (140.05 - 143.75)	141.09 (± 1.52)	145.85 (143.85 - 147.05)	145.47 (± 1.45)
Multiple organ	[A] Dexamethasone	140 (135.5 - 141.5)	139.26 (± 2.2)	142.5 (135.5 - 143.5)	140.7 (± 3.56)	144.4 (141.5 - 153.5)	146.08 (± 4.68)
Multiple organ	[E] Mitomycin-C	144.25 (141.61 - 145.93)	144.05 (± 1.19)	144 (143 - 145)	143.8 (± 0.84)	143 (142 - 144)	142.8 (± 0.84)
Physiological stressor	[C] 1,1-Dichloroethylene & maleic acid	144.45 (142.2 - 147)	144.54 (± 1.48)	143.9 (142.4 - 147.4)	144.06 (± 2.02)	145.2 (143.6 - 146.7)	145.2 (± 1.22)
Physiological stressor	[C] 2,4-Dinitrophenol	142.5 (140.4 - 144.3)	142.52 (± 1.33)	143.1 (142.8 - 143.9)	143.26 (± 0.44)	147.8 (145.3 - 149.3)	147.52 (± 1.63)
Physiological stressor	[B] 4-Pentenoic acid	144 (143 - 146)	144.06 (± 0.96)	140 (139 - 143.59)	140.32 (± 1.9)	142 (141 - 143)	142 (± 1)
Physiological stressor	[D] Acetazolamide	144.5 (140.98 - 146.5)	144.25 (± 1.91)	142.5 (140.5 - 144.52)	142.9 (± 1.68)	140.5 (138.5 - 141.5)	140.3 (± 1.3)
Physiological stressor	[C] Acivicin	144.65 (142.45 - 147.65)	144.59 (± 1.42)	142.95 (141.55 - 144.45)	143.17 (± 1.2)	142.15 (141.65 - 145.35)	143.19 (± 1.72)
Physiological stressor	[E] Ammonium chloride			143 (143 - 144)	143.2 (± 0.45)	144 (143 - 145)	143.8 (± 0.84)

Target organ	Toxin	24 h post dose		48 h post dose		168 h post dose	
		Median (min - max)	Mean (± SD)	Median (min - max)	Mean (± SD)	Median (min - max)	Mean (± SD)
Physiological stressor	[D] Carboplatin	139.5 (138 - 142)	139.7 (± 1.34)	146 (144 - 147)	145.8 (± 1.3)	146 (144 - 147)	146 (± 1.22)
Physiological stressor	[A] Choline and choline/methionine deficiency (chronic)	131.5 (118 - 136)	130.7 (± 5.6)	144 (143 - 145)	144.2 (± 0.84)	144 (144 - 145)	144.4 (± 0.55)
Physiological stressor	[B] Food restriction (chronic)	143.5 (141.5 - 144.5)	143.17 (± 0.87)	145 (144.5 - 146.5)	145.25 (± 0.96)	144.5 (143.5 - 145.5)	144.3 (± 0.84)
Physiological stressor	[D] Furosemide	141.5 (137.5 - 142.5)	140.9 (± 1.35)	146.5 (143.5 - 147.5)	145.7 (± 1.64)	147.5 (141.5 - 148.5)	145.7 (± 3.42)
Physiological stressor	[B] Insulin	144 (142 - 145)	143.5 (± 0.97)	145 (144 - 145)	144.6 (± 0.55)	142 (142 - 145)	142.8 (± 1.3)
Physiological stressor	[E] Methotrexate	143.3 (142.56 - 144.4)	143.4 (± 0.59)	144 (143 - 146)	144 (± 1.22)	143 (142 - 144)	143 (± 1)
Physiological stressor	[A] Partial hepatectomy	141.75 (139.4 - 144.8)	141.55 (± 1.71)	140.7 (139.5 - 143.2)	140.96 (± 1.47)	144.1 (139.5 - 146.3)	143.52 (± 2.48)
Physiological stressor	[A] Phenobarbital (chronic)	142 (140 - 145)	142.4 (± 1.43)	147 (144 - 148)	146.8 (± 1.64)	146 (144 - 148)	146.4 (± 1.67)
Physiological stressor	[A] Pregnenolone 16 alpha carbonitrile (chronic)						
Physiological stressor	[A] Probenecid	141.8 (140.4 - 144.1)	141.87 (± 1.08)	145.4 (144.5 - 147.4)	145.58 (± 1.12)	146.2 (144.8 - 149)	146.72 (± 1.67)
Physiological stressor	[C] Rosiglitazone	143.75 (141.65 - 146.65)	143.94 (± 1.37)	142.95 (140.65 - 143.45)	142.63 (± 1.16)	142.45 (142.35 - 144.35)	143.19 (± 1.06)
Physiological stressor	[C] Rosiglitazone (chronic)	143.15 (140.35 - 146.15)	143.08 (± 1.93)				
Physiological stressor	[E] Sodium bicarbonate	143.47 (142.75 - 144.04)	143.43 (± 0.46)				
Physiological stressor	[A] Unilateral nephrectomy	143.25 (140.45 - 145.65)	143.24 (± 1.51)	137.05 (131.55 - 147.75)	138.69 (± 6.09)	142.65 (140.95 - 143.55)	142.51 (± 0.96)
Physiological stressor	[B] Water deprivation (chronic)	147 (143.65 - 147)	146.24 (± 1.24)	149.5 (143.9 - 152)	148.98 (± 2.86)	144.5 (143 - 146)	144.49 (± 1.06)
No Effect	[E] Acetaminophen (chronic)	143 (141 - 144)	142.8 (± 1.3)			143 (141 - 145)	142.6 (± 1.67)
No Effect	[C] Buthionine sulphoxime	141.8 (138.7 - 145.5)	142.31 (± 2.04)	143.9 (138.5 - 148.5)	144.18 (± 3.83)	143.7 (142.6 - 145.4)	143.82 (± 1.19)
No Effect	[C] Ferrous sulphate	143.45 (140 - 146.2)	143.29 (± 1.99)	144 (142.2 - 147.6)	144.76 (± 2.11)	144.9 (143.1 - 146.1)	144.62 (± 1.15)
No Effect	[B] Ifosfamide	144 (143 - 146)	144.3 (± 0.82)	144 (142 - 145)	143.6 (± 1.52)	142 (141 - 142)	141.6 (± 0.55)
No Effect	[B] Lithocholic acid	143 (143 - 147)	144 (± 1.49)	143 (141 - 144)	142.8 (± 1.3)	144 (142 - 144)	143.4 (± 0.89)

Target organ	Toxin	24 h post dose		48 h post dose		168 h post dose	
		Median (min - max)	Mean (± SD)	Median (min - max)	Mean (± SD)	Median (min - max)	Mean (± SD)
No Effect	[E] Paraquat			142.5 (141.5 - 144.5)	142.9 (± 1.14)	143.5 (141.5 - 144.5)	142.9 (± 1.34)
No Effect	[D] Potassium dichromate	141.5 (139 - 143)	141.3 (± 1.34)	144 (143 - 145)	144.2 (± 0.84)	145 (141 - 147)	144.4 (± 2.41)
No Effect	[C] Trichlorethylene	140.95 (138.45 - 145.65)	141.05 (± 2.04)	147.15 (145.95 - 147.95)	146.99 (± 0.91)	145.95 (145.05 - 148.55)	146.57 (± 1.41)

A-F: Indicates Pharmaceutical Company & sample origin

Supplementary Table S22. Summary statistics for serum potassium (mmol/L) at 24 hrs, 48 hrs and 168 hrs post high dose.

Target organ	Toxin	24 h post dose		48 h post dose		168 h post dose	
		Median (min - max)	Mean (± SD)	Median (min - max)	Mean (± SD)	Median (min - max)	Mean (± SD)
Liver	[E] 1,1-Dichloroethylene	6.16 (6 - 6.33)	6.17 (± 0.1)	6.4 (6.1 - 6.6)	6.36 (± 0.21)	6.1 (5.9 - 6.4)	6.14 (± 0.21)
Liver	[E] 1,2,3,4,5,6-hexachlorocyclohexane	6.45 (6.25 - 7.31)	6.61 (± 0.36)	6.05 (5.75 - 6.45)	6.07 (± 0.26)	6.65 (6.05 - 7.05)	6.59 (± 0.4)
Liver	[B] 1-Fluoropentane	5.9 (5.4 - 6.1)	5.8 (± 0.25)	5.8 (5.2 - 6.2)	5.64 (± 0.43)	6.1 (5.8 - 6.2)	6.06 (± 0.17)
Liver	[B] 2,4,6-Trihydroxyacetophenone (THA)	6 (5.6 - 6.3)	5.98 (± 0.25)	6 (5.8 - 6.2)	6 (± 0.16)	6.1 (6 - 6.4)	6.16 (± 0.18)
Liver	[B] 4-Amino-2,6-dichlorophenol (ADCP)	5.77 (5.62 - 6.32)	5.87 (± 0.27)	6.02 (5.52 - 6.22)	5.94 (± 0.28)	6.22 (6.02 - 6.62)	6.32 (± 0.24)
Liver	[C] Aflatoxin	5.96 (5.48 - 7.3)	6.06 (± 0.6)	6.68 (5.63 - 6.81)	6.42 (± 0.48)	7.18 (6.03 - 7.79)	6.92 (± 0.78)
Liver	[C] Allyl alcohol	5.44 (4.11 - 6.16)	5.43 (± 0.66)	5.78 (4.92 - 6.1)	5.68 (± 0.48)	6.51 (6.14 - 6.85)	6.52 (± 0.32)
Liver	[C] Allyl formate	5.72 (5.06 - 6.58)	5.81 (± 0.54)	6.24 (6.06 - 6.68)	6.35 (± 0.27)	6.91 (5.82 - 7.57)	6.69 (± 0.7)
Liver	[B] Azathioprine	5.42 (5.12 - 5.92)	5.49 (± 0.27)	5.52 (4.82 - 5.62)	5.4 (± 0.33)	6.22 (6.02 - 6.62)	6.28 (± 0.24)
Liver	[B] Bromobenzene	5.95 (5.6 - 6.7)	5.96 (± 0.31)	6.1 (6 - 6.3)	6.1 (± 0.12)	6 (5.9 - 6.9)	6.16 (± 0.42)
Liver	[C] Butylated hydroxytoluene	5.48 (4.72 - 6.13)	5.49 (± 0.46)	5.84 (5.06 - 6.29)	5.78 (± 0.51)	6.26 (6 - 6.71)	6.31 (± 0.28)
Liver	[D] Carbon tetrachloride	5.54 (5.41 - 5.81)	5.57 (± 0.13)	6.55 (5.35 - 6.95)	6.37 (± 0.61)	5.35 (4.85 - 6.35)	5.59 (± 0.63)
Liver	[C] Chlorpromazine	5.27 (4.28 - 6.84)	5.33 (± 0.75)	6.52 (4.78 - 6.66)	6.18 (± 0.78)	5.94 (5.26 - 6.24)	5.83 (± 0.39)

Target organ	Toxin	24 h post dose		48 h post dose		168 h post dose	
		Median (min - max)	Mean (± SD)	Median (min - max)	Mean (± SD)	Median (min - max)	Mean (± SD)
Liver	[B] Clofibrate	5.7 (5.2 - 5.9)	5.64 (± 0.22)	5.9 (5.3 - 6.2)	5.78 (± 0.37)	6.3 (5.7 - 6.7)	6.34 (± 0.41)
Liver	[B] Cyproterone acetate	5.95 (5.4 - 6.4)	5.86 (± 0.32)	6.4 (6 - 7.1)	6.46 (± 0.4)	6.5 (6 - 6.6)	6.34 (± 0.31)
Liver	[A] D-galactosamine	5.65 (5.26 - 5.98)	5.63 (± 0.26)	8.44 (5.39 - 10.12)	8.25 (± 1.77)	5.46 (4.46 - 6.56)	5.58 (± 0.93)
Liver	[B] Diethylhexylphthalate (DEHP)			5.9 (5.7 - 6.9)	6.22 (± 0.58)	6.5 (6.4 - 7)	6.66 (± 0.27)
Liver	[C] Dimethylformamide (DMF)	5.8 (5.18 - 6.66)	5.9 (± 0.44)	5.82 (5.66 - 6.2)	5.86 (± 0.2)	6.64 (6.22 - 6.76)	6.52 (± 0.26)
Liver	[C] Dimethylnitrosamine (DMN)	5.22 (4.43 - 6.47)	5.33 (± 0.7)	5.34 (4.63 - 6.01)	5.28 (± 0.62)	6.74 (5.79 - 6.91)	6.42 (± 0.54)
Liver	[A] Gadolinium chloride	6.12 (5.6 - 6.44)	6.04 (± 0.28)	6.24 (5.47 - 8.57)	6.86 (± 1.53)	6.02 (5.59 - 7.31)	6.23 (± 0.73)
Liver	[A, B, C, D, F] Hydrazine	5.94 (5.2 - 7.24)	6.05 (± 0.55)	6.6 (5.05 - 10.95)	6.91 (± 1.38)	6.02 (4.8 - 7.05)	5.98 (± 0.65)
Liver	[E] Hydrazine	8 (6.25 - 9.55)	7.75 (± 1.06)	5.4 (5.15 - 5.55)	5.38 (± 0.21)	5.45 (4.55 - 6.35)	5.51 (± 0.65)
Liver	[E] Indomethacin	6.3 (5.25 - 7.17)	6.22 (± 0.75)	5.35 (4.75 - 5.55)	5.27 (± 0.31)	6.53 (5.65 - 9.65)	6.89 (± 1.61)
Liver	[E] Ketoconazole			5.65 (5.25 - 6.15)	5.71 (± 0.33)	6.35 (6.05 - 6.45)	6.29 (± 0.15)
Liver	[C] Lead acetate	5.88 (5.14 - 6.55)	5.85 (± 0.53)	5.6 (4.93 - 6.29)	5.58 (± 0.49)	7.21 (6.66 - 8.73)	7.38 (± 0.79)
Liver	[A] Lipopolysaccharide (LPS)	4.91 (4.67 - 5.94)	5.02 (± 0.38)	5.59 (5.35 - 6.3)	5.77 (± 0.39)	6.51 (6.24 - 7.33)	6.6 (± 0.42)
Liver	[B] Methapyrilene	6.55 (6.45 - 6.95)	6.68 (± 0.22)	5.75 (5.35 - 6.05)	5.77 (± 0.27)	5.45 (5.25 - 6.05)	5.53 (± 0.33)
Liver	[E] Methylene dianiline	6.58 (5.71 - 7.06)	6.51 (± 0.45)	6.8 (6.1 - 7)	6.62 (± 0.36)	6.3 (6 - 6.8)	6.36 (± 0.3)
Liver	[C] Monocrotaline	5.96 (5.08 - 6.78)	5.98 (± 0.47)	5.56 (5.06 - 5.73)	5.46 (± 0.28)	5.74 (5.56 - 6.12)	5.79 (± 0.24)
Liver	[C] N-methylformamide (NMF)	5.82 (5.13 - 6.85)	5.81 (± 0.52)	5.95 (5.59 - 6.93)	6.03 (± 0.53)	6.52 (5.65 - 6.7)	6.39 (± 0.42)
Liver	[D] Phalloidin (chronic)			6.38 (6.29 - 7.14)	6.51 (± 0.35)	7.06 (5.92 - 8.5)	7 (± 1.07)
Liver	[E] Phenyl diisothiocyanate	6.38 (5.85 - 7.43)	6.46 (± 0.47)	7.6 (6.8 - 8.1)	7.48 (± 0.61)	6.5 (6.35 - 6.8)	6.52 (± 0.17)
Liver	[E] Phenyl isothiocyanate	6.19 (5.84 - 7.11)	6.25 (± 0.39)	6.45 (6.05 - 6.95)	6.41 (± 0.38)	6.15 (5.95 - 6.45)	6.17 (± 0.23)

Target organ	Toxin	24 h post dose		48 h post dose		168 h post dose	
		Median (min - max)	Mean (± SD)	Median (min - max)	Mean (± SD)	Median (min - max)	Mean (± SD)
Liver	[B] Retinyl palmitate	5.72 (5.12 - 6.22)	5.7 (± 0.33)	6.02 (5.42 - 6.32)	5.9 (± 0.37)	5.92 (5.42 - 6.22)	5.88 (± 0.29)
Liver	[B] Sodium Valproate			5.5 (5 - 5.82)	5.44 (± 0.31)	6.4 (6.15 - 6.5)	6.35 (± 0.17)
Liver	[C] a-Naphthylisothiocyanate (ANIT)	4.18 (4.06 - 4.89)	4.28 (± 0.28)	5.56 (4.78 - 6.18)	5.49 (± 0.51)	6.64 (6.22 - 7.28)	6.75 (± 0.44)
Kidney	[D] 2-Bromophenol	5.8 (5.5 - 6.2)	5.83 (± 0.19)	8.5 (6.8 - 8.81)	8.06 (± 0.87)	7.3 (6.4 - 9.2)	7.42 (± 1.09)
Kidney	[E] 3,5-Dichloroaniline hydrochloride	6.06 (5.48 - 6.31)	6.01 (± 0.26)	5.65 (5.45 - 6.15)	5.73 (± 0.28)	6.35 (6.25 - 6.55)	6.35 (± 0.12)
Kidney	[E] Atractyloside	6.82 (6.14 - 8.3)	6.96 (± 0.49)	6.5 (5.6 - 7.4)	6.46 (± 0.77)	6 (5.6 - 6.1)	5.96 (± 0.21)
Kidney	[D] Bromoethylamine hydrobromide	6.1 (5.66 - 6.6)	6.06 (± 0.29)	7.3 (4.8 - 7.5)	6.59 (± 1.18)	6.4 (4.8 - 6.5)	6.04 (± 0.72)
Kidney	[D] Cephaloridine	6.05 (5.4 - 7.1)	6.04 (± 0.5)	6.9 (5.7 - 7.1)	6.66 (± 0.59)	5.8 (5.5 - 7.5)	6.06 (± 0.81)
Kidney	[B] Chlorethanamine	6 (5.7 - 6.4)	6.02 (± 0.21)	5.6 (5.3 - 6)	5.64 (± 0.25)	6.2 (6 - 6.3)	6.18 (± 0.13)
Kidney	[A] Cisplatin	5.35 (4.8 - 6.2)	5.42 (± 0.39)	6.3 (5.7 - 7.6)	6.58 (± 0.73)	7.2 (6.9 - 8.3)	7.42 (± 0.54)
Kidney	[A] D-limonene (chronic)						
Kidney	[E] Dichlorophenyl succinimide	6.26 (5.94 - 6.44)	6.25 (± 0.17)	6.5 (6.2 - 6.9)	6.54 (± 0.25)	6.2 (5.8 - 6.5)	6.16 (± 0.27)
Kidney	[D] Ethylene glycol	6.05 (5.6 - 6.3)	6.02 (± 0.19)	7.4 (6.9 - 8)	7.42 (± 0.44)	7.2 (6.1 - 7.8)	7.02 (± 0.7)
Kidney	[A] Folic acid	7.05 (4.9 - 9.1)	6.79 (± 1.34)	7.12 (5.7 - 8.2)	6.99 (± 0.91)	6.3 (6 - 7.1)	6.42 (± 0.44)
Kidney	[A] Gentamicin	6.05 (5.5 - 6.7)	6.08 (± 0.43)	7.1 (5.6 - 8.27)	6.81 (± 1.08)	6.36 (6.1 - 8.4)	6.87 (± 0.98)
Kidney	[B] Maleic acid	6.92 (6.32 - 7.32)	6.83 (± 0.31)	5.52 (5.22 - 6.52)	5.68 (± 0.5)	5.72 (5.42 - 6.22)	5.76 (± 0.32)
Kidney	[A] N-phenylanthranilic acid (chronic)	5.85 (5.25 - 6.94)	5.95 (± 0.5)	5.23 (5.09 - 7.55)	5.73 (± 1.03)	5.8 (4.59 - 6.52)	5.64 (± 0.71)
Kidney	[D] Para-aminophenol	5.85 (5.45 - 7.65)	5.98 (± 0.63)	7.55 (6.75 - 8.81)	7.74 (± 0.86)	5.85 (5.65 - 7.89)	6.48 (± 0.99)
Kidney	[A] Puromycin			5.5 (4.9 - 6.3)	5.56 (± 0.63)	5.7 (4.4 - 6.3)	5.56 (± 0.71)
Kidney	[B] Vancomycin hydrochloride			6 (5.9 - 6.4)	6.1 (± 0.23)	6.4 (6.2 - 6.5)	6.36 (± 0.11)

Target organ	Toxin	24 h post dose		48 h post dose		168 h post dose	
		Median (min - max)	Mean (± SD)	Median (min - max)	Mean (± SD)	Median (min - max)	Mean (± SD)
Liver & Kidney	[E] Acetaminophen	7.01 (6.14 - 7.99)	6.9 (± 0.64)	6.75 (5.95 - 8.85)	6.99 (± 1.11)	6.15 (5.65 - 6.55)	6.11 (± 0.32)
Liver & Kidney	[B] Aurothiomalate	5.42 (4.82 - 7.22)	5.79 (± 0.86)	5.92 (5.22 - 7.92)	6.38 (± 1.11)	5.52 (5.22 - 5.82)	5.56 (± 0.26)
Liver & Kidney	[C] Chloroform	6.42 (5.82 - 7.78)	6.56 (± 0.56)	6.16 (5.54 - 6.48)	6.02 (± 0.42)	6.66 (6.43 - 7.44)	6.89 (± 0.45)
Liver & Kidney	[D] Cyclosporin	4.75 (4.7 - 6.1)	5 (± 0.5)	6.9 (6.26 - 7.9)	7.01 (± 0.72)	7.48 (6.5 - 8.7)	7.58 (± 0.81)
Liver & Kidney	[D] Dichlorobenzene	5.2 (4.8 - 6)	5.3 (± 0.4)	6.8 (4.7 - 8.2)	6.46 (± 1.35)	7.2 (5 - 8.3)	6.98 (± 1.21)
Liver & Kidney	[C] Ethionine	6.56 (5.07 - 7.05)	6.33 (± 0.62)	5.33 (4.9 - 6.32)	5.42 (± 0.55)	6.04 (5.08 - 6.73)	5.97 (± 0.6)
Liver & Kidney	[B] Hexachlorobutadiene (HCBD)			7.45 (6.45 - 8.65)	7.57 (± 0.93)	6.75 (5.85 - 7.25)	6.69 (± 0.52)
Liver & Kidney	[B] Mercuric chloride	5.8 (5 - 6.6)	5.84 (± 0.46)	6 (5.2 - 6.7)	6.06 (± 0.59)	6 (4.1 - 6.15)	5.66 (± 0.88)
Liver & Kidney	[E] Microcystin-LR			5.8 (5.2 - 6.1)	5.73 (± 0.38)	6.35 (6.3 - 7.4)	6.59 (± 0.47)
Liver & Kidney	[E] Rotenone	7.11 (5.73 - 7.42)	6.78 (± 0.67)	6.36 (5.9 - 7.7)	6.58 (± 0.85)	6.38 (6 - 6.53)	6.34 (± 0.18)
Liver & Kidney	[E] S-(1,2-dichlorovinyl)-cysteine (DCVC)	6.05 (5.44 - 8.01)	6.26 (± 0.75)	6.4 (6.1 - 8)	6.76 (± 0.8)	6.5 (5.9 - 6.6)	6.34 (± 0.29)
Liver & Kidney	[D] Thioacetamide	5.75 (5.4 - 6.3)	5.78 (± 0.3)	7.2 (6 - 8.2)	7.28 (± 0.85)	5.8 (5.1 - 7.6)	6.04 (± 0.94)
Pancreas	[E] 1-Cyano-2-hydroxy-3-butene	6.26 (5.68 - 6.69)	6.25 (± 0.34)	5.9 (5.3 - 6.2)	5.77 (± 0.4)	5.6 (5.5 - 6)	5.68 (± 0.19)
Pancreas	[C] Caerulin	5.92 (5.24 - 6.65)	5.9 (± 0.51)	6.12 (5.26 - 6.66)	6.05 (± 0.59)	6.12 (5.72 - 6.25)	6.05 (± 0.2)
Pancreas	[E] L-arginine	6.38 (5.2 - 16.2)	7.03 (± 2.24)	6.19 (5.8 - 7.4)	6.34 (± 0.56)	6.1 (5.4 - 6.9)	6.04 (± 0.42)
Pancreas	[B] Streptozotocin	5.95 (5 - 6.7)	5.91 (± 0.44)	5.7 (5.4 - 6.3)	5.8 (± 0.33)	5.6 (4.6 - 5.8)	5.4 (± 0.48)
Testicular	[D] 1,3-Dinitrobenzene	5.75 (5.1 - 6.1)	5.72 (± 0.32)	7.4 (6.2 - 7.9)	7.1 (± 0.72)	6.4 (5.7 - 6.8)	6.38 (± 0.43)
Testicular	[C] Cadmium chloride	6.21 (5.38 - 6.89)	6.19 (± 0.48)	6.2 (4.93 - 6.82)	6.08 (± 0.7)	6 (5.86 - 7.22)	6.25 (± 0.55)
Testicular	[D] Cadmium chloride	5.32 (4.95 - 5.57)	5.26 (± 0.21)	6.57 (6.13 - 8.38)	6.92 (± 0.87)	6.31 (5.47 - 8.59)	6.68 (± 1.27)
Testicular	[D] Carbendazim	6 (5.6 - 7)	5.98 (± 0.44)	7.2 (6.2 - 8.1)	7.04 (± 0.76)	7.52 (6.4 - 10.1)	7.92 (± 1.55)

Target organ	Toxin	24 h post dose		48 h post dose		168 h post dose	
		Median (min - max)	Mean (± SD)	Median (min - max)	Mean (± SD)	Median (min - max)	Mean (± SD)
Testicular	[D] Di-n-pentyl-phthalate	5.33 (5.06 - 5.87)	5.35 (± 0.24)	6.73 (6.1 - 8.13)	6.81 (± 0.79)	6.29 (5.95 - 7.76)	6.55 (± 0.73)
Testicular	[D] Ethane dimethane sulfonate (EDS)	5.5 (4.8 - 5.9)	5.38 (± 0.35)	7.6 (6.8 - 8.7)	7.7 (± 0.84)	8.6 (6.3 - 8.9)	7.98 (± 1.14)
Testicular	[D] Methoxyacetic acid	6.06 (5.54 - 6.15)	5.95 (± 0.21)	6.88 (6.61 - 8.86)	7.36 (± 0.93)	7.36 (6.86 - 9.25)	7.69 (± 1.01)
Multiple organ	[B] Adriamycin	6.15 (5.3 - 6.7)	6.16 (± 0.39)	5.6 (5.5 - 6.2)	5.74 (± 0.3)	5.8 (5.7 - 6.4)	5.92 (± 0.29)
Multiple organ	[C] Amphotericin B	6.07 (5.54 - 7.31)	6.24 (± 0.56)	6.24 (5.91 - 7.02)	6.36 (± 0.45)	6.59 (6.3 - 7)	6.64 (± 0.26)
Multiple organ	[C] Azaserine	6.16 (4.97 - 6.97)	6.1 (± 0.6)	5.83 (5.37 - 5.91)	5.69 (± 0.25)	6.12 (5.16 - 6.46)	5.83 (± 0.58)
Multiple organ	[A] Dexamethasone	4.4 (4.2 - 4.9)	4.44 (± 0.23)	8.38 (7.5 - 9.2)	8.31 (± 0.66)	7.8 (6.32 - 8.7)	7.81 (± 0.93)
Multiple organ	[E] Mitomycin-C	6.44 (5.94 - 6.73)	6.41 (± 0.24)	6.1 (5.9 - 6.4)	6.16 (± 0.19)	5.8 (5.5 - 6.4)	5.9 (± 0.35)
Physiological stressor	[C] 1,1-Dichloroethylene & maleic acid	6.15 (5.19 - 7.05)	6.18 (± 0.6)	5.92 (5.53 - 6.5)	5.94 (± 0.36)	6.25 (5.92 - 7.36)	6.49 (± 0.59)
Physiological stressor	[C] 2,4-Dinitrophenol	6.13 (5.18 - 6.96)	6.04 (± 0.51)	5.62 (5.15 - 6.57)	5.76 (± 0.55)	7.19 (7.09 - 7.5)	7.26 (± 0.16)
Physiological stressor	[B] 4-Pentenoic acid	6.3 (5.85 - 6.65)	6.32 (± 0.28)	5.85 (5.75 - 6.15)	5.88 (± 0.15)	6.05 (5.95 - 6.45)	6.17 (± 0.22)
Physiological stressor	[D] Acetazolamide	5.7 (5.15 - 6.25)	5.68 (± 0.33)	6.25 (5.45 - 8.75)	6.55 (± 1.27)	6.75 (5.85 - 7.45)	6.75 (± 0.59)
Physiological stressor	[C] Acivicin	6.33 (5.36 - 7.14)	6.3 (± 0.48)	5.76 (5.64 - 6.86)	6 (± 0.5)	6.15 (5.82 - 6.66)	6.18 (± 0.34)
Physiological stressor	[E] Ammonium chloride			5.9 (5.5 - 6)	5.82 (± 0.22)	6.3 (5.7 - 6.4)	6.2 (± 0.28)
Physiological stressor	[D] Carboplatin	5.4 (4.7 - 6.2)	5.45 (± 0.48)	7.5 (6.7 - 9.3)	7.62 (± 1.01)	6.7 (5.7 - 9.7)	7.14 (± 1.51)
Physiological stressor	[A] Choline and choline/methionine deficiency (chronic)	6.45 (5.1 - 7.9)	6.58 (± 0.8)	5.9 (5.8 - 6.1)	5.94 (± 0.15)	5.7 (5.6 - 5.9)	5.76 (± 0.13)
Physiological stressor	[B] Food restriction (chronic)	5.6 (5.5 - 5.9)	5.62 (± 0.14)	5.7 (5.6 - 6.3)	5.82 (± 0.32)	5.5 (4.8 - 5.9)	5.44 (± 0.4)
Physiological stressor	[D] Furosemide	6.35 (5.95 - 7.15)	6.46 (± 0.39)	6.65 (6.55 - 7.55)	6.97 (± 0.53)	6.95 (5.85 - 8.55)	6.87 (± 1.07)
Physiological stressor	[B] Insulin	6.9 (6.4 - 7.3)	6.86 (± 0.33)	5.5 (5.2 - 5.9)	5.52 (± 0.29)	5.6 (5.4 - 6.1)	5.66 (± 0.27)
Physiological stressor	[E] Methotrexate	6.4 (6.05 - 6.58)	6.34 (± 0.18)	6.2 (5.9 - 6.6)	6.26 (± 0.29)	6.2 (6 - 6.5)	6.2 (± 0.19)
Physiological stressor	[A] Partial hepatectomy	6.22 (5.84 - 6.54)	6.19 (± 0.22)	6.04 (5.84 - 8.09)	6.43 (± 0.94)	5.91 (5.68 - 6.65)	6 (± 0.38)

Target organ	Toxin	24 h post dose		48 h post dose		168 h post dose	
		Median (min - max)	Mean (± SD)	Median (min - max)	Mean (± SD)	Median (min - max)	Mean (± SD)
Physiological stressor	[A] Phenobarbital (chronic)	5.9 (5.6 - 6.6)	6.01 (± 0.33)	6.2 (5.8 - 7.3)	6.3 (± 0.6)	6.5 (5.8 - 7.5)	6.62 (± 0.61)
Physiological stressor	[A] Pregnenolone 16 alpha carbonitrile (chronic)						
Physiological stressor	[A] Probenecid	5.5 (4.81 - 6.15)	5.53 (± 0.46)	4.81 (3.93 - 7.92)	5.22 (± 1.58)	5.9 (5.34 - 6.83)	6.08 (± 0.57)
Physiological stressor	[C] Rosiglitazone	5.89 (5.26 - 6.56)	5.91 (± 0.35)	6.07 (5.44 - 6.68)	6.01 (± 0.47)	5.82 (5.73 - 6.32)	5.95 (± 0.27)
Physiological stressor	[C] Rosiglitazone (chronic)	5.88 (5.48 - 6.53)	5.9 (± 0.31)				
Physiological stressor	[E] Sodium bicarbonate	6.13 (5.84 - 6.39)	6.09 (± 0.18)				
Physiological stressor	[A] Unilateral nephrectomy	5.94 (5.46 - 6.48)	5.92 (± 0.32)	5.02 (4.62 - 5.4)	5.04 (± 0.29)	5.42 (4.72 - 6.26)	5.47 (± 0.57)
Physiological stressor	[B] Water deprivation (chronic)	5.95 (5.5 - 6.3)	5.89 (± 0.26)	5.7 (5.4 - 6.16)	5.73 (± 0.25)	6.38 (5.9 - 6.6)	6.33 (± 0.25)
No Effect	[E] Acetaminophen (chronic)	6.3 (6 - 6.7)	6.32 (± 0.26)			6.3 (5.9 - 6.7)	6.3 (± 0.29)
No Effect	[C] Buthionine sulphoxime	6.33 (5.58 - 6.94)	6.27 (± 0.42)	5.34 (5.12 - 5.92)	5.44 (± 0.31)	5.53 (5.42 - 6.86)	5.78 (± 0.61)
No Effect	[C] Ferrous sulphate	6.08 (5.7 - 7.36)	6.26 (± 0.58)	5.94 (5.26 - 6.14)	5.78 (± 0.36)	5.83 (5.46 - 6.32)	5.92 (± 0.37)
No Effect	[B] Ifosfamide	5.8 (5.5 - 6.2)	5.85 (± 0.26)	5.9 (5.8 - 6.3)	6.02 (± 0.26)	6.2 (5.8 - 6.7)	6.24 (± 0.34)
No Effect	[B] Lithocholic acid	6.15 (6 - 6.8)	6.31 (± 0.31)	5.9 (5.5 - 6.2)	5.86 (± 0.3)	6.1 (5.7 - 6.5)	6.1 (± 0.35)
No Effect	[E] Paraquat			6.1 (5.3 - 6.3)	5.96 (± 0.42)	5.6 (5.4 - 6.2)	5.78 (± 0.39)
No Effect	[D] Potassium dichromate	5.55 (4.6 - 7.6)	5.7 (± 0.83)	6 (5.6 - 8.2)	6.64 (± 1.11)	6.3 (5.8 - 7.4)	6.46 (± 0.6)
No Effect	[C] Trichlorethylene	6.13 (5.36 - 6.55)	6.01 (± 0.4)	6.54 (6.13 - 7.29)	6.61 (± 0.42)	7.03 (6.26 - 7.17)	6.9 (± 0.38)

A-F: Indicates Pharmaceutical Company & sample origin

Supplementary Table S23. Summary statistics for serum phosphate (mmol/L) at 24 hrs, 48 hrs and 168 hrs post high dose.

Target organ	Toxin	24 h post dose		48 h post dose		168 h post dose	
		Median (min - max)	Mean (± SD)	Median (min - max)	Mean (± SD)	Median (min - max)	Mean (± SD)
Liver	[E] 1,1-Dichloroethylene	3.06 (2.88 - 3.46)	3.09 (± 0.17)	3.2 (2.98 - 3.27)	3.17 (± 0.11)	2.81 (2.49 - 3.11)	2.8 (± 0.22)
Liver	[E] 1,2,3,4,5,6-hexachlorocyclohexane	3.25 (2.91 - 3.43)	3.23 (± 0.17)	3.17 (2.94 - 3.23)	3.12 (± 0.12)	3.14 (2.72 - 3.46)	3.09 (± 0.28)
Liver	[B] 1-Fluoropentane	2.75 (2.38 - 3.38)	2.83 (± 0.29)	2.99 (2.9 - 3.06)	2.99 (± 0.07)	3.12 (2.86 - 3.15)	3.05 (± 0.13)
Liver	[B] 2,4,6-Trihydroxyacetophenone (THA)	3.12 (2.94 - 3.69)	3.19 (± 0.23)	2.85 (2.69 - 3.04)	2.83 (± 0.13)	3.11 (2.52 - 3.14)	2.93 (± 0.27)
Liver	[B] 4-Amino-2,6-dichlorophenol (ADCP)	3.5 (3.04 - 3.66)	3.44 (± 0.21)	2.91 (2.79 - 2.98)	2.9 (± 0.07)	2.82 (2.66 - 3.24)	2.87 (± 0.22)
Liver	[C] Aflatoxin	3.58 (3.03 - 3.97)	3.53 (± 0.32)	3.69 (3.29 - 3.99)	3.64 (± 0.25)	3.43 (2.95 - 4.08)	3.57 (± 0.47)
Liver	[C] Allyl alcohol	2.99 (2.54 - 4.02)	3.14 (± 0.54)	2.28 (1.66 - 2.58)	2.18 (± 0.35)	3.37 (3.01 - 3.41)	3.29 (± 0.17)
Liver	[C] Allyl formate	2.93 (2.73 - 3.35)	2.96 (± 0.2)	2.86 (2.63 - 3.23)	2.88 (± 0.23)	3.01 (2.81 - 3.62)	3.1 (± 0.31)
Liver	[B] Azathioprine	3.3 (3.21 - 3.66)	3.37 (± 0.16)	3.01 (2.72 - 3.14)	2.97 (± 0.16)	2.95 (2.37 - 2.98)	2.8 (± 0.26)
Liver	[B] Bromobenzene	3.72 (3.3 - 4.07)	3.71 (± 0.22)	3.49 (3.33 - 3.78)	3.53 (± 0.17)	3.04 (2.62 - 3.46)	3.03 (± 0.3)
Liver	[C] Butylated hydroxytoluene	3.18 (2.68 - 3.53)	3.17 (± 0.26)	2.72 (2.48 - 3.06)	2.71 (± 0.24)	3.1 (2.81 - 3.37)	3.13 (± 0.22)
Liver	[D] Carbon tetrachloride	2.65 (2.35 - 2.87)	2.66 (± 0.14)	3.71 (3.11 - 4.31)	3.77 (± 0.5)	3.21 (2.51 - 3.31)	3.05 (± 0.32)
Liver	[C] Chlorpromazine	3.11 (2.84 - 4.35)	3.27 (± 0.44)	3.32 (3 - 3.37)	3.23 (± 0.16)	2.65 (2.51 - 3)	2.68 (± 0.19)
Liver	[B] Clofibrate	3.61 (3.35 - 4.32)	3.69 (± 0.3)	3.28 (2.8 - 3.48)	3.21 (± 0.28)	3.22 (2.7 - 3.35)	3.08 (± 0.26)
Liver	[B] Cyproterone acetate	3.43 (3.36 - 3.65)	3.46 (± 0.1)	2.88 (2.69 - 3.43)	2.94 (± 0.3)	2.81 (2.75 - 3.01)	2.85 (± 0.1)
Liver	[A] D-galactosamine	2.88 (1.89 - 3)	2.77 (± 0.32)	3.83 (2.65 - 4.25)	3.7 (± 0.62)	3.54 (3.12 - 3.86)	3.46 (± 0.33)
Liver	[B] Diethylhexylphthalate (DEHP)			3.67 (3.28 - 3.86)	3.61 (± 0.23)	3.28 (2.96 - 3.35)	3.18 (± 0.17)
Liver	[C] Dimethylformamide (DMF)	2.91 (2.27 - 3.22)	2.9 (± 0.27)	2.81 (2.48 - 2.97)	2.73 (± 0.2)	2.89 (2.6 - 3.07)	2.87 (± 0.17)
Liver	[C] Dimethylnitrosamine (DMN)	3.09 (2.5 - 3.48)	3.03 (± 0.27)	3.17 (2.61 - 3.63)	3.09 (± 0.39)	2.76 (2.51 - 2.88)	2.7 (± 0.15)
Liver	[A] Gadolinium chloride	3.22 (2.87 - 3.57)	3.21 (± 0.22)	3.69 (3.39 - 3.85)	3.67 (± 0.17)	3.59 (3.36 - 3.91)	3.6 (± 0.22)

Target organ	Toxin	24 h post dose		48 h post dose		168 h post dose	
		Median (min - max)	Mean (± SD)	Median (min - max)	Mean (± SD)	Median (min - max)	Mean (± SD)
Liver	[A, B, C, D, F] Hydrazine	3.02 (2.51 - 3.74)	3.06 (± 0.28)	3.38 (2.86 - 4.74)	3.57 (± 0.44)	2.6 (1.09 - 3.57)	2.6 (± 0.73)
Liver	[E] Hydrazine	3.13 (2.99 - 3.25)	3.12 (± 0.07)	3.04 (2.98 - 3.18)	3.06 (± 0.09)	2.93 (2.72 - 3.4)	3.01 (± 0.28)
Liver	[E] Indomethacin	3.62 (3.06 - 4.09)	3.63 (± 0.37)	2.86 (2.51 - 3.38)	2.89 (± 0.31)	3.09 (2.48 - 5.19)	3.43 (± 1.03)
Liver	[E] Ketoconazole			2.98 (2.78 - 3.33)	3 (± 0.2)	2.98 (2.59 - 3.49)	3.06 (± 0.36)
Liver	[C] Lead acetate	3.3 (2.7 - 4.46)	3.35 (± 0.5)	2.79 (2.14 - 2.95)	2.68 (± 0.31)	3.29 (3.02 - 3.43)	3.24 (± 0.2)
Liver	[A] Lipopolysaccharide (LPS)	2.56 (2.2 - 2.98)	2.55 (± 0.21)	3.33 (3.23 - 3.51)	3.34 (± 0.12)	3.49 (3.17 - 3.65)	3.42 (± 0.22)
Liver	[B] Methapyrilene	3.54 (3.33 - 3.75)	3.57 (± 0.12)	3.2 (2.75 - 3.53)	3.16 (± 0.28)	2.81 (2.69 - 3.11)	2.87 (± 0.17)
Liver	[E] Methylene dianiline	3.11 (2.9 - 3.54)	3.15 (± 0.2)	2.9 (2.7 - 3.48)	2.96 (± 0.31)	3.02 (2.48 - 3.15)	2.89 (± 0.29)
Liver	[C] Monocrotaline	3.48 (2.81 - 4.26)	3.51 (± 0.51)	2.84 (2.31 - 3.27)	2.79 (± 0.36)	2.61 (2.48 - 3.2)	2.8 (± 0.36)
Liver	[C] N-methylformamide (NMF)	2.98 (2.75 - 3.38)	3.02 (± 0.22)	2.69 (2.59 - 3.12)	2.76 (± 0.21)	2.9 (2.87 - 3.2)	2.99 (± 0.16)
Liver	[D] Phalloidin (chronic)			3.22 (2.83 - 3.34)	3.15 (± 0.2)	3.79 (2.57 - 4.39)	3.61 (± 0.67)
Liver	[E] Phenyl diisothiocyanate	3.03 (2.77 - 3.42)	3.01 (± 0.2)	3.98 (3.36 - 5.98)	4.17 (± 1.05)	3.22 (2.65 - 3.33)	3.08 (± 0.28)
Liver	[E] Phenyl isothiocyanate	2.98 (2.59 - 3.62)	3.05 (± 0.36)	3.11 (2.94 - 3.3)	3.1 (± 0.15)	3.33 (3.14 - 3.62)	3.34 (± 0.19)
Liver	[B] Retinyl palmitate	3.67 (2.95 - 4.24)	3.63 (± 0.37)	3.14 (2.95 - 3.43)	3.18 (± 0.2)	3.27 (3.14 - 3.56)	3.31 (± 0.17)
Liver	[B] Sodium Valproate			3.79 (2.99 - 4.21)	3.7 (± 0.44)	3.17 (3.01 - 3.21)	3.15 (± 0.08)
Liver	[C] a-Naphthylisothiocyanate (ANIT)	3.52 (3.15 - 4.28)	3.57 (± 0.35)	3.23 (2.74 - 3.56)	3.13 (± 0.34)	3.06 (2.71 - 3.36)	3.05 (± 0.23)
Kidney	[D] 2-Bromophenol	2.66 (2.46 - 2.96)	2.68 (± 0.14)	4.46 (3.86 - 5.36)	4.5 (± 0.59)	3.66 (3.66 - 4.36)	3.8 (± 0.31)
Kidney	[E] 3,5-Dichloroaniline hydrochloride	3.01 (2.62 - 3.46)	3.06 (± 0.26)	2.59 (2.43 - 2.69)	2.56 (± 0.13)	2.88 (2.72 - 3.23)	2.91 (± 0.22)
Kidney	[E] Atractyloside	3.33 (2.88 - 3.46)	3.22 (± 0.19)	2.85 (2.65 - 3.65)	3.03 (± 0.43)	3.04 (2.62 - 3.3)	2.98 (± 0.32)
Kidney	[D] Bromoethylamine hydrobromide	2.71 (2.51 - 2.81)	2.69 (± 0.11)	3.61 (2.91 - 3.81)	3.42 (± 0.4)	4.51 (3.48 - 4.71)	4.24 (± 0.5)

Target organ	Toxin	24 h post dose		48 h post dose		168 h post dose	
		Median (min - max)	Mean (± SD)	Median (min - max)	Mean (± SD)	Median (min - max)	Mean (± SD)
Kidney	[D] Cephaloridine	2.96 (2.71 - 3.51)	3.02 (± 0.22)	4.71 (4.31 - 4.81)	4.63 (± 0.22)	3.81 (3.41 - 3.91)	3.73 (± 0.22)
Kidney	[B] Chlorethanamine	3.2 (2.88 - 3.3)	3.18 (± 0.13)	2.91 (2.75 - 3.07)	2.92 (± 0.15)	3.01 (2.52 - 3.2)	2.9 (± 0.32)
Kidney	[A] Cisplatin	2.36 (2.11 - 2.96)	2.43 (± 0.23)	3.11 (2.81 - 4.31)	3.33 (± 0.61)	3.41 (3.21 - 3.51)	3.38 (± 0.13)
Kidney	[A] D-limonene (chronic)						
Kidney	[E] Dichlorophenyl succinimide	3.15 (2.61 - 3.28)	3.07 (± 0.22)	2.99 (2.67 - 3.38)	3.04 (± 0.27)	3.02 (2.93 - 3.12)	3.03 (± 0.08)
Kidney	[D] Ethylene glycol	2.56 (2.31 - 2.71)	2.52 (± 0.14)	3.41 (3.21 - 3.71)	3.43 (± 0.23)	3.31 (3.01 - 3.51)	3.29 (± 0.19)
Kidney	[A] Folic acid	4.76 (2.26 - 6.46)	4.24 (± 1.39)	6.46 (3.48 - 8.76)	6.56 (± 2.01)	3.36 (2.76 - 3.51)	3.26 (± 0.32)
Kidney	[A] Gentamicin	3.11 (2.67 - 3.41)	3.08 (± 0.21)	3.91 (3.71 - 5.21)	4.11 (± 0.63)	3.31 (3.11 - 3.61)	3.39 (± 0.22)
Kidney	[B] Maleic acid	2.91 (2.14 - 3.33)	2.78 (± 0.46)	2.79 (2.4 - 3.79)	2.88 (± 0.54)	2.62 (2.49 - 2.62)	2.57 (± 0.07)
Kidney	[A] N-phenylanthranilic acid (chronic)	1.97 (1.62 - 2.74)	2.1 (± 0.43)	3.59 (3.2 - 4.37)	3.66 (± 0.45)	3.49 (3.01 - 3.59)	3.43 (± 0.24)
Kidney	[D] Para-aminophenol	2.26 (1.91 - 4.01)	2.48 (± 0.61)	3.81 (3.31 - 6.51)	4.31 (± 1.34)	3.51 (1.91 - 4.31)	3.35 (± 0.89)
Kidney	[A] Puromycin			3.06 (2.76 - 3.36)	3.02 (± 0.26)	2.86 (2.66 - 3.36)	3.02 (± 0.32)
Kidney	[B] Vancomycin hydrochloride			3.14 (3.04 - 3.23)	3.14 (± 0.09)	3.07 (3.04 - 3.17)	3.09 (± 0.06)
Liver & Kidney	[E] Acetaminophen	3.06 (2.85 - 4.69)	3.23 (± 0.54)	2.81 (2.49 - 5.07)	3.23 (± 1.07)	2.85 (2.49 - 3.11)	2.85 (± 0.23)
Liver & Kidney	[B] Aurothiomalate	3.98 (3.21 - 4.98)	4.12 (± 0.59)	4.37 (2.91 - 5.14)	4.24 (± 0.82)	2.49 (2.04 - 3.04)	2.57 (± 0.44)
Liver & Kidney	[C] Chloroform	3.1 (2.74 - 3.64)	3.18 (± 0.31)	2.9 (2.41 - 3.31)	2.84 (± 0.38)	2.89 (2.86 - 3.16)	2.96 (± 0.12)
Liver & Kidney	[D] Cyclosporin	2.86 (2.71 - 3.11)	2.87 (± 0.13)	3.81 (3.3 - 4.31)	3.77 (± 0.41)	3.41 (3.11 - 3.83)	3.51 (± 0.31)
Liver & Kidney	[D] Dichlorobenzene	2.81 (2.51 - 3.11)	2.84 (± 0.16)	3.11 (3.01 - 3.81)	3.35 (± 0.38)	4.21 (3.81 - 4.71)	4.23 (± 0.35)
Liver & Kidney	[C] Ethionine	3.49 (2.79 - 3.84)	3.43 (± 0.31)	3.2 (3.08 - 3.48)	3.22 (± 0.16)	2.87 (2.63 - 3.01)	2.83 (± 0.15)
Liver & Kidney	[B] Hexachlorobutadiene (HCBD)			3.19 (2.54 - 3.35)	3.05 (± 0.32)	2.57 (1.83 - 3.64)	2.63 (± 0.65)

Target organ	Toxin	24 h post dose		48 h post dose		168 h post dose	
		Median (min - max)	Mean (± SD)	Median (min - max)	Mean (± SD)	Median (min - max)	Mean (± SD)
Liver & Kidney	[B] Mercuric chloride	3.5 (3.01 - 4.24)	3.53 (± 0.4)	4.17 (2.91 - 4.62)	3.92 (± 0.73)	3.21 (2.11 - 4.04)	3.09 (± 0.72)
Liver & Kidney	[E] Microcystin-LR			2.7 (2.09 - 2.87)	2.54 (± 0.33)	2.93 (2.8 - 3.44)	3.06 (± 0.26)
Liver & Kidney	[E] Rotenone	3.56 (2.88 - 4.82)	3.62 (± 0.65)	3.43 (2.84 - 5.2)	3.72 (± 1.04)	3.3 (3.11 - 3.98)	3.42 (± 0.33)
Liver & Kidney	[E] S-(1,2-dichlorovinyl)-cysteine (DCVC)	3.15 (2.51 - 5.25)	3.28 (± 0.78)	2.86 (2.54 - 3.19)	2.88 (± 0.26)	3.22 (2.96 - 3.32)	3.14 (± 0.16)
Liver & Kidney	[D] Thioacetamide	2.61 (2.26 - 2.95)	2.57 (± 0.28)	3.17 (2.96 - 3.29)	3.13 (± 0.16)	2.96 (2.66 - 4.36)	3.24 (± 0.68)
Pancreas	[E] 1-Cyano-2-hydroxy-3-butene	3.3 (2.51 - 3.59)	3.2 (± 0.33)	3.11 (2.87 - 3.75)	3.17 (± 0.34)	2.52 (2.33 - 2.94)	2.64 (± 0.26)
Pancreas	[C] Caerulin	3.2 (2.81 - 3.81)	3.15 (± 0.3)	2.69 (2.6 - 3.33)	2.81 (± 0.3)	3.01 (2.47 - 3.3)	2.94 (± 0.32)
Pancreas	[E] L-arginine	3.33 (0.14 - 5.59)	2.95 (± 1.43)	3.01 (2.49 - 3.85)	3.1 (± 0.45)	2.46 (1.98 - 2.91)	2.49 (± 0.25)
Pancreas	[B] Streptozotocin	2.95 (2.63 - 3.47)	2.96 (± 0.29)	2.79 (2.59 - 2.98)	2.8 (± 0.16)	3.08 (2.79 - 3.4)	3.07 (± 0.22)
Testicular	[D] 1,3-Dinitrobenzene	2.91 (2.51 - 3.31)	2.92 (± 0.26)	4.41 (3.71 - 5.01)	4.37 (± 0.49)	4.01 (3.81 - 4.41)	4.11 (± 0.24)
Testicular	[C] Cadmium chloride	2.75 (2.17 - 3.16)	2.67 (± 0.32)	2.44 (1.86 - 3.18)	2.45 (± 0.52)	2.88 (2.58 - 3.26)	2.91 (± 0.25)
Testicular	[D] Cadmium chloride	2.56 (2.22 - 2.78)	2.52 (± 0.2)	3.31 (2.88 - 3.62)	3.25 (± 0.3)	3.42 (2.68 - 3.89)	3.3 (± 0.53)
Testicular	[D] Carbendazim	2.61 (2.41 - 3.01)	2.67 (± 0.2)	3.41 (3.11 - 3.51)	3.39 (± 0.16)	3.51 (3.01 - 4.21)	3.55 (± 0.45)
Testicular	[D] Di-n-pentyl-phthalate	2.83 (2.42 - 3.32)	2.83 (± 0.28)	3.43 (3.1 - 3.65)	3.38 (± 0.21)	3.61 (3.51 - 3.73)	3.61 (± 0.11)
Testicular	[D] Ethane dimethane sulfonate (EDS)	2.51 (2.11 - 2.71)	2.47 (± 0.21)	3.91 (3.81 - 4.21)	3.93 (± 0.16)	3.91 (3.31 - 4.11)	3.81 (± 0.32)
Testicular	[D] Methoxyacetic acid	3.22 (2.91 - 3.47)	3.22 (± 0.15)	4.21 (3.65 - 4.5)	4.05 (± 0.38)	3.75 (3.64 - 4.14)	3.8 (± 0.21)
Multiple organ	[B] Adriamycin	3.77 (3.4 - 4.08)	3.76 (± 0.19)	3.4 (3.17 - 3.49)	3.35 (± 0.13)	2.85 (2.65 - 3.04)	2.83 (± 0.14)
Multiple organ	[C] Amphotericin B	3.65 (3.04 - 4.28)	3.63 (± 0.35)	3.23 (2.19 - 3.36)	3.01 (± 0.49)	2.97 (2.85 - 3.1)	2.97 (± 0.1)
Multiple organ	[C] Azaserine	3.09 (2.71 - 4.1)	3.16 (± 0.41)	2.21 (1.94 - 2.71)	2.26 (± 0.28)	2.78 (1.74 - 3.21)	2.63 (± 0.62)
Multiple organ	[A] Dexamethasone	1.26 (0.86 - 1.56)	1.21 (± 0.24)	4.16 (3.36 - 6.06)	4.28 (± 1.11)	3.76 (3.42 - 4.16)	3.77 (± 0.3)
Multiple organ	[E] Mitomycin-C	3.43 (3.04 - 3.69)	3.38 (± 0.23)	3.07 (2.94 - 3.14)	3.06 (± 0.08)	2.91 (2.78 - 3.11)	2.92 (± 0.12)

Target organ	Toxin	24 h post dose		48 h post dose		168 h post dose	
		Median (min - max)	Mean (± SD)	Median (min - max)	Mean (± SD)	Median (min - max)	Mean (± SD)
Physiological stressor	[C] 1,1-Dichloroethylene & maleic acid	3.12 (2.67 - 3.84)	3.17 (± 0.33)	3.05 (2.72 - 3.27)	3.02 (± 0.2)	2.89 (2.7 - 3.13)	2.89 (± 0.15)
Physiological stressor	[C] 2,4-Dinitrophenol	3 (2.56 - 3.47)	3.05 (± 0.27)	2.9 (2.73 - 3.15)	2.94 (± 0.2)	3.28 (2.91 - 3.35)	3.17 (± 0.22)
Physiological stressor	[B] 4-Pentenoic acid	3.01 (0.43 - 3.27)	2.8 (± 0.85)	2.85 (2.78 - 2.99)	2.88 (± 0.08)	3.23 (3.11 - 3.33)	3.22 (± 0.09)
Physiological stressor	[D] Acetazolamide	2.71 (2.61 - 2.98)	2.73 (± 0.14)	3.61 (3.3 - 5.61)	3.92 (± 0.95)	3.81 (3.51 - 4.11)	3.79 (± 0.28)
Physiological stressor	[C] Acivicin	3.48 (2.37 - 3.87)	3.37 (± 0.46)	2.91 (2.27 - 2.95)	2.7 (± 0.32)	2.79 (2.54 - 3.07)	2.79 (± 0.19)
Physiological stressor	[E] Ammonium chloride			3.41 (3.25 - 3.77)	3.46 (± 0.19)	3.22 (2.99 - 3.35)	3.17 (± 0.16)
Physiological stressor	[D] Carboplatin	2.66 (2.26 - 2.86)	2.61 (± 0.2)	3.66 (3.26 - 4.76)	3.98 (± 0.68)	3.66 (3.46 - 3.96)	3.68 (± 0.19)
Physiological stressor	[A] Choline and choline/methionine deficiency (chronic)	2.91 (2.44 - 3.18)	2.85 (± 0.23)	3.21 (3.01 - 3.51)	3.27 (± 0.19)	2.99 (2.63 - 3.38)	3.01 (± 0.28)
Physiological stressor	[B] Food restriction (chronic)	3.46 (3.23 - 3.75)	3.53 (± 0.18)	3.27 (2.72 - 3.49)	3.19 (± 0.34)	2.88 (2.85 - 3.04)	2.92 (± 0.08)
Physiological stressor	[D] Furosemide	2.51 (2.36 - 3.35)	2.58 (± 0.31)	3.56 (3.46 - 3.96)	3.68 (± 0.22)	3.56 (3.36 - 3.96)	3.68 (± 0.27)
Physiological stressor	[B] Insulin	3.51 (3.31 - 3.8)	3.5 (± 0.15)	2.99 (2.76 - 3.31)	3.05 (± 0.21)	2.83 (2.5 - 2.96)	2.8 (± 0.19)
Physiological stressor	[E] Methotrexate	3.44 (3.28 - 3.73)	3.48 (± 0.15)	2.73 (2.38 - 2.9)	2.7 (± 0.21)	2.93 (2.57 - 3.38)	2.95 (± 0.3)
Physiological stressor	[A] Partial hepatectomy	2.99 (2.36 - 3.43)	2.91 (± 0.34)	3.3 (2.52 - 3.59)	3.13 (± 0.44)	2.94 (2.49 - 3.3)	2.97 (± 0.32)
Physiological stressor	[A] Phenobarbital (chronic)	2.71 (2.51 - 3.21)	2.77 (± 0.23)	3.81 (3.71 - 4.41)	3.97 (± 0.32)	3.91 (3.31 - 4.41)	3.83 (± 0.41)
Physiological stressor	[A] Pregnenolone 16 alpha carbonitrile (chronic)						
Physiological stressor	[A] Probenecid	2.88 (1.88 - 3.2)	2.76 (± 0.4)	3.52 (3.32 - 4.22)	3.66 (± 0.34)	3.43 (3.01 - 3.78)	3.36 (± 0.35)
Physiological stressor	[C] Rosiglitazone	3.09 (2.85 - 3.56)	3.1 (± 0.21)	2.71 (2.51 - 2.86)	2.71 (± 0.14)	2.48 (2.14 - 2.89)	2.51 (± 0.28)
Physiological stressor	[C] Rosiglitazone (chronic)	2.51 (2.21 - 2.73)	2.47 (± 0.17)				
Physiological stressor	[E] Sodium bicarbonate	3.13 (2.88 - 3.21)	3.1 (± 0.11)				
Physiological stressor	[A] Unilateral nephrectomy	2.78 (2.65 - 3.11)	2.83 (± 0.13)	2.91 (2.88 - 3.07)	2.97 (± 0.1)	3.01 (2.85 - 3.2)	3.01 (± 0.13)

Target organ	Toxin	24 h post dose		48 h post dose		168 h post dose	
		Median (min - max)	Mean (± SD)	Median (min - max)	Mean (± SD)	Median (min - max)	Mean (± SD)
Physiological stressor	[B] Water deprivation (chronic)	3.22 (2.96 - 3.49)	3.26 (± 0.17)	2.96 (2.88 - 3.3)	3.03 (± 0.17)	3.09 (2.78 - 3.46)	3.11 (± 0.23)
No Effect	[E] Acetaminophen (chronic)	3.19 (3.02 - 3.22)	3.14 (± 0.08)			2.9 (2.73 - 3.44)	3.02 (± 0.31)
No Effect	[C] Buthionine sulphoxime	2.92 (2.42 - 3.31)	2.92 (± 0.3)	2.41 (2.14 - 2.67)	2.4 (± 0.22)	2.58 (2.3 - 2.64)	2.52 (± 0.13)
No Effect	[C] Ferrous sulphate	1.9 (1.41 - 2.31)	1.85 (± 0.3)	2.59 (2.25 - 2.89)	2.61 (± 0.26)	3.21 (2.9 - 3.49)	3.19 (± 0.21)
No Effect	[B] Ifosfamide	3.53 (3.27 - 3.72)	3.49 (± 0.15)	2.75 (2.52 - 2.85)	2.71 (± 0.14)	3.04 (2.88 - 3.23)	3.05 (± 0.15)
No Effect	[B] Lithocholic acid	3.32 (3.11 - 3.65)	3.33 (± 0.18)	3.07 (2.91 - 3.3)	3.08 (± 0.16)	3.07 (2.98 - 3.17)	3.07 (± 0.07)
No Effect	[E] Paraquat			3.15 (2.99 - 3.44)	3.19 (± 0.2)	3.32 (3.09 - 3.57)	3.32 (± 0.18)
No Effect	[D] Potassium dichromate	2.36 (2.11 - 2.71)	2.34 (± 0.21)	3.21 (3.11 - 3.61)	3.33 (± 0.26)	3.51 (2.91 - 3.71)	3.37 (± 0.34)
No Effect	[C] Trichlorethylene	3.04 (2.69 - 4.27)	3.14 (± 0.45)	2.87 (2.43 - 3.25)	2.84 (± 0.32)	3.03 (2.86 - 3.19)	3 (± 0.13)

A-F: Indicates Pharmaceutical Company & sample origin

Supplementary Table S24. Summary statistics for serum glucose (mmol/L) at 24 hrs, 48 hrs and 168 hrs post high dose.

Target organ	Toxin	24 h post dose		48 h post dose		168 h post dose	
		Median (min - max)	Mean (± SD)	Median (min - max)	Mean (± SD)	Median (min - max)	Mean (± SD)
Liver	[E] 1,1-Dichloroethylene	6.83 (6.05 - 7.44)	6.73 (± 0.45)	10.99 (9.21 - 11.16)	10.59 (± 0.82)	13.38 (11.99 - 14.82)	13.46 (± 1.16)
Liver	[E] 1,2,3,4,5,6-hexachlorocyclohexane	8.24 (7.33 - 9.88)	8.4 (± 0.89)	14.27 (10.1 - 17.54)	13.93 (± 2.79)	13.88 (11.99 - 17.04)	13.92 (± 2.04)
Liver	[B] 1-Fluoropentane	9.53 (9.03 - 10.48)	9.58 (± 0.49)	10.67 (10.4 - 12.4)	10.99 (± 0.81)	9.92 (8.83 - 13.39)	10.34 (± 1.88)
Liver	[B] 2,4,6-Trihydroxyacetophenone (THA)	8.65 (6.99 - 11.23)	8.84 (± 1.37)	10.05 (8.96 - 11.15)	9.9 (± 0.9)	11.74 (10.59 - 14.72)	12.12 (± 1.69)
Liver	[B] 4-Amino-2,6-dichlorophenol (ADCP)	8.6 (7 - 11.14)	8.75 (± 1.17)	9.01 (8.63 - 11.28)	9.36 (± 1.09)	11.57 (9.73 - 12.38)	11.49 (± 1.08)
Liver	[C] Aflatoxin	6.35 (5.1 - 6.9)	6.26 (± 0.56)	5.9 (5.5 - 6.1)	5.86 (± 0.22)	5.8 (4.2 - 6.2)	5.58 (± 0.79)

Target organ	Toxin	24 h post dose		48 h post dose		168 h post dose	
		Median (min - max)	Mean (± SD)	Median (min - max)	Mean (± SD)	Median (min - max)	Mean (± SD)
Liver	[C] Allyl alcohol	8.15 (6.79 - 11.96)	8.62 (± 1.66)	7.5 (7.19 - 8.6)	7.61 (± 0.57)	8.13 (7.61 - 9.89)	8.35 (± 0.9)
Liver	[C] Allyl formate	8.65 (7.5 - 10.4)	8.76 (± 0.82)	8.5 (8 - 10)	8.74 (± 0.76)	8.3 (7.1 - 11.4)	8.72 (± 1.69)
Liver	[B] Azathioprine	7.01 (5.73 - 7.79)	6.85 (± 0.63)	7.08 (6.36 - 7.61)	7.03 (± 0.5)	8.48 (7.39 - 9.25)	8.53 (± 0.73)
Liver	[B] Bromobenzene	9.37 (8.38 - 11.6)	9.56 (± 0.99)	9.77 (8.05 - 11.21)	9.65 (± 1.52)	8.99 (8.82 - 10.77)	9.4 (± 0.83)
Liver	[C] Butylated hydroxytoluene	7.55 (6.47 - 9.34)	7.84 (± 1.05)	7.86 (7.24 - 8.33)	7.86 (± 0.47)	9.72 (8.51 - 10.34)	9.43 (± 0.75)
Liver	[D] Carbon tetrachloride	7.67 (7.24 - 8.37)	7.7 (± 0.33)	13.88 (10.84 - 16.44)	13.73 (± 2.08)	10.3 (9.38 - 12.64)	10.45 (± 1.31)
Liver	[C] Chlorpromazine	9.73 (7.64 - 11.15)	9.4 (± 1.23)	9.96 (7.63 - 12.43)	9.92 (± 1.92)	9.49 (7.57 - 10.62)	9.28 (± 1.11)
Liver	[B] Clofibrate	9.02 (8.33 - 9.86)	9.01 (± 0.54)	9.99 (8.7 - 10.41)	9.76 (± 0.66)	9.65 (9.13 - 10.98)	9.81 (± 0.7)
Liver	[B] Cyproterone acetate	7.52 (5.94 - 8.37)	7.45 (± 0.65)	6.19 (5.14 - 7.96)	6.33 (± 1.03)	10.11 (8.81 - 11.97)	10.06 (± 1.32)
Liver	[A] D-galactosamine	7.42 (5.05 - 8.14)	7.11 (± 0.93)	8.19 (4.94 - 10.21)	7.73 (± 2.18)	11.71 (11.1 - 14.54)	12.61 (± 1.7)
Liver	[B] Diethylhexylphthalate (DEHP)			8.21 (7.6 - 8.88)	8.15 (± 0.49)	9.99 (8.94 - 10.49)	9.87 (± 0.66)
Liver	[C] Dimethylformamide (DMF)	8.05 (7.4 - 8.7)	8.09 (± 0.41)	8 (7.1 - 8.5)	7.86 (± 0.59)	6.8 (6.8 - 10.2)	7.62 (± 1.47)
Liver	[C] Dimethylnitrosamine (DMN)	7.4 (7 - 8.9)	7.61 (± 0.55)	7.9 (7 - 8.2)	7.68 (± 0.59)	7.2 (6.7 - 8.3)	7.4 (± 0.6)
Liver	[A] Gadolinium chloride	8.75 (8.3 - 11.3)	9.24 (± 0.98)	12.82 (10.05 - 15.32)	13.13 (± 2.15)	14.82 (12.93 - 15.82)	14.62 (± 1.05)
Liver	[A, B, C, D, F] Hydrazine	9.47 (6.98 - 12.35)	9.56 (± 1.2)	11.14 (8.91 - 14.18)	11.52 (± 1.41)	10.59 (9.17 - 14.72)	10.89 (± 1.47)
Liver	[E] Hydrazine	8.17 (7.7 - 9.37)	8.41 (± 0.58)	10.58 (9.83 - 12.83)	10.96 (± 1.3)	11.08 (9.69 - 13.99)	11.24 (± 1.72)
Liver	[E] Indomethacin	7.08 (5.72 - 8.27)	6.98 (± 0.81)	9.6 (7.88 - 12.43)	9.8 (± 1.86)	11.25 (5.94 - 18.65)	11.22 (± 4.71)
Liver	[E] Ketoconazole			13.54 (10.38 - 14.49)	13.07 (± 1.58)	14.38 (11.27 - 18.04)	14.55 (± 2.41)
Liver	[C] Lead acetate	8 (5.6 - 9.5)	7.76 (± 1.31)	9.36 (8.5 - 10.2)	9.33 (± 0.74)	7 (6.6 - 7.5)	6.98 (± 0.36)
Liver	[A] Lipopolysaccharide (LPS)	8.38 (6.11 - 10.1)	8.34 (± 1.1)	15.6 (14.49 - 18.15)	16.18 (± 1.5)	18.48 (15.48 - 25.53)	19.21 (± 4.24)

Target organ	Toxin	24 h post dose		48 h post dose		168 h post dose	
		Median (min - max)	Mean (± SD)	Median (min - max)	Mean (± SD)	Median (min - max)	Mean (± SD)
Liver	[B] Methapyrilene	8.89 (6.84 - 10.05)	8.71 (± 1.1)	10.05 (7.44 - 11.27)	9.77 (± 1.45)	9.38 (9.17 - 10.45)	9.67 (± 0.55)
Liver	[E] Methylene dianiline	8.05 (6.61 - 10.77)	8.24 (± 1.32)	11.38 (11.05 - 13.16)	11.75 (± 0.84)	11.44 (8.94 - 12.16)	10.95 (± 1.36)
Liver	[C] Monocrotaline	8.8 (7.6 - 10.2)	8.84 (± 0.89)	9.7 (8.8 - 10.4)	9.62 (± 0.61)	8.9 (7.7 - 9.6)	8.8 (± 0.72)
Liver	[C] N-methylformamide (NMF)	7 (5.7 - 8.8)	7.06 (± 1)	8.68 (8.14 - 9.72)	8.86 (± 0.6)	7.8 (7.8 - 8.3)	7.94 (± 0.22)
Liver	[D] Phalloidin (chronic)			6.74 (6.48 - 7.43)	6.88 (± 0.41)	9.86 (6.94 - 10.25)	9.05 (± 1.42)
Liver	[E] Phenyl diisothiocyanate	10.13 (5.66 - 14.16)	9.68 (± 3.07)	11.27 (7.11 - 11.93)	10.36 (± 1.99)	11.37 (11.12 - 13.99)	12.11 (± 1.25)
Liver	[E] Phenyl isothiocyanate	6.11 (5.55 - 8.1)	6.46 (± 0.86)	8.27 (7.27 - 8.88)	8.24 (± 0.65)	9.33 (8.66 - 13.38)	10.27 (± 1.96)
Liver	[B] Retinyl palmitate	9.14 (8.42 - 11.35)	9.38 (± 0.94)	11.86 (10.76 - 12.44)	11.78 (± 0.67)	8.56 (8.19 - 10.06)	8.79 (± 0.75)
Liver	[B] Sodium Valproate			9.51 (3.94 - 19.7)	9.92 (± 6.44)	8.44 (7.49 - 10.85)	8.76 (± 1.35)
Liver	[C] a-Naphthylisothiocyanate (ANIT)	11.25 (7.34 - 12.4)	10.63 (± 1.77)	7.05 (6.74 - 11.28)	8.65 (± 2.4)	8.52 (8.07 - 9.95)	8.72 (± 0.75)
Kidney	[D] 2-Bromophenol	9.1 (8 - 10.5)	9.1 (± 0.73)	14.1 (11.2 - 22.7)	16.64 (± 5.54)	17.4 (14.9 - 17.8)	16.76 (± 1.21)
Kidney	[E] 3,5-Dichloroaniline hydrochloride	7.72 (6.22 - 8.88)	7.78 (± 0.86)	8.94 (8.27 - 11.16)	9.55 (± 1.28)	11.05 (9.71 - 11.99)	11.14 (± 0.94)
Kidney	[E] Atractyloside	7.6 (6.05 - 15.04)	8.78 (± 2.87)	11.55 (11.05 - 12.1)	11.65 (± 0.42)	10.55 (9.49 - 11.71)	10.7 (± 0.84)
Kidney	[D] Bromoethylamine hydrobromide	6.95 (6.3 - 9.07)	7.33 (± 1.05)	9.2 (8.41 - 10.9)	9.52 (± 1.12)	9.8 (8.6 - 13.2)	10.65 (± 2.06)
Kidney	[D] Cephaloridine	7.3 (6.9 - 8.1)	7.41 (± 0.41)	12.5 (11.4 - 16.5)	12.98 (± 2.08)	11.2 (8.5 - 12.6)	10.54 (± 1.69)
Kidney	[B] Chlorethanamine	9.47 (8.62 - 10.62)	9.53 (± 0.71)	10.28 (9.86 - 10.91)	10.32 (± 0.38)	9.57 (8.89 - 9.64)	9.45 (± 0.32)
Kidney	[A] Cisplatin	8.5 (7.7 - 9.1)	8.49 (± 0.53)	13 (8.2 - 17.5)	12.34 (± 4.03)	13.8 (8.9 - 17.4)	13.63 (± 3.61)
Kidney	[A] D-limonene (chronic)						
Kidney	[E] Dichlorophenyl succinimide	7.85 (7.05 - 10.94)	8.11 (± 1.05)	11.1 (10.38 - 12.32)	11.44 (± 0.86)	10.55 (8.27 - 13.99)	10.51 (± 2.19)
Kidney	[D] Ethylene glycol	8.1 (7.6 - 8.7)	8.13 (± 0.36)	11.3 (8 - 13.8)	10.68 (± 2.26)	11.9 (10.5 - 14.7)	12.06 (± 1.61)

Target organ	Toxin	24 h post dose		48 h post dose		168 h post dose	
		Median (min - max)	Mean (± SD)	Median (min - max)	Mean (± SD)	Median (min - max)	Mean (± SD)
Kidney	[A] Folic acid	7.55 (5.1 - 9.1)	7.63 (± 1.12)	11.65 (6.2 - 40.6)	15.48 (± 12.49)	13.3 (8.6 - 19.2)	12.94 (± 4.44)
Kidney	[A] Gentamicin	8.45 (7.9 - 9.1)	8.49 (± 0.37)	9.1 (8.7 - 24.1)	13.72 (± 6.9)	11.1 (10.2 - 12.7)	11.34 (± 0.98)
Kidney	[B] Maleic acid	10.29 (8.87 - 13.42)	10.66 (± 1.28)	11.06 (9.84 - 14.72)	11.51 (± 1.89)	11.15 (9.85 - 12.67)	11.18 (± 1.08)
Kidney	[A] N-phenylanthranilic acid (chronic)	8.18 (7.1 - 9.16)	8.2 (± 0.73)	12.32 (11.38 - 14.21)	12.61 (± 1.12)	15.15 (14.01 - 18.26)	15.52 (± 1.6)
Kidney	[D] Para-aminophenol	8.15 (7.2 - 8.8)	8 (± 0.61)	12.8 (8.7 - 20.3)	13.8 (± 4.58)	11.4 (9.5 - 19.6)	13.24 (± 4.31)
Kidney	[A] Puromycin			9.4 (7.7 - 13)	10.06 (± 2.22)	12 (10.7 - 14.4)	12.22 (± 1.41)
Kidney	[B] Vancomycin hydrochloride			11.75 (8.37 - 12.21)	10.91 (± 1.6)	10.69 (10.08 - 11.22)	10.71 (± 0.44)
Liver & Kidney	[E] Acetaminophen	7.44 (5.11 - 8.05)	7.15 (± 1.03)	9.55 (4.72 - 10.27)	8.73 (± 2.3)	11.05 (10.71 - 11.32)	11 (± 0.26)
Liver & Kidney	[B] Aurothiomalate	8.59 (7.73 - 14.25)	9.49 (± 2.12)	8.48 (2.8 - 10.26)	7.65 (± 2.84)	10.68 (10.27 - 11.86)	11.02 (± 0.76)
Liver & Kidney	[C] Chloroform	7.1 (6 - 8.7)	7.11 (± 0.73)	8.7 (7.8 - 11.5)	9.06 (± 1.42)	8.1 (7.5 - 8.5)	8.04 (± 0.4)
Liver & Kidney	[D] Cyclosporin	15.75 (8.55 - 18.9)	14.58 (± 3.57)	13.35 (6.9 - 22.9)	13.67 (± 5.27)	12.1 (10.25 - 17)	13.01 (± 2.54)
Liver & Kidney	[D] Dichlorobenzene	8.65 (6.8 - 9.5)	8.37 (± 0.86)	8.3 (7.2 - 9.6)	8.36 (± 1.13)	12.4 (7.8 - 14.8)	11.56 (± 3.25)
Liver & Kidney	[C] Ethionine	6.85 (6.02 - 10.35)	7.33 (± 1.47)	8.54 (7.71 - 9.68)	8.57 (± 0.78)	8.79 (7.54 - 9.11)	8.46 (± 0.71)
Liver & Kidney	[B] Hexachlorobutadiene (HCBD)			7.94 (6.44 - 8.49)	7.69 (± 0.83)	8.99 (8.16 - 10.66)	9.18 (± 1.02)
Liver & Kidney	[B] Mercuric chloride	8.71 (6.33 - 10.1)	8.49 (± 1.26)	8.77 (7.1 - 9.66)	8.38 (± 1.1)	9.89 (9.1 - 10.85)	9.89 (± 0.65)
Liver & Kidney	[E] Microcystin-LR			7.44 (6.49 - 10.03)	7.75 (± 1.43)	10.32 (9.88 - 11.77)	10.68 (± 0.84)
Liver & Kidney	[E] Rotenone	7.51 (5.88 - 10.32)	7.68 (± 1.44)	12.07 (9.38 - 14.21)	11.93 (± 2.55)	11.6 (11.25 - 13.77)	11.96 (± 0.95)
Liver & Kidney	[E] S-(1,2-dichlorovinyl)-cysteine (DCVC)	7.16 (3.28 - 9.21)	6.99 (± 1.8)	12.77 (10.82 - 13.88)	12.62 (± 1.16)	11.71 (10.88 - 13.1)	11.85 (± 0.8)
Liver & Kidney	[D] Thioacetamide	7.25 (6.2 - 8.04)	7.19 (± 0.56)	8.3 (6.4 - 9.49)	8.09 (± 1.19)	8.1 (7.4 - 10.2)	8.32 (± 1.1)
Pancreas	[E] 1-Cyano-2-hydroxy-3-butene	5.8 (4.77 - 9.05)	6.12 (± 1.25)	8.94 (6.77 - 10.03)	8.53 (± 1.52)	9.33 (8.49 - 11.05)	9.41 (± 0.98)

Target organ	Toxin	24 h post dose		48 h post dose		168 h post dose	
		Median (min - max)	Mean (± SD)	Median (min - max)	Mean (± SD)	Median (min - max)	Mean (± SD)
Pancreas	[C] Caerulin	8.35 (6.8 - 9.6)	8.35 (± 0.78)	9.2 (8.2 - 11.4)	9.56 (± 1.19)	8.1 (7.9 - 8.7)	8.16 (± 0.33)
Pancreas	[E] L-arginine	5.69 (3.72 - 20.43)	7.02 (± 3.64)	10.21 (7.22 - 14.49)	10.69 (± 2.2)	8.02 (6.72 - 12.21)	8.63 (± 1.97)
Pancreas	[B] Streptozotocin	9.72 (8.65 - 10.16)	9.51 (± 0.47)	10.16 (9.33 - 11.04)	10.12 (± 0.67)	9.94 (8.49 - 10.81)	9.89 (± 0.93)
Testicular	[D] 1,3-Dinitrobenzene	8.2 (7.1 - 9.4)	8.17 (± 0.74)	11.7 (9 - 14)	11.54 (± 2.03)	14.7 (13.5 - 15.7)	14.46 (± 0.89)
Testicular	[C] Cadmium chloride	7.15 (6.2 - 8.3)	7.11 (± 0.65)	7.6 (6.1 - 10)	7.78 (± 1.43)	8.5 (7.4 - 10.3)	8.74 (± 1.11)
Testicular	[D] Cadmium chloride	7.27 (6.17 - 7.88)	7.13 (± 0.54)	9.79 (8.03 - 13.05)	10.41 (± 1.92)	9.72 (8.51 - 10.49)	9.56 (± 0.95)
Testicular	[D] Carbendazim	8.95 (8.2 - 9.5)	8.95 (± 0.49)	12.8 (11.9 - 15.4)	13.36 (± 1.36)	14.1 (11.8 - 24.2)	15.76 (± 4.86)
Testicular	[D] Di-n-pentyl-phthalate	8.05 (7.63 - 9.14)	8.19 (± 0.58)	7.66 (7.47 - 8.02)	7.7 (± 0.21)	7.34 (6.95 - 10.77)	7.96 (± 1.59)
Testicular	[D] Ethane dimethane sulfonate (EDS)	7.95 (7.4 - 8.8)	8.12 (± 0.47)	12.7 (11.6 - 14.4)	12.9 (± 1.26)	13.1 (12.9 - 16.7)	13.98 (± 1.59)
Testicular	[D] Methoxyacetic acid	8.59 (8.14 - 10.1)	8.77 (± 0.66)	10.19 (10.05 - 11.83)	10.74 (± 0.85)	8.79 (6.33 - 11.46)	8.44 (± 2.07)
Multiple organ	[B] Adriamycin	10.3 (8.9 - 11.9)	10.37 (± 0.87)	9.6 (8.94 - 13.93)	10.42 (± 2.01)	7.55 (6.22 - 9.49)	7.65 (± 1.2)
Multiple organ	[C] Amphotericin B	7.85 (6.1 - 13.5)	8.53 (± 2.46)	9.1 (7.7 - 10.3)	9.07 (± 1.13)	8.1 (6.6 - 9.4)	8.04 (± 1.2)
Multiple organ	[C] Azaserine	7 (5.7 - 8.8)	6.99 (± 1.06)	7.5 (5.7 - 7.9)	7.14 (± 0.87)	8 (6.6 - 10.5)	8.14 (± 1.6)
Multiple organ	[A] Dexamethasone	23.85 (18.2 - 26.8)	22.82 (± 3.05)	28.2 (15.7 - 33.2)	26.36 (± 7.54)	19.1 (12.15 - 42)	21.91 (± 11.88)
Multiple organ	[E] Mitomycin-C	7.08 (6.11 - 8.72)	7.09 (± 0.84)	12.49 (11.38 - 14.82)	12.85 (± 1.38)	11.99 (9.94 - 13.32)	11.7 (± 1.41)
Physiological stressor	[C] 1,1-Dichloroethylene & maleic acid	8.55 (7.7 - 10.9)	8.81 (± 0.94)	9.2 (8.8 - 12.7)	10.06 (± 1.66)	8.4 (7.6 - 9.8)	8.6 (± 0.84)
Physiological stressor	[C] 2,4-Dinitrophenol	7.9 (7.5 - 12.3)	8.38 (± 1.42)	8.8 (8.3 - 10.8)	9.22 (± 1.01)	8.1 (7.5 - 8.4)	8.04 (± 0.39)
Physiological stressor	[B] 4-Pentenoic acid	9.48 (8.16 - 11.07)	9.58 (± 0.98)	9.51 (9.17 - 11.99)	10.03 (± 1.13)	9.36 (8.94 - 10.56)	9.52 (± 0.66)
Physiological stressor	[D] Acetazolamide	8.8 (7.5 - 9.9)	8.81 (± 0.81)	11.01 (9.6 - 21.8)	12.86 (± 5.04)	8.1 (7.1 - 17.6)	9.88 (± 4.41)
Physiological stressor	[C] Acivicin	7.95 (7.1 - 9.2)	8.03 (± 0.72)	9.9 (9.7 - 10.4)	9.98 (± 0.26)	8.6 (7.4 - 8.7)	8.4 (± 0.56)
Physiological stressor	[E] Ammonium chloride			12.1 (10.55 - 12.71)	11.66 (± 0.95)	11.05 (10.44 - 12.43)	11.2 (± 0.74)

Target organ	Toxin	24 h post dose		48 h post dose		168 h post dose	
		Median (min - max)	Mean (± SD)	Median (min - max)	Mean (± SD)	Median (min - max)	Mean (± SD)
Physiological stressor	[D] Carboplatin	7.85 (7 - 8.6)	7.81 (± 0.41)	16 (13.8 - 18.9)	15.9 (± 1.93)	14.9 (10.9 - 21.1)	15.5 (± 3.65)
Physiological stressor	[A] Choline and choline/methionine deficiency (chronic)	7.66 (6.88 - 8.44)	7.68 (± 0.44)	12.9 (11.8 - 14.9)	13.34 (± 1.35)	12.93 (11.43 - 15.32)	13.22 (± 1.42)
Physiological stressor	[B] Food restriction (chronic)	9.2 (7.38 - 10.61)	9.09 (± 1.15)	8.07 (7.31 - 9.5)	8.24 (± 0.99)	8.53 (7.98 - 9.62)	8.71 (± 0.72)
Physiological stressor	[D] Furosemide	7.35 (6.9 - 8.5)	7.49 (± 0.57)	11 (8.4 - 16.7)	11.46 (± 3.24)	10.5 (8.7 - 14.3)	10.64 (± 2.27)
Physiological stressor	[B] Insulin	9.77 (9.35 - 10.04)	9.73 (± 0.22)	9.69 (8.81 - 10.08)	9.52 (± 0.59)	10.35 (10.13 - 11.24)	10.56 (± 0.46)
Physiological stressor	[E] Methotrexate	8.99 (7.05 - 11.32)	9.09 (± 1.2)	9.99 (9.77 - 10.71)	10.17 (± 0.41)	10.49 (9.6 - 11.32)	10.55 (± 0.75)
Physiological stressor	[A] Partial hepatectomy	8.08 (7.37 - 8.65)	7.99 (± 0.42)	10 (8.36 - 10.39)	9.71 (± 0.79)	11.89 (10.75 - 12.22)	11.66 (± 0.64)
Physiological stressor	[A] Phenobarbital (chronic)	7.85 (6.9 - 8.3)	7.76 (± 0.41)	12.3 (9.8 - 15.7)	12.56 (± 2.53)	15.1 (11.2 - 18.7)	14.88 (± 2.77)
Physiological stressor	[A] Pregnenolone 16 alpha carbonitrile (chronic)						
Physiological stressor	[A] Probenecid	8.27 (7.33 - 9.32)	8.32 (± 0.54)	12.04 (10.38 - 14.04)	12.31 (± 1.53)	13.26 (9.6 - 15.98)	12.84 (± 2.38)
Physiological stressor	[C] Rosiglitazone	8.75 (7.4 - 10.2)	8.94 (± 1.04)	8.8 (7.8 - 8.9)	8.6 (± 0.45)	7.8 (7.1 - 8.8)	7.88 (± 0.68)
Physiological stressor	[C] Rosiglitazone (chronic)	7.5 (6 - 8.9)	7.5 (± 0.73)				
Physiological stressor	[E] Sodium bicarbonate	8.44 (8.01 - 9.08)	8.49 (± 0.34)				
Physiological stressor	[A] Unilateral nephrectomy	8.95 (8.5 - 9.69)	9.06 (± 0.43)	11.41 (10.37 - 13.19)	11.79 (± 1.2)	12.28 (12.12 - 13.34)	12.52 (± 0.51)
Physiological stressor	[B] Water deprivation (chronic)	9.87 (9.24 - 12.74)	10.27 (± 1.05)	10.68 (7.89 - 11.82)	10.35 (± 1.37)	9.24 (8.54 - 14.22)	10.1 (± 2.13)
No Effect	[E] Acetaminophen (chronic)	10.77 (9.77 - 10.99)	10.57 (± 0.48)			12.82 (11.44 - 14.71)	13 (± 1.44)
No Effect	[C] Buthionine sulphoxime	8.25 (7.4 - 9.2)	8.27 (± 0.55)	10.2 (10 - 11)	10.46 (± 0.5)	8.2 (7.3 - 9.6)	8.32 (± 0.82)
No Effect	[C] Ferrous sulphate	7.85 (6.5 - 8.6)	7.73 (± 0.56)	8.1 (7.5 - 9.6)	8.24 (± 0.81)	8 (7.3 - 8.7)	8.08 (± 0.58)
No Effect	[B] Ifosfamide	8.77 (5.86 - 11.69)	8.61 (± 1.57)	9.47 (7.65 - 12.9)	10.36 (± 2.28)	9.66 (8.51 - 11.95)	9.94 (± 1.42)
No Effect	[B] Lithocholic acid	9.14 (8.05 - 10.56)	9.08 (± 0.77)	12.29 (10.83 - 13.83)	12.47 (± 1.22)	9.7 (8.82 - 10.14)	9.6 (± 0.49)

Target organ	Toxin	24 h post dose		48 h post dose		168 h post dose	
		Median (min - max)	Mean (± SD)	Median (min - max)	Mean (± SD)	Median (min - max)	Mean (± SD)
No Effect	[E] Paraquat			12.88 (10.99 - 16.93)	13.1 (± 2.29)	13.04 (11.99 - 14.16)	13.06 (± 0.83)
No Effect	[D] Potassium dichromate	7.9 (5.7 - 9)	7.7 (± 1.08)	11.6 (9.8 - 14)	11.54 (± 1.56)	12.1 (9.1 - 13.1)	11.62 (± 1.54)
No Effect	[C] Trichlorethylene	8 (7 - 8.7)	7.83 (± 0.61)	8 (7.1 - 9.1)	8.06 (± 0.72)	8.9 (7.3 - 9.7)	8.72 (± 0.89)

A-F: Indicates Pharmaceutical Company & sample origin

Supplementary Table S25. Summary statistics for serum albumin (g/L) at 24 hrs, 48 hrs and 168 hrs post high dose.

Target organ	Toxin	24 h post dose		48 h post dose		168 h post dose	
		Median (min - max)	Mean (± SD)	Median (min - max)	Mean (± SD)	Median (min - max)	Mean (± SD)
Liver	[E] 1,1-Dichloroethylene	41.95 (39.45 - 43.45)	41.75 (± 1.16)	35.45 (33.45 - 38.45)	35.65 (± 1.92)	35.45 (33.45 - 36.45)	35.25 (± 1.3)
Liver	[E] 1,2,3,4,5,6-hexachlorocyclohexane	39.45 (36.45 - 40.45)	38.95 (± 1.08)	35.45 (34.45 - 36.45)	35.45 (± 0.71)	37.45 (36.45 - 37.45)	37.25 (± 0.45)
Liver	[B] 1-Fluoropentane	37.95 (32.95 - 40.95)	37.55 (± 2.41)	35.95 (34.95 - 37.95)	36.15 (± 1.1)	34.95 (34.95 - 36.95)	35.35 (± 0.89)
Liver	[B] 2,4,6-Trihydroxyacetophenone (THA)	36.45 (34.45 - 39.45)	36.65 (± 1.55)	34.45 (33.45 - 38.45)	35.45 (± 2)	35.45 (35.45 - 38.45)	36.45 (± 1.41)
Liver	[B] 4-Amino-2,6-dichlorophenol (ADCP)	37.55 (35.05 - 42.05)	38.05 (± 2.05)	39.05 (38.05 - 40.05)	39.05 (± 1)	39.05 (35.05 - 43.05)	39.05 (± 2.83)
Liver	[C] Aflatoxin	36.95 (35.45 - 40.45)	37.45 (± 1.56)	34.45 (31.45 - 34.45)	33.45 (± 1.41)	34.45 (28.45 - 38.45)	33.45 (± 4.36)
Liver	[C] Allyl alcohol	30 (28.3 - 39.6)	30.83 (± 3.25)	29.7 (28.1 - 32.8)	30.16 (± 1.74)	36.1 (34.3 - 39.6)	36.28 (± 2.04)
Liver	[C] Allyl formate	34.65 (27.05 - 38.28)	33.71 (± 3.31)	37.59 (36.2 - 37.87)	37.31 (± 0.65)	36.75 (36.05 - 38.74)	37.17 (± 1.02)
Liver	[B] Azathioprine	43.55 (42.05 - 46.05)	43.75 (± 1.34)	39.05 (37.05 - 43.05)	39.45 (± 2.61)	43.05 (40.05 - 45.05)	43.05 (± 1.87)
Liver	[B] Bromobenzene	41.95 (36.45 - 42.45)	40.95 (± 2.01)	36.45 (35.45 - 38.45)	36.85 (± 1.14)	43.45 (43.45 - 44.45)	43.85 (± 0.55)
Liver	[C] Butylated hydroxytoluene	39.85 (37.3 - 41.5)	39.62 (± 1.2)	35.8 (32.4 - 37.1)	35.32 (± 1.95)	37.6 (36 - 39.5)	37.72 (± 1.65)
Liver	[D] Carbon tetrachloride	36.82 (33.66 - 38.67)	36.73 (± 1.35)	34.45 (30.45 - 38.45)	33.85 (± 3.13)	38.45 (37.45 - 41.45)	39.05 (± 1.52)
Liver	[C] Chlorpromazine	36.6 (24.35 - 41.25)	35.92 (± 4.94)	37.45 (33.55 - 38.35)	36.31 (± 2.24)	38.15 (34.35 - 39.25)	37.37 (± 1.96)

Target organ	Toxin	24 h post dose		48 h post dose		168 h post dose	
		Median (min - max)	Mean (± SD)	Median (min - max)	Mean (± SD)	Median (min - max)	Mean (± SD)
Liver	[B] Clofibrate	38.45 (36.45 - 40.45)	38.35 (± 1.29)	37.45 (37.45 - 39.45)	37.85 (± 0.89)	37.45 (36.45 - 38.45)	37.45 (± 0.71)
Liver	[B] Cyproterone acetate	40.95 (36.95 - 43.95)	40.55 (± 1.78)	36.95 (34.95 - 39.95)	37.35 (± 1.82)	40.95 (37.95 - 43.95)	41.15 (± 2.17)
Liver	[A] D-galactosamine	37.77 (32.33 - 39.76)	37.19 (± 2.29)	33.45 (4.45 - 34.45)	27.65 (± 13.03)	37.45 (35.45 - 40.45)	37.65 (± 1.92)
Liver	[B] Diethylhexylphthalate (DEHP)			40.45 (40.45 - 42.45)	41.05 (± 0.89)	42.45 (40.45 - 42.45)	41.85 (± 0.89)
Liver	[C] Dimethylformamide (DMF)	37.85 (35.1 - 39.7)	37.73 (± 1.26)	37.3 (35.1 - 37.4)	36.46 (± 1.24)	38.9 (36.4 - 40.7)	38.66 (± 1.67)
Liver	[C] Dimethylnitrosamine (DMN)	34.6 (33.15 - 37.05)	34.85 (± 1.41)	26.05 (24.85 - 28.95)	26.53 (± 1.67)	35.85 (34.25 - 36.95)	35.65 (± 1.1)
Liver	[A] Gadolinium chloride	36.45 (35.95 - 38.95)	36.75 (± 1.03)	36.95 (35.95 - 36.95)	36.55 (± 0.55)	35.95 (35.95 - 38.95)	37.15 (± 1.64)
Liver	[A, B, C, D, F] Hydrazine	37.25 (34 - 40.7)	37.1 (± 1.67)	36.45 (31.45 - 39.45)	36.39 (± 2.21)	38.45 (15.95 - 44.45)	37.22 (± 6.29)
Liver	[E] Hydrazine	37.75 (37.5 - 37.8)	37.71 (± 0.12)	36.85 (36.7 - 37)	36.85 (± 0.13)	36.6 (36 - 37.5)	36.68 (± 0.54)
Liver	[E] Indomethacin	25.45 (22.45 - 26.45)	24.95 (± 1.43)	24.45 (22.45 - 32.45)	25.65 (± 4.15)	26.45 (25.45 - 38.13)	28.79 (± 5.27)
Liver	[E] Ketoconazole			37.45 (35.45 - 38.45)	37.05 (± 1.14)	36.45 (35.45 - 36.45)	36.05 (± 0.55)
Liver	[C] Lead acetate	33.2 (28 - 38.3)	33.35 (± 3.08)	33.2 (29 - 37.02)	32.76 (± 3.14)	39.4 (34.8 - 39.7)	38.3 (± 2.04)
Liver	[A] Lipopolysaccharide (LPS)	32.95 (29.45 - 37.75)	32.98 (± 2.19)	31.45 (30.45 - 36.27)	32.22 (± 2.31)	37.45 (36.45 - 38.45)	37.25 (± 0.84)
Liver	[B] Methapyrilene	36.45 (31.45 - 39.45)	36.25 (± 2.04)	34.45 (30.45 - 35.45)	33.85 (± 2.07)	38.45 (36.45 - 40.45)	38.45 (± 1.58)
Liver	[E] Methylene dianiline	37.45 (35.95 - 42.95)	38.45 (± 2.22)	32.95 (32.95 - 34.95)	33.55 (± 0.89)	35.95 (33.95 - 37.95)	36.15 (± 1.48)
Liver	[C] Monocrotaline	37.35 (34.6 - 40.3)	37.28 (± 1.65)	36.3 (35.1 - 37.9)	36.32 (± 1.14)	36.8 (35.9 - 41.5)	37.84 (± 2.22)
Liver	[C] N-methylformamide (NMF)	39.45 (35.2 - 40.4)	39 (± 1.53)	36.2 (34.6 - 38.5)	36.22 (± 1.5)	37.4 (36.1 - 38.5)	37.3 (± 0.91)
Liver	[D] Phalloidin (chronic)			33.08 (30.74 - 35.76)	33.14 (± 1.79)	35.19 (33.98 - 40.06)	36.27 (± 2.68)
Liver	[E] Phenyl diisothiocyanate	38.45 (37.45 - 43.51)	39.33 (± 2.38)	33.45 (31.45 - 35.45)	33.25 (± 1.79)	37.31 (33.45 - 38.52)	36.57 (± 2.11)
Liver	[E] Phenyl isothiocyanate	41.45 (38.45 - 43.45)	41.15 (± 1.49)	36.45 (35.45 - 36.45)	36.25 (± 0.45)	36.45 (35.45 - 36.45)	36.05 (± 0.55)

Target organ	Toxin	24 h post dose		48 h post dose		168 h post dose	
		Median (min - max)	Mean (± SD)	Median (min - max)	Mean (± SD)	Median (min - max)	Mean (± SD)
Liver	[B] Retinyl palmitate	44.05 (40.05 - 47.05)	43.35 (± 2.31)	38.05 (36.05 - 40.05)	38.05 (± 1.58)	43.05 (41.05 - 46.05)	43.05 (± 2.12)
Liver	[B] Sodium Valproate			37.31 (34.95 - 38.95)	36.82 (± 1.81)	35.95 (34.95 - 36.95)	35.9 (± 0.94)
Liver	[C] a-Naphthylisothiocyanate (ANIT)	34.85 (31.45 - 38.15)	34.94 (± 2.06)	30.95 (27.65 - 31.65)	30.11 (± 1.73)	37.35 (36.55 - 39.35)	37.79 (± 1.17)
Kidney	[D] 2-Bromophenol	36.45 (34.45 - 38.45)	36.75 (± 1.06)	37.45 (36.45 - 39.45)	38.05 (± 1.34)	38.45 (38.45 - 39.45)	38.65 (± 0.45)
Kidney	[E] 3,5-Dichloroaniline hydrochloride	35.95 (32.95 - 38.95)	35.85 (± 1.85)	30.95 (30.95 - 31.95)	31.35 (± 0.55)	33.95 (31.95 - 34.95)	33.75 (± 1.3)
Kidney	[E] Atractyloside	38.45 (32.45 - 41.45)	38.36 (± 2.63)	35.45 (32.45 - 36.45)	34.85 (± 1.52)	34.45 (33.45 - 36.45)	34.45 (± 1.22)
Kidney	[D] Bromoethylamine hydrobromide	38.2 (36.45 - 41.45)	38.36 (± 1.34)	36.45 (28.45 - 40.45)	35.73 (± 4.67)	36.45 (35.45 - 38.16)	36.39 (± 1.11)
Kidney	[D] Cephaloridine	37.95 (36.45 - 43.45)	38.35 (± 2.28)	38.45 (37.45 - 41.45)	39.05 (± 1.82)	40.45 (39.45 - 40.45)	40.05 (± 0.55)
Kidney	[B] Chlorethanamine	39.45 (37.45 - 42.45)	39.75 (± 1.57)	35.45 (34.45 - 36.45)	35.25 (± 0.84)	37.45 (37.45 - 38.45)	37.65 (± 0.45)
Kidney	[A] Cisplatin	38.45 (36.45 - 40.45)	38.42 (± 1.25)	39.45 (37.45 - 40.45)	39.25 (± 1.3)	37.45 (35.45 - 40.45)	37.64 (± 1.92)
Kidney	[A] D-limonene (chronic)						
Kidney	[E] Dichlorophenyl succinimide	39.45 (36.95 - 40.95)	39.55 (± 1.43)	35.95 (34.95 - 37.95)	35.95 (± 1.22)	35.95 (34.95 - 37.95)	35.95 (± 1.22)
Kidney	[D] Ethylene glycol	36.95 (33.45 - 38.45)	36.65 (± 1.4)	38.45 (37.45 - 40.45)	38.45 (± 1.22)	38.45 (35.45 - 39.45)	38.05 (± 1.67)
Kidney	[A] Folic acid	33.95 (31.95 - 36.95)	34.15 (± 1.81)	33.45 (31.95 - 37.95)	34.29 (± 2.26)	38.95 (38.8 - 39.46)	39.02 (± 0.25)
Kidney	[A] Gentamicin	36.45 (32.95 - 38.95)	36.15 (± 2.25)	38.95 (34.95 - 40.95)	37.95 (± 2.45)	39.95 (38.95 - 43.95)	40.35 (± 2.07)
Kidney	[B] Maleic acid	41.05 (38.05 - 44.05)	41.35 (± 1.64)	39.05 (38.05 - 42.05)	39.25 (± 1.64)	43.05 (41.05 - 43.05)	42.45 (± 0.89)
Kidney	[A] N-phenylanthranilic acid (chronic)	36.07 (34.45 - 39.11)	36.56 (± 1.59)	38.45 (35.45 - 54.45)	40.65 (± 7.82)	37.45 (34.45 - 40.45)	37.39 (± 2.49)
Kidney	[D] Para-aminophenol	32.45 (28.45 - 35.45)	32.15 (± 2.41)	36.45 (31.45 - 38.45)	35.25 (± 2.77)	38.45 (37.45 - 38.45)	38.05 (± 0.55)
Kidney	[A] Puromycin			37.45 (35.45 - 38.45)	37.05 (± 1.14)	35.45 (29.45 - 36.45)	33.85 (± 2.88)
Kidney	[B] Vancomycin hydrochloride			36.45 (36.45 - 37.45)	36.65 (± 0.45)	36.45 (35.45 - 39.45)	37.25 (± 1.64)

Target organ	Toxin	24 h post dose		48 h post dose		168 h post dose	
		Median (min - max)	Mean (± SD)	Median (min - max)	Mean (± SD)	Median (min - max)	Mean (± SD)
Liver & Kidney	[E] Acetaminophen	42.95 (40.95 - 44.95)	43.15 (± 1.4)	35.95 (32.95 - 37.95)	35.75 (± 1.92)	38.95 (37.95 - 38.95)	38.55 (± 0.55)
Liver & Kidney	[B] Aurothiomalate	41.05 (37.05 - 43.05)	40.75 (± 1.7)	37.05 (35.05 - 39.05)	36.85 (± 1.48)	38.05 (37.05 - 43.05)	39.65 (± 2.7)
Liver & Kidney	[C] Chloroform	41.15 (37 - 44.2)	40.98 (± 2.22)	38.9 (33.5 - 40.5)	37.9 (± 3.01)	39.6 (38.3 - 40.4)	39.52 (± 0.8)
Liver & Kidney	[D] Cyclosporin	37.45 (35.45 - 40.45)	37.39 (± 1.53)	33.45 (29.45 - 37.71)	33.16 (± 3.02)	37.45 (35.45 - 38.05)	37.17 (± 0.99)
Liver & Kidney	[D] Dichlorobenzene	37.45 (34.45 - 40.45)	36.95 (± 1.78)	37.45 (34.45 - 39.45)	37.25 (± 1.92)	43.45 (38.45 - 45.45)	42.65 (± 2.77)
Liver & Kidney	[C] Ethionine	40.9 (39 - 41.9)	40.68 (± 1.14)	36.8 (35.7 - 37.2)	36.7 (± 0.6)	38.7 (35.5 - 39.6)	38.14 (± 1.58)
Liver & Kidney	[B] Hexachlorobutadiene (HCBD)			36.45 (34.45 - 36.45)	35.85 (± 0.89)	37.45 (34.45 - 40.45)	37.85 (± 2.3)
Liver & Kidney	[B] Mercuric chloride	34.45 (31.95 - 38.95)	34.85 (± 1.85)	33.95 (32.95 - 34.95)	33.75 (± 0.84)	37.1 (35.95 - 38.95)	37.33 (± 1.16)
Liver & Kidney	[E] Microcystin-LR			33.45 (32.45 - 37.44)	34.65 (± 2.17)	37.31 (35.45 - 38.45)	37.02 (± 1.13)
Liver & Kidney	[E] Rotenone	40.74 (35.88 - 47.45)	40.82 (± 4.35)	40.95 (37.24 - 46.45)	41.4 (± 3.99)	36.38 (33.45 - 38.52)	36.22 (± 2.07)
Liver & Kidney	[E] S-(1,2-dichlorovinyl)-cysteine (DCVC)	38.45 (36.95 - 44.95)	39.35 (± 2.84)	34.95 (33.95 - 35.95)	34.75 (± 0.84)	34.95 (33.95 - 36.95)	35.35 (± 1.14)
Liver & Kidney	[D] Thioacetamide	36.89 (34.79 - 38.69)	36.63 (± 1.19)	37.11 (32.95 - 39.95)	37.11 (± 2.69)	40.95 (38.95 - 42.95)	41.15 (± 1.48)
Pancreas	[E] 1-Cyano-2-hydroxy-3-butene	41.45 (8.45 - 47.45)	38.8 (± 11.06)	37.44 (34.45 - 38.45)	37.05 (± 1.67)	33.45 (32.45 - 36.45)	34.45 (± 1.87)
Pancreas	[C] Caerulin	34.95 (33.45 - 38.45)	35.82 (± 1.85)	33.45 (30.45 - 36.45)	33.65 (± 2.17)	38.04 (36.45 - 40.45)	38.57 (± 1.81)
Pancreas	[E] L-arginine	41.95 (31.95 - 45.95)	40.56 (± 3.62)	34.95 (32.95 - 36.53)	34.79 (± 1.19)	34.45 (31.95 - 35.95)	34.05 (± 1.52)
Pancreas	[B] Streptozotocin	37.45 (35.45 - 39.45)	37.55 (± 1.29)	36.45 (35.45 - 37.45)	36.65 (± 0.84)	35.45 (34.45 - 37.45)	35.65 (± 1.1)
Testicular	[D] 1,3-Dinitrobenzene	35.45 (34.45 - 38.45)	35.95 (± 1.27)	33.45 (32.45 - 37.02)	34.16 (± 2.01)	39.45 (36.45 - 40.45)	39.25 (± 1.64)
Testicular	[C] Cadmium chloride	34.3 (29.9 - 39.3)	34.29 (± 2.85)	28.6 (24.9 - 38.4)	31.3 (± 5.66)	36.5 (35.4 - 38.9)	36.86 (± 1.47)
Testicular	[D] Cadmium chloride	30.32 (27.12 - 35.64)	30.64 (± 2.46)	29.34 (23.74 - 29.91)	27.82 (± 2.69)	36.6 (34.79 - 37.78)	36.47 (± 1.07)
Testicular	[D] Carbendazim	36.95 (35.45 - 38.45)	36.95 (± 1.08)	36.45 (35.45 - 37.45)	36.45 (± 1)	38.45 (38.45 - 39.45)	38.85 (± 0.55)

Target organ	Toxin	24 h post dose		48 h post dose		168 h post dose	
		Median (min - max)	Mean (± SD)	Median (min - max)	Mean (± SD)	Median (min - max)	Mean (± SD)
Testicular	[D] Di-n-pentyl-phthalate	37.57 (36.38 - 40.15)	38.02 (± 1.28)	36.63 (35.07 - 38.41)	36.59 (± 1.23)	36.57 (34.98 - 37.99)	36.52 (± 1.09)
Testicular	[D] Ethane dimethane sulfonate (EDS)	34.95 (33.45 - 38.45)	35.05 (± 1.43)	37.45 (35.45 - 39.45)	37.25 (± 1.48)	38.45 (35.45 - 40.45)	38.45 (± 1.87)
Testicular	[D] Methoxyacetic acid	37.37 (35.29 - 38.7)	37.27 (± 1.12)	37.79 (35.24 - 38.09)	36.98 (± 1.36)	38 (36.04 - 38.57)	37.66 (± 0.98)
Multiple organ	[B] Adriamycin	36.95 (35.45 - 38.45)	36.95 (± 1.08)	37.45 (34.45 - 38.45)	36.85 (± 1.52)	29.45 (26.45 - 35.45)	30.25 (± 3.27)
Multiple organ	[C] Amphotericin B	34.45 (28.95 - 38.95)	34.05 (± 3.14)	34.95 (30.95 - 38.09)	34.78 (± 2.9)	33.95 (29.95 - 35.95)	33.15 (± 2.28)
Multiple organ	[C] Azaserine	38.45 (36.45 - 40.45)	38.45 (± 1.15)	36.45 (33.45 - 38.45)	36.05 (± 2.07)	37.45 (37.45 - 40.45)	38.25 (± 1.3)
Multiple organ	[A] Dexamethasone	45.45 (41.95 - 47.95)	45.05 (± 2.64)	44.95 (39.95 - 47.95)	44.09 (± 3.25)	44.95 (37.95 - 47.95)	43.25 (± 4.74)
Multiple organ	[E] Mitomycin-C	39.95 (37.45 - 43.45)	40.35 (± 1.66)	35.45 (34.45 - 36.45)	35.45 (± 1)	34.45 (33.45 - 34.45)	34.05 (± 0.55)
Physiological stressor	[C] 1,1-Dichloroethylene & maleic acid	37.45 (33.95 - 40.95)	37.45 (± 2.12)	35.95 (32.95 - 38.95)	36.15 (± 2.17)	37.95 (36.95 - 38.95)	38.15 (± 0.84)
Physiological stressor	[C] 2,4-Dinitrophenol	37.95 (34.25 - 39.85)	37.63 (± 1.51)	35.75 (33.55 - 37.95)	35.65 (± 1.61)	37.55 (35.15 - 39.85)	37.73 (± 1.74)
Physiological stressor	[B] 4-Pentenoic acid	39.25 (36.95 - 39.95)	38.91 (± 1.22)	37.31 (33.95 - 37.95)	36.82 (± 1.66)	37.95 (35.95 - 38.95)	37.95 (± 1.22)
Physiological stressor	[D] Acetazolamide	37.45 (34.45 - 41.45)	37.55 (± 1.91)	37.45 (34.45 - 40.45)	37.1 (± 2.32)	36.45 (35.45 - 38.45)	36.65 (± 1.3)
Physiological stressor	[C] Acivicin	37.95 (35.45 - 41.45)	38.25 (± 2.04)	37.45 (32.45 - 39.45)	36.45 (± 2.92)	38.45 (37.45 - 40.45)	38.85 (± 1.52)
Physiological stressor	[E] Ammonium chloride			37.45 (37.45 - 37.45)	37.45 (± 0)	37.45 (37.45 - 37.45)	37.45 (± 0)
Physiological stressor	[D] Carboplatin	37.45 (34.45 - 40.45)	37.35 (± 1.73)	38.45 (35.45 - 40.45)	38.25 (± 1.92)	37.45 (34.45 - 38.45)	36.85 (± 1.52)
Physiological stressor	[A] Choline and choline/methionine deficiency (chronic)	41.95 (38.45 - 47.45)	42.34 (± 2.88)	36.45 (36.45 - 39.45)	37.25 (± 1.3)	37.45 (35.45 - 39.45)	37.25 (± 1.79)
Physiological stressor	[B] Food restriction (chronic)	37.45 (34.45 - 38.45)	36.67 (± 1.3)	39.45 (36.45 - 40.45)	38.95 (± 1.91)	37.45 (37.45 - 40.45)	38.45 (± 1.41)
Physiological stressor	[D] Furosemide	35.95 (34.45 - 37.45)	36.09 (± 1.19)	38.45 (37.45 - 39.45)	38.45 (± 1)	36.45 (34.45 - 39.45)	36.65 (± 1.92)
Physiological stressor	[B] Insulin	36.95 (35.95 - 38.95)	37.15 (± 0.92)	36.95 (35.95 - 38.95)	37.35 (± 1.14)	38.95 (38.95 - 39.95)	39.35 (± 0.55)
Physiological stressor	[E] Methotrexate	38.95 (35.95 - 39.95)	38.55 (± 1.17)	36.95 (34.95 - 36.95)	36.55 (± 0.89)	34.95 (34.95 - 34.95)	34.95 (± 0)
Physiological stressor	[A] Partial hepatectomy	32.45 (29.45 - 34.45)	32.45 (± 1.63)	28.45 (26.45 - 30.45)	28.65 (± 1.48)	30.45 (25.45 - 31.45)	29.05 (± 2.51)

Target organ	Toxin	24 h post dose		48 h post dose		168 h post dose	
		Median (min - max)	Mean (± SD)	Median (min - max)	Mean (± SD)	Median (min - max)	Mean (± SD)
Physiological stressor	[A] Phenobarbital (chronic)	38.45 (36.45 - 40.45)	38.35 (± 1.52)	37.45 (36.45 - 40.45)	37.65 (± 1.64)	40.45 (40.45 - 43.45)	41.25 (± 1.3)
Physiological stressor	[A] Pregnenolone 16 alpha carbonitrile (chronic)						
Physiological stressor	[A] Probenecid	37.3 (35.95 - 40.25)	37.51 (± 1.41)	36.75 (34.65 - 37.45)	36.41 (± 1.17)	37.45 (36.25 - 38.65)	37.35 (± 0.94)
Physiological stressor	[C] Rosiglitazone	38.95 (34.95 - 41.95)	38.55 (± 2.12)	36.95 (35.95 - 38.95)	36.95 (± 1.22)	36.95 (33.95 - 37.95)	35.95 (± 1.87)
Physiological stressor	[C] Rosiglitazone (chronic)	39.45 (37.45 - 41.45)	39.25 (± 1.32)				
Physiological stressor	[E] Sodium bicarbonate	37.03 (35.75 - 38.2)	37.01 (± 0.88)				
Physiological stressor	[A] Unilateral nephrectomy	35.45 (33.45 - 37.45)	35.15 (± 1.06)	33.45 (30.45 - 36.45)	33.25 (± 2.39)	36.45 (35.45 - 38.45)	36.65 (± 1.3)
Physiological stressor	[B] Water deprivation (chronic)	41.45 (37.55 - 43.45)	41.04 (± 1.72)	41.95 (37.69 - 43.45)	41.49 (± 2.2)	36.82 (35.45 - 39.45)	37.24 (± 1.47)
No Effect	[E] Acetaminophen (chronic)	37.45 (37.45 - 39.45)	37.85 (± 0.89)			37.45 (36.45 - 38.45)	37.25 (± 0.84)
No Effect	[C] Buthionine sulphoxime	39.45 (37.45 - 41.45)	39.15 (± 1.42)	39.45 (37.45 - 40.45)	39.25 (± 1.1)	39.45 (38.45 - 40.45)	39.45 (± 1)
No Effect	[C] Ferrous sulphate	34.65 (30.35 - 41.45)	35.08 (± 3.33)	34.25 (30.55 - 36.55)	33.81 (± 2.2)	36.75 (34.25 - 37.55)	36.37 (± 1.26)
No Effect	[B] Ifosfamide	37.45 (35.45 - 39.45)	37.65 (± 1.23)	35.45 (33.45 - 36.45)	35.25 (± 1.1)	37.45 (35.45 - 37.45)	36.85 (± 0.89)
No Effect	[B] Lithocholic acid	38.95 (36.45 - 42.45)	38.85 (± 1.65)	35.45 (32.45 - 36.45)	35.05 (± 1.67)	38.45 (36.45 - 40.45)	38.45 (± 1.58)
No Effect	[E] Paraquat			38.45 (37.45 - 39.45)	38.25 (± 0.84)	37.45 (37.45 - 39.45)	38.25 (± 1.1)
No Effect	[D] Potassium dichromate	34.45 (24.45 - 36.45)	31.95 (± 4.62)	36.45 (35.45 - 36.45)	36.25 (± 0.45)	37.45 (35.45 - 41.45)	37.85 (± 2.19)
No Effect	[C] Trichlorethylene	40.65 (35.8 - 42)	39.62 (± 2.23)	40.6 (36.5 - 41.2)	39.18 (± 2.41)	40.6 (39.1 - 41.7)	40.54 (± 0.93)

A-F: Indicates Pharmaceutical Company & sample origin

Supplementary Table S26. Summary statistics for serum total protein (g/L) at 24 hrs, 48 hrs and 168 hrs post high dose.

Target organ	Toxin	24 h post dose		48 h post dose		168 h post dose	
		Median (min - max)	Mean (± SD)	Median (min - max)	Mean (± SD)	Median (min - max)	Mean (± SD)
Liver	[E] 1,1-Dichloroethylene	67 (64.5 - 69.5)	67 (± 1.72)	54.5 (52.5 - 58.5)	54.5 (± 2.45)	53.5 (50.5 - 54.5)	52.9 (± 1.52)
Liver	[E] 1,2,3,4,5,6-hexachlorocyclohexane	65.5 (62 - 68)	65.3 (± 2.06)	57 (55 - 59)	57 (± 1.58)	60 (59 - 60)	59.6 (± 0.55)
Liver	[B] 1-Fluoropentane	61.5 (57 - 65)	61.5 (± 2.27)	58 (57 - 62)	59.2 (± 2.17)	59 (57 - 61)	58.8 (± 1.48)
Liver	[B] 2,4,6-Trihydroxyacetophenone (THA)	58.5 (55.5 - 61.5)	58.3 (± 1.87)	58.5 (56.5 - 61.5)	59.1 (± 1.95)	55.5 (52.5 - 57.5)	55.3 (± 1.92)
Liver	[B] 4-Amino-2,6-dichlorophenol (ADCP)	60.9 (58.4 - 66.4)	60.9 (± 2.42)	61.4 (60.4 - 63.4)	61.8 (± 1.14)	57.4 (55.4 - 60.4)	57.8 (± 1.82)
Liver	[C] Aflatoxin	56.65 (53.35 - 60.65)	56.97 (± 2.24)	51.25 (49.15 - 52.85)	51.33 (± 1.45)	52.65 (46.45 - 62.35)	53.09 (± 6.52)
Liver	[C] Allyl alcohol	54.6 (49.1 - 68.6)	55.5 (± 5.03)	55.2 (53.8 - 56.1)	54.9 (± 0.97)	59.2 (56.7 - 61.3)	58.88 (± 1.79)
Liver	[C] Allyl formate	57.25 (48.5 - 60.94)	56.63 (± 3.68)	58.89 (58.07 - 59.56)	58.77 (± 0.6)	59.8 (58.6 - 61.94)	59.97 (± 1.27)
Liver	[B] Azathioprine	63.9 (61.4 - 65.4)	63.6 (± 1.62)	55.4 (52.4 - 61.4)	56.6 (± 3.7)	63.4 (60.4 - 64.4)	62.6 (± 1.64)
Liver	[B] Bromobenzene	65 (57.5 - 67.5)	64.2 (± 3.4)	57.5 (52.5 - 58.5)	55.9 (± 2.7)	65.5 (64.5 - 66.5)	65.5 (± 0.71)
Liver	[C] Butylated hydroxytoluene	62.05 (60.6 - 65.1)	62.69 (± 1.91)	54.9 (52.9 - 57.1)	54.96 (± 1.98)	61.7 (59.5 - 66.9)	61.96 (± 2.97)
Liver	[D] Carbon tetrachloride	60.52 (59.08 - 61.92)	60.45 (± 0.91)	54 (49 - 59)	54 (± 3.61)	62 (58 - 67)	62 (± 3.67)
Liver	[C] Chlorpromazine	61.15 (36.55 - 68.25)	59.36 (± 9.25)	61.05 (58.15 - 64.85)	61.01 (± 2.55)	61.35 (55.55 - 62.85)	59.97 (± 2.98)
Liver	[B] Clofibrate	59 (58 - 63)	59.8 (± 1.93)	56 (54 - 59)	56.4 (± 2.07)	61 (59 - 63)	60.8 (± 1.48)
Liver	[B] Cyproterone acetate	65 (60 - 67)	64.3 (± 2.31)	55 (54 - 60)	56.2 (± 2.68)	65 (59 - 69)	65 (± 3.94)
Liver	[A] D-galactosamine	59.18 (56.7 - 62.22)	59.24 (± 1.87)	46 (36 - 51)	44.4 (± 5.5)	59 (56 - 63)	59.4 (± 3.05)
Liver	[B] Diethylhexylphthalate (DEHP)			65 (63 - 66)	64.6 (± 1.14)	65 (62 - 65)	64.2 (± 1.3)
Liver	[C] Dimethylformamide (DMF)	59.15 (55.8 - 61.4)	58.79 (± 1.77)	57.1 (55.9 - 58.8)	57.1 (± 1.21)	61.7 (58.4 - 65.3)	61.72 (± 2.54)
Liver	[C] Dimethylnitrosamine (DMN)	53.15 (50.55 - 58.25)	53.59 (± 2.73)	42.75 (40.25 - 46.75)	43.41 (± 2.56)	59.15 (56.35 - 60.75)	58.93 (± 1.76)
Liver	[A] Gadolinium chloride	58 (55 - 62)	58.4 (± 2.17)	57 (54 - 60)	57.6 (± 2.51)	61 (58 - 63)	60.8 (± 1.92)

Target organ	Toxin	24 h post dose		48 h post dose		168 h post dose	
		Median (min - max)	Mean (± SD)	Median (min - max)	Mean (± SD)	Median (min - max)	Mean (± SD)
Liver	[A, B, C, D, F] Hydrazine	55 (51.5 - 59.4)	55.26 (± 1.87)	53.5 (47.6 - 58)	53.18 (± 3.01)	59.46 (39 - 69)	58.21 (± 7.38)
Liver	[E] Hydrazine	59.3 (58.85 - 59.55)	59.26 (± 0.21)	58.1 (57.95 - 58.45)	58.15 (± 0.22)	57.25 (56.25 - 58.75)	57.23 (± 1.02)
Liver	[E] Indomethacin	37 (33 - 41)	36.5 (± 2.68)	41 (36 - 61)	44 (± 9.85)	39 (38 - 61.17)	43.83 (± 9.81)
Liver	[E] Ketoconazole			57.5 (55.5 - 59.5)	57.5 (± 1.58)	57.5 (54.5 - 58.5)	56.7 (± 1.64)
Liver	[C] Lead acetate	60.7 (55.45 - 76.65)	62.23 (± 6.13)	56.05 (54.65 - 67.15)	58.67 (± 5.22)	59.35 (57.35 - 67.55)	61.27 (± 4.13)
Liver	[A] Lipopolysaccharide (LPS)	53 (47 - 59.24)	52.72 (± 3.64)	51 (48 - 58.98)	51.6 (± 4.33)	60 (60 - 62)	60.4 (± 0.89)
Liver	[B] Methapyrilene	56.5 (52.5 - 59.5)	56.1 (± 2.37)	54.5 (51.5 - 59.5)	55.5 (± 3.08)	57.5 (55.5 - 59.5)	57.5 (± 1.58)
Liver	[E] Methylene dianiline	63.5 (59.5 - 72.5)	63.9 (± 4.03)	55.5 (52.5 - 59.5)	56.3 (± 2.77)	59.5 (55.5 - 62.5)	59.3 (± 2.86)
Liver	[C] Monocrotaline	57.25 (51.65 - 58.55)	56.88 (± 1.95)	56.15 (53.45 - 58.15)	55.93 (± 1.79)	63.35 (61.95 - 67.35)	63.93 (± 2.3)
Liver	[C] N-methylformamide (NMF)	60.6 (58.25 - 63.05)	60.72 (± 1.42)	55.55 (54.45 - 56.95)	55.57 (± 1.02)	61.95 (59.25 - 63.45)	61.47 (± 1.67)
Liver	[D] Phalloidin (chronic)			60.28 (58.62 - 65.83)	60.81 (± 2.9)	60.5 (59.11 - 68.17)	62.3 (± 3.61)
Liver	[E] Phenyl diisothiocyanate	65.5 (60.5 - 75.5)	66.1 (± 4.53)	56.5 (50.5 - 68.5)	58.5 (± 7.75)	60.47 (53.5 - 61.17)	58.75 (± 3.3)
Liver	[E] Phenyl isothiocyanate	64 (58 - 71)	63.4 (± 3.84)	59 (54 - 60)	58.2 (± 2.49)	56 (54 - 57)	55.8 (± 1.3)
Liver	[B] Retinyl palmitate	62.4 (59.4 - 64.4)	62.1 (± 1.49)	58.4 (55.4 - 59.4)	57.8 (± 1.82)	62.4 (60.4 - 65.4)	62.6 (± 1.92)
Liver	[B] Sodium Valproate			56 (51 - 59.23)	54.65 (± 3.58)	57 (57 - 60.67)	57.93 (± 1.59)
Liver	[C] a-Naphthylisothiocyanate (ANIT)	59.4 (56.85 - 64.15)	59.9 (± 2.52)	57.95 (52.35 - 63.15)	57.43 (± 4.11)	60.55 (59.85 - 64.45)	61.63 (± 2.05)
Kidney	[D] 2-Bromophenol	58 (54 - 59)	57.1 (± 1.6)	60 (56 - 65)	60.6 (± 3.51)	62 (61 - 69)	63.2 (± 3.35)
Kidney	[E] 3,5-Dichloroaniline hydrochloride	59.5 (53.5 - 64.5)	59.4 (± 3.03)	51.5 (49.5 - 53.5)	51.7 (± 1.48)	54.5 (50.5 - 57.5)	54.3 (± 2.49)
Kidney	[E] Atractyloside	64.5 (52.5 - 68.5)	62.78 (± 4.9)	55.5 (50.5 - 59.5)	55.3 (± 3.27)	54.5 (53.5 - 58.5)	55.5 (± 2)
Kidney	[D] Bromoethylamine hydrobromide	60.02 (57 - 63)	60.12 (± 1.99)	56 (49 - 61.56)	56.11 (± 4.74)	59 (55 - 61)	58.84 (± 2.31)

Target organ	Toxin	24 h post dose		48 h post dose		168 h post dose	
		Median (min - max)	Mean (± SD)	Median (min - max)	Mean (± SD)	Median (min - max)	Mean (± SD)
Kidney	[D] Cephaloridine	57.5 (56 - 64)	58.9 (± 3)	58 (57 - 61)	58.6 (± 1.52)	63 (61 - 68)	63.6 (± 2.7)
Kidney	[B] Chlorethanamine	65.5 (59.5 - 67.5)	64.6 (± 2.38)	55.5 (54.5 - 57.5)	56.1 (± 1.34)	59.5 (59.5 - 62.5)	60.3 (± 1.3)
Kidney	[A] Cisplatin	58 (55.5 - 61.5)	58.36 (± 1.81)	58.5 (57.5 - 61.5)	59.1 (± 1.52)	61.15 (56.5 - 62.5)	60.23 (± 2.35)
Kidney	[A] D-limonene (chronic)						
Kidney	[E] Dichlorophenyl succinimide	65.5 (62.5 - 70.5)	65.7 (± 2.97)	57.5 (55.5 - 57.5)	56.9 (± 0.89)	60.5 (56.5 - 61.5)	59.5 (± 2.35)
Kidney	[D] Ethylene glycol	58 (55 - 63)	58.4 (± 2.27)	61 (58 - 64)	61 (± 2.24)	61 (61 - 64)	62 (± 1.41)
Kidney	[A] Folic acid	58.5 (56 - 60)	58 (± 1.63)	65 (58.64 - 72)	65.27 (± 4.82)	64 (60.1 - 65)	62.97 (± 2.37)
Kidney	[A] Gentamicin	58 (56 - 63)	58.6 (± 2.32)	61 (59 - 67)	61.8 (± 3.03)	66 (65 - 71)	67 (± 2.35)
Kidney	[B] Maleic acid	63.9 (59.4 - 66.4)	63.5 (± 2.38)	62.4 (59.4 - 64.4)	62 (± 2.51)	64.4 (62.4 - 66.4)	64.4 (± 1.41)
Kidney	[A] N-phenylanthranilic acid (chronic)	58 (55 - 60.88)	58.13 (± 2.07)	61 (54 - 99)	66.6 (± 18.34)	59 (51 - 64)	58.02 (± 4.86)
Kidney	[D] Para-aminophenol	53 (46 - 57)	52.9 (± 3.07)	58 (52 - 61)	57.2 (± 3.42)	63 (61 - 66)	62.8 (± 2.05)
Kidney	[A] Puromycin			57 (55 - 58)	56.8 (± 1.1)	57 (52 - 58)	55.6 (± 2.51)
Kidney	[B] Vancomycin hydrochloride			59 (57 - 63)	59.8 (± 2.28)	58 (57 - 62)	59 (± 2)
Liver & Kidney	[E] Acetaminophen	67 (64.5 - 73.5)	68.1 (± 3.06)	52.5 (47.5 - 59.5)	53.3 (± 4.49)	59.5 (57.5 - 61.5)	59.7 (± 1.79)
Liver & Kidney	[B] Aurothiomalate	69.9 (62.4 - 75.4)	68.5 (± 4.77)	62.4 (60.4 - 65.4)	63 (± 2.3)	61.4 (56.4 - 68.4)	62 (± 5.32)
Liver & Kidney	[C] Chloroform	63.85 (58.85 - 69.15)	63.8 (± 3.26)	57.85 (51.65 - 63.95)	57.21 (± 5.2)	61.35 (59.65 - 63.35)	61.65 (± 1.48)
Liver & Kidney	[D] Cyclosporin	59 (57 - 63)	58.83 (± 1.84)	52 (46 - 60.62)	52.44 (± 4.72)	58 (55 - 62)	58.5 (± 2.78)
Liver & Kidney	[D] Dichlorobenzene	57 (51.5 - 59.5)	56 (± 3.03)	55.5 (51.5 - 59.5)	55.7 (± 3.03)	67.5 (61.5 - 70.5)	66.1 (± 3.91)
Liver & Kidney	[C] Ethionine	61.15 (57.55 - 63.15)	61.02 (± 1.83)	52.75 (50.45 - 54.75)	52.37 (± 1.78)	60.15 (55.85 - 61.55)	59.23 (± 2.34)
Liver & Kidney	[B] Hexachlorobutadiene (HCBD)			56.5 (54.5 - 58.5)	56.3 (± 1.48)	61.5 (53.5 - 64.5)	60.1 (± 4.1)

Target organ	Toxin	24 h post dose		48 h post dose		168 h post dose	
		Median (min - max)	Mean (± SD)	Median (min - max)	Mean (± SD)	Median (min - max)	Mean (± SD)
Liver & Kidney	[B] Mercuric chloride	60.5 (56.5 - 63.5)	60.3 (± 2.04)	61.5 (54.5 - 69.5)	61.7 (± 5.36)	60.67 (58.5 - 63.5)	60.73 (± 1.93)
Liver & Kidney	[E] Microcystin-LR			51.5 (49.5 - 60.67)	53.33 (± 4.78)	59.5 (56.5 - 60.5)	58.89 (± 1.81)
Liver & Kidney	[E] Rotenone	62.84 (59.87 - 75)	65 (± 5.42)	63.69 (59 - 75)	65.34 (± 7.32)	57.24 (50 - 61.17)	56.63 (± 4.89)
Liver & Kidney	[E] S-(1,2-dichlorovinyl)-cysteine (DCVC)	61 (59 - 77)	63.3 (± 5.77)	55 (54 - 56)	55 (± 1)	57 (54 - 59)	56.2 (± 2.17)
Liver & Kidney	[D] Thioacetamide	56.9 (54 - 58.71)	56.56 (± 1.49)	57 (49 - 58.8)	56.01 (± 4)	64 (60 - 65)	63.4 (± 2.07)
Pancreas	[E] 1-Cyano-2-hydroxy-3-butene	64 (5 - 77)	60.21 (± 20.27)	59 (52 - 60.67)	57.73 (± 3.49)	52 (48 - 55)	51.6 (± 3.05)
Pancreas	[C] Caerulin	58.35 (55.5 - 64.2)	58.65 (± 2.77)	56.9 (53.3 - 60.6)	56.7 (± 2.7)	61.52 (59.7 - 63.3)	61.47 (± 1.37)
Pancreas	[E] L-arginine	69 (48 - 77)	66.36 (± 8.12)	56 (54 - 61)	57.15 (± 2.27)	51 (48 - 58)	51.9 (± 3.31)
Pancreas	[B] Streptozotocin	59.5 (55 - 64)	59.4 (± 2.72)	58 (57 - 62)	59 (± 2)	55 (52 - 60)	55.6 (± 3.05)
Testicular	[D] 1,3-Dinitrobenzene	53 (50 - 57)	53.6 (± 2.01)	51 (49 - 58)	52.2 (± 3.42)	60 (58 - 63)	60.6 (± 2.3)
Testicular	[C] Cadmium chloride	54.6 (48.55 - 61.05)	55.22 (± 4.03)	58.55 (51.45 - 60.25)	57.29 (± 3.58)	59.45 (57.25 - 64.35)	59.91 (± 2.69)
Testicular	[D] Cadmium chloride	51.97 (47.39 - 57.86)	52.7 (± 3.24)	57.3 (48.06 - 61.69)	54.89 (± 5.84)	58.02 (57.3 - 61.09)	58.42 (± 1.55)
Testicular	[D] Carbendazim	58.5 (54 - 60)	57.8 (± 1.81)	58 (55 - 60)	57.6 (± 2.07)	63 (62 - 67)	63.8 (± 1.92)
Testicular	[D] Di-n-pentyl-phthalate	59.67 (56.65 - 62.18)	59.49 (± 1.88)	53.78 (53.17 - 56.66)	54.48 (± 1.42)	59.04 (58.26 - 62.49)	59.59 (± 1.69)
Testicular	[D] Ethane dimethane sulfonate (EDS)	53.5 (52 - 55)	53.6 (± 0.97)	56 (55 - 59)	56.6 (± 1.52)	65 (59 - 66)	64 (± 2.83)
Testicular	[D] Methoxyacetic acid	57.97 (56.44 - 62.47)	58.42 (± 1.92)	56.42 (56 - 60.71)	57.33 (± 1.94)	60.21 (54.26 - 61.72)	59.49 (± 3.02)
Multiple organ	[B] Adriamycin	57.5 (54 - 63)	57.4 (± 2.5)	55 (54 - 60)	56.2 (± 2.39)	53 (50 - 57)	53.2 (± 2.59)
Multiple organ	[C] Amphotericin B	61.75 (57.7 - 74.4)	62.73 (± 5.08)	60.6 (60.1 - 67.7)	62.62 (± 3.32)	57.2 (52.9 - 60)	56.74 (± 3.01)
Multiple organ	[C] Azaserine	55.5 (51.9 - 60)	56.16 (± 2.43)	52.4 (50 - 58)	53.4 (± 3.12)	58.2 (57.9 - 61.3)	58.8 (± 1.41)
Multiple organ	[A] Dexamethasone	68.5 (66 - 73)	68.9 (± 2.18)	69 (63 - 77)	68.8 (± 5.67)	68 (55 - 72)	65 (± 6.9)
Multiple organ	[E] Mitomycin-C	64 (60 - 67)	63.7 (± 2.41)	54 (53 - 55)	54 (± 0.71)	53 (51 - 54)	52.6 (± 1.14)

Target organ	Toxin	24 h post dose		48 h post dose		168 h post dose	
		Median (min - max)	Mean (± SD)	Median (min - max)	Mean (± SD)	Median (min - max)	Mean (± SD)
Physiological stressor	[C] 1,1-Dichloroethylene & maleic acid	60.65 (57.7 - 66.2)	60.76 (± 2.29)	58.3 (57.1 - 59.9)	58.52 (± 1.07)	62.4 (60.5 - 63.2)	62.1 (± 1.21)
Physiological stressor	[C] 2,4-Dinitrophenol	60.1 (56.1 - 62.5)	59.81 (± 1.94)	57.2 (53.6 - 59.1)	56.98 (± 2.09)	60.4 (55.9 - 61.6)	58.94 (± 2.76)
Physiological stressor	[B] 4-Pentenoic acid	59.5 (55 - 62)	58.6 (± 3.03)	58 (55 - 60)	58.05 (± 1.9)	57 (55 - 63)	58.2 (± 3.63)
Physiological stressor	[D] Acetazolamide	59.63 (58 - 63)	60.03 (± 1.7)	56 (54 - 60.62)	56.92 (± 2.51)	58 (57 - 61)	58.8 (± 1.64)
Physiological stressor	[C] Acivicin	61.15 (57.5 - 63.6)	60.96 (± 2.09)	59.2 (54.5 - 61.4)	58.46 (± 3.14)	60.5 (58.6 - 63.6)	61.08 (± 2.23)
Physiological stressor	[E] Ammonium chloride			59 (58.99 - 59)	59 (± 0)	59 (59 - 59)	59 (± 0)
Physiological stressor	[D] Carboplatin	58.5 (56.5 - 63.5)	59.1 (± 2.07)	59.5 (58.5 - 61.5)	59.9 (± 1.14)	58.5 (55.5 - 62.5)	58.9 (± 3.05)
Physiological stressor	[A] Choline and choline/methionine deficiency (chronic)	67 (61 - 85)	70.4 (± 8.34)	59 (57 - 62)	59.4 (± 2.07)	61 (56 - 64)	59.8 (± 3.27)
Physiological stressor	[B] Food restriction (chronic)	55 (52 - 59)	55.56 (± 2.46)	60.5 (59 - 65)	61.25 (± 2.63)	58 (58 - 60)	58.6 (± 0.89)
Physiological stressor	[D] Furosemide	55.5 (54.5 - 57.85)	55.74 (± 1.11)	57.5 (57.5 - 62.5)	58.7 (± 2.17)	59.5 (57.5 - 64.5)	59.9 (± 2.7)
Physiological stressor	[B] Insulin	58.5 (57 - 60)	58.6 (± 0.97)	59 (57 - 59)	58.2 (± 1.1)	61 (58 - 62)	60.6 (± 1.52)
Physiological stressor	[E] Methotrexate	61.5 (55.5 - 62.5)	60.3 (± 2.53)	55.5 (54.5 - 57.5)	55.7 (± 1.1)	50.5 (50.5 - 52.5)	51.1 (± 0.89)
Physiological stressor	[A] Partial hepatectomy	51 (48 - 53)	50.7 (± 1.49)	47 (45 - 48)	46.6 (± 1.14)	54 (52 - 57)	54.2 (± 1.92)
Physiological stressor	[A] Phenobarbital (chronic)	56.5 (53 - 61)	56.5 (± 2.27)	57 (55 - 70)	59.4 (± 6.11)	62 (60 - 67)	62.4 (± 2.7)
Physiological stressor	[A] Pregnenolone 16 alpha carbonitrile (chronic)						
Physiological stressor	[A] Probenecid	59.3 (56.6 - 65.7)	59.98 (± 2.56)	57.2 (54.3 - 59.8)	57.2 (± 2.56)	57.2 (55.4 - 61.8)	57.98 (± 2.46)
Physiological stressor	[C] Rosiglitazone	59.95 (57.45 - 62.95)	60.2 (± 1.47)	55.85 (54.25 - 56.85)	55.67 (± 1.04)	57.35 (54.65 - 60.15)	57.43 (± 1.95)
Physiological stressor	[C] Rosiglitazone (chronic)	59.5 (55.4 - 63.3)	59.57 (± 2.18)				
Physiological stressor	[E] Sodium bicarbonate	60.35 (55.47 - 61.32)	60.04 (± 1.68)				
Physiological stressor	[A] Unilateral nephrectomy	59 (56 - 62)	58.8 (± 2.04)	54 (50 - 61)	54.4 (± 4.39)	56 (55 - 60)	57.2 (± 2.17)

Target organ	Toxin	24 h post dose		48 h post dose		168 h post dose	
		Median (min - max)	Mean (± SD)	Median (min - max)	Mean (± SD)	Median (min - max)	Mean (± SD)
Physiological stressor	B Water deprivation (chronic)	65 (59.36 - 67.5)	63.91 (± 2.44)	67 (60.52 - 70.5)	67 (± 3.67)	59.33 (57.5 - 61.5)	59.44 (± 1.51)
No Effect	E Acetaminophen (chronic)	58.5 (58.5 - 63.5)	59.7 (± 2.17)			60.5 (57.5 - 60.5)	59.3 (± 1.64)
No Effect	C Buthionine sulphoxime	61.6 (57.9 - 63.7)	61.16 (± 1.8)	58.8 (57.1 - 59.5)	58.52 (± 0.9)	60.6 (57.8 - 62.5)	60.4 (± 1.78)
No Effect	C Ferrous sulphate	55.1 (49.5 - 64)	55.71 (± 4.05)	56.6 (51.3 - 58.7)	55.52 (± 2.85)	59.1 (56.4 - 60)	58.8 (± 1.46)
No Effect	B Ifosfamide	58.5 (56 - 63)	58.7 (± 2)	54 (52 - 56)	54 (± 1.58)	58 (53 - 60)	57 (± 2.92)
No Effect	B Lithocholic acid	61.5 (58 - 66)	61.8 (± 2.74)	58 (55 - 62)	58.4 (± 2.7)	60 (58 - 63)	60.4 (± 1.82)
No Effect	E Paraquat			58.5 (58.5 - 61.5)	59.5 (± 1.41)	58.5 (57.5 - 60.5)	58.9 (± 1.14)
No Effect	D Potassium dichromate	56.5 (40 - 59)	52 (± 7.85)	58 (57 - 59)	57.8 (± 0.84)	58 (56 - 61)	58.2 (± 2.28)
No Effect	C Trichlorethylene	60.15 (57.35 - 63.25)	60.12 (± 1.88)	59.15 (58.35 - 60.15)	59.19 (± 0.77)	61.75 (59.85 - 64.85)	62.09 (± 1.88)

A-F: Indicates Pharmaceutical Company & sample origin

Supplementary Table S27. Summary statistics for serum total bilirubin (umol/L) at 24 hrs, 48 hrs and 168 hrs post high dose.

Target organ	Toxin	24 h post dose		48 h post dose		168 h post dose	
		Median (min - max)	Mean (± SD)	Median (min - max)	Mean (± SD)	Median (min - max)	Mean (± SD)
Liver	E 1,1-Dichloroethylene	2.91 (1.2 - 4.62)	2.4 (± 1.15)	2.91 (1.2 - 4.62)	2.91 (± 1.71)	2.31 (1.2 - 2.91)	2.22 (± 0.64)
Liver	E 1,2,3,4,5,6-hexachlorocyclohexane	2.91 (1.2 - 8.04)	2.87 (± 1.98)	1.84 (1.2 - 3.34)	1.93 (± 0.84)	1.2 (1.2 - 2.65)	1.66 (± 0.67)
Liver	B 1-Fluoropentane	1.71 (1.2 - 1.88)	1.68 (± 0.23)	2.06 (1.88 - 2.57)	2.16 (± 0.26)	1.2 (0.86 - 1.54)	1.2 (± 0.24)
Liver	B 2,4,6-Trihydroxyacetophenone (THA)	1.71 (1.37 - 2.06)	1.76 (± 0.24)	1.2 (1.03 - 1.54)	1.23 (± 0.22)	1.54 (0.86 - 1.71)	1.44 (± 0.35)
Liver	B 4-Amino-2,6-dichlorophenol (ADCP)	1.22 (0.79 - 1.99)	1.34 (± 0.4)	0.79 (0.62 - 1.48)	0.93 (± 0.33)	0.62 (0.28 - 0.79)	0.55 (± 0.2)
Liver	C Aflatoxin	2.3 (1 - 18)	4.6 (± 5.25)	61.7 (47.8 - 80.1)	64.12 (± 11.85)	2.5 (1 - 7.8)	3.88 (± 3.24)

Target organ	Toxin	24 h post dose		48 h post dose		168 h post dose	
		Median (min - max)	Mean (± SD)	Median (min - max)	Mean (± SD)	Median (min - max)	Mean (± SD)
Liver	[C] Allyl alcohol	2.45 (1.5 - 15.6)	4.5 (± 4.38)	2 (1.2 - 9.3)	3.46 (± 3.35)	1.9 (1.3 - 2.2)	1.82 (± 0.37)
Liver	[C] Allyl formate	1.77 (0.8 - 2.7)	1.86 (± 0.7)	1.2 (1.2 - 1.5)	1.28 (± 0.13)	1 (0.9 - 1.38)	1.06 (± 0.19)
Liver	[B] Azathioprine	2.59 (2.16 - 4.38)	2.86 (± 0.66)	2.33 (1.14 - 3.19)	2.26 (± 0.88)	0.62 (0.28 - 0.79)	0.62 (± 0.21)
Liver	[B] Bromobenzene	1.71 (1.2 - 2.4)	1.7 (± 0.46)	5.82 (3.77 - 6.33)	5.17 (± 1.22)	1.37 (1.37 - 2.06)	1.61 (± 0.33)
Liver	[C] Butylated hydroxytoluene	9.25 (6.7 - 12.3)	9.5 (± 1.8)	4.9 (3.1 - 7.9)	4.82 (± 1.97)	1.3 (1.2 - 1.8)	1.4 (± 0.25)
Liver	[D] Carbon tetrachloride	2.3 (1.87 - 11.43)	3.36 (± 2.89)	2.3 (1.2 - 3.2)	2.22 (± 0.72)	1.3 (1 - 1.94)	1.38 (± 0.37)
Liver	[C] Chlorpromazine	1.4 (0.9 - 2.3)	1.46 (± 0.41)	1.7 (1.2 - 2.8)	1.74 (± 0.65)	1.7 (1.5 - 2.8)	1.9 (± 0.54)
Liver	[B] Clofibrate	0.86 (0.35 - 1.54)	0.89 (± 0.39)	0.86 (0.18 - 1.4)	0.8 (± 0.47)	0.86 (0.69 - 1.2)	0.93 (± 0.19)
Liver	[B] Cyproterone acetate	3 (1.2 - 11.12)	4.04 (± 2.78)	7.7 (3.25 - 9.07)	6.98 (± 2.37)	1.37 (1.2 - 2.23)	1.54 (± 0.4)
Liver	[A] D-galactosamine	30.98 (2.63 - 58.09)	29.14 (± 18.55)	83.28 (6.33 - 96.96)	62.08 (± 41.83)	2.91 (1.2 - 2.91)	2.23 (± 0.94)
Liver	[B] Diethylhexylphthalate (DEHP)			0.77 (0.43 - 0.77)	0.67 (± 0.15)	1.11 (0.6 - 1.11)	0.94 (± 0.24)
Liver	[C] Dimethylformamide (DMF)	0.9 (0.7 - 1.5)	0.99 (± 0.3)	2.9 (1.6 - 4.7)	3.24 (± 1.23)	0.8 (0.7 - 1.9)	1.04 (± 0.51)
Liver	[C] Dimethylnitrosamine (DMN)	2.95 (2.3 - 4.7)	3.11 (± 0.71)	3.3 (2.3 - 4.5)	3.32 (± 0.87)	1.4 (0.9 - 1.8)	1.4 (± 0.37)
Liver	[A] Gadolinium chloride	1.2 (1.2 - 1.2)	1.2 (± 0)	1.2 (1.2 - 1.71)	1.37 (± 0.24)	1.2 (1.2 - 1.2)	1.2 (± 0)
Liver	[A, B, C, D, F] Hydrazine	1.42 (0.1 - 2.6)	1.5 (± 0.45)	1.47 (0.94 - 4.62)	1.85 (± 0.95)	1.26 (0.94 - 2.2)	1.39 (± 0.34)
Liver	[E] Hydrazine	1.3 (1 - 1.4)	1.26 (± 0.13)	1.1 (1 - 1.2)	1.1 (± 0.08)	1.1 (1 - 2)	1.26 (± 0.42)
Liver	[E] Indomethacin	2.75 (1.2 - 14.88)	4.27 (± 4.22)	2.02 (1.2 - 3.94)	2.25 (± 1.18)	1.39 (1.2 - 2.91)	1.75 (± 0.74)
Liver	[E] Ketoconazole			1.68 (1.2 - 1.88)	1.57 (± 0.28)	1.97 (1.2 - 2.91)	2.04 (± 0.86)
Liver	[C] Lead acetate	1.7 (0.65 - 3.05)	1.58 (± 0.68)	1.85 (0.85 - 4.15)	2.08 (± 1.26)	1.25 (0.85 - 1.25)	1.09 (± 0.22)
Liver	[A] Lipopolysaccharide (LPS)	1.63 (1.2 - 3.01)	1.74 (± 0.56)	1.2 (1.2 - 2.76)	1.51 (± 0.7)	1.2 (1.2 - 1.71)	1.3 (± 0.23)

Target organ	Toxin	24 h post dose		48 h post dose		168 h post dose	
		Median (min - max)	Mean (± SD)	Median (min - max)	Mean (± SD)	Median (min - max)	Mean (± SD)
Liver	[B] Methapyrilene	2.57 (1.71 - 22.24)	4.62 (± 6.25)	3.42 (1.03 - 4.62)	3.15 (± 1.52)	1.88 (1.71 - 4.45)	2.47 (± 1.16)
Liver	[E] Methylene dianiline	6.33 (2.91 - 35.4)	10.26 (± 10.29)	2.91 (1.2 - 3.13)	2.37 (± 0.87)	2.73 (1.2 - 6.33)	2.87 (± 2.1)
Liver	[C] Monocrotaline	2.1 (1.4 - 2.6)	2 (± 0.41)	0.9 (0.9 - 1.5)	1.04 (± 0.26)	1.2 (0.7 - 1.5)	1.18 (± 0.31)
Liver	[C] N-methylformamide (NMF)	1.8 (1.05 - 2.65)	1.83 (± 0.51)	1.85 (1.25 - 6.25)	2.83 (± 2.06)	1.05 (0.75 - 2.45)	1.45 (± 0.75)
Liver	[D] Phalloidin (chronic)			9 (8.03 - 11.05)	9.16 (± 1.15)	2.98 (1.74 - 4.76)	3.12 (± 1.1)
Liver	[E] Phenyl diisothiocyanate	3.15 (1.2 - 5.18)	3.15 (± 1)	2.91 (1.2 - 4.62)	2.91 (± 1.21)	2.48 (1.2 - 4.62)	2.6 (± 1.26)
Liver	[E] Phenyl isothiocyanate	4.62 (1.2 - 38.82)	7.53 (± 11.26)	1.2 (1.2 - 5.98)	2.5 (± 2.08)	1.53 (1.2 - 2.91)	1.95 (± 0.89)
Liver	[B] Retinyl palmitate	1.22 (0.28 - 1.48)	1.17 (± 0.36)	0.45 (0.28 - 1.14)	0.66 (± 0.44)	0.79 (0.28 - 0.96)	0.69 (± 0.26)
Liver	[B] Sodium Valproate			3.26 (0.34 - 6.3)	3.15 (± 2.14)	1.21 (0.86 - 1.88)	1.3 (± 0.37)
Liver	[C] a-Naphthylisothiocyanate (ANIT)	40.45 (7.4 - 61.5)	40.21 (± 14.58)	139.3 (108 - 272)	163.28 (± 64.7)	7 (5.9 - 9.4)	7.22 (± 1.3)
Kidney	[D] 2-Bromophenol	1.05 (0.8 - 1.24)	1.02 (± 0.15)	1.1 (0.8 - 1.56)	1.15 (± 0.32)	1.26 (0.9 - 1.87)	1.28 (± 0.36)
Kidney	[E] 3,5-Dichloroaniline hydrochloride	3.76 (0.34 - 5.48)	2.95 (± 1.69)	0.34 (0.34 - 2.05)	0.84 (± 0.76)	0.34 (0.34 - 2.57)	1.13 (± 1.09)
Kidney	[E] Atractyloside	2.91 (1.2 - 4.62)	2.67 (± 1.09)	1.2 (1.2 - 2.91)	1.88 (± 0.94)	1.2 (1.2 - 4.62)	1.96 (± 1.49)
Kidney	[D] Bromoethylamine hydrobromide	1.64 (1.05 - 2.75)	1.85 (± 0.57)	1.75 (0.85 - 1.92)	1.5 (± 0.48)	1.65 (0.95 - 1.71)	1.46 (± 0.32)
Kidney	[D] Cephaloridine	1.15 (0.8 - 2.2)	1.3 (± 0.5)	1.9 (0.9 - 2.1)	1.62 (± 0.5)	1.2 (0.9 - 1.6)	1.24 (± 0.27)
Kidney	[B] Chlorethanamine	0.6 (0.17 - 0.86)	0.58 (± 0.25)	1.03 (0.52 - 1.2)	0.89 (± 0.28)	2.23 (2.05 - 2.4)	2.26 (± 0.15)
Kidney	[A] Cisplatin	1.48 (1 - 4.8)	1.82 (± 1.09)	1.9 (1.1 - 2)	1.65 (± 0.4)	1.33 (0.7 - 2.1)	1.33 (± 0.51)
Kidney	[A] D-limonene (chronic)						
Kidney	[E] Dichlorophenyl succinimide	2.91 (1.2 - 4.62)	3.17 (± 1.28)	1.2 (1.2 - 2.91)	1.54 (± 0.76)	1.2 (1.2 - 2.91)	1.77 (± 0.8)
Kidney	[D] Ethylene glycol	1.05 (0 - 1.5)	0.74 (± 0.65)	1.5 (1 - 2.5)	1.52 (± 0.61)	1.45 (1.39 - 2)	1.55 (± 0.26)

Target organ	Toxin	24 h post dose		48 h post dose		168 h post dose	
		Median (min - max)	Mean (± SD)	Median (min - max)	Mean (± SD)	Median (min - max)	Mean (± SD)
Kidney	[A] Folic acid	5.65 (0.85 - 7.4)	4.56 (± 2.41)	4.2 (2.46 - 6.5)	4.41 (± 1.65)	1.26 (1 - 1.41)	1.21 (± 0.16)
Kidney	[A] Gentamicin	1.45 (1 - 2)	1.43 (± 0.31)	1.4 (1.1 - 2.37)	1.59 (± 0.51)	1.38 (1.1 - 1.6)	1.34 (± 0.23)
Kidney	[B] Maleic acid	0.62 (0.28 - 2.16)	0.86 (± 0.65)	0.62 (0.28 - 0.96)	0.66 (± 0.25)	1.14 (0.45 - 1.31)	0.96 (± 0.34)
Kidney	[A] N-phenylanthranilic acid (chronic)	1.2 (1.2 - 2.91)	1.64 (± 0.72)	1.2 (1.2 - 2.91)	1.54 (± 0.76)	1.2 (1.2 - 1.47)	1.25 (± 0.12)
Kidney	[D] Para-aminophenol	6.45 (1.2 - 9.6)	6.37 (± 2.27)	7.7 (3.2 - 10.6)	7.34 (± 2.72)	1.2 (1.09 - 1.9)	1.31 (± 0.34)
Kidney	[A] Puromycin			1.6 (1.3 - 2)	1.56 (± 0.29)	1.4 (1.2 - 1.93)	1.51 (± 0.3)
Kidney	[B] Vancomycin hydrochloride			1.2 (1.03 - 1.54)	1.23 (± 0.22)	1.03 (1.03 - 1.54)	1.17 (± 0.22)
Liver & Kidney	[E] Acetaminophen	7.19 (4.62 - 26.85)	10.09 (± 6.73)	6.33 (4.62 - 11.46)	6.67 (± 2.81)	2.14 (1.2 - 2.42)	1.9 (± 0.53)
Liver & Kidney	[B] Aurothiomalate	5.41 (0.28 - 8.49)	5.27 (± 2.2)	6.27 (2.85 - 7.98)	5.99 (± 1.95)	1.14 (0.96 - 1.31)	1.14 (± 0.12)
Liver & Kidney	[C] Chloroform	2.05 (1.65 - 3.35)	2.26 (± 0.58)	2.15 (0.35 - 2.55)	1.79 (± 0.87)	0.35 (0.35 - 0.95)	0.49 (± 0.26)
Liver & Kidney	[D] Cyclosporin	5.6 (1.6 - 7.1)	4.95 (± 1.91)	6.1 (1.47 - 9.4)	5.29 (± 3.09)	2.2 (1.74 - 2.4)	2.15 (± 0.25)
Liver & Kidney	[D] Dichlorobenzene	1.7 (1 - 2.5)	1.66 (± 0.44)	3.7 (3 - 4.8)	3.84 (± 0.69)	1.4 (0.9 - 2.5)	1.52 (± 0.59)
Liver & Kidney	[C] Ethionine	1.1 (0.5 - 1.8)	1.14 (± 0.46)	1.6 (1.2 - 1.8)	1.58 (± 0.23)	0.8 (0.5 - 1.3)	0.9 (± 0.31)
Liver & Kidney	[B] Hexachlorobutadiene (HCBD)			1.71 (1.03 - 2.06)	1.61 (± 0.41)	1.88 (1.71 - 2.06)	1.88 (± 0.12)
Liver & Kidney	[B] Mercuric chloride	1.37 (1.2 - 1.71)	1.44 (± 0.2)	1.89 (1.54 - 2.91)	1.99 (± 0.54)	1.03 (0.86 - 1.96)	1.18 (± 0.46)
Liver & Kidney	[E] Microcystin-LR			98.67 (2.16 - 119.19)	69.11 (± 57.46)	2.91 (2.63 - 8.04)	3.88 (± 2.33)
Liver & Kidney	[E] Rotenone	4.9 (3.06 - 25.14)	7.02 (± 6.52)	4.62 (2.33 - 8.04)	4.9 (± 2.35)	2.06 (1.2 - 2.63)	2.02 (± 0.52)
Liver & Kidney	[E] S-(1,2-dichlorovinyl)-cysteine (DCVC)	2.77 (1.2 - 6.33)	2.83 (± 1.62)	1.71 (1.2 - 2.91)	1.88 (± 0.75)	1.2 (1.2 - 2.91)	1.54 (± 0.76)
Liver & Kidney	[D] Thioacetamide	2.29 (1.4 - 5.14)	2.67 (± 1.05)	1.87 (1.5 - 3.1)	2.09 (± 0.62)	1.5 (1.19 - 1.9)	1.56 (± 0.29)
Pancreas	[E] 1-Cyano-2-hydroxy-3-butene	3.77 (1.2 - 23.43)	5.51 (± 6.76)	2.16 (1.2 - 8.04)	3.1 (± 2.85)	1.86 (1.2 - 7.09)	2.96 (± 2.39)

Target organ	Toxin	24 h post dose		48 h post dose		168 h post dose	
		Median (min - max)	Mean (± SD)	Median (min - max)	Mean (± SD)	Median (min - max)	Mean (± SD)
Pancreas	[C] Caerulin	2 (1.15 - 2.65)	1.89 (± 0.44)	1.05 (0.85 - 1.45)	1.13 (± 0.26)	0.95 (0.45 - 1.36)	0.91 (± 0.32)
Pancreas	[E] L-arginine	2.91 (1.2 - 8.04)	3.09 (± 1.88)	1.2 (1.2 - 2.41)	1.38 (± 0.41)	1.2 (1.2 - 4.62)	2.06 (± 1.21)
Pancreas	[B] Streptozotocin	0.78 (0.6 - 1.97)	0.98 (± 0.4)	1.28 (1.11 - 1.63)	1.32 (± 0.22)	0.95 (0.26 - 1.28)	0.84 (± 0.37)
Testicular	[D] 1,3-Dinitrobenzene	1.7 (1.14 - 3)	1.8 (± 0.51)	1.5 (1.2 - 1.7)	1.46 (± 0.21)	1.8 (1.1 - 2.4)	1.76 (± 0.47)
Testicular	[C] Cadmium chloride	1.45 (1.05 - 2.45)	1.59 (± 0.43)	1.75 (1.15 - 2.15)	1.73 (± 0.38)	1.95 (1.75 - 2.35)	2.07 (± 0.27)
Testicular	[D] Cadmium chloride	2.34 (1.93 - 2.6)	2.32 (± 0.2)	2.62 (1.96 - 2.85)	2.48 (± 0.34)	1.25 (0.8 - 1.5)	1.2 (± 0.26)
Testicular	[D] Carbendazim	1.16 (0.9 - 1.31)	1.14 (± 0.15)	1.3 (1.1 - 1.4)	1.25 (± 0.12)	1.52 (1.1 - 1.56)	1.36 (± 0.24)
Testicular	[D] Di-n-pentyl-phthalate	1.39 (0.85 - 1.71)	1.33 (± 0.29)	1.23 (1.14 - 1.38)	1.23 (± 0.1)	1.52 (1.16 - 2.46)	1.62 (± 0.53)
Testicular	[D] Ethane dimethane sulfonate (EDS)	1.61 (0.9 - 2.2)	1.6 (± 0.44)	1.23 (0.9 - 1.68)	1.2 (± 0.32)	1.2 (1.1 - 2.19)	1.42 (± 0.46)
Testicular	[D] Methoxyacetic acid	1.22 (0.7 - 1.83)	1.31 (± 0.38)	0.82 (0.7 - 1.28)	0.9 (± 0.24)	1.27 (0.86 - 1.35)	1.15 (± 0.24)
Multiple organ	[B] Adriamycin	0.6 (0.17 - 1.21)	0.59 (± 0.32)	0.17 (0.17 - 1.03)	0.41 (± 0.38)	0.34 (0.34 - 0.69)	0.48 (± 0.19)
Multiple organ	[C] Amphotericin B	2.65 (0.8 - 3.5)	2.34 (± 0.88)	2 (0.9 - 3.5)	2.21 (± 1.12)	0.7 (0.7 - 1)	0.8 (± 0.14)
Multiple organ	[C] Azaserine	2 (1.2 - 4)	2.28 (± 0.94)	3.3 (2.8 - 5)	3.6 (± 0.91)	2.6 (1.2 - 6.8)	3.18 (± 2.24)
Multiple organ	[A] Dexamethasone	1.85 (1.1 - 2.68)	1.91 (± 0.6)	4.53 (1.5 - 8.61)	4.53 (± 3.12)	1.2 (1.2 - 2.83)	1.58 (± 0.71)
Multiple organ	[E] Mitomycin-C	1.2 (1.2 - 6.33)	2.21 (± 1.65)	1.2 (1.2 - 2.91)	1.61 (± 0.74)	2.06 (1.61 - 2.91)	2.22 (± 0.65)
Physiological stressor	[C] 1,1-Dichloroethylene & maleic acid	1.05 (0.5 - 1.8)	1.11 (± 0.44)	1.1 (0.7 - 1.4)	1.08 (± 0.29)	1.2 (0.7 - 1.6)	1.18 (± 0.35)
Physiological stressor	[C] 2,4-Dinitrophenol	1.15 (0.8 - 1.5)	1.1 (± 0.24)	1.6 (0.9 - 2.2)	1.56 (± 0.56)	0.8 (0.8 - 1.5)	1.02 (± 0.32)
Physiological stressor	[B] 4-Pentenoic acid	1.2 (1.11 - 1.8)	1.32 (± 0.25)	1.97 (1.8 - 2.65)	2.14 (± 0.4)	1.11 (1.11 - 1.8)	1.29 (± 0.3)
Physiological stressor	[D] Acetazolamide	1.1 (0.75 - 2.9)	1.29 (± 0.64)	1.38 (0.45 - 1.95)	1.22 (± 0.58)	1.37 (0.75 - 2.08)	1.38 (± 0.48)
Physiological stressor	[C] Acivicin	1.2 (1.1 - 2.2)	1.34 (± 0.34)	1.7 (1.1 - 2.4)	1.68 (± 0.54)	1.3 (1.1 - 1.6)	1.3 (± 0.21)
Physiological stressor	[E] Ammonium chloride			1.2 (1.2 - 2.91)	1.54 (± 0.76)	1.2 (1.2 - 2.91)	1.88 (± 0.94)

Target organ	Toxin	24 h post dose		48 h post dose		168 h post dose	
		Median (min - max)	Mean (± SD)	Median (min - max)	Mean (± SD)	Median (min - max)	Mean (± SD)
Physiological stressor	[D] Carboplatin	1.55 (1.05 - 1.85)	1.45 (± 0.27)	1.25 (0.95 - 1.98)	1.34 (± 0.44)	1.45 (0.85 - 2.78)	1.56 (± 0.73)
Physiological stressor	[A] Choline and choline/methionine deficiency (chronic)	1.2 (1.2 - 3.24)	1.57 (± 0.79)	2.91 (1.2 - 2.91)	2.57 (± 0.76)	1.2 (1.2 - 1.2)	1.2 (± 0)
Physiological stressor	[B] Food restriction (chronic)	2.4 (1.03 - 2.74)	1.85 (± 0.78)	2.14 (1.88 - 2.91)	2.27 (± 0.45)	2.4 (2.23 - 2.74)	2.43 (± 0.19)
Physiological stressor	[D] Furosemide	1.25 (0.9 - 4.03)	1.54 (± 0.92)	1.3 (0.9 - 1.4)	1.24 (± 0.21)	1.3 (0.9 - 1.6)	1.28 (± 0.26)
Physiological stressor	[B] Insulin	1.03 (0.69 - 1.37)	1 (± 0.23)	0.52 (0.35 - 1.2)	0.69 (± 0.34)	0.52 (0.01 - 1.4)	0.52 (± 0.54)
Physiological stressor	[E] Methotrexate	2.91 (1.2 - 6.33)	3.08 (± 1.88)	1.2 (1.2 - 4.62)	2.23 (± 1.53)	1.2 (1.2 - 2.91)	1.88 (± 0.94)
Physiological stressor	[A] Partial hepatectomy	5.65 (3.08 - 14.03)	6.88 (± 3.83)	4.28 (3.25 - 4.62)	4.14 (± 0.57)	1.5 (1.2 - 2.23)	1.64 (± 0.4)
Physiological stressor	[A] Phenobarbital (chronic)	1.42 (0.9 - 1.89)	1.37 (± 0.33)	1.3 (1.2 - 2)	1.54 (± 0.42)	1.7 (1.2 - 1.9)	1.62 (± 0.26)
Physiological stressor	[A] Pregnenolone 16 alpha carbonitrile (chronic)						
Physiological stressor	[A] Probenecid	1.2 (1.2 - 1.2)	1.2 (± 0)	1.2 (1.2 - 1.2)	1.2 (± 0)	1.2 (1.2 - 2.91)	1.54 (± 0.76)
Physiological stressor	[C] Rosiglitazone	1.3 (0.8 - 2.2)	1.35 (± 0.49)	1.5 (0.9 - 2)	1.54 (± 0.42)	1 (0.8 - 2.1)	1.22 (± 0.55)
Physiological stressor	[C] Rosiglitazone (chronic)	1.7 (0.85 - 2.75)	1.63 (± 0.7)				
Physiological stressor	[E] Sodium bicarbonate	3.56 (2.73 - 5.18)	3.85 (± 0.84)				
Physiological stressor	[A] Unilateral nephrectomy	1.55 (0.69 - 2.25)	1.55 (± 0.41)	2.28 (1.37 - 2.98)	2.12 (± 0.72)	1.36 (1.17 - 2.91)	1.61 (± 0.73)
Physiological stressor	[B] Water deprivation (chronic)	1.71 (1.37 - 2.5)	1.73 (± 0.34)	1.97 (1.37 - 2.4)	1.94 (± 0.37)	1.36 (1.03 - 1.54)	1.31 (± 0.25)
No Effect	[E] Acetaminophen (chronic)	0.34 (0.34 - 3.76)	1.03 (± 1.53)			3.76 (0.34 - 5.47)	3.42 (± 1.87)
No Effect	[C] Buthionine sulphoxime	1.55 (0.95 - 1.85)	1.5 (± 0.28)	1.25 (0.75 - 1.85)	1.33 (± 0.41)	0.75 (0.75 - 1.45)	0.93 (± 0.3)
No Effect	[C] Ferrous sulphate	1.45 (0.8 - 3)	1.5 (± 0.68)	1.9 (1.2 - 2.1)	1.74 (± 0.38)	1.1 (0.8 - 1.3)	1.08 (± 0.19)
No Effect	[B] Ifosfamide	2.14 (1.37 - 3.25)	2.07 (± 0.57)	1.71 (1.37 - 2.06)	1.71 (± 0.27)	1.2 (1.03 - 1.37)	1.17 (± 0.14)
No Effect	[B] Lithocholic acid	1.2 (0.69 - 2.06)	1.29 (± 0.4)	1.2 (0.86 - 1.37)	1.2 (± 0.21)	1.03 (0.52 - 1.71)	1.03 (± 0.47)

Target organ	Toxin	24 h post dose		48 h post dose		168 h post dose	
		Median (min - max)	Mean (± SD)	Median (min - max)	Mean (± SD)	Median (min - max)	Mean (± SD)
No Effect	[E] Paraquat			1.5 (1.2 - 9.75)	3.31 (± 3.67)	1.2 (1.2 - 2.91)	1.54 (± 0.76)
No Effect	[D] Potassium dichromate	1.29 (0.85 - 1.85)	1.34 (± 0.36)	1.47 (1.05 - 1.63)	1.39 (± 0.23)	1.22 (1.05 - 1.75)	1.29 (± 0.27)
No Effect	[C] Trichlorethylene	1.7 (0.95 - 2.45)	1.65 (± 0.41)	0.55 (0.15 - 0.75)	0.49 (± 0.28)	0.75 (0.15 - 1.05)	0.69 (± 0.36)

A-F: Indicates Pharmaceutical Company & sample origin

Supplementary Table S28. Summary statistics for serum urea nitrogen (umol/L) at 24 hrs, 48 hrs and 168 hrs post high dose.

Target organ	Toxin	24 h post dose		48 h post dose		168 h post dose	
		Median (min - max)	Mean (± SD)	Median (min - max)	Mean (± SD)	Median (min - max)	Mean (± SD)
Liver	[E] 1,1-Dichloroethylene	3929 (2144 - 5357)	3964.7 (± 832.15)	3929 (2858 - 5714)	4071.8 (± 1145.74)	5714 (5000 - 6428)	5714 (± 714)
Liver	[E] 1,2,3,4,5,6-hexachlorocyclohexane	4821.5 (3572 - 6071)	4821.5 (± 811.47)	4643 (2858 - 5357)	4143.2 (± 1059.03)	5714 (5357 - 6428)	5785.4 (± 465.47)
Liver	[B] 1-Fluoropentane	11010 (11010 - 11010)	11010 (± 0)	6410.31 (5721.07 - 9192.6)	6897.35 (± 1437.52)	7488.64 (7000.74 - 7767.51)	7453.84 (± 291.57)
Liver	[B] 2,4,6-Trihydroxyacetophenone (THA)	4145.6 (2543.6 - 5854.4)	4095.76 (± 990.59)	5106.8 (4430.4 - 6317.2)	5170.88 (± 709.95)	4359.2 (3718.4 - 4786.4)	4323.6 (± 388.35)
Liver	[B] 4-Amino-2,6-dichlorophenol (ADCP)	3279.64 (2015.84 - 6430.24)	3496.8 (± 1266.81)	3439.84 (2834.64 - 4329.84)	3539.52 (± 560.29)	3902.64 (3617.84 - 4507.84)	4045.04 (± 375.91)
Liver	[C] Aflatoxin	5121.3 (4629 - 5977.6)	5188.03 (± 415.5)	4783 (4083 - 5464.3)	4806.32 (± 637.37)	5058.3 (4321 - 5375.6)	4918.3 (± 464.81)
Liver	[C] Allyl alcohol	3415 (1655 - 5295)	3483 (± 1116.85)	2815 (1885 - 4285)	3073 (± 941.63)	3685 (3395 - 3835)	3631 (± 214.78)
Liver	[C] Allyl formate	4911.35 (3989.7 - 6057.06)	4865.62 (± 607.3)	4615 (4223 - 5105)	4666.32 (± 351.26)	5198.3 (4932.3 - 6067.73)	5301.27 (± 447.11)
Liver	[B] Azathioprine	3439.84 (1624.24 - 4685.84)	3322.36 (± 1005.39)	2443.04 (2051.44 - 3831.44)	2635.28 (± 691.5)	6145.44 (4828.24 - 6679.44)	5846.4 (± 786.67)
Liver	[B] Bromobenzene	4715.2 (3433.6 - 4928.8)	4398.36 (± 588.2)	7527.6 (6637.6 - 8951.6)	7513.36 (± 921.69)	4572.8 (4216.8 - 6566.4)	4950.16 (± 949.13)
Liver	[C] Butylated hydroxytoluene	4514.65 (4120.3 - 4741)	4481.05 (± 209.72)	5305.7 (5067.7 - 5599.7)	5324.34 (± 192.55)	5109.7 (4951 - 5595)	5160.06 (± 255.25)
Liver	[D] Carbon tetrachloride	10031.58 (7500 - 14000)	10526.32 (± 2105.47)	5600 (3300 - 8100)	5960 (± 2118.49)	6400 (2800 - 9200)	6180 (± 2272)
Liver	[C] Chlorpromazine	6892.35 (5226.35 - 7825.65)	6897.94 (± 789.37)	5800.35 (5487.65 - 7405.65)	6084.07 (± 787.19)	5044.35 (4727.05 - 5553.05)	5119.03 (± 313.91)
Liver	[B] Clofibrate	3896.4 (2241 - 5089)	3871.48 (± 865.01)	4448.2 (3522.6 - 5765.4)	4711.64 (± 973.52)	5551.8 (5373.8 - 6085.8)	5608.76 (± 276.44)

Target organ	Toxin	24 h post dose		48 h post dose		168 h post dose	
		Median (min - max)	Mean (± SD)	Median (min - max)	Mean (± SD)	Median (min - max)	Mean (± SD)
Liver	[B] Cyproterone acetate	1991.8 (1635.8 - 4519.4)	2372.72 (± 877.02)	3522.6 (2739.4 - 3914.2)	3501.24 (± 459.78)	4412.6 (3878.6 - 6263.8)	4697.4 (± 912.15)
Liver	[A] D-galactosamine	6249.5 (4286 - 8213)	6249.5 (± 1228.07)	5714 (4643 - 13568)	7036.92 (± 3677.98)	4643 (4286 - 5000)	4643 (± 357)
Liver	[B] Diethylhexylphthalate (DEHP)			6174.8 (5035.6 - 6815.6)	5897.12 (± 789.81)	5498.4 (4893.2 - 6139.2)	5555.36 (± 483.3)
Liver	[C] Dimethylformamide (DMF)	5053.65 (4680.35 - 5487.65)	5019.12 (± 228.73)	4507.65 (4274.35 - 4876.35)	4583.25 (± 248.83)	5006.95 (4572.95 - 5398.95)	4997.63 (± 297.89)
Liver	[C] Dimethylnitrosamine (DMN)	4703.65 (4015.3 - 5508.7)	4819.86 (± 485.31)	5695.3 (5476 - 9088)	6317.86 (± 1551.74)	4738.7 (4636 - 5233.3)	4896.4 (± 287.02)
Liver	[A] Gadolinium chloride	5400 (4700 - 6100)	5310 (± 506.51)	4300 (3900 - 5700)	4420 (± 742.97)	4300 (3900 - 4700)	4220 (± 334.66)
Liver	[A, B, C, D, F] Hydrazine	5946.5 (2864 - 9819.71)	5815.29 (± 1683.06)	7856 (4565 - 9819.71)	7655.77 (± 1266.23)	5714 (3576 - 9129.6)	5933.6 (± 1727.59)
Liver	[E] Hydrazine	6000 (1000 - 9000)	5700 (± 2213.59)	11500 (9000 - 14000)	11500 (± 2380.48)	4000 (2000 - 7000)	4400 (± 2073.64)
Liver	[E] Indomethacin	7856 (3929 - 18923)	9141.2 (± 4763.57)	4643 (2501 - 5714)	4500.2 (± 1252.05)	6013.63 (5000 - 23921)	9486.73 (± 8110.08)
Liver	[E] Ketoconazole			5357 (4286 - 5714)	5071.4 (± 731.63)	4643 (2858 - 5000)	4286 (± 910.17)
Liver	[C] Lead acetate	4708.3 (4477.35 - 5275.35)	4816.12 (± 257.52)	5167.95 (4416.65 - 10511.35)	6083.23 (± 2500.86)	4892.65 (4103.95 - 5284.65)	4712.51 (± 530.42)
Liver	[A] Lipopolysaccharide (LPS)	7150 (5421.2 - 13200)	7582.12 (± 2212.69)	5050.55 (4300 - 6100)	5110.11 (± 687.74)	4300 (3900 - 5000)	4440 (± 421.9)
Liver	[B] Methapyrilene	3647.2 (2419 - 4483.8)	3704.16 (± 666.96)	4626.2 (3771.8 - 5729.8)	4768.6 (± 717.76)	4697.4 (3878.6 - 5373.8)	4683.16 (± 587.99)
Liver	[E] Methylene dianiline	3572 (2144 - 5000)	3714.8 (± 909.4)	5357 (4643 - 6071)	5285.6 (± 638.62)	4286 (3572 - 5714)	4571.6 (± 924.07)
Liver	[C] Monocrotaline	4293.05 (4066.7 - 5065.4)	4422.77 (± 357.86)	4622 (4118 - 5004.7)	4543.62 (± 351.63)	5364 (5042 - 6353.4)	5517.08 (± 525.45)
Liver	[C] N-methylformamide (NMF)	4437.7 (4055.05 - 5142.35)	4511.42 (± 341.8)	4479.65 (4134.35 - 4797.05)	4475.95 (± 313.38)	4848.35 (4573.05 - 5030.35)	4835.29 (± 177.39)
Liver	[D] Phalloidin (chronic)			5170 (4160 - 5530)	4932 (± 602.93)	4990 (4240 - 7428.29)	5259.66 (± 1258.69)
Liver	[E] Phenyl diisothiocyanate	9284 (4643 - 21065)	10212.2 (± 4973)	31418 (16781 - 55337)	34702.4 (± 18008.87)	6071 (5620.93 - 6428)	6086.01 (± 309.44)
Liver	[E] Phenyl isothiocyanate	5000 (3572 - 6428)	4892.9 (± 825.31)	5357 (3215 - 5714)	4928.6 (± 990.63)	5000 (3929 - 6428)	5000 (± 977.68)
Liver	[B] Retinyl palmitate	2710.04 (1588.64 - 3795.84)	2727.84 (± 768.13)	3404.24 (2977.04 - 4685.84)	3674.8 (± 668.67)	3795.84 (3119.44 - 4579.04)	3874.16 (± 541.31)
Liver	[B] Sodium Valproate			6410.76 (5462.5 - 8595.5)	6848.35 (± 1407.9)	4964.5 (4038.5 - 7698.99)	5375.8 (± 1406.76)

Target organ	Toxin	24 h post dose		48 h post dose		168 h post dose	
		Median (min - max)	Mean (± SD)	Median (min - max)	Mean (± SD)	Median (min - max)	Mean (± SD)
Liver	[C] a-Naphthylisothiocyanate (ANIT)	3445 (2665 - 5405)	3695 (± 914.16)	4745 (3875 - 5785)	4833 (± 805.09)	4745 (4475 - 6475)	5155 (± 816.36)
Kidney	[D] 2-Bromophenol	4500 (2950 - 5550)	4460 (± 772.37)	4850 (3450 - 5350)	4650 (± 809.32)	4450 (4050 - 5750)	4810 (± 782.94)
Kidney	[E] 3,5-Dichloroaniline hydrochloride	4643 (3215 - 6428)	4785.8 (± 909.4)	5000 (2858 - 6071)	4571.6 (± 1321.38)	3929 (3215 - 5000)	4000.4 (± 686.7)
Kidney	[E] Atractyloside	27134 (3929 - 39629)	24269.92 (± 9682.87)	37487 (21422 - 46769)	35487.8 (± 10093.07)	5714 (4643 - 6428)	5642.6 (± 686.7)
Kidney	[D] Bromoethylamine hydrobromide	7536.11 (6572.17 - 11106.26)	7910.04 (± 1236.86)	4568.7 (3678.7 - 7230.36)	5189.03 (± 1468.25)	4788.7 (3498.7 - 5788.7)	4694.7 (± 839.06)
Kidney	[D] Cephaloridine	4732.5 (3894 - 7000)	5100.4 (± 1053.93)	4568 (4178 - 5388)	4742 (± 563.45)	6288 (5498 - 6498)	6134 (± 434.09)
Kidney	[B] Chlorethanamine	4590.5 (3202.5 - 10250.5)	5120.9 (± 1946.54)	5266.5 (4092.5 - 6299.5)	5259.9 (± 863.64)	4234.5 (3807.5 - 4910.5)	4305.5 (± 398.61)
Kidney	[A] Cisplatin	4855 (3555 - 5765)	4854.47 (± 756.56)	6165 (4665 - 7665)	6049 (± 1129.26)	4555 (4275 - 7945)	5326.06 (± 1528.19)
Kidney	[A] D-limonene (chronic)						
Kidney	[E] Dichlorophenyl succinimide	4643 (3929 - 6071)	4714.4 (± 602.1)	5714 (5000 - 6785)	5785.4 (± 686.7)	5000 (4286 - 5714)	5000 (± 564.47)
Kidney	[D] Ethylene glycol	5001.12 (4501.3 - 5679.45)	5090.38 (± 426.85)	6105.98 (5675.98 - 8105.98)	6513.98 (± 1009.84)	5995.98 (4895.98 - 6105.98)	5777.98 (± 510.07)
Kidney	[A] Folic acid	34645 (4210 - 51300)	27998 (± 16911.58)	47535 (5230.55 - 90500)	51650.09 (± 29042.64)	5849.92 (5100 - 6647.33)	5797.45 (± 571.95)
Kidney	[A] Gentamicin	5125 (4180 - 5680)	5072 (± 483.75)	5570 (5390 - 6890)	5826 (± 628.87)	5070 (3400 - 7180)	5486 (± 1558.89)
Kidney	[B] Maleic acid	5130.84 (4329.84 - 7142.24)	5255.44 (± 798.16)	4614.64 (4009.44 - 6252.24)	4814 (± 892.35)	3831.44 (3261.84 - 4187.44)	3774.48 (± 387.21)
Kidney	[A] N-phenylanthranilic acid (chronic)	5714 (4561.38 - 6428)	5590.17 (± 511.97)	4643 (3929 - 43556)	12354.2 (± 17446.67)	5357 (4643 - 6785)	5526.98 (± 832.91)
Kidney	[D] Para-aminophenol	17817.78 (5215.14 - 38810.29)	18435.42 (± 9101.6)	35020 (19990 - 79610)	38928 (± 23847.76)	6890 (5000 - 8210)	6684 (± 1534.04)
Kidney	[A] Puromycin			5090 (3810 - 6800)	4998 (± 1176.04)	4590 (4410 - 5590)	4874 (± 535.1)
Kidney	[B] Vancomycin hydrochloride			6299.4 (4839.8 - 8043.8)	6320.76 (± 1195.81)	5373.8 (4270.2 - 6904.6)	5544.68 (± 948.39)
Liver & Kidney	[E] Acetaminophen	5714 (4643 - 21779)	7320.5 (± 5130.13)	6428 (5000 - 86396)	22064.6 (± 35967.11)	6071 (5000 - 6071)	5642.6 (± 586.61)
Liver & Kidney	[B] Aurothiomalate	23411.44 (5041.84 - 32418.24)	21919.8 (± 8297.77)	56234.64 (34874.64 - 64600.64)	52831.28 (± 12187.39)	7355.84 (3404.24 - 14048.64)	8509.28 (± 5188)
Liver & Kidney	[C] Chloroform	5203 (4526.3 - 5576.3)	5120.39 (± 401.73)	4983.7 (3966.3 - 7503.7)	5326.2 (± 1335.29)	4671 (4461 - 4871.7)	4669.14 (± 187.62)

Target organ	Toxin	24 h post dose		48 h post dose		168 h post dose	
		Median (min - max)	Mean (± SD)	Median (min - max)	Mean (± SD)	Median (min - max)	Mean (± SD)
Liver & Kidney	[D] Cyclosporin	4860 (2920 - 6915.5)	4700.55 (± 1227.46)	7645.88 (6020 - 13700)	8388.63 (± 2734.63)	5020 (4590 - 7428.29)	5593.66 (± 1132.63)
Liver & Kidney	[D] Dichlorobenzene	5747.39 (4140.82 - 6247.21)	5540.32 (± 640.82)	5639.53 (4859.53 - 8249.53)	5947.53 (± 1345.39)	5749.53 (4859.53 - 6849.53)	5731.53 (± 736.63)
Liver & Kidney	[C] Ethionine	6299.65 (5210.05 - 6969.35)	6205.88 (± 530.24)	6092.05 (5270.65 - 6666.05)	5953.89 (± 558.75)	5130.65 (4612.65 - 5928.65)	5200.65 (± 473.01)
Liver & Kidney	[B] Hexachlorobutadiene (HCBD)			26929.6 (19667.2 - 36862)	27776.88 (± 6212.81)	5676.4 (5035.6 - 8097.2)	5939.84 (± 1250.97)
Liver & Kidney	[B] Mercuric chloride	21643.5 (5428 - 24865)	19699.8 (± 5566.75)	48468 (34833 - 54520)	47143.6 (± 8011.63)	7698.99 (4609 - 33694)	12286.38 (± 12080.75)
Liver & Kidney	[E] Microcystin-LR			6036.4 (3750.5 - 6963.5)	5635.68 (± 1330.69)	5535.5 (4107.5 - 6296.49)	5330.7 (± 872.61)
Liver & Kidney	[E] Rotenone	7512.76 (4821.5 - 23028.5)	9086.57 (± 5348.79)	13389.5 (6036.4 - 18030.5)	12711.48 (± 5258.15)	6155.06 (5620.93 - 7677.5)	6410.76 (± 771.15)
Liver & Kidney	[E] S-(1,2-dichlorovinyl)-cysteine (DCVC)	5178.5 (2858 - 13925)	5963.9 (± 2982.37)	6428 (4286 - 17495)	8284.4 (± 5243.16)	5714 (3929 - 6071)	5214.2 (± 1028.51)
Liver & Kidney	[D] Thioacetamide	4107 (3429 - 5107)	4307.1 (± 548.66)	4606 (3326 - 5426)	4536 (± 852.7)	5816 (4426 - 6316)	5554 (± 817.2)
Pancreas	[E] 1-Cyano-2-hydroxy-3-butene	4821.5 (2322.5 - 7320.5)	4821.5 (± 1387.77)	4464.5 (1965.5 - 51588.5)	13346.88 (± 21436.48)	3393.5 (2322.5 - 4464.5)	3322.1 (± 852.33)
Pancreas	[C] Caerulin	4500.65 (3824 - 5893.26)	4560.59 (± 581.84)	4407.3 (4211.3 - 4612.7)	4434.4 (± 153.33)	5107.3 (5009.3 - 6545.17)	5416.35 (± 645.76)
Pancreas	[E] L-arginine	7856 (5000 - 63548)	14539.22 (± 15828.6)	5000 (3929 - 8927)	5361.8 (± 1485.4)	3572 (2501 - 5357)	3857.6 (± 1074.96)
Pancreas	[B] Streptozotocin	3950 (2241 - 6121)	3964 (± 1267.05)	5837 (5587 - 6477)	5893.4 (± 367.04)	6228 (5908 - 7261)	6349.2 (± 532.16)
Testicular	[D] 1,3-Dinitrobenzene	4400 (3200 - 14100)	5350 (± 3156.39)	4400 (3100 - 13000)	6178.35 (± 4049.06)	4900 (3800 - 5200)	4720 (± 563.03)
Testicular	[C] Cadmium chloride	4897.35 (4232.35 - 5296.35)	4784.43 (± 414.31)	4796.95 (4680.35 - 6010.35)	5026.59 (± 555.44)	5240.35 (4908.95 - 5814.35)	5336.47 (± 399.35)
Testicular	[D] Cadmium chloride	3715 (2945 - 5765)	3899 (± 935.99)	4305 (4095 - 4425)	4299 (± 127.59)	5005 (3955 - 5715)	4759 (± 727)
Testicular	[D] Carbendazim	4400 (4050 - 5750)	4640 (± 556.68)	5750 (3850 - 6650)	5390 (± 1096.81)	5350 (4850 - 5950)	5370 (± 443.85)
Testicular	[D] Di-n-pentyl-phthalate	4085 (3015 - 4865)	4022 (± 580.84)	4515 (3895 - 4725)	4409 (± 358.72)	4525 (4015 - 6135)	4771 (± 810.33)
Testicular	[D] Ethane dimethane sulfonate (EDS)	3000 (2500 - 4700)	3300 (± 737.86)	4200 (3200 - 7700)	4600 (± 1801.39)	5200 (4500 - 5900)	5280 (± 549.55)
Testicular	[D] Methoxyacetic acid	3965 (3195 - 5415)	4144 (± 666.19)	4825 (3515 - 5065)	4497 (± 669.75)	5385 (4695 - 6195)	5299 (± 624.2)
Multiple organ	[B] Adriamycin	5551.8 (4234.6 - 6014.6)	5352.42 (± 610.06)	4839.8 (3771.8 - 6014.6)	4854.04 (± 907.83)	4555 (2347.8 - 10108.6)	5081.88 (± 2996.69)
Multiple organ	[C] Amphotericin B	7295.36 (4969.65 - 15738.23)	7829.79 (± 3188.94)	5742.26 (4838.95 - 18073.65)	9368.58 (± 5853.24)	6299.65 (5207.65 - 6887.65)	6301.51 (± 676.75)

Target organ	Toxin	24 h post dose		48 h post dose		168 h post dose	
		Median (min - max)	Mean (± SD)	Median (min - max)	Mean (± SD)	Median (min - max)	Mean (± SD)
Multiple organ	[C] Azaserine	5987.05 (5340.65 - 7072.05)	6051.89 (± 531.07)	5182.05 (4650.05 - 8122.05)	5815.77 (± 1382.73)	8182.65 (5028.05 - 15500.05)	8833.23 (± 4204.51)
Multiple organ	[A] Dexamethasone	5230 (4730 - 6050)	5263 (± 346.09)	6340 (5340 - 12479.17)	7745.83 (± 2976.14)	6932.93 (4550 - 8230)	6678.59 (± 1469.59)
Multiple organ	[E] Mitomycin-C	5000 (4286 - 6428)	5102.79 (± 690.84)	5357 (5000 - 6071)	5357 (± 437.23)	6071 (5000 - 6785)	5928.2 (± 648.52)
Physiological stressor	[C] 1,1-Dichloroethylene & maleic acid	4855.3 (4162.35 - 5226.35)	4794.18 (± 286.9)	5044.35 (4680.35 - 5212.35)	4979.95 (± 234.18)	5529.65 (4750.35 - 6238.95)	5488.57 (± 539.21)
Physiological stressor	[C] 2,4-Dinitrophenol	4934.65 (4605.65 - 5123.65)	4891.71 (± 164.04)	5179.65 (4703.65 - 5478.35)	5062.99 (± 340.71)	5496.95 (4810.95 - 5562.35)	5383.11 (± 321.42)
Physiological stressor	[B] 4-Pentenoic acid	5373.8 (3932 - 10173.08)	5478.15 (± 1809.59)	5356 (4181.2 - 6410.76)	5324.87 (± 805.54)	4928.8 (4323.6 - 5284.8)	4772.16 (± 417.14)
Physiological stressor	[D] Acetazolamide	5963.94 (4000.36 - 12104.61)	6474.47 (± 2314.28)	4996.17 (4716.17 - 7271.76)	5467.29 (± 1041.24)	4826.17 (4426.17 - 6496.17)	5136.17 (± 797.93)
Physiological stressor	[C] Acivicin	5021 (4533.3 - 5606.7)	5107.34 (± 365.87)	4412 (4146 - 4892.7)	4492.26 (± 285.58)	5471.3 (5046.7 - 5662.7)	5429.34 (± 255.06)
Physiological stressor	[E] Ammonium chloride			3572 (3215 - 3929)	3643.4 (± 298.69)	5714 (3929 - 6785)	5499.8 (± 1200.07)
Physiological stressor	[D] Carboplatin	3950 (2650 - 6150)	4120 (± 898.21)	3750 (3250 - 4250)	3770 (± 370.14)	4250 (3250 - 5150)	4150 (± 734.85)
Physiological stressor	[A] Choline and choline/methionine deficiency (chronic)	3392.5 (2143 - 3928)	3356.8 (± 510.45)	3901 (2901 - 4301)	3641 (± 572.71)	3928 (3214 - 4285)	3785.2 (± 541.42)
Physiological stressor	[B] Food restriction (chronic)	2223.2 (1938.4 - 3967.6)	2606.89 (± 754.93)	3255.6 (1760.4 - 7314)	3896.4 (± 2640.65)	3255.8 (2294.4 - 3967.6)	3205.8 (± 597.62)
Physiological stressor	[D] Furosemide	4393.07 (3571.94 - 6805.04)	4480.75 (± 927.39)	4997.58 (4397.58 - 5497.58)	4913.58 (± 411.44)	5067.58 (4177.58 - 6177.58)	5155.58 (± 792.1)
Physiological stressor	[B] Insulin	4608.4 (4163.4 - 4875.4)	4604.84 (± 195.13)	4875.4 (4270.2 - 5195.8)	4797.08 (± 344.6)	4377 (3879 - 4626)	4320.2 (± 273.96)
Physiological stressor	[E] Methotrexate	4821.5 (3929 - 5357)	4785.8 (± 451.57)	5000 (3929 - 6071)	4928.6 (± 852.33)	5000 (3929 - 5357)	4857.2 (± 541.42)
Physiological stressor	[A] Partial hepatectomy	4286 (3572 - 5357)	4321.7 (± 517.34)	3215 (1787 - 3572)	2929.4 (± 686.7)	4286 (3929 - 4643)	4286 (± 252.44)
Physiological stressor	[A] Phenobarbital (chronic)	5000 (3555 - 6665)	5051 (± 891.53)	5765 (5375 - 7165)	6083 (± 720.92)	4275 (3945 - 6665)	4621 (± 1154.48)
Physiological stressor	[A] Pregnenolone 16 alpha carbonitrile (chronic)						
Physiological stressor	[A] Probenecid	4643 (3929 - 5000)	4571.6 (± 438.85)	5000 (4286 - 5357)	4857.2 (± 541.42)	5714 (4643 - 6428)	5499.8 (± 695.92)
Physiological stressor	[C] Rosiglitazone	4733.95 (4316.35 - 5356.95)	4792.3 (± 324.9)	5216.95 (4792.35 - 5305.65)	5114.31 (± 211.19)	4712.95 (4348.95 - 5216.95)	4813.77 (± 370.3)
Physiological stressor	[C] Rosiglitazone (chronic)	4703.7 (4409.7 - 6514.4)	4818.49 (± 615.02)				

Target organ	Toxin	24 h post dose		48 h post dose		168 h post dose	
		Median (min - max)	Mean (± SD)	Median (min - max)	Mean (± SD)	Median (min - max)	Mean (± SD)
Physiological stressor	E Sodium bicarbonate	6353.23 (5980.36 - 8619.46)	6583.77 (± 833.75)				
Physiological stressor	A Unilateral nephrectomy	6963.5 (5357 - 7856)	6713.6 (± 748.85)	5714 (3929 - 6428)	5428.4 (± 990.63)	7499 (6785 - 8213)	7427.6 (± 529.52)
Physiological stressor	B Water deprivation (chronic)	5694.2 (4804.2 - 8951.22)	6105.51 (± 1363.48)	4377 (3629.4 - 6898.87)	4720.18 (± 1178.74)	4857.6 (3949.8 - 8785.82)	5438.14 (± 1840.44)
No Effect	E Acetaminophen (chronic)	4643 (3929 - 5357)	4714.4 (± 638.62)			5000 (4643 - 5714)	5142.8 (± 407.04)
No Effect	C Buthionine sulphoxime	4584.65 (3756.3 - 5282.3)	4595.39 (± 461.88)	5352.3 (4377 - 5576.3)	5067.66 (± 519.68)	4573 (4377 - 5221.7)	4716.74 (± 349.86)
No Effect	C Ferrous sulphate	5067.65 (4472.7 - 5681.3)	5100.8 (± 395.52)	4594 (4421.3 - 4776)	4599.6 (± 146.64)	4860 (4346.7 - 5210)	4867.48 (± 337.17)
No Effect	B Ifosfamide	5000 (3629.4 - 5943.4)	4868.28 (± 666.4)	5338.2 (5124.6 - 6584.2)	5751.16 (± 732.44)	5017.8 (4519.4 - 5765.4)	5138.84 (± 584.75)
No Effect	B Lithocholic acid	5053.4 (4021 - 5801)	5017.8 (± 505.41)	3878.6 (3202.2 - 4519.4)	3899.96 (± 543.76)	5373.8 (4270.2 - 5480.6)	5032.04 (± 562.1)
No Effect	E Paraquat			5178.5 (4464.5 - 5535.5)	5035.7 (± 407.04)	4464.5 (3393.5 - 4821.5)	4250.3 (± 541.42)
No Effect	D Potassium dichromate	4800 (3100 - 6500)	4700 (± 1065.62)	4100 (3100 - 5200)	4120 (± 772.66)	5100 (3800 - 6200)	4960 (± 912.69)
No Effect	C Trichlorethylene	4941.7 (4570.65 - 5233.35)	4918.35 (± 192.59)	5023.35 (4692.05 - 5219.35)	4950.55 (± 236.02)	5294.05 (4986.05 - 5718.65)	5293.09 (± 275.5)

A-F: Indicates Pharmaceutical Company & sample origin

Supplementary Table S29. Summary statistics for serum creatinine (umol/L) at 24 hrs, 48 hrs and 168 hrs post high dose.

Target organ	Toxin	24 h post dose		48 h post dose		168 h post dose	
		Median (min - max)	Mean (± SD)	Median (min - max)	Mean (± SD)	Median (min - max)	Mean (± SD)
Liver	E 1,1-Dichloroethylene	35.36 (35.36 - 44.2)	38.9 (± 4.56)	26.52 (26.52 - 35.36)	28.29 (± 3.95)	35.36 (26.52 - 44.2)	37.13 (± 7.4)
Liver	E 1,2,3,4,5,6-hexachlorocyclohexane	38.44 (26.52 - 44.2)	37.88 (± 5.19)	26.52 (26.52 - 26.52)	26.52 (± 0)	35.36 (35.36 - 35.36)	35.36 (± 0)
Liver	B 1-Fluoropentane	30.4 (28.09 - 34.79)	31.06 (± 2.16)	38.01 (38.01 - 46.85)	41.55 (± 4.84)	38.01 (38.01 - 38.01)	38.01 (± 0)
Liver	B 2,4,6-Trihydroxyacetophenone (THA)	35.36 (35.36 - 35.36)	35.36 (± 0)	35.36 (35.36 - 35.36)	35.36 (± 0)	35.36 (35.36 - 35.36)	35.36 (± 0)
Liver	B 4-Amino-2,6-dichlorophenol (ADCP)	30.77 (21.93 - 30.77)	29.89 (± 2.8)	30.77 (30.77 - 30.77)	30.77 (± 0)	30.77 (30.77 - 30.77)	30.77 (± 0)

Target organ	Toxin	24 h post dose		48 h post dose		168 h post dose	
		Median (min - max)	Mean (± SD)	Median (min - max)	Mean (± SD)	Median (min - max)	Mean (± SD)
Liver	[C] Aflatoxin	39.36 (32.36 - 43.36)	38.76 (± 3.6)	21.36 (19.36 - 26.36)	21.96 (± 2.7)	40.36 (32.36 - 43.36)	39.56 (± 4.55)
Liver	[C] Allyl alcohol	30.86 (25.86 - 33.86)	30.76 (± 2.18)	33.86 (30.86 - 34.86)	33.26 (± 1.82)	35.86 (31.86 - 37.86)	35.46 (± 2.19)
Liver	[C] Allyl formate	31.86 (26.36 - 43.4)	33.16 (± 4.88)	34.36 (32.36 - 35.36)	33.96 (± 1.14)	39.36 (32.36 - 41.28)	37.54 (± 3.61)
Liver	[B] Azathioprine	30.77 (30.77 - 39.61)	31.66 (± 2.8)	30.77 (30.77 - 30.77)	30.77 (± 0)	30.77 (30.77 - 30.77)	30.77 (± 0)
Liver	[B] Bromobenzene	35.36 (26.52 - 35.36)	34.48 (± 2.8)	35.36 (35.36 - 44.2)	37.13 (± 3.95)	35.36 (35.36 - 35.36)	35.36 (± 0)
Liver	[C] Butylated hydroxytoluene	35.36 (31.36 - 38.36)	34.86 (± 2.42)	34.36 (30.36 - 36.36)	33.96 (± 2.3)	36.36 (33.36 - 38.36)	35.96 (± 1.82)
Liver	[D] Carbon tetrachloride	37.86 (33.36 - 44.36)	38.28 (± 3.48)	40.36 (38.36 - 44.36)	40.96 (± 2.79)	39.36 (35.36 - 40.36)	38.76 (± 2.07)
Liver	[C] Chlorpromazine	37.86 (7.36 - 46.36)	35.46 (± 10.85)	34.36 (31.36 - 39.36)	34.76 (± 2.97)	36.36 (34.36 - 40.36)	36.56 (± 2.28)
Liver	[B] Clofibrate	35.36 (35.36 - 35.36)	35.36 (± 0)	35.36 (35.36 - 35.36)	35.36 (± 0)	35.36 (35.36 - 35.36)	35.36 (± 0)
Liver	[B] Cyproterone acetate	35.36 (26.52 - 35.36)	33.59 (± 3.73)	35.36 (35.36 - 35.36)	35.36 (± 0)	35.36 (35.36 - 44.2)	38.9 (± 4.84)
Liver	[A] D-galactosamine	35.36 (35.36 - 44.2)	37.13 (± 3.73)	35.36 (35.36 - 43.3)	36.95 (± 3.55)	35.36 (35.36 - 44.2)	38.9 (± 4.84)
Liver	[B] Diethylhexylphthalate (DEHP)			44.2 (35.36 - 44.2)	40.66 (± 4.84)	35.36 (35.36 - 35.36)	35.36 (± 0)
Liver	[C] Dimethylformamide (DMF)	35.86 (33.86 - 40.86)	36.16 (± 2.11)	31.86 (28.86 - 34.86)	31.86 (± 2.24)	36.86 (34.86 - 40.86)	37.66 (± 3.03)
Liver	[C] Dimethylnitrosamine (DMN)	32.36 (28.36 - 36.36)	32.06 (± 2.36)	36.36 (32.36 - 40.36)	35.96 (± 2.97)	34.36 (31.36 - 36.36)	34.16 (± 1.92)
Liver	[A] Gadolinium chloride	36.24 (31.82 - 39.78)	35.71 (± 2.83)	33.59 (28.29 - 38.9)	33.77 (± 3.87)	32.71 (27.4 - 39.78)	33.06 (± 5.4)
Liver	[A, B, C, D, F] Hydrazine	33.98 (23.86 - 70.72)	37.48 (± 10.27)	35.36 (21.86 - 70.72)	39.06 (± 14.31)	37.36 (31.36 - 46.85)	38.39 (± 4.1)
Liver	[E] Hydrazine	35.46 (35.36 - 35.66)	35.49 (± 0.08)	35.26 (35.26 - 35.26)	35.26 (± 0)	35.36 (35.26 - 35.36)	35.32 (± 0.05)
Liver	[E] Indomethacin	44.2 (35.36 - 53.04)	42.43 (± 5.59)	35.36 (26.52 - 35.36)	33.59 (± 3.95)	35.36 (26.52 - 52.1)	35.17 (± 10.44)
Liver	[E] Ketoconazole			35.36 (35.36 - 35.36)	35.36 (± 0)	35.36 (35.36 - 44.2)	38.9 (± 4.84)
Liver	[C] Lead acetate	32.86 (26.86 - 36.86)	32.46 (± 3.17)	30.86 (28.86 - 42.93)	34.47 (± 6.45)	39.86 (35.86 - 40.86)	38.66 (± 2.17)

Target organ	Toxin	24 h post dose		48 h post dose		168 h post dose	
		Median (min - max)	Mean (± SD)	Median (min - max)	Mean (± SD)	Median (min - max)	Mean (± SD)
Liver	[A] Lipopolysaccharide (LPS)	31.38 (27.85 - 43.15)	32.82 (± 5.03)	34.03 (30.5 - 43.35)	35.37 (± 5.53)	33.15 (30.5 - 41.99)	34.74 (± 4.35)
Liver	[B] Methapyrilene	35.36 (26.52 - 35.36)	34.48 (± 2.8)	35.36 (35.36 - 35.36)	35.36 (± 0)	26.52 (26.52 - 35.36)	28.29 (± 3.95)
Liver	[E] Methylene dianiline	44.2 (44.2 - 53.04)	47.74 (± 4.56)	44.2 (35.36 - 44.2)	40.66 (± 4.84)	35.36 (35.36 - 44.2)	38.9 (± 4.84)
Liver	[C] Monocrotaline	33.36 (27.36 - 36.36)	33.16 (± 2.78)	35.36 (28.36 - 35.36)	33.36 (± 3.08)	38.36 (34.36 - 41.36)	37.96 (± 2.51)
Liver	[C] N-methylformamide (NMF)	33.86 (30.36 - 37.36)	33.56 (± 2.15)	34.36 (30.36 - 38.36)	34.56 (± 2.86)	36.36 (34.36 - 39.36)	36.56 (± 1.92)
Liver	[D] Phalloidin (chronic)			34.56 (33.56 - 37.96)	35.44 (± 2.12)	38.86 (36.16 - 41.93)	38.53 (± 2.38)
Liver	[E] Phenyl diisothiocyanate	43.76 (37.19 - 129.31)	53.13 (± 27.62)	53.04 (44.2 - 70.72)	53.04 (± 10.83)	46.26 (44.2 - 52.1)	47.59 (± 3.81)
Liver	[E] Phenyl isothiocyanate	40.41 (35.36 - 61.88)	41.83 (± 7.81)	35.36 (35.36 - 35.36)	35.36 (± 0)	35.36 (26.52 - 35.36)	31.82 (± 4.84)
Liver	[B] Retinyl palmitate	30.77 (21.93 - 30.77)	29.89 (± 2.8)	30.77 (30.77 - 30.77)	30.77 (± 0)	30.77 (21.93 - 30.77)	29 (± 3.95)
Liver	[B] Sodium Valproate			35.36 (35.36 - 39.59)	36.21 (± 1.89)	35.36 (35.36 - 37.71)	35.83 (± 1.05)
Liver	[C] a-Naphthylisothiocyanate (ANIT)	26.86 (19.36 - 32.36)	26.26 (± 4.09)	18.36 (12.36 - 43.21)	21.53 (± 12.45)	36.36 (35.36 - 38.36)	36.56 (± 1.3)
Kidney	[D] 2-Bromophenol	33.86 (30.86 - 35.86)	33.46 (± 1.78)	35.86 (29.86 - 42.86)	36.46 (± 5.81)	35.86 (34.86 - 37.86)	36.26 (± 1.14)
Kidney	[E] 3,5-Dichloroaniline hydrochloride	44.2 (26.52 - 88.4)	44.2 (± 16.67)	35.36 (17.68 - 44.2)	35.36 (± 10.83)	44.2 (35.36 - 44.2)	40.66 (± 4.84)
Kidney	[E] Atractyloside	176.8 (44.2 - 212.16)	151.01 (± 56.22)	176.8 (106.08 - 256.36)	169.73 (± 59.1)	44.2 (35.36 - 44.2)	42.43 (± 3.95)
Kidney	[D] Bromoethylamine hydrobromide	39.86 (35.36 - 45.36)	39.9 (± 3.02)	38.36 (35.36 - 41.36)	38.56 (± 2.39)	36.36 (35.36 - 41.36)	37.36 (± 2.55)
Kidney	[D] Cephaloridine	38.86 (36.36 - 75.36)	43.06 (± 11.91)	38.36 (36.36 - 42.36)	38.76 (± 2.3)	37.36 (34.36 - 40.36)	37.56 (± 2.17)
Kidney	[B] Chlorethanamine	35.36 (35.36 - 35.36)	35.36 (± 0)	35.36 (17.36 - 35.36)	31.76 (± 8.05)	35.36 (35.36 - 35.36)	35.36 (± 0)
Kidney	[A] Cisplatin	36.36 (33.36 - 43.15)	37.14 (± 2.81)	40.36 (35.36 - 49.36)	41.56 (± 5.22)	40.36 (31.36 - 50.36)	42.23 (± 7.83)
Kidney	[A] D-limonene (chronic)						
Kidney	[E] Dichlorophenyl succinimide	44.2 (35.36 - 44.2)	43.32 (± 2.8)	44.2 (35.36 - 44.2)	40.66 (± 4.84)	35.36 (35.36 - 44.2)	38.9 (± 4.84)

Target organ	Toxin	24 h post dose		48 h post dose		168 h post dose	
		Median (min - max)	Mean (± SD)	Median (min - max)	Mean (± SD)	Median (min - max)	Mean (± SD)
Kidney	[D] Ethylene glycol	33.36 (31.36 - 38.36)	34.36 (± 2.36)	40.36 (35.36 - 46.36)	40.56 (± 4.15)	40.36 (39.36 - 43.36)	40.76 (± 1.67)
Kidney	[A] Folic acid	175.86 (31.86 - 267.86)	162.26 (± 96.74)	200.86 (43.34 - 402.86)	225.77 (± 125.2)	42.55 (34.86 - 45.86)	41 (± 4.89)
Kidney	[A] Gentamicin	31.36 (27.36 - 35.36)	31.46 (± 2.56)	36.36 (31.36 - 43.36)	37.36 (± 4.64)	37.36 (35.36 - 41.36)	37.56 (± 2.28)
Kidney	[B] Maleic acid	35.19 (30.77 - 66.13)	39.61 (± 12.5)	39.61 (30.77 - 57.29)	39.61 (± 10.83)	30.77 (30.77 - 30.77)	30.77 (± 0)
Kidney	[A] N-phenylanthranilic acid (chronic)	35.36 (26.52 - 42.37)	34.11 (± 5.93)	35.36 (26.52 - 106.08)	47.74 (± 32.84)	35.36 (35.36 - 42.55)	36.8 (± 3.21)
Kidney	[D] Para-aminophenol	125.36 (38.36 - 219.36)	125.56 (± 50.58)	207.36 (95.36 - 332.36)	204.56 (± 93.68)	42.36 (36.36 - 44.36)	40.76 (± 3.29)
Kidney	[A] Puromycin			33.36 (29.36 - 37.36)	33.16 (± 2.86)	36.36 (34.36 - 44.36)	37.56 (± 3.9)
Kidney	[B] Vancomycin hydrochloride			35.36 (35.36 - 44.2)	38.9 (± 4.84)	35.36 (35.36 - 35.36)	35.36 (± 0)
Liver & Kidney	[E] Acetaminophen	39.78 (30.94 - 75.14)	45.08 (± 12.64)	48.62 (48.62 - 366.86)	112.27 (± 142.32)	30.94 (22.1 - 30.94)	29.17 (± 3.95)
Liver & Kidney	[B] Aurothiomalate	265.04 (30.77 - 340.18)	241.17 (± 95.65)	596.54 (207.58 - 658.43)	527.59 (± 182.29)	48.45 (30.77 - 74.97)	51.99 (± 18.33)
Liver & Kidney	[C] Chloroform	37.86 (35.36 - 44.36)	38.76 (± 3.03)	41.36 (32.36 - 43.36)	38.76 (± 5.03)	34.36 (32.36 - 37.36)	34.76 (± 1.82)
Liver & Kidney	[D] Cyclosporin	34.36 (26.36 - 44.36)	35.22 (± 5.85)	39.86 (35.36 - 41.36)	38.69 (± 2.66)	35.36 (31.36 - 41.93)	36.87 (± 4.6)
Liver & Kidney	[D] Dichlorobenzene	35.36 (31.36 - 39.36)	35.86 (± 2.88)	37.36 (35.36 - 40.36)	37.56 (± 2.28)	39.36 (39.36 - 41.36)	40.16 (± 1.1)
Liver & Kidney	[C] Ethionine	38.36 (30.36 - 47.36)	37.96 (± 5.62)	37.36 (33.36 - 39.36)	36.56 (± 2.59)	36.36 (32.36 - 41.36)	36.56 (± 3.49)
Liver & Kidney	[B] Hexachlorobutadiene (HCBD)			274.05 (176.8 - 291.73)	254.6 (± 48.58)	44.2 (35.36 - 53.04)	42.43 (± 7.4)
Liver & Kidney	[B] Mercuric chloride	224.86 (26.36 - 247.36)	195.86 (± 65.55)	388.36 (273.36 - 423.36)	365.36 (± 61.11)	37.71 (35.36 - 105.36)	50.3 (± 30.8)
Liver & Kidney	[E] Microcystin-LR			44.2 (44.2 - 53.04)	46.73 (± 3.89)	35.36 (35.36 - 46.26)	39.31 (± 5.46)
Liver & Kidney	[E] Rotenone	53.04 (44.2 - 61.88)	52.89 (± 6.06)	53.04 (48 - 61.88)	53.99 (± 5.77)	45.23 (35.36 - 52.1)	44.08 (± 7.37)
Liver & Kidney	[E] S-(1,2-dichlorovinyl)-cysteine (DCVC)	44.2 (35.36 - 53.04)	42.43 (± 6.97)	35.36 (26.52 - 35.36)	33.59 (± 3.95)	26.52 (26.52 - 35.36)	30.06 (± 4.84)
Liver & Kidney	[D] Thioacetamide	32.86 (28.86 - 35.86)	32.66 (± 2.15)	38.86 (36.86 - 39.86)	38.46 (± 1.52)	37.86 (35.86 - 42.86)	38.66 (± 2.59)

Target organ	Toxin	24 h post dose		48 h post dose		168 h post dose	
		Median (min - max)	Mean (± SD)	Median (min - max)	Mean (± SD)	Median (min - max)	Mean (± SD)
Pancreas	[E] 1-Cyano-2-hydroxy-3-butene	44.2 (35.36 - 132.6)	53.92 (± 28.09)	26.52 (26.52 - 106.08)	46.73 (± 34.46)	35.36 (17.68 - 44.2)	31.82 (± 10.08)
Pancreas	[C] Caerulin	30.86 (28.36 - 41.1)	32.23 (± 3.72)	37.36 (31.36 - 40.36)	36.36 (± 3.54)	36.36 (35.36 - 38.98)	36.68 (± 1.53)
Pancreas	[E] L-arginine	35.36 (26.52 - 97.24)	40.33 (± 15.48)	35.36 (35.36 - 48.09)	37.76 (± 4.85)	44.2 (44.2 - 53.04)	45.08 (± 2.8)
Pancreas	[B] Streptozotocin	35.36 (26.36 - 35.36)	33.56 (± 3.79)	35.36 (35.36 - 43.36)	36.96 (± 3.58)	35.36 (35.36 - 35.36)	35.36 (± 0)
Testicular	[D] 1,3-Dinitrobenzene	32.86 (29.36 - 50.36)	34.56 (± 5.9)	36.36 (30.36 - 52.36)	39.16 (± 9.47)	38.36 (36.36 - 40.36)	37.96 (± 1.67)
Testicular	[C] Cadmium chloride	30.36 (25.86 - 43.51)	31.03 (± 4.79)	35.86 (28.86 - 40.86)	35.46 (± 4.34)	38.86 (34.86 - 39.86)	38.46 (± 2.07)
Testicular	[D] Cadmium chloride	32.11 (29.21 - 34.81)	32.23 (± 1.72)	30.21 (27.01 - 32.41)	29.45 (± 2.27)	36.61 (35.31 - 39.61)	37.13 (± 1.87)
Testicular	[D] Carbendazim	32.86 (31.86 - 38.86)	34.16 (± 2.5)	36.86 (33.86 - 39.86)	37.06 (± 2.39)	36.86 (34.86 - 42.86)	37.86 (± 3.16)
Testicular	[D] Di-n-pentyl-phthalate	39.21 (36.31 - 43.51)	39.33 (± 2.28)	34.21 (32.41 - 37.71)	34.65 (± 2.11)	34.11 (32.81 - 36.21)	34.41 (± 1.32)
Testicular	[D] Ethane dimethane sulfonate (EDS)	31.86 (27.86 - 33.86)	31.46 (± 2.07)	31.86 (30.86 - 38.86)	33.86 (± 3.39)	36.86 (34.86 - 38.86)	37.06 (± 1.48)
Testicular	[D] Methoxyacetic acid	33.31 (31.91 - 40.51)	34.04 (± 2.56)	34.21 (30.01 - 39.11)	34.11 (± 3.3)	37.51 (34.41 - 40.21)	37.07 (± 2.35)
Multiple organ	[B] Adriamycin	35.6 (35.36 - 35.84)	35.6 (± 0.25)	35.36 (26.52 - 35.36)	31.82 (± 4.84)	35.36 (35.36 - 35.36)	35.36 (± 0)
Multiple organ	[C] Amphotericin B	38.86 (6.86 - 50.86)	38.36 (± 13.14)	42.86 (39.86 - 78.86)	48.87 (± 16.83)	34.86 (28.86 - 43.86)	36.26 (± 5.55)
Multiple organ	[C] Azaserine	41.36 (34.36 - 48.36)	40.86 (± 4.97)	73.36 (66.36 - 117.36)	79.96 (± 21.17)	141.36 (40.36 - 214.36)	121.96 (± 78.86)
Multiple organ	[A] Dexamethasone	39.36 (38.36 - 44.36)	40.36 (± 2.11)	49.36 (47.36 - 51.36)	49.56 (± 1.79)	41.36 (35.36 - 44.85)	40.86 (± 3.5)
Multiple organ	[E] Mitomycin-C	44.2 (35.36 - 44.2)	43.32 (± 2.8)	35.36 (35.36 - 35.36)	35.36 (± 0)	35.36 (35.36 - 35.36)	35.36 (± 0)
Physiological stressor	[C] 1,1-Dichloroethylene & maleic acid	33.36 (29.36 - 37.36)	33.46 (± 2.13)	39.36 (31.36 - 43.36)	37.76 (± 4.56)	42.36 (39.36 - 44.36)	42.16 (± 1.92)
Physiological stressor	[C] 2,4-Dinitrophenol	33.36 (30.36 - 38.36)	33.96 (± 3.13)	33.36 (29.36 - 36.36)	32.56 (± 3.11)	36.36 (32.36 - 37.36)	35.36 (± 2)
Physiological stressor	[B] 4-Pentenoic acid	35.36 (17.68 - 35.36)	33.59 (± 5.59)	35.36 (35.36 - 39.59)	36.21 (± 1.89)	35.36 (26.52 - 35.36)	33.59 (± 3.95)
Physiological stressor	[D] Acetazolamide	31.86 (30.36 - 100.36)	39.96 (± 21.55)	36.36 (34.36 - 42.36)	37.76 (± 3.44)	33.36 (31.36 - 36.36)	33.56 (± 1.92)
Physiological stressor	[C] Acivicin	34.36 (31.36 - 36.36)	34.06 (± 1.64)	27.36 (24.36 - 31.36)	28.36 (± 3)	35.36 (33.36 - 38.36)	35.36 (± 2.12)

Target organ	Toxin	24 h post dose		48 h post dose		168 h post dose	
		Median (min - max)	Mean (± SD)	Median (min - max)	Mean (± SD)	Median (min - max)	Mean (± SD)
Physiological stressor	E Ammonium chloride			44.2 (35.36 - 44.2)	42.43 (± 3.95)	35.36 (35.36 - 44.2)	37.13 (± 3.95)
Physiological stressor	D Carboplatin	32.36 (28.36 - 34.36)	31.96 (± 1.51)	36.36 (35.36 - 39.36)	37.16 (± 1.64)	42.36 (39.36 - 44.36)	42.36 (± 2.12)
Physiological stressor	A Choline and choline/methionine deficiency (chronic)	35.36 (35.36 - 35.36)	35.36 (± 0)	35.36 (35.36 - 35.36)	35.36 (± 0)	35.36 (26.52 - 35.36)	31.82 (± 4.84)
Physiological stressor	B Food restriction (chronic)	35.36 (26.52 - 35.36)	31.43 (± 4.66)	35.36 (35.36 - 35.36)	35.36 (± 0)	35.36 (35.36 - 35.36)	35.36 (± 0)
Physiological stressor	D Furosemide	33.36 (27.36 - 38.75)	33.2 (± 2.78)	39.36 (35.36 - 44.36)	39.56 (± 3.83)	40.36 (38.36 - 43.36)	40.76 (± 2.07)
Physiological stressor	B Insulin	26.52 (26.52 - 35.36)	29.17 (± 4.27)	35.84 (26.84 - 35.84)	32.24 (± 4.93)	35.84 (26.84 - 35.84)	34.04 (± 4.02)
Physiological stressor	E Methotrexate	35.36 (26.52 - 35.36)	33.59 (± 3.73)	26.52 (17.68 - 26.52)	22.98 (± 4.84)	26.52 (17.68 - 35.36)	28.29 (± 7.4)
Physiological stressor	A Partial hepatectomy	31.82 (27.85 - 35.8)	31.65 (± 2.21)	27.85 (22.54 - 29.61)	26.96 (± 2.65)	31.38 (28.73 - 34.03)	31.21 (± 2.45)
Physiological stressor	A Phenobarbital (chronic)	35.36 (32.86 - 37.86)	35.26 (± 1.65)	32.86 (31.86 - 37.86)	34.66 (± 2.95)	39.86 (37.86 - 48.86)	41.66 (± 4.27)
Physiological stressor	A Pregnenolone 16 alpha carbonitrile (chronic)						
Physiological stressor	A Probenecid	35.36 (26.52 - 44.2)	33.59 (± 5.59)	35.36 (35.36 - 35.36)	35.36 (± 0)	44.2 (35.36 - 44.2)	42.43 (± 3.95)
Physiological stressor	C Rosiglitazone	35.86 (34.36 - 39.36)	35.96 (± 1.58)	33.36 (32.36 - 35.36)	33.76 (± 1.14)	35.36 (34.36 - 38.36)	35.76 (± 1.67)
Physiological stressor	C Rosiglitazone (chronic)	34.36 (32.36 - 35.36)	33.96 (± 0.97)				
Physiological stressor	E Sodium bicarbonate	50.7 (50.63 - 56.86)	51.86 (± 2.42)				
Physiological stressor	A Unilateral nephrectomy	45.53 (35.8 - 52.6)	45.53 (± 4.79)	41.99 (36.69 - 50.83)	42.7 (± 5.09)	44.64 (42.87 - 49.95)	45.7 (± 2.89)
Physiological stressor	B Water deprivation (chronic)	35.36 (35.36 - 44.2)	36.79 (± 2.83)	35.36 (35.36 - 40.15)	36.16 (± 1.96)	38.01 (35.36 - 44.2)	39.19 (± 4.39)
No Effect	E Acetaminophen (chronic)	35.36 (35.36 - 35.36)	35.36 (± 0)			44.2 (44.2 - 44.2)	44.2 (± 0)
No Effect	C Buthionine sulphoximе	33.86 (31.86 - 36.86)	33.86 (± 1.49)	35.86 (33.86 - 36.86)	35.46 (± 1.14)	32.86 (29.86 - 36.86)	32.86 (± 3.08)
No Effect	C Ferrous sulphate	30.86 (28.36 - 34.36)	31.16 (± 1.62)	30.36 (28.36 - 34.36)	31.36 (± 2.45)	36.36 (35.36 - 38.36)	36.56 (± 1.1)

Target organ	Toxin	24 h post dose		48 h post dose		168 h post dose	
		Median (min - max)	Mean (± SD)	Median (min - max)	Mean (± SD)	Median (min - max)	Mean (± SD)
No Effect	[B] Ifosfamide	35.36 (26.52 - 35.36)	34.48 (± 2.8)	35.36 (35.36 - 44.2)	37.13 (± 3.95)	35.36 (35.36 - 35.36)	35.36 (± 0)
No Effect	[B] Lithocholic acid	35.36 (35.36 - 35.36)	35.36 (± 0)	35.36 (35.36 - 35.36)	35.36 (± 0)	35.36 (35.36 - 35.36)	35.36 (± 0)
No Effect	[E] Paraquat			30.94 (30.94 - 30.94)	30.94 (± 0)	30.94 (30.94 - 30.94)	30.94 (± 0)
No Effect	[D] Potassium dichromate	30.86 (24.36 - 37.36)	30.96 (± 4.01)	34.36 (31.36 - 39.36)	34.76 (± 3.21)	43.36 (34.36 - 46.36)	40.96 (± 5.27)
No Effect	[C] Trichlorethylene	34.36 (27.86 - 37.86)	34.26 (± 2.95)	37.86 (31.86 - 39.86)	36.66 (± 3.27)	38.86 (36.86 - 42.86)	39.06 (± 2.28)

A-F: Indicates Pharmaceutical Company & sample origin

Supplementary Table S30. Summary statistics for serum alanine aminotransferase (IU/L) at 24 hrs, 48 hrs and 168 hrs post high dose.

Target organ	Toxin	24 h post dose		48 h post dose		168 h post dose	
		Median (min - max)	Mean (± SD)	Median (min - max)	Mean (± SD)	Median (min - max)	Mean (± SD)
Liver	[E] 1,1-Dichloroethylene	77.5 (54.5 - 179.5)	90.7 (± 38.26)	56.5 (52.5 - 72.5)	60.5 (± 8.28)	43.5 (40.5 - 46.5)	43.1 (± 2.3)
Liver	[E] 1,2,3,4,5,6-hexachlorocyclohexane	51.5 (44 - 62)	52.5 (± 5.78)	48 (44 - 64)	50.2 (± 7.95)	48 (44 - 53)	48.4 (± 3.36)
Liver	[B] 1-Fluoropentane	29.8 (23.7 - 45.36)	31.98 (± 6.63)	47.51 (45.79 - 74.88)	55.16 (± 12.62)	47.05 (46.15 - 58.58)	50.42 (± 5.57)
Liver	[B] 2,4,6-Trihydroxyacetophenone (THA)	50 (41 - 65)	52 (± 8.69)	53 (41 - 60)	52.2 (± 6.98)	57 (51 - 59)	55.8 (± 3.03)
Liver	[B] 4-Amino-2,6-dichlorophenol (ADCP)	160.25 (117.25 - 218.25)	166.05 (± 37.84)	67.25 (60.25 - 84.25)	69.65 (± 9.5)	55.25 (45.25 - 70.25)	57.45 (± 9.6)
Liver	[C] Aflatoxin	660.8 (229.4 - 2060.6)	863.18 (± 662.04)	5174.6 (4184.6 - 6746.6)	5431.4 (± 1110.68)	69.2 (57.2 - 124.4)	84.32 (± 28.77)
Liver	[C] Allyl alcohol	1618.7 (175.7 - 2709.5)	1577.18 (± 977.7)	277.7 (74.3 - 464.9)	292.94 (± 161.37)	41.3 (29.9 - 59.3)	42.86 (± 10.82)
Liver	[C] Allyl formate	170.9 (48.5 - 996.5)	294.54 (± 313.83)	60.5 (52.1 - 83.9)	63.38 (± 12.81)	56.9 (53.3 - 59.9)	56.59 (± 2.94)
Liver	[B] Azathioprine	41.75 (31.25 - 76.25)	46.25 (± 15.81)	42.25 (26.25 - 140.25)	61.45 (± 45.49)	50.25 (40.25 - 64.25)	51.05 (± 8.67)
Liver	[B] Bromobenzene	102.5 (44 - 305)	120.1 (± 79.01)	268 (83 - 1008)	356.6 (± 379.39)	52 (48 - 53)	51.2 (± 2.17)
Liver	[C] Butylated hydroxytoluene	51.5 (32.6 - 88.4)	57.32 (± 17.84)	81.8 (71 - 97.4)	83.12 (± 11.23)	51.2 (42.2 - 57.2)	50 (± 6.01)

Target organ	Toxin	24 h post dose		48 h post dose		168 h post dose	
		Median (min - max)	Mean (± SD)	Median (min - max)	Mean (± SD)	Median (min - max)	Mean (± SD)
Liver	[D] Carbon tetrachloride	155 (102 - 2356)	391.8 (± 694.42)	123 (95 - 916)	287.6 (± 353.05)	57 (48 - 71)	58.2 (± 8.29)
Liver	[C] Chlorpromazine	54.8 (39.5 - 326.9)	85.34 (± 86.17)	60.5 (44.3 - 89.9)	63.26 (± 16.73)	63.5 (50.9 - 80.3)	63.86 (± 10.66)
Liver	[B] Clofibrate	62 (43.5 - 138.5)	67.4 (± 26.09)	65.5 (48.5 - 71.5)	62.9 (± 9.26)	57.5 (39.5 - 60.5)	52.7 (± 8.64)
Liver	[B] Cyproterone acetate	41.5 (31.5 - 60.5)	45.5 (± 11.6)	75.5 (57.5 - 92.5)	75.3 (± 13.08)	119.5 (52.5 - 190.5)	122.9 (± 51.86)
Liver	[A] D-galactosamine	1566.5 (351.5 - 13601.5)	3874.5 (± 5047.7)	1433.5 (292.5 - 7416.5)	2533.81 (± 2945.49)	28.5 (17.5 - 31.5)	25.3 (± 7.22)
Liver	[B] Diethylhexylphthalate (DEHP)			65.5 (51.5 - 69.5)	63.3 (± 6.87)	56.5 (46.5 - 61.5)	54.7 (± 5.81)
Liver	[C] Dimethylformamide (DMF)	43.7 (37.1 - 56.3)	45.2 (± 6.13)	203.9 (65.3 - 361.1)	215.78 (± 134.42)	51.5 (40.7 - 53.3)	48.74 (± 5.46)
Liver	[C] Dimethylnitrosamine (DMN)	65.6 (48.2 - 94.4)	66.74 (± 12.23)	94.4 (64.4 - 237.8)	117.32 (± 68.73)	45.2 (41 - 65.6)	50.12 (± 10.08)
Liver	[A] Gadolinium chloride	72 (44 - 97)	73.5 (± 15.71)	65 (54 - 93)	69.8 (± 15.22)	53 (46 - 63)	53.6 (± 6.69)
Liver	[A, B, C, D, F] Hydrazine	24.31 (1 - 134.52)	30.24 (± 24.97)	19.5 (1 - 55.28)	23.86 (± 17.67)	35 (10.5 - 96.83)	37.57 (± 18.75)
Liver	[E] Hydrazine	19 (15 - 30)	20.4 (± 4.27)	16 (15 - 16)	15.75 (± 0.5)	37 (14 - 45)	34 (± 11.73)
Liver	[E] Indomethacin	44 (37.5 - 60.5)	45 (± 6.43)	31.5 (31.5 - 33.5)	32.1 (± 0.89)	44.5 (32.5 - 57.48)	44.9 (± 9.17)
Liver	[E] Ketoconazole			51 (50 - 54)	51.6 (± 1.52)	47 (40 - 51)	46 (± 4.47)
Liver	[C] Lead acetate	53 (45.5 - 87.5)	58.1 (± 13)	57.76 (35.3 - 96.5)	58.15 (± 23.65)	47.9 (38.3 - 78.5)	53.18 (± 17.16)
Liver	[A] Lipopolysaccharide (LPS)	92 (29.5 - 236.5)	110.59 (± 77.84)	33.5 (18.5 - 224.91)	66.78 (± 88.63)	46.5 (40.5 - 51.5)	46.1 (± 5.03)
Liver	[B] Methapyrilene	148.5 (65.5 - 531.5)	217 (± 158.96)	167.5 (53.5 - 214.5)	158.9 (± 62.4)	74.5 (51.5 - 80.5)	68.7 (± 12.21)
Liver	[E] Methylene dianiline	238.5 (77.5 - 952.5)	320.2 (± 282.22)	228.5 (96.5 - 453.5)	240.7 (± 133.71)	47.5 (38.5 - 50.5)	45.1 (± 5.27)
Liver	[C] Monocrotaline	58.7 (48.8 - 66.2)	57.02 (± 5.37)	54.8 (47 - 54.8)	52.16 (± 3.71)	50 (48.8 - 54.8)	51.32 (± 2.7)
Liver	[C] N-methylformamide (NMF)	60.5 (26.3 - 79.7)	58.4 (± 16.08)	91.1 (66.5 - 315.5)	132.86 (± 103.22)	52.1 (48.5 - 59.3)	53.18 (± 4.03)
Liver	[D] Phalloidin (chronic)			73 (63.3 - 130.9)	84.9 (± 27.48)	45.1 (40 - 51)	45.9 (± 4.4)

Target organ	Toxin	24 h post dose		48 h post dose		168 h post dose	
		Median (min - max)	Mean (± SD)	Median (min - max)	Mean (± SD)	Median (min - max)	Mean (± SD)
Liver	[E] Phenyl diisothiocyanate	62 (42.5 - 572.15)	114.33 (± 161.55)	70.5 (62.5 - 74.5)	70.1 (± 4.98)	49.02 (42.5 - 57.48)	49.77 (± 5.41)
Liver	[E] Phenyl isothiocyanate	71.5 (61.5 - 100.5)	75.2 (± 14.38)	55.5 (52.5 - 166.5)	77.7 (± 49.74)	50.5 (47.5 - 54.5)	50.9 (± 2.7)
Liver	[B] Retinyl palmitate	50.75 (37.25 - 85.25)	53.65 (± 12.73)	41.25 (39.25 - 65.25)	45.25 (± 11.22)	54.25 (47.25 - 64.25)	56.05 (± 7.46)
Liver	[B] Sodium Valproate			40 (33 - 74.5)	48.7 (± 18.21)	55 (42.85 - 59)	52.17 (± 6.32)
Liver	[C] a-Naphthylisothiocyanate (ANIT)	156.5 (95.6 - 372.2)	186.98 (± 87.83)	1076.6 (965.6 - 1452.8)	1185.2 (± 214.6)	53 (44 - 62)	53.48 (± 7.92)
Kidney	[D] 2-Bromophenol	45.5 (32.5 - 53.5)	43.9 (± 7.26)	53.5 (38.5 - 66.5)	50.7 (± 11.78)	50.5 (41.5 - 55.5)	48.1 (± 6.31)
Kidney	[E] 3,5-Dichloroaniline hydrochloride	56 (49 - 99)	60.3 (± 15.27)	43 (34 - 69)	46.8 (± 13.18)	57 (51 - 62)	56 (± 4.9)
Kidney	[E] Atractyloside	55.5 (42.5 - 288.34)	70.02 (± 60.71)	44.5 (40.5 - 45.5)	43.9 (± 2.07)	41.5 (39.5 - 50.5)	43.9 (± 4.51)
Kidney	[D] Bromoethylamine hydrobromide	25 (19 - 103.75)	35.67 (± 27.07)	29 (25 - 62.91)	34.58 (± 15.97)	36 (25 - 46)	35.6 (± 7.64)
Kidney	[D] Cephaloridine	54 (37 - 71)	55.1 (± 10.27)	62 (51 - 77)	63.4 (± 9.76)	60 (46 - 66)	57.2 (± 8.41)
Kidney	[B] Chlorethanamine	28.5 (23 - 46)	30.8 (± 6.32)	27 (26 - 29)	27 (± 1.22)	53 (50 - 57)	53.2 (± 3.27)
Kidney	[A] Cisplatin	44.5 (32 - 191.5)	60.25 (± 47.26)	40 (36 - 54)	41.4 (± 7.27)	52 (34 - 66)	51 (± 11.79)
Kidney	[A] D-limonene (chronic)						
Kidney	[E] Dichlorophenyl succinimide	53.5 (23.5 - 61.5)	50.9 (± 10.7)	43.5 (18.5 - 47.5)	39.7 (± 11.97)	47.5 (43.5 - 58.5)	50.1 (± 6.19)
Kidney	[D] Ethylene glycol	52.5 (37.5 - 63.5)	52.4 (± 7.68)	50.5 (37.5 - 63.5)	51.3 (± 9.5)	60.5 (39.5 - 69.5)	57.3 (± 11.3)
Kidney	[A] Folic acid	38 (28 - 58)	39 (± 9.33)	32 (23 - 193.2)	56.53 (± 67.13)	67 (45 - 187.2)	106.68 (± 74.01)
Kidney	[A] Gentamicin	43 (34 - 61)	43.6 (± 7.24)	46 (40 - 56)	45.8 (± 6.34)	62 (50 - 96)	67.6 (± 17.27)
Kidney	[B] Maleic acid	46.25 (34.25 - 50.25)	44.35 (± 4.91)	45.25 (31.25 - 50.25)	42.65 (± 7.13)	53.25 (52.25 - 62.25)	55.85 (± 4.62)
Kidney	[A] N-phenylanthranilic acid (chronic)	76 (52 - 226.86)	98.61 (± 67.85)	48 (17 - 55)	43.4 (± 15.04)	51 (37 - 185.05)	74.21 (± 62.69)
Kidney	[D] Para-aminophenol	62 (45 - 69)	59.1 (± 9.47)	65 (42 - 138)	77.6 (± 36.5)	45 (38 - 51)	44.4 (± 5.08)

Target organ	Toxin	24 h post dose		48 h post dose		168 h post dose	
		Median (min - max)	Mean (± SD)	Median (min - max)	Mean (± SD)	Median (min - max)	Mean (± SD)
Kidney	[A] Puromycin			53.5 (44.5 - 70.5)	56.5 (± 10.42)	52.5 (48.5 - 54.5)	52.1 (± 2.61)
Kidney	[B] Vancomycin hydrochloride			53 (42 - 55)	50 (± 5.7)	49 (44 - 56)	49.4 (± 4.51)
Liver & Kidney	[E] Acetaminophen	1316 (207 - 11787)	3149.3 (± 3902.84)	657 (319 - 3090)	1174.8 (± 1148.26)	41 (40 - 59)	45.4 (± 7.96)
Liver & Kidney	[B] Aurothiomalate	46.25 (36.25 - 51.25)	44.85 (± 5.7)	33.25 (15.25 - 39.25)	31.25 (± 9.27)	37.25 (33.25 - 59.25)	42.05 (± 10.43)
Liver & Kidney	[C] Chloroform	56 (34.7 - 268.7)	78.44 (± 69.35)	76.7 (49.1 - 160.1)	84.14 (± 44.95)	48.5 (32.3 - 65.9)	47.18 (± 13.4)
Liver & Kidney	[D] Cyclosporin	30 (17.5 - 59.71)	32.02 (± 13.28)	36 (27.5 - 59.31)	37.97 (± 11.1)	53.5 (39.5 - 71.5)	52.4 (± 13.34)
Liver & Kidney	[D] Dichlorobenzene	66 (44.5 - 501.5)	118.1 (± 139.14)	106.5 (92.5 - 127.5)	108.7 (± 15.48)	44.5 (32.5 - 46.5)	40.7 (± 6.65)
Liver & Kidney	[C] Ethionine	72.5 (53 - 174.8)	79.82 (± 35.12)	68.6 (51.8 - 77.6)	64.76 (± 10.8)	52.4 (41 - 124.4)	65.6 (± 33.74)
Liver & Kidney	[B] Hexachlorobutadiene (HCBD)			57 (43 - 60)	53.2 (± 7.01)	39 (29 - 51)	39.2 (± 8.01)
Liver & Kidney	[B] Mercuric chloride	73.5 (49 - 125)	74.5 (± 22.33)	54 (37 - 59)	48.6 (± 9.91)	52 (42.85 - 96.83)	63.54 (± 22.4)
Liver & Kidney	[E] Microcystin-LR			831 (70.18 - 1872)	843.24 (± 726.59)	51.37 (41 - 55)	49.27 (± 6.45)
Liver & Kidney	[E] Rotenone	70.67 (34.5 - 157.5)	75.77 (± 39.36)	31.5 (26.5 - 70.33)	39.96 (± 20.45)	49.76 (39.5 - 57.48)	49.06 (± 5.94)
Liver & Kidney	[E] S-(1,2-dichlorovinyl)-cysteine (DCVC)	89 (59 - 114)	91 (± 17.38)	58 (45 - 87)	61 (± 15.57)	52 (50 - 57)	52.6 (± 2.7)
Liver & Kidney	[D] Thioacetamide	114.5 (73.5 - 609.5)	172.1 (± 159.43)	87.5 (59.5 - 209.5)	119.5 (± 61.88)	42.5 (39.5 - 50.5)	43.9 (± 4.28)
Pancreas	[E] 1-Cyano-2-hydroxy-3-butene	76 (47.5 - 801.5)	148.7 (± 229.88)	60.5 (49.5 - 2333.5)	514.84 (± 1016.69)	77.5 (67.5 - 125.5)	87.5 (± 24.63)
Pancreas	[C] Caerulin	56.3 (47.3 - 91.7)	60.37 (± 13.58)	49.1 (41.3 - 61.1)	48.98 (± 8.1)	50.9 (48.54 - 59.9)	52.71 (± 4.77)
Pancreas	[E] L-arginine	137.5 (12 - 327)	144.11 (± 82.05)	91 (34 - 137)	86.01 (± 34.78)	61.5 (49 - 100)	66.2 (± 15.1)
Pancreas	[B] Streptozotocin	49.5 (40 - 59)	49.2 (± 5.41)	51 (44 - 58)	51.2 (± 4.97)	80 (54 - 191)	98.4 (± 53.31)
Testicular	[D] 1,3-Dinitrobenzene	52.5 (43 - 65)	52.9 (± 8.28)	57 (48 - 62)	56.8 (± 5.54)	59 (51 - 70)	61 (± 7.58)
Testicular	[C] Cadmium chloride	29.9 (20.6 - 54.46)	32.93 (± 11.53)	80.6 (40.4 - 373.4)	127.76 (± 139.67)	44.6 (41 - 68.6)	48.92 (± 11.27)

Target organ	Toxin	24 h post dose		48 h post dose		168 h post dose	
		Median (min - max)	Mean (± SD)	Median (min - max)	Mean (± SD)	Median (min - max)	Mean (± SD)
Testicular	[D] Cadmium chloride	167.8 (143.2 - 198.2)	167.45 (± 19.89)	70.3 (64.8 - 79.8)	71.18 (± 5.48)	50 (46.5 - 61.9)	52.36 (± 5.93)
Testicular	[D] Carbendazim	46 (32.5 - 56.5)	44.5 (± 6.46)	49.5 (31.5 - 53.5)	46.3 (± 8.64)	42.5 (33.5 - 51.5)	41.1 (± 7.37)
Testicular	[D] Di-n-pentyl-phthalate	51.2 (40.7 - 89.6)	54.53 (± 13.7)	58.6 (51.7 - 72.7)	61.5 (± 8.03)	46.5 (43.3 - 53.5)	47.5 (± 3.84)
Testicular	[D] Ethane dimethane sulfonate (EDS)	44.5 (36 - 55)	44.8 (± 5.77)	53 (45 - 58)	51 (± 5.43)	43 (40 - 50)	43.8 (± 3.77)
Testicular	[D] Methoxyacetic acid	38.95 (34.65 - 42.65)	38.74 (± 2.88)	47.95 (37.15 - 56.65)	48.37 (± 7.82)	54.25 (51.05 - 57.25)	54.23 (± 2.27)
Multiple organ	[B] Adriamycin	40.5 (35.5 - 58.5)	42.2 (± 6.72)	32.5 (25.5 - 42.5)	32.9 (± 6.19)	34.5 (27.5 - 42.5)	35.5 (± 6.08)
Multiple organ	[C] Amphotericin B	52.2 (7 - 92.6)	49.02 (± 31.08)	56.6 (29.6 - 61.76)	49.47 (± 13.32)	39.8 (30.8 - 56)	41.24 (± 9.74)
Multiple organ	[C] Azaserine	64.7 (44.6 - 110.6)	71.42 (± 21.34)	147.8 (90.2 - 210.8)	153.2 (± 53.7)	64.4 (48.2 - 92)	65.48 (± 18.2)
Multiple organ	[A] Dexamethasone	71 (55 - 108)	74.3 (± 15.39)	247 (137 - 835)	346 (± 279.33)	113.2 (103 - 283)	164.77 (± 79.58)
Multiple organ	[E] Mitomycin-C	47.5 (39 - 65.67)	49.17 (± 10.13)	37 (34 - 43)	37.6 (± 3.58)	52 (46 - 73)	55 (± 10.77)
Physiological stressor	[C] 1,1-Dichloroethylene & maleic acid	50.9 (42.2 - 81.8)	53.3 (± 11.6)	50 (48.2 - 74)	56 (± 11.12)	53 (41.6 - 58.4)	52.52 (± 6.75)
Physiological stressor	[C] 2,4-Dinitrophenol	41.9 (37.1 - 54.5)	43.16 (± 5.72)	41.9 (37.7 - 59.3)	46.7 (± 10.06)	40.1 (37.1 - 44.9)	40.58 (± 3.38)
Physiological stressor	[B] 4-Pentenoic acid	62 (47 - 144)	70.28 (± 28.44)	196 (74.5 - 1289)	375.5 (± 515.41)	55 (41 - 63)	52.2 (± 9.01)
Physiological stressor	[D] Acetazolamide	46 (35 - 74)	50.1 (± 11.51)	59.31 (49 - 84)	63.66 (± 13.42)	47 (45 - 51)	47.2 (± 2.28)
Physiological stressor	[C] Acivicin	44.6 (39.5 - 53.9)	45.74 (± 5.02)	35.9 (29.9 - 58.1)	41.18 (± 11.77)	51.5 (47.3 - 55.7)	50.78 (± 3.38)
Physiological stressor	[E] Ammonium chloride			93.5 (24.5 - 162.5)	87.85 (± 51.36)	20.5 (7.5 - 46.3)	22.46 (± 16.13)
Physiological stressor	[D] Carboplatin	47.5 (39 - 63)	49.2 (± 8.16)	58 (52 - 60)	56.8 (± 3.03)	71 (58 - 75)	67.2 (± 7.66)
Physiological stressor	[A] Choline and choline/methionine deficiency (chronic)	22 (14 - 32)	21.4 (± 5.58)	29 (22 - 39)	30 (± 6.4)	24 (22 - 24)	23.4 (± 0.89)
Physiological stressor	[B] Food restriction (chronic)	43 (34 - 49)	42.67 (± 5.07)	38 (35 - 52)	40.75 (± 7.68)	40 (36 - 46)	40 (± 3.94)
Physiological stressor	[D] Furosemide	53.5 (43.5 - 61.8)	52.33 (± 4.94)	54.5 (46.5 - 60.5)	54.1 (± 4.98)	47.5 (38.5 - 52.5)	46.7 (± 5.12)
Physiological stressor	[B] Insulin	60 (49 - 72)	60.5 (± 8.59)	59 (48 - 63)	57 (± 5.79)	56 (47 - 58)	53.6 (± 4.83)

Target organ	Toxin	24 h post dose		48 h post dose		168 h post dose	
		Median (min - max)	Mean (± SD)	Median (min - max)	Mean (± SD)	Median (min - max)	Mean (± SD)
Physiological stressor	[E] Methotrexate	39 (32.5 - 50.5)	41.2 (± 6.25)	35.5 (33.5 - 55.5)	39.5 (± 9.06)	41.5 (40.5 - 60.5)	47.7 (± 9.58)
Physiological stressor	[A] Partial hepatectomy	371 (231 - 857)	473.1 (± 230.27)	97 (69 - 140)	104.2 (± 34.3)	59 (35 - 72)	56 (± 13.96)
Physiological stressor	[A] Phenobarbital (chronic)	60.5 (52 - 69)	60.7 (± 6.15)	52 (47 - 91)	59.2 (± 18.01)	48 (40 - 53)	47 (± 5.1)
Physiological stressor	[A] Pregnenolone 16 alpha carbonitrile (chronic)						
Physiological stressor	[A] Probenecid	66 (51 - 100)	70.4 (± 17.27)	45 (43 - 66)	51.8 (± 10.47)	56 (53 - 83)	61 (± 12.63)
Physiological stressor	[C] Rosiglitazone	41.6 (23.3 - 48.5)	38.36 (± 9.18)	49.1 (44.9 - 53.9)	49.22 (± 3.61)	34.7 (27.5 - 47.9)	35.42 (± 7.94)
Physiological stressor	[C] Rosiglitazone (chronic)	51.5 (39.2 - 66.2)	51.56 (± 7.37)				
Physiological stressor	[E] Sodium bicarbonate	70.38 (63.35 - 100.45)	72.37 (± 10.37)				
Physiological stressor	[A] Unilateral nephrectomy	45.5 (35.5 - 55.5)	46.4 (± 6.44)	37.5 (33.5 - 43.5)	38.1 (± 3.71)	48.5 (39.5 - 59.5)	48.7 (± 7.53)
Physiological stressor	[B] Water deprivation (chronic)	49 (37 - 103.72)	54.6 (± 18.44)	36 (32 - 63.24)	40.21 (± 11.51)	53 (39 - 87.83)	55.64 (± 17.16)
No Effect	[E] Acetaminophen (chronic)	52 (43 - 53)	50.2 (± 4.09)			47 (45 - 51)	48.2 (± 2.68)
No Effect	[C] Buthionine sulphoxime	46.4 (40.1 - 53.3)	46.28 (± 3.75)	46.7 (43.7 - 55.7)	49.22 (± 5.25)	60.5 (51.5 - 65.9)	60.86 (± 5.89)
No Effect	[C] Ferrous sulphate	26.3 (17 - 257.6)	50.06 (± 73.16)	40.4 (23 - 45.2)	37.28 (± 8.46)	50.6 (35.6 - 65.6)	52.04 (± 11.31)
No Effect	[B] Ifosfamide	38 (32.5 - 42.5)	37.8 (± 3.47)	40.5 (34.5 - 50.5)	41.3 (± 6.76)	49.5 (41.5 - 52.5)	48.9 (± 4.51)
No Effect	[B] Lithocholic acid	53.5 (43.5 - 59.5)	52.9 (± 4.81)	52.5 (46.5 - 55.5)	51.7 (± 3.42)	51.5 (48.5 - 57.5)	52.3 (± 3.27)
No Effect	[E] Paraquat			54.5 (33.5 - 59.5)	49.5 (± 10.79)	46.5 (45.5 - 66.5)	52.3 (± 9.42)
No Effect	[D] Potassium dichromate	45.5 (32 - 279)	90.6 (± 98.42)	45 (39 - 48)	44.8 (± 3.49)	44 (33 - 52)	44 (± 7.58)
No Effect	[C] Trichlorethylene	54.5 (44 - 67.4)	55.46 (± 7.63)	53.6 (51.2 - 68)	57.56 (± 7.37)	60.2 (54.2 - 66.8)	60.56 (± 5.36)

A-F: Indicates Pharmaceutical Company & sample origin

Supplementary Table S31. Summary statistics for serum aspartate aminotransferase (IU/L) at 24 hrs, 48 hrs and 168 hrs post high dose.

Target organ	Toxin	24 h post dose		48 h post dose		168 h post dose	
		Median (min - max)	Mean (± SD)	Median (min - max)	Mean (± SD)	Median (min - max)	Mean (± SD)
Liver	[E] 1,1-Dichloroethylene	157 (119.5 - 306.5)	185.1 (± 71.03)	110.5 (100.5 - 139.5)	113.1 (± 15.47)	93.5 (83.5 - 98.5)	92.1 (± 5.59)
Liver	[E] 1,2,3,4,5,6-hexachlorocyclohexane	84 (71.5 - 112.5)	85.2 (± 11.8)	84.5 (75.5 - 100.5)	84.9 (± 9.5)	108.5 (86.5 - 145.5)	108.5 (± 24.42)
Liver	[B] 1-Fluoropentane	74 (68 - 78)	73.6 (± 3.69)	131 (109 - 143)	129 (± 14.21)	118 (115 - 122)	118.2 (± 2.59)
Liver	[B] 2,4,6-Trihydroxyacetophenone (THA)	154 (118.5 - 212.5)	155.2 (± 31.45)	101.5 (90.5 - 139.5)	107.1 (± 18.93)	105.5 (96.5 - 116.5)	105.9 (± 9.07)
Liver	[B] 4-Amino-2,6-dichlorophenol (ADCP)	1176.52 (725.52 - 1578.52)	1152.62 (± 254.17)	316.52 (223.52 - 442.52)	322.92 (± 78.21)	99.52 (82.52 - 132.52)	108.92 (± 22.52)
Liver	[C] Aflatoxin	893.2 (306.4 - 3873.4)	1480.42 (± 1269.98)	7152.4 (6660.4 - 8712.4)	7393.6 (± 791.62)	143.8 (127.6 - 213.4)	155.32 (± 33.85)
Liver	[C] Allyl alcohol	3051.4 (297.4 - 4710.4)	2778.64 (± 1744.48)	519.4 (135.4 - 985)	554.92 (± 332.2)	103 (75.4 - 139.6)	102.4 (± 24.39)
Liver	[C] Allyl formate	230.8 (87.1 - 1724.5)	452.2 (± 526.18)	104.5 (78.7 - 126.7)	104.38 (± 18.43)	103.9 (76.3 - 137.75)	104.31 (± 22.11)
Liver	[B] Azathioprine	140.52 (108.52 - 190.52)	144.02 (± 28.12)	225.52 (113.52 - 254.52)	199.72 (± 58.85)	108.52 (77.52 - 159.52)	118.52 (± 34.15)
Liver	[B] Bromobenzene	252.5 (105.5 - 555.5)	266.8 (± 131.24)	470.5 (190.5 - 1549.5)	693.9 (± 589.09)	60.5 (49.5 - 109.5)	69.9 (± 23.63)
Liver	[C] Butylated hydroxytoluene	118.9 (93.4 - 367)	153.1 (± 86.61)	130 (110.2 - 269.2)	153.16 (± 66.29)	94.6 (88 - 98.2)	94 (± 4.26)
Liver	[D] Carbon tetrachloride	504.43 (310 - 5129.38)	969.21 (± 1472.91)	430 (300 - 2364)	785.6 (± 887.16)	103 (97 - 113)	103.2 (± 6.18)
Liver	[C] Chlorpromazine	140.5 (108.1 - 1128.7)	315.16 (± 330.85)	159.1 (112.9 - 256.9)	166.78 (± 58.46)	103.3 (91.3 - 132.7)	110.5 (± 19.41)
Liver	[B] Clofibrate	149 (114.5 - 205.5)	152.2 (± 30.27)	143.5 (122.5 - 165.5)	143.3 (± 15.63)	105.5 (78.5 - 119.5)	102.5 (± 14.92)
Liver	[B] Cyproterone acetate	120 (100.5 - 140.5)	121.3 (± 12.83)	160.5 (130.5 - 191.5)	159.7 (± 21.97)	164.5 (124.5 - 264.5)	189.7 (± 57.75)
Liver	[A] D-galactosamine	3248 (708 - 19168)	5775.2 (± 6073.33)	2424 (771 - 11248)	5070.8 (± 5159.89)	84 (53 - 96)	74.6 (± 19.79)
Liver	[B] Diethylhexylphthalate (DEHP)			123 (109 - 140)	123.4 (± 11.46)	101 (100 - 117)	104.4 (± 7.23)
Liver	[C] Dimethylformamide (DMF)	90.4 (82.6 - 123.4)	98.14 (± 14.81)	575.2 (163 - 2432.8)	883 (± 913.68)	120.4 (99.4 - 170.2)	129.04 (± 26.33)
Liver	[C] Dimethylnitrosamine (DMN)	195.1 (155.5 - 248.5)	205.12 (± 30.92)	381.7 (282.1 - 1603.3)	617.26 (± 554.73)	99.7 (94.3 - 114.1)	102.82 (± 7.75)
Liver	[A] Gadolinium chloride	181.5 (114.5 - 206.5)	168.6 (± 34.86)	96.5 (57.5 - 142.5)	96.7 (± 32.8)	54.5 (51.5 - 81.5)	61.3 (± 12.54)
Liver	[A, B, C, D, F] Hydrazine	55.65 (0.5 - 147.1)	55.42 (± 28.14)	46.5 (6.5 - 123.5)	53.73 (± 27.58)	68.5 (34.5 - 132.72)	73.88 (± 28.26)

Target organ	Toxin	24 h post dose		48 h post dose		168 h post dose	
		Median (min - max)	Mean (± SD)	Median (min - max)	Mean (± SD)	Median (min - max)	Mean (± SD)
Liver	[E] Hydrazine	67 (44 - 91)	66.6 (± 14)	51.5 (32 - 63)	49.5 (± 14.62)	93 (66 - 492)	176.2 (± 180.39)
Liver	[E] Indomethacin	77.5 (54 - 137)	80.8 (± 22.81)	59 (42 - 62)	53.6 (± 8.96)	68 (39 - 131.05)	74.01 (± 36.13)
Liver	[E] Ketoconazole			91 (81 - 95)	88.8 (± 6.87)	80 (73 - 100)	82 (± 10.89)
Liver	[C] Lead acetate	152.2 (86.8 - 304.6)	168.22 (± 76.86)	140.99 (91.6 - 313)	156.44 (± 90.89)	120.4 (101.8 - 146.8)	121.36 (± 18.14)
Liver	[A] Lipopolysaccharide (LPS)	154.5 (74 - 401)	201.89 (± 112.37)	64 (46 - 132.85)	73.37 (± 34.15)	75 (69 - 87)	76.4 (± 6.77)
Liver	[B] Methapyrilene	264.5 (140.5 - 683.5)	341.9 (± 203.74)	323.5 (108.5 - 925.5)	394.9 (± 310.72)	131.5 (120.5 - 164.5)	137.1 (± 18.12)
Liver	[E] Methylene dianiline	509 (152.5 - 1281.5)	593.9 (± 424.34)	406.5 (167.5 - 689.5)	397.3 (± 199.54)	92.5 (85.5 - 162.5)	110.5 (± 32.07)
Liver	[C] Monocrotaline	111.7 (99.4 - 132.4)	115.06 (± 11.98)	122.2 (104.8 - 149.2)	126.16 (± 17.01)	107.8 (89.8 - 110.8)	103.24 (± 8.66)
Liver	[C] N-methylformamide (NMF)	184 (132.7 - 220.3)	182.32 (± 27.78)	311.5 (240.7 - 1422.7)	527.02 (± 504.38)	99.7 (90.7 - 110.5)	100.9 (± 8.58)
Liver	[D] Phalloidin (chronic)			147.5 (123.7 - 293.9)	172 (± 68.95)	115.1 (97.6 - 176.1)	127.47 (± 30.89)
Liver	[E] Phenyl diisothiocyanate	139.59 (95.66 - 167.61)	136.89 (± 23.06)	188.5 (159.5 - 237.5)	193.9 (± 29.09)	130.93 (87.5 - 146.68)	125.13 (± 22.18)
Liver	[E] Phenyl isothiocyanate	186 (114 - 382)	196.6 (± 83.86)	153 (116 - 315)	178.8 (± 78.39)	85 (70 - 93)	83.2 (± 8.41)
Liver	[B] Retinyl palmitate	103.02 (95.52 - 127.52)	107.82 (± 11.75)	106.52 (91.52 - 119.52)	105.12 (± 10.97)	95.52 (91.52 - 120.52)	101.32 (± 12.01)
Liver	[B] Sodium Valproate			172.5 (104.5 - 224.5)	165.44 (± 43.24)	103.5 (97.5 - 135.75)	109.35 (± 15.57)
Liver	[C] a-Naphthylisothiocyanate (ANIT)	358.3 (224.5 - 901.9)	432.34 (± 217.41)	2750.5 (1973.5 - 2999.5)	2548.9 (± 499.77)	87.7 (83.5 - 93.1)	87.46 (± 3.9)
Kidney	[D] 2-Bromophenol	91.5 (83 - 105)	92.8 (± 6.25)	109 (97 - 119)	108.8 (± 9.44)	112 (87 - 162)	122 (± 37.42)
Kidney	[E] 3,5-Dichloroaniline hydrochloride	187 (145 - 277)	190.4 (± 42.31)	104 (97 - 109)	104.4 (± 4.72)	102 (86 - 134)	108.4 (± 19.01)
Kidney	[E] Atractyloside	100 (85 - 152.36)	107.22 (± 18.06)	92 (70 - 99)	86.4 (± 11.67)	91 (89 - 105)	95.8 (± 7.56)
Kidney	[D] Bromoethylamine hydrobromide	76.5 (59 - 156.71)	89.28 (± 35.83)	69 (50 - 161.7)	84.94 (± 45.5)	78 (71 - 98)	80.6 (± 10.57)
Kidney	[D] Cephaloridine	109.5 (81 - 135)	111.4 (± 15.78)	111 (96 - 133)	111.8 (± 14.92)	94 (83 - 116)	96.4 (± 13.76)
Kidney	[B] Chlorethanamine	83.5 (71 - 102)	84.4 (± 9.66)	69 (62 - 74)	68.2 (± 4.32)	95 (90 - 124)	100.4 (± 13.5)

Target organ	Toxin	24 h post dose		48 h post dose		168 h post dose	
		Median (min - max)	Mean (± SD)	Median (min - max)	Mean (± SD)	Median (min - max)	Mean (± SD)
Kidney	[A] Cisplatin	123 (101.5 - 145.5)	123.3 (± 14.19)	93.5 (72.5 - 116.5)	96.3 (± 17.64)	85.5 (72.1 - 100.5)	86.22 (± 10.91)
Kidney	[A] D-limonene (chronic)						
Kidney	[E] Dichlorophenyl succinimide	100 (57.5 - 128.5)	102.1 (± 21.64)	98.5 (46.5 - 99.5)	87.7 (± 23.12)	102.5 (89.5 - 122.5)	103.9 (± 13.78)
Kidney	[D] Ethylene glycol	105 (80 - 133)	103.3 (± 16.61)	86 (80 - 138)	101.6 (± 25.56)	102 (83 - 106)	95.8 (± 11.32)
Kidney	[A] Folic acid	146.5 (103 - 155)	136.7 (± 19.15)	83.5 (74 - 132.85)	92.31 (± 23.27)	111 (85 - 147.58)	113.92 (± 28.42)
Kidney	[A] Gentamicin	94 (87 - 104)	94.3 (± 5.31)	102 (90 - 115)	101.8 (± 9.83)	127 (123 - 152)	135.4 (± 14.33)
Kidney	[B] Maleic acid	98.02 (66.52 - 128.52)	97.62 (± 16.54)	92.52 (82.52 - 97.52)	90.52 (± 6.67)	100.52 (76.52 - 125.52)	101.12 (± 21.93)
Kidney	[A] N-phenylanthranilic acid (chronic)	94.5 (85.5 - 151.56)	107.31 (± 26.34)	96.5 (94.5 - 114.5)	100.1 (± 8.17)	108.5 (88.5 - 138.03)	110.81 (± 19.59)
Kidney	[D] Para-aminophenol	122 (88 - 190)	123.5 (± 32.01)	86 (80 - 99)	88.6 (± 8.47)	84 (77 - 95)	85 (± 6.67)
Kidney	[A] Puromycin			92.5 (80.5 - 122.5)	96.3 (± 15.79)	105.5 (95.5 - 126.5)	108.3 (± 12.44)
Kidney	[B] Vancomycin hydrochloride			102 (99 - 113)	103.2 (± 5.63)	97 (92 - 102)	97.2 (± 4.32)
Liver & Kidney	[E] Acetaminophen	2621.5 (779 - 29731)	8872.1 (± 10574.11)	1921 (1027 - 9082)	3428.2 (± 3270.4)	94 (84 - 112)	93.8 (± 11.23)
Liver & Kidney	[B] Aurothiomalate	246.52 (106.52 - 559.52)	279.32 (± 142.53)	173.52 (68.52 - 249.52)	165.72 (± 65.3)	92.52 (82.52 - 155.52)	104.32 (± 29.96)
Liver & Kidney	[C] Chloroform	156.7 (106.6 - 606.4)	196.72 (± 146.65)	145 (134.8 - 443.8)	224.92 (± 132.72)	73.6 (58.6 - 85)	73.48 (± 10.47)
Liver & Kidney	[D] Cyclosporin	79.5 (57 - 143.16)	87.42 (± 26.73)	145.07 (95 - 176)	145.02 (± 29.28)	96 (90 - 138.15)	103.63 (± 19.53)
Liver & Kidney	[D] Dichlorobenzene	249.5 (147 - 1431)	388.1 (± 385.53)	424 (142 - 881)	427 (± 290.28)	87 (71 - 97)	84.4 (± 11.91)
Liver & Kidney	[C] Ethionine	150.7 (127.3 - 200.5)	153.82 (± 21.7)	157.3 (135.1 - 160.9)	153.58 (± 10.46)	91.9 (77.5 - 124.3)	99.58 (± 19.69)
Liver & Kidney	[B] Hexachlorobutadiene (HCBD)			122 (93 - 173)	125 (± 31.76)	82 (70 - 110)	84 (± 15.62)
Liver & Kidney	[B] Mercuric chloride	666 (100 - 854)	570.3 (± 250.51)	262 (233 - 314)	266.2 (± 31.03)	135.75 (109 - 151)	134.09 (± 15.67)
Liver & Kidney	[E] Microcystin-LR			2280.5 (162.2 - 4902.5)	2371.84 (± 2103.42)	100.5 (98.5 - 146.68)	109.94 (± 20.74)
Liver & Kidney	[E] Rotenone	137.91 (104 - 276)	143.61 (± 49.08)	102 (75 - 163.2)	110.55 (± 38.4)	117.47 (78 - 146.68)	113.28 (± 27.07)

Target organ	Toxin	24 h post dose		48 h post dose		168 h post dose	
		Median (min - max)	Mean (± SD)	Median (min - max)	Mean (± SD)	Median (min - max)	Mean (± SD)
Liver & Kidney	[E] S-(1,2-dichlorovinyl)-cysteine (DCVC)	373 (113 - 532)	339.2 (± 130.52)	133 (100 - 220)	143.6 (± 48.47)	107 (92 - 113)	103.8 (± 8.58)
Liver & Kidney	[D] Thioacetamide	437 (216.5 - 1689.5)	584.9 (± 447.52)	260.5 (214.5 - 506.5)	342.9 (± 136.86)	97.5 (82.5 - 104.5)	94.5 (± 8.86)
Pancreas	[E] 1-Cyano-2-hydroxy-3-butene	158.5 (112.5 - 3112.96)	626.05 (± 1044.41)	126.5 (111.5 - 3634.5)	831.64 (± 1566.96)	107.5 (103.5 - 176.5)	123.3 (± 30.93)
Pancreas	[C] Caerulin	114.7 (97 - 210.4)	121.75 (± 32.54)	86.8 (67 - 146.2)	95.56 (± 30.49)	95.8 (87.4 - 137.75)	103.71 (± 20.14)
Pancreas	[E] L-arginine	357.5 (73 - 892.47)	362.61 (± 202.47)	171 (70 - 250)	167.53 (± 62.01)	83.5 (71 - 103)	85.3 (± 10.17)
Pancreas	[B] Streptozotocin	89.5 (74 - 121)	93.1 (± 15.42)	88 (83 - 98)	88.4 (± 6.19)	96 (78 - 216)	120 (± 55.55)
Testicular	[D] 1,3-Dinitrobenzene	98 (82 - 114)	97.3 (± 11.16)	115 (82 - 130)	110.8 (± 20.32)	125 (103 - 143)	123.6 (± 16.7)
Testicular	[C] Cadmium chloride	118.9 (88.9 - 164.5)	117.83 (± 21.48)	197.5 (118.9 - 704.5)	296.74 (± 243.27)	100.9 (78.1 - 120.7)	96.82 (± 18.56)
Testicular	[D] Cadmium chloride	339.7 (304.7 - 442.8)	356.45 (± 43.32)	245 (196.2 - 267)	238.92 (± 26.37)	93.6 (78.8 - 109.2)	94.12 (± 11.67)
Testicular	[D] Carbendazim	104.5 (86 - 114)	102.8 (± 9.95)	112 (86 - 117)	105.4 (± 12.52)	86 (83 - 93)	87.2 (± 3.9)
Testicular	[D] Di-n-pentyl-phthalate	92.35 (82.8 - 114.4)	96.19 (± 10.38)	139.8 (110.3 - 251.9)	162.64 (± 56.75)	100.4 (85.6 - 119.8)	100.8 (± 12.29)
Testicular	[D] Ethane dimethane sulfonate (EDS)	105.5 (94 - 137)	108.8 (± 13.77)	88 (76 - 122)	93 (± 17.2)	78 (72 - 82)	77.6 (± 4.16)
Testicular	[D] Methoxyacetic acid	90.6 (83.45 - 94.15)	89.62 (± 3.99)	94.35 (81.15 - 102.35)	93.53 (± 7.72)	126.75 (119.25 - 157.75)	134.55 (± 17.72)
Multiple organ	[B] Adriamycin	92.5 (87.5 - 123.5)	96.8 (± 10.64)	93.5 (79.5 - 120.5)	98.7 (± 16.54)	73.5 (67.5 - 82.5)	73.9 (± 5.94)
Multiple organ	[C] Amphotericin B	418 (115.3 - 712.3)	380.74 (± 199.13)	160.95 (78.1 - 1080.7)	328.67 (± 423.81)	108.7 (61.3 - 109.9)	95.02 (± 21.4)
Multiple organ	[C] Azaserine	183.4 (107.8 - 723.4)	240.46 (± 183.08)	523.6 (187.6 - 998.8)	511.84 (± 312.61)	103 (83.8 - 131.2)	106.12 (± 21.25)
Multiple organ	[A] Dexamethasone	124 (95 - 178)	132.1 (± 27.73)	267 (194 - 977)	401.4 (± 324.39)	155 (100.07 - 398)	192.75 (± 117.32)
Multiple organ	[E] Mitomycin-C	104 (81 - 184.14)	108.91 (± 28.51)	91 (80 - 137)	99.6 (± 22.2)	84 (72 - 106)	86.6 (± 12.48)
Physiological stressor	[C] 1,1-Dichloroethylene & maleic acid	103.6 (67.6 - 143.2)	104.86 (± 26.22)	103 (79 - 232)	128.8 (± 63.34)	97 (73 - 101.8)	91.48 (± 11.77)
Physiological stressor	[C] 2,4-Dinitrophenol	91.9 (85.3 - 138.1)	98.26 (± 15.68)	92.5 (85.3 - 107.5)	94.9 (± 9.61)	86.5 (81.7 - 93.7)	86.86 (± 4.72)
Physiological stressor	[B] 4-Pentenoic acid	111 (81 - 239)	119.66 (± 47.53)	270 (150 - 1820)	588.44 (± 704.69)	99 (90 - 121)	101.6 (± 12.28)
Physiological stressor	[D] Acetazolamide	90 (71 - 140)	94 (± 22)	80 (72 - 149.15)	97.63 (± 32.94)	71 (67 - 89)	73.6 (± 8.82)

Target organ	Toxin	24 h post dose		48 h post dose		168 h post dose	
		Median (min - max)	Mean (± SD)	Median (min - max)	Mean (± SD)	Median (min - max)	Mean (± SD)
Physiological stressor	[C] Acivicin	102.4 (91.3 - 138.1)	104.74 (± 13.71)	93.7 (84.1 - 103.3)	92.86 (± 7.27)	123.1 (102.7 - 132.7)	119.62 (± 11.29)
Physiological stressor	[E] Ammonium chloride			108 (103 - 118)	110.6 (± 6.58)	102 (84 - 130)	103.2 (± 17.37)
Physiological stressor	[D] Carboplatin	97 (74 - 150)	97.6 (± 21.2)	123 (101 - 253)	151 (± 64.93)	134 (99 - 149)	125.6 (± 21.15)
Physiological stressor	[A] Choline and choline/methionine deficiency (chronic)	73.5 (62.5 - 83.5)	72.3 (± 6.61)	92.5 (85.5 - 107.5)	93.9 (± 8.2)	85.5 (81.5 - 88.5)	85.1 (± 2.51)
Physiological stressor	[B] Food restriction (chronic)	101 (94 - 112)	101.56 (± 6.89)	96.5 (88 - 138)	104.75 (± 22.97)	98 (88 - 114)	99 (± 9.7)
Physiological stressor	[D] Furosemide	112 (92.5 - 223.31)	123.58 (± 38.12)	85.5 (78.5 - 117.5)	91.7 (± 15.79)	82.5 (64.5 - 103.5)	84.3 (± 17.44)
Physiological stressor	[B] Insulin	101 (92 - 130)	104.1 (± 13.49)	101 (94 - 113)	102.8 (± 7.36)	104 (97 - 111)	104.2 (± 6.3)
Physiological stressor	[E] Methotrexate	91 (80.5 - 110.5)	92.6 (± 9.35)	95.5 (86.5 - 115.5)	97.5 (± 10.75)	87.5 (68.5 - 92.5)	83.7 (± 9.83)
Physiological stressor	[A] Partial hepatectomy	736.5 (585 - 2311)	964.6 (± 570.58)	240 (233 - 394)	289.2 (± 74.29)	108 (87 - 134)	111 (± 18.77)
Physiological stressor	[A] Phenobarbital (chronic)	116.5 (89 - 146)	118.8 (± 17.79)	70 (65 - 133)	81.6 (± 28.82)	78 (62 - 93)	77.8 (± 11.17)
Physiological stressor	[A] Pregnenolone 16 alpha carbonitrile (chronic)						
Physiological stressor	[A] Probenecid	154.5 (97 - 353)	185.9 (± 95.38)	92 (77 - 106)	93 (± 11.25)	103 (101 - 122)	106.2 (± 8.87)
Physiological stressor	[C] Rosiglitazone	88 (71.2 - 130.6)	95.44 (± 19.84)	97 (89.2 - 122.2)	100.84 (± 13.74)	77.2 (73.6 - 88)	79 (± 6.03)
Physiological stressor	[C] Rosiglitazone (chronic)	106.6 (97 - 140.2)	110.86 (± 14.07)				
Physiological stressor	[E] Sodium bicarbonate	137.91 (137.91 - 152.36)	140.54 (± 5.58)				
Physiological stressor	[A] Unilateral nephrectomy	127.5 (103 - 174)	133.3 (± 20.11)	92 (66 - 322)	132.6 (± 106.83)	95 (73 - 101)	90.4 (± 12.07)
Physiological stressor	[B] Water deprivation (chronic)	97 (81 - 147.26)	103.79 (± 19.6)	94 (77 - 143.95)	99.33 (± 22.89)	87 (57 - 132.72)	89.79 (± 25.55)
No Effect	[E] Acetaminophen (chronic)	111 (108 - 130)	115.6 (± 8.85)			103 (92 - 112)	101.2 (± 7.66)
No Effect	[C] Buthionine sulphoxime	82.6 (73.9 - 141.1)	88.36 (± 19.5)	102.7 (88.9 - 128.5)	105.22 (± 16.57)	95.5 (91.3 - 127.9)	100.9 (± 15.24)
No Effect	[C] Ferrous sulphate	91 (64.6 - 430)	124.3 (± 108.89)	73.6 (64.6 - 127)	86.32 (± 25.93)	89.2 (86.2 - 116.8)	95.68 (± 12.68)
No Effect	[B] Ifosfamide	90 (81 - 111)	92.8 (± 9.57)	96 (91 - 112)	99 (± 8.57)	85 (77 - 99)	86.6 (± 8.17)

Target organ	Toxin	24 h post dose		48 h post dose		168 h post dose	
		Median (min - max)	Mean (± SD)	Median (min - max)	Mean (± SD)	Median (min - max)	Mean (± SD)
No Effect	[B] Lithocholic acid	109 (97.5 - 139.5)	112 (± 11.91)	97.5 (93.5 - 107.5)	99.7 (± 6.02)	95.5 (85.5 - 107.5)	97.3 (± 8.32)
No Effect	[E] Paraquat			108.5 (91.5 - 116.5)	106.5 (± 10.93)	93.5 (80.5 - 100.5)	91.1 (± 8.73)
No Effect	[D] Potassium dichromate	102 (82 - 556)	185.7 (± 182.75)	97 (80 - 110)	96.8 (± 11.03)	87 (81 - 114)	95.6 (± 14.83)
No Effect	[C] Trichlorethylene	95.2 (79.6 - 132.4)	98.44 (± 14.51)	106 (87.4 - 115.6)	102.4 (± 10.79)	90.4 (85 - 99.4)	92.32 (± 5.65)

A-F: Indicates Pharmaceutical Company & sample origin

Supplementary Table S32. Summary statistics for urine total volume collected (mL) at 24 hrs, 48 hrs and 168 hrs post high dose.

Target organ	Toxin	24 h post dose		48 h post dose		168 h post dose	
		Median (min - max)	Mean (± SD)	Median (min - max)	Mean (± SD)	Median (min - max)	Mean (± SD)
Liver	[E] 1,1-Dichloroethylene	5.75 (4.5 - 25)	9.2 (± 6.51)	10 (7 - 25)	11.45 (± 5.27)	12 (8.5 - 16)	12.1 (± 2.75)
Liver	[E] 1,2,3,4,5,6-hexachlorocyclohexane	10.25 (6 - 13)	10.15 (± 2.35)	14 (7 - 19)	13.7 (± 3.4)	18 (12 - 22)	17.8 (± 3.63)
Liver	[B] 1-Fluoropentane	9.2 (4 - 14.8)	8.77 (± 3.78)	9.9 (6.4 - 23.4)	11.26 (± 4.88)	16.4 (11.2 - 21.8)	16.32 (± 4.21)
Liver	[B] 2,4,6-Trihydroxyacetophenone (THA)	10 (3.2 - 12.8)	9.8 (± 3.15)	10.5 (5.6 - 13.2)	10.3 (± 2.47)	15.6 (10.2 - 19.8)	16 (± 3.75)
Liver	[B] 4-Amino-2,6-dichlorophenol (ADCP)	6.4 (4 - 15)	7.6 (± 3.67)	6.6 (3 - 12.8)	7.44 (± 3.1)	10.4 (10.2 - 18)	12.4 (± 3.34)
Liver	[C] Aflatoxin	25 (9 - 45)	25.6 (± 11.64)	26 (12 - 60)	26.6 (± 13.87)	13 (10 - 23)	15.6 (± 5.59)
Liver	[C] Allyl alcohol	27 (18 - 42)	26.7 (± 6.63)	16 (8 - 30)	16.56 (± 6.64)	11 (9 - 12)	11 (± 1.22)
Liver	[C] Allyl formate	12.5 (4 - 27)	13.4 (± 7.15)	13 (10 - 16)	12.95 (± 1.71)	14 (12 - 16)	14.2 (± 1.48)
Liver	[B] Azathioprine	31.6 (9.8 - 38.6)	27.96 (± 10.25)	14.7 (5.8 - 30)	14.94 (± 6.78)	12.6 (9 - 25.6)	14.68 (± 6.39)
Liver	[B] Bromobenzene	8.1 (5.4 - 10.2)	7.93 (± 1.46)	11.4 (9.4 - 16.2)	11.8 (± 2.05)	15.2 (9.4 - 19.3)	14.52 (± 4.58)
Liver	[C] Butylated hydroxytoluene	12.5 (10 - 29)	14.4 (± 5.44)	20 (16 - 23)	19.8 (± 2.04)	17 (12 - 20)	16.4 (± 3.05)
Liver	[D] Carbon tetrachloride	6.5 (4 - 20)	7.8 (± 4.44)	12 (10 - 25)	13.8 (± 4.57)	12 (10 - 16)	12.4 (± 2.19)

Target organ	Toxin	24 h post dose		48 h post dose		168 h post dose	
		Median (min - max)	Mean (± SD)	Median (min - max)	Mean (± SD)	Median (min - max)	Mean (± SD)
Liver	[C] Chlorpromazine	11 (8 - 13)	10.8 (± 1.62)	12 (7 - 20)	12.3 (± 3.8)	14 (10 - 15)	13.6 (± 2.07)
Liver	[B] Clofibrate	16.6 (4.2 - 32.2)	18.02 (± 8.67)	24.9 (15.2 - 51)	28.98 (± 12.6)	13.8 (11.4 - 16.4)	13.88 (± 1.78)
Liver	[B] Cyproterone acetate	18.2 (8.4 - 67.2)	23.5 (± 17.2)	36.1 (1 - 69.6)	32.98 (± 22.87)	15.8 (13.4 - 19)	15.96 (± 2.36)
Liver	[A] D-galactosamine	12 (8.8 - 24)	13.22 (± 4.06)	13 (9.6 - 21.2)	13.78 (± 3.64)	13.6 (11.2 - 16.4)	13.76 (± 1.97)
Liver	[B] Diethylhexylphthalate (DEHP)	8.1 (3.2 - 14.8)	8.44 (± 3.15)	13.2 (6.4 - 26)	14.04 (± 5.69)	14 (11 - 20.4)	14.8 (± 3.55)
Liver	[C] Dimethylformamide (DMF)	12 (9 - 16)	12.2 (± 1.93)	16 (10 - 26)	17 (± 5.27)	14 (11 - 18)	14.6 (± 2.61)
Liver	[C] Dimethylnitrosamine (DMN)	19 (8 - 36)	20 (± 9.71)	8.5 (4 - 30)	11 (± 8)	14 (2 - 22)	14 (± 7.65)
Liver	[A] Gadolinium chloride	11.3 (7 - 25)	12.9 (± 5.66)	12.6 (7.8 - 24.8)	13.98 (± 4.84)	15.4 (9.8 - 23.2)	15.48 (± 5.15)
Liver	[A, B, C, D, F] Hydrazine	11 (4.5 - 66)	14.64 (± 11.46)	11.2 (4.8 - 40)	12.77 (± 7)	12 (6.8 - 35.79)	13.75 (± 7.45)
Liver	[E] Hydrazine	13.75 (9 - 25)	14.15 (± 4.84)	7.5 (6 - 12.5)	8 (± 2.21)	13 (4 - 27)	14.8 (± 8.93)
Liver	[E] Indomethacin	6.25 (4.5 - 10.5)	6.75 (± 1.89)	11 (7.5 - 16)	11.45 (± 3.05)	10.34 (6 - 32)	17.27 (± 13.1)
Liver	[E] Ketoconazole	10.75 (6.5 - 16)	10.9 (± 3.49)	10.75 (8 - 23)	12.33 (± 4.61)	16 (10.5 - 17)	15.1 (± 2.7)
Liver	[C] Lead acetate	8 (6 - 20)	10.1 (± 4.33)	9.43 (3 - 18)	10.09 (± 4.53)	20 (12 - 22)	17.8 (± 4.49)
Liver	[A] Lipopolysaccharide (LPS)	13.15 (10.1 - 22.4)	14.76 (± 4.29)	11.75 (7.8 - 16.8)	12.2 (± 2.75)	12.3 (9.4 - 13.4)	12.06 (± 1.62)
Liver	[B] Methapyrilene	15.4 (2.8 - 20)	13 (± 5.82)	16.4 (3.4 - 19.6)	13.84 (± 5.69)	11.6 (10.8 - 12.2)	11.48 (± 0.58)
Liver	[E] Methylene dianiline	9 (6 - 11.5)	8.7 (± 1.95)	12.75 (8.5 - 21)	13 (± 3.76)	9.5 (8.5 - 18)	11.9 (± 4.02)
Liver	[C] Monocrotaline	17.5 (8 - 39)	19.1 (± 8.66)	13 (10 - 29)	14.9 (± 6.05)	13 (10 - 20)	14 (± 3.67)
Liver	[C] N-methylformamide (NMF)	18 (8 - 28)	17.4 (± 6.04)	29 (20 - 46)	31.6 (± 8.93)	16 (14 - 16)	15.2 (± 1.1)
Liver	[D] Phalloidin (chronic)	10.65 (6.8 - 13.5)	10.54 (± 2.08)	9.75 (8 - 14.5)	10.45 (± 2.17)	12.97 (12.27 - 16.32)	13.86 (± 1.76)
Liver	[E] Phenyl diisothiocyanate	3.75 (2 - 9)	4.1 (± 1.9)	2.25 (1.5 - 9.5)	3.1 (± 2.38)	13.22 (10.34 - 14)	12.28 (± 1.73)
Liver	[E] Phenyl isothiocyanate	8 (4 - 24)	10.5 (± 6.41)	19 (7 - 31)	19.2 (± 7.5)	17 (13.5 - 31)	19.6 (± 6.78)

Target organ	Toxin	24 h post dose		48 h post dose		168 h post dose	
		Median (min - max)	Mean (± SD)	Median (min - max)	Mean (± SD)	Median (min - max)	Mean (± SD)
Liver	[B] Retinyl palmitate	12.7 (8.8 - 23.2)	14.49 (± 5.46)	11.1 (7 - 13.8)	10.92 (± 2.27)	14.2 (9.8 - 31.4)	16.6 (± 8.54)
Liver	[B] Sodium Valproate	12.6 (6.4 - 39.6)	20.5 (± 13.61)	14.8 (2 - 27.4)	15.46 (± 8.77)	11.4 (5 - 18.4)	11.9 (± 4.93)
Liver	[C] a-Naphthylisothiocyanate (ANIT)	11.92 (6 - 31.47)	16.08 (± 9.89)	10.5 (7 - 42.95)	13.94 (± 10.62)	16 (12 - 20)	15.8 (± 3.03)
Kidney	[D] 2-Bromophenol	8 (6 - 15)	9.35 (± 3.11)	8.5 (6.5 - 13)	8.9 (± 2.12)	13 (11 - 18)	14.2 (± 3.11)
Kidney	[E] 3,5-Dichloroaniline hydrochloride	5.5 (4.5 - 11)	6.1 (± 2.01)	8 (5 - 14)	8.5 (± 2.6)	14 (10 - 24)	15.3 (± 5.26)
Kidney	[E] Atractyloside	25 (9.5 - 34)	24.3 (± 6.98)	25.69 (11 - 48)	27.18 (± 12.21)	19 (9 - 27)	18.8 (± 6.42)
Kidney	[D] Bromoethylamine hydrobromide	20 (17 - 23)	20 (± 1.89)	41 (16 - 62)	38.8 (± 12.33)	34 (26 - 38)	32.4 (± 4.56)
Kidney	[D] Cephaloridine	10 (5.5 - 24)	12.55 (± 6.04)	12.5 (6 - 26)	14.05 (± 7.33)	11 (10 - 18)	12.6 (± 3.21)
Kidney	[B] Chlorethanamine	18.4 (7 - 29.2)	18.26 (± 6.57)	29.7 (13 - 46)	30.78 (± 10.7)	23.4 (18.4 - 25.2)	22.28 (± 3.1)
Kidney	[A] Cisplatin	16 (7.6 - 30)	16.42 (± 7.83)	9 (4.2 - 19)	10 (± 4.96)	24 (10 - 30)	23 (± 7.68)
Kidney	[A] D-limonene (chronic)	8.5 (7 - 12)	9.1 (± 1.66)	10 (7 - 16)	10 (± 2.49)	13 (9 - 17)	13 (± 3.54)
Kidney	[E] Dichlorophenyl succinimide	7.25 (6 - 11.5)	8.25 (± 2.02)	12 (6.5 - 15)	11.95 (± 2.86)	13 (7.5 - 15)	12.3 (± 2.91)
Kidney	[D] Ethylene glycol	17 (13 - 30)	18.2 (± 5.22)	13.5 (9 - 21)	13.6 (± 3.63)	17 (12 - 18)	15.8 (± 2.39)
Kidney	[A] Folic acid	11.5 (3.6 - 15)	10.66 (± 3.85)	14.5 (2 - 28)	15.7 (± 7.69)	12.6 (12 - 15)	13.41 (± 1.47)
Kidney	[A] Gentamicin	11 (10 - 16)	11.4 (± 2.01)	11.5 (8.2 - 16)	11.46 (± 2.29)	11 (10 - 18)	13 (± 3.74)
Kidney	[B] Maleic acid	13.9 (6.4 - 30)	14.62 (± 7.06)	13.3 (7.6 - 22.4)	15 (± 4.13)	16.8 (12 - 18)	16.08 (± 2.41)
Kidney	[A] N-phenylanthranilic acid (chronic)	24.8 (16.4 - 33)	24.34 (± 4.8)	24 (7 - 33)	22.76 (± 8.36)	15.7 (12.6 - 21.8)	16.3 (± 3.43)
Kidney	[D] Para-aminophenol	19 (10 - 28)	19.1 (± 6.1)	23 (12 - 34)	21.8 (± 6.2)	20 (16 - 24)	20 (± 3.16)
Kidney	[A] Puromycin	7.9 (4 - 24)	12.42 (± 8.65)	10 (8 - 19)	11.53 (± 3.72)	15 (10 - 18)	14 (± 3.08)
Kidney	[B] Vancomycin hydrochloride	12.4 (3.8 - 15)	10.8 (± 4.2)	16.7 (4.4 - 22)	15.14 (± 5.31)	13.2 (11.4 - 14.2)	13.16 (± 1.11)
Liver & Kidney	[E] Acetaminophen	9.25 (4.5 - 15)	8.75 (± 3.23)	9.5 (3.5 - 16)	9.2 (± 3.34)	13.5 (10 - 20)	15.1 (± 4.25)

Target organ	Toxin	24 h post dose		48 h post dose		168 h post dose	
		Median (min - max)	Mean (± SD)	Median (min - max)	Mean (± SD)	Median (min - max)	Mean (± SD)
Liver & Kidney	[B] Aurothiomalate	18.5 (8.4 - 49)	22.28 (± 13)	11.79 (1.6 - 46)	15.4 (± 14.26)	19.8 (14.8 - 20.4)	18.48 (± 2.37)
Liver & Kidney	[C] Chloroform	13.5 (7 - 26)	14.7 (± 6.25)	15.33 (10 - 23)	15.67 (± 4.47)	14 (14 - 18)	15.2 (± 1.79)
Liver & Kidney	[D] Cyclosporin	22.5 (7 - 32)	21.1 (± 7.26)	16.5 (11 - 50)	21.67 (± 12.1)	18 (12.27 - 19)	16.65 (± 2.95)
Liver & Kidney	[D] Dichlorobenzene	6.1 (4 - 20)	9.37 (± 6.18)	14.5 (10 - 39)	16.7 (± 8.33)	14 (9.5 - 17)	13.5 (± 3.2)
Liver & Kidney	[C] Ethionine	17.15 (8 - 34.1)	16.49 (± 7.33)	19 (16 - 32)	21.6 (± 6.31)	12 (11 - 15)	12.6 (± 1.52)
Liver & Kidney	[B] Hexachlorobutadiene (HCBD)	17.25 (13.2 - 29.8)	18.81 (± 5.26)	15.4 (13.4 - 19.4)	15.76 (± 1.95)	18.2 (16 - 21.8)	19.08 (± 2.39)
Liver & Kidney	[B] Mercuric chloride	17.1 (9.8 - 34)	20.06 (± 7.7)	18.9 (1.3 - 32.4)	18.65 (± 9.38)	17.6 (6.8 - 40.2)	22.15 (± 13.15)
Liver & Kidney	[E] Microcystin-LR	13 (9 - 19)	13.5 (± 2.76)	11 (1.5 - 20)	11.15 (± 5.09)	15 (12 - 17)	14.66 (± 2.02)
Liver & Kidney	[E] Rotenone	13.5 (1.5 - 50)	15.25 (± 13.57)	11.12 (4 - 35.29)	12.9 (± 8.5)	15.16 (10.34 - 26)	17.31 (± 6.35)
Liver & Kidney	[E] S-(1,2-dichlorovinyl)-cysteine (DCVC)	11 (6 - 26)	12.15 (± 5.53)	12.75 (9 - 23)	13.95 (± 4.59)	13 (10.5 - 21)	14.1 (± 4.02)
Liver & Kidney	[D] Thioacetamide	18 (6 - 38)	19.6 (± 10.21)	16 (8 - 40)	17.4 (± 9.06)	14 (10 - 17)	13.8 (± 2.86)
Pancreas	[E] 1-Cyano-2-hydroxy-3-butene	10.25 (5 - 15)	9.85 (± 3.34)	6.75 (5 - 14)	7.25 (± 2.54)	8 (5.5 - 13)	8.7 (± 2.8)
Pancreas	[C] Caerulin	9.5 (8 - 20)	11.1 (± 4.15)	10 (8 - 16)	10.39 (± 2.41)	14 (10 - 21)	15.17 (± 4.1)
Pancreas	[E] L-arginine	11.28 (5 - 22)	12.75 (± 4.38)	5.5 (3 - 14)	6.3 (± 3.02)	14 (6.5 - 28)	15.3 (± 6.89)
Pancreas	[B] Streptozotocin	27.25 (8.4 - 49)	28.64 (± 13.79)	65.65 (13 - 119)	64.13 (± 31.65)	154 (62 - 186)	141.8 (± 47.19)
Testicular	[D] 1,3-Dinitrobenzene	9 (6.8 - 37)	12.53 (± 9.06)	17.5 (9 - 38)	19.7 (± 8.6)	21 (13 - 25)	19 (± 5.66)
Testicular	[C] Cadmium chloride	7 (4 - 10)	6.7 (± 2)	12 (5 - 24)	12.6 (± 5.32)	14 (12 - 17)	14.4 (± 1.82)
Testicular	[D] Cadmium chloride	15 (8 - 26)	17.06 (± 6.19)	22.5 (7.5 - 42)	22.15 (± 9.09)	20 (13 - 35)	21.4 (± 8.2)
Testicular	[D] Carbendazim	8.1 (6.1 - 10)	8.1 (± 1.14)	12 (8 - 18)	12.39 (± 3.14)	18 (15 - 31)	20.6 (± 6.35)
Testicular	[D] Di-n-pentyl-phthalate	22.5 (8 - 34)	22.75 (± 8.66)	12.8 (9 - 17)	12.83 (± 2.77)	17.5 (14.9 - 22.5)	18.08 (± 3.37)
Testicular	[D] Ethane dimethane sulfonate (EDS)	22 (8.8 - 79)	25.89 (± 20.66)	13.5 (10 - 26)	15.2 (± 4.96)	12 (9.5 - 17)	12.9 (± 2.79)

Target organ	Toxin	24 h post dose		48 h post dose		168 h post dose	
		Median (min - max)	Mean (± SD)	Median (min - max)	Mean (± SD)	Median (min - max)	Mean (± SD)
Testicular	[D] Methoxyacetic acid	11.25 (6.3 - 16)	11.3 (± 2.77)	11.05 (8.4 - 13.4)	11 (± 1.7)	10 (9.5 - 14)	10.9 (± 1.82)
Multiple organ	[B] Adriamycin	23.1 (12.4 - 61)	26 (± 14.52)	17.9 (15.6 - 39.2)	24.68 (± 10)	8.6 (4 - 12.6)	8.56 (± 3.17)
Multiple organ	[C] Amphotericin B	14 (10 - 22)	14.9 (± 4.12)	18 (9.32 - 34)	19.23 (± 7.84)	20 (12 - 27)	21 (± 6)
Multiple organ	[C] Azaserine	37 (12 - 67)	34.6 (± 17.38)	27 (12 - 64)	32.6 (± 17.96)	20 (10 - 37)	22.6 (± 11.57)
Multiple organ	[A] Dexamethasone	19.5 (9 - 35)	19.6 (± 7.15)	21 (8 - 52)	27.6 (± 16.24)	18 (12 - 72)	32.8 (± 26.33)
Multiple organ	[E] Mitomycin-C	10.25 (4 - 20)	11.35 (± 4.67)	10 (6 - 15)	10.6 (± 3.48)	19 (14 - 21)	17.6 (± 2.97)
Physiological stressor	[C] 1,1-Dichloroethylene & maleic acid	11 (7 - 12)	10.1 (± 1.73)	12 (8 - 36)	14.5 (± 8.34)	12 (8 - 15)	11 (± 3)
Physiological stressor	[C] 2,4-Dinitrophenol	11 (5 - 12)	9.7 (± 2.26)	10 (8 - 15)	10.6 (± 2.37)	13 (12 - 20)	14 (± 3.39)
Physiological stressor	[B] 4-Pentenoic acid	10.7 (3.2 - 14.8)	10.2 (± 4.58)	11.5 (4.4 - 15.6)	11.18 (± 3.93)	11.8 (2.6 - 17)	11.08 (± 5.25)
Physiological stressor	[D] Acetazolamide	17 (11 - 25)	16.8 (± 4.59)	15 (14 - 20)	16 (± 2.31)	12 (11 - 14)	12 (± 1.22)
Physiological stressor	[C] Acivicin	10 (6 - 15)	10.6 (± 2.8)	10.5 (6 - 14)	10 (± 2.79)	15 (10 - 16)	14 (± 2.55)
Physiological stressor	[E] Ammonium chloride	15 (11 - 22)	15.5 (± 3.1)	14.5 (10 - 20)	14.3 (± 2.75)	13.22 (10.34 - 13.36)	12.48 (± 1.3)
Physiological stressor	[D] Carboplatin	21 (10 - 64)	30.3 (± 21)	17.5 (10 - 44)	19.5 (± 10.69)	21 (13 - 28)	21.8 (± 6.38)
Physiological stressor	[A] Choline and choline/methionine deficiency (chronic)	5.65 (3.4 - 9.9)	5.98 (± 1.75)	7.7 (5.8 - 13)	8.26 (± 2.3)	9 (8 - 11)	9.2 (± 1.3)
Physiological stressor	[B] Food restriction (chronic)	22.6 (12.4 - 30)	20.84 (± 5.96)	16.4 (4.4 - 39)	18.13 (± 11.81)	11.8 (7 - 16.4)	11.64 (± 3.64)
Physiological stressor	[D] Furosemide	8.2 (6.4 - 14)	9.46 (± 2.61)	11.5 (8.2 - 15)	12.02 (± 2.37)	16 (14 - 22)	16.4 (± 3.29)
Physiological stressor	[B] Insulin	11.9 (9.4 - 14.9)	12.26 (± 1.71)	13.7 (11 - 17.2)	13.93 (± 2.07)	14 (12 - 21.4)	14.88 (± 3.78)
Physiological stressor	[E] Methotrexate	12.75 (9.5 - 18)	13.25 (± 3.08)	13 (8 - 20)	13.5 (± 4.25)	12 (12 - 21)	14.2 (± 3.9)
Physiological stressor	[A] Partial hepatectomy	6.25 (4 - 13)	7.58 (± 2.83)	10 (4 - 17.5)	10.05 (± 4.46)	11.5 (7 - 17)	11.5 (± 4.34)
Physiological stressor	[A] Phenobarbital (chronic)	9 (7.5 - 11)	9 (± 1.18)	10 (6 - 16)	10.5 (± 2.64)	17 (14 - 20)	17.2 (± 2.39)
Physiological stressor	[A] Pregnenolone 16 alpha carbonitrile (chronic)	10 (8 - 16)	10.4 (± 2.41)	11 (10 - 14)	11.6 (± 1.71)	19 (12 - 26)	18.8 (± 6.76)

Target organ	Toxin	24 h post dose		48 h post dose		168 h post dose	
		Median (min - max)	Mean (± SD)	Median (min - max)	Mean (± SD)	Median (min - max)	Mean (± SD)
Physiological stressor	[A] Probenecid	11.6 (7.3 - 25)	13.51 (± 4.98)	11 (9.3 - 20.6)	12.97 (± 3.72)	14.7 (12.8 - 15.5)	14.48 (± 1.02)
Physiological stressor	[C] Rosiglitazone	12.5 (8 - 22)	13.7 (± 4.27)	12.5 (7 - 24)	13.2 (± 4.39)	16 (9 - 21)	15.4 (± 4.39)
Physiological stressor	[C] Rosiglitazone (chronic)	12 (8 - 30)	13.3 (± 6.07)	13 (10 - 16)	12.9 (± 1.66)	14 (12 - 18)	14.75 (± 2.63)
Physiological stressor	[E] Sodium bicarbonate	22.5 (9.5 - 26)	20.15 (± 6.07)	20 (9 - 27)	19.3 (± 4.85)	18 (11 - 32)	18.2 (± 5.9)
Physiological stressor	[A] Unilateral nephrectomy	6 (4 - 11)	6.8 (± 2.75)	11.75 (6 - 18)	11.65 (± 3.6)	17.5 (15 - 19.5)	17.5 (± 1.7)
Physiological stressor	[B] Water deprivation (chronic)	7.7 (6.4 - 12.46)	8.38 (± 1.98)	4.3 (3.6 - 14.32)	5.53 (± 3.13)	15.3 (10.8 - 17.2)	14.86 (± 2.35)
No Effect	[E] Acetaminophen (chronic)	12 (9 - 26)	14.2 (± 6.31)	11 (8.5 - 22)	12 (± 3.79)	15.5 (11.5 - 36.5)	20.75 (± 9.78)
No Effect	[C] Buthionine sulphoxime	12 (9 - 15)	12 (± 1.76)	13.5 (10 - 21)	13.9 (± 3.93)	18 (12 - 20)	16.4 (± 3.29)
No Effect	[C] Ferrous sulphate	8 (5 - 11)	7.9 (± 1.97)	12 (8 - 14)	11.5 (± 1.84)	13 (10 - 14)	12.2 (± 1.64)
No Effect	[B] Ifosfamide	9.18 (6.4 - 36.9)	16.91 (± 13.24)	9.7 (1.4 - 30.8)	12.4 (± 9.08)	12.6 (11 - 24.8)	14.68 (± 5.72)
No Effect	[B] Lithocholic acid	10.7 (7.2 - 16.8)	11.48 (± 3.54)	14.3 (10.8 - 20.6)	14.9 (± 3.13)	16.4 (12.6 - 21.6)	16.44 (± 3.49)
No Effect	[E] Paraquat	9.88 (6 - 19)	11.18 (± 3.69)	11.88 (9.5 - 15.5)	12.18 (± 1.74)	15 (9.5 - 19)	15.3 (± 3.96)
No Effect	[D] Potassium dichromate	11.5 (7 - 32)	13.4 (± 7)	14.5 (7 - 28)	15.1 (± 6.37)	18 (11 - 29)	18.4 (± 6.88)
No Effect	[C] Trichlorethylene	10.5 (9 - 12)	10.7 (± 1.25)	10 (8 - 18)	11.5 (± 3.34)	15 (12 - 16)	14.4 (± 1.82)

A-F: Indicates Pharmaceutical Company & sample origin

Supplementary Table S33. Summary statistics for urine pH at 24 hrs, 48 hrs and 168 hrs post high dose.

Target organ	Toxin	24 h post dose		48 h post dose		168 h post dose	
		Median (min - max)	Mean (± SD)	Median (min - max)	Mean (± SD)	Median (min - max)	Mean (± SD)
Liver	[E] 1,1-Dichloroethylene	6.05 (5.8 - 6.6)	6.12 (± 0.28)	6.85 (6.3 - 7.4)	6.87 (± 0.32)	7.1 (6.9 - 7.4)	7.14 (± 0.18)
Liver	[E] 1,2,3,4,5,6-hexachlorocyclohexane	6.65 (6.1 - 7.1)	6.64 (± 0.3)	6.65 (6.3 - 7.3)	6.7 (± 0.29)	6.7 (6.5 - 7.1)	6.74 (± 0.23)

Target organ	Toxin	24 h post dose		48 h post dose		168 h post dose	
		Median (min - max)	Mean (± SD)	Median (min - max)	Mean (± SD)	Median (min - max)	Mean (± SD)
Liver	[B] 1-Fluoropentane	6 (6 - 6)	6 (± 0)	7 (6.5 - 7)	6.95 (± 0.16)	8 (8 - 8)	8 (± 0)
Liver	[B] 2,4,6-Trihydroxyacetophenone (THA)	6.5 (5 - 7)	6.45 (± 0.6)	7 (6 - 7)	6.75 (± 0.35)	8 (7 - 8)	7.8 (± 0.45)
Liver	[B] 4-Amino-2,6-dichlorophenol (ADCP)	6.5 (6.5 - 7)	6.65 (± 0.24)	7 (6 - 8)	7.05 (± 0.76)	8 (7 - 8)	7.8 (± 0.45)
Liver	[C] Aflatoxin	7.5 (6 - 8)	7.3 (± 0.59)	7 (6.5 - 8)	7.15 (± 0.47)	7.5 (7 - 7.5)	7.3 (± 0.27)
Liver	[C] Allyl alcohol	7.5 (7 - 8)	7.4 (± 0.32)	8 (7.5 - 8.5)	8 (± 0.35)	7.5 (7 - 7.5)	7.3 (± 0.27)
Liver	[C] Allyl formate	7.5 (7 - 8)	7.45 (± 0.28)	7.75 (6.5 - 8.5)	7.54 (± 0.69)	7.74 (7 - 8)	7.65 (± 0.42)
Liver	[B] Azathioprine	7.5 (6.5 - 8)	7.45 (± 0.6)	6.5 (6 - 6.5)	6.4 (± 0.21)	8 (7 - 8.5)	7.9 (± 0.55)
Liver	[B] Bromobenzene	6.5 (6 - 7)	6.55 (± 0.44)	6.5 (5 - 7)	6.5 (± 0.58)	8 (7 - 8)	7.6 (± 0.55)
Liver	[C] Butylated hydroxytoluene	7 (6.5 - 7.5)	7 (± 0.33)	6.5 (5.5 - 6.5)	6.3 (± 0.35)	7 (7 - 7.5)	7.2 (± 0.27)
Liver	[D] Carbon tetrachloride	6 (6 - 6)	6 (± 0)	6 (5 - 7)	6 (± 0.47)	8 (7 - 8)	7.8 (± 0.45)
Liver	[C] Chlorpromazine	6.75 (6.5 - 7)	6.75 (± 0.26)	7.5 (6 - 8)	7.55 (± 0.6)	8 (7.5 - 8)	7.8 (± 0.27)
Liver	[B] Clofibrate	7 (6.5 - 9)	7 (± 0.75)	7 (7 - 8)	7.3 (± 0.48)	9 (8 - 9)	8.6 (± 0.55)
Liver	[B] Cyproterone acetate	8 (7 - 8)	7.7 (± 0.48)	6.75 (6 - 7)	6.6 (± 0.46)	7 (7 - 8)	7.2 (± 0.45)
Liver	[A] D-galactosamine	6.5 (6.5 - 7)	6.6 (± 0.21)	7 (6.5 - 7.5)	6.9 (± 0.39)	7.5 (6.5 - 7.5)	7.2 (± 0.45)
Liver	[B] Diethylhexylphthalate (DEHP)	6 (5 - 6.5)	5.65 (± 0.58)	6 (5 - 6.5)	6 (± 0.41)	8 (7 - 8)	7.8 (± 0.45)
Liver	[C] Dimethylformamide (DMF)	7.5 (7.5 - 8)	7.65 (± 0.24)	7.5 (7 - 7.5)	7.3 (± 0.26)	7.5 (7 - 8)	7.5 (± 0.35)
Liver	[C] Dimethylnitrosamine (DMN)	7.5 (7 - 8)	7.45 (± 0.28)	6.5 (6.5 - 7.5)	6.7 (± 0.35)	7.5 (7 - 7.77)	7.45 (± 0.28)
Liver	[A] Gadolinium chloride	7 (6.5 - 7.5)	7.05 (± 0.28)	7.25 (7 - 7.5)	7.25 (± 0.26)	7.5 (7 - 7.5)	7.4 (± 0.22)
Liver	[A, B, C, D, F] Hydrazine	7 (6 - 8)	6.98 (± 0.49)	7 (6 - 8.5)	6.99 (± 0.59)	8 (6.5 - 9)	8.02 (± 0.67)
Liver	[E] Hydrazine	6 (6 - 7)	6.3 (± 0.48)	6 (6 - 7)	6.4 (± 0.52)	8 (7 - 8)	7.6 (± 0.55)
Liver	[E] Indomethacin	6.7 (6.3 - 7.6)	6.81 (± 0.41)	6.65 (6.3 - 7.3)	6.77 (± 0.37)	7.11 (6.4 - 8.2)	7.34 (± 0.75)

Target organ	Toxin	24 h post dose		48 h post dose		168 h post dose	
		Median (min - max)	Mean (± SD)	Median (min - max)	Mean (± SD)	Median (min - max)	Mean (± SD)
Liver	[E] Ketoconazole	7.2 (6.4 - 7.6)	7.15 (± 0.42)	6.9 (6.6 - 7.5)	7 (± 0.28)	7.7 (7.1 - 8.1)	7.66 (± 0.36)
Liver	[C] Lead acetate	7.25 (7 - 7.5)	7.25 (± 0.26)	7 (6.5 - 7.5)	7.01 (± 0.46)	7.5 (7.5 - 8)	7.6 (± 0.22)
Liver	[A] Lipopolysaccharide (LPS)	6.5 (6 - 7.08)	6.51 (± 0.26)	7 (6.5 - 7.17)	6.82 (± 0.28)	7.5 (7 - 7.5)	7.3 (± 0.27)
Liver	[B] Methapyrilene	7.1 (6.41 - 7.4)	7.03 (± 0.27)	7.62 (6.91 - 8)	7.5 (± 0.39)	7.2 (7.15 - 7.5)	7.28 (± 0.14)
Liver	[E] Methylene dianiline	6.4 (6.2 - 6.7)	6.42 (± 0.18)	7.6 (6.7 - 7.8)	7.52 (± 0.32)	7.5 (7.3 - 8)	7.62 (± 0.31)
Liver	[C] Monocrotaline	8 (7 - 8)	7.75 (± 0.35)	7.5 (7 - 8)	7.45 (± 0.37)	8 (7.5 - 8)	7.9 (± 0.22)
Liver	[C] N-methylformamide (NMF)	7.25 (6.5 - 8)	7.25 (± 0.42)	7.5 (7 - 7.5)	7.35 (± 0.24)	8 (7.5 - 8.5)	8 (± 0.35)
Liver	[D] Phalloidin (chronic)	6.99 (6.85 - 7.53)	7.12 (± 0.26)	7.18 (6.98 - 7.74)	7.25 (± 0.28)	7.18 (7.1 - 7.46)	7.24 (± 0.14)
Liver	[E] Phenyl diisothiocyanate	6.3 (6.1 - 7)	6.35 (± 0.26)	6 (5.7 - 7)	6.1 (± 0.42)	7.45 (7.11 - 7.9)	7.52 (± 0.3)
Liver	[E] Phenyl isothiocyanate	6.5 (5.6 - 6.8)	6.38 (± 0.4)	6.25 (5.8 - 7.1)	6.39 (± 0.46)	7.4 (7.2 - 7.5)	7.36 (± 0.11)
Liver	[B] Retinyl palmitate	6.5 (6 - 8)	6.67 (± 0.65)	6.75 (6 - 8)	6.7 (± 0.63)	7 (7 - 8)	7.4 (± 0.55)
Liver	[B] Sodium Valproate	7 (5 - 8)	6.9 (± 0.84)	6.7 (6 - 8)	6.74 (± 0.59)	7.35 (7 - 9)	7.67 (± 0.85)
Liver	[C] a-Naphthylisothiocyanate (ANIT)	7.12 (6 - 7.51)	6.91 (± 0.5)	6.25 (6 - 7.65)	6.43 (± 0.57)	8 (8 - 8.5)	8.2 (± 0.27)
Kidney	[D] 2-Bromophenol	7.16 (6.52 - 7.39)	7.12 (± 0.25)	7.37 (7.14 - 7.67)	7.38 (± 0.2)	7.5 (7.05 - 7.74)	7.49 (± 0.28)
Kidney	[E] 3,5-Dichloroaniline hydrochloride	6.15 (6 - 7)	6.27 (± 0.31)	7.1 (6.2 - 7.5)	7.04 (± 0.42)	7.7 (7.5 - 8.2)	7.82 (± 0.28)
Kidney	[E] Atractyloside	7 (6.7 - 7.4)	7.02 (± 0.25)	6.83 (6.4 - 7.34)	6.81 (± 0.27)	6.7 (6 - 6.9)	6.59 (± 0.35)
Kidney	[D] Bromoethylamine hydrobromide	6.25 (6.1 - 6.7)	6.38 (± 0.24)	6.7 (6.2 - 7.1)	6.69 (± 0.25)	6.9 (6.5 - 7.5)	6.88 (± 0.39)
Kidney	[D] Cephaloridine	6.78 (6.52 - 7.04)	6.77 (± 0.17)	6.94 (5.75 - 7.49)	6.85 (± 0.51)	7.48 (7.35 - 7.65)	7.5 (± 0.12)
Kidney	[B] Chlorethanamine	6 (6 - 6.5)	6.05 (± 0.16)	7 (6 - 7)	6.75 (± 0.35)	8 (7 - 8)	7.8 (± 0.45)
Kidney	[A] Cisplatin	7.01 (6.56 - 7.36)	6.96 (± 0.27)	6.42 (5.95 - 6.89)	6.45 (± 0.29)	7.17 (6.58 - 7.67)	7.14 (± 0.51)
Kidney	[A] D-limonene (chronic)	6.46 (6.28 - 6.67)	6.47 (± 0.14)	7.18 (6.77 - 7.55)	7.14 (± 0.23)	7.38 (6.92 - 7.66)	7.34 (± 0.27)

Target organ	Toxin	24 h post dose		48 h post dose		168 h post dose	
		Median (min - max)	Mean (± SD)	Median (min - max)	Mean (± SD)	Median (min - max)	Mean (± SD)
Kidney	[E] Dichlorophenyl succinimide	6.55 (6.2 - 6.9)	6.59 (± 0.21)	7.55 (7.2 - 7.7)	7.54 (± 0.16)	7.5 (7 - 7.9)	7.48 (± 0.35)
Kidney	[D] Ethylene glycol	6.12 (5.64 - 6.64)	6.15 (± 0.32)	7.09 (6.39 - 7.61)	7.1 (± 0.35)	7.11 (6.52 - 7.17)	6.96 (± 0.28)
Kidney	[A] Folic acid	7.41 (6.45 - 7.75)	7.29 (± 0.46)	7.12 (6.25 - 7.56)	7 (± 0.49)	7.25 (7.12 - 7.42)	7.26 (± 0.12)
Kidney	[A] Gentamicin	6.84 (6.49 - 7.3)	6.88 (± 0.28)	6.49 (5.92 - 7.05)	6.51 (± 0.43)	6.46 (6.4 - 6.96)	6.6 (± 0.25)
Kidney	[B] Maleic acid	6.95 (6.34 - 7.57)	6.9 (± 0.44)	6.93 (6.2 - 7.8)	6.92 (± 0.56)	7.5 (7.34 - 7.87)	7.6 (± 0.24)
Kidney	[A] N-phenylanthranilic acid (chronic)	7 (6.5 - 7.5)	6.85 (± 0.34)	7 (6 - 7)	6.75 (± 0.35)	7.14 (7 - 8)	7.3 (± 0.39)
Kidney	[D] Para-aminophenol	7.08 (6.78 - 7.5)	7.14 (± 0.26)	6.74 (6.21 - 7.04)	6.71 (± 0.24)	6.86 (6.76 - 6.99)	6.85 (± 0.1)
Kidney	[A] Puromycin	6.78 (6.15 - 7.16)	6.69 (± 0.34)	6.49 (6.13 - 7.08)	6.56 (± 0.32)	7.07 (6.93 - 7.38)	7.13 (± 0.21)
Kidney	[B] Vancomycin hydrochloride	7 (7 - 8.5)	7.35 (± 0.58)	8 (7 - 8.5)	8.05 (± 0.44)	7 (7 - 7)	7 (± 0)
Liver & Kidney	[E] Acetaminophen	6.15 (5.9 - 6.4)	6.13 (± 0.2)	6.35 (6 - 7.9)	6.55 (± 0.59)	7.3 (7 - 7.9)	7.4 (± 0.33)
Liver & Kidney	[B] Aurothiomalate	7 (6 - 8)	7.1 (± 0.57)	6.25 (6 - 7)	6.3 (± 0.35)	7 (6 - 7.64)	6.97 (± 0.6)
Liver & Kidney	[C] Chloroform	6.5 (6 - 7)	6.6 (± 0.32)	6.5 (6 - 7)	6.55 (± 0.28)	7.5 (7.5 - 8)	7.7 (± 0.27)
Liver & Kidney	[D] Cyclosporin	6.66 (6.22 - 7)	6.65 (± 0.24)	6.4 (6.02 - 7.09)	6.44 (± 0.36)	7.45 (7.04 - 7.7)	7.4 (± 0.24)
Liver & Kidney	[D] Dichlorobenzene	6.8 (6.27 - 7.11)	6.78 (± 0.23)	7.3 (6.91 - 7.54)	7.28 (± 0.2)	7.49 (7.07 - 7.62)	7.43 (± 0.21)
Liver & Kidney	[C] Ethionine	7 (6.5 - 8)	7.1 (± 0.46)	7 (6.5 - 7.5)	7.1 (± 0.32)	8 (7.5 - 8)	7.8 (± 0.27)
Liver & Kidney	[B] Hexachlorobutadiene (HCBD)	8 (7 - 8)	7.8 (± 0.42)	5 (5 - 6)	5.2 (± 0.42)	7 (6.5 - 8)	7.3 (± 0.67)
Liver & Kidney	[B] Mercuric chloride	7 (7 - 8)	7.4 (± 0.52)	7 (6 - 8)	6.8 (± 0.59)	7.7 (5 - 8)	6.94 (± 1.37)
Liver & Kidney	[E] Microcystin-LR	6.35 (6.1 - 7.8)	6.59 (± 0.59)	6.4 (6.1 - 7.9)	6.71 (± 0.66)	7.6 (7.32 - 7.7)	7.52 (± 0.16)
Liver & Kidney	[E] Rotenone	5.7 (5.2 - 6.7)	5.92 (± 0.51)	5.8 (5.4 - 7.41)	6.26 (± 0.78)	7.51 (7.11 - 8)	7.57 (± 0.37)
Liver & Kidney	[E] S-(1,2-dichlorovinyl)-cysteine (DCVC)	7.35 (6.7 - 8.8)	7.38 (± 0.57)	6.75 (6 - 8.6)	6.89 (± 0.73)	7.4 (7 - 7.7)	7.36 (± 0.27)
Liver & Kidney	[D] Thioacetamide	7.03 (6.48 - 7.18)	6.97 (± 0.2)	6.96 (6.3 - 7.1)	6.87 (± 0.27)	7.14 (6.82 - 7.45)	7.15 (± 0.26)

Target organ	Toxin	24 h post dose		48 h post dose		168 h post dose	
		Median (min - max)	Mean (± SD)	Median (min - max)	Mean (± SD)	Median (min - max)	Mean (± SD)
Pancreas	[E] 1-Cyano-2-hydroxy-3-butene	6.6 (6.1 - 6.9)	6.58 (± 0.23)	7.25 (5.7 - 7.8)	7.18 (± 0.59)	7.6 (6.3 - 7.9)	7.38 (± 0.62)
Pancreas	[C] Caerulin	6.5 (6.5 - 8)	6.75 (± 0.49)	7.5 (6.5 - 8)	7.48 (± 0.51)	7.5 (7 - 8)	7.49 (± 0.36)
Pancreas	[E] L-arginine	5.9 (5.6 - 6.98)	6.01 (± 0.38)	5.75 (5.5 - 7.8)	6.18 (± 0.77)	6.25 (5.5 - 7.4)	6.45 (± 0.61)
Pancreas	[B] Streptozotocin	7 (7 - 8)	7.1 (± 0.32)	7 (6.5 - 8)	7.15 (± 0.47)	7 (7 - 7)	7 (± 0)
Testicular	[D] 1,3-Dinitrobenzene	6.72 (6.35 - 7.32)	6.78 (± 0.33)	7.19 (6.66 - 7.51)	7.14 (± 0.28)	7.46 (6.77 - 7.54)	7.34 (± 0.32)
Testicular	[C] Cadmium chloride	7 (6.5 - 8.5)	7.15 (± 0.67)	8 (7 - 9)	8.05 (± 0.6)	7.5 (7.5 - 8)	7.7 (± 0.27)
Testicular	[D] Cadmium chloride	6.94 (6.54 - 7.79)	7.03 (± 0.34)	6.7 (6.44 - 7.39)	6.79 (± 0.3)	7.34 (7.13 - 7.61)	7.36 (± 0.17)
Testicular	[D] Carbendazim	6.73 (6.31 - 7.18)	6.7 (± 0.3)	7.39 (6.95 - 7.8)	7.32 (± 0.3)	7.18 (6.88 - 7.7)	7.26 (± 0.31)
Testicular	[D] Di-n-pentyl-phthalate	6.93 (6.18 - 7.32)	6.82 (± 0.34)	6.62 (6.1 - 6.87)	6.6 (± 0.23)	7.35 (7.14 - 7.45)	7.33 (± 0.12)
Testicular	[D] Ethane dimethane sulfonate (EDS)	7.15 (6.8 - 7.36)	7.12 (± 0.22)	6.85 (6.48 - 7.25)	6.86 (± 0.24)	7.28 (7.1 - 8.12)	7.45 (± 0.4)
Testicular	[D] Methoxyacetic acid	7.15 (6.87 - 7.56)	7.19 (± 0.24)	6.6 (6.43 - 7.06)	6.64 (± 0.2)	7.1 (6.61 - 7.45)	7.1 (± 0.35)
Multiple organ	[B] Adriamycin	7 (7 - 8)	7.2 (± 0.42)	7 (6.5 - 7)	6.95 (± 0.16)	8 (6.77 - 8)	7.55 (± 0.62)
Multiple organ	[C] Amphotericin B	6.5 (6.5 - 8)	6.8 (± 0.54)	7 (6.5 - 7.5)	6.92 (± 0.4)	7 (6.5 - 7)	6.8 (± 0.27)
Multiple organ	[C] Azaserine	7 (6.5 - 7.5)	6.9 (± 0.32)	6.75 (6.5 - 7.5)	6.8 (± 0.35)	7 (5.5 - 8)	6.9 (± 0.96)
Multiple organ	[A] Dexamethasone	6.54 (6.33 - 6.9)	6.55 (± 0.18)	7 (6.1 - 7.14)	6.91 (± 0.3)	7.55 (7 - 7.93)	7.43 (± 0.39)
Multiple organ	[E] Mitomycin-C	6.95 (6.5 - 7.2)	6.86 (± 0.25)	6.85 (6.5 - 7.2)	6.84 (± 0.33)	7.5 (7.2 - 7.7)	7.48 (± 0.19)
Physiological stressor	[C] 1,1-Dichloroethylene & maleic acid	7.5 (6.5 - 8)	7.45 (± 0.5)	7.5 (7 - 8)	7.55 (± 0.37)	7.5 (7 - 7.5)	7.4 (± 0.22)
Physiological stressor	[C] 2,4-Dinitrophenol	7.5 (6.5 - 8)	7.35 (± 0.47)	7.75 (7 - 8)	7.55 (± 0.5)	7.5 (7 - 8)	7.6 (± 0.42)
Physiological stressor	[B] 4-Pentenoic acid	7 (6.5 - 8)	7.15 (± 0.47)	8 (7 - 8.5)	7.75 (± 0.54)	7 (7 - 7)	7 (± 0)
Physiological stressor	[D] Acetazolamide	7.51 (7.08 - 7.83)	7.5 (± 0.22)	6.7 (6.5 - 7.36)	6.76 (± 0.25)	7.09 (7.02 - 7.44)	7.18 (± 0.18)
Physiological stressor	[C] Acivicin	7.25 (7 - 8)	7.4 (± 0.46)	8 (7 - 8)	7.85 (± 0.34)	7.5 (7.5 - 7.5)	7.5 (± 0)

Target organ	Toxin	24 h post dose		48 h post dose		168 h post dose	
		Median (min - max)	Mean (± SD)	Median (min - max)	Mean (± SD)	Median (min - max)	Mean (± SD)
Physiological stressor	[E] Ammonium chloride	6 (5.9 - 6.5)	6.12 (± 0.23)	5.6 (5.5 - 5.7)	5.58 (± 0.06)	7.41 (7.11 - 7.5)	7.36 (± 0.15)
Physiological stressor	[D] Carboplatin	7.26 (7.03 - 7.46)	7.25 (± 0.14)	7 (6.81 - 7.16)	7.01 (± 0.12)	7.24 (7.11 - 7.55)	7.3 (± 0.18)
Physiological stressor	[A] Choline and choline/methionine deficiency (chronic)	6.5 (6 - 7.5)	6.7 (± 0.42)	6.5 (6.5 - 7)	6.65 (± 0.24)	7 (6.5 - 7)	6.8 (± 0.27)
Physiological stressor	[B] Food restriction (chronic)	7 (7 - 8)	7.44 (± 0.53)	7 (7 - 8)	7.37 (± 0.48)	8 (7 - 8)	7.8 (± 0.45)
Physiological stressor	[D] Furosemide	7.42 (7.18 - 7.95)	7.43 (± 0.22)	7.23 (7.11 - 7.85)	7.32 (± 0.24)	7.22 (7.05 - 7.71)	7.32 (± 0.31)
Physiological stressor	[B] Insulin	8 (7 - 8)	7.6 (± 0.52)	7 (7 - 8)	7.3 (± 0.48)	7 (7 - 8)	7.4 (± 0.55)
Physiological stressor	[E] Methotrexate	6.8 (6.6 - 7.2)	6.86 (± 0.21)	6.9 (6.6 - 7.5)	6.99 (± 0.3)	7.3 (6.8 - 7.7)	7.28 (± 0.35)
Physiological stressor	[A] Partial hepatectomy	7 (7 - 7.5)	7.2 (± 0.26)	8 (7.5 - 8)	7.85 (± 0.24)	8 (8 - 8)	8 (± 0)
Physiological stressor	[A] Phenobarbital (chronic)	7.12 (6.9 - 7.37)	7.11 (± 0.15)	7 (6.7 - 7.37)	6.98 (± 0.23)	6.92 (6.74 - 7.25)	6.94 (± 0.21)
Physiological stressor	[A] Pregnenolone 16 alpha carbonitrile (chronic)	6.88 (6.73 - 7.2)	6.91 (± 0.17)	6.99 (6.76 - 7.78)	7.08 (± 0.34)	7.2 (6.92 - 7.61)	7.21 (± 0.3)
Physiological stressor	[A] Probenecid	7.5 (7 - 8)	7.65 (± 0.34)	8 (7.5 - 8)	7.8 (± 0.26)	7.5 (7.5 - 8)	7.7 (± 0.27)
Physiological stressor	[C] Rosiglitazone	7.5 (7 - 8)	7.65 (± 0.34)	7.5 (6.5 - 8)	7.45 (± 0.44)	7.5 (7 - 8)	7.4 (± 0.42)
Physiological stressor	[C] Rosiglitazone (chronic)	7.5 (7 - 8)	7.4 (± 0.32)	7.5 (6.5 - 8)	7.45 (± 0.44)	7 (6.5 - 7.52)	7 (± 0.36)
Physiological stressor	[E] Sodium bicarbonate	8.5 (8.2 - 8.6)	8.48 (± 0.12)	8.6 (8.2 - 8.7)	8.53 (± 0.16)	8.2 (6.7 - 8.5)	7.96 (± 0.63)
Physiological stressor	[A] Unilateral nephrectomy	7 (6.5 - 7.5)	7.1 (± 0.32)	7.5 (7 - 7.5)	7.4 (± 0.21)	7 (7 - 7.5)	7.2 (± 0.27)
Physiological stressor	[B] Water deprivation (chronic)	7 (6.5 - 7.53)	6.92 (± 0.29)	6 (6 - 6.98)	6.22 (± 0.35)	8 (7 - 8.3)	7.72 (± 0.57)
No Effect	[E] Acetaminophen (chronic)	6.95 (6.7 - 7.4)	6.98 (± 0.25)	6.8 (6.7 - 7.2)	6.86 (± 0.15)	7 (6.7 - 7.3)	7.02 (± 0.17)
No Effect	[C] Buthionine sulphoxime	7 (6 - 7)	6.75 (± 0.35)	7 (6.5 - 7.5)	6.95 (± 0.44)	7.5 (7 - 7.5)	7.4 (± 0.22)
No Effect	[C] Ferrous sulphate	6.5 (6 - 7)	6.45 (± 0.37)	8 (6.5 - 8.5)	8 (± 0.62)	7 (7 - 8)	7.4 (± 0.55)
No Effect	[B] Ifosfamide	7 (6 - 8)	6.97 (± 0.63)	7 (6.5 - 8)	6.95 (± 0.44)	7 (7 - 8)	7.4 (± 0.55)
No Effect	[B] Lithocholic acid	8 (7 - 8)	7.6 (± 0.52)	7.5 (7 - 8)	7.5 (± 0.53)	7 (7 - 8)	7.4 (± 0.55)

Target organ	Toxin	24 h post dose		48 h post dose		168 h post dose	
		Median (min - max)	Mean (± SD)	Median (min - max)	Mean (± SD)	Median (min - max)	Mean (± SD)
No Effect	E Paraquat	7.2 (6.6 - 7.5)	7.11 (± 0.29)	7.15 (6.4 - 7.6)	7.08 (± 0.32)	7.5 (7.1 - 7.7)	7.46 (± 0.23)
No Effect	D Potassium dichromate	7.14 (6.75 - 7.39)	7.08 (± 0.26)	7.16 (6.91 - 7.6)	7.21 (± 0.21)	7.68 (7.24 - 7.85)	7.57 (± 0.27)
No Effect	C Trichlorethylene	7 (6.5 - 7)	6.9 (± 0.21)	7.25 (7 - 7.5)	7.25 (± 0.26)	7.5 (7 - 8)	7.5 (± 0.35)

A-F: Indicates Pharmaceutical Company & sample origin

Supplementary Table S34. Summary statistics for urine osmolality (mOsm/L) at 24 hrs, 48 hrs and 168 hrs post high dose.

Target organ	Toxin	24 h post dose		48 h post dose		168 h post dose	
		Median (min - max)	Mean (± SD)	Median (min - max)	Mean (± SD)	Median (min - max)	Mean (± SD)
Liver	E 1,1-Dichloroethylene	1534 (538 - 1906)	1287 (± 490.92)	1613 (883 - 2362)	1596.4 (± 448.85)	1722 (1437 - 2168)	1746.8 (± 272.56)
Liver	E 1,2,3,4,5,6-hexachlorocyclohexane	1591 (1209 - 2268)	1655 (± 333.06)	1499 (1114 - 1995)	1538.5 (± 303.74)	1462.53 (1019.8 - 1667.39)	1414.87 (± 238.16)
Liver	B 1-Fluoropentane	1177 (812 - 2088)	1250.6 (± 400.75)	1346 (490 - 1950)	1394.4 (± 414.86)	1866 (1538 - 3782)	2185.2 (± 904.07)
Liver	B 2,4,6-Trihydroxyacetophenone (THA)	1111 (670 - 2252)	1154.4 (± 466.71)	1774 (1144 - 2364)	1729.4 (± 318.39)	1538 (1292 - 1984)	1547.6 (± 265.84)
Liver	B 4-Amino-2,6-dichlorophenol (ADCP)	1130 (570 - 1866)	1146.2 (± 478)	1640 (1016 - 2446)	1657.4 (± 469.96)	1740 (1232 - 2284)	1804.8 (± 397.15)
Liver	C Aflatoxin	701.75 (376.9 - 1387.57)	820.04 (± 327.79)	673.95 (323.4 - 1153.6)	754.74 (± 258.82)	1601.6 (885.4 - 1752.55)	1414.68 (± 353.75)
Liver	C Allyl alcohol	747.65 (652 - 1065)	807.44 (± 159.65)	1237.05 (734.7 - 1431.5)	1227.84 (± 227.12)	1626.07 (1552.8 - 1732.75)	1643.74 (± 67.52)
Liver	C Allyl formate	1361.3 (910.1 - 1590.5)	1366.58 (± 197.46)	1423.74 (1203.99 - 1714.7)	1427.05 (± 132.15)	1674.45 (1393.4 - 1788.9)	1647.11 (± 149.79)
Liver	B Azathioprine	299 (174 - 852)	394.4 (± 233.5)	823 (338 - 1248)	820.4 (± 303.3)	1904 (618 - 2128)	1718.8 (± 628.23)
Liver	B Bromobenzene	1613 (1187 - 2198)	1616.2 (± 343.46)	1720 (1591 - 2382)	1786.9 (± 230.13)	1699 (1326 - 2408)	1885.4 (± 457.01)
Liver	C Butylated hydroxytoluene	1380.25 (779.8 - 1649.6)	1348.1 (± 250.8)	900.15 (834 - 1292.1)	967.02 (± 137.9)	1459.95 (1284.3 - 1591.3)	1442.12 (± 137.15)
Liver	D Carbon tetrachloride	1599 (645 - 1953)	1529.19 (± 415.09)	1560.7 (531 - 1801)	1435.84 (± 377.14)	1840 (1252 - 1922)	1707.53 (± 276.29)
Liver	C Chlorpromazine	1383.95 (1060.2 - 1523.97)	1383.16 (± 142.85)	1401.85 (1172.7 - 1534.3)	1395.04 (± 119.3)	1540.2 (1321.75 - 1758.1)	1532.43 (± 159.48)
Liver	B Clofibrate	796 (332 - 2071)	888.9 (± 556.01)	604.5 (401 - 1012)	643.4 (± 223.77)	1880 (1308 - 1980)	1739.8 (± 282.22)

Target organ	Toxin	24 h post dose		48 h post dose		168 h post dose	
		Median (min - max)	Mean (± SD)	Median (min - max)	Mean (± SD)	Median (min - max)	Mean (± SD)
Liver	[B] Cyproterone acetate	400 (138 - 988)	478.4 (± 287.95)	440 (130 - 3418)	784.2 (± 1000.76)	1234 (1106 - 1338)	1207.6 (± 99.18)
Liver	[A] D-galactosamine	1019.5 (368 - 1482)	1021.6 (± 279.63)	885.5 (533 - 1187)	838.2 (± 204.83)	1289 (1010 - 1725)	1332.6 (± 263.88)
Liver	[B] Diethylhexylphthalate (DEHP)	1473.5 (778 - 1873)	1423.1 (± 325.11)	1233 (877 - 1789)	1260.6 (± 257.65)	1874 (1191 - 1943)	1733.4 (± 314.4)
Liver	[C] Dimethylformamide (DMF)	1414.55 (1113.9 - 1467.95)	1373.62 (± 109.42)	1234.95 (769.8 - 1646.6)	1234.97 (± 237.24)	1544.5 (1282.85 - 1738.4)	1524.18 (± 208.93)
Liver	[C] Dimethylnitrosamine (DMN)	1073.21 (374.7 - 1454.98)	966.05 (± 375.04)	1348.78 (700.8 - 1602.8)	1277.62 (± 270.68)	1308.33 (984.58 - 1483.4)	1274.19 (± 206.94)
Liver	[A] Gadolinium chloride	1384 (710 - 1834)	1334.1 (± 326.73)	1222.5 (912 - 1648)	1274.7 (± 257.26)	1300 (549 - 1343)	1126.6 (± 333.1)
Liver	[A, B, C, D, F] Hydrazine	892.32 (288 - 1603.57)	933.82 (± 394.28)	1437 (449 - 2218)	1364.56 (± 424.37)	1534.35 (344 - 2231.9)	1307.34 (± 515.64)
Liver	[E] Hydrazine	950 (507 - 1413)	945.7 (± 239.35)	1810 (1295 - 2074)	1749.2 (± 256.07)	1017 (882 - 1874)	1203 (± 409.2)
Liver	[E] Indomethacin	1533 (1267 - 2462)	1733.1 (± 434.43)	1103.5 (928 - 1722)	1182 (± 280.1)	1551 (516 - 1781.7)	1242.74 (± 636.06)
Liver	[E] Ketoconazole	1677.5 (765 - 2158)	1578.9 (± 400.67)	1838 (987 - 2342)	1750.2 (± 450.1)	1677 (1381 - 1703)	1600.2 (± 136.52)
Liver	[C] Lead acetate	1318.45 (1216.7 - 1406.67)	1323.12 (± 62.05)	1410.3 (958.43 - 1646.6)	1376.1 (± 175.14)	1299 (1204.1 - 1732.75)	1379.67 (± 221.52)
Liver	[A] Lipopolysaccharide (LPS)	969.5 (646 - 1657.9)	1019.29 (± 311.08)	1144.5 (660 - 1774.8)	1134.68 (± 292.66)	1466 (1339 - 1700)	1482.2 (± 134.01)
Liver	[B] Methapyrilene	1761 (814 - 2306)	1728 (± 385.5)	1308 (724 - 1424)	1208.8 (± 240.78)	2102 (2060 - 2250)	2125.2 (± 76.61)
Liver	[E] Methylene dianiline	1449 (1142 - 2354)	1538 (± 357.43)	1450.5 (1156 - 2010)	1499.3 (± 300.63)	1900 (1242 - 2120)	1787.8 (± 377.9)
Liver	[C] Monocrotaline	1261.97 (700.4 - 1457.29)	1161.41 (± 293.46)	1336.45 (842.3 - 1605.2)	1330.31 (± 248.22)	1404.3 (1349.6 - 1852.4)	1519.18 (± 208.67)
Liver	[C] N-methylformamide (NMF)	1188.05 (701.7 - 1404.23)	1142.3 (± 229.28)	754.55 (603.8 - 1207.4)	827.72 (± 219.87)	1546.83 (1438.5 - 1630.9)	1528.49 (± 78.45)
Liver	[D] Phalloidin (chronic)	1756 (1291 - 2173)	1724.6 (± 312.67)	1661.5 (704 - 2022)	1550.5 (± 428.63)	1715.77 (1573.85 - 1823.9)	1701.94 (± 95.01)
Liver	[E] Phenyl diisothiocyanate	1208 (901 - 1451)	1209.4 (± 172.31)	1759.5 (1208 - 2360)	1811.6 (± 416.45)	1726.3 (1516 - 1930)	1719.72 (± 154.31)
Liver	[E] Phenyl isothiocyanate	1089 (445 - 2018)	1194 (± 507.35)	808.5 (425 - 1152)	810.5 (± 295.97)	1346 (816 - 1495)	1257.8 (± 269.77)
Liver	[B] Retinyl palmitate	750 (470 - 1549.9)	807.99 (± 354.14)	1019 (502 - 1624)	1037.2 (± 351.74)	1964 (700 - 2356)	1701.6 (± 653.58)
Liver	[B] Sodium Valproate	1285.5 (282 - 1887)	1193.57 (± 579.81)	1061 (651 - 1742)	1052.8 (± 323.46)	1705 (1587 - 2241.4)	1787.28 (± 258.99)
Liver	[C] a-Naphthylisothiocyanate (ANIT)	1289.25 (578.3 - 1576.8)	1161.56 (± 360.82)	1230.15 (645.5 - 1632.6)	1187.75 (± 302.15)	1219.9 (1015.1 - 1337.9)	1201.89 (± 118)

Target organ	Toxin	24 h post dose		48 h post dose		168 h post dose	
		**Median (min - max)**	**Mean (± SD)**	**Median (min - max)**	**Mean (± SD)**	**Median (min - max)**	**Mean (± SD)**
Kidney	[D] 2-Bromophenol	1606 (953 - 1923)	1549.5 (± 327.09)	1765 (1510 - 1967)	1747.18 (± 138.84)	1789 (1094 - 1901)	1577 (± 360.67)
Kidney	[E] 3,5-Dichloroaniline hydrochloride	1023.5 (492 - 1316)	1002 (± 269.2)	1673 (665 - 1919)	1549.1 (± 399.18)	1201 (1061 - 1978)	1358 (± 362.11)
Kidney	[E] Atractyloside	639 (519 - 1803)	745.27 (± 339.96)	653.5 (414 - 1740)	795.45 (± 393.58)	1203 (949 - 1984)	1334 (± 404.77)
Kidney	[D] Bromoethylamine hydrobromide	384 (329 - 455)	393.4 (± 33.61)	463 (327 - 562)	456.2 (± 62.11)	694 (591 - 943)	717.6 (± 133.49)
Kidney	[D] Cephaloridine	941.5 (463 - 1975)	1059.2 (± 473.34)	1243.5 (876 - 1698)	1265.24 (± 308.78)	1642 (1331 - 1704.8)	1574.56 (± 158.82)
Kidney	[B] Chlorethanamine	588.5 (505 - 1234)	686.5 (± 229.75)	577 (364 - 1309)	648.4 (± 280.43)	1164 (954 - 1307)	1138.8 (± 137.62)
Kidney	[A] Cisplatin	629.5 (400 - 1724)	805.5 (± 443.54)	1259 (630 - 1984)	1288.18 (± 424.1)	648 (518 - 1804.47)	857.09 (± 532.95)
Kidney	[A] D-limonene (chronic)	1515.75 (1267.8 - 1780)	1549.66 (± 174.77)	1708 (1495 - 1812)	1683.97 (± 102.67)	1701 (1653 - 1907)	1737.42 (± 99.83)
Kidney	[E] Dichlorophenyl succinimide	1621.5 (1122 - 1902)	1550.5 (± 251.14)	1498 (1290 - 2080)	1559.1 (± 272.06)	1702 (1533 - 2116)	1768.4 (± 234.55)
Kidney	[D] Ethylene glycol	1597 (1051 - 1947)	1602.53 (± 284.83)	1528 (985 - 1878)	1488.83 (± 291.48)	1683 (1279 - 1794)	1582.76 (± 211.05)
Kidney	[A] Folic acid	450 (334 - 1104)	618.2 (± 322.04)	433.5 (201 - 1672)	774.3 (± 556.43)	1715.7 (1236 - 1990)	1638.41 (± 311.78)
Kidney	[A] Gentamicin	1656.5 (1142 - 1978)	1622.12 (± 221.78)	1727.95 (1399 - 1956)	1684.42 (± 200.26)	1629.3 (1051 - 1803)	1493.86 (± 301.32)
Kidney	[B] Maleic acid	1061 (374 - 2210)	1153.1 (± 536.43)	1410 (886 - 2546)	1427.8 (± 436.26)	1498 (1414 - 2084)	1615.2 (± 272.7)
Kidney	[A] N-phenylanthranilic acid (chronic)	438 (387 - 713)	465.5 (± 97.7)	502.5 (401 - 761)	524.3 (± 124.61)	1151.5 (864 - 1845.34)	1288.81 (± 391.05)
Kidney	[D] Para-aminophenol	693.5 (351 - 918)	689.9 (± 177.83)	679 (411 - 1133)	716.5 (± 239.17)	1230 (742 - 1397)	1162.2 (± 269.27)
Kidney	[A] Puromycin	989.61 (614 - 1950)	1107.97 (± 468.96)	1513.75 (1097 - 1983)	1507.3 (± 278.69)	1695 (1152 - 1981)	1636.5 (± 307.69)
Kidney	[B] Vancomycin hydrochloride	1685.5 (1240 - 1871)	1642.5 (± 210.52)	1092 (775 - 1417)	1124.1 (± 188.95)	1818 (1735 - 1922)	1826.8 (± 85.12)
Liver & Kidney	[E] Acetaminophen	1129.5 (822 - 1756)	1191.7 (± 293.39)	1471.5 (358 - 2082)	1389.5 (± 462.03)	1533 (996 - 1675)	1408.6 (± 300.94)
Liver & Kidney	[B] Aurothiomalate	491 (290 - 716)	474.4 (± 145.74)	328 (262 - 1486.7)	466.27 (± 369.84)	1511.6 (400 - 3566)	1619.54 (± 1191)
Liver & Kidney	[C] Chloroform	1115.98 (688.5 - 1379.7)	1105.21 (± 220.45)	1171.5 (829.4 - 1532.7)	1160.13 (± 282.28)	1390.53 (1340.43 - 1465.8)	1395.96 (± 46.04)
Liver & Kidney	[D] Cyclosporin	569.5 (343 - 925)	579.7 (± 164.17)	778 (385 - 1717.6)	820.06 (± 376.64)	1253 (1184 - 1573.85)	1299.77 (± 158.89)
Liver & Kidney	[D] Dichlorobenzene	1410 (577 - 1916)	1310.5 (± 473.06)	1269 (583 - 1762.6)	1234.26 (± 374.69)	1729 (1511 - 1949)	1727.2 (± 178.16)

Target organ	Toxin	24 h post dose		48 h post dose		168 h post dose	
		Median (min - max)	Mean (± SD)	Median (min - max)	Mean (± SD)	Median (min - max)	Mean (± SD)
Liver & Kidney	[C] Ethionine	1250.85 (662 - 1606.65)	1204.16 (± 246.28)	1085.2 (622.1 - 1372.13)	1018.24 (± 244.26)	1657.4 (1597.8 - 1711.2)	1654.74 (± 47.46)
Liver & Kidney	[B] Hexachlorobutadiene (HCBD)	663 (361 - 1153)	686.4 (± 219.46)	519.5 (424 - 885)	604.6 (± 173.21)	1549 (1107 - 1747)	1461.8 (± 255.24)
Liver & Kidney	[B] Mercuric chloride	485 (322 - 1963)	611.5 (± 483.24)	387 (206 - 1815)	585.5 (± 507.62)	909 (415 - 1387)	890.24 (± 443.28)
Liver & Kidney	[E] Microcystin-LR	761 (650 - 1552)	873.5 (± 295.58)	1273.5 (453 - 1783)	1235.6 (± 377.9)	1609 (1409 - 2008)	1663.08 (± 217.98)
Liver & Kidney	[E] Rotenone	732.5 (248 - 1356)	770.1 (± 332.86)	1147.5 (474 - 1984)	1219.88 (± 516.86)	1544.7 (1051 - 1781.7)	1462.23 (± 308.49)
Liver & Kidney	[E] S-(1,2-dichlorovinyl)-cysteine (DCVC)	845.5 (292 - 1533)	929.8 (± 405.38)	1259 (513 - 1827)	1280.7 (± 365.93)	1656 (1116 - 1940)	1592 (± 329.33)
Liver & Kidney	[D] Thioacetamide	750.5 (417 - 1790)	891.6 (± 430.03)	1083.5 (538 - 1762)	1100.2 (± 354.34)	1617 (1235 - 1882)	1576.85 (± 241.71)
Pancreas	[E] 1-Cyano-2-hydroxy-3-butene	1179 (654 - 1773)	1162 (± 370.31)	1511.5 (592 - 1923)	1451.4 (± 369.28)	1352 (1327 - 1504)	1381.6 (± 73.85)
Pancreas	[C] Caerulin	1171.04 (791.2 - 1524.62)	1142.35 (± 246.01)	1477.95 (1168.5 - 1646.12)	1457.5 (± 127.77)	1590.3 (1282.53 - 1765)	1579.44 (± 197.01)
Pancreas	[E] L-arginine	1196 (856 - 2428)	1297.99 (± 410.98)	1719 (1358 - 2112)	1736.68 (± 218.17)	1487 (744 - 1940)	1412.3 (± 347.96)
Pancreas	[B] Streptozotocin	958 (375 - 2146)	908.7 (± 516.95)	902.5 (750 - 2025)	1003.3 (± 366.27)	886 (868 - 1020)	918.2 (± 64.48)
Testicular	[D] 1,3-Dinitrobenzene	1407 (462 - 1798.69)	1342.06 (± 396.66)	1228 (486 - 1706)	1248 (± 416.25)	1257 (1050 - 1795.1)	1360.9 (± 303.53)
Testicular	[C] Cadmium chloride	1339.75 (968.4 - 1444.2)	1303.2 (± 136.31)	1325.08 (730.7 - 1588)	1273.71 (± 262.63)	1440.03 (1348.5 - 1584.17)	1448.72 (± 87.07)
Testicular	[D] Cadmium chloride	491.5 (285 - 689)	479.7 (± 124.7)	483 (256 - 1218)	572.3 (± 289.22)	1218 (776 - 1621)	1222.8 (± 308.49)
Testicular	[D] Carbendazim	1715.85 (1507.2 - 1999)	1750.23 (± 184.75)	1682.35 (1586 - 1992)	1737 (± 157.51)	1636 (902 - 1847)	1510.2 (± 376.01)
Testicular	[D] Di-n-pentyl-phthalate	548 (238 - 1404)	604.8 (± 341)	1364 (994 - 1603)	1321.7 (± 242.23)	1562 (1179 - 1810)	1491.4 (± 263.5)
Testicular	[D] Ethane dimethane sulfonate (EDS)	833.5 (200 - 1810)	937.9 (± 523.39)	1608.05 (891 - 1926)	1436.73 (± 370.41)	1717.9 (1693.9 - 1860.3)	1744.48 (± 69.36)
Testicular	[D] Methoxyacetic acid	1513 (957 - 2166)	1478.8 (± 346.92)	1654.5 (1323 - 1788)	1609.9 (± 158.14)	1768 (1463 - 1826)	1714.6 (± 143.68)
Multiple organ	[B] Adriamycin	666 (268 - 995)	653.6 (± 251.33)	998.5 (303 - 1395)	851.8 (± 418.27)	1295 (926 - 1698)	1335.65 (± 298.43)
Multiple organ	[C] Amphotericin B	1343.17 (1060.3 - 1470.4)	1269.63 (± 155.06)	1134.27 (713.9 - 1551.1)	1134.3 (± 305.81)	1196.8 (806.6 - 1767.21)	1204.71 (± 370.81)
Multiple organ	[C] Azaserine	570.22 (370.4 - 1505.57)	755.38 (± 432.17)	809.7 (642.03 - 1434.05)	918.37 (± 281.28)	1338.9 (891 - 1533)	1275.92 (± 275.57)
Multiple organ	[A] Dexamethasone	1339.5 (673 - 1663)	1312.63 (± 298.03)	1351.4 (898 - 1693)	1367.83 (± 239.13)	1464 (584 - 1875.75)	1401.58 (± 518.68)

Target organ	Toxin	24 h post dose		48 h post dose		168 h post dose	
		Median (min - max)	Mean (± SD)	Median (min - max)	Mean (± SD)	Median (min - max)	Mean (± SD)
Multiple organ	[E] Mitomycin-C	1321.5 (662 - 1744)	1332.2 (± 362.66)	1514 (894 - 2056)	1493.4 (± 389.75)	1292 (1094 - 1594)	1310.4 (± 184.55)
Physiological stressor	[C] 1,1-Dichloroethylene & maleic acid	1369.02 (1047.2 - 1514.1)	1365.21 (± 135.8)	1498.59 (570.02 - 1602.6)	1411.21 (± 303.55)	1692.37 (1558.95 - 1775)	1662.29 (± 91.83)
Physiological stressor	[C] 2,4-Dinitrophenol	1417.7 (1245.19 - 1628.16)	1418.36 (± 119.13)	1380.49 (1226.59 - 1593.3)	1400.98 (± 105.33)	1630.51 (1319.33 - 1802.7)	1566.34 (± 206.51)
Physiological stressor	[B] 4-Pentenoic acid	1987 (1152 - 2367)	1862.9 (± 386.89)	1509 (898 - 1773)	1471.1 (± 283.52)	1845 (1491 - 1905)	1753.2 (± 179.22)
Physiological stressor	[D] Acetazolamide	770 (565 - 1296)	823.3 (± 219.13)	1432.5 (1065 - 1964)	1455.9 (± 282.44)	1501.1 (1228 - 1626)	1476.82 (± 163.86)
Physiological stressor	[C] Acivicin	1497.3 (1240.5 - 1573.24)	1459.13 (± 109.78)	1433.93 (1394.8 - 1582.7)	1465.85 (± 74.36)	1389.63 (1384.53 - 1624.75)	1480.23 (± 127.94)
Physiological stressor	[E] Ammonium chloride	1785.5 (1265 - 2086)	1724.9 (± 215.75)	1970 (1573 - 2244)	1930.3 (± 171.93)	1644.6 (1531.5 - 1781.7)	1658.1 (± 98.5)
Physiological stressor	[D] Carboplatin	815 (212 - 1514)	805 (± 471.99)	1447.5 (585 - 1799)	1319.9 (± 497.14)	1072 (725 - 1219)	994 (± 201.82)
Physiological stressor	[A] Choline and choline/methionine deficiency (chronic)	1058 (786 - 1512)	1112.9 (± 257.37)	979.5 (741 - 1376)	1042.3 (± 196.88)	1292 (879 - 1411)	1223.4 (± 222.21)
Physiological stressor	[B] Food restriction (chronic)	1247 (541 - 1677)	1160.89 (± 396.93)	1018 (711 - 1810.1)	1159.46 (± 367.84)	1220 (1035 - 1669)	1259.4 (± 249.08)
Physiological stressor	[D] Furosemide	1345.5 (959 - 1590)	1335 (± 200.73)	1617 (1304 - 1944)	1646.41 (± 200.26)	1661 (1299 - 1809)	1637.8 (± 205.65)
Physiological stressor	[B] Insulin	1411.5 (974 - 1819)	1423.8 (± 242.67)	1391 (1006 - 1765)	1412.7 (± 243.07)	1477 (1096 - 1850)	1503.8 (± 329.15)
Physiological stressor	[E] Methotrexate	1229 (750 - 1908)	1266.2 (± 377.15)	1429.5 (806 - 2044)	1412.1 (± 413.31)	1752 (1140 - 1944)	1674 (± 332.67)
Physiological stressor	[A] Partial hepatectomy	945 (699 - 1145)	940.7 (± 134.01)	927.5 (483 - 1264)	913.8 (± 235.04)	1247 (1002 - 1636)	1318.2 (± 285.31)
Physiological stressor	[A] Phenobarbital (chronic)	1632.45 (1265 - 1951)	1637.59 (± 205.24)	1567.55 (926 - 1782)	1499.33 (± 275.16)	1515 (1333 - 1805)	1535.2 (± 209.83)
Physiological stressor	[A] Pregnenolone 16 alpha carbonitrile (chronic)	1693.5 (1055 - 1922)	1620.6 (± 278.11)	1638.5 (1065 - 1808)	1542.56 (± 281.15)	1267 (1151 - 1991)	1499 (± 395.09)
Physiological stressor	[A] Probenecid	1274 (705 - 1948)	1275.7 (± 349.24)	1477 (1000 - 1693)	1436.9 (± 209.16)	1497 (1301 - 1847)	1532.2 (± 224.26)
Physiological stressor	[C] Rosiglitazone	1193.01 (713.9 - 1411.79)	1170.72 (± 199.87)	1381.36 (835.9 - 1518.7)	1319.85 (± 190.89)	1382.63 (1213.3 - 1776.35)	1422.44 (± 210.74)
Physiological stressor	[C] Rosiglitazone (chronic)	1331.04 (560.9 - 1664.8)	1287.33 (± 300.72)	1377.76 (1272.95 - 1475.35)	1368.28 (± 70.75)	1602.83 (1536.63 - 1681)	1602.67 (± 59.09)
Physiological stressor	[E] Sodium bicarbonate	1199.5 (1042 - 1942)	1310.7 (± 281.69)	1192.5 (978 - 1547)	1213.3 (± 152.19)	1285.5 (730 - 1554)	1275 (± 232.6)
Physiological stressor	[A] Unilateral nephrectomy	1034.5 (816 - 1412)	1036.2 (± 191.24)	985 (786 - 1193)	992.9 (± 114.22)	1131 (1024 - 1262)	1136 (± 84.48)
Physiological stressor	[B] Water deprivation (chronic)	2344.5 (1217.5 - 2666)	2185.97 (± 434.86)	2275.5 (1084 - 2855)	2233.43 (± 585.65)	1779.9 (1692 - 2448)	1902.63 (± 284.55)

Target organ	Toxin	24 h post dose		48 h post dose		168 h post dose	
		Median (min - max)	Mean (± SD)	Median (min - max)	Mean (± SD)	Median (min - max)	Mean (± SD)
No Effect	E Acetaminophen (chronic)	1401.2 (516.5 - 1637.4)	1241.63 (± 383.75)	1574.5 (660 - 1927)	1473.8 (± 367.49)	1481 (707 - 2298)	1411.4 (± 563.79)
No Effect	C Buthionine sulphoxime	1338.89 (1107.5 - 1523.9)	1325.84 (± 118.12)	1423.15 (968 - 1652)	1354.81 (± 210.82)	1424.93 (1345.23 - 1804.7)	1475.23 (± 188.1)
No Effect	C Ferrous sulphate	1370.28 (1146.1 - 1680.97)	1385.92 (± 165.17)	1479.03 (1077.1 - 1580.3)	1423.52 (± 169.33)	1638.97 (1590.3 - 1675.95)	1636.95 (± 35.98)
No Effect	B Ifosfamide	815 (168 - 1384)	711.64 (± 406.94)	1287 (564 - 1922)	1318.6 (± 552.32)	1650 (1326 - 1982)	1614.8 (± 250.07)
No Effect	B Lithocholic acid	1473 (1204 - 1880)	1528 (± 294.82)	1480 (1236 - 1722)	1480.8 (± 204.37)	1702 (1316 - 1902)	1663.2 (± 221.92)
No Effect	E Paraquat	1305.5 (477 - 2018)	1361.9 (± 425.45)	1515 (743 - 1939)	1423.6 (± 422.81)	1510 (1255 - 1984)	1560.2 (± 296.37)
No Effect	D Potassium dichromate	1228 (371 - 1773)	1210.7 (± 401.4)	1310 (579 - 1754)	1340.4 (± 329.35)	1460 (728 - 1854)	1396.6 (± 415.51)
No Effect	C Trichlorethylene	1438 (1167.4 - 1685.5)	1436.77 (± 142.48)	1463.45 (1189.43 - 1606.7)	1429.79 (± 121.56)	1583.95 (1521 - 1757)	1604 (± 93.61)

A-F: Indicates Pharmaceutical Company & sample origin

Supplementary Table S35. Summary statistics for urine glucose (mmol/L) at 24 hrs, 48 hrs and 168 hrs post high dose.

Target organ	Toxin	24 h post dose		48 h post dose		168 h post dose	
		Median (min - max)	Mean (± SD)	Median (min - max)	Mean (± SD)	Median (min - max)	Mean (± SD)
Liver	E 1,1-Dichloroethylene	1.5 (0.67 - 1.78)	1.29 (± 0.44)	1.75 (0.94 - 2.44)	1.75 (± 0.44)	1.61 (1.28 - 2.11)	1.67 (± 0.34)
Liver	E 1,2,3,4,5,6-hexachlorocyclohexane	1.61 (1.17 - 2.89)	1.79 (± 0.53)	1.61 (0.67 - 3.16)	1.73 (± 0.75)	1.26 (1.15 - 1.6)	1.35 (± 0.22)
Liver	B 1-Fluoropentane	6.22 (1.91 - 26.63)	9.07 (± 7.76)	2.35 (1.21 - 39.03)	8.02 (± 11.9)	1.29 (0.89 - 50.39)	11.03 (± 22)
Liver	B 2,4,6-Trihydroxyacetophenone (THA)	1.58 (0.98 - 10.86)	2.63 (± 2.96)	1.32 (0.95 - 1.64)	1.33 (± 0.22)	1.04 (0.88 - 1.29)	1.05 (± 0.15)
Liver	B 4-Amino-2,6-dichlorophenol (ADCP)	0.94 (0.49 - 1.93)	1.02 (± 0.45)	1.53 (0.89 - 2.92)	1.57 (± 0.63)	1.21 (0.93 - 1.78)	1.27 (± 0.37)
Liver	C Aflatoxin	0.65 (0.26 - 1.22)	0.67 (± 0.31)	0.57 (0.17 - 0.86)	0.59 (± 0.21)	1.55 (1.35 - 5.74)	2.42 (± 1.88)
Liver	C Allyl alcohol	17.44 (2.06 - 44.23)	19.23 (± 14.98)	2.57 (1.08 - 5.5)	2.62 (± 1.36)	2.03 (1.65 - 3.47)	2.5 (± 0.89)
Liver	C Allyl formate	1.92 (1.28 - 62.25)	9.11 (± 18.98)	1.87 (1.12 - 3.07)	1.95 (± 0.65)	2.77 (1.64 - 5.5)	3.12 (± 1.54)
Liver	B Azathioprine	55.79 (8.66 - 77.04)	47.65 (± 24.15)	5.94 (1.92 - 21.66)	10.13 (± 7.93)	1.42 (0.92 - 1.6)	1.36 (± 0.26)

Target organ	Toxin	24 h post dose		48 h post dose		168 h post dose	
		Median (min - max)	Mean (± SD)	Median (min - max)	Mean (± SD)	Median (min - max)	Mean (± SD)
Liver	[B] Bromobenzene	4.71 (1.48 - 7.28)	4.05 (± 1.92)	4.32 (1.15 - 11.96)	4.23 (± 3.1)	1.3 (0.88 - 1.57)	1.3 (± 0.27)
Liver	[C] Butylated hydroxytoluene	1.76 (1.02 - 26.53)	4.21 (± 7.85)	1.23 (0.88 - 34.39)	11.92 (± 14.53)	1.97 (1.5 - 2.37)	1.92 (± 0.37)
Liver	[D] Carbon tetrachloride	1.75 (0.6 - 3.7)	2.07 (± 0.9)	1.4 (0.5 - 2.3)	1.42 (± 0.56)	2 (1.1 - 2.7)	1.84 (± 0.65)
Liver	[C] Chlorpromazine	1.7 (1.08 - 14)	3.82 (± 4.51)	1.64 (1.04 - 8.32)	2.38 (± 2.13)	1.89 (1.31 - 2.72)	1.94 (± 0.54)
Liver	[B] Clofibrate	0.82 (0.27 - 1.75)	0.84 (± 0.56)	0.45 (0.14 - 0.87)	0.5 (± 0.23)	1.02 (0.7 - 1.35)	1 (± 0.25)
Liver	[B] Cyproterone acetate	0.42 (0.13 - 0.75)	0.44 (± 0.21)	0.26 (0.11 - 1.17)	0.4 (± 0.31)	0.95 (0.65 - 1.09)	0.87 (± 0.19)
Liver	[A] D-galactosamine	0.92 (0.22 - 1.28)	0.92 (± 0.29)	0.89 (0.61 - 1.22)	0.88 (± 0.22)	1.05 (0.83 - 1.33)	1.07 (± 0.18)
Liver	[B] Diethylhexylphthalate (DEHP)	10.47 (2.51 - 25.39)	10.86 (± 7.46)	16.16 (1.68 - 61.65)	21.86 (± 20.83)	1.41 (0.68 - 41.27)	10.45 (± 17.45)
Liver	[C] Dimethylformamide (DMF)	1.46 (1.17 - 5.5)	1.97 (± 1.32)	1.72 (1.02 - 37.3)	5.22 (± 11.28)	1.84 (1.22 - 2.17)	1.79 (± 0.38)
Liver	[C] Dimethylnitrosamine (DMN)	0.87 (0.34 - 1.62)	0.9 (± 0.43)	1.21 (0.45 - 2.02)	1.25 (± 0.56)	1.32 (1.03 - 5.19)	2.08 (± 1.76)
Liver	[A] Gadolinium chloride	1.44 (0.67 - 2.16)	1.44 (± 0.46)	1.28 (0.78 - 1.94)	1.27 (± 0.36)	1.5 (0.56 - 1.94)	1.4 (± 0.52)
Liver	[A, B, C, D, F] Hydrazine	1.1 (0.01 - 74.39)	7.01 (± 12.87)	1.59 (0 - 43.99)	6.06 (± 9.83)	1.1 (0 - 43)	8.1 (± 13.86)
Liver	[E] Hydrazine	18 (8 - 27)	18.3 (± 5.56)	26.5 (16 - 35)	26.6 (± 5.97)	17 (13 - 64)	25.8 (± 21.58)
Liver	[E] Indomethacin	2.19 (1.61 - 4.16)	2.49 (± 0.87)	1 (0.44 - 1.89)	1.14 (± 0.44)	1.17 (0.44 - 2.01)	1.2 (± 0.56)
Liver	[E] Ketoconazole	1.83 (1.11 - 2.22)	1.72 (± 0.36)	2 (1.05 - 2.28)	1.9 (± 0.4)	1.39 (1 - 2.16)	1.49 (± 0.43)
Liver	[C] Lead acetate	1.7 (1.03 - 2.58)	1.75 (± 0.45)	1.75 (1.01 - 4.58)	1.95 (± 0.99)	1.07 (0.93 - 1.68)	1.28 (± 0.35)
Liver	[A] Lipopolysaccharide (LPS)	0.78 (0.33 - 2.21)	0.91 (± 0.51)	0.86 (0.44 - 2.18)	0.91 (± 0.49)	1 (0.83 - 1.11)	0.97 (± 0.11)
Liver	[B] Methapyrilene	1.32 (0.65 - 1.93)	1.32 (± 0.33)	1.09 (0.42 - 1.4)	1.04 (± 0.31)	1.48 (1.34 - 1.67)	1.49 (± 0.12)
Liver	[E] Methylene dianiline	1.61 (1 - 2.33)	1.67 (± 0.39)	1.89 (1.11 - 2.16)	1.79 (± 0.38)	1.89 (1 - 2.16)	1.81 (± 0.47)
Liver	[C] Monocrotaline	1.52 (0.54 - 12.27)	2.54 (± 3.48)	1.89 (0.93 - 7.74)	2.65 (± 2.2)	2.37 (1.73 - 5.5)	2.87 (± 1.55)
Liver	[C] N-methylformamide (NMF)	2.02 (1.03 - 39.44)	9.25 (± 14.68)	14.16 (0.72 - 43.17)	15.19 (± 13.87)	2.46 (2.3 - 3)	2.54 (± 0.27)

Target organ	Toxin	24 h post dose		48 h post dose		168 h post dose	
		Median (min - max)	Mean (± SD)	Median (min - max)	Mean (± SD)	Median (min - max)	Mean (± SD)
Liver	[D] Phalloidin (chronic)	1.16 (0.69 - 1.6)	1.14 (± 0.3)	1.06 (0.45 - 1.35)	1 (± 0.28)	1.6 (1.44 - 1.85)	1.61 (± 0.18)
Liver	[E] Phenyl diisothiocyanate	1.5 (1 - 103.03)	15.17 (± 31.8)	2.22 (0.67 - 9.94)	3.14 (± 2.83)	1.72 (1.22 - 2.01)	1.69 (± 0.32)
Liver	[E] Phenyl isothiocyanate	1.44 (0.5 - 2.16)	1.4 (± 0.59)	0.92 (0.61 - 1.5)	0.96 (± 0.33)	0.83 (0.39 - 1.55)	0.88 (± 0.43)
Liver	[B] Retinyl palmitate	5.64 (1.21 - 14.27)	6.49 (± 4.45)	4.57 (1.4 - 67.04)	13 (± 20.14)	1.44 (1 - 2.64)	1.59 (± 0.62)
Liver	[B] Sodium Valproate	22.44 (1.5 - 156.48)	34.47 (± 46.32)	7.49 (0.97 - 80.43)	23.54 (± 27.56)	1.24 (1.01 - 1.77)	1.35 (± 0.31)
Liver	[C] a-Naphthylisothiocyanate (ANIT)	5.03 (1.45 - 64.19)	17.07 (± 23.18)	7.17 (1.08 - 16.87)	6.15 (± 4.73)	5.5 (1.9 - 5.5)	4.69 (± 1.57)
Kidney	[D] 2-Bromophenol	1.5 (1 - 2.1)	1.45 (± 0.34)	1.95 (1.6 - 2.5)	2 (± 0.3)	1.5 (0.9 - 1.8)	1.38 (± 0.37)
Kidney	[E] 3,5-Dichloroaniline hydrochloride	1.14 (0.67 - 1.44)	1.03 (± 0.24)	1.64 (0.67 - 2.5)	1.67 (± 0.55)	0.94 (0.78 - 1.67)	1.11 (± 0.36)
Kidney	[E] Atractyloside	49.79 (1.33 - 93.09)	53.88 (± 26.09)	18.11 (1.22 - 57.9)	20.2 (± 15.34)	0.89 (0.72 - 2.28)	1.18 (± 0.64)
Kidney	[D] Bromoethylamine hydrobromide	3.3 (2.4 - 4.1)	3.24 (± 0.45)	1.2 (0.8 - 2.3)	1.34 (± 0.49)	0.8 (0.5 - 0.9)	0.74 (± 0.15)
Kidney	[D] Cephaloridine	0.9 (0.5 - 80.2)	8.86 (± 25.07)	1.85 (0.8 - 228)	25.68 (± 71.22)	1.6 (0.9 - 1.9)	1.5 (± 0.41)
Kidney	[B] Chlorethanamine	1.1 (0.39 - 2.91)	1.2 (± 0.76)	1.28 (0.38 - 2.49)	1.37 (± 0.78)	0.72 (0.64 - 0.74)	0.7 (± 0.04)
Kidney	[A] Cisplatin	1.1 (0.6 - 2.1)	1.29 (± 0.59)	4.15 (2.1 - 10.9)	5.08 (± 2.92)	2.9 (1.4 - 4.4)	2.72 (± 1.18)
Kidney	[A] D-limonene (chronic)	2.2 (1.4 - 3.5)	2.23 (± 0.68)	1.55 (1 - 2.3)	1.63 (± 0.39)	1.8 (1.4 - 2.2)	1.84 (± 0.32)
Kidney	[E] Dichlorophenyl succinimide	0.86 (0.52 - 1.14)	0.84 (± 0.23)	0.5 (0.33 - 1.08)	0.58 (± 0.22)	0.84 (0.44 - 0.98)	0.78 (± 0.21)
Kidney	[D] Ethylene glycol	1.4 (0.8 - 2.9)	1.58 (± 0.56)	1.25 (0.9 - 1.8)	1.27 (± 0.29)	2.6 (2.2 - 3.6)	2.8 (± 0.53)
Kidney	[A] Folic acid	2.2 (0.9 - 3.1)	1.97 (± 0.73)	1.2 (0.7 - 2.77)	1.33 (± 0.66)	1.9 (1.1 - 2.15)	1.71 (± 0.41)
Kidney	[A] Gentamicin	4.25 (2.8 - 7.1)	4.51 (± 1.26)	3.2 (2.4 - 6.5)	3.53 (± 1.16)	2.2 (1 - 3.3)	2.08 (± 0.91)
Kidney	[B] Maleic acid	18.12 (1.38 - 287.49)	60.33 (± 92.08)	3.08 (1.04 - 182.21)	33.87 (± 59.96)	1.18 (1.03 - 2.01)	1.36 (± 0.39)
Kidney	[A] N-phenylanthranilic acid (chronic)	0.94 (0.5 - 4.27)	1.43 (± 1.24)	0.67 (0.44 - 1.44)	0.78 (± 0.32)	0.83 (0.61 - 2.15)	1.13 (± 0.65)
Kidney	[D] Para-aminophenol	86.8 (31.5 - 178)	92.67 (± 40.64)	51.8 (8.5 - 165)	55.63 (± 43.69)	1 (0.7 - 1.3)	0.98 (± 0.22)

Target organ	Toxin	24 h post dose		48 h post dose		168 h post dose	
		Median (min - max)	Mean (± SD)	Median (min - max)	Mean (± SD)	Median (min - max)	Mean (± SD)
Kidney	[A] Puromycin	1.15 (0.4 - 1.8)	1.07 (± 0.59)	1.4 (0.7 - 2.8)	1.5 (± 0.68)	1.4 (1.1 - 2)	1.5 (± 0.43)
Kidney	[B] Vancomycin hydrochloride	1.37 (0.75 - 1.92)	1.35 (± 0.3)	0.75 (0.51 - 0.95)	0.73 (± 0.17)	1.35 (1.2 - 1.55)	1.34 (± 0.14)
Liver & Kidney	[E] Acetaminophen	4.27 (1.05 - 88.37)	19.11 (± 28.11)	1.94 (1.11 - 3.89)	2.24 (± 0.83)	1.39 (0.94 - 1.55)	1.29 (± 0.24)
Liver & Kidney	[B] Aurothiomalate	88.18 (0.52 - 121.11)	81.25 (± 36.78)	43.68 (0.41 - 123.04)	52.45 (± 42.67)	1.21 (0.74 - 2.45)	1.35 (± 0.68)
Liver & Kidney	[C] Chloroform	2.68 (1.09 - 26.25)	6.51 (± 8.01)	1.46 (0.95 - 78.01)	10.2 (± 24)	1.24 (1.11 - 1.73)	1.32 (± 0.24)
Liver & Kidney	[D] Cyclosporin	2 (0.6 - 112)	14.19 (± 34.66)	3.85 (1 - 175)	29.16 (± 54.22)	1.2 (1.1 - 1.8)	1.38 (± 0.34)
Liver & Kidney	[D] Dichlorobenzene	2.7 (1.2 - 4.7)	2.8 (± 1.07)	3.4 (1.3 - 5.3)	3.12 (± 1.35)	2.5 (1.8 - 3.8)	2.56 (± 0.84)
Liver & Kidney	[C] Ethionine	2.17 (0.93 - 23.93)	4.65 (± 6.91)	1.76 (1.02 - 37.36)	8.8 (± 12.95)	2.21 (1.65 - 3.66)	2.49 (± 0.78)
Liver & Kidney	[B] Hexachlorobutadiene (HCBD)	98.26 (0 - 199.8)	97.35 (± 52.15)	67.96 (8.27 - 125.54)	71.07 (± 35.44)	0.91 (0.57 - 7.31)	2.1 (± 2.92)
Liver & Kidney	[B] Mercuric chloride	52.65 (1.63 - 70.52)	47.61 (± 21.01)	19.76 (1.49 - 51.74)	22.33 (± 14.52)	1.07 (0 - 32.55)	7.2 (± 14.19)
Liver & Kidney	[E] Microcystin-LR	13.82 (1.22 - 22.54)	12.27 (± 7.87)	6.05 (1.33 - 26.53)	10.81 (± 9.74)	1.78 (0.72 - 3.5)	2.11 (± 1.08)
Liver & Kidney	[E] Rotenone	1.22 (0.39 - 14.27)	3.23 (± 4.23)	1.33 (0 - 1.94)	1.22 (± 0.7)	1.29 (0.56 - 2.01)	1.28 (± 0.57)
Liver & Kidney	[E] S-(1,2-dichlorovinyl)-cysteine (DCVC)	6.52 (1.11 - 97.92)	14.98 (± 29.3)	4.05 (1.89 - 97.86)	15.99 (± 30.22)	1.44 (1.22 - 1.61)	1.42 (± 0.15)
Liver & Kidney	[D] Thioacetamide	0.8 (0.3 - 1.6)	0.82 (± 0.39)	1.1 (0.5 - 6.2)	1.61 (± 1.64)	1.5 (1.1 - 2.8)	1.78 (± 0.68)
Pancreas	[E] 1-Cyano-2-hydroxy-3-butene	1.75 (0.83 - 19.21)	4.88 (± 6.63)	1.72 (0.67 - 2.05)	1.64 (± 0.38)	2.05 (1 - 2.22)	1.72 (± 0.61)
Pancreas	[C] Caerulin	1.12 (0.55 - 1.74)	1.16 (± 0.36)	1.41 (0.9 - 1.91)	1.51 (± 0.34)	1.83 (0.84 - 2.6)	1.85 (± 0.65)
Pancreas	[E] L-arginine	3.91 (1.33 - 9.21)	4.65 (± 2.69)	2.5 (1.22 - 4.27)	2.8 (± 0.91)	1.19 (0.67 - 2.33)	1.28 (± 0.52)
Pancreas	[B] Streptozotocin	60.08 (0.56 - 433.23)	146.81 (± 179.66)	859.97 (3.38 - 1117.21)	801 (± 331.7)	567.76 (396.27 - 669.88)	555.67 (± 101.17)
Testicular	[D] 1,3-Dinitrobenzene	1.75 (0.7 - 3.8)	2 (± 0.99)	1.35 (0.6 - 3.4)	1.5 (± 0.76)	1.4 (1 - 3.8)	2.04 (± 1.18)
Testicular	[C] Cadmium chloride	2.09 (0.89 - 5.72)	2.52 (± 1.54)	1.27 (0.48 - 2.13)	1.21 (± 0.56)	1.39 (1.33 - 1.5)	1.41 (± 0.08)
Testicular	[D] Cadmium chloride	0.74 (0.42 - 1.11)	0.73 (± 0.22)	0.72 (0.41 - 1.51)	0.83 (± 0.35)	0.73 (0.4 - 1.03)	0.72 (± 0.24)

Target organ	Toxin	24 h post dose		48 h post dose		168 h post dose	
		Median (min - max)	Mean (± SD)	Median (min - max)	Mean (± SD)	Median (min - max)	Mean (± SD)
Testicular	[D] Carbendazim	2.35 (1.6 - 3.8)	2.68 (± 0.84)	2.05 (1.4 - 2.9)	2.05 (± 0.42)	1.4 (1 - 1.5)	1.28 (± 0.26)
Testicular	[D] Di-n-pentyl-phthalate	2.28 (1.51 - 2.9)	2.19 (± 0.51)	1.39 (1.15 - 2.1)	1.53 (± 0.39)	0.89 (0.82 - 1.31)	1.03 (± 0.23)
Testicular	[D] Ethane dimethane sulfonate (EDS)	0.85 (0.4 - 2.4)	1.07 (± 0.65)	2.1 (1.2 - 4.6)	2.29 (± 0.96)	1.5 (1.3 - 1.6)	1.48 (± 0.11)
Testicular	[D] Methoxyacetic acid	1.52 (0.74 - 2.04)	1.48 (± 0.42)	1.38 (1 - 2.01)	1.4 (± 0.32)	1.2 (1.15 - 1.37)	1.25 (± 0.1)
Multiple organ	[B] Adriamycin	0.47 (0.24 - 0.85)	0.53 (± 0.21)	0.86 (0.26 - 1.35)	0.76 (± 0.37)	1.14 (0.73 - 21.05)	5.07 (± 8.94)
Multiple organ	[C] Amphotericin B	1.27 (0.9 - 1.81)	1.31 (± 0.23)	1.22 (0.83 - 2.79)	1.43 (± 0.66)	1.16 (0.71 - 2.35)	1.37 (± 0.66)
Multiple organ	[C] Azaserine	0.72 (0.19 - 1.94)	0.79 (± 0.61)	15.59 (1.61 - 30.27)	15.54 (± 9.1)	40.75 (2.11 - 88.63)	43.35 (± 41.79)
Multiple organ	[A] Dexamethasone	123.75 (2.3 - 377)	128 (± 125.2)	487 (290 - 770)	525.1 (± 177.78)	2.8 (1.2 - 756)	187.6 (± 326.56)
Multiple organ	[E] Mitomycin-C	1.5 (0.78 - 2.44)	1.5 (± 0.45)	1.72 (1.33 - 2.72)	1.85 (± 0.48)	0.83 (0.61 - 1)	0.81 (± 0.17)
Physiological stressor	[C] 1,1-Dichloroethylene & maleic acid	1.96 (1.39 - 2.23)	1.84 (± 0.31)	1.67 (0.33 - 2.49)	1.54 (± 0.59)	1.76 (1.47 - 2.09)	1.77 (± 0.22)
Physiological stressor	[C] 2,4-Dinitrophenol	1.48 (1.15 - 1.96)	1.55 (± 0.26)	1.71 (1.35 - 1.94)	1.66 (± 0.23)	1.33 (1.01 - 1.49)	1.27 (± 0.19)
Physiological stressor	[B] 4-Pentenoic acid	1.47 (0.84 - 39.38)	6.04 (± 11.94)	1.28 (0.84 - 39.17)	7.95 (± 14.42)	1.42 (1.24 - 1.47)	1.37 (± 0.1)
Physiological stressor	[D] Acetazolamide	0.7 (0.5 - 1.3)	0.72 (± 0.23)	2 (1 - 2.7)	1.97 (± 0.58)	2.3 (1.7 - 3.9)	2.56 (± 0.85)
Physiological stressor	[C] Acivicin	1.63 (1.43 - 4.18)	2 (± 0.89)	1.54 (1.28 - 1.94)	1.56 (± 0.21)	1.22 (1.1 - 1.57)	1.32 (± 0.2)
Physiological stressor	[E] Ammonium chloride	1.42 (1.11 - 1.78)	1.43 (± 0.21)	1.67 (1.17 - 2.28)	1.67 (± 0.32)	1.93 (1.58 - 7.79)	3 (± 2.68)
Physiological stressor	[D] Carboplatin	0.65 (0.2 - 1.6)	0.78 (± 0.51)	1.35 (0.6 - 1.9)	1.26 (± 0.46)	1 (0.8 - 1.2)	0.98 (± 0.18)
Physiological stressor	[A] Choline and choline/methionine deficiency (chronic)	2.25 (1.5 - 3.39)	2.35 (± 0.68)	1.97 (1.5 - 3.44)	2.14 (± 0.58)	2.22 (1.55 - 2.89)	2.31 (± 0.56)
Physiological stressor	[B] Food restriction (chronic)	0.62 (0.46 - 1.08)	0.71 (± 0.24)	1.02 (0.31 - 1.88)	1 (± 0.58)	0.91 (0.69 - 1.14)	0.9 (± 0.19)
Physiological stressor	[D] Furosemide	1.95 (1.1 - 2.6)	1.87 (± 0.57)	2.3 (1.3 - 3.2)	2.18 (± 0.63)	2 (1.3 - 2.4)	1.92 (± 0.43)
Physiological stressor	[B] Insulin	0.68 (0.46 - 1.08)	0.71 (± 0.21)	0.96 (0.73 - 1.49)	0.99 (± 0.23)	1.33 (0.79 - 1.72)	1.24 (± 0.37)
Physiological stressor	[E] Methotrexate	1.33 (0.83 - 2.28)	1.33 (± 0.44)	1.64 (0.94 - 2.05)	1.6 (± 0.39)	1.67 (1.05 - 3.05)	1.89 (± 0.76)

Target organ	Toxin	24 h post dose		48 h post dose		168 h post dose	
		Median (min - max)	Mean (± SD)	Median (min - max)	Mean (± SD)	Median (min - max)	Mean (± SD)
Physiological stressor	[A] Partial hepatectomy	0.09 (0.04 - 0.1)	0.08 (± 0.02)	0.08 (0.03 - 0.12)	0.08 (± 0.03)	0.08 (0.06 - 0.12)	0.09 (± 0.03)
Physiological stressor	[A] Phenobarbital (chronic)	2.9 (2 - 4.2)	3.02 (± 0.73)	2.55 (1 - 4.1)	2.42 (± 0.93)	1.9 (1.3 - 3.4)	2.08 (± 0.79)
Physiological stressor	[A] Pregnenolone 16 alpha carbonitrile (chronic)	1.7 (0.8 - 3.2)	1.82 (± 0.61)	1.7 (0.9 - 2.8)	1.79 (± 0.54)	1.2 (1 - 1.7)	1.28 (± 0.28)
Physiological stressor	[A] Probenecid	1.22 (0.89 - 22.3)	3.41 (± 6.65)	1.36 (0.78 - 1.61)	1.31 (± 0.24)	1.33 (1 - 1.5)	1.27 (± 0.21)
Physiological stressor	[C] Rosiglitazone	1.12 (0.54 - 2.29)	1.12 (± 0.49)	1.3 (0.78 - 1.83)	1.32 (± 0.28)	1.11 (0.87 - 1.65)	1.18 (± 0.29)
Physiological stressor	[C] Rosiglitazone (chronic)	1.4 (0.48 - 2.02)	1.4 (± 0.44)	1.62 (1.07 - 2.83)	1.67 (± 0.49)	1.62 (1.32 - 2.11)	1.61 (± 0.32)
Physiological stressor	[E] Sodium bicarbonate	1.08 (0.78 - 2.39)	1.24 (± 0.48)	1.39 (0.94 - 1.72)	1.33 (± 0.32)	1.05 (0.72 - 1.83)	1.1 (± 0.33)
Physiological stressor	[A] Unilateral nephrectomy	0.08 (0.05 - 0.12)	0.08 (± 0.02)	0.3 (0.12 - 0.52)	0.32 (± 0.12)	0.08 (0.08 - 0.09)	0.08 (± 0.01)
Physiological stressor	[B] Water deprivation (chronic)	2.08 (1.23 - 16.77)	3.23 (± 4.28)	2.82 (2 - 4.92)	3 (± 0.78)	1.2 (1.18 - 1.46)	1.26 (± 0.12)
No Effect	[E] Acetaminophen (chronic)	1.22 (0.44 - 1.61)	1.19 (± 0.4)	1.39 (0.67 - 1.83)	1.35 (± 0.36)	1.28 (0.56 - 2.39)	1.36 (± 0.61)
No Effect	[C] Buthionine sulphoxime	1.65 (0.91 - 3.13)	1.7 (± 0.63)	1.42 (0.99 - 1.91)	1.4 (± 0.28)	1.36 (1.15 - 1.72)	1.39 (± 0.21)
No Effect	[C] Ferrous sulphate	2.75 (1.84 - 10.95)	4.46 (± 3.24)	1.43 (1.03 - 5.5)	1.88 (± 1.3)	1.94 (1.49 - 2.53)	2 (± 0.48)
No Effect	[B] Ifosfamide	0.85 (0 - 2.29)	0.97 (± 0.75)	1.49 (0.75 - 2.43)	1.49 (± 0.58)	1.13 (0.82 - 1.24)	1.09 (± 0.16)
No Effect	[B] Lithocholic acid	1.41 (0.8 - 38.71)	6.71 (± 12.3)	1.31 (0.95 - 5.14)	1.68 (± 1.27)	1.37 (0.95 - 41.14)	9.24 (± 17.83)
No Effect	[E] Paraquat	1.39 (0.78 - 2.06)	1.49 (± 0.39)	1.25 (0.67 - 2.17)	1.31 (± 0.42)	1.22 (0.83 - 1.39)	1.14 (± 0.22)
No Effect	[D] Potassium dichromate	1.55 (0.4 - 1.9)	1.42 (± 0.47)	1.5 (0.8 - 2.1)	1.5 (± 0.44)	1.5 (0.8 - 2.1)	1.44 (± 0.51)
No Effect	[C] Trichlorethylene	1.67 (1.31 - 5.5)	2.2 (± 1.36)	1.87 (1.04 - 5.5)	2.17 (± 1.23)	1.85 (1.38 - 2.93)	1.95 (± 0.58)

A-F: Indicates Pharmaceutical Company & sample origin

Supplementary Table S36. Summary statistics for urine protein (g/L) at 24 hrs, 48 hrs and 168 hrs post high dose.

Target organ	Toxin	24 h post dose		48 h post dose		168 h post dose	
		Median (min - max)	Mean (± SD)	Median (min - max)	Mean (± SD)	Median (min - max)	Mean (± SD)
Liver	[E] 1,1-Dichloroethylene	0.38 (0.24 - 0.59)	0.4 (± 0.14)	0.39 (0.29 - 0.64)	0.41 (± 0.11)	0.66 (0.42 - 0.85)	0.63 (± 0.16)
Liver	[E] 1,2,3,4,5,6-hexachlorocyclohexane	0.66 (0.48 - 1.03)	0.69 (± 0.16)	0.88 (0.59 - 1.13)	0.88 (± 0.17)	0.63 (0.56 - 0.75)	0.64 (± 0.08)
Liver	[B] 1-Fluoropentane	1.62 (1.14 - 2.06)	1.58 (± 0.33)	1.36 (0.95 - 1.98)	1.42 (± 0.35)	1.17 (0.66 - 1.82)	1.22 (± 0.42)
Liver	[B] 2,4,6-Trihydroxyacetophenone (THA)	0.6 (0.37 - 2.27)	0.93 (± 0.63)	0.79 (0.43 - 2.07)	0.98 (± 0.56)	0.61 (0.53 - 0.89)	0.68 (± 0.16)
Liver	[B] 4-Amino-2,6-dichlorophenol (ADCP)	0.89 (0.23 - 2.52)	1.01 (± 0.74)	1.04 (0.39 - 2.3)	1.14 (± 0.63)	0.98 (0.21 - 1.74)	0.9 (± 0.57)
Liver	[C] Aflatoxin	0.33 (0.23 - 1)	0.41 (± 0.22)	0.3 (0.19 - 0.38)	0.3 (± 0.06)	1 (0.3 - 1)	0.72 (± 0.38)
Liver	[C] Allyl alcohol	1.23 (0.3 - 1.83)	1.06 (± 0.52)	0.3 (0.3 - 0.86)	0.44 (± 0.23)	1 (1 - 1)	1 (± 0)
Liver	[C] Allyl formate	1 (0.3 - 3)	1.32 (± 1.2)	1 (0.3 - 1)	0.92 (± 0.22)	1 (1 - 1.07)	1.01 (± 0.03)
Liver	[B] Azathioprine	1.38 (0.94 - 1.99)	1.45 (± 0.31)	1.33 (1.16 - 1.82)	1.4 (± 0.21)	1.26 (0.48 - 1.5)	1.14 (± 0.39)
Liver	[B] Bromobenzene	1.5 (0.9 - 2.38)	1.61 (± 0.44)	1.32 (1.13 - 2.04)	1.45 (± 0.34)	1.05 (0.62 - 1.44)	1.05 (± 0.38)
Liver	[C] Butylated hydroxytoluene	0.6 (0.3 - 1.99)	0.66 (± 0.5)	0.56 (0.3 - 2.86)	0.94 (± 0.78)	1 (0.3 - 1)	0.72 (± 0.38)
Liver	[D] Carbon tetrachloride	0.3 (0.22 - 0.52)	0.32 (± 0.1)	0.23 (0.2 - 0.52)	0.28 (± 0.1)	0.71 (0.67 - 0.83)	0.72 (± 0.06)
Liver	[C] Chlorpromazine	0.3 (0.3 - 1)	0.48 (± 0.29)	1 (0.3 - 1)	0.79 (± 0.34)	1 (0.3 - 1)	0.72 (± 0.38)
Liver	[B] Clofibrate	0.57 (0.06 - 1.72)	0.77 (± 0.65)	0.23 (0.11 - 1.12)	0.44 (± 0.39)	0.66 (0.4 - 0.97)	0.68 (± 0.22)
Liver	[B] Cyproterone acetate	0.11 (0.02 - 0.47)	0.17 (± 0.14)	0.08 (0.02 - 0.38)	0.13 (± 0.12)	0.52 (0.41 - 0.74)	0.54 (± 0.12)
Liver	[A] D-galactosamine	0.3 (0.12 - 0.67)	0.33 (± 0.14)	0.31 (0.18 - 1.03)	0.41 (± 0.27)	0.42 (0.3 - 0.66)	0.44 (± 0.14)
Liver	[B] Diethylhexylphthalate (DEHP)	1.09 (0.69 - 2)	1.25 (± 0.47)	1.54 (0.55 - 2.1)	1.43 (± 0.46)	1.25 (0.5 - 1.29)	1.09 (± 0.34)
Liver	[C] Dimethylformamide (DMF)	0.3 (0.3 - 1)	0.51 (± 0.34)	0.43 (0.3 - 1.93)	0.66 (± 0.53)	1 (0.3 - 1)	0.72 (± 0.38)
Liver	[C] Dimethylnitrosamine (DMN)	0.31 (0.24 - 0.58)	0.35 (± 0.1)	0.45 (0.3 - 0.94)	0.51 (± 0.23)	0.3 (0 - 0.37)	0.25 (± 0.15)
Liver	[A] Gadolinium chloride	1.19 (0.4 - 1.85)	1.14 (± 0.5)	0.9 (0.32 - 1.48)	0.9 (± 0.36)	0.93 (0.35 - 1.43)	0.91 (± 0.38)

Target organ	Toxin	24 h post dose		48 h post dose		168 h post dose	
		Median (min - max)	Mean (± SD)	Median (min - max)	Mean (± SD)	Median (min - max)	Mean (± SD)
Liver	[A, B, C, D, F] Hydrazine	0.2 (0 - 1.89)	0.38 (± 0.42)	0.25 (0.06 - 2.3)	0.44 (± 0.47)	0.12 (0 - 2.4)	0.61 (± 0.81)
Liver	[E] Hydrazine	0.03 (0.02 - 0.04)	0.03 (± 0.01)	0.05 (0.04 - 0.06)	0.05 (± 0.01)	0.03 (0.02 - 0.1)	0.04 (± 0.03)
Liver	[E] Indomethacin	0.58 (0.42 - 0.8)	0.62 (± 0.16)	0.6 (0.47 - 0.86)	0.64 (± 0.14)	0.27 (0.22 - 0.76)	0.43 (± 0.26)
Liver	[E] Ketoconazole	0.46 (0.35 - 0.64)	0.48 (± 0.11)	0.5 (0.33 - 0.69)	0.51 (± 0.12)	0.74 (0.6 - 0.81)	0.7 (± 0.09)
Liver	[C] Lead acetate	0.3 (0.3 - 1)	0.4 (± 0.22)	0.47 (0.3 - 1)	0.59 (± 0.32)	0.35 (0.3 - 1)	0.49 (± 0.3)
Liver	[A] Lipopolysaccharide (LPS)	0.61 (0.22 - 0.76)	0.55 (± 0.19)	0.62 (0.14 - 1.12)	0.61 (± 0.3)	0.98 (0.52 - 1.49)	0.95 (± 0.43)
Liver	[B] Methapyrilene	1.08 (0.47 - 1.79)	1.08 (± 0.33)	0.66 (0.41 - 1.04)	0.7 (± 0.21)	1.43 (1.24 - 1.47)	1.39 (± 0.1)
Liver	[E] Methylene dianiline	0.59 (0.42 - 0.79)	0.6 (± 0.12)	0.62 (0.36 - 0.79)	0.58 (± 0.16)	0.85 (0.63 - 1.03)	0.82 (± 0.17)
Liver	[C] Monocrotaline	0.3 (0.3 - 1.2)	0.6 (± 0.39)	1 (0.3 - 1.29)	0.75 (± 0.4)	1 (0.3 - 1)	0.86 (± 0.31)
Liver	[C] N-methylformamide (NMF)	0.3 (0.3 - 1.53)	0.54 (± 0.47)	1.16 (0.33 - 1.99)	1.07 (± 0.55)	1 (1 - 1)	1 (± 0)
Liver	[D] Phalloidin (chronic)	0.3 (0.1 - 0.53)	0.28 (± 0.15)	0.17 (0.02 - 0.34)	0.17 (± 0.1)	0.72 (0.65 - 1.04)	0.77 (± 0.16)
Liver	[E] Phenyl diisothiocyanate	0.53 (0.41 - 0.73)	0.56 (± 0.11)	0.88 (0.56 - 1.38)	0.9 (± 0.24)	0.76 (0.51 - 1.11)	0.81 (± 0.24)
Liver	[E] Phenyl isothiocyanate	1.12 (0.27 - 3.4)	1.69 (± 1.26)	0.44 (0.33 - 4.14)	0.83 (± 1.17)	0.54 (0.34 - 0.79)	0.54 (± 0.18)
Liver	[B] Retinyl palmitate	1.4 (1.04 - 1.87)	1.42 (± 0.29)	1.31 (1 - 2.08)	1.48 (± 0.4)	1.27 (0.59 - 1.5)	1.06 (± 0.42)
Liver	[B] Sodium Valproate	1.57 (0.92 - 2.13)	1.56 (± 0.38)	1.47 (0.68 - 2.82)	1.5 (± 0.64)	1.08 (0.85 - 2.09)	1.29 (± 0.51)
Liver	[C] a-Naphthylisothiocyanate (ANIT)	0.99 (0.3 - 2.11)	1.02 (± 0.64)	0.68 (0.3 - 3)	0.88 (± 0.84)	0.3 (0.3 - 1)	0.47 (± 0.3)
Kidney	[D] 2-Bromophenol	0.42 (0.26 - 0.69)	0.44 (± 0.14)	0.45 (0.28 - 0.85)	0.5 (± 0.15)	0.87 (0.63 - 0.93)	0.81 (± 0.13)
Kidney	[E] 3,5-Dichloroaniline hydrochloride	0.53 (0.27 - 0.94)	0.6 (± 0.25)	0.51 (0.32 - 0.97)	0.6 (± 0.24)	0.57 (0.42 - 0.72)	0.59 (± 0.11)
Kidney	[E] Atractyloside	1.43 (0.29 - 2.42)	1.44 (± 0.62)	0.97 (0.17 - 2.86)	1.06 (± 0.82)	0.5 (0.33 - 0.98)	0.56 (± 0.27)
Kidney	[D] Bromoethylamine hydrobromide	1.61 (0.94 - 2.76)	1.65 (± 0.54)	0.86 (0.46 - 1.26)	0.79 (± 0.26)	0.3 (0.15 - 0.55)	0.36 (± 0.17)
Kidney	[D] Cephaloridine	0.24 (0.16 - 3.16)	0.54 (± 0.92)	0.37 (0.18 - 2.85)	0.74 (± 0.85)	0.42 (0.26 - 2.07)	0.75 (± 0.75)

Target organ	Toxin	24 h post dose		48 h post dose		168 h post dose	
		Median (min - max)	Mean (± SD)	Median (min - max)	Mean (± SD)	Median (min - max)	Mean (± SD)
Kidney	[B] Chlorethanamine	1.7 (1.11 - 2.43)	1.71 (± 0.41)	1.79 (0.61 - 2.49)	1.7 (± 0.6)	0.57 (0.38 - 0.82)	0.6 (± 0.17)
Kidney	[A] Cisplatin	0.38 (0.28 - 0.79)	0.44 (± 0.17)	0.84 (0.54 - 0.96)	0.8 (± 0.12)	0.58 (0.36 - 1.95)	0.79 (± 0.65)
Kidney	[A] D-limonene (chronic)	0.74 (0.44 - 1.04)	0.75 (± 0.21)	0.65 (0.48 - 0.85)	0.66 (± 0.12)	0.76 (0.6 - 0.88)	0.75 (± 0.11)
Kidney	[E] Dichlorophenyl succinimide	0.86 (0.52 - 1.14)	0.84 (± 0.23)	0.5 (0.33 - 1.08)	0.58 (± 0.22)	0.84 (0.44 - 0.98)	0.78 (± 0.21)
Kidney	[D] Ethylene glycol	0.33 (0.16 - 0.86)	0.39 (± 0.2)	0.43 (0.23 - 1.88)	0.59 (± 0.48)	0.86 (0.42 - 2.24)	1.04 (± 0.7)
Kidney	[A] Folic acid	0.38 (0.2 - 3.3)	0.68 (± 0.93)	0.21 (0.08 - 0.83)	0.31 (± 0.24)	1.29 (0.69 - 1.56)	1.15 (± 0.35)
Kidney	[A] Gentamicin	0.59 (0.38 - 0.96)	0.6 (± 0.18)	0.56 (0.36 - 0.9)	0.58 (± 0.15)	0.47 (0.35 - 0.89)	0.53 (± 0.22)
Kidney	[B] Maleic acid	1.45 (0.72 - 4.13)	2.08 (± 1.21)	1.54 (0.71 - 2.8)	1.71 (± 0.7)	0.92 (0.75 - 1.39)	1 (± 0.25)
Kidney	[A] N-phenylanthranilic acid (chronic)	0.9 (0.36 - 1.96)	0.97 (± 0.58)	0.29 (0.15 - 1.45)	0.52 (± 0.5)	0.46 (0.21 - 1.56)	0.65 (± 0.51)
Kidney	[D] Para-aminophenol	3.63 (1.4 - 7.4)	4.27 (± 2.22)	1.23 (0.71 - 4.05)	1.71 (± 1.13)	0.38 (0.3 - 0.71)	0.45 (± 0.16)
Kidney	[A] Puromycin	0.26 (0.11 - 0.42)	0.25 (± 0.13)	0.26 (0.19 - 0.46)	0.28 (± 0.09)	1.36 (0.53 - 11.25)	3.2 (± 4.52)
Kidney	[B] Vancomycin hydrochloride	1.02 (0.46 - 1.44)	1 (± 0.3)	0.39 (0.27 - 0.58)	0.41 (± 0.11)	0.9 (0.87 - 1.17)	0.98 (± 0.13)
Liver & Kidney	[E] Acetaminophen	0.92 (0.39 - 2.33)	0.99 (± 0.52)	0.82 (0.53 - 1.23)	0.81 (± 0.22)	0.71 (0.62 - 0.96)	0.75 (± 0.13)
Liver & Kidney	[B] Aurothiomalate	5.18 (0.26 - 8.1)	4.8 (± 2.68)	2.91 (0.18 - 6.51)	3.12 (± 2.03)	0.69 (0.23 - 0.92)	0.6 (± 0.31)
Liver & Kidney	[C] Chloroform	0.35 (0.3 - 1.66)	0.56 (± 0.44)	0.3 (0.3 - 3)	0.61 (± 0.85)	0.3 (0.3 - 0.3)	0.3 (± 0)
Liver & Kidney	[D] Cyclosporin	0.15 (0.1 - 0.29)	0.17 (± 0.06)	0.22 (0.12 - 0.74)	0.28 (± 0.19)	0.17 (0.15 - 0.68)	0.27 (± 0.23)
Liver & Kidney	[D] Dichlorobenzene	0.3 (0.14 - 0.43)	0.29 (± 0.1)	0.22 (0.16 - 0.38)	0.24 (± 0.07)	0.43 (0.3 - 2.22)	0.79 (± 0.81)
Liver & Kidney	[C] Ethionine	0.3 (0.29 - 1)	0.43 (± 0.26)	0.3 (0.3 - 1.71)	0.64 (± 0.56)	1 (1 - 1)	1 (± 0)
Liver & Kidney	[B] Hexachlorobutadiene (HCBD)	2.5 (1.28 - 4.76)	2.71 (± 0.99)	0.87 (0.34 - 2.12)	1.06 (± 0.58)	0.77 (0.28 - 1.6)	0.84 (± 0.51)
Liver & Kidney	[B] Mercuric chloride	2.56 (0.95 - 5.62)	2.85 (± 1.35)	0.9 (0.2 - 2.02)	0.99 (± 0.58)	0.48 (0.12 - 0.81)	0.46 (± 0.3)
Liver & Kidney	[E] Microcystin-LR	2.49 (0.22 - 4.06)	2.21 (± 1.42)	1.09 (0.24 - 4.78)	1.95 (± 1.76)	0.51 (0.13 - 0.73)	0.46 (± 0.24)

Target organ	Toxin	24 h post dose		48 h post dose		168 h post dose	
		**Median (min - max)**	**Mean (± SD)**	**Median (min - max)**	**Mean (± SD)**	**Median (min - max)**	**Mean (± SD)**
Liver & Kidney	[E] Rotenone	0.58 (0.13 - 1.42)	0.73 (± 0.52)	0.58 (0 - 1.05)	0.57 (± 0.3)	0.53 (0.31 - 1.11)	0.59 (± 0.33)
Liver & Kidney	[E] S-(1,2-dichlorovinyl)-cysteine (DCVC)	1.15 (0.48 - 3.52)	1.47 (± 0.99)	0.69 (0.45 - 2.76)	1.11 (± 0.8)	0.7 (0.62 - 0.8)	0.72 (± 0.08)
Liver & Kidney	[D] Thioacetamide	0.18 (0.08 - 0.24)	0.16 (± 0.05)	0.44 (0.12 - 1.78)	0.55 (± 0.45)	0.74 (0.45 - 0.77)	0.66 (± 0.14)
Pancreas	[E] 1-Cyano-2-hydroxy-3-butene	0.56 (0.36 - 3.68)	0.91 (± 0.99)	0.62 (0.35 - 0.97)	0.64 (± 0.21)	0.3 (0.12 - 0.62)	0.35 (± 0.23)
Pancreas	[C] Caerulin	1 (0.3 - 3)	0.92 (± 0.81)	1 (0.3 - 1)	0.9 (± 0.23)	1 (0.3 - 3)	1.25 (± 1.02)
Pancreas	[E] L-arginine	1.08 (0.63 - 2.07)	1.13 (± 0.42)	1.35 (0.47 - 3.1)	1.38 (± 0.67)	0.33 (0.11 - 0.87)	0.4 (± 0.24)
Pancreas	[B] Streptozotocin	0.22 (0.07 - 1.34)	0.41 (± 0.39)	0.14 (0.05 - 1.29)	0.24 (± 0.38)	0.04 (0.03 - 0.15)	0.06 (± 0.05)
Testicular	[D] 1,3-Dinitrobenzene	0.72 (0.07 - 1.12)	0.66 (± 0.31)	0.41 (0.08 - 0.96)	0.48 (± 0.29)	0.7 (0.41 - 0.96)	0.72 (± 0.21)
Testicular	[C] Cadmium chloride	0.38 (0.3 - 1)	0.56 (± 0.32)	0.3 (0.28 - 1)	0.44 (± 0.3)	0.3 (0.3 - 0.3)	0.3 (± 0)
Testicular	[D] Cadmium chloride	0.22 (0.14 - 0.44)	0.24 (± 0.1)	0.16 (0.1 - 0.53)	0.23 (± 0.14)	0.1 (0.03 - 0.21)	0.1 (± 0.08)
Testicular	[D] Carbendazim	1.29 (0.77 - 1.76)	1.27 (± 0.43)	0.99 (0.72 - 1.92)	1.16 (± 0.42)	0.89 (0.67 - 1.92)	1.02 (± 0.51)
Testicular	[D] Di-n-pentyl-phthalate	0.97 (0.78 - 1.99)	1.12 (± 0.37)	0.79 (0.6 - 1.62)	0.96 (± 0.37)	0.6 (0.51 - 1.14)	0.69 (± 0.26)
Testicular	[D] Ethane dimethane sulfonate (EDS)	0.17 (0.07 - 0.75)	0.28 (± 0.24)	0.84 (0.26 - 1.42)	0.83 (± 0.47)	0.98 (0.39 - 1.75)	1.01 (± 0.54)
Testicular	[D] Methoxyacetic acid	0.47 (0.34 - 0.92)	0.55 (± 0.19)	0.53 (0.39 - 0.83)	0.56 (± 0.14)	1 (0.51 - 1.03)	0.9 (± 0.22)
Multiple organ	[B] Adriamycin	0.25 (0.07 - 0.73)	0.29 (± 0.2)	0.5 (0.1 - 1.03)	0.46 (± 0.31)	0.17 (0.16 - 3.1)	0.99 (± 1.29)
Multiple organ	[C] Amphotericin B	1 (0.3 - 1)	0.79 (± 0.34)	1 (0.3 - 1.1)	0.8 (± 0.34)	1 (0.3 - 1)	0.72 (± 0.38)
Multiple organ	[C] Azaserine	0.3 (0.21 - 1)	0.44 (± 0.3)	1 (0.3 - 3)	1.46 (± 1.1)	1 (0.3 - 3)	1.26 (± 1.02)
Multiple organ	[A] Dexamethasone	2.67 (0.91 - 8.6)	3.04 (± 2.22)	4 (2.1 - 14)	4.74 (± 3.43)	0.78 (0.3 - 1.88)	0.95 (± 0.66)
Multiple organ	[E] Mitomycin-C	0.67 (0.4 - 1.24)	0.74 (± 0.28)	0.8 (0.48 - 1.93)	0.98 (± 0.48)	0.82 (0.55 - 1.33)	0.87 (± 0.28)
Physiological stressor	[C] 1,1-Dichloroethylene & maleic acid	1 (0.3 - 1)	0.86 (± 0.3)	1 (0.3 - 1)	0.82 (± 0.3)	1 (1 - 3)	1.4 (± 0.89)
Physiological stressor	[C] 2,4-Dinitrophenol	0.3 (0.3 - 1)	0.37 (± 0.22)	0.93 (0.3 - 1)	0.71 (± 0.35)	1 (1 - 1)	1 (± 0)

Target organ	Toxin	24 h post dose		48 h post dose		168 h post dose	
		Median (min - max)	Mean (± SD)	Median (min - max)	Mean (± SD)	Median (min - max)	Mean (± SD)
Physiological stressor	[B] 4-Pentenoic acid	1.18 (1.02 - 1.89)	1.27 (± 0.25)	0.96 (0.41 - 2.21)	1.07 (± 0.5)	1.25 (0.84 - 1.43)	1.19 (± 0.22)
Physiological stressor	[D] Acetazolamide	0.29 (0.2 - 0.73)	0.32 (± 0.16)	0.48 (0.32 - 0.93)	0.49 (± 0.19)	0.7 (0.64 - 0.93)	0.77 (± 0.13)
Physiological stressor	[C] Acivicin	1 (0.3 - 3)	0.92 (± 0.81)	1 (1 - 1)	1 (± 0)	1 (1 - 1)	1 (± 0)
Physiological stressor	[E] Ammonium chloride	0.71 (0.52 - 0.97)	0.72 (± 0.16)	0.92 (0.75 - 1.32)	1 (± 0.22)	0.76 (0.7 - 1.11)	0.83 (± 0.17)
Physiological stressor	[D] Carboplatin	0.25 (0.08 - 0.32)	0.22 (± 0.1)	0.4 (0.23 - 0.66)	0.39 (± 0.12)	0.8 (0.6 - 1.2)	0.83 (± 0.23)
Physiological stressor	[A] Choline and choline/methionine deficiency (chronic)	0.34 (0.16 - 0.98)	0.39 (± 0.24)	0.27 (0.17 - 0.43)	0.27 (± 0.07)	0.42 (0.25 - 0.57)	0.42 (± 0.11)
Physiological stressor	[B] Food restriction (chronic)	0.25 (0.16 - 0.75)	0.31 (± 0.19)	0.27 (0.12 - 1.44)	0.57 (± 0.52)	0.25 (0.24 - 0.52)	0.32 (± 0.12)
Physiological stressor	[D] Furosemide	0.31 (0.22 - 0.65)	0.35 (± 0.13)	0.38 (0.33 - 1.4)	0.52 (± 0.33)	0.66 (0.61 - 0.94)	0.72 (± 0.14)
Physiological stressor	[B] Insulin	0.81 (0.47 - 1.48)	0.88 (± 0.35)	0.74 (0.39 - 1.37)	0.8 (± 0.33)	1.07 (0.56 - 1.88)	1.2 (± 0.61)
Physiological stressor	[E] Methotrexate	0.66 (0.36 - 0.82)	0.62 (± 0.16)	0.86 (0.39 - 1.23)	0.81 (± 0.24)	0.91 (0.78 - 1.23)	0.95 (± 0.17)
Physiological stressor	[A] Partial hepatectomy	0.22 (0.18 - 0.35)	0.23 (± 0.05)	0.22 (0.12 - 0.35)	0.22 (± 0.07)	0.27 (0.23 - 0.42)	0.31 (± 0.08)
Physiological stressor	[A] Phenobarbital (chronic)	0.65 (0.43 - 0.95)	0.67 (± 0.18)	0.59 (0.23 - 0.86)	0.57 (± 0.22)	0.71 (0.42 - 1.78)	0.87 (± 0.55)
Physiological stressor	[A] Pregnenolone 16 alpha carbonitrile (chronic)	0.52 (0.16 - 0.92)	0.55 (± 0.23)	0.48 (0.23 - 0.94)	0.5 (± 0.23)	0.58 (0.32 - 1.04)	0.61 (± 0.26)
Physiological stressor	[A] Probenecid	0.91 (0.27 - 2.21)	0.93 (± 0.51)	0.87 (0.56 - 1.34)	0.9 (± 0.3)	1.09 (0.91 - 1.69)	1.17 (± 0.31)
Physiological stressor	[C] Rosiglitazone	0.3 (0.3 - 1)	0.53 (± 0.33)	1 (0.3 - 1)	0.86 (± 0.3)	1 (0.3 - 1)	0.72 (± 0.38)
Physiological stressor	[C] Rosiglitazone (chronic)	1 (0.3 - 1)	0.86 (± 0.29)	1 (0.3 - 3)	1.33 (± 0.91)	1 (0.98 - 1)	1 (± 0.01)
Physiological stressor	[E] Sodium bicarbonate	0.3 (0.17 - 0.63)	0.32 (± 0.14)	0.35 (0.23 - 0.58)	0.39 (± 0.11)	0.36 (0.31 - 1.24)	0.5 (± 0.29)
Physiological stressor	[A] Unilateral nephrectomy	0.76 (0.42 - 1.09)	0.74 (± 0.22)	0.54 (0.21 - 0.94)	0.58 (± 0.22)	0.65 (0.34 - 1.14)	0.66 (± 0.3)
Physiological stressor	[B] Water deprivation (chronic)	1.85 (0.89 - 2.1)	1.74 (± 0.35)	2.62 (1 - 2.69)	2.33 (± 0.6)	1.24 (0.75 - 1.59)	1.18 (± 0.29)
No Effect	[E] Acetaminophen (chronic)	0.47 (0.25 - 0.69)	0.45 (± 0.14)	0.55 (0.3 - 0.79)	0.55 (± 0.14)	0.84 (0.49 - 1.33)	0.9 (± 0.33)
No Effect	[C] Buthionine sulphoxime	1 (0.3 - 1)	0.72 (± 0.36)	1 (0.3 - 1)	0.72 (± 0.36)	1 (0.3 - 1)	0.72 (± 0.38)

Target organ	Toxin	24 h post dose		48 h post dose		168 h post dose	
		Median (min - max)	Mean (± SD)	Median (min - max)	Mean (± SD)	Median (min - max)	Mean (± SD)
No Effect	[C] Ferrous sulphate	0.3 (0.3 - 1)	0.58 (± 0.36)	0.3 (0.3 - 1)	0.58 (± 0.36)	1 (1 - 1)	1 (± 0)
No Effect	[B] Ifosfamide	0.64 (0.18 - 3.64)	1.27 (± 1.24)	1.78 (0.54 - 3.64)	1.87 (± 1.1)	1.03 (0.63 - 1.33)	1.01 (± 0.28)
No Effect	[B] Lithocholic acid	1.03 (0.55 - 1.32)	0.98 (± 0.28)	0.99 (0.49 - 1.14)	0.88 (± 0.26)	1.11 (0.69 - 1.47)	1.06 (± 0.33)
No Effect	[E] Paraquat	0.54 (0.29 - 0.72)	0.54 (± 0.15)	0.58 (0.36 - 0.91)	0.59 (± 0.18)	0.88 (0.6 - 1.01)	0.84 (± 0.16)
No Effect	[D] Potassium dichromate	0.31 (0.12 - 0.61)	0.3 (± 0.15)	0.33 (0.18 - 0.96)	0.39 (± 0.23)	0.59 (0.22 - 0.87)	0.54 (± 0.25)
No Effect	[C] Trichlorethylene	0.3 (0.3 - 1)	0.44 (± 0.3)	1 (0.3 - 1)	0.86 (± 0.3)	1 (1 - 1)	1 (± 0)

A-F: Indicates Pharmaceutical Company & sample origin
